# Adolescent Addiction Curriculum: Impact on Knowledge Self-Assessment in Pediatric Learners

**DOI:** 10.15766/mep_2374-8265.10716

**Published:** 2018-05-07

**Authors:** Edore Onigu-Otite, Daryl Shorter

**Affiliations:** 1 Assistant Professor, Menninger Department of Psychiatry and Behavioral Sciences, Baylor College of Medicine

**Keywords:** Curriculum, Adolescent, Adolescence, Substance-Related Disorders, Addiction, Mental Health, Graduate Medical Education, Alcohol, Physician-in-Training, Substance Use

## Abstract

**Introduction:**

Addiction is developmentally a pediatric-onset disease. Adolescent addiction recently gained the nation's attention due to the steep increase in opioid-related drug overdose deaths. Educating future adolescent health providers on adolescent addiction is a strategic initiative to mitigate the impact of this challenging public health concern.

**Methods:**

We used a logic model worksheet to identify key target areas informing the curriculum content development. The curriculum was written to be delivered in three successive parts—the Science of Addiction, Adolescence and Addiction, and Diagnosis and Treatment—each within a 2-hour interactive lecture session using PowerPoint presentations, brief videos, and learner activities. We collected data using pre- and postsession self-evaluation questionnaires. We calculated mean differences in scores and obtained qualitative data from learner comments.

**Results:**

Sessions were well received by attendees. A total of 31 participants attended at least one session. Knowledge of adolescent addiction increased in each session, with the greatest increase in the Science of Addiction (1.6, *p* = .0011), followed by Diagnosis and Treatment (1.1, *p* < .0001) and Adolescence and Addiction (0.9, *p* < .0001).

**Discussion:**

Attendance at one or more sessions improved participants' addiction-related knowledge. Graduate medical training programs can provide adolescent addiction education using systematic curricula such as this. Furthermore, this curriculum can be adapted to suit different groups of learners.

## Educational Objectives

By the end of this activity, learners will be able to:

1. Define an addictive disorder.
2. Articulate the disease model of addiction.
3. Describe the neurobiology of addiction and common substances of abuse/misuse.
4. Discuss the link between pain and addiction.
5. Recognize signs and symptoms of addictive disorders in adolescents.
6. Screen for substance use in adolescents.
7. Identify risk and protective factors for addiction in adolescents.
8. Recognize common comorbid mental health conditions in adolescent addictive disorders.
9. Explain the principles of treatment of adolescent addictive disorders.
10. Conduct a brief motivational intervention.

## Introduction

Addictive disorders include substance-related and non-substance-related disorders. Developmentally, substance use disorders (SUDs) are diseases of pediatric onset. Among adult patients admitted to drug treatment facilities, the majority, 74%, reported they initiated use of substances before the age of 18 years.^1^ Despite these findings, adolescence is often overlooked as an opportunity for early identification and intervention in treatment of SUDs.

Recently, adolescent SUDs have gained even greater publicity due to increasing rates of opioid use disorder and fatal outcomes, such as overdose deaths.^2^ According to the Treatment Episode Data Set Report, in 2013, 21,000 adolescents had used heroin in the past year.^1,3^ Additionally, the rate of drug overdose deaths involving heroin among adolescents ages 15–19 increased during the 1999–2015 period and was three times higher in 2015 than in 1999.^4^ Altogether, these increases emphasize the critical need for continued age-targeted efforts to prevent or delay substance use initiation, to detect initiation early, and to intervene in a timely manner should it occur. Future adolescent health providers must be better trained to deal with this group of disorders, especially when early symptoms begin to emerge.

There remains a profound discrepancy between the significant public health problem of SUDs and the training of future health providers.^5^ Relatively few educational resources have been published in this field of study.^6^ This deficiency is only steeper for graduate medical education learners, who often have no resources to prepare the future pediatric health provider. A PubMed and *MedEdPORTAL* search of the literature revealed no comprehensive adolescent addiction curricula for pediatric learners. Stigma is often cited as an additional impediment to education in the care of patients with SUDs.^7^ With increasing pressure on primary care physicians to treat and advocate for adolescents with SUDs, it is imperative that physicians providing care for adolescent patients have the knowledge and skills to appropriately address this segment of the population.^8^

This curriculum is intended to provide a foundational knowledge base for future adolescent primary care providers, who often encounter the first signs and symptoms of emerging SUDs. Lecture-style teaching methodology is a time-efficient way of delivering large amounts of comprehensive educational material within a short time to a large group of learners. Furthermore, there is also evidence that medical learners prefer interactive lectures and problem-based learning as teaching methodologies.^9^ This educational experience was conducted in a series of three 2-hour sessions of interactive lecture-style format. It is thought that in this format, this curriculum can be more readily delivered to pediatric trainees, as well as being potentially adaptable to other groups of learners.

## Methods

This curriculum was developed as a project during the Master Teachers Fellowship Program in the Office of Faculty Development of Baylor College of Medicine. The study was approved by the institutional review board. The target learners are intermediate and advanced graduate medical trainees who are anticipated to provide care for children and adolescents in their professional life. The authors met with pediatric education training directors to gain further insight into practical educational needs. A logic model worksheet was completed. Availability of both faculty and learners was taken into consideration. The curriculum was tailored to the available time and space. The immediate goal was specifically to increase knowledge and awareness of adolescent addictive disorders in future pediatric providers. An interim goal was to make more learners open to, and comfortable with, interacting with adolescents with addictive disorders. The ultimate goal was to facilitate future pediatric providers becoming comfortable with dealing with adolescents with addictive disorders. Information on adolescence and addiction science was reviewed from multiple sources, including texts, reports from national population surveys and studies, relevant journal articles, and updated information from National Institutes of Health–supported resources.

**Figure 1.**
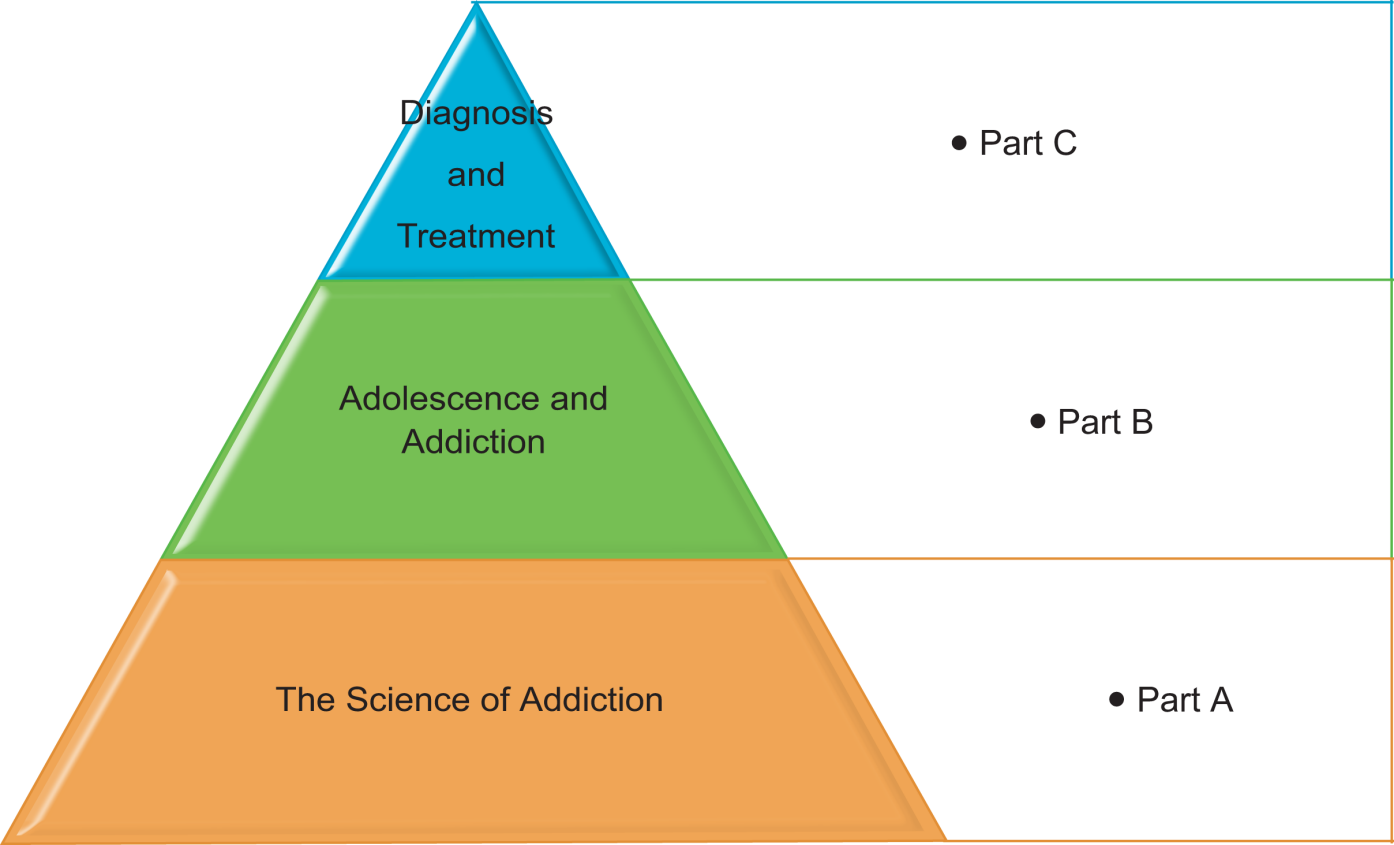
Three-part stepwise curriculum.

This curriculum was written to be given in three successive didactic sessions utilizing an interactive lecture-style format (Figure 1). Each session was organized to be delivered over 2 hours with two breaks, the first for 10 minutes and the second for 5 minutes. Lectures consisted of PowerPoint presentations, short videos, and case-based discussions. Prior to each session, self-assessment and session evaluation forms were distributed to learners.

For session 1, the Science of Addiction, the goal was to introduce learners to addictive disorders and commonly used substances in adolescents. A lecture plan (Appendix A) and detailed instructor notes (Appendix B) were provided. Learner self-assessment forms Appendix D were distributed before the session. The definitions of addictive disorders as currently defined by the American Society of Addiction Medicine and the American Psychiatric Association were reviewed. Scientific information obtained from reputable resources including the National Institute of Drug Abuse, Monitoring the Future, the Substance Abuse and Mental Health Administration, and the Centers for Disease Control and Prevention, many of which are also supported by the National Institutes of Health, was presented. Consequently, the eventual content was derived from multiple sources and included useful visuals such as infographics and short videos (Appendix C).

For session 2, Adolescence and Addiction, the goal was to provide information on adolescence and addiction within a bilateral developmental framework. A lecture plan Appendix E and detailed instructor notes Appendix F were provided. Learner self-assessment forms Appendix H were distributed before the session. The session presentation Appendix G included information aimed at increasing attention to adolescence as a prime time for the onset of addictive disorders and recognition of the process of development of an addictive disorder. This session also included a section on alcohol use in adolescence, which reviewed the basic neurobiology of alcohol along with public health and safety concerns. For this portion, content was obtained from an additional source, the National Institute on Alcohol Abuse and Alcoholism. Two adolescent outpatient cases derived from true clinic cases were processed, with a focus on having the learners identify risk and protective factors as they impacted the development of the addictive disorder along the adolescent's developmental trajectory. Learners also gained an understanding of the process of development of the addictive disorder and the positive impact that adequate treatment of underlying psychiatric and medical conditions has on adolescent addictive disorders (Appendices I & J).

For session 3, Diagnosis and Treatment, the goal was to facilitate early identification of adolescents with addictive disorders, provide information on the principles of treatment, and review brief interventions available for use in office settings. A lecture plan Appendix K and detailed instructor notes Appendix L were provided. Learner self-assessment forms Appendix N were distributed before the session. In the session presentation Appendix M, the diagnosis of addictive disorders was reviewed. Clinical assessment of the adolescent with an addictive disorder was presented, current evidence-based screening tools were provided, and drug testing in the adolescent was reviewed. A short section on internet gaming disorder was included because pediatric electronic gaming addiction with functional impairment that is sometimes comparable to SUDs is on the rise. For treatment, although most medications are not approved for use in the pediatric population, the currently available FDA-approved medications were reviewed. Thus, learners were kept up to date on current treatments in the field of addiction, especially given that pediatric providers now sometimes provide medical care for young adults. Brief interventions that could be used in outpatient clinic visits and other short patient encounters were reviewed. A motivational interviewing section was included to provide learners with knowledge and practice, with the aim of increasing skills relevant to conducting a brief motivational intervention Appendix M. We did not include acute treatment of addiction such as detoxification from substances. The acuity and specialized hands-on training needed to cover this aspect of treatment of addictive disorders are beyond the scope of this curriculum.

In summary, the adolescent addiction curriculum involves three 2-hour sessions of PowerPoint slides, short videos, case examples, and learner activities that can be done in pairs or small groups. The curriculum was first given in September-November 2016. Participants completed pre- and postassessments at each session. Each self-assessment form consisted of 10 session-specific items assessed on a 5-point Likert scale. The self-assessment items and learner activities matched the session-specific learner objectives. Participants also completed session evaluations at the end of each didactic session. Mean differences between pre- and postscores were analyzed, while qualitative information was obtained from learner comments.

## Results

The sessions were given during dedicated didactic lecture time for adolescent medicine fellows and rotating pediatric residents. The sessions were held in a conference room. Each session was attended by nine to 11 learners. A total of 31 participants attended at least one session, including adolescent medicine fellows, pediatric residents, rotating medical students, pediatric faculty, and one psychology intern (Table). The three sessions were scheduled at 1- and 2-month intervals. Due to changing fellow and resident rotation schedules, few participants could attend two or three sessions. Out of professional interest, some faculty attended the sessions and provided highly valuable feedback that was used to inform future sessions.

**Table.** Learner Identification and Number of Attendees

	Number of Attendees		
Learner Identification	Session 1	Session 2	Session 3
Adolescent medicine fellow	5	4	3
Pediatric resident	1	2	2
Psychology intern	1	0	0
Faculty	2	2	2
Learner did not identify	0	4	3
Total	9	12	10

**Figure 2.**
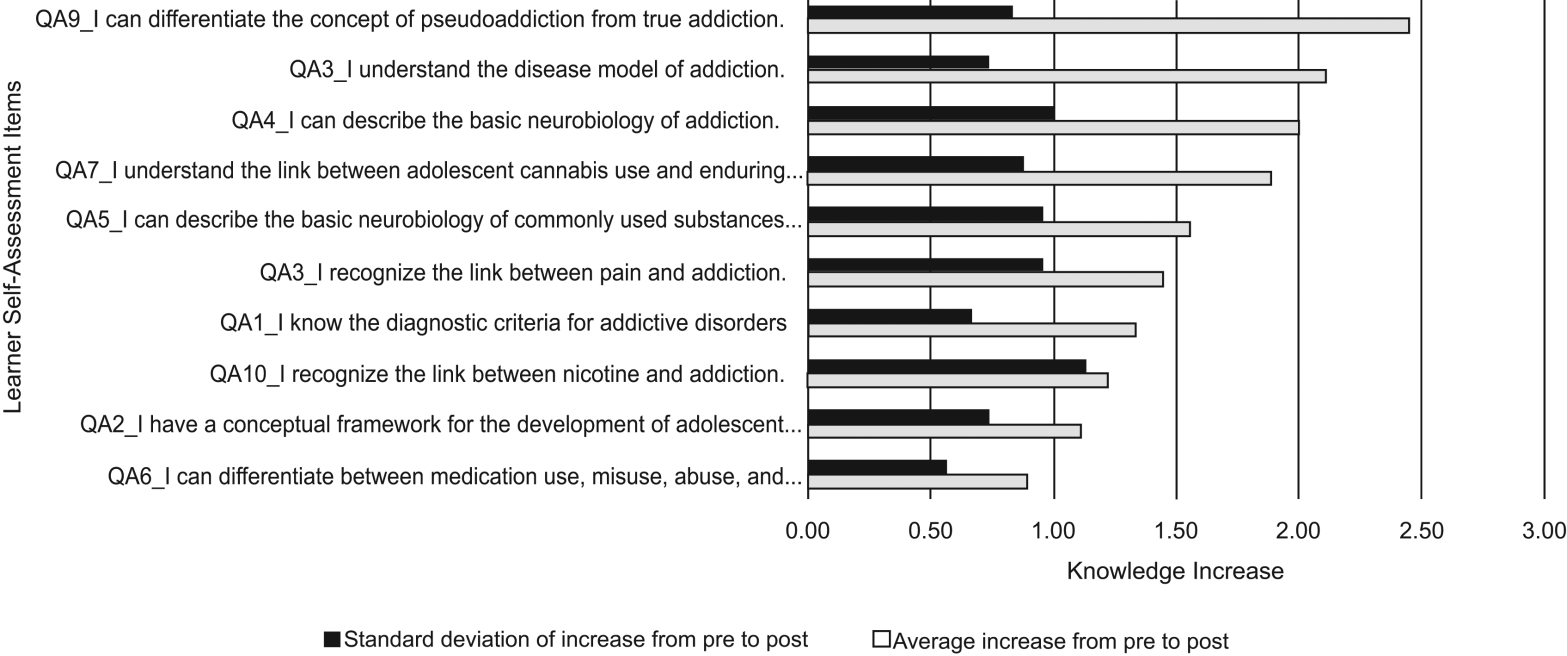
Learner self-assessment scores for session 1 (the Science of Addiction).

**Figure 3.**
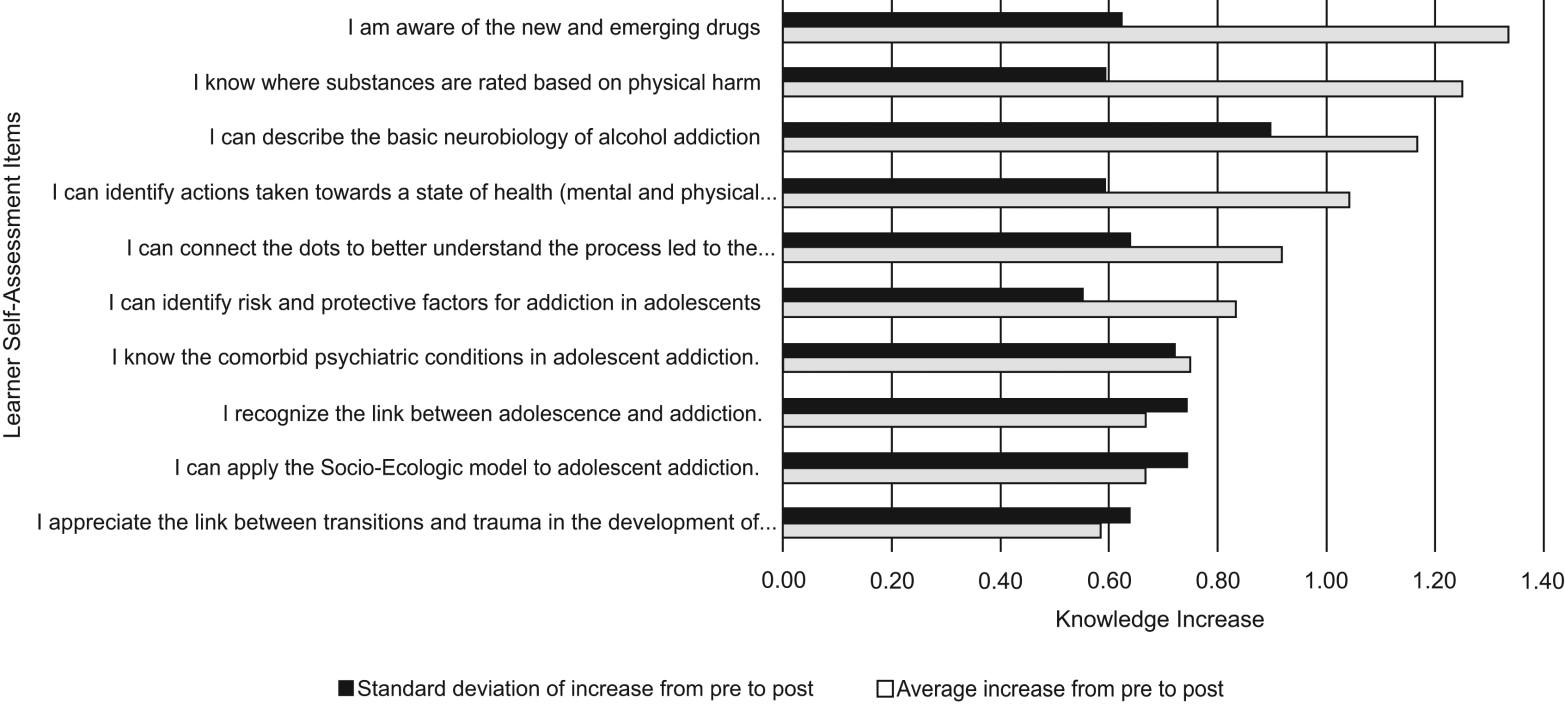
Learner self-assessment scores for session 2 (Adolescence and Addiction).

**Figure 4.**
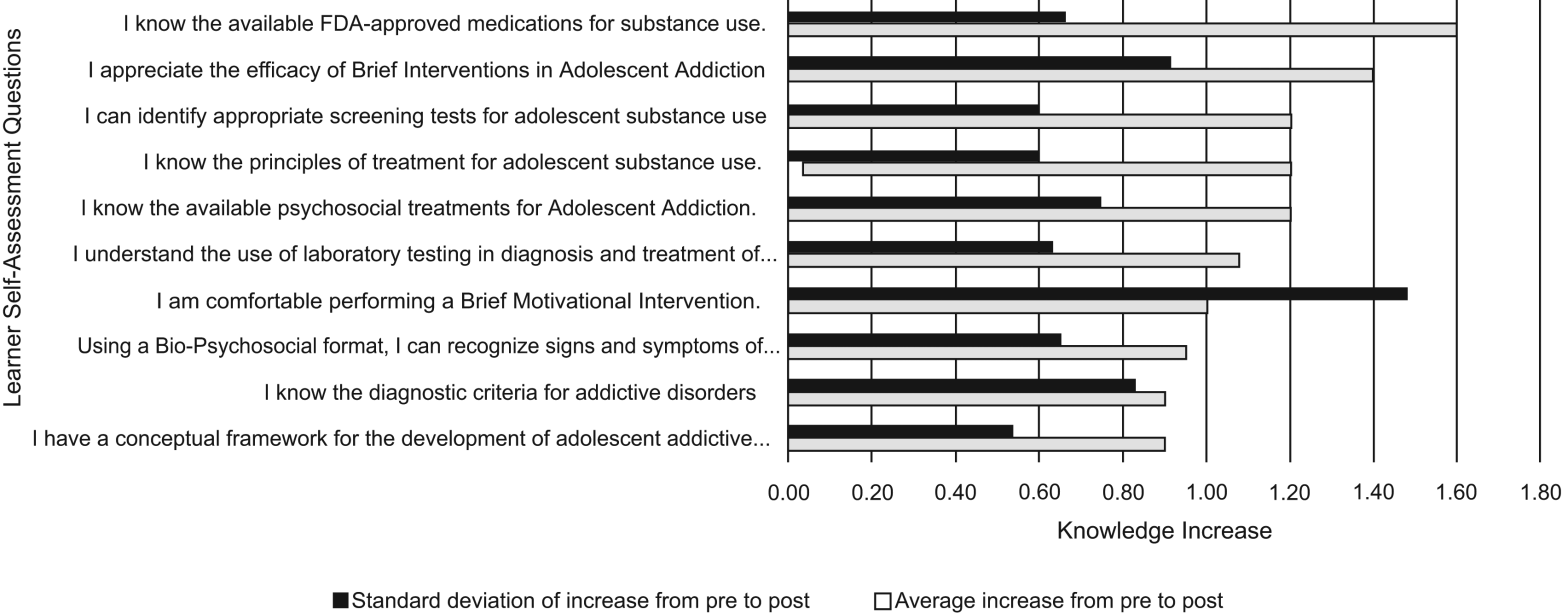
Learner self-assessment scores for session 3 (Diagnosis and Treatment).

Each session showed mean increases in knowledge, as depicted in Figure 2, Figure 3, and Figure 4. The greatest increase occurred in the Science of Addiction session (0.6, *SD* = 0.49; 95% confidence interval [CI], 1.29–1.91, *p* = .0011). In this session, knowledge increase was greatest in the concept of pseudoaddiction, understanding the disease model of addiction, and describing the basic neurobiology of addiction. The area of least knowledge increase was differentiating between medication use, misuse, abuse, and addiction, which was discussed as part of the pain and addiction subsection. The item that showed the most variation in knowledge increase was related to understanding the link between nicotine and addiction.

The session with the next greatest increase in knowledge was Diagnosis and Treatment (1.1, *SD* = 0.26; 95% CI, 0.76–1.09, *p* < .0001). In this session, the greatest knowledge increases were in awareness of new and emerging drugs, rating of substances based on addictive potential and physical harm, and basic neurobiology of alcohol. The area of least knowledge increase was in appreciation of the link between transitions and trauma in the development of adolescent addiction. The item that showed the most variation in knowledge increase was describing the basic neurobiology of alcohol.

Finally, the session that showed the least increase in knowledge was Adolescence and Addiction (0.9, *SD* = 0.23; 95% CI, 0.99–1.28, *p* < .0001). In this session, the greatest increases related to knowledge of the FDA-approved medications for SUDs, appreciation for the efficacy of brief motivational intervention, and identification of appropriate screening tests for SUDs in adolescents. The area of least knowledge increase was in obtaining a conceptual framework for the development of adolescent addictive disorders. The item that showed the greatest variation in knowledge in this session and overall was comfort level in conducting a brief motivational intervention.

Qualitative feedback showed that the sessions were well received. The learners enjoyed the sessions and identified various aspects of interest, including the neurobiology of addiction and an appreciation of the biological basis interpreted within the behavioral and psychosocial aspects of addiction. Participants expressed that this was high yield and were pleased to have obtained practical tips for identifying and intervening in clinical practice. Some learners said they would like to know more about how to approach a patient who was abusing or possibly addicted. Several learners noted that they would like to have more discussions on the topic.

## Discussion

This pilot study of an educational curriculum intervention intended to fill a gap in training regarding adolescent addictive disorders in graduate medical education learners resulted in knowledge increase in vital areas. The greatest knowledge increase was in the Science of Addiction session, which covered the medical foundation of addictive disorders. The least knowledge increase was in the Adolescence and Addiction session. This may reflect swifter assimilation of fact-based knowledge when compared to content involving conceptualizing disease process. It might also signify the need for more time to adequately work through this type of learning and could be addressed by including more case-based learning or by utilizing the flipped classroom approach for this portion of the curriculum. In the three sessions, the themes common to areas of least knowledge increase related to processes of development of adolescent addictive disorders. In practical reality, these disorders are multicomplex developmental processes that require a deeper level of knowledge and hands-on training and experience than a curriculum such as this can aspire to provide. Notably, due to within-group differences in attendee discipline, level of training, and prior exposure to adolescent addiction, changes in comfort level could not be realistically assessed across groups.

This adolescent addiction curriculum addresses an educational need for increasing knowledge of adolescent addiction among future adolescent medical providers. It was well received, was seen as valuable, and generated interest among attendees. Feedback indicated that participants would have liked additional time for more discussions on the topics provided in the curriculum. This desire can be met by including more patient case discussions. Discussions could focus on connecting the dots, such as reasoning backward to the antecedents of the symptoms, which would show successive stages in the development of the disease and highlight intervention points along the disease process. Such intervention potentially could have positively impacted the trajectory of the patient's illness. Importantly, incorporating this knowledge in practice would depend on the individual provider's comfort level. Although increasing learners' comfort with speaking with adolescents who engage in addictive behaviors was a target of the curriculum, it could not be adequately measured given the changing constitution of each session's attendees. The sessions could be adapted across medical education to better suit different groups of learners, including undergraduate, graduate, and continuing medical education, according to the needs of various disciplines.

Strengths of this curriculum include the focus on offering foundational concepts and frameworks upon which to conceptualize adolescent addictive disorders. These provide learners with a more comprehensive understanding of the adolescent patients they encounter, as well as ways to mitigate the onset or progression of the disorder. This curriculum can be easily implemented using standard graduate medical education program infrastructure, including didactic teaching time, which does not require added preparation or funding support.

There were several limitations experienced with this pilot project. Only a limited number of participants were able to attend more than one session. This was due in part to the large size of the training program and to rotational learner schedules in locations across a large metropolis. To facilitate attendance, sessions could be placed at the beginning of the day, prior to learners dispersing to attend to other duties. Alternatively, sessions could be transmitted by electronic media to learners at other locations who might be unable to be physically present at the main lecture location. Additionally, sessions can potentially be shortened to 1-hour time slots and scheduled to be given within a consecutive time block to facilitate successive development of knowledge. The combination of didactics to provide foundational knowledge balanced with experiential methods to facilitate skills development is a future direction for improvement on this aspect of provider training. Depending on the institution and the availability of instructors, a related clinical rotation could be included. When that is the case, if feasible, it is suggested that learners undergo this comprehensive curriculum prior to engaging in the clinical arena. Finally, additional faculty-supervised practice and training are required to more fully enhance the clinical implementation and comfort level of trainees encountering adolescents with addictions across various settings.

The field of adolescent addiction is a rapidly changing one. Despite information from national epidemiological studies (e.g., Monitoring the Future) that indicate impending concerns, predicting the future with accuracy is hardly possible. Therefore, it is best to equip practitioners with the principles of the disease in order to increase their readiness to provide developmentally appropriate, age-targeted clinical care. This curriculum fills a gap in educating future adolescent health providers and has been found to be effective in increasing addiction-related knowledge pertaining to adolescents. Additionally, it has the potential to be adapted to suit other groups of learners.

## Appendices

A. Addiction Session 1 Lecture Plan.docx
B. Addiction Session 1 Instructor Notes.docx
C. Addiction Session 1 Slides.pptx
D. Addiction Session 1 Self-Assessment.docx
E. Addiction Session 2 Lecture Plan.docx
F. Addiction Session 2 Instructor Notes.docx
G. Addiction Session 2 Slides.pptx
H. Addiction Session 2 Self-Assessment.docx
I. Addiction Session 2 Worksheets.docx
J. Addiction Session 2 Patient Case B.docx
K. Addiction Session 3 Lecture Plan.docx
L. Addiction Session 3 Instructor Notes.docx
M. Addiction Session 3 Slides.pptx
N. Addiction Session 3 Self-Assessment.docx All appendices are peer reviewed as integral parts of the Original Publication.
