# Supplementary material for: Adolescent Addiction Curriculum: Impact on Knowledge Self-Assessment in Pediatric Learners
Source: MedEdPORTAL. 2018 May 7;14:10716. doi: 10.15766/mep_2374-8265.10716 (PMC6342343; doi:10.15766/mep_2374-8265.10716)
Supplement: Supplementary file 1 — A. Addiction Session 1 Lecture Plan.docx B. Addiction Session 1 Instructor Notes.docx C. Addiction Session 1 Slides.pptx D. Addiction Session 1 Self-Assessment.docx E. Addiction Session 2 Lecture Plan.docx F. Addiction Session 2 Instructor Notes.docx G. Addiction Session 2 Slides.pptx H. Addiction Session 2 Self-Assessment.docx I. Addiction Session 2 Worksheets.docx J. Addiction Session 2 Patient Case B.docx K. Addiction Session 3 Lecture Plan.docx L. Addiction Session 3 Instructor Notes.docx M. Addiction Session 3 Slides.pptx N. Addiction Session 3 Self-Assessment.docx [file mep-14-10716-s001.zip › A._Addiction_Session_1_Lecture_Plan.docx]

**Adolescent Addiction Session 1 Lecture Plan**

**Title**: The Science of Addiction

**Description**: This lecture reviews the basics of the science of addiction including diagnostic criteria, substances of abuse among adolescents, and pain and addiction.

**Learning Objectives**:

By the end of this seminar, the participant will be able to:

1. Define an addictive disorder
2. Describe the symptoms of addictive disorders
3. Explain the disease model of addiction
4. Describe the neurobiology of addiction
5. Revise the most commonly used substances in adolescents
6. Describe the neurobiology of common substances of misuse and problematic use in adolescents.
7. Discuss the link between pain and addiction
8. Define pseudoaddiction
9. Distinguish between appropriate medication use, misuse, abuse, and addiction.

**Seminar Outline:**

1. Introduction
2. Neurobiology of Addiction
3. Diagnostic Criteria: DSM V
4. Substances of Abuse: DSM V, DEA
5. Neurobiology of commonly used substances
6. Pain and Addiction
7. Summary

**Recommended Reading**:

1. National Institute of Drug Abuse (NIDA) https://www.drugabuse.gov
2. Clinical Handbook of Adolescent Addiction Editor(s): Richard Rosner. Published Online: 4 DEC 2012. Print ISBN: 9780470972342. Online ISBN: 9781118340851. DOI: 10.1002/9781118340851
3. Siqueira LM and AAP Committee on Substance Use and Prevention. Nicotine and Tobacco as Substances of Abuse in Children and Adolescents. Pediatrics.2017;139(1):e2016343. Available at <http://pediatrics.aappublications.org/content/pediatrics/139/1/e20163436.full.pdf> (Accessed April 6, 2018)
4. The Impact of Marijuana Policies on Youth: Clinical, Research, and Legal Update. Committee on Substance Abuse, Committee on Adolescence. Pediatrics: 2015;135;584. DOI: 10.1542/peds.2014-4146. Available at <http://pediatrics.aappublications.org/content/pediatrics/135/3/584.full.pdf> (Accessed April 6, 2018)
5. The Neurobiology of Opioid Dependence: Implications for Treatment. Korsten, T.R., George, T.P. [Sci Pract Perspect](https://www.ncbi.nlm.nih.gov/pmc/articles/PMC2851054/). 2002 Jul; 1(1): 13–20.Available at <https://www.ncbi.nlm.nih.gov/pmc/articles/PMC2851054/pdf/spp-01-1-13.pdf> (Accessed April 6, 2018)
