# Supplementary material for: Adolescent Addiction Curriculum: Impact on Knowledge Self-Assessment in Pediatric Learners
Source: MedEdPORTAL. 2018 May 7;14:10716. doi: 10.15766/mep_2374-8265.10716 (PMC6342343; doi:10.15766/mep_2374-8265.10716)
Supplement: Supplementary file 1 — A. Addiction Session 1 Lecture Plan.docx B. Addiction Session 1 Instructor Notes.docx C. Addiction Session 1 Slides.pptx D. Addiction Session 1 Self-Assessment.docx E. Addiction Session 2 Lecture Plan.docx F. Addiction Session 2 Instructor Notes.docx G. Addiction Session 2 Slides.pptx H. Addiction Session 2 Self-Assessment.docx I. Addiction Session 2 Worksheets.docx J. Addiction Session 2 Patient Case B.docx K. Addiction Session 3 Lecture Plan.docx L. Addiction Session 3 Instructor Notes.docx M. Addiction Session 3 Slides.pptx N. Addiction Session 3 Self-Assessment.docx [file mep-14-10716-s001.zip › B._Addiction_Session_1_Instructor_Notes.docx]

**Adolescent Addiction Session 1 Instructor Notes**

**Learner Objectives, Activities, Notes to the Educator, and Corresponding PowerPoint Lecture Slides**

Session Title: The Science of Addiction

Learning Goal: To introduce addictive disorders in adolescents

|  | Learner Objective | Learner Activity | Instructor Lecture Points | PowerPoint Lecture Slides |
| --- | --- | --- | --- | --- |
|  | Review overall epidemiology of adolescent addiction (current and trends) |  | Highlight why this is important | A7 to A14 |
|  | Discuss the term "Addiction" | Q&A: Ask participants what they understand by the term “Addiction” | Examine the etymology of the term addiction. Inform learners that historically, the term “Addicere” was originally a commerce term which indicated that goods had changed hands, i.e. the handing over of goods from one individual to another. Modern day translations include “to hand over”, “to surrender” or “to devote to”, etc. | A15 |
|  |  |  | Review the current ASAM and APA definition of addiction | A16 |
|  | Explain the disease model of addiction | Q&A: Ask participants how the individual with addiction is commonly viewed in society and in clinics/hospitals where they work. | Summarize common models of addiction | A17 & 18 |
|  |  |  | Review the disease model of addiction | A19 – A22 |
|  | Revise the neurobiology of Addiction | Watch video titled   - Title: Brain Reward: Understanding How the Brain Responds to Natural Rewards and Drugs of Abuse - Authors: NIDA/NIH - Website: https://www.youtube.com/watch?v=7VUlKP4LDyQ (https://youtu.be/7VUlKP4LDyQ - Running Time: Running time: 9:11 mins (Slide A23)   Alternative Video Information   - Title: The Reward Circuit - Authors: NIDA/NIH - Website: https://www.youtube.com/watch?v=DMcmrP-BWGk&feature=youtu.be - Running Time: 1:48mins | Introduce the concept of Reward  Review the neurobiology of Addiction | A23 – 25  (Video A23) |
|  | Review the diagnostic criteria for Substance Use Disorders | Q&A: Ask participants from their experience how they knew a patient was addicted. | Discuss each diagnostic criterion giving examples | A26 to A38 |
|  | Review DEA Schedule of Substances |  | Discuss accessibility of these substances to adolescents | A39 and 40 |
|  | Review Other Substance Use Disorders and Non-Substance Related Addictive Disorders |  | Have participants understand that similar principles apply to non-substance use related disorders especially in terms of daily functional impairment. | A41 and A42 |
|  | BREAK | BREAK | BREAK | BREAK A43 |
|  | Revise the neurobiology of commonly used substances | Watch short video clip titled  “Animated infographic: Monitoring the Future 2015 Survey Results”  Running Time: 2:45mins (Slide A44) | Give overview of recent prescription, OTC, illicit drug use in adolescents | A44 |
|  |  |  | Discuss substances by class | A45 |
|  |  |  | Cannabis | A46-54 |
|  |  |  | Prescription Stimulants | A55 |
|  |  |  | Cocaine | A56 |
|  |  |  | Other Stimulants | A57 |
|  |  |  | Cough Medicines | A58 |
|  |  |  | Inhalants | A59 |
|  |  |  | Prescription Sedatives | A60 |
|  |  |  | Anabolic Steroids | A61 |
|  |  |  | Hallucinogens | A62 |
|  |  |  | Dissociative Anesthetics | A63 |
|  |  |  | Tobacco and Nicotine | A64 to 75 |
|  | BREAK | BREAK | BREAK | BREAK |
|  | Examine Opioids and Addiction | Watch infomercial titled ‘Prescription opioids even when prescribed by a doctor’  Running Time: 1:31mins (Slide A78) | Review Prescription Opioids and Addiction | A78 to 85  (Video A78) |
| 10. | Explore thinking around patient pain treatment (i.e. learners to explore their own thoughts about pain management) | Q&A: Ask participants two open-ended question:   1. What is pain? 2. When is pain adequately treated? 3. Allow for short discussion 4. Then take a vote giving 3 options. Pain is adequately treated: 5. When the patient feels happy 6. When the patient is comfortable 7. When the patient is functional | Facilitate brief discussion | A86 and A87 |
| 11. | Differentiate between Medication use, misuse, abuse, and addiction |  | Discuss this topic using patient examples | A88 to 91 |
| 12. | Formulate Take Home Points |  | Review summary slide | A92 |
| 13. | Take Questions |  |  | A93 |
| 14 | Provide Resources |  |  | A94 |
