# Supplementary material for: Adolescent Addiction Curriculum: Impact on Knowledge Self-Assessment in Pediatric Learners
Source: MedEdPORTAL. 2018 May 7;14:10716. doi: 10.15766/mep_2374-8265.10716 (PMC6342343; doi:10.15766/mep_2374-8265.10716)
Supplement: Supplementary file 1 — A. Addiction Session 1 Lecture Plan.docx B. Addiction Session 1 Instructor Notes.docx C. Addiction Session 1 Slides.pptx D. Addiction Session 1 Self-Assessment.docx E. Addiction Session 2 Lecture Plan.docx F. Addiction Session 2 Instructor Notes.docx G. Addiction Session 2 Slides.pptx H. Addiction Session 2 Self-Assessment.docx I. Addiction Session 2 Worksheets.docx J. Addiction Session 2 Patient Case B.docx K. Addiction Session 3 Lecture Plan.docx L. Addiction Session 3 Instructor Notes.docx M. Addiction Session 3 Slides.pptx N. Addiction Session 3 Self-Assessment.docx [file mep-14-10716-s001.zip › C._Addiction_Session_1_Slides.pptx]

## Slide 1
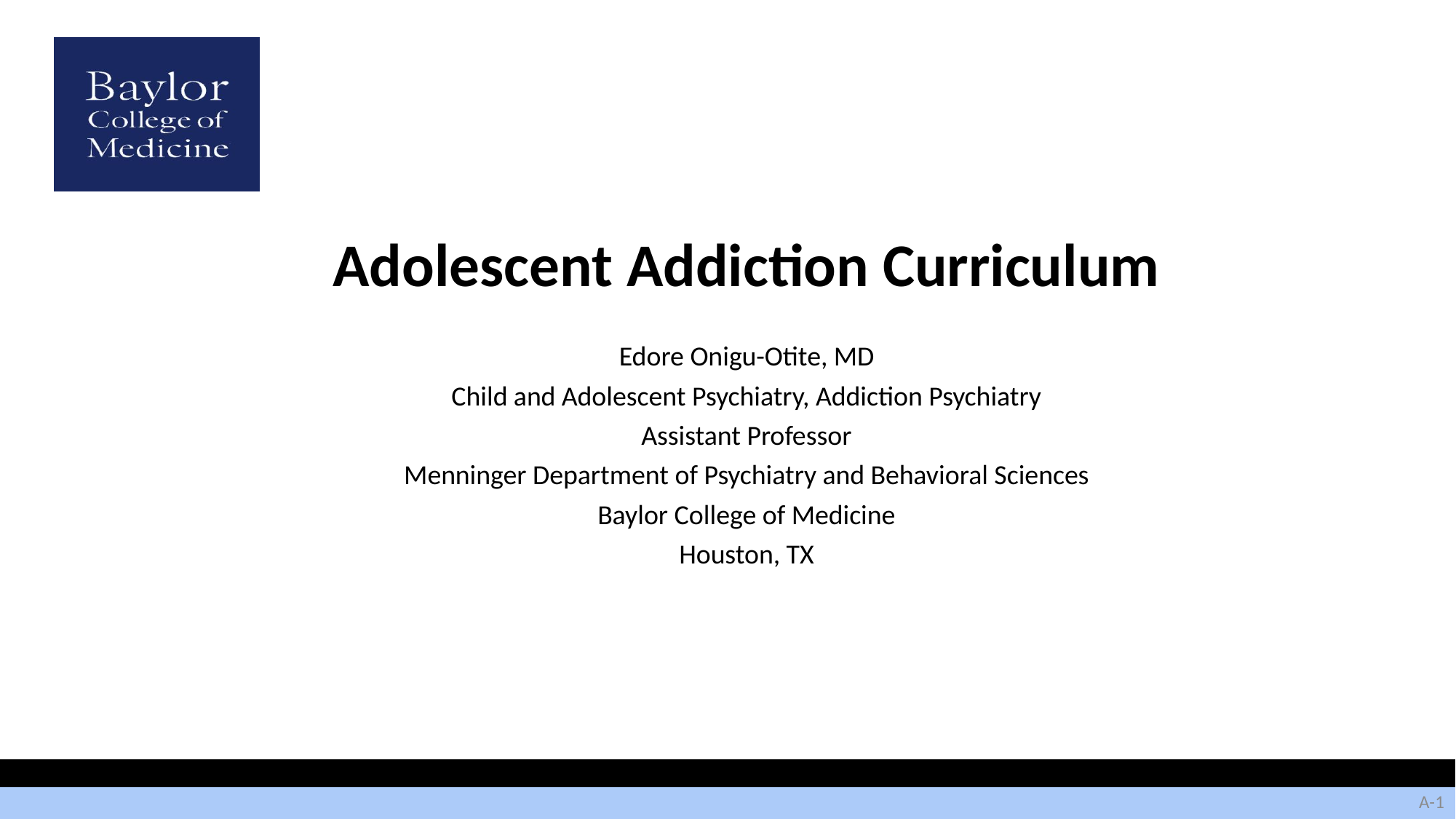

Adolescent Addiction Curriculum
Edore Onigu-Otite, MD
Child and Adolescent Psychiatry, Addiction Psychiatry
Assistant Professor
Menninger Department of Psychiatry and Behavioral Sciences
Baylor College of Medicine
Houston, TX
A-1

## Slide 2
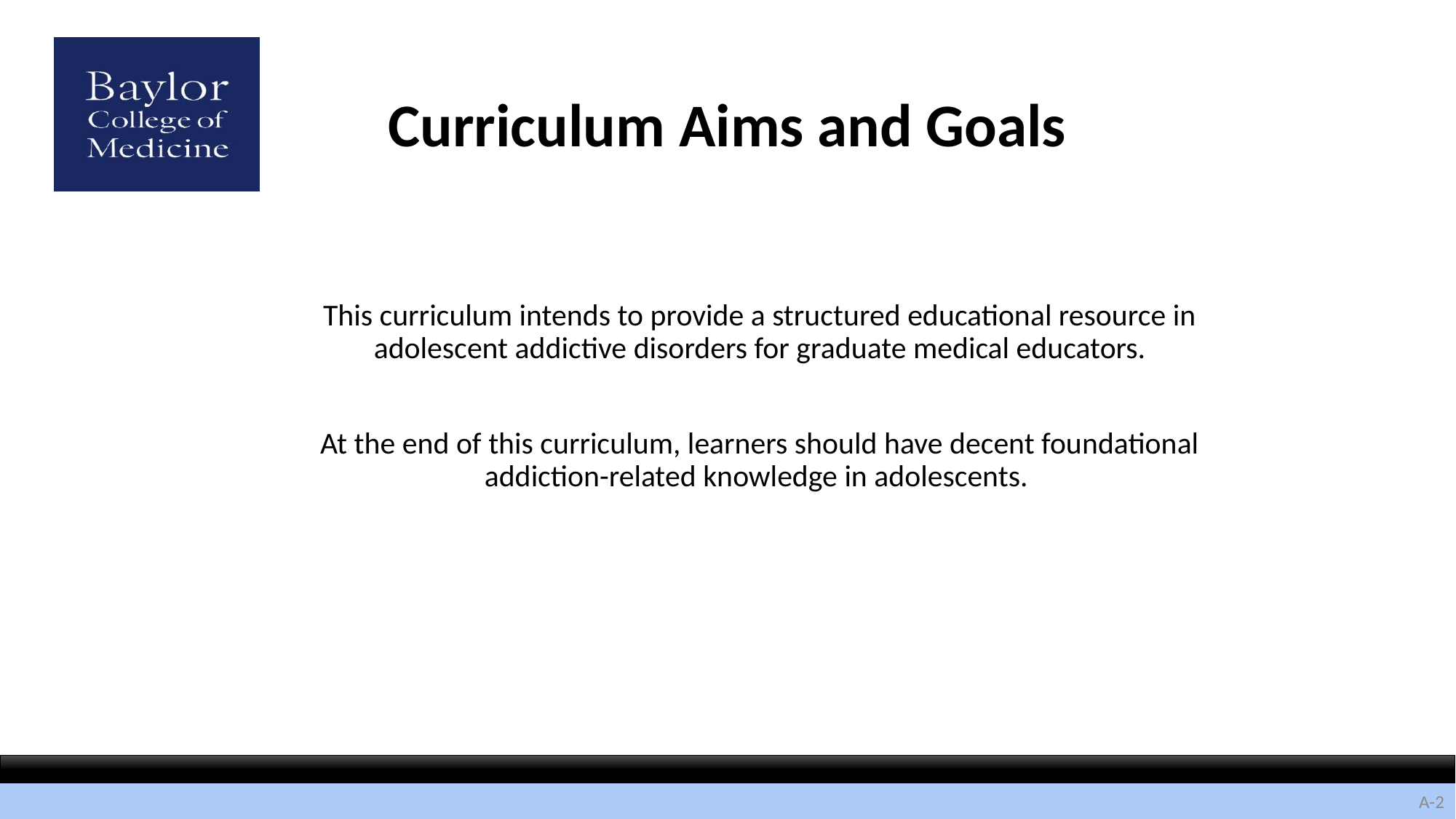

Curriculum Aims and Goals
This curriculum intends to provide a structured educational resource in adolescent addictive disorders for graduate medical educators.
At the end of this curriculum, learners should have decent foundational addiction-related knowledge in adolescents.
A-2

## Slide 3
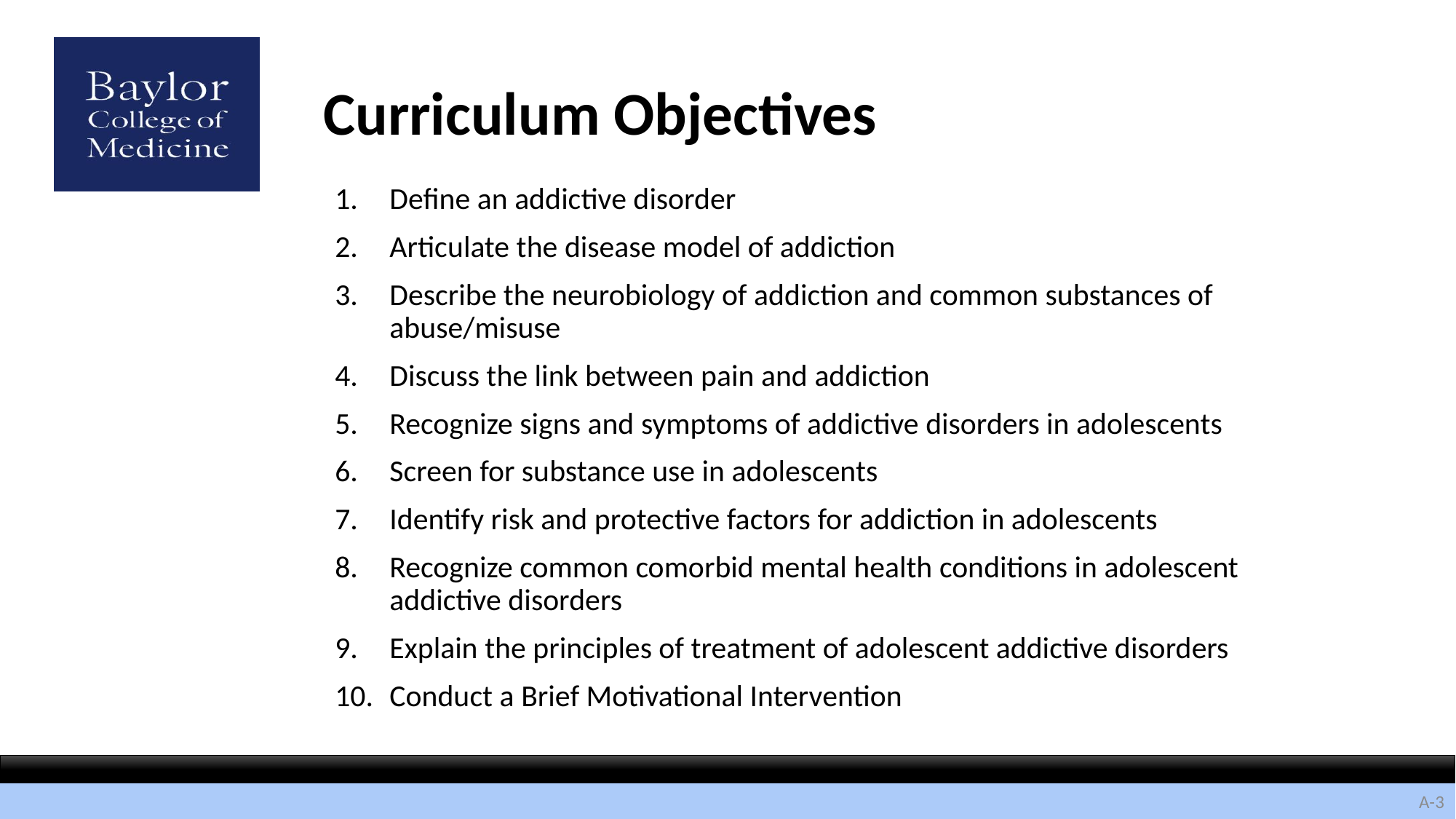

Curriculum Objectives
Define an addictive disorder
Articulate the disease model of addiction
Describe the neurobiology of addiction and common substances of abuse/misuse
Discuss the link between pain and addiction
Recognize signs and symptoms of addictive disorders in adolescents
Screen for substance use in adolescents
Identify risk and protective factors for addiction in adolescents
Recognize common comorbid mental health conditions in adolescent addictive disorders
Explain the principles of treatment of adolescent addictive disorders
Conduct a Brief Motivational Intervention
A-3

## Slide 4
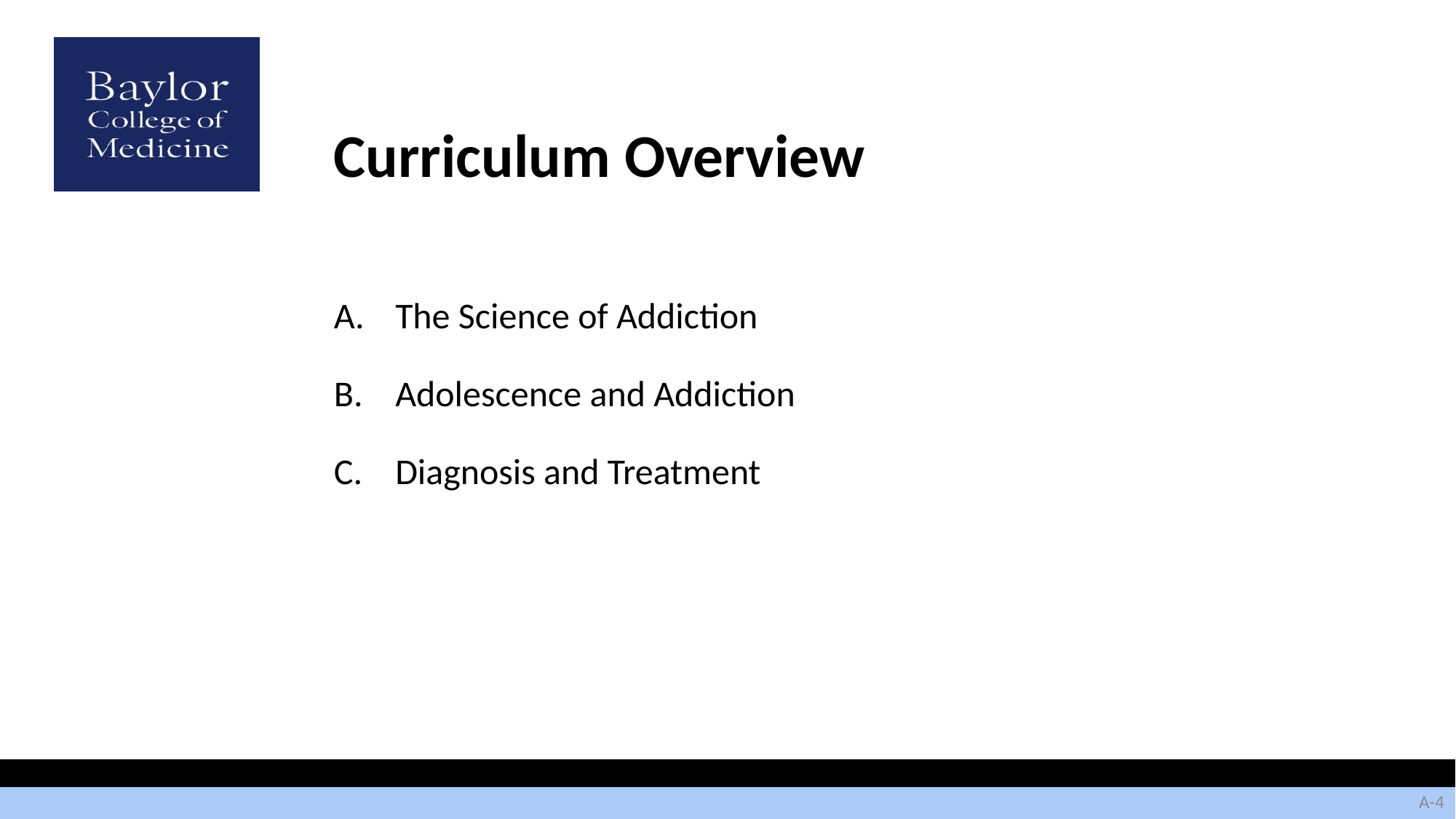

Curriculum Overview
The Science of Addiction
Adolescence and Addiction
Diagnosis and Treatment
A-4

## Slide 5
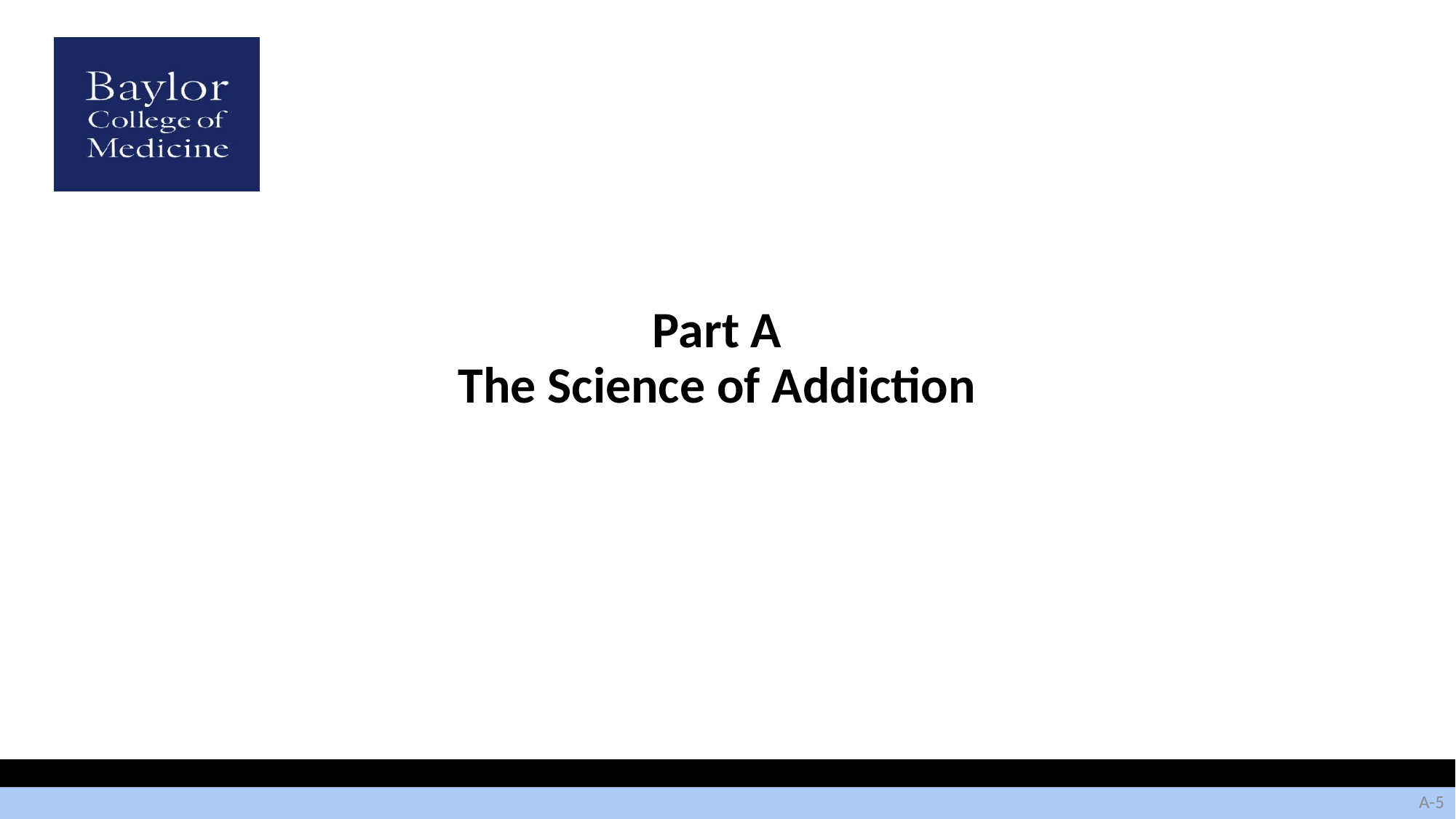

Part A
The Science of Addiction
A-5

## Slide 6
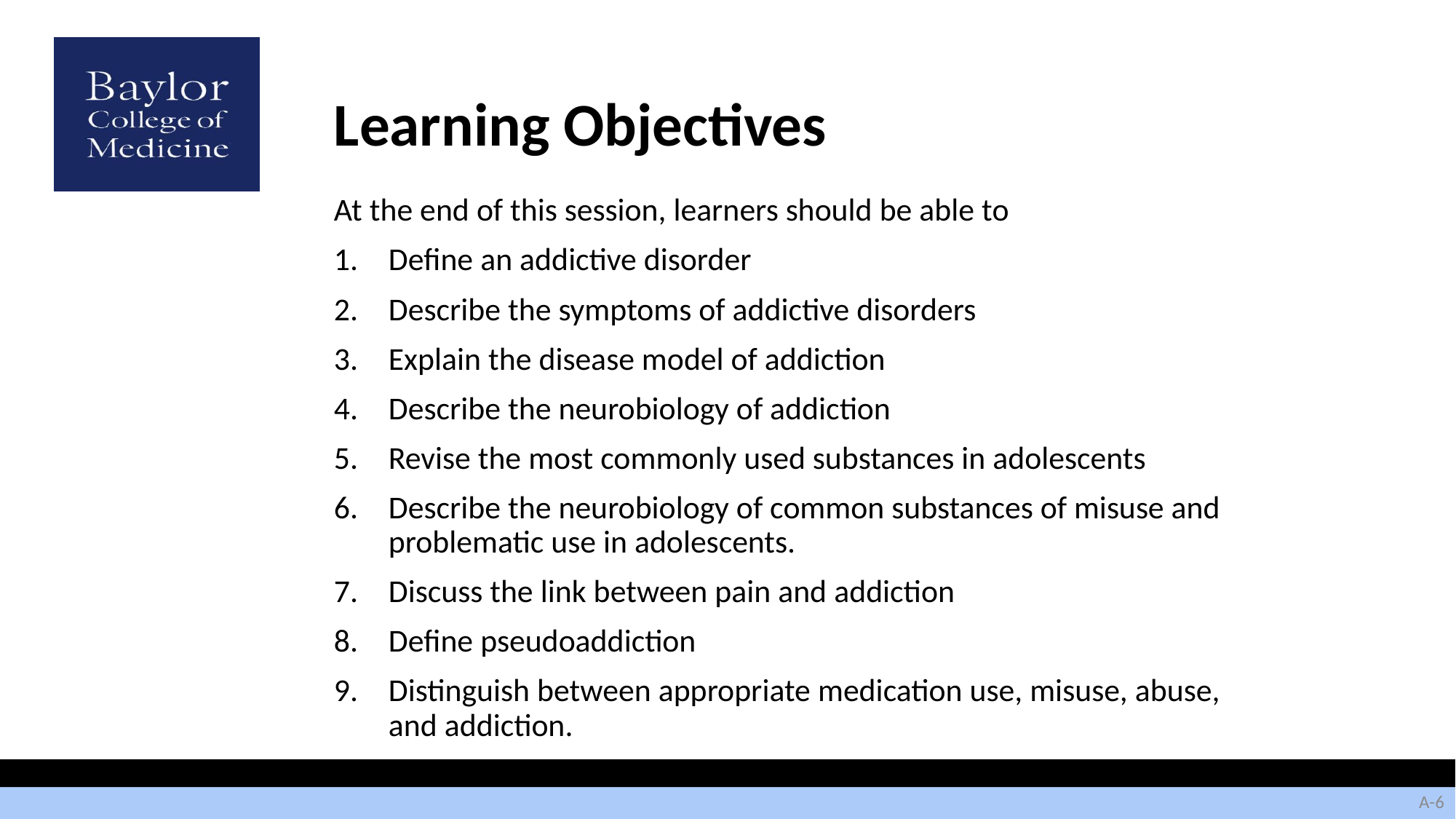

Learning Objectives
At the end of this session, learners should be able to
Define an addictive disorder
Describe the symptoms of addictive disorders
Explain the disease model of addiction
Describe the neurobiology of addiction
Revise the most commonly used substances in adolescents
Describe the neurobiology of common substances of misuse and problematic use in adolescents.
Discuss the link between pain and addiction
Define pseudoaddiction
Distinguish between appropriate medication use, misuse, abuse, and addiction.
A-6

## Slide 7
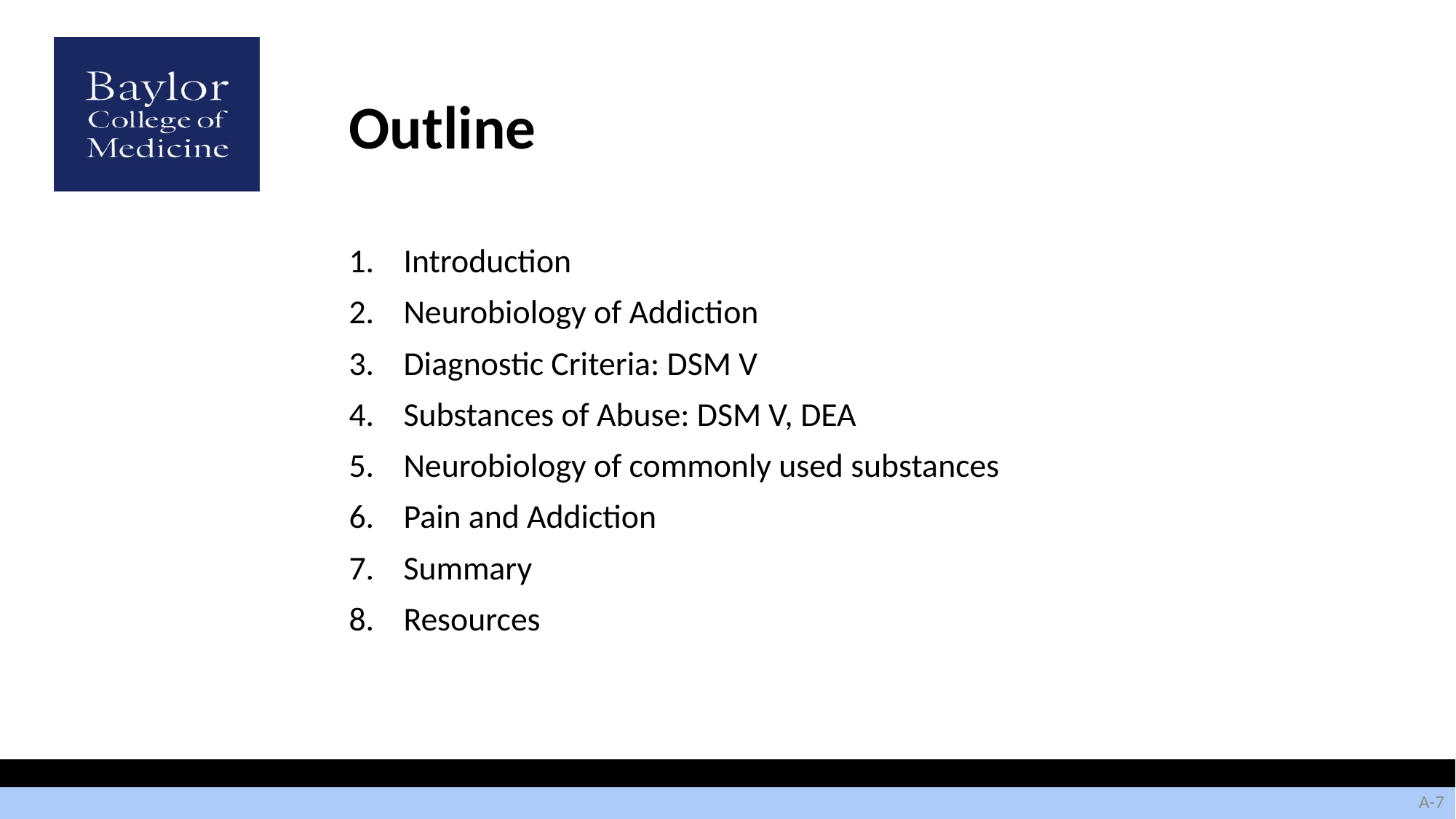

Outline
Introduction
Neurobiology of Addiction
Diagnostic Criteria: DSM V
Substances of Abuse: DSM V, DEA
Neurobiology of commonly used substances
Pain and Addiction
Summary
Resources
A-7

## Slide 8
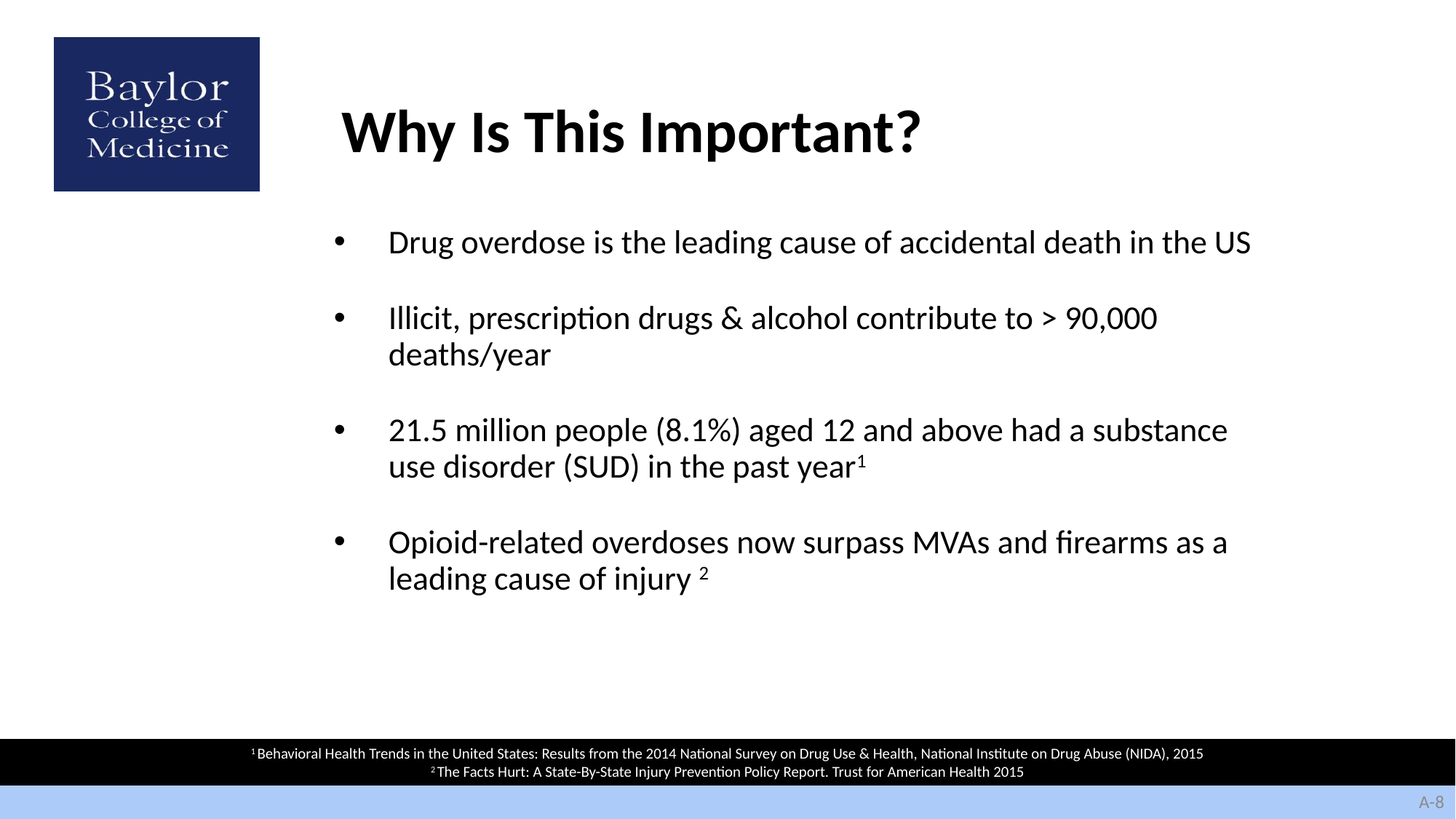

Why Is This Important?
Drug overdose is the leading cause of accidental death in the US
Illicit, prescription drugs & alcohol contribute to > 90,000 deaths/year
21.5 million people (8.1%) aged 12 and above had a substance use disorder (SUD) in the past year1
Opioid-related overdoses now surpass MVAs and firearms as a leading cause of injury 2
1 Behavioral Health Trends in the United States: Results from the 2014 National Survey on Drug Use & Health, National Institute on Drug Abuse (NIDA), 2015
2 The Facts Hurt: A State-By-State Injury Prevention Policy Report. Trust for American Health 2015
A-8

## Slide 9
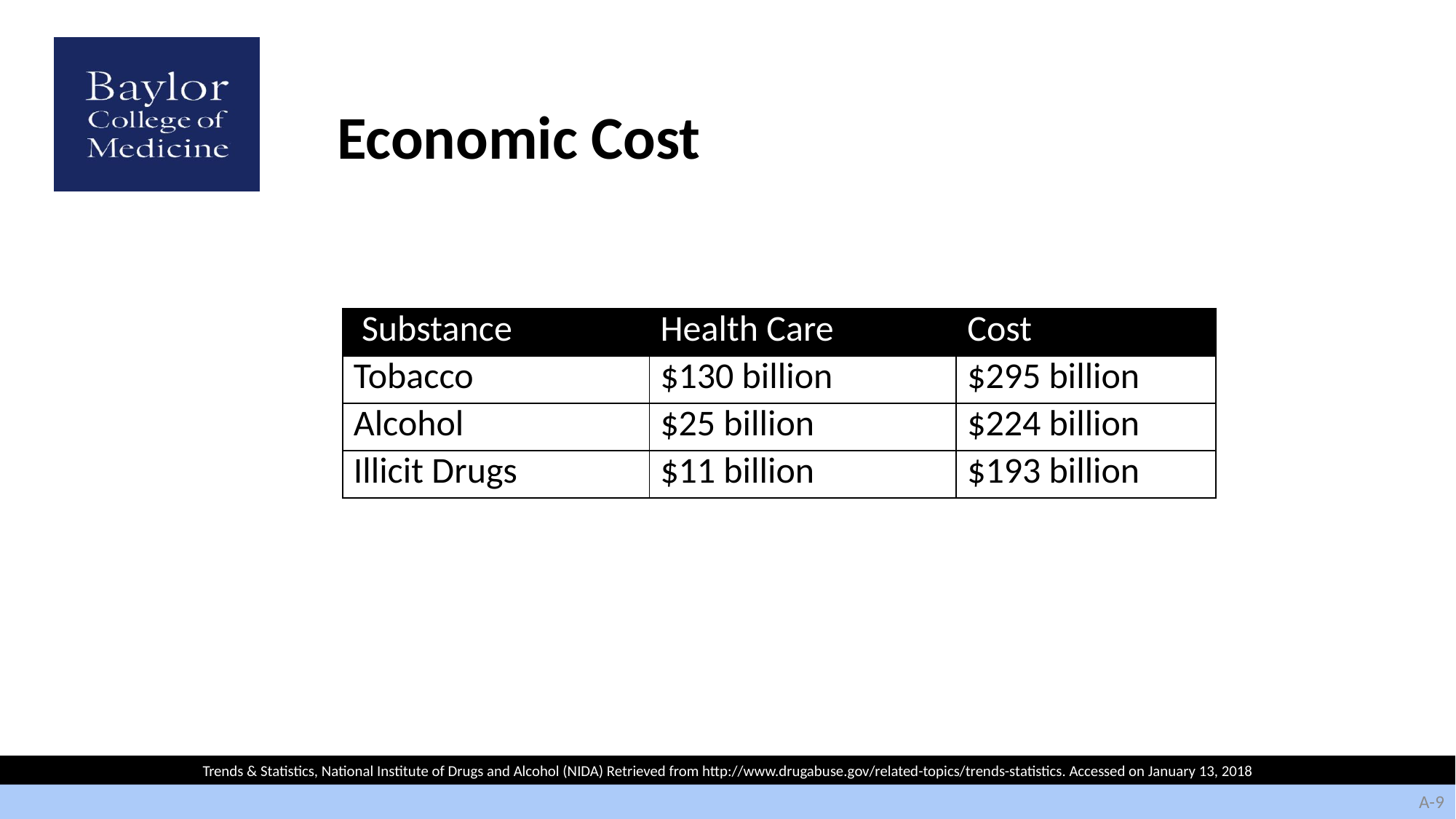

Economic Cost
| Substance | Health Care | Cost |
| --- | --- | --- |
| Tobacco | $130 billion | $295 billion |
| Alcohol | $25 billion | $224 billion |
| Illicit Drugs | $11 billion | $193 billion |
Trends & Statistics, National Institute of Drugs and Alcohol (NIDA) Retrieved from http://www.drugabuse.gov/related-topics/trends-statistics. Accessed on January 13, 2018
A-9

## Slide 10
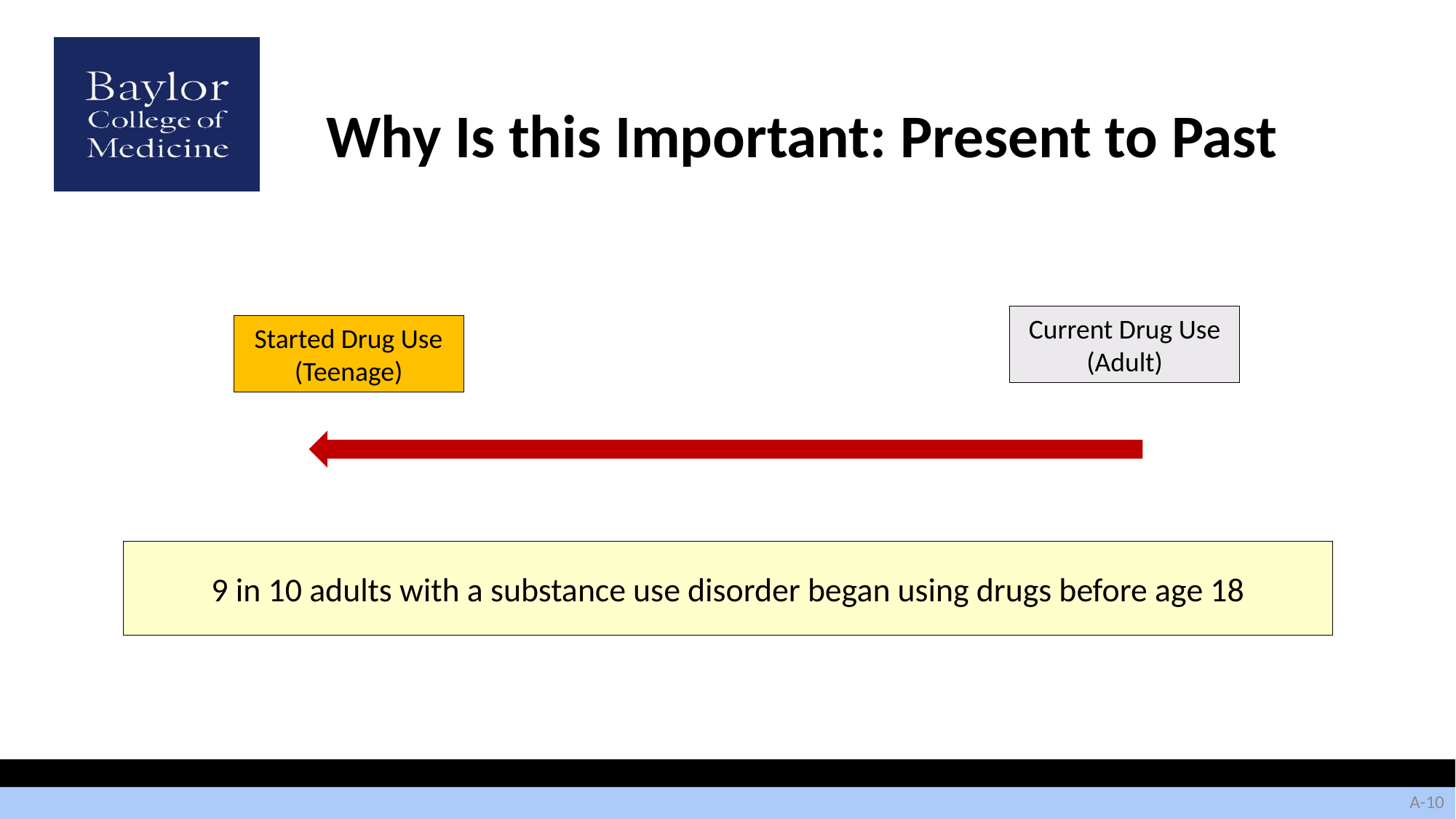

Why Is this Important: Present to Past
Current Drug Use
(Adult)
Started Drug Use
(Teenage)
9 in 10 adults with a substance use disorder began using drugs before age 18
A-10

## Slide 11
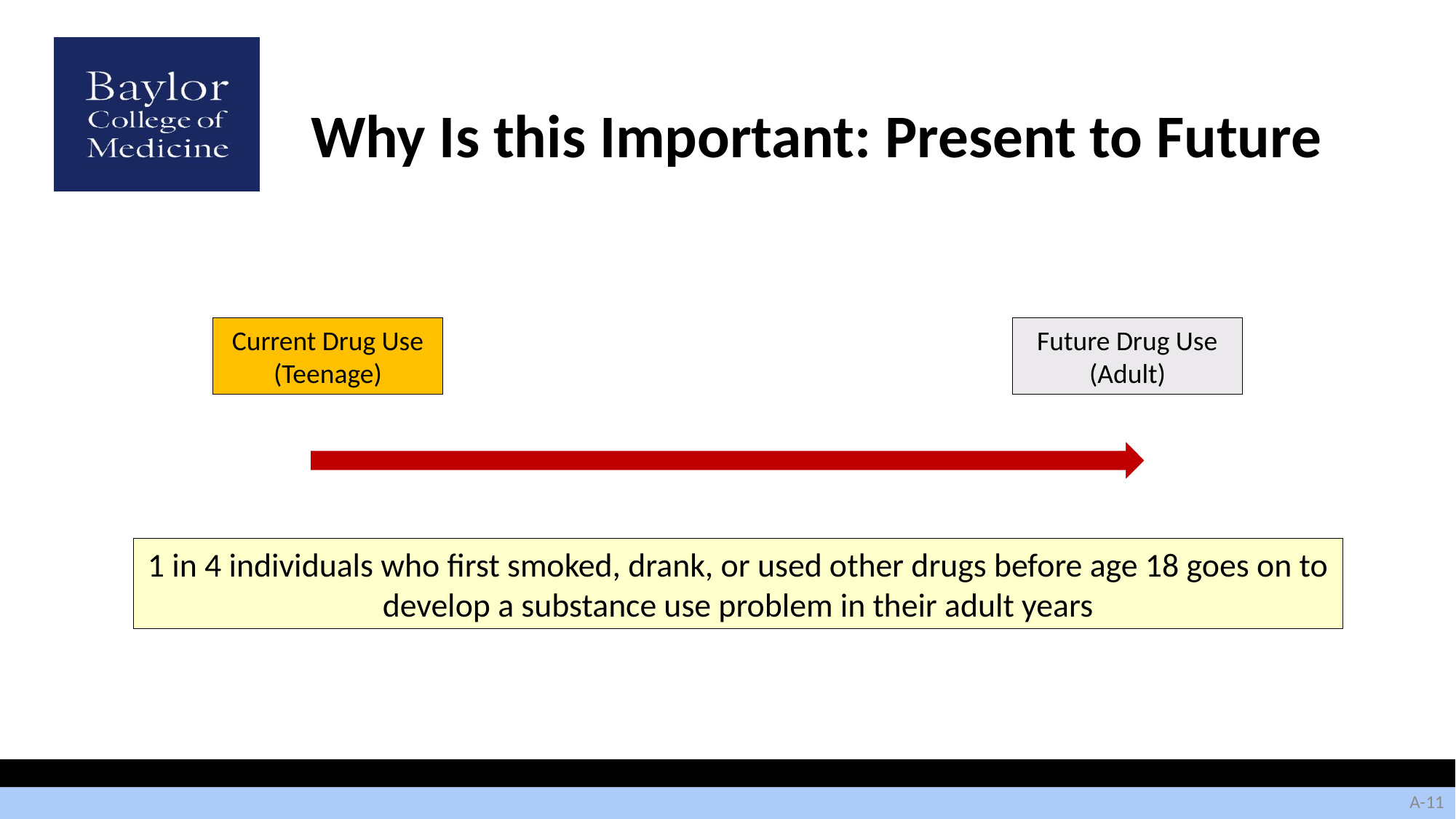

Why Is this Important: Present to Future
Future Drug Use
(Adult)
Current Drug Use
(Teenage)
1 in 4 individuals who first smoked, drank, or used other drugs before age 18 goes on to develop a substance use problem in their adult years
A-11

## Slide 12
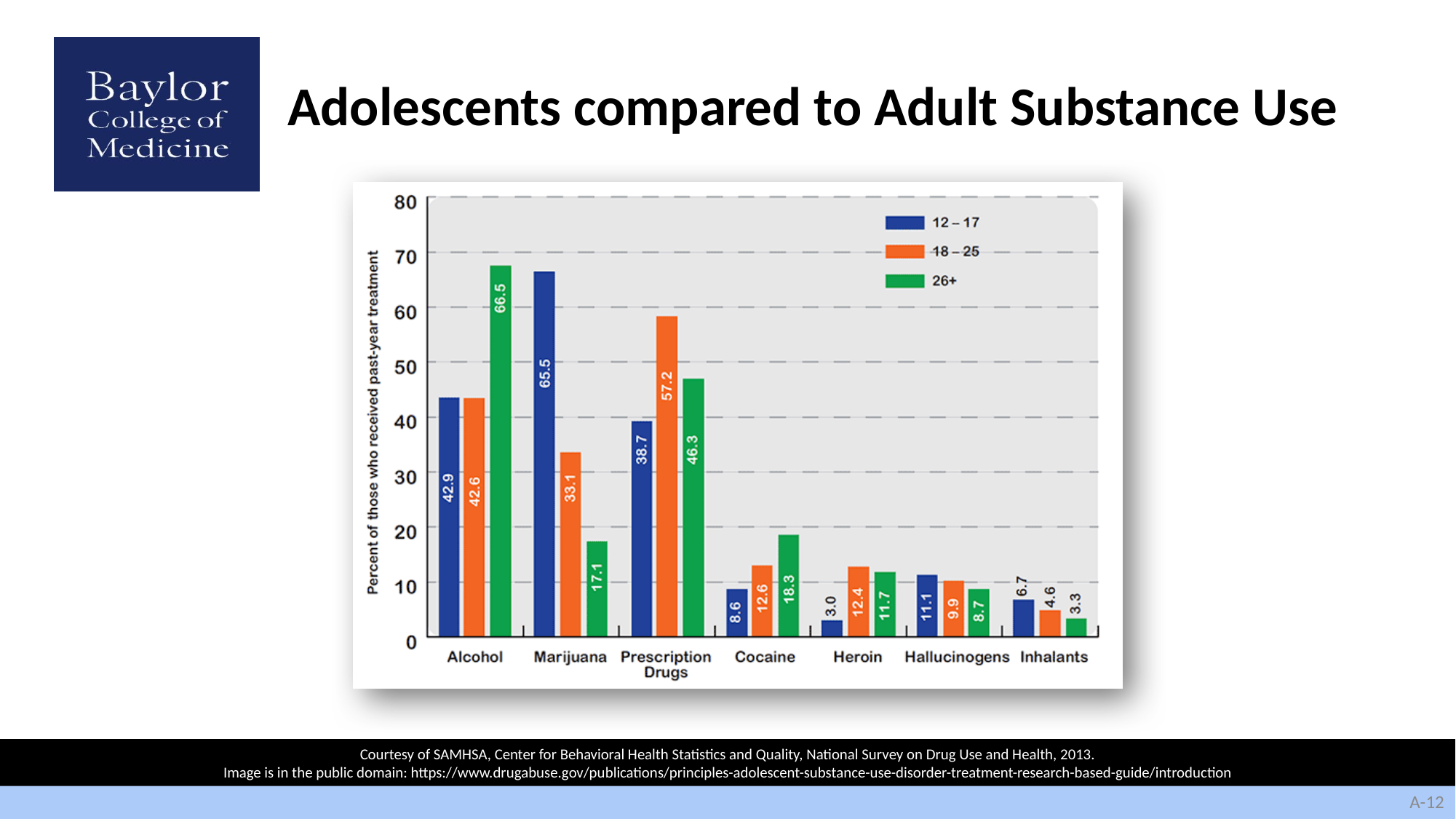

Adolescents compared to Adult Substance Use
Courtesy of SAMHSA, Center for Behavioral Health Statistics and Quality, National Survey on Drug Use and Health, 2013.
Image is in the public domain: https://www.drugabuse.gov/publications/principles-adolescent-substance-use-disorder-treatment-research-based-guide/introduction
A-12

## Slide 13
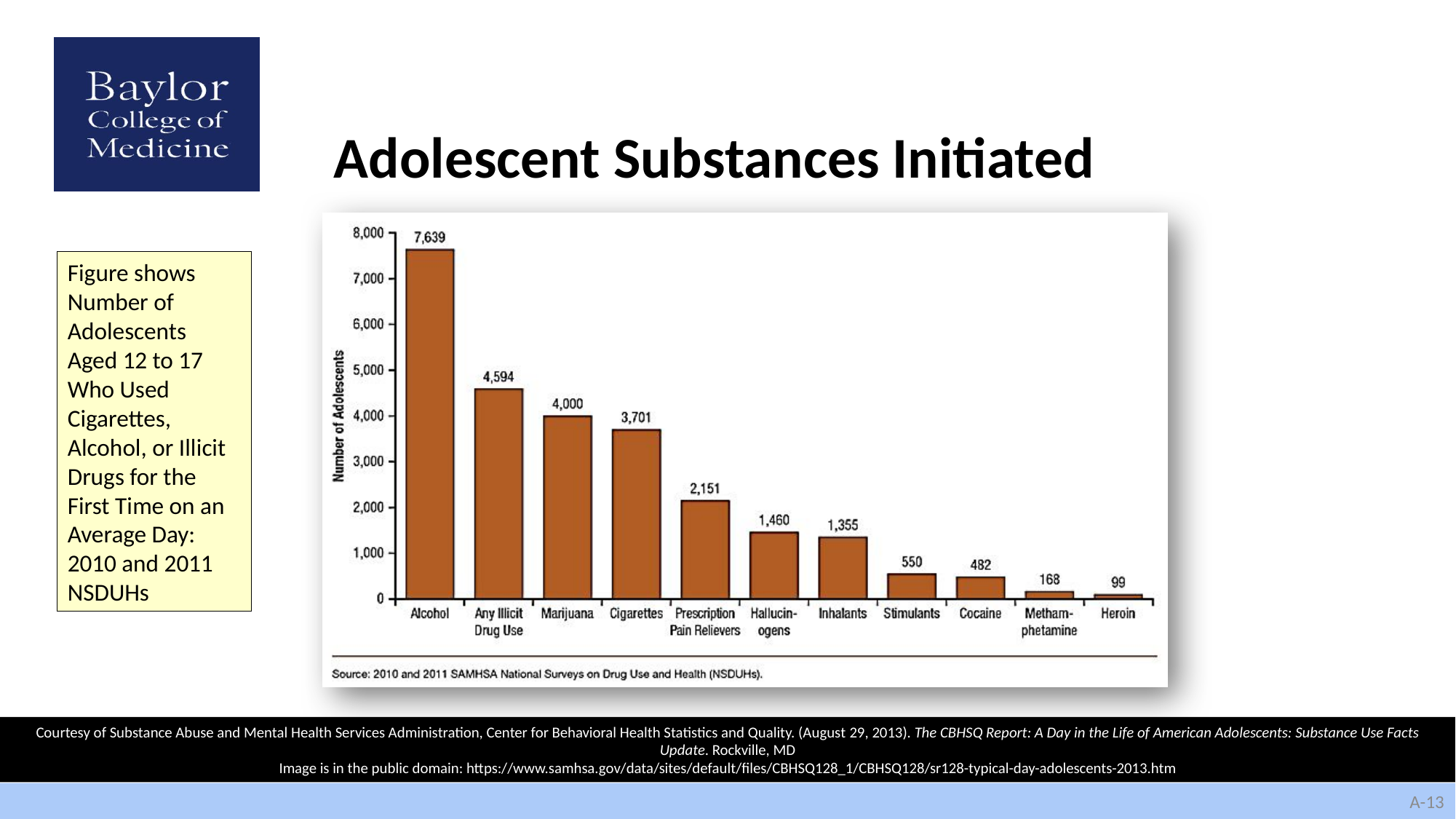

Adolescent Substances Initiated
Figure shows Number of Adolescents Aged 12 to 17 Who Used Cigarettes, Alcohol, or Illicit Drugs for the First Time on an Average Day: 2010 and 2011 NSDUHs
Courtesy of Substance Abuse and Mental Health Services Administration, Center for Behavioral Health Statistics and Quality. (August 29, 2013). The CBHSQ Report: A Day in the Life of American Adolescents: Substance Use Facts Update. Rockville, MD
Image is in the public domain: https://www.samhsa.gov/data/sites/default/files/CBHSQ128_1/CBHSQ128/sr128-typical-day-adolescents-2013.htm
A-13

## Slide 14
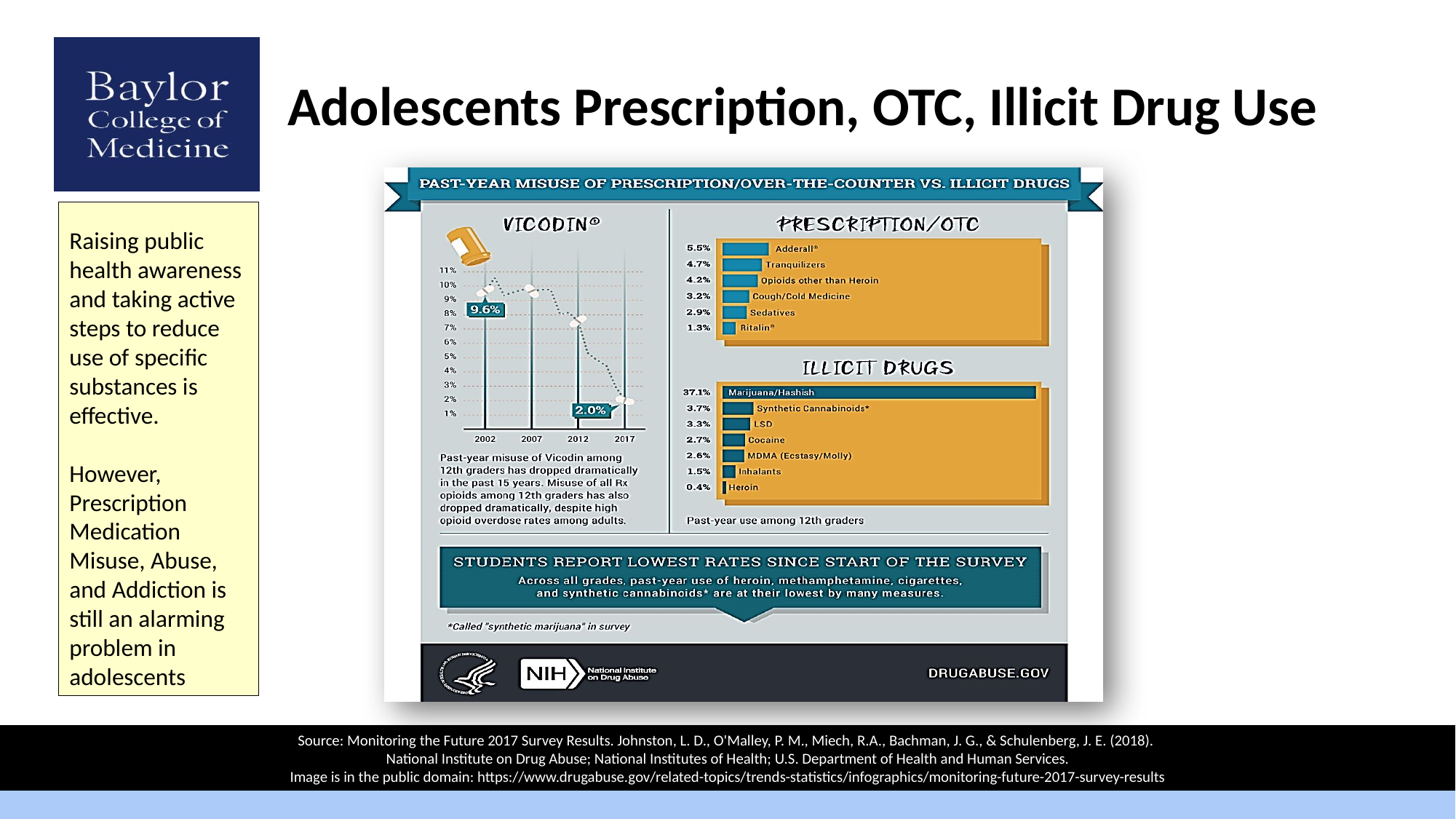

Adolescents Prescription, OTC, Illicit Drug Use
Raising public health awareness and taking active steps to reduce use of specific substances is effective.
However, Prescription Medication Misuse, Abuse, and Addiction is still an alarming problem in adolescents
Source: Monitoring the Future 2017 Survey Results. Johnston, L. D., O'Malley, P. M., Miech, R.A., Bachman, J. G., & Schulenberg, J. E. (2018).
National Institute on Drug Abuse; National Institutes of Health; U.S. Department of Health and Human Services.
Image is in the public domain: https://www.drugabuse.gov/related-topics/trends-statistics/infographics/monitoring-future-2017-survey-results

## Slide 15
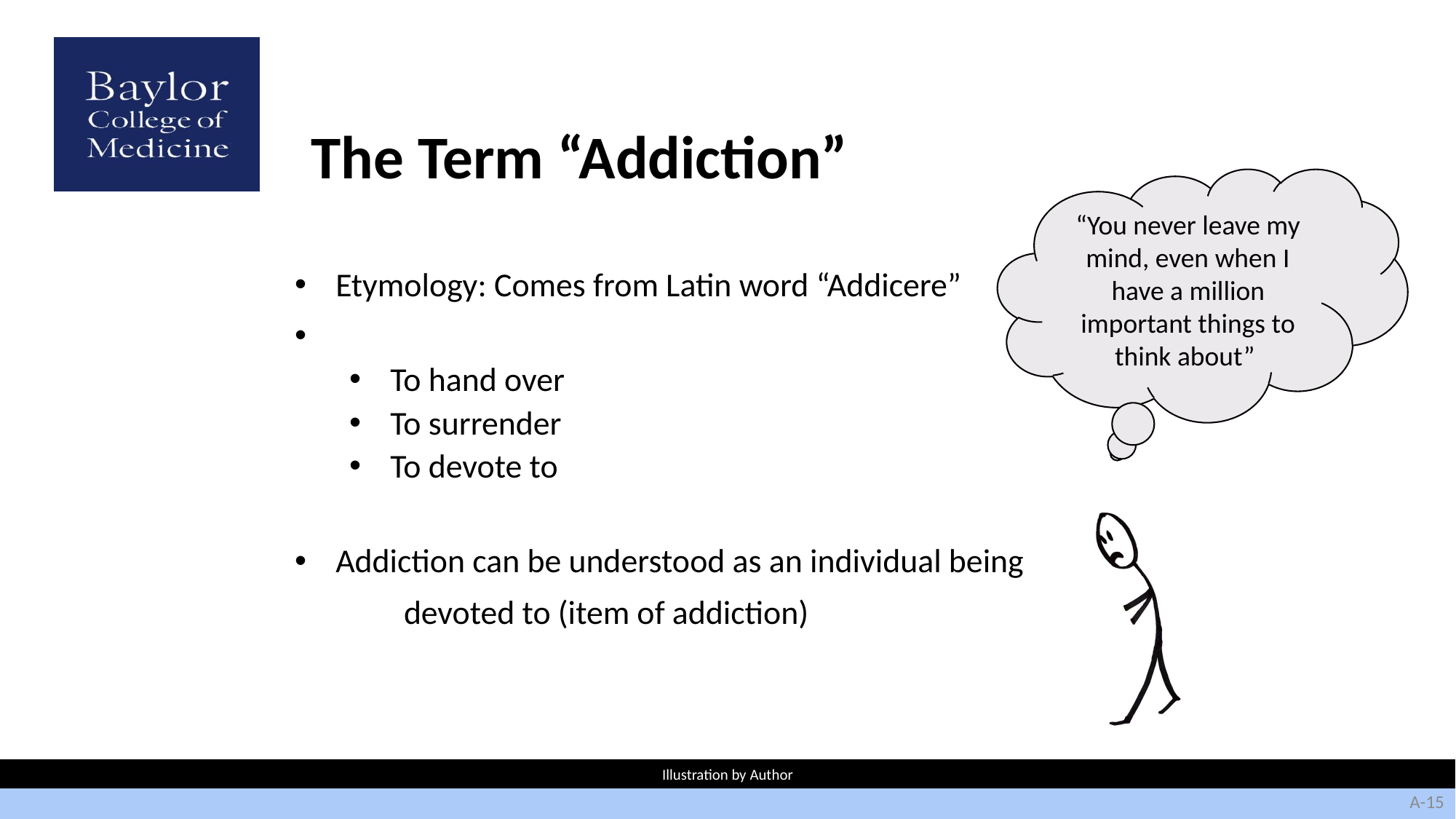

The Term “Addiction”
“You never leave my mind, even when I have a million important things to think about”
Etymology: Comes from Latin word “Addicere”
To hand over
To surrender
To devote to
Addiction can be understood as an individual being
	devoted to (item of addiction)
Illustration by Author
A-15

## Slide 16
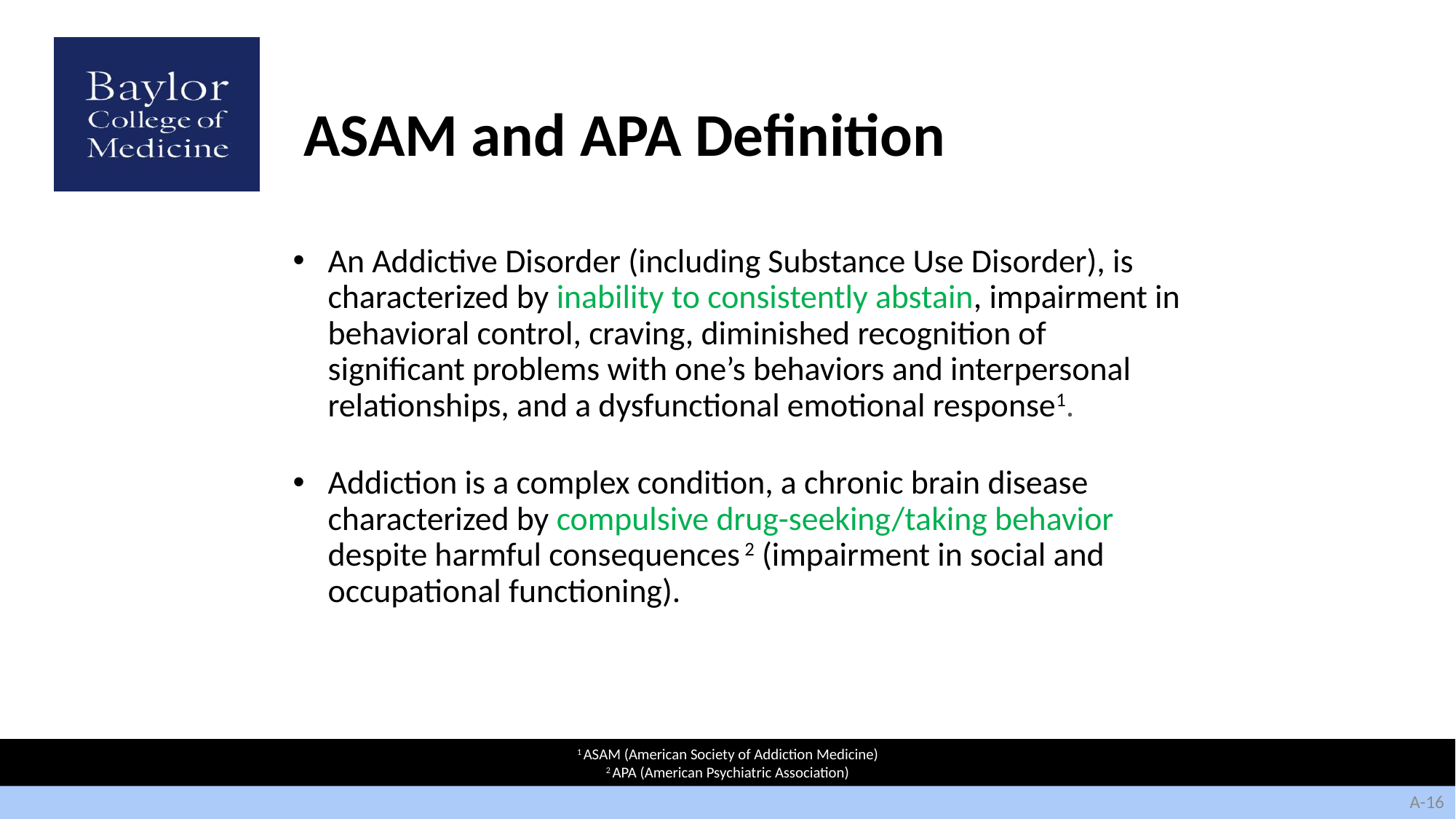

ASAM and APA Definition
An Addictive Disorder (including Substance Use Disorder), is characterized by inability to consistently abstain, impairment in behavioral control, craving, diminished recognition of significant problems with one’s behaviors and interpersonal relationships, and a dysfunctional emotional response1.
Addiction is a complex condition, a chronic brain disease characterized by compulsive drug-seeking/taking behavior despite harmful consequences 2 (impairment in social and occupational functioning).
1 ASAM (American Society of Addiction Medicine)
2 APA (American Psychiatric Association)
A-16

## Slide 17
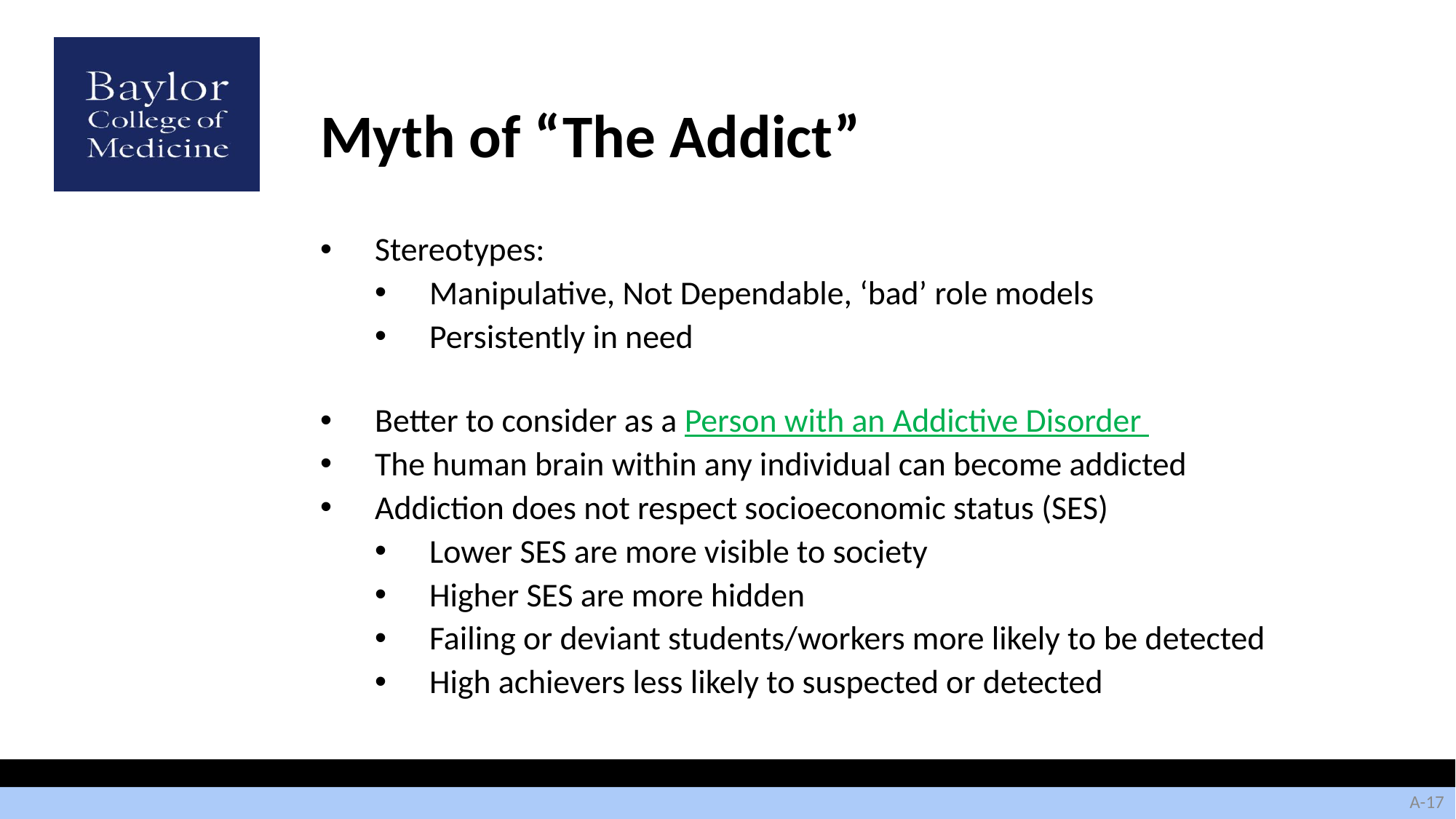

Myth of “The Addict”
Stereotypes:
Manipulative, Not Dependable, ‘bad’ role models
Persistently in need
Better to consider as a Person with an Addictive Disorder
The human brain within any individual can become addicted
Addiction does not respect socioeconomic status (SES)
Lower SES are more visible to society
Higher SES are more hidden
Failing or deviant students/workers more likely to be detected
High achievers less likely to suspected or detected
A-17

## Slide 18
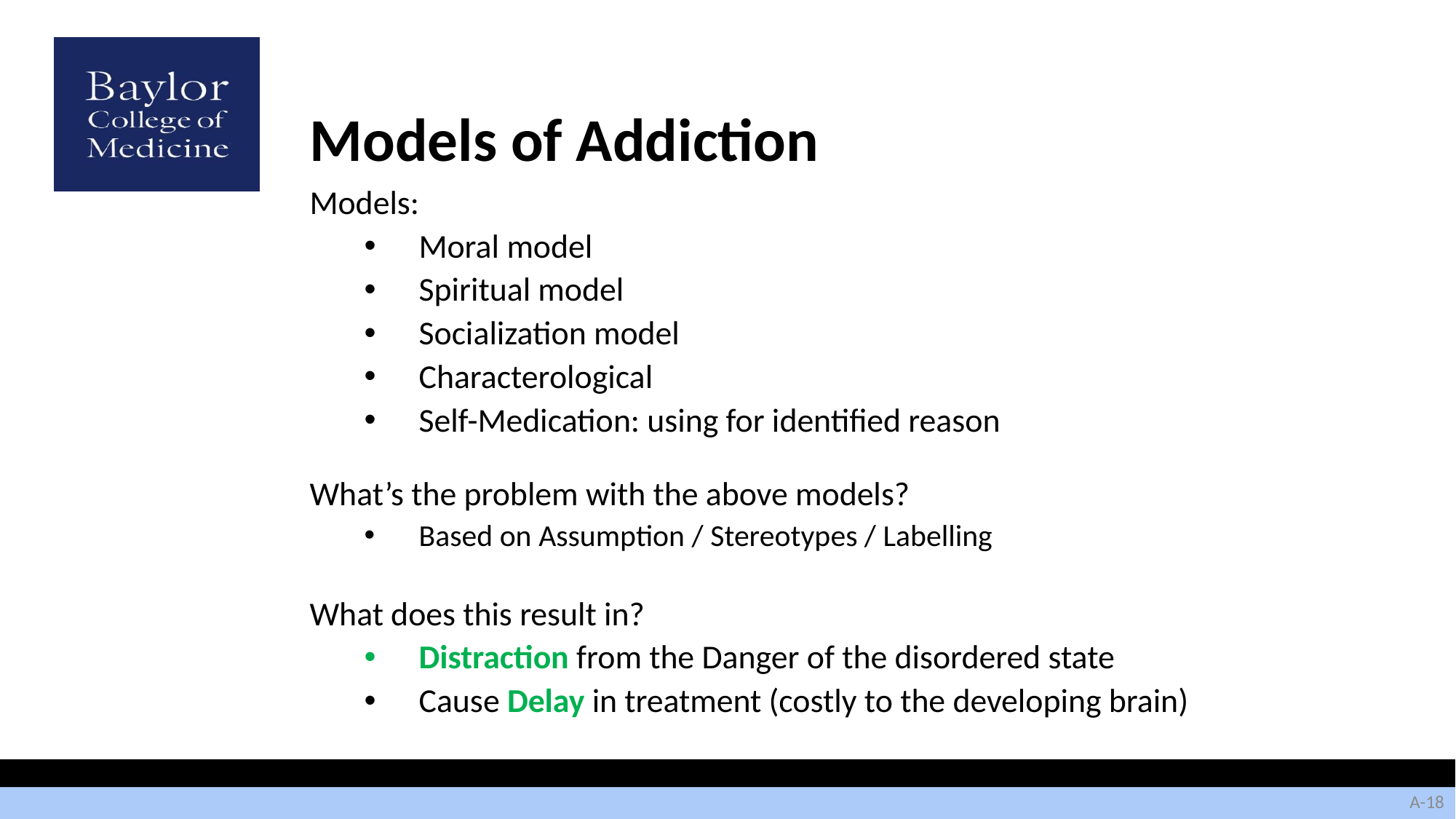

Models of Addiction
Models:
Moral model
Spiritual model
Socialization model
Characterological
Self-Medication: using for identified reason
What’s the problem with the above models?
Based on Assumption / Stereotypes / Labelling
What does this result in?
Distraction from the Danger of the disordered state
Cause Delay in treatment (costly to the developing brain)
A-18

## Slide 19
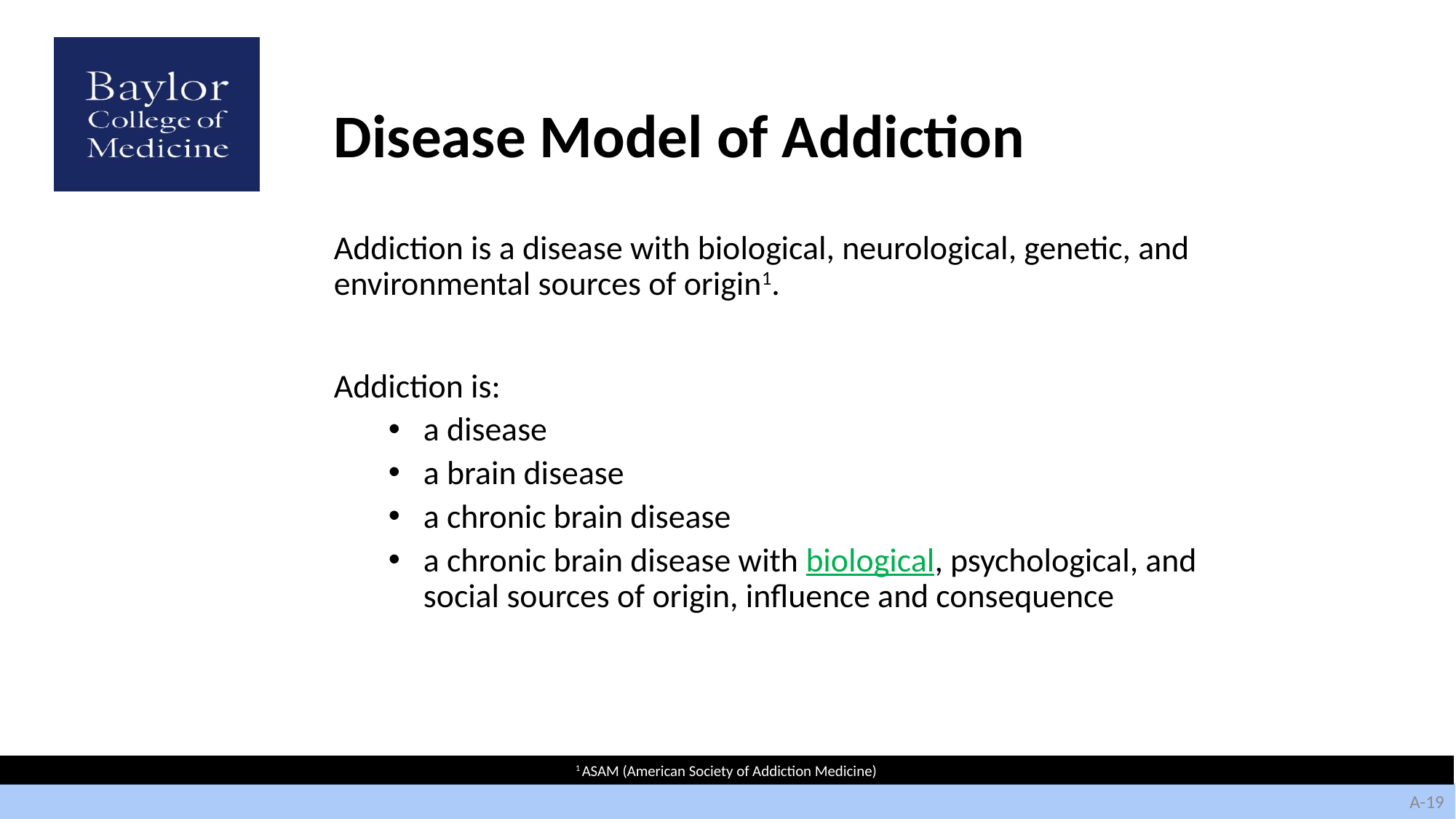

Disease Model of Addiction
Addiction is a disease with biological, neurological, genetic, and environmental sources of origin1.
Addiction is:
a disease
a brain disease
a chronic brain disease
a chronic brain disease with biological, psychological, and social sources of origin, influence and consequence
1 ASAM (American Society of Addiction Medicine)
A-19

## Slide 20
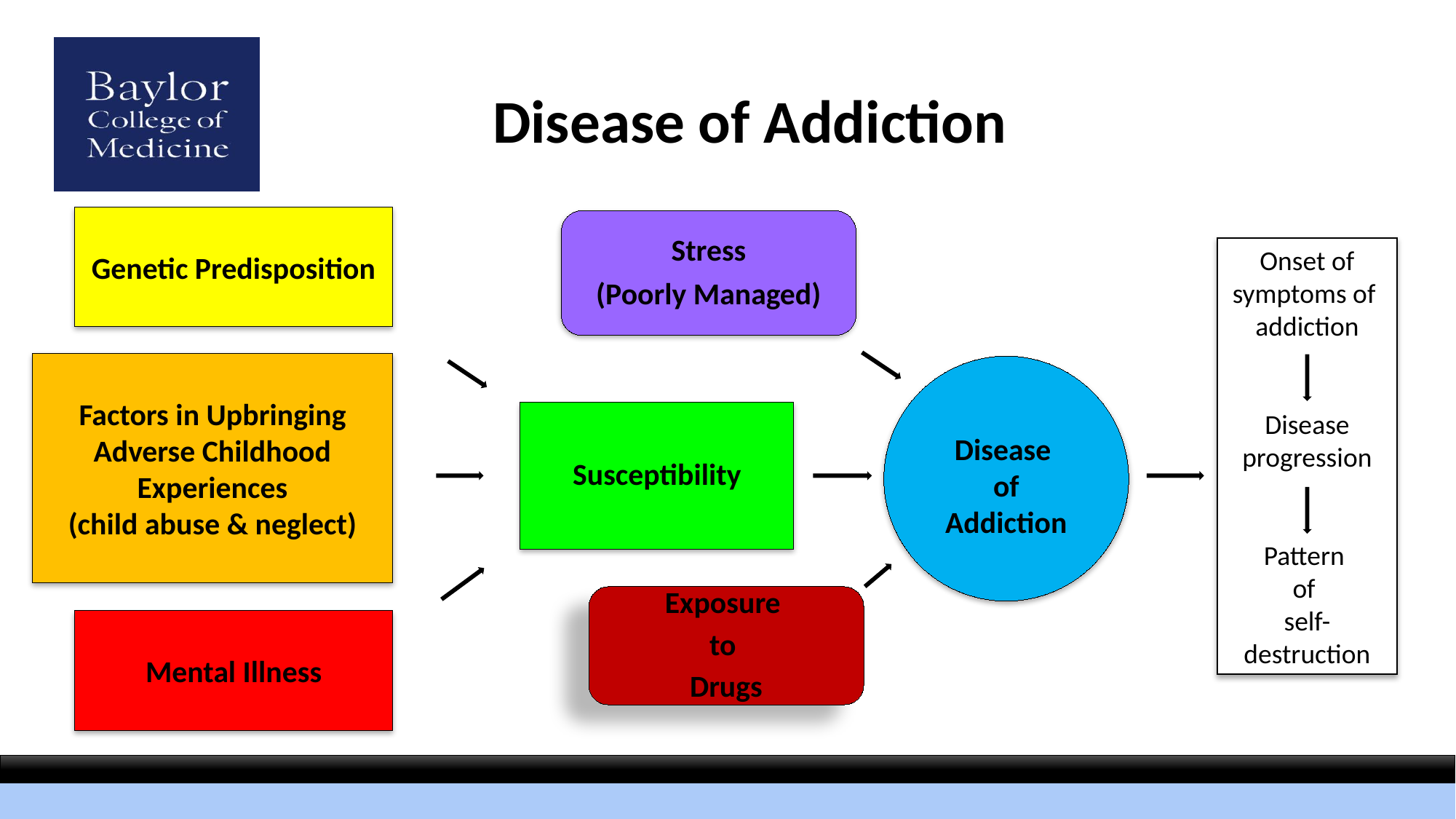

Disease of Addiction
Genetic Predisposition
Stress
(Poorly Managed)
Onset of symptoms of addiction
Disease progression
Pattern
of
self-
destruction
Factors in Upbringing
Adverse Childhood Experiences
(child abuse & neglect)
Disease
of Addiction
Susceptibility
Exposure
to
Drugs
Mental Illness

## Slide 21
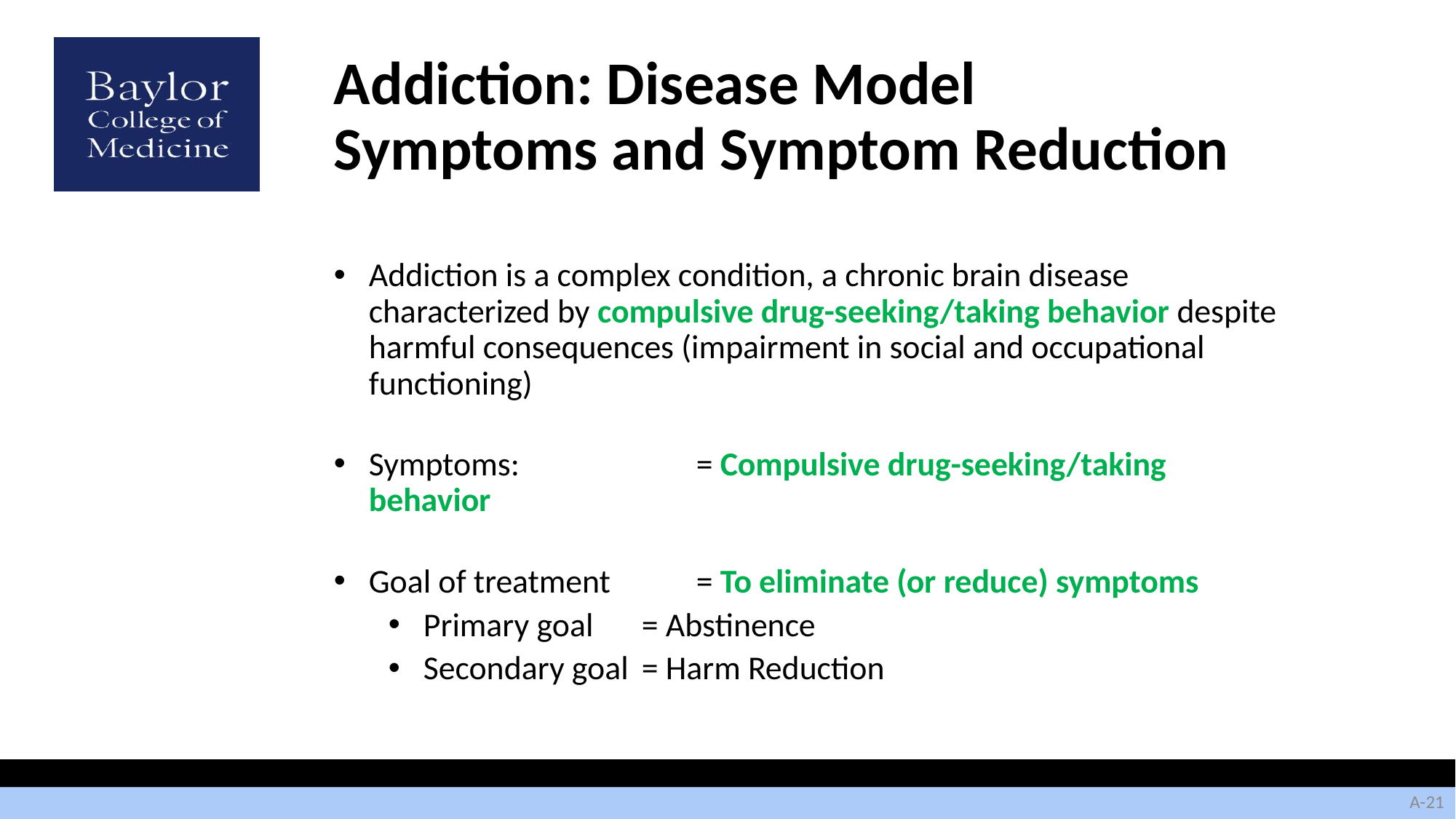

Addiction: Disease Model
Symptoms and Symptom Reduction
Addiction is a complex condition, a chronic brain disease characterized by compulsive drug-seeking/taking behavior despite harmful consequences (impairment in social and occupational functioning)
Symptoms:		= Compulsive drug-seeking/taking behavior
Goal of treatment 	= To eliminate (or reduce) symptoms
Primary goal 	= Abstinence
Secondary goal 	= Harm Reduction
A-21

## Slide 22
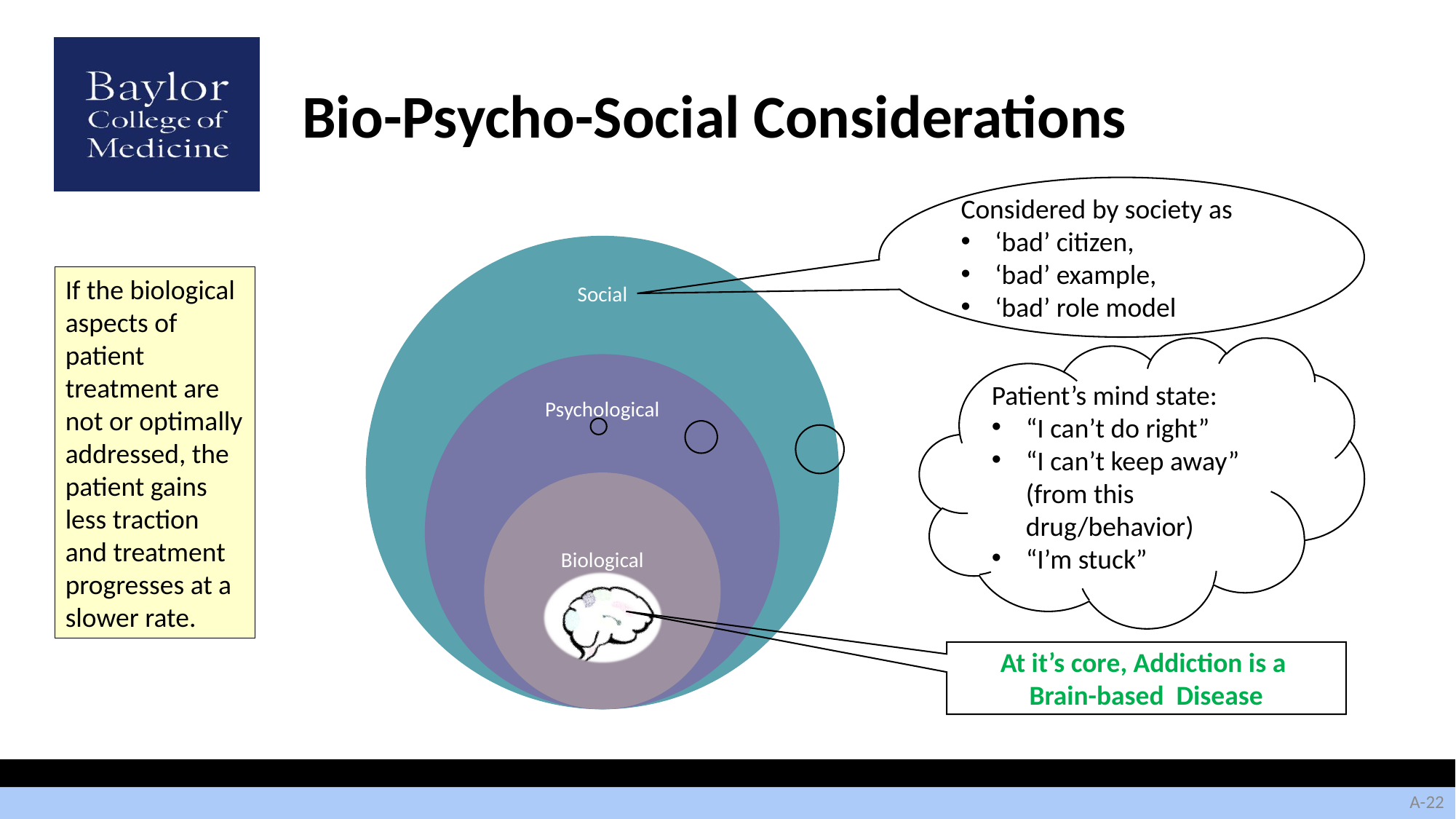

Bio-Psycho-Social Considerations
Considered by society as
‘bad’ citizen,
‘bad’ example,
‘bad’ role model
If the biological aspects of patient treatment are not or optimally addressed, the patient gains less traction and treatment progresses at a slower rate.
Patient’s mind state:
“I can’t do right”
“I can’t keep away” (from this drug/behavior)
“I’m stuck”
At it’s core, Addiction is a
Brain-based Disease
A-22

## Slide 23
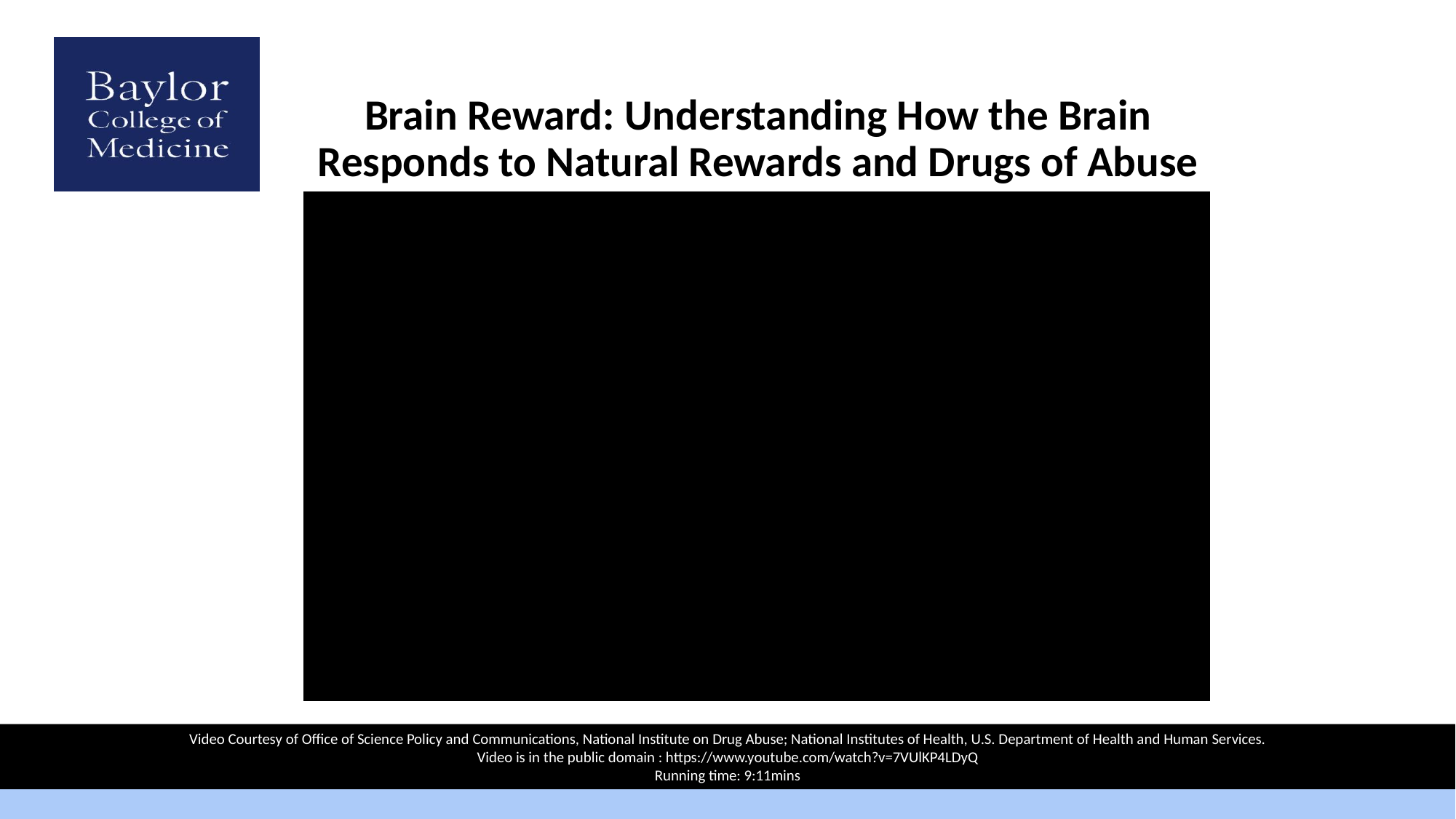

Brain Reward: Understanding How the Brain Responds to Natural Rewards and Drugs of Abuse
Video Courtesy of Office of Science Policy and Communications, National Institute on Drug Abuse; National Institutes of Health, U.S. Department of Health and Human Services.
Video is in the public domain : https://www.youtube.com/watch?v=7VUlKP4LDyQ
Running time: 9:11mins

## Slide 24
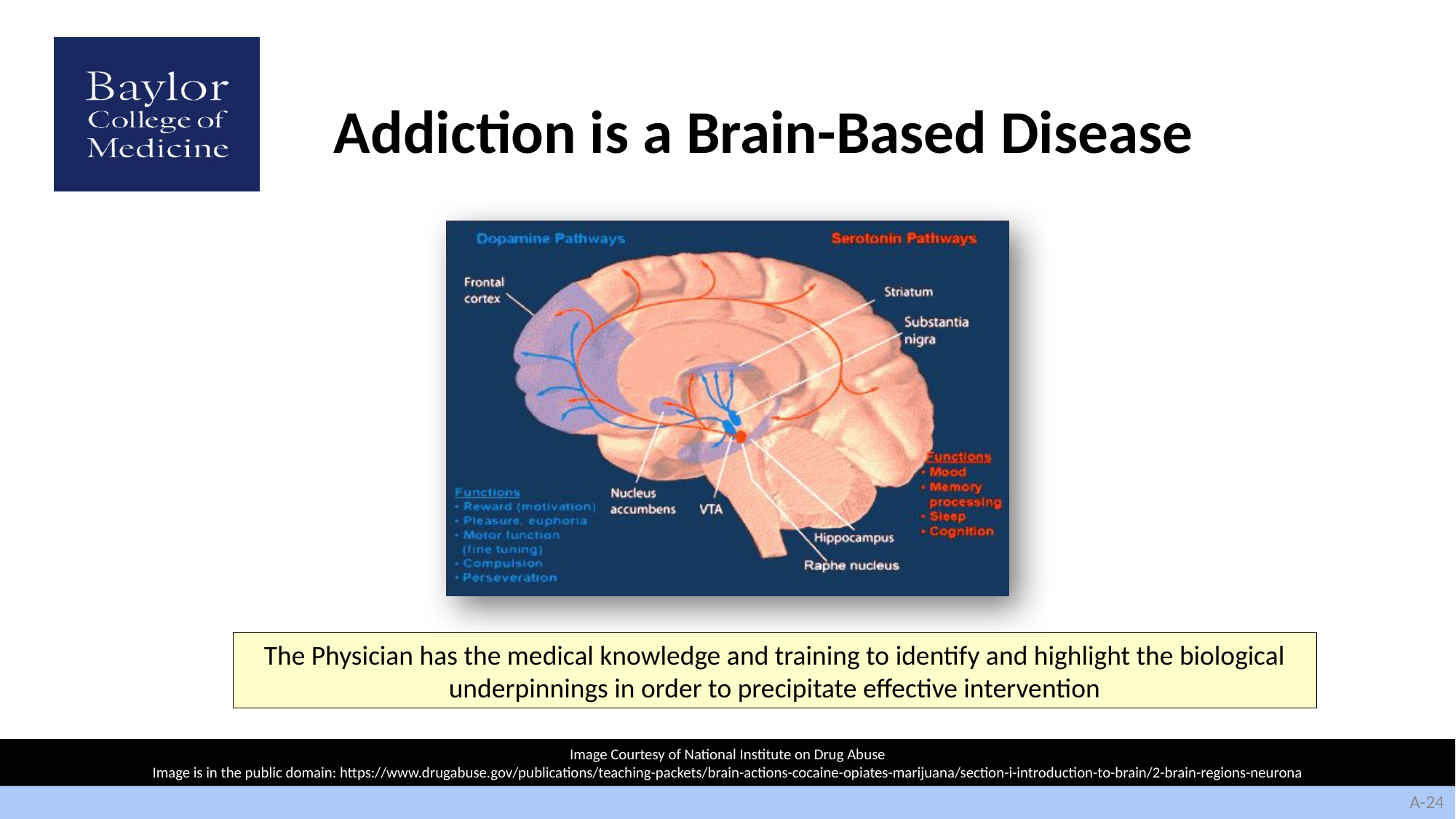

Addiction is a Brain-Based Disease
The Physician has the medical knowledge and training to identify and highlight the biological underpinnings in order to precipitate effective intervention
Image Courtesy of National Institute on Drug Abuse
Image is in the public domain: https://www.drugabuse.gov/publications/teaching-packets/brain-actions-cocaine-opiates-marijuana/section-i-introduction-to-brain/2-brain-regions-neurona
A-24

## Slide 25
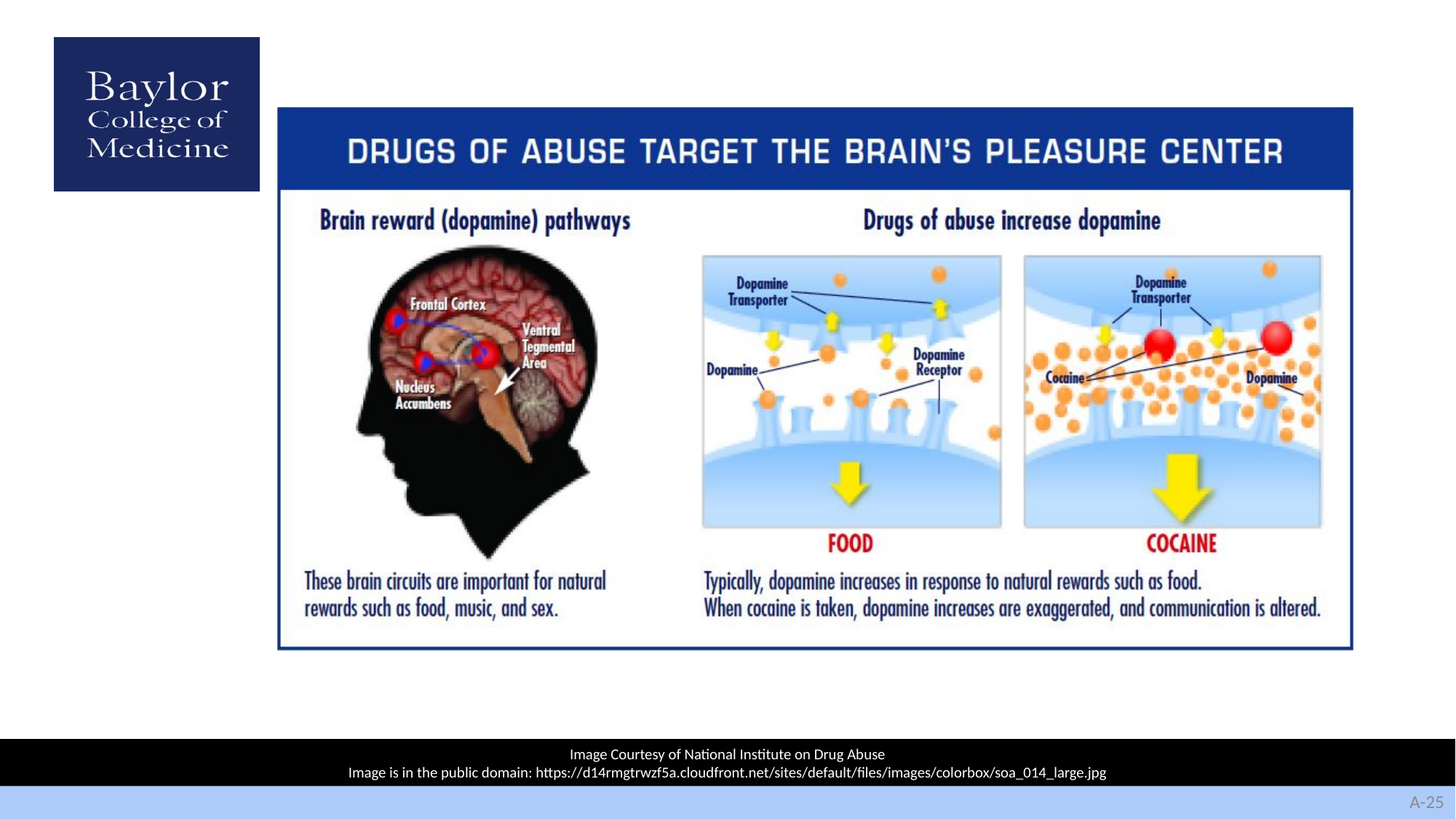

Image Courtesy of National Institute on Drug Abuse
Image is in the public domain: https://d14rmgtrwzf5a.cloudfront.net/sites/default/files/images/colorbox/soa_014_large.jpg
A-25

## Slide 26
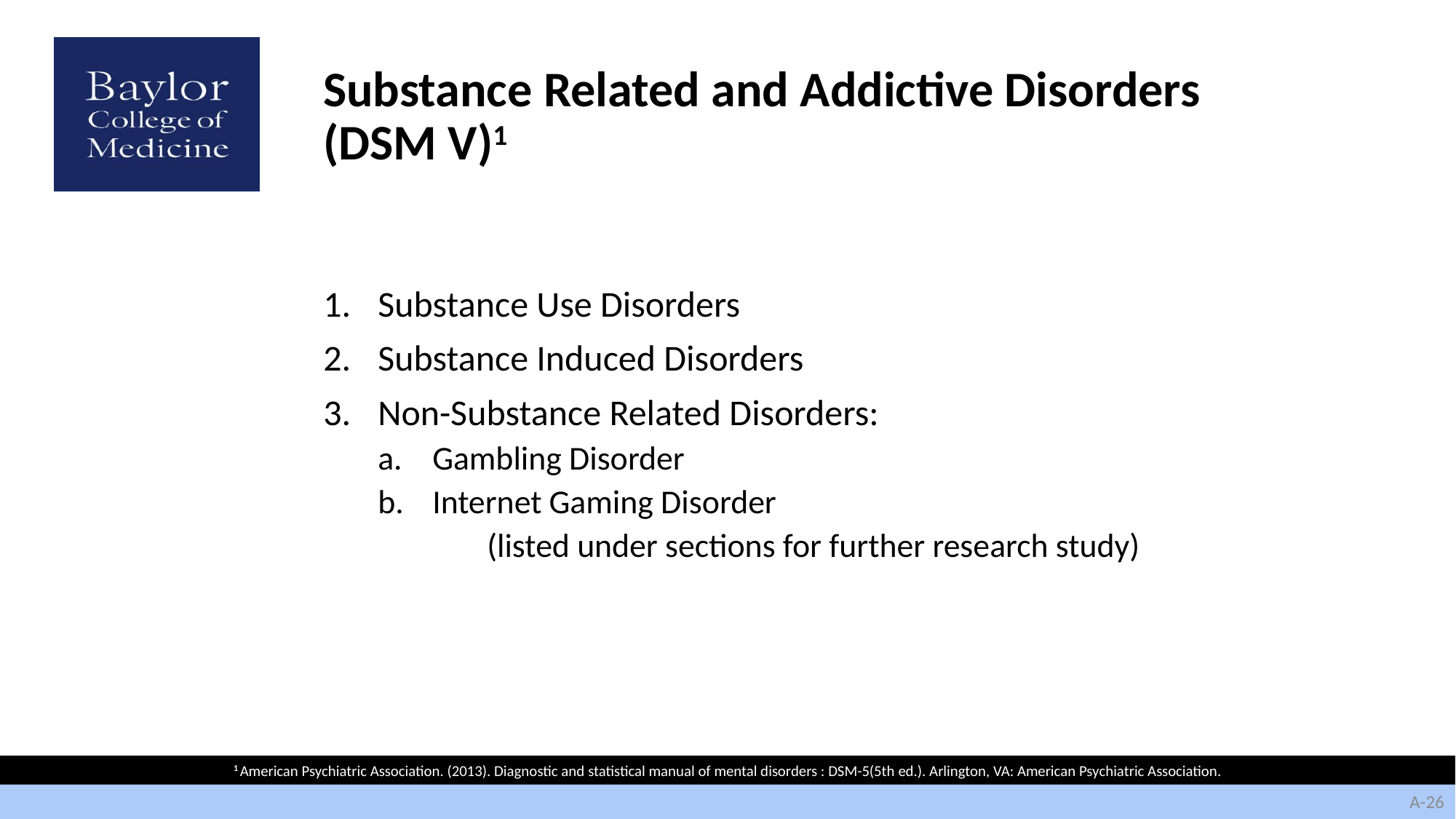

Substance Related and Addictive Disorders
(DSM V)1
Substance Use Disorders
Substance Induced Disorders
Non-Substance Related Disorders:
Gambling Disorder
Internet Gaming Disorder
	(listed under sections for further research study)
1 American Psychiatric Association. (2013). Diagnostic and statistical manual of mental disorders : DSM-5(5th ed.). Arlington, VA: American Psychiatric Association.
A-26

## Slide 27
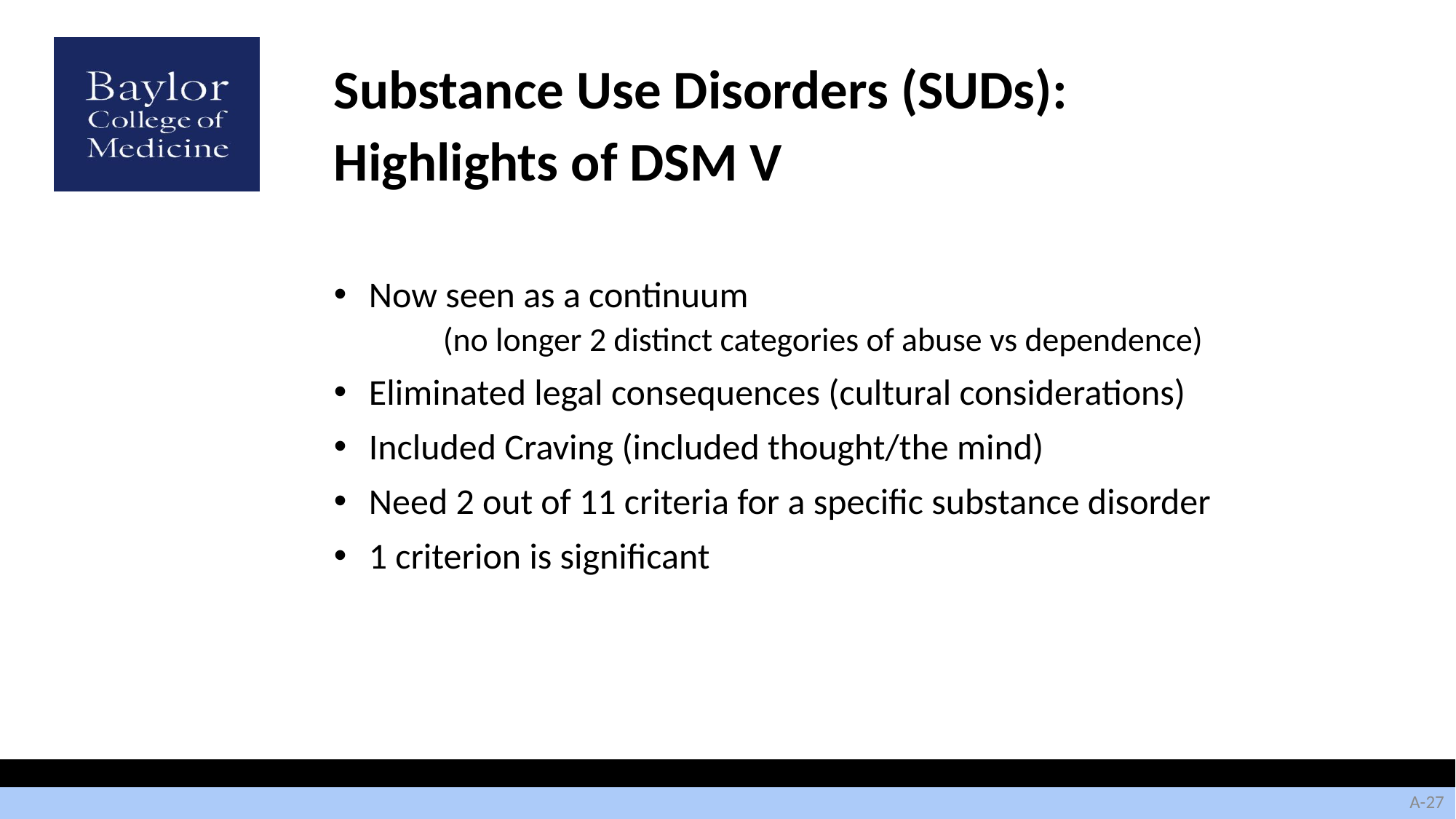

Substance Use Disorders (SUDs):
Highlights of DSM V
Now seen as a continuum
	(no longer 2 distinct categories of abuse vs dependence)
Eliminated legal consequences (cultural considerations)
Included Craving (included thought/the mind)
Need 2 out of 11 criteria for a specific substance disorder
1 criterion is significant
A-27

## Slide 28
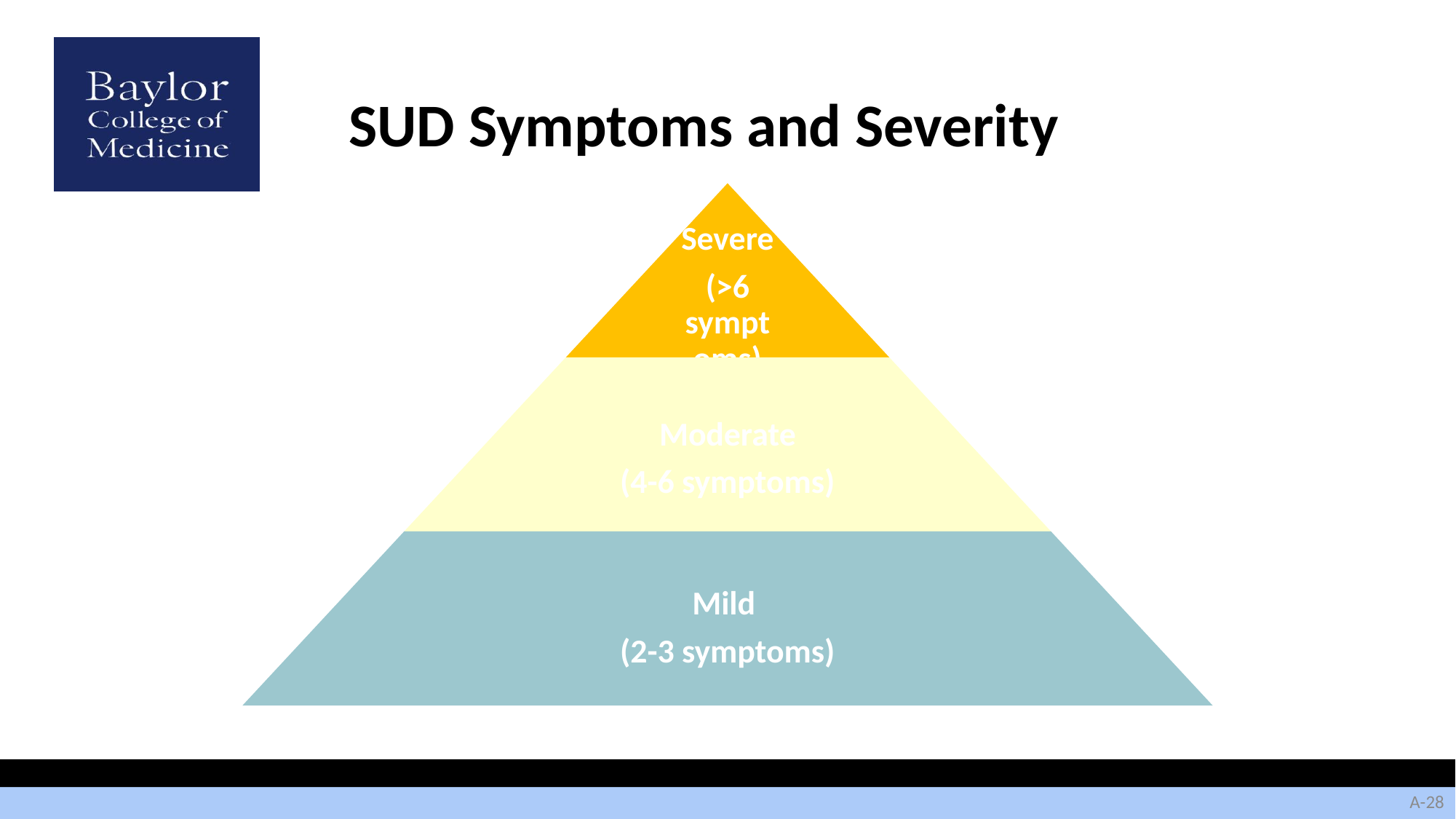

SUD Symptoms and Severity
A-28

## Slide 29
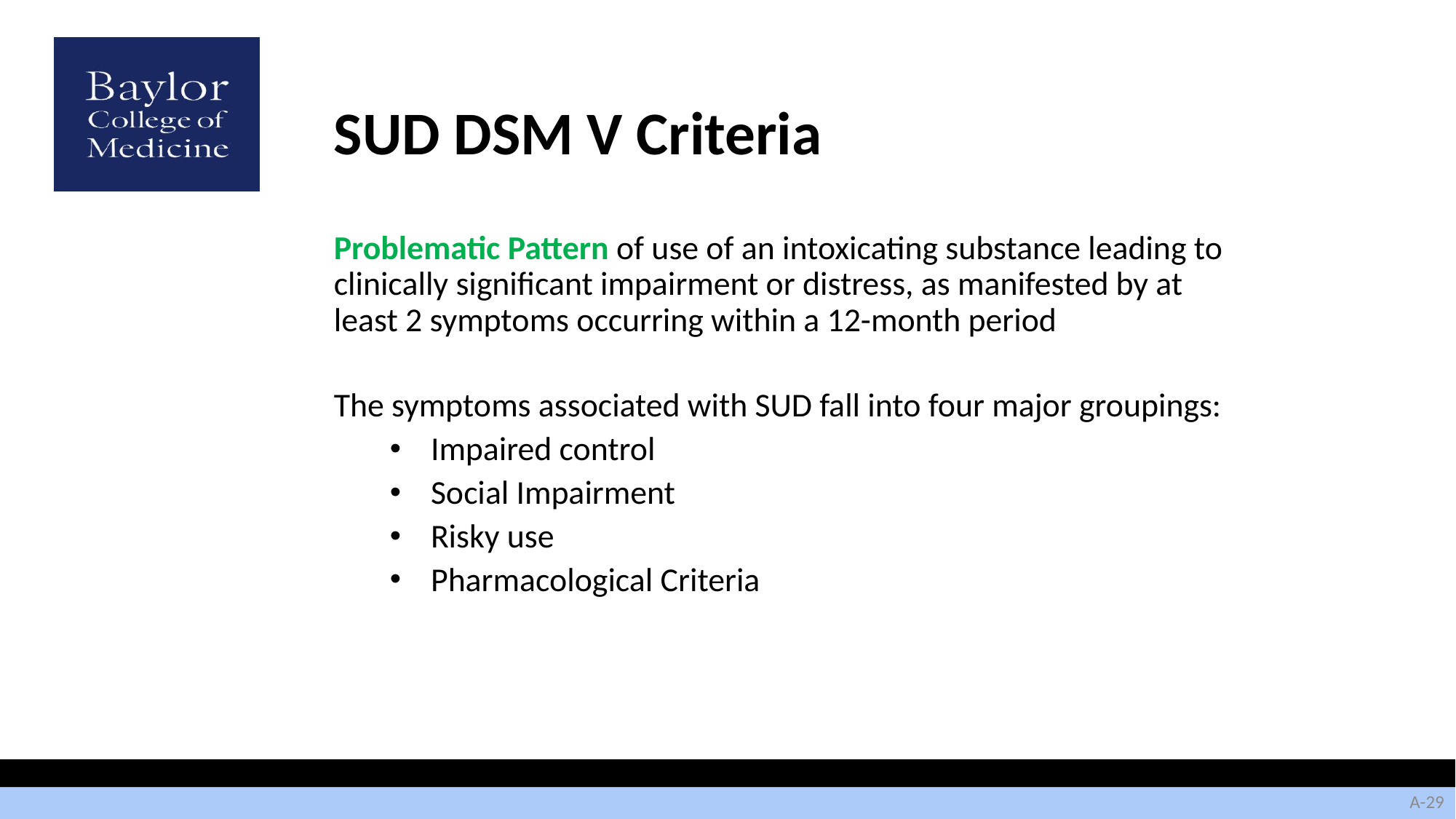

SUD DSM V Criteria
Problematic Pattern of use of an intoxicating substance leading to clinically significant impairment or distress, as manifested by at least 2 symptoms occurring within a 12-month period
The symptoms associated with SUD fall into four major groupings:
Impaired control
Social Impairment
Risky use
Pharmacological Criteria
A-29

## Slide 30
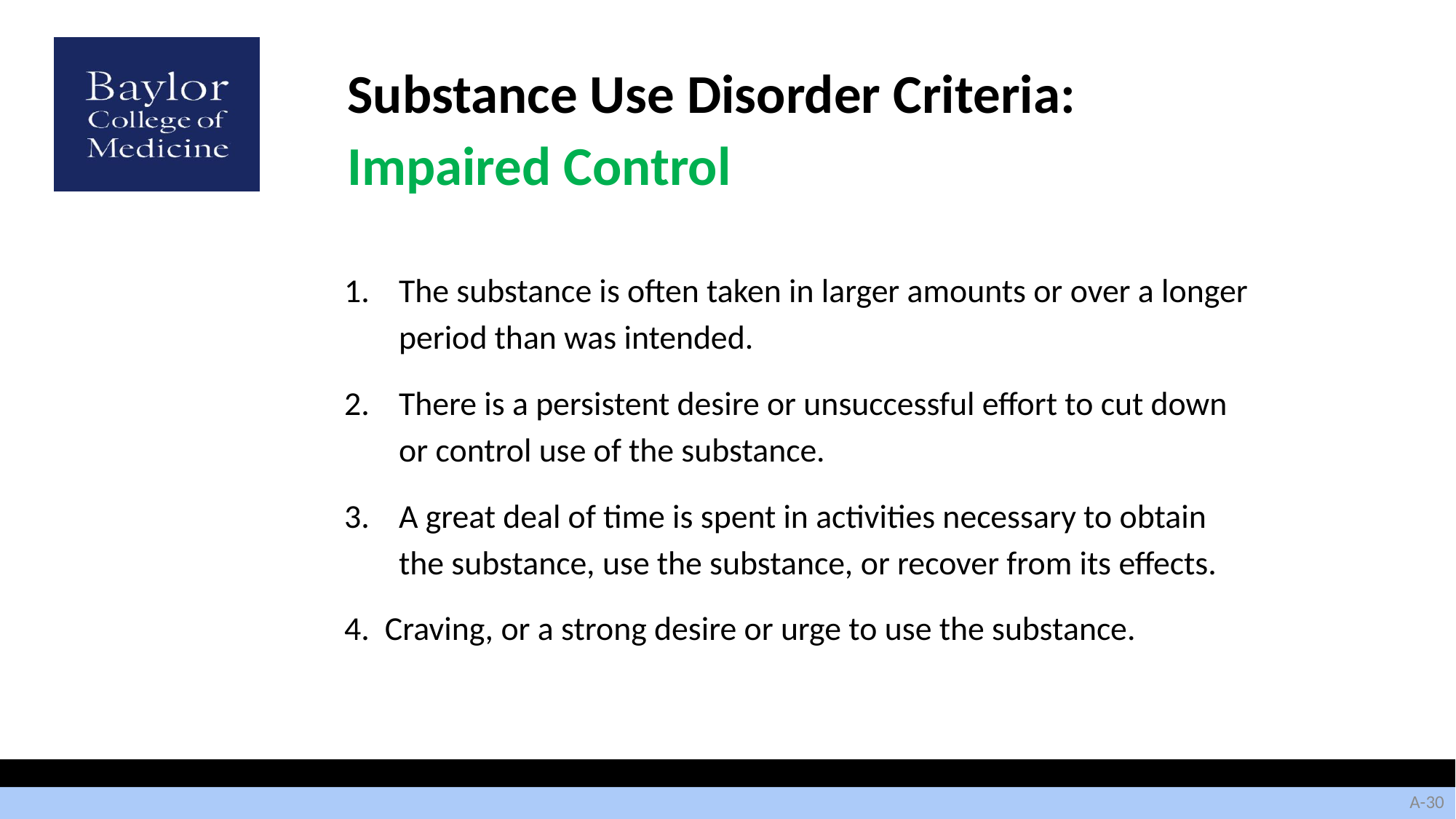

Substance Use Disorder Criteria:
Impaired Control
The substance is often taken in larger amounts or over a longer period than was intended.
There is a persistent desire or unsuccessful effort to cut down or control use of the substance.
A great deal of time is spent in activities necessary to obtain the substance, use the substance, or recover from its effects.
4.	Craving, or a strong desire or urge to use the substance.
A-30

## Slide 31
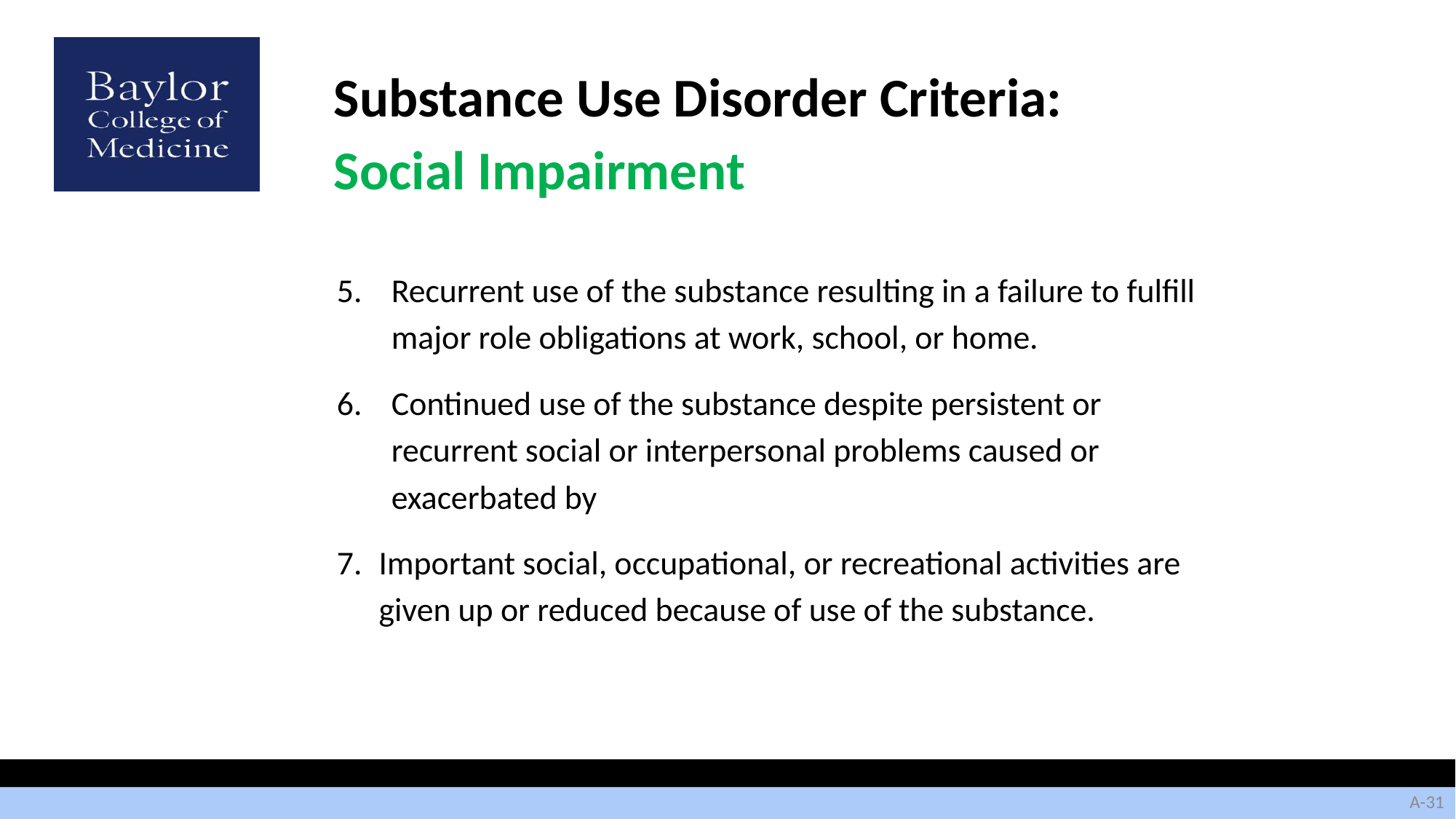

Substance Use Disorder Criteria:
Social Impairment
Recurrent use of the substance resulting in a failure to fulfill major role obligations at work, school, or home.
Continued use of the substance despite persistent or recurrent social or interpersonal problems caused or exacerbated by
7.	Important social, occupational, or recreational activities are given up or reduced because of use of the substance.
A-31

## Slide 32
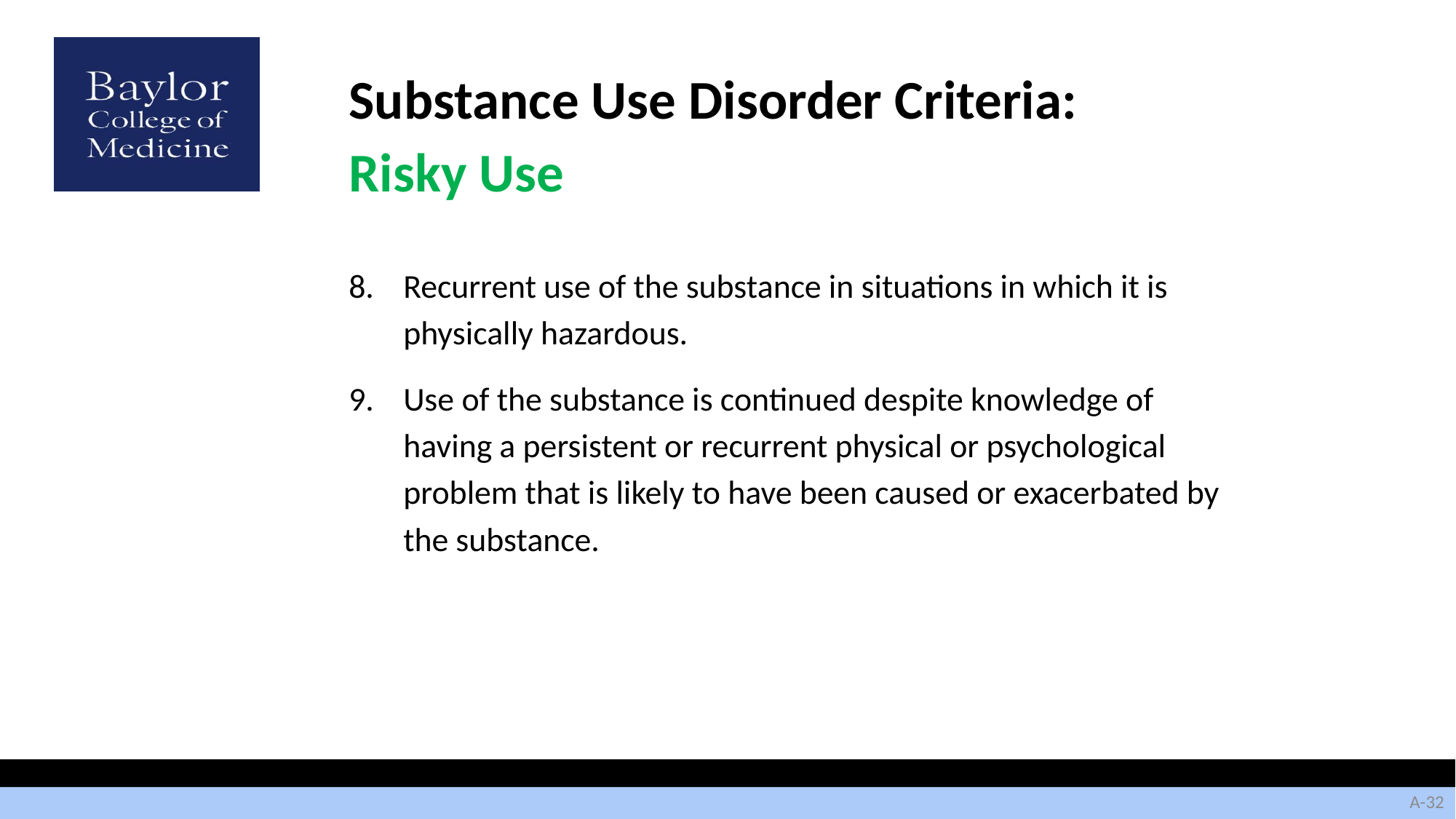

Substance Use Disorder Criteria:
Risky Use
Recurrent use of the substance in situations in which it is physically hazardous.
9.	Use of the substance is continued despite knowledge of having a persistent or recurrent physical or psychological problem that is likely to have been caused or exacerbated by the substance.
A-32

## Slide 33
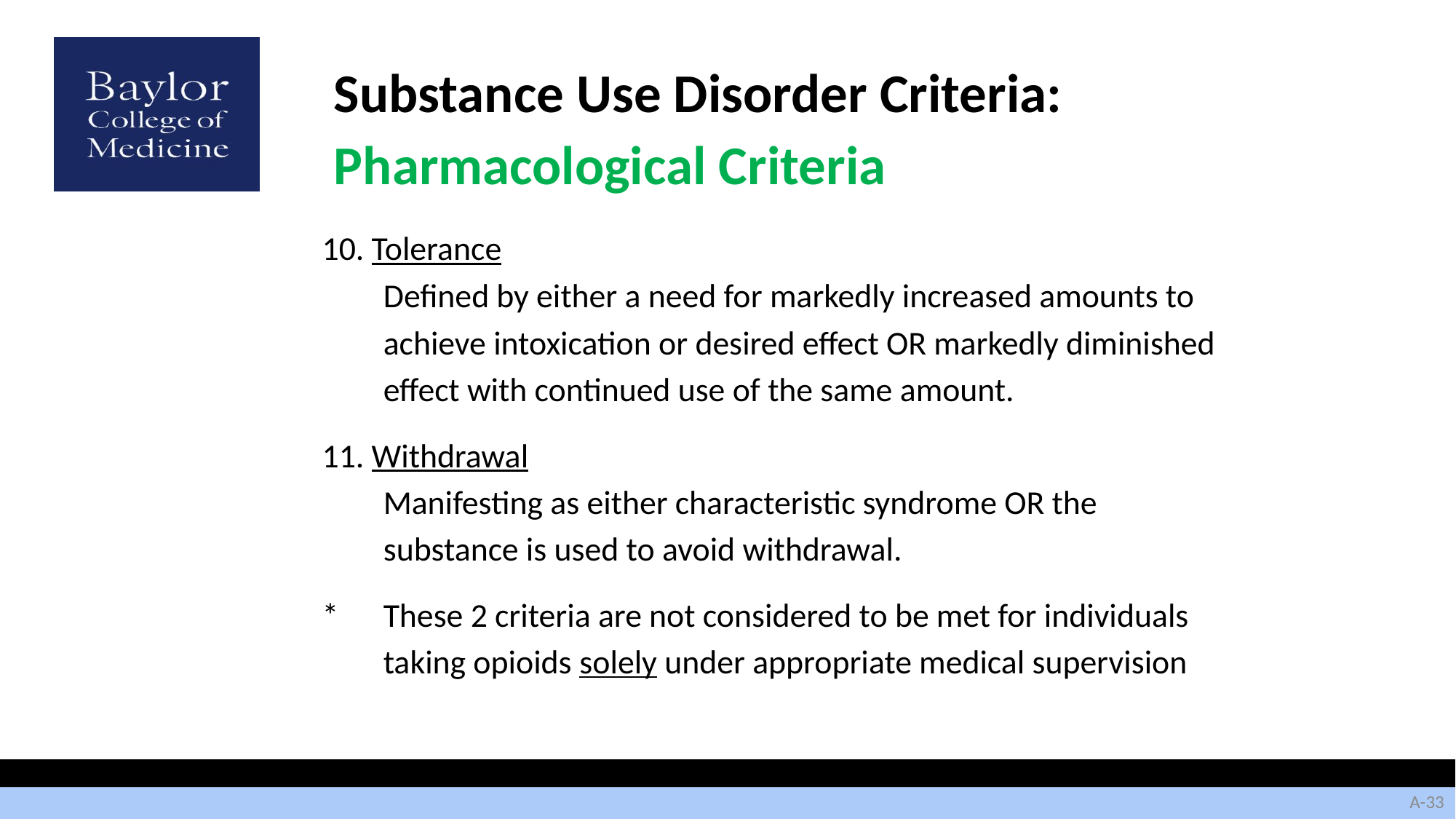

Substance Use Disorder Criteria:
Pharmacological Criteria
10. Tolerance
	Defined by either a need for markedly increased amounts to achieve intoxication or desired effect OR markedly diminished effect with continued use of the same amount.
11. Withdrawal
	Manifesting as either characteristic syndrome OR the substance is used to avoid withdrawal.
* 	These 2 criteria are not considered to be met for individuals taking opioids solely under appropriate medical supervision
A-33

## Slide 34
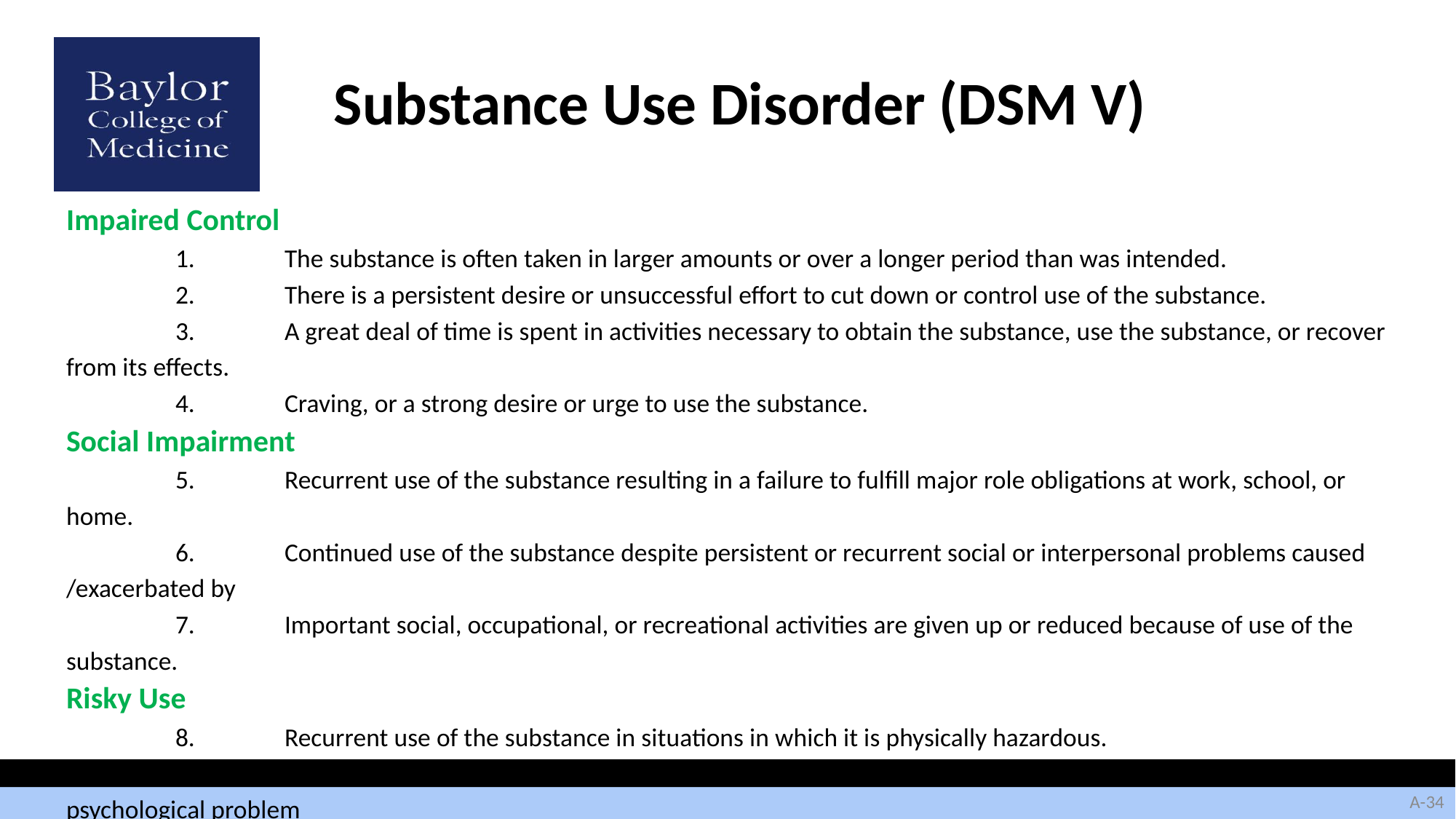

Substance Use Disorder (DSM V)
Impaired Control
	1.	The substance is often taken in larger amounts or over a longer period than was intended.
	2.	There is a persistent desire or unsuccessful effort to cut down or control use of the substance.
	3.	A great deal of time is spent in activities necessary to obtain the substance, use the substance, or recover from its effects.
	4.	Craving, or a strong desire or urge to use the substance.
Social Impairment
	5.	Recurrent use of the substance resulting in a failure to fulfill major role obligations at work, school, or home.
	6.	Continued use of the substance despite persistent or recurrent social or interpersonal problems caused /exacerbated by
	7.	Important social, occupational, or recreational activities are given up or reduced because of use of the substance.
Risky Use
	8.	Recurrent use of the substance in situations in which it is physically hazardous.
	9.	Use of the substance is continued despite knowledge of having a persistent or recurrent physical or psychological problem
Pharmacological Criteria
	10.	Tolerance
	11.	Withdrawal
A-34

## Slide 35
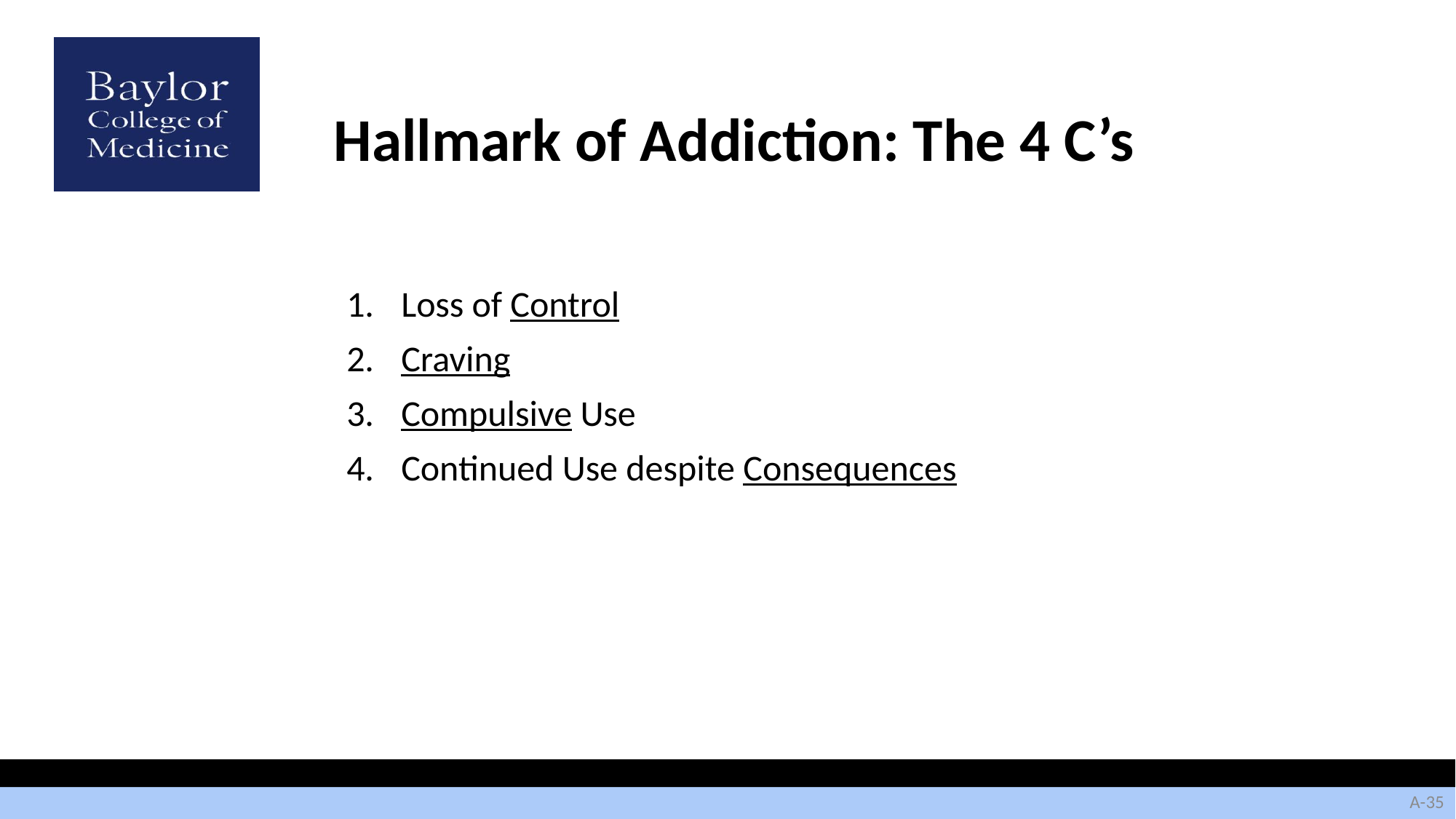

Hallmark of Addiction: The 4 C’s
Loss of Control
Craving
Compulsive Use
Continued Use despite Consequences
A-35

## Slide 36
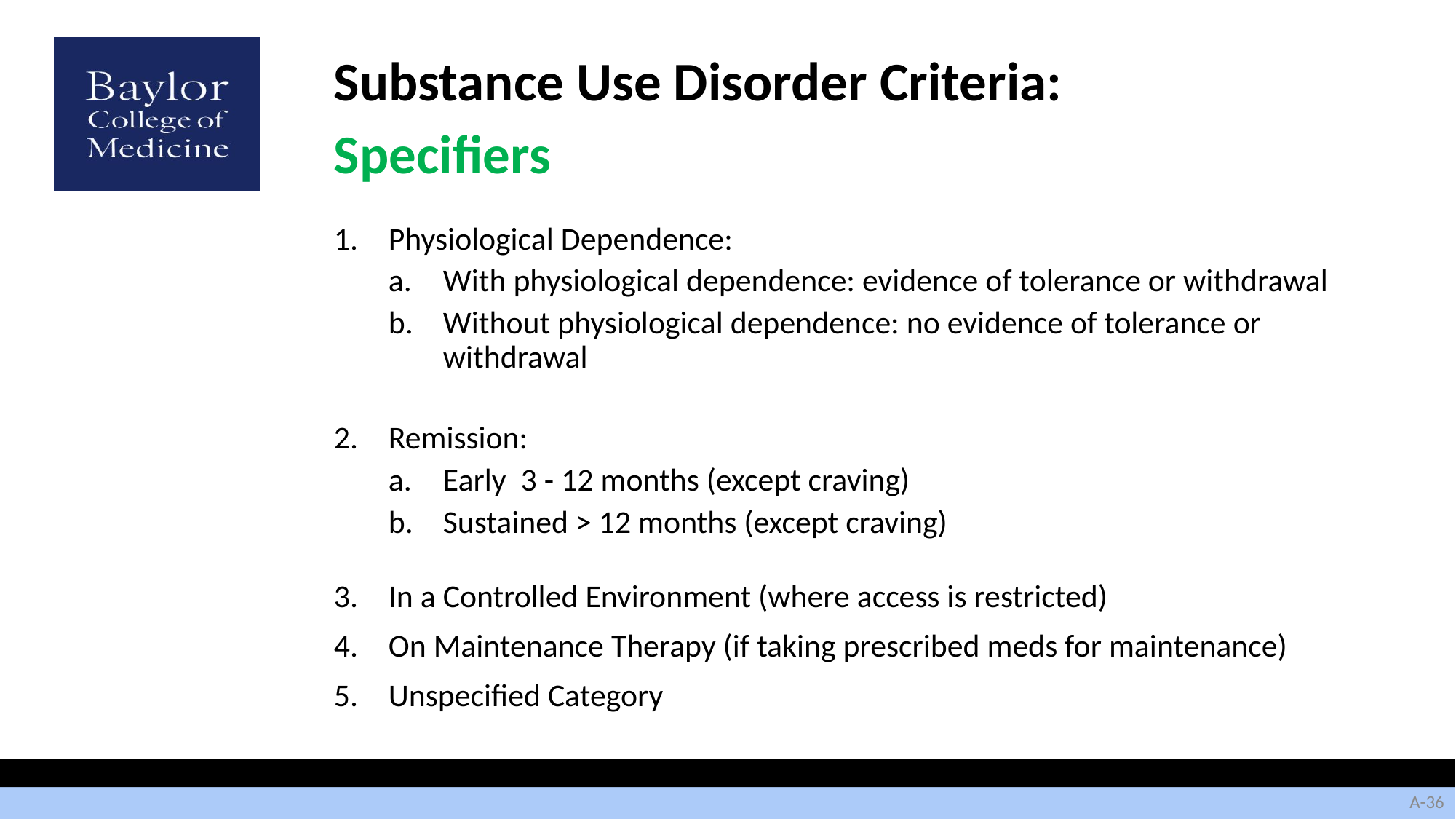

Substance Use Disorder Criteria:
Specifiers
Physiological Dependence:
With physiological dependence: evidence of tolerance or withdrawal
Without physiological dependence: no evidence of tolerance or withdrawal
Remission:
Early 3 - 12 months (except craving)
Sustained > 12 months (except craving)
In a Controlled Environment (where access is restricted)
On Maintenance Therapy (if taking prescribed meds for maintenance)
Unspecified Category
A-36

## Slide 37
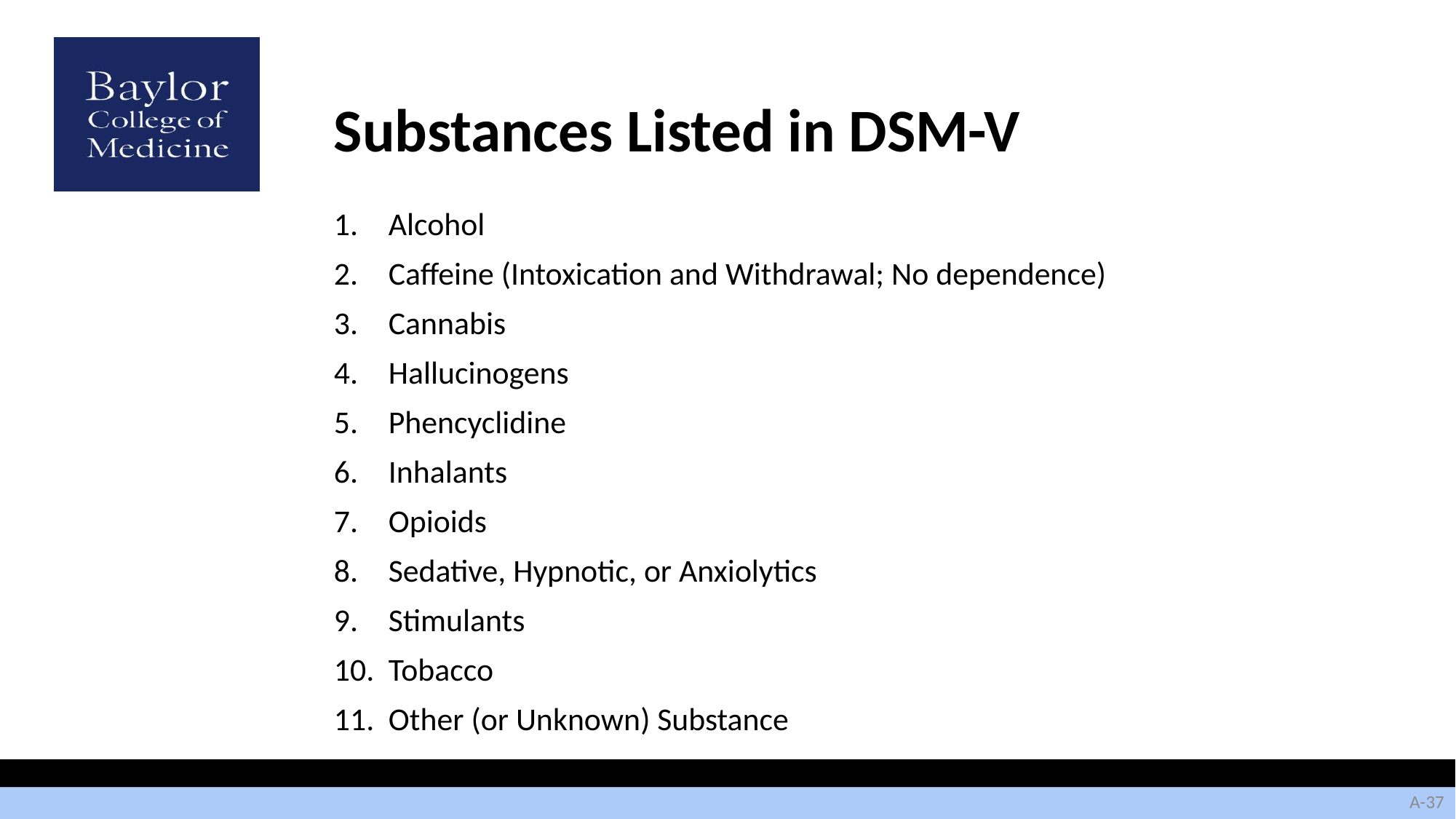

Substances Listed in DSM-V
Alcohol
Caffeine (Intoxication and Withdrawal; No dependence)
Cannabis
Hallucinogens
Phencyclidine
Inhalants
Opioids
Sedative, Hypnotic, or Anxiolytics
Stimulants
Tobacco
Other (or Unknown) Substance
A-37

## Slide 38
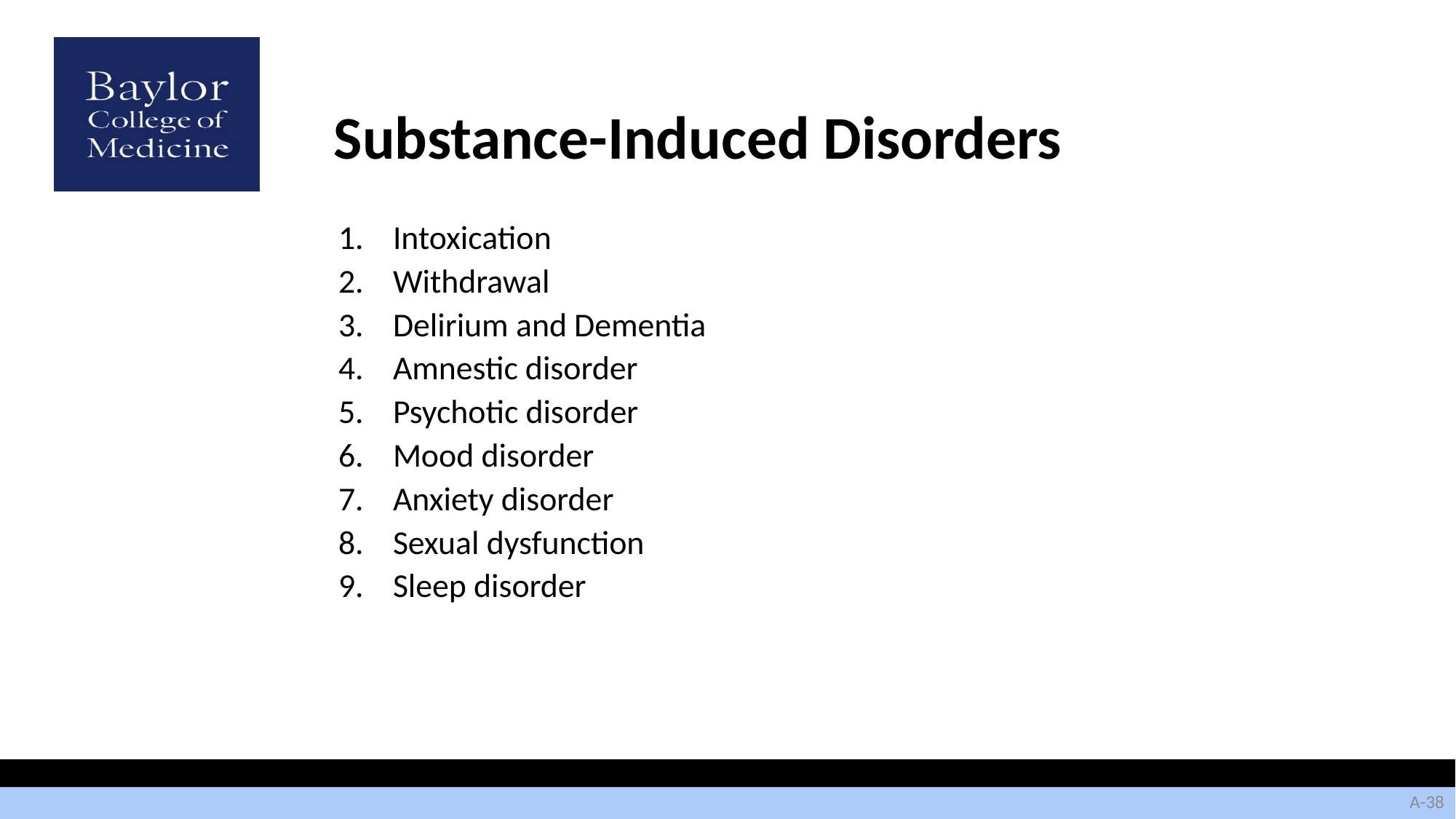

Substance-Induced Disorders
Intoxication
Withdrawal
Delirium and Dementia
Amnestic disorder
Psychotic disorder
Mood disorder
Anxiety disorder
Sexual dysfunction
Sleep disorder
A-38

## Slide 39
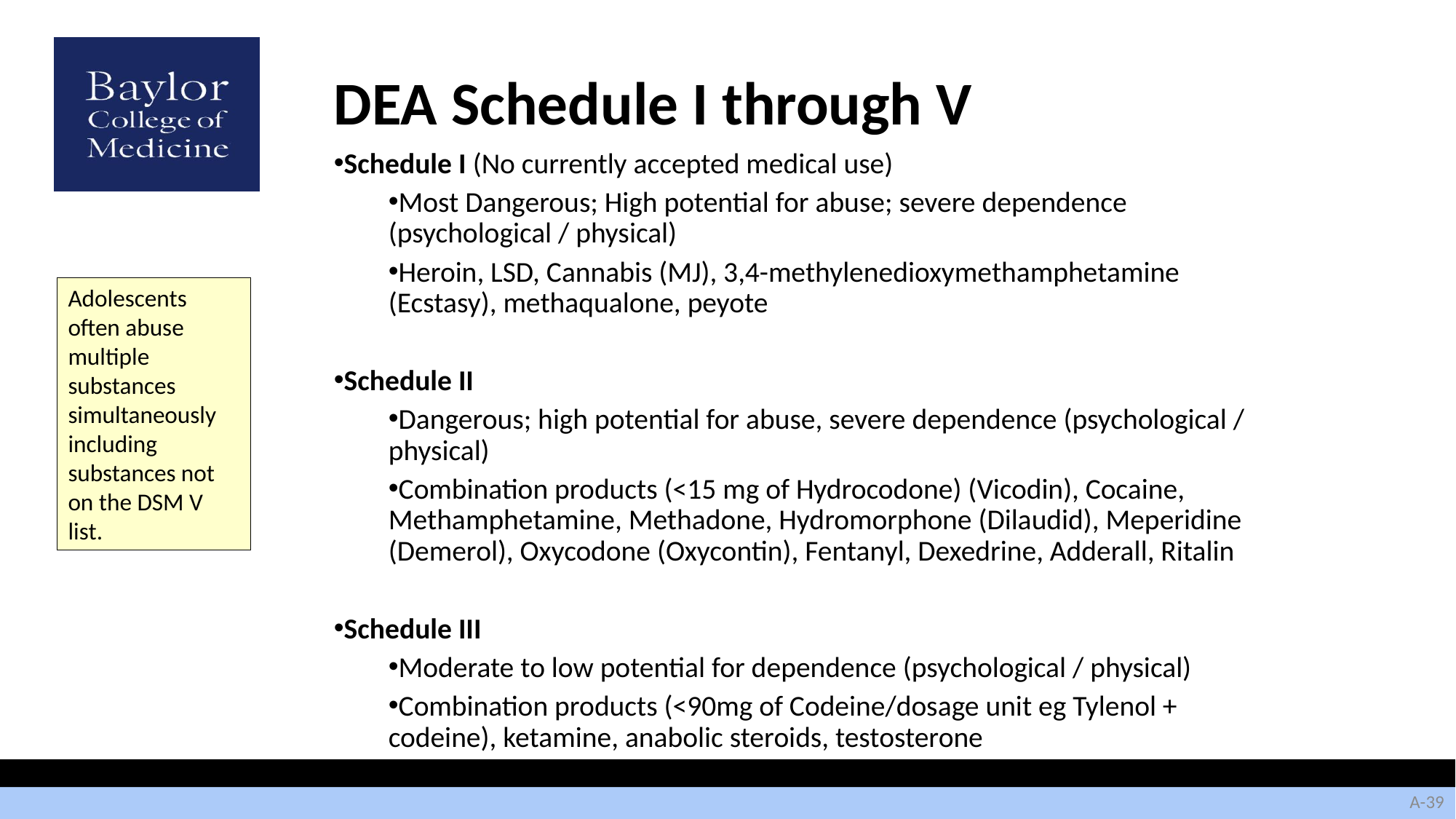

DEA Schedule I through V
Schedule I (No currently accepted medical use)
Most Dangerous; High potential for abuse; severe dependence (psychological / physical)
Heroin, LSD, Cannabis (MJ), 3,4-methylenedioxymethamphetamine (Ecstasy), methaqualone, peyote
Schedule II
Dangerous; high potential for abuse, severe dependence (psychological / physical)
Combination products (<15 mg of Hydrocodone) (Vicodin), Cocaine, Methamphetamine, Methadone, Hydromorphone (Dilaudid), Meperidine (Demerol), Oxycodone (Oxycontin), Fentanyl, Dexedrine, Adderall, Ritalin
Schedule III
Moderate to low potential for dependence (psychological / physical)
Combination products (<90mg of Codeine/dosage unit eg Tylenol + codeine), ketamine, anabolic steroids, testosterone
Adolescents often abuse multiple substances simultaneously including substances not on the DSM V list.
A-39

## Slide 40
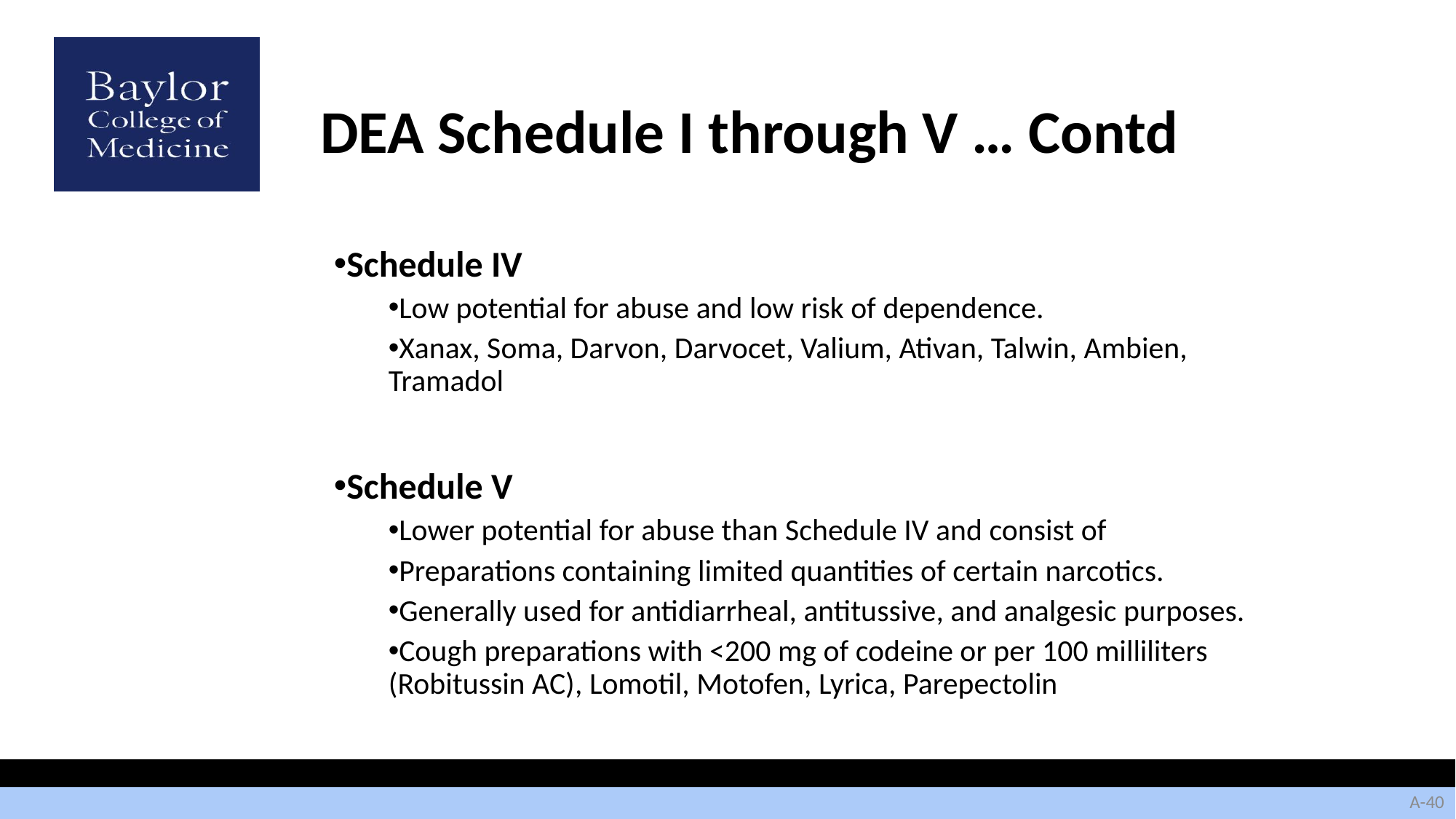

DEA Schedule I through V … Contd
Schedule IV
Low potential for abuse and low risk of dependence.
Xanax, Soma, Darvon, Darvocet, Valium, Ativan, Talwin, Ambien, Tramadol
Schedule V
Lower potential for abuse than Schedule IV and consist of
Preparations containing limited quantities of certain narcotics.
Generally used for antidiarrheal, antitussive, and analgesic purposes.
Cough preparations with <200 mg of codeine or per 100 milliliters (Robitussin AC), Lomotil, Motofen, Lyrica, Parepectolin
A-40

## Slide 41
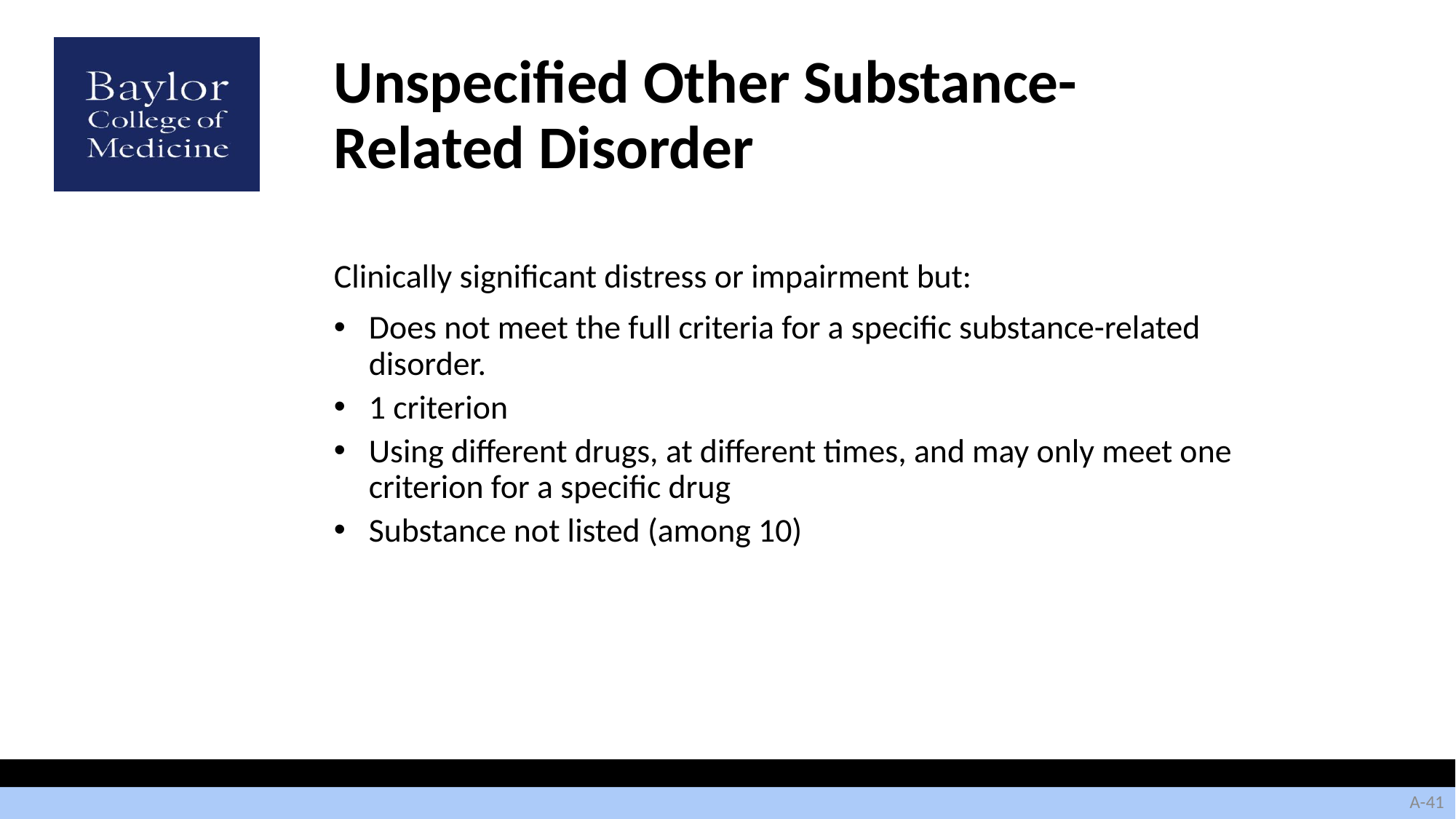

Unspecified Other Substance-Related Disorder
Clinically significant distress or impairment but:
Does not meet the full criteria for a specific substance-related disorder.
1 criterion
Using different drugs, at different times, and may only meet one criterion for a specific drug
Substance not listed (among 10)
A-41

## Slide 42
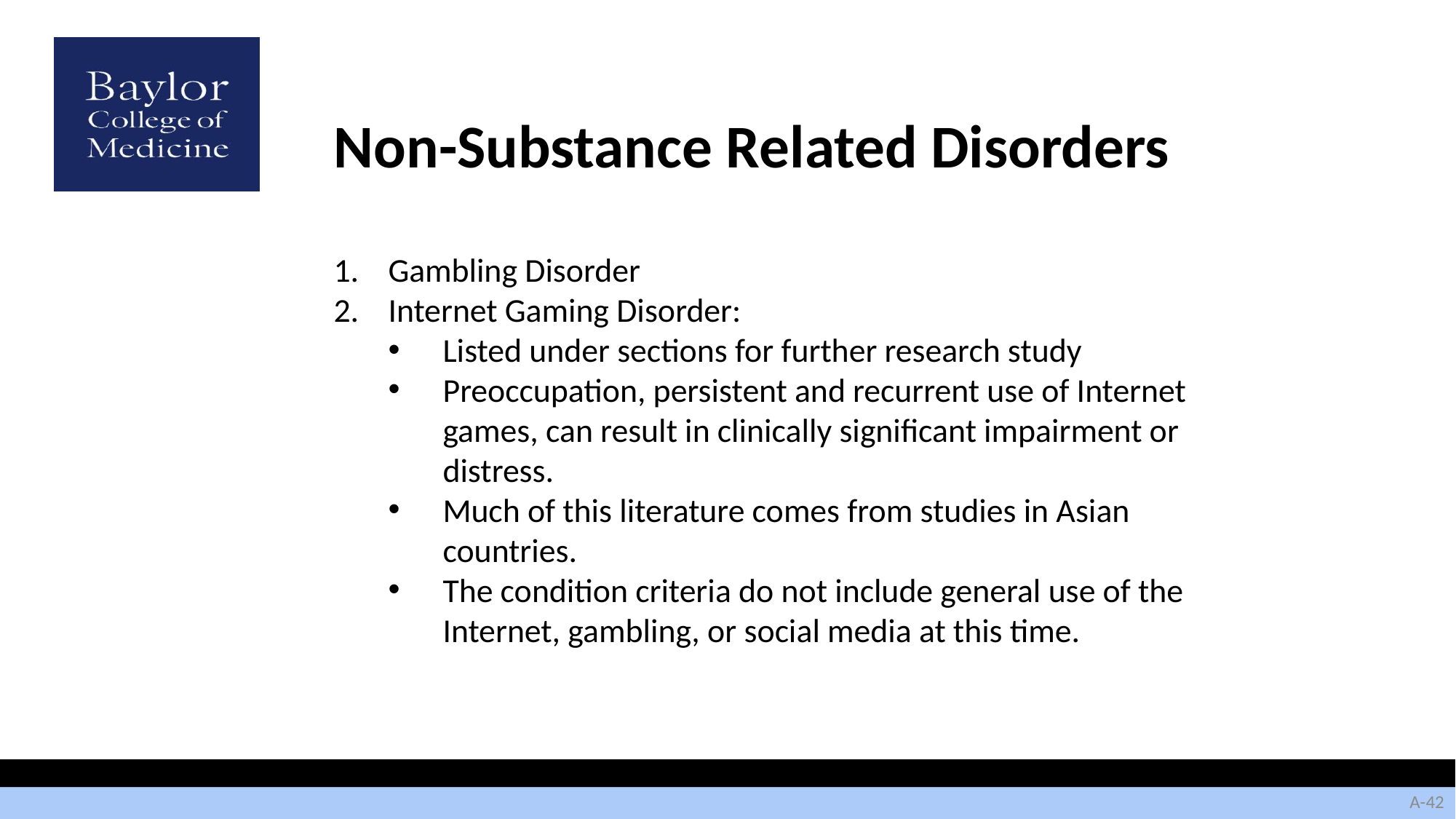

Non-Substance Related Disorders
Gambling Disorder
Internet Gaming Disorder:
Listed under sections for further research study
Preoccupation, persistent and recurrent use of Internet games, can result in clinically significant impairment or distress.
Much of this literature comes from studies in Asian countries.
The condition criteria do not include general use of the Internet, gambling, or social media at this time.
A-42

## Slide 43
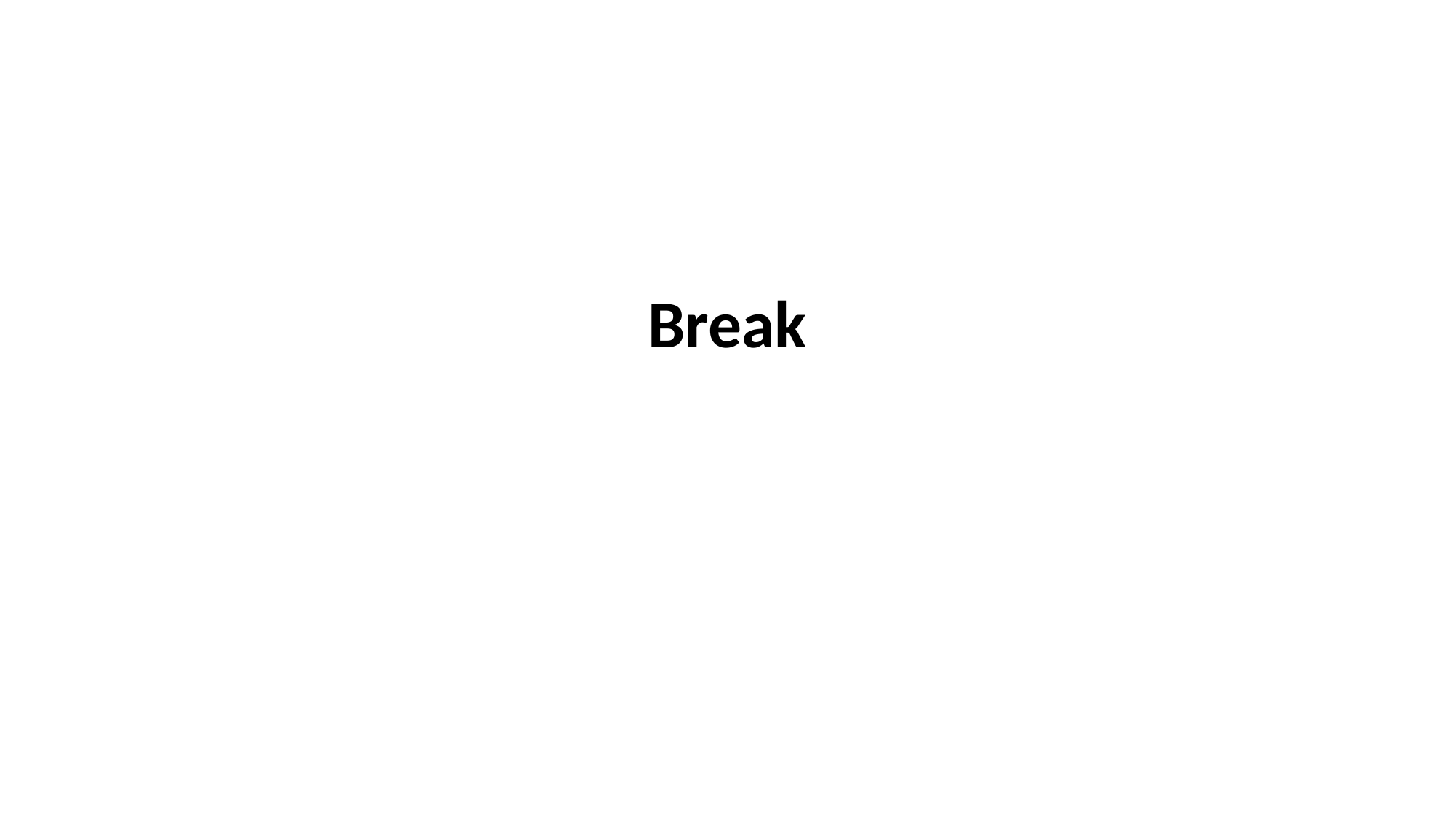

# Break

## Slide 44
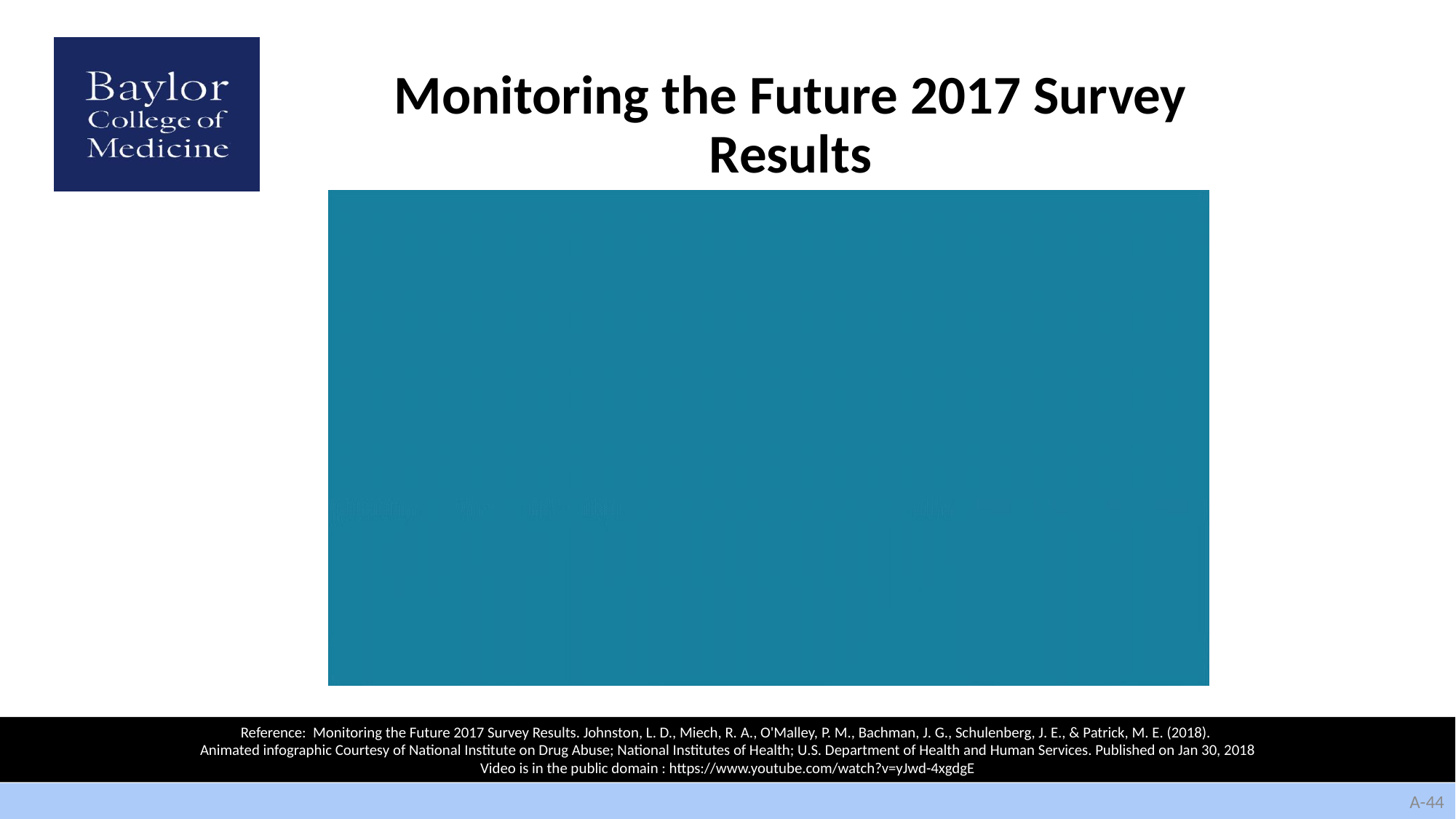

Monitoring the Future 2017 Survey Results
Reference:  Monitoring the Future 2017 Survey Results. Johnston, L. D., Miech, R. A., O'Malley, P. M., Bachman, J. G., Schulenberg, J. E., & Patrick, M. E. (2018).
Animated infographic Courtesy of National Institute on Drug Abuse; National Institutes of Health; U.S. Department of Health and Human Services. Published on Jan 30, 2018
Video is in the public domain : https://www.youtube.com/watch?v=yJwd-4xgdgE
A-44

## Slide 45
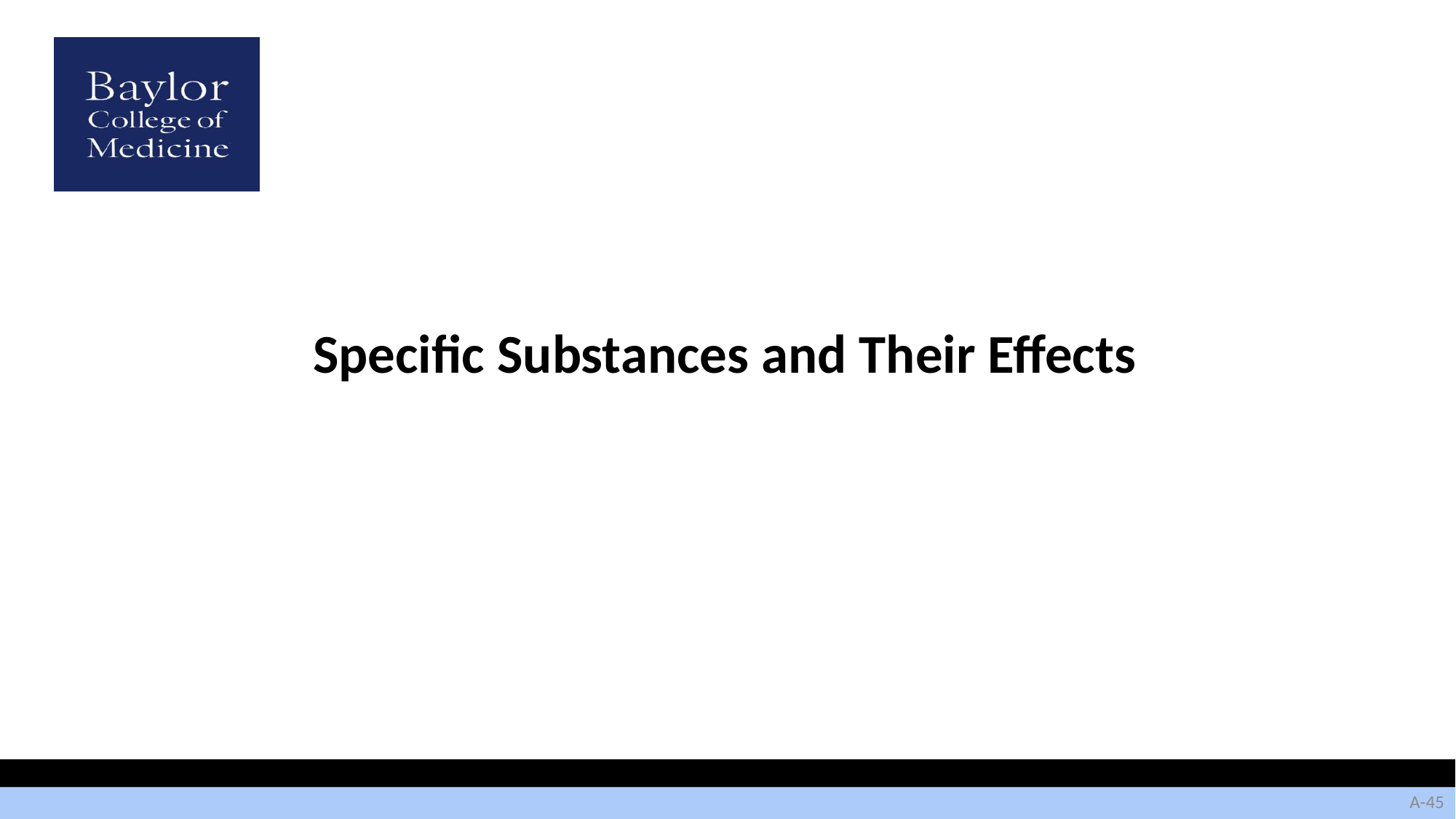

Specific Substances and Their Effects
A-45

## Slide 46
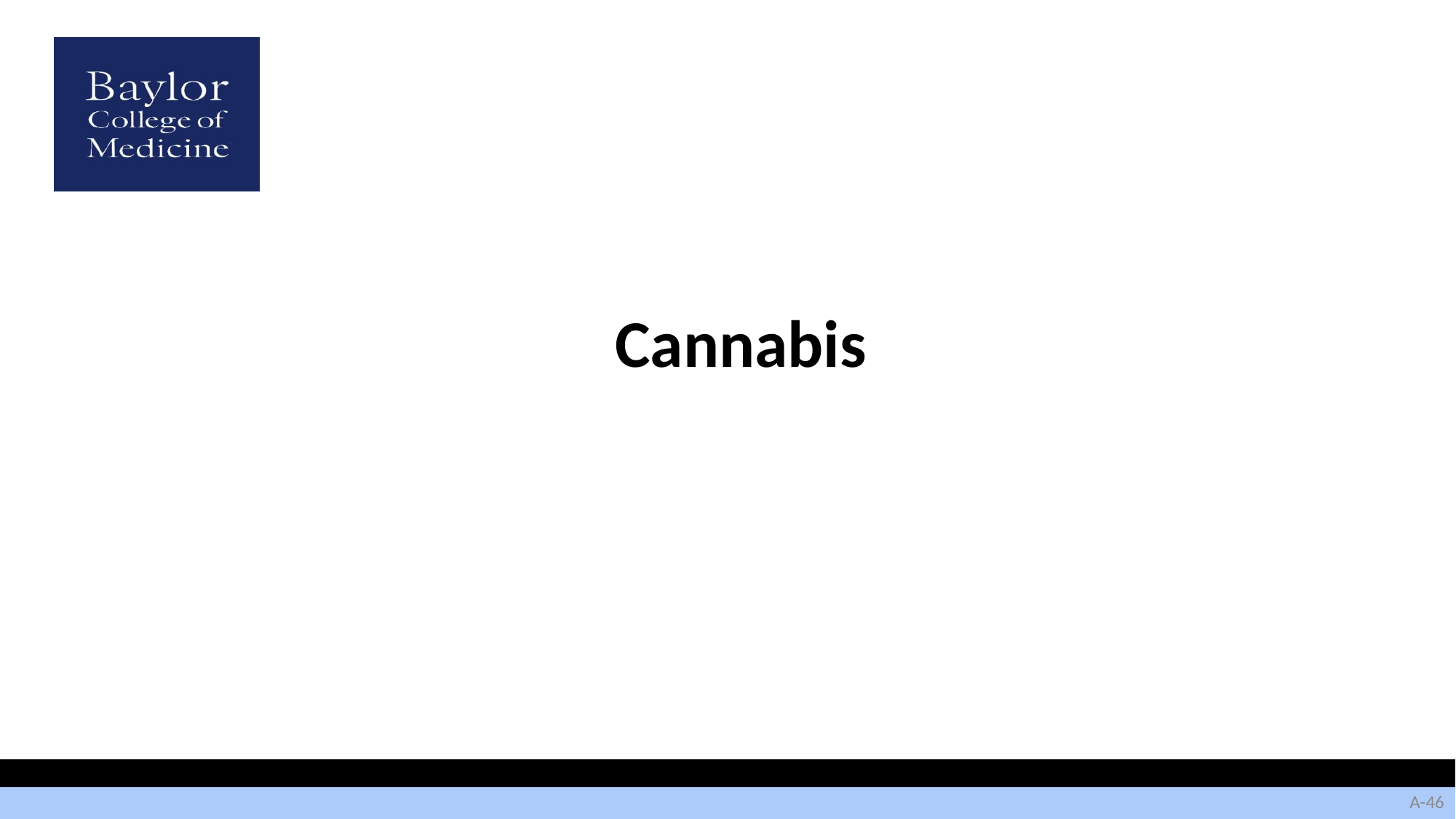

Cannabis
A-46

## Slide 47
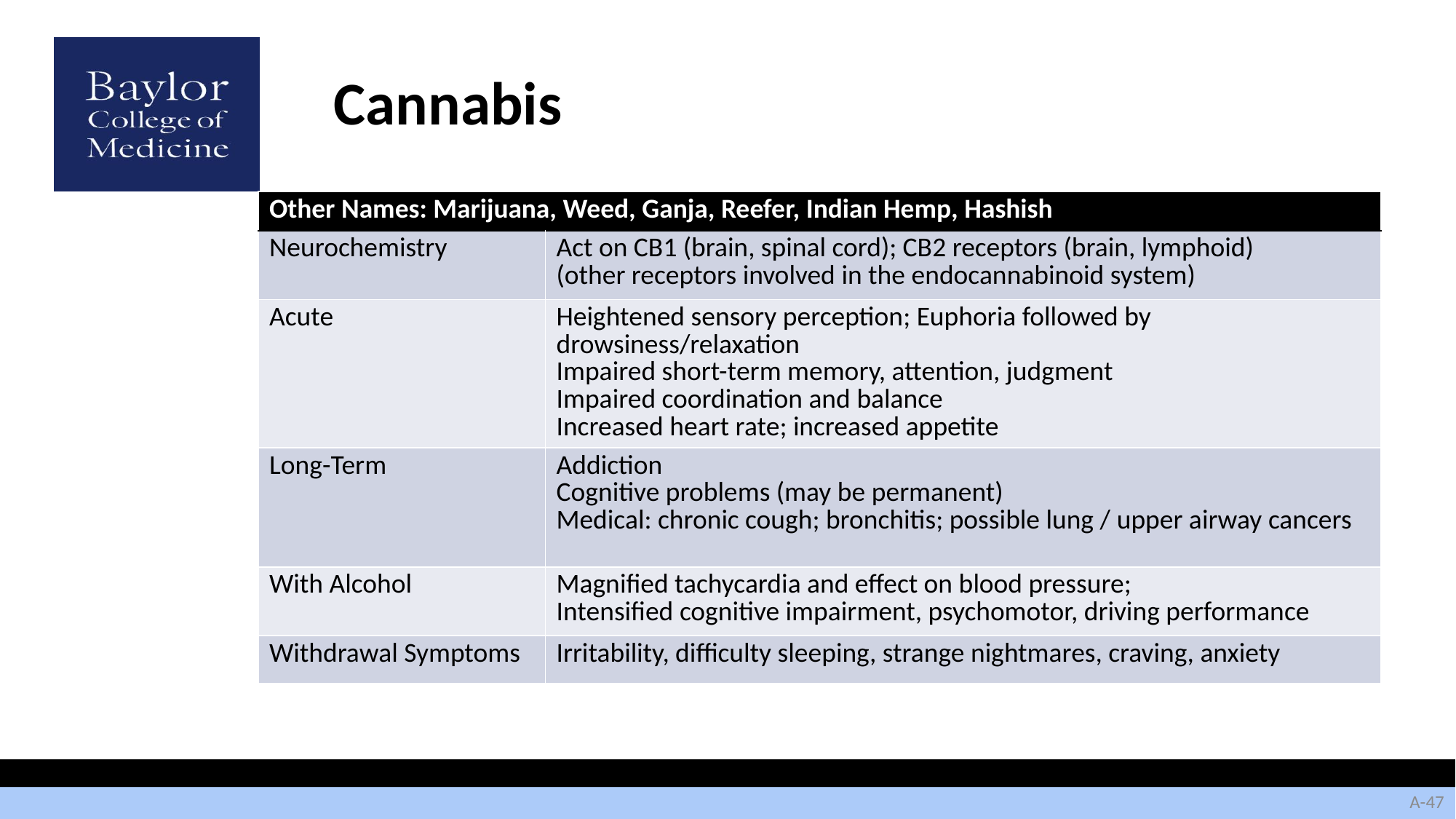

Cannabis
| Other Names: Marijuana, Weed, Ganja, Reefer, Indian Hemp, Hashish | |
| --- | --- |
| Neurochemistry | Act on CB1 (brain, spinal cord); CB2 receptors (brain, lymphoid) (other receptors involved in the endocannabinoid system) |
| Acute | Heightened sensory perception; Euphoria followed by drowsiness/relaxation Impaired short-term memory, attention, judgment Impaired coordination and balance Increased heart rate; increased appetite |
| Long-Term | Addiction Cognitive problems (may be permanent) Medical: chronic cough; bronchitis; possible lung / upper airway cancers |
| With Alcohol | Magnified tachycardia and effect on blood pressure;  Intensified cognitive impairment, psychomotor, driving performance |
| Withdrawal Symptoms | Irritability, difficulty sleeping, strange nightmares, craving, anxiety |
A-47

## Slide 48
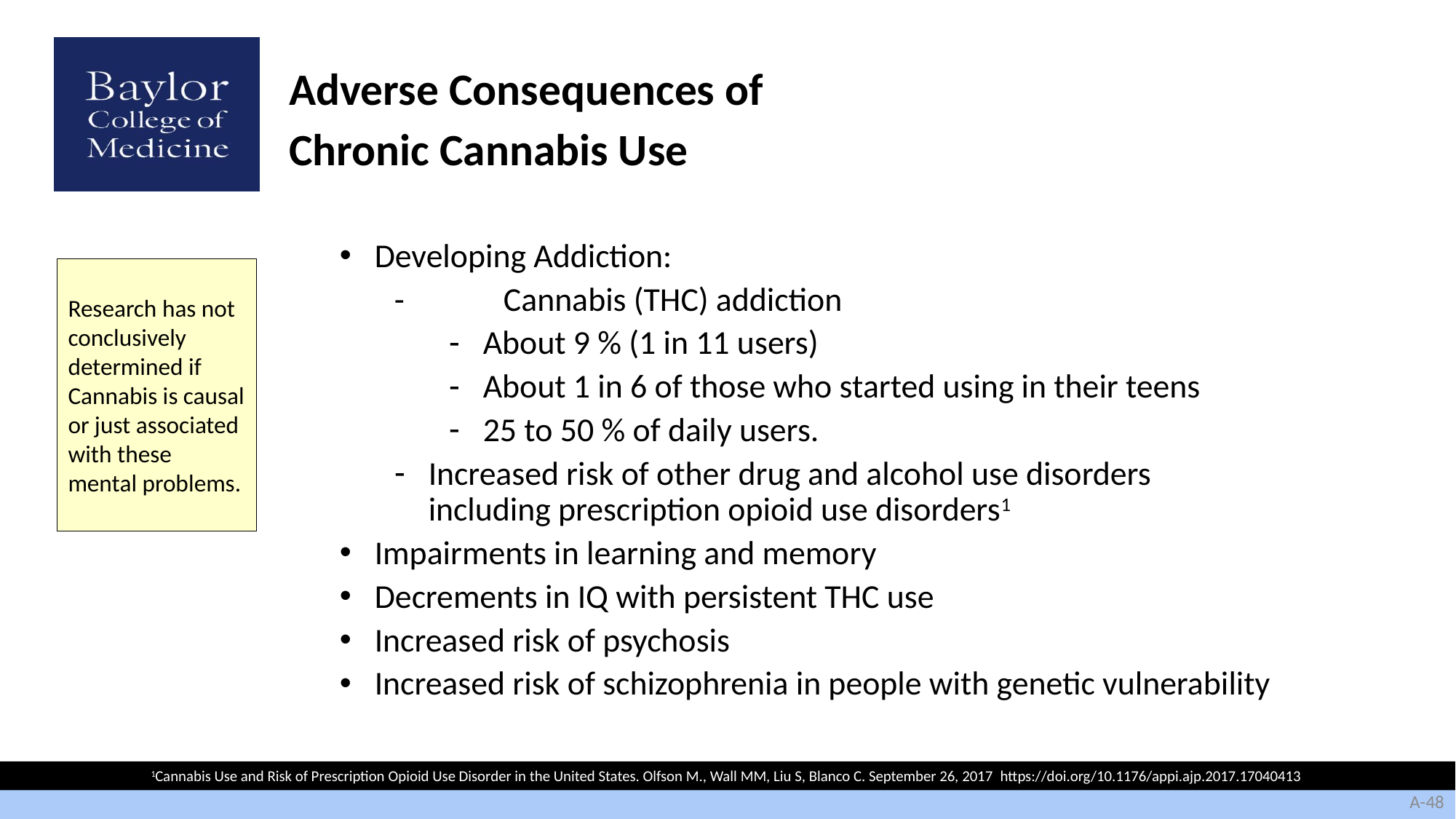

Adverse Consequences of
Chronic Cannabis Use
Developing Addiction:
- 	Cannabis (THC) addiction
About 9 % (1 in 11 users)
About 1 in 6 of those who started using in their teens
25 to 50 % of daily users.
Increased risk of other drug and alcohol use disorders including prescription opioid use disorders1
Impairments in learning and memory
Decrements in IQ with persistent THC use
Increased risk of psychosis
Increased risk of schizophrenia in people with genetic vulnerability
Research has not conclusively determined if Cannabis is causal or just associated with these mental problems.
1Cannabis Use and Risk of Prescription Opioid Use Disorder in the United States. Olfson M., Wall MM, Liu S, Blanco C. September 26, 2017  https://doi.org/10.1176/appi.ajp.2017.17040413
A-48

## Slide 49
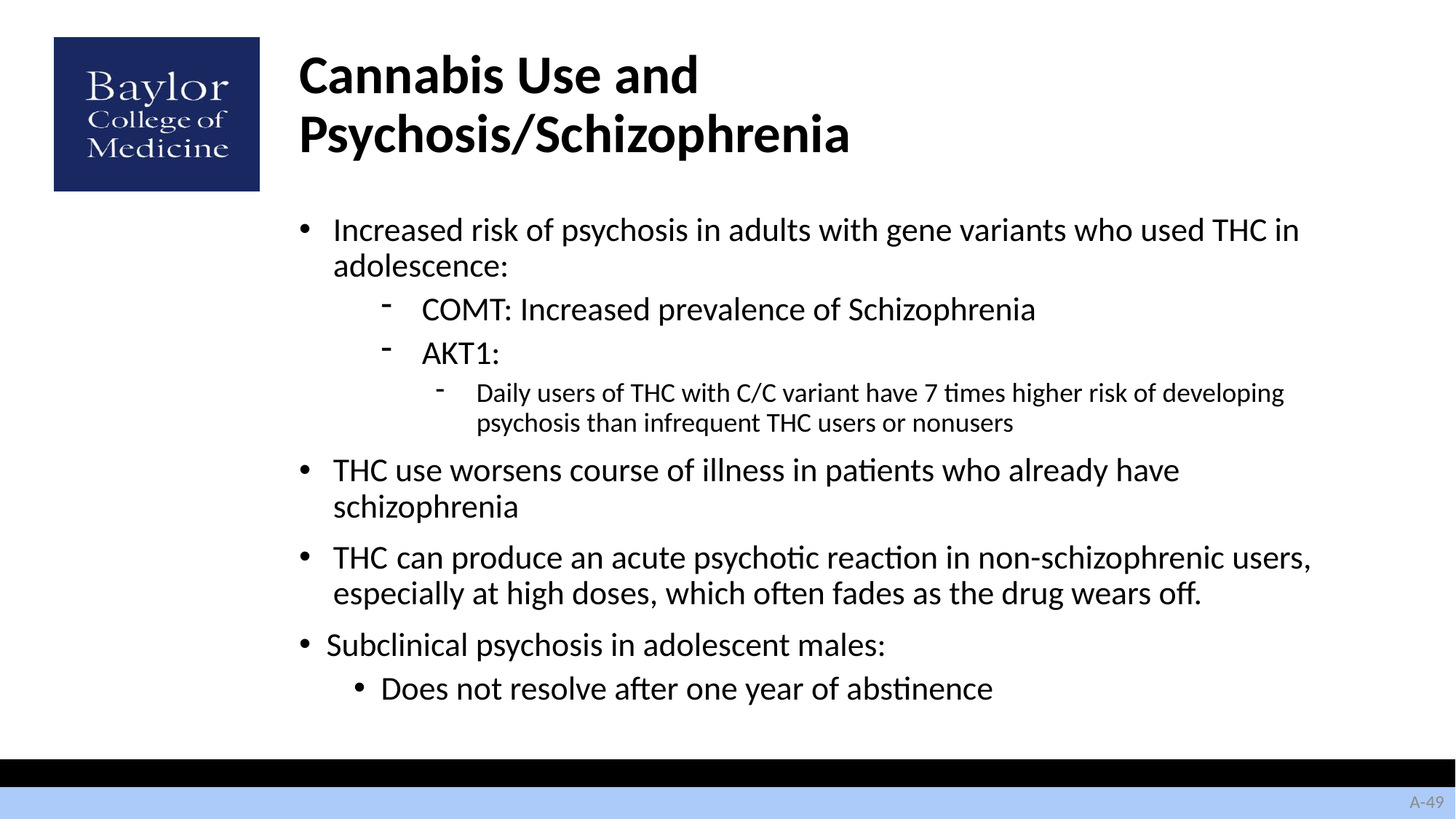

Cannabis Use and Psychosis/Schizophrenia
Increased risk of psychosis in adults with gene variants who used THC in adolescence:
COMT: Increased prevalence of Schizophrenia
AKT1:
Daily users of THC with C/C variant have 7 times higher risk of developing psychosis than infrequent THC users or nonusers
THC use worsens course of illness in patients who already have schizophrenia
THC can produce an acute psychotic reaction in non-schizophrenic users, especially at high doses, which often fades as the drug wears off.
Subclinical psychosis in adolescent males:
Does not resolve after one year of abstinence
A-49

## Slide 50
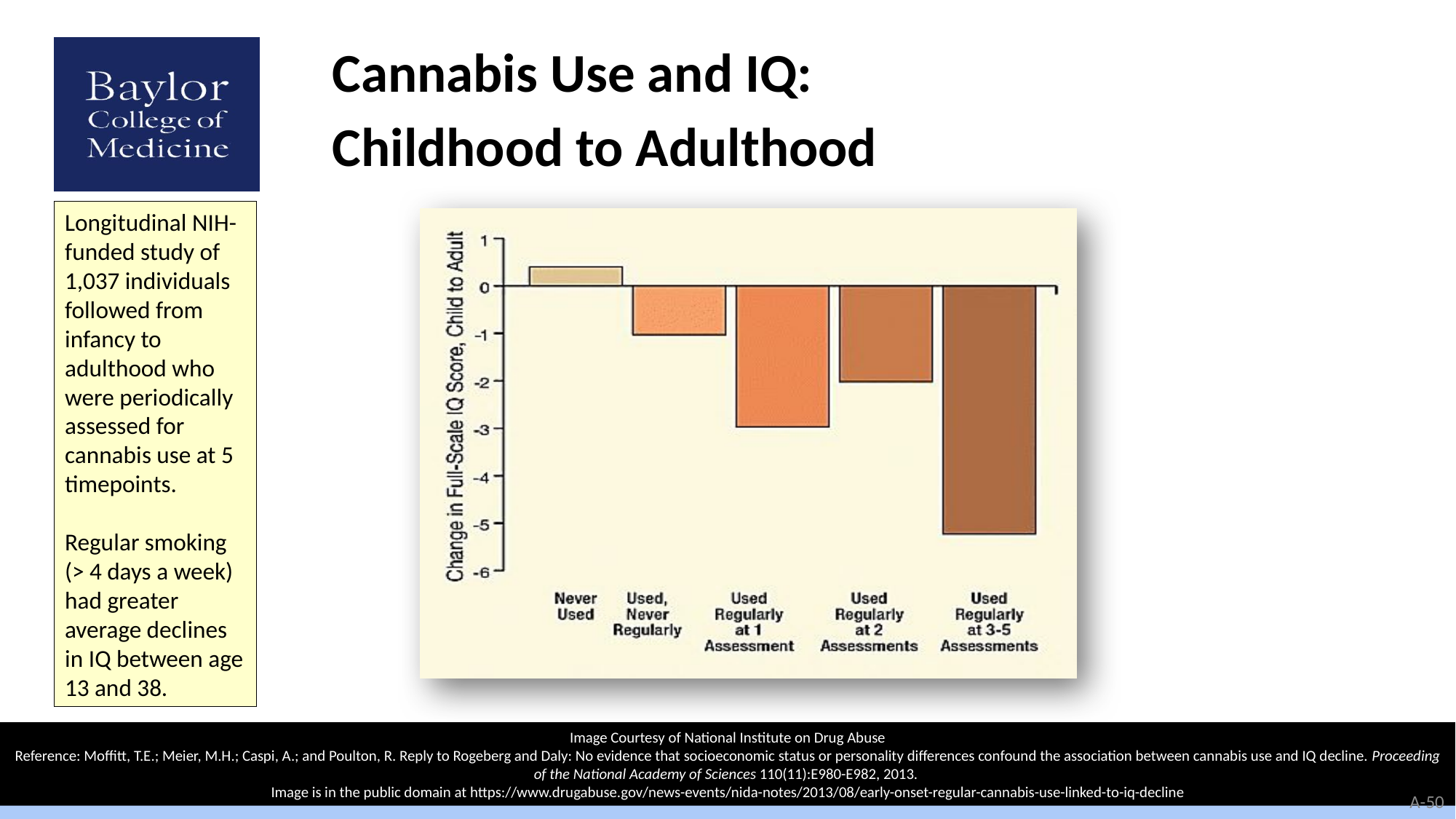

Cannabis Use and IQ:
Childhood to Adulthood
Longitudinal NIH-funded study of 1,037 individuals followed from infancy to adulthood who were periodically assessed for cannabis use at 5 timepoints.
Regular smoking
(> 4 days a week) had greater average declines in IQ between age 13 and 38.
Image Courtesy of National Institute on Drug Abuse
Reference: Moffitt, T.E.; Meier, M.H.; Caspi, A.; and Poulton, R. Reply to Rogeberg and Daly: No evidence that socioeconomic status or personality differences confound the association between cannabis use and IQ decline. Proceeding of the National Academy of Sciences 110(11):E980-E982, 2013.
Image is in the public domain at https://www.drugabuse.gov/news-events/nida-notes/2013/08/early-onset-regular-cannabis-use-linked-to-iq-decline
A-50

## Slide 51
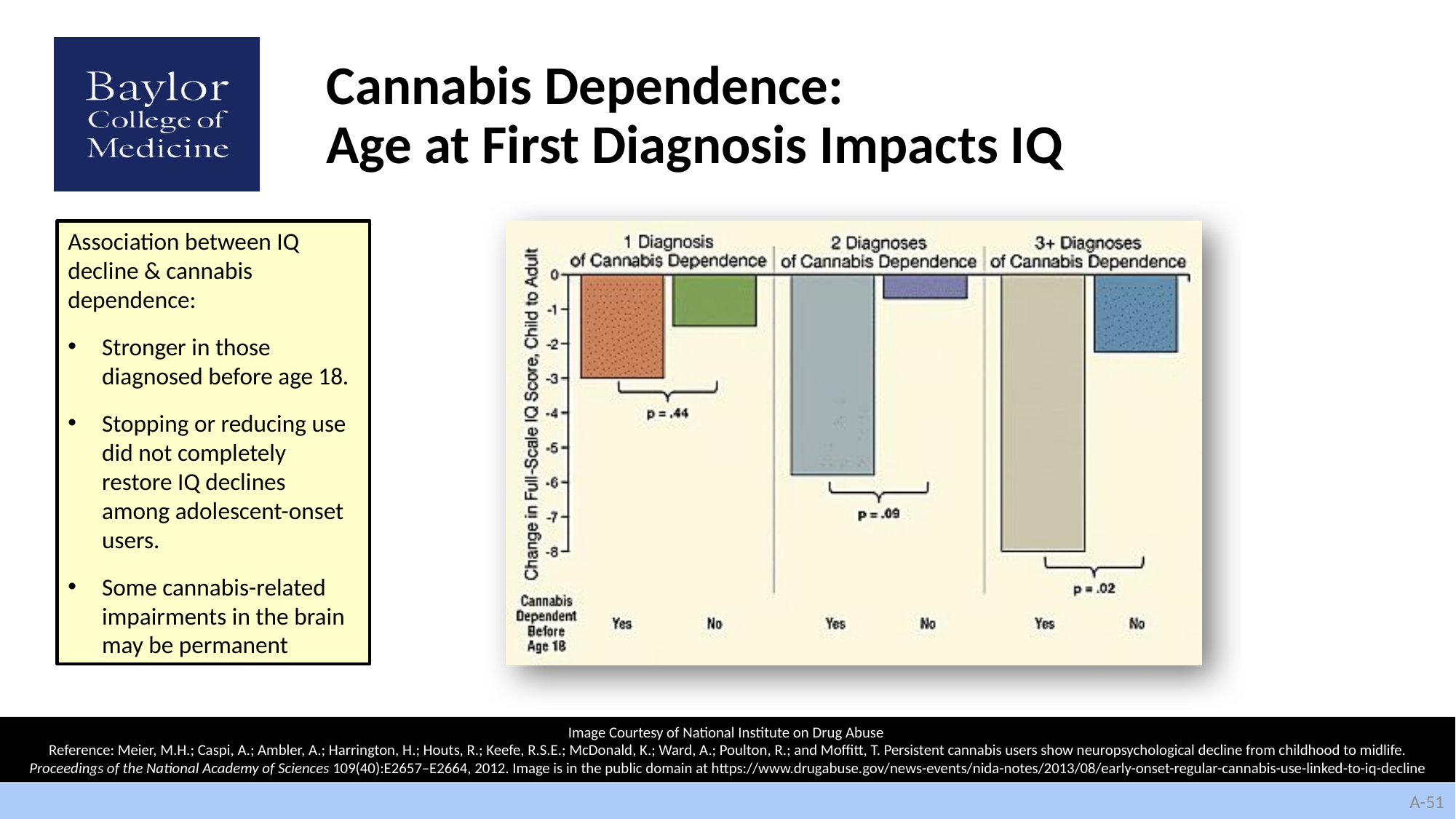

Cannabis Dependence:
Age at First Diagnosis Impacts IQ
Association between IQ decline & cannabis dependence:
Stronger in those diagnosed before age 18.
Stopping or reducing use did not completely restore IQ declines among adolescent-onset users.
Some cannabis-related impairments in the brain may be permanent
Image Courtesy of National Institute on Drug Abuse
Reference: Meier, M.H.; Caspi, A.; Ambler, A.; Harrington, H.; Houts, R.; Keefe, R.S.E.; McDonald, K.; Ward, A.; Poulton, R.; and Moffitt, T. Persistent cannabis users show neuropsychological decline from childhood to midlife. Proceedings of the National Academy of Sciences 109(40):E2657–E2664, 2012. Image is in the public domain at https://www.drugabuse.gov/news-events/nida-notes/2013/08/early-onset-regular-cannabis-use-linked-to-iq-decline
A-51

## Slide 52
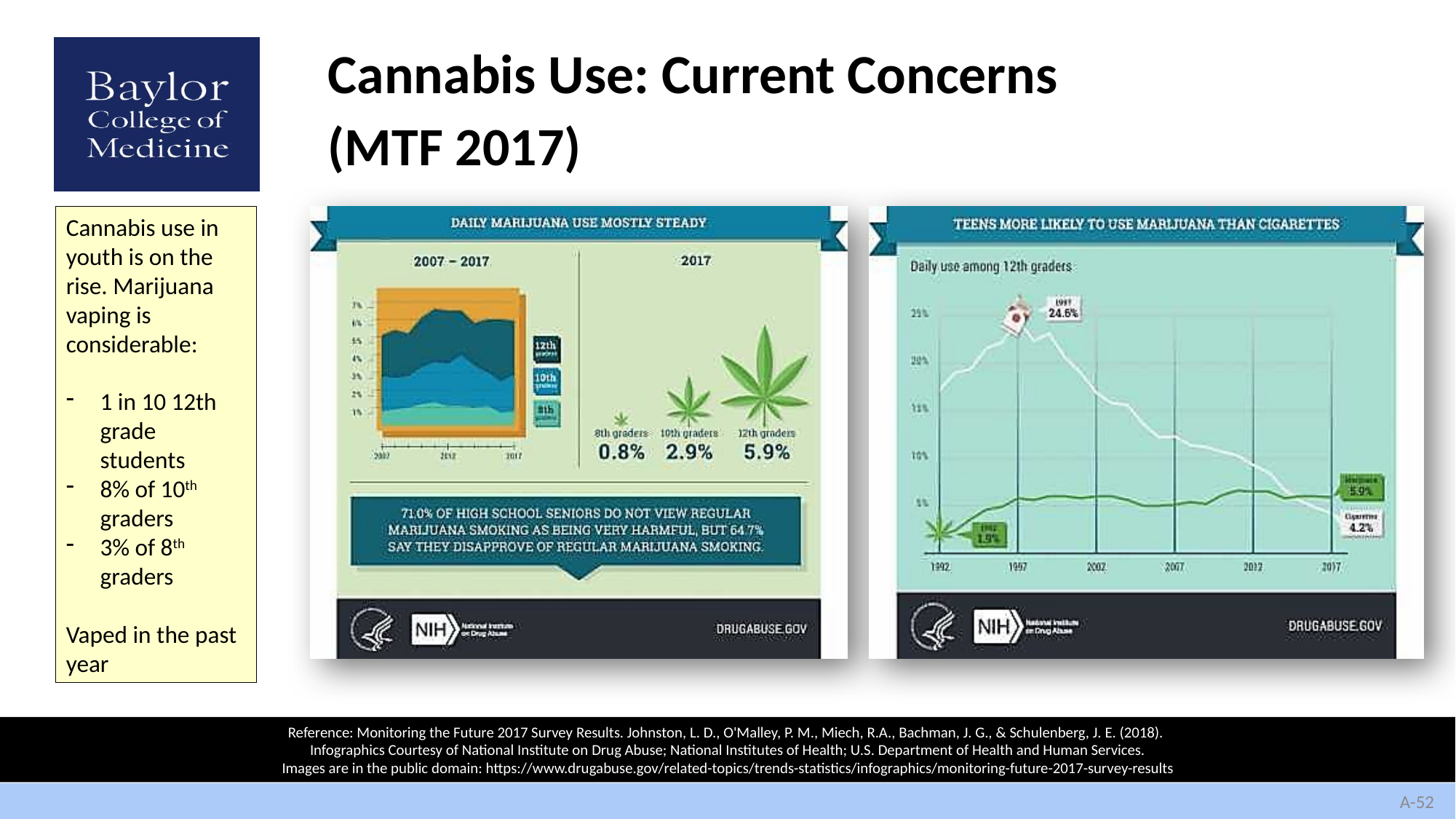

Cannabis Use: Current Concerns
(MTF 2017)
Cannabis use in youth is on the rise. Marijuana vaping is considerable:
1 in 10 12th grade students
8% of 10th graders
3% of 8th graders
Vaped in the past year
Reference: Monitoring the Future 2017 Survey Results. Johnston, L. D., O'Malley, P. M., Miech, R.A., Bachman, J. G., & Schulenberg, J. E. (2018).
Infographics Courtesy of National Institute on Drug Abuse; National Institutes of Health; U.S. Department of Health and Human Services.
Images are in the public domain: https://www.drugabuse.gov/related-topics/trends-statistics/infographics/monitoring-future-2017-survey-results
A-52

## Slide 53
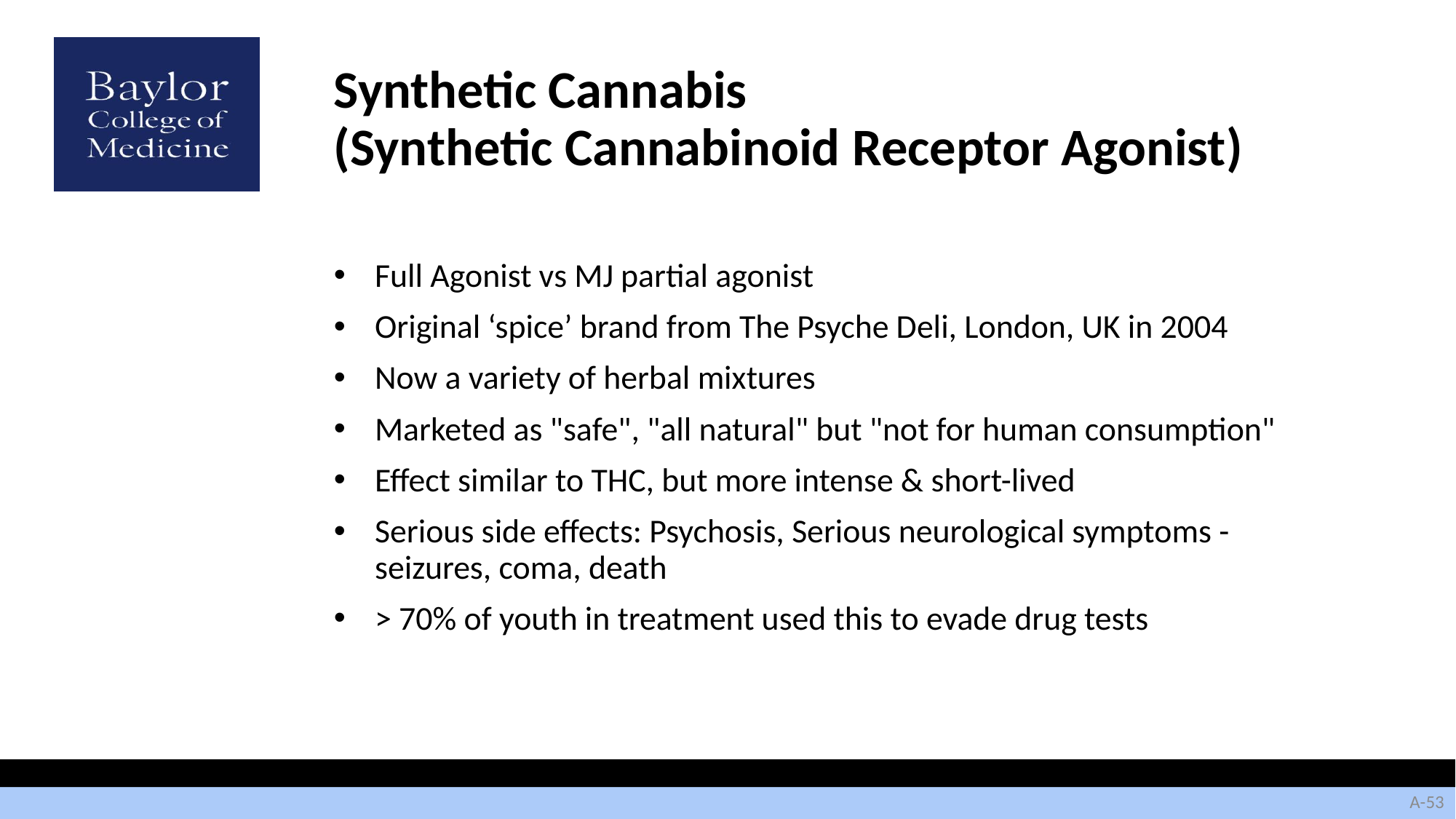

Synthetic Cannabis
(Synthetic Cannabinoid Receptor Agonist)
Full Agonist vs MJ partial agonist
Original ‘spice’ brand from The Psyche Deli, London, UK in 2004
Now a variety of herbal mixtures
Marketed as "safe", "all natural" but "not for human consumption"
Effect similar to THC, but more intense & short-lived
Serious side effects: Psychosis, Serious neurological symptoms - seizures, coma, death
> 70% of youth in treatment used this to evade drug tests
A-53

## Slide 54
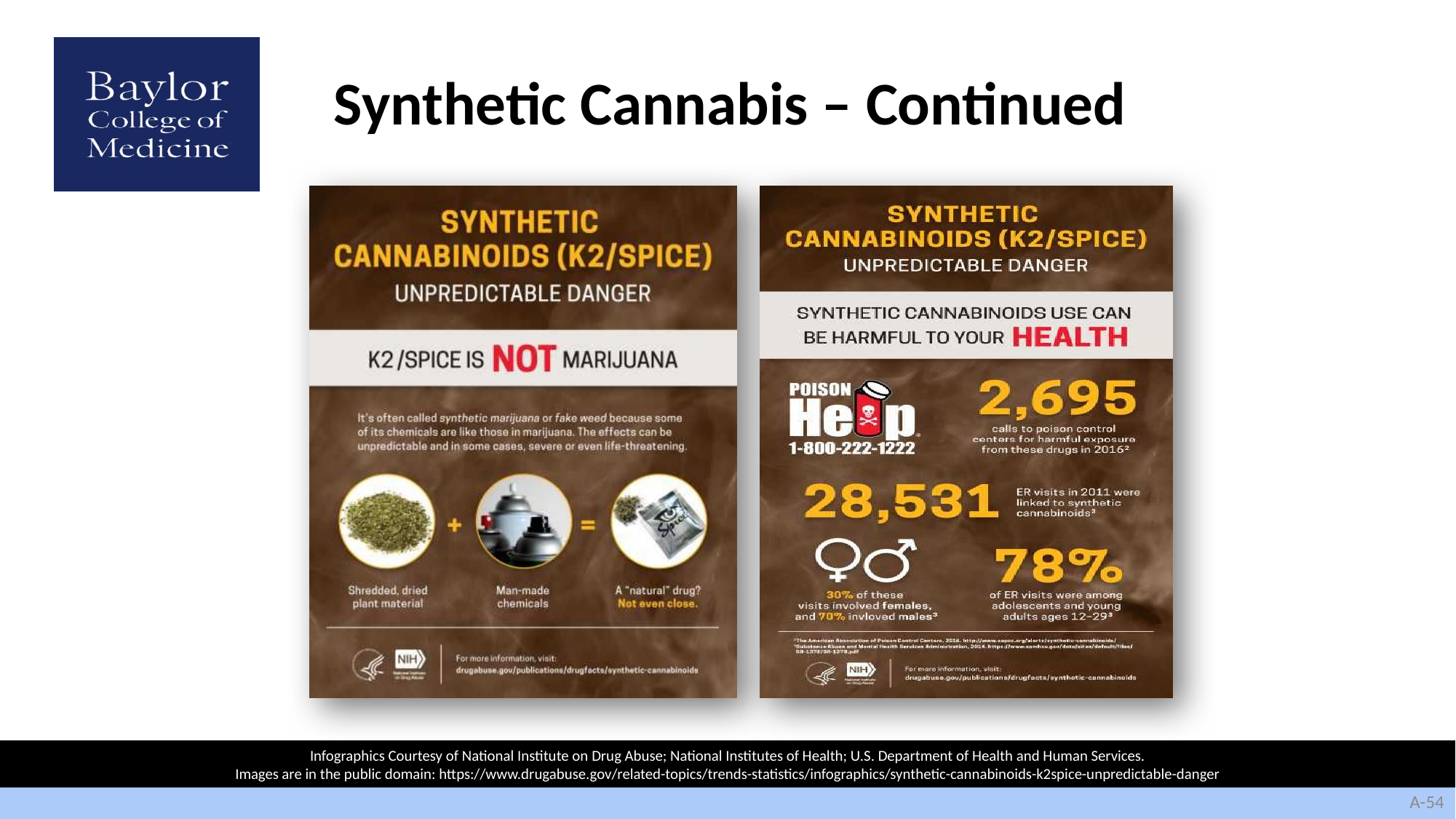

Synthetic Cannabis – Continued
Infographics Courtesy of National Institute on Drug Abuse; National Institutes of Health; U.S. Department of Health and Human Services.
Images are in the public domain: https://www.drugabuse.gov/related-topics/trends-statistics/infographics/synthetic-cannabinoids-k2spice-unpredictable-danger
A-54

## Slide 55
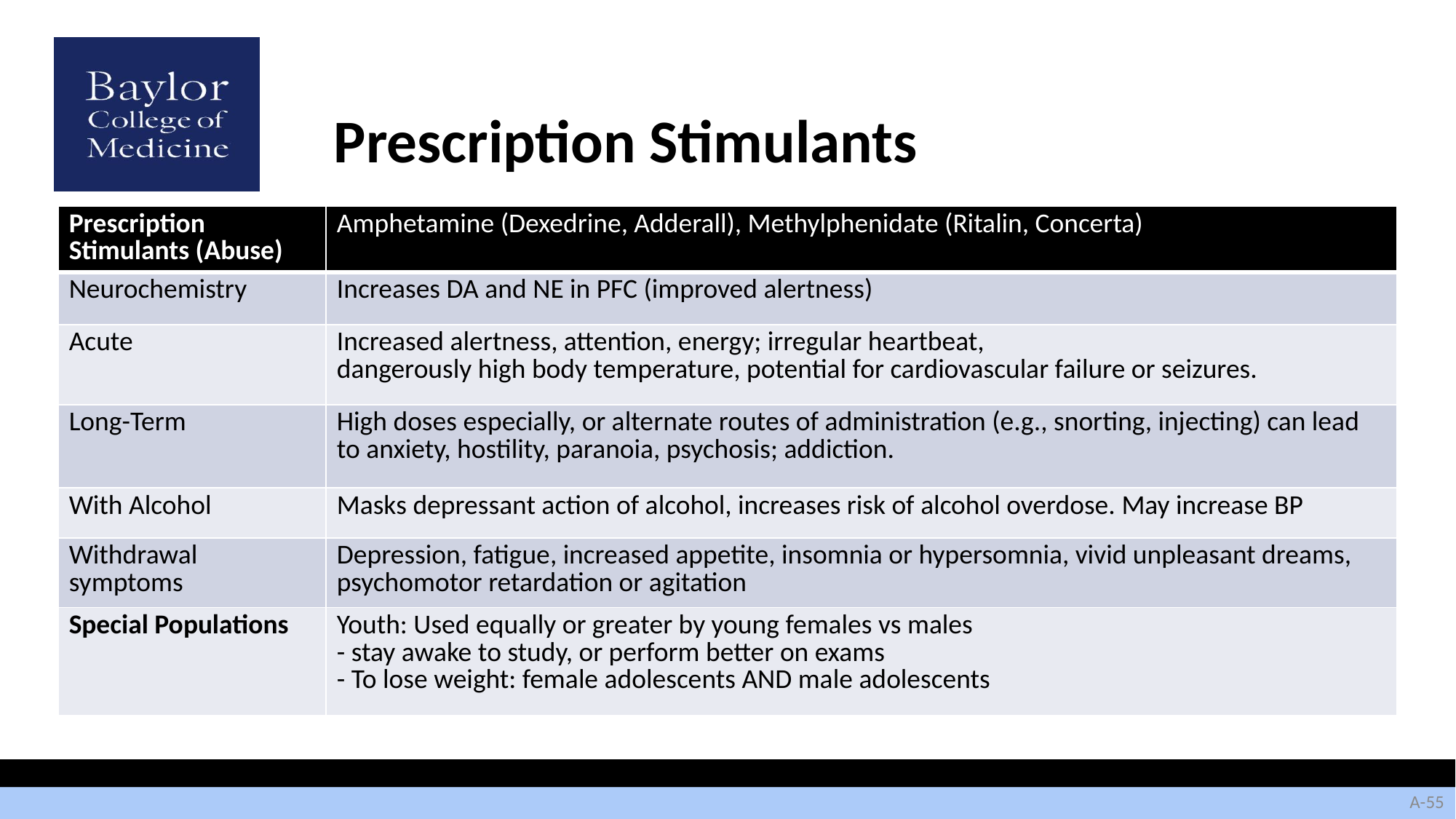

Prescription Stimulants
| Prescription Stimulants (Abuse) | Amphetamine (Dexedrine, Adderall), Methylphenidate (Ritalin, Concerta) |
| --- | --- |
| Neurochemistry | Increases DA and NE in PFC (improved alertness) |
| Acute | Increased alertness, attention, energy; irregular heartbeat, dangerously high body temperature, potential for cardiovascular failure or seizures. |
| Long-Term | High doses especially, or alternate routes of administration (e.g., snorting, injecting) can lead to anxiety, hostility, paranoia, psychosis; addiction. |
| With Alcohol | Masks depressant action of alcohol, increases risk of alcohol overdose. May increase BP |
| Withdrawal symptoms | Depression, fatigue, increased appetite, insomnia or hypersomnia, vivid unpleasant dreams, psychomotor retardation or agitation |
| Special Populations | Youth: Used equally or greater by young females vs males - stay awake to study, or perform better on exams - To lose weight: female adolescents AND male adolescents |
A-55

## Slide 56
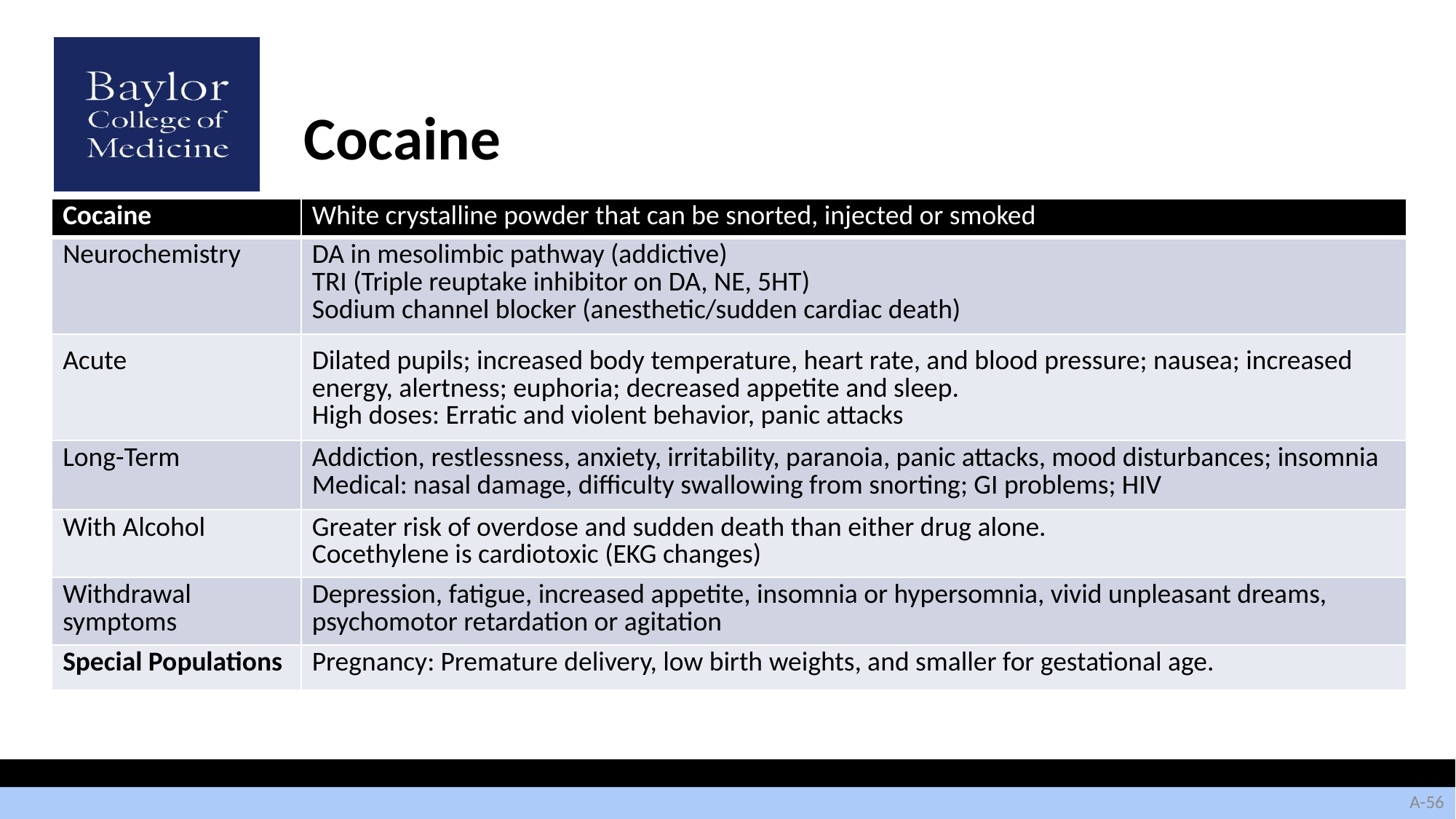

Cocaine
| Cocaine | White crystalline powder that can be snorted, injected or smoked |
| --- | --- |
| Neurochemistry | DA in mesolimbic pathway (addictive) TRI (Triple reuptake inhibitor on DA, NE, 5HT) Sodium channel blocker (anesthetic/sudden cardiac death) |
| Acute | Dilated pupils; increased body temperature, heart rate, and blood pressure; nausea; increased energy, alertness; euphoria; decreased appetite and sleep. High doses: Erratic and violent behavior, panic attacks |
| Long-Term | Addiction, restlessness, anxiety, irritability, paranoia, panic attacks, mood disturbances; insomnia Medical: nasal damage, difficulty swallowing from snorting; GI problems; HIV |
| With Alcohol | Greater risk of overdose and sudden death than either drug alone. Cocethylene is cardiotoxic (EKG changes) |
| Withdrawal symptoms | Depression, fatigue, increased appetite, insomnia or hypersomnia, vivid unpleasant dreams, psychomotor retardation or agitation |
| Special Populations | Pregnancy: Premature delivery, low birth weights, and smaller for gestational age. |
A-56

## Slide 57
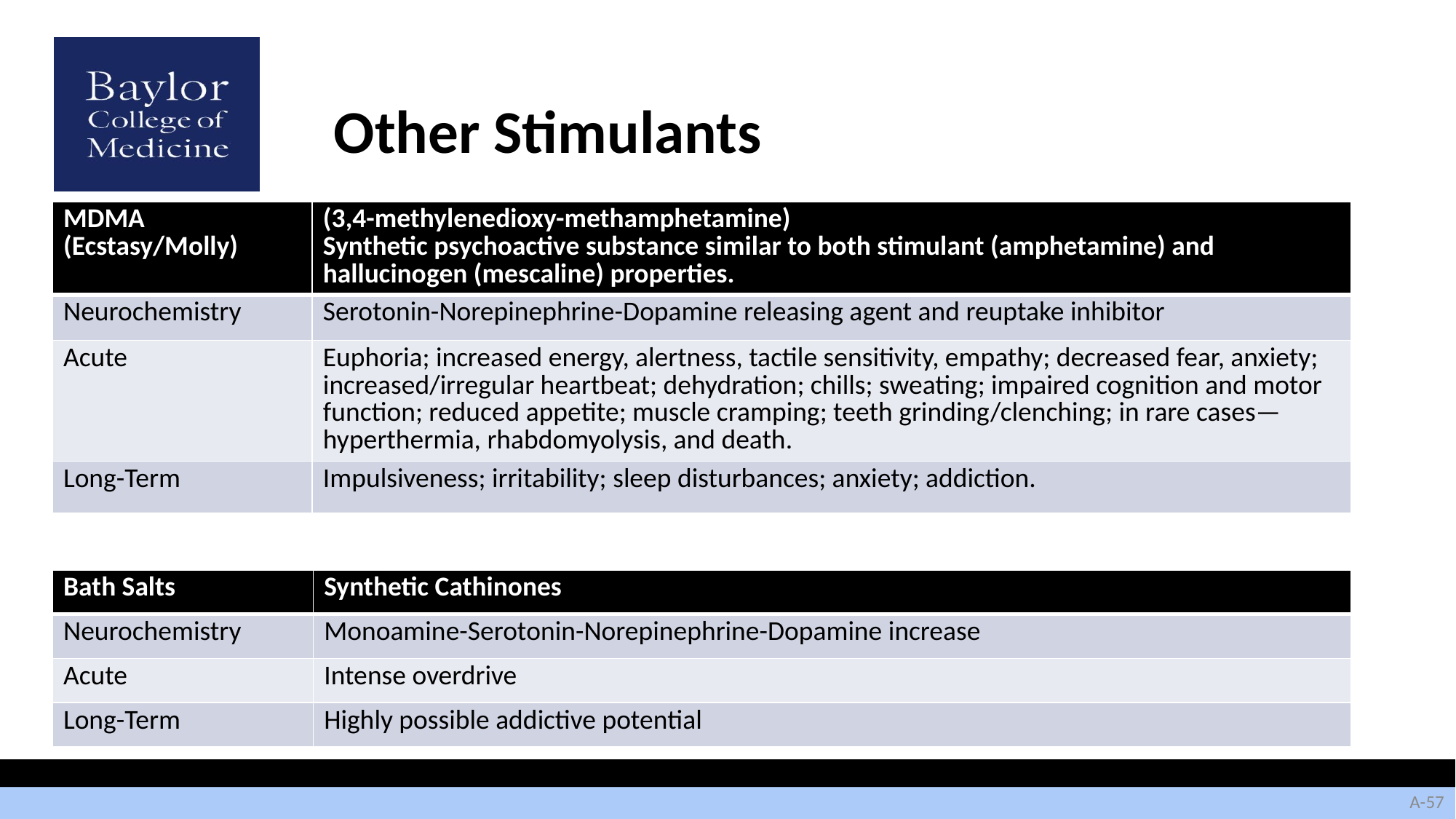

Other Stimulants
| MDMA (Ecstasy/Molly) | (3,4-methylenedioxy-methamphetamine) Synthetic psychoactive substance similar to both stimulant (amphetamine) and hallucinogen (mescaline) properties. |
| --- | --- |
| Neurochemistry | Serotonin-Norepinephrine-Dopamine releasing agent and reuptake inhibitor |
| Acute | Euphoria; increased energy, alertness, tactile sensitivity, empathy; decreased fear, anxiety; increased/irregular heartbeat; dehydration; chills; sweating; impaired cognition and motor function; reduced appetite; muscle cramping; teeth grinding/clenching; in rare cases—hyperthermia, rhabdomyolysis, and death. |
| Long-Term | Impulsiveness; irritability; sleep disturbances; anxiety; addiction. |
| Bath Salts | Synthetic Cathinones |
| --- | --- |
| Neurochemistry | Monoamine-Serotonin-Norepinephrine-Dopamine increase |
| Acute | Intense overdrive |
| Long-Term | Highly possible addictive potential |
A-57

## Slide 58
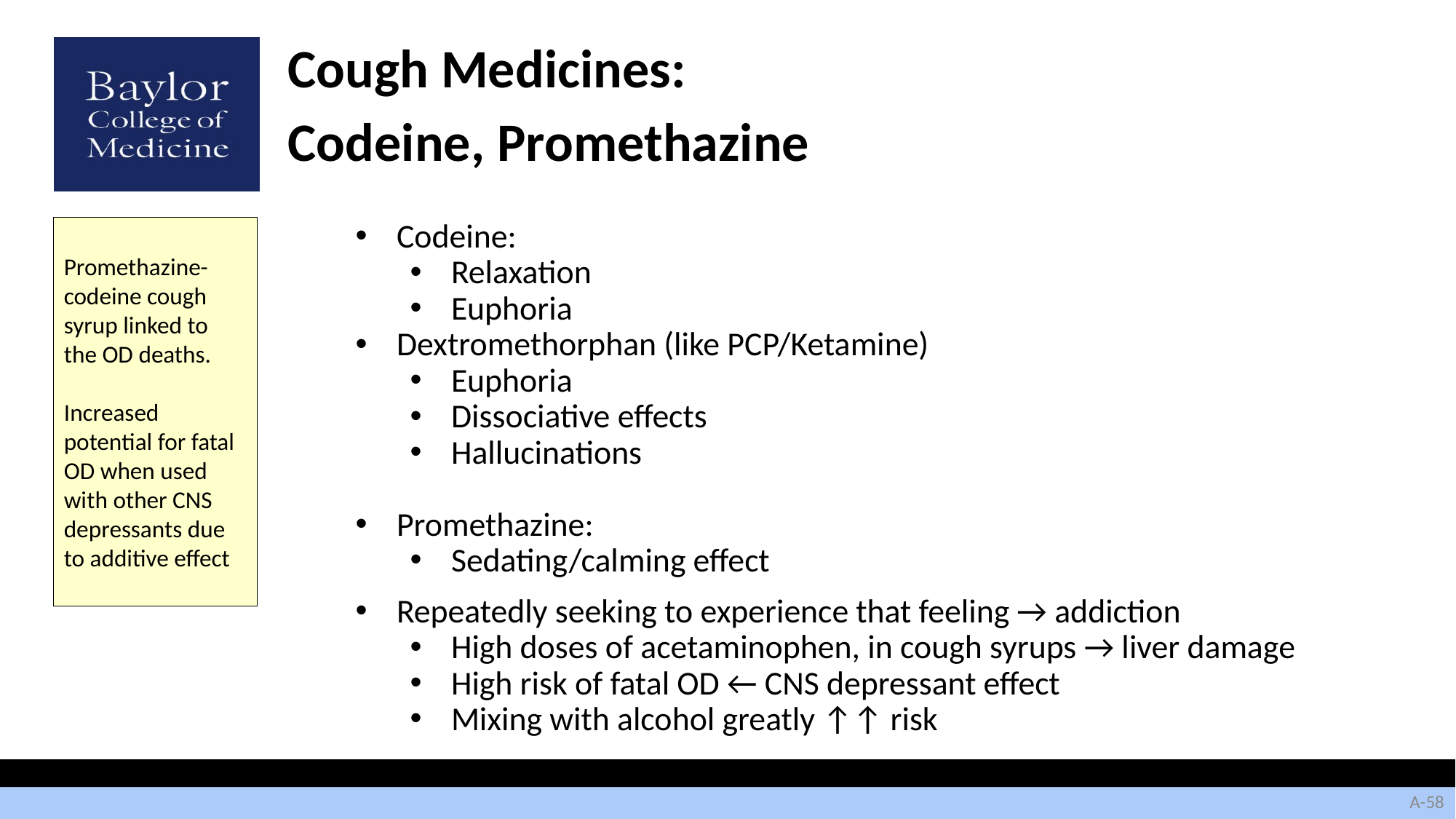

Cough Medicines:
Codeine, Promethazine
Codeine:
Relaxation
Euphoria
Dextromethorphan (like PCP/Ketamine)
Euphoria
Dissociative effects
Hallucinations
Promethazine:
Sedating/calming effect
Repeatedly seeking to experience that feeling → addiction
High doses of acetaminophen, in cough syrups → liver damage
High risk of fatal OD ← CNS depressant effect
Mixing with alcohol greatly ↑↑ risk
Promethazine-codeine cough syrup linked to the OD deaths.
Increased potential for fatal OD when used with other CNS depressants due to additive effect
A-58

## Slide 59
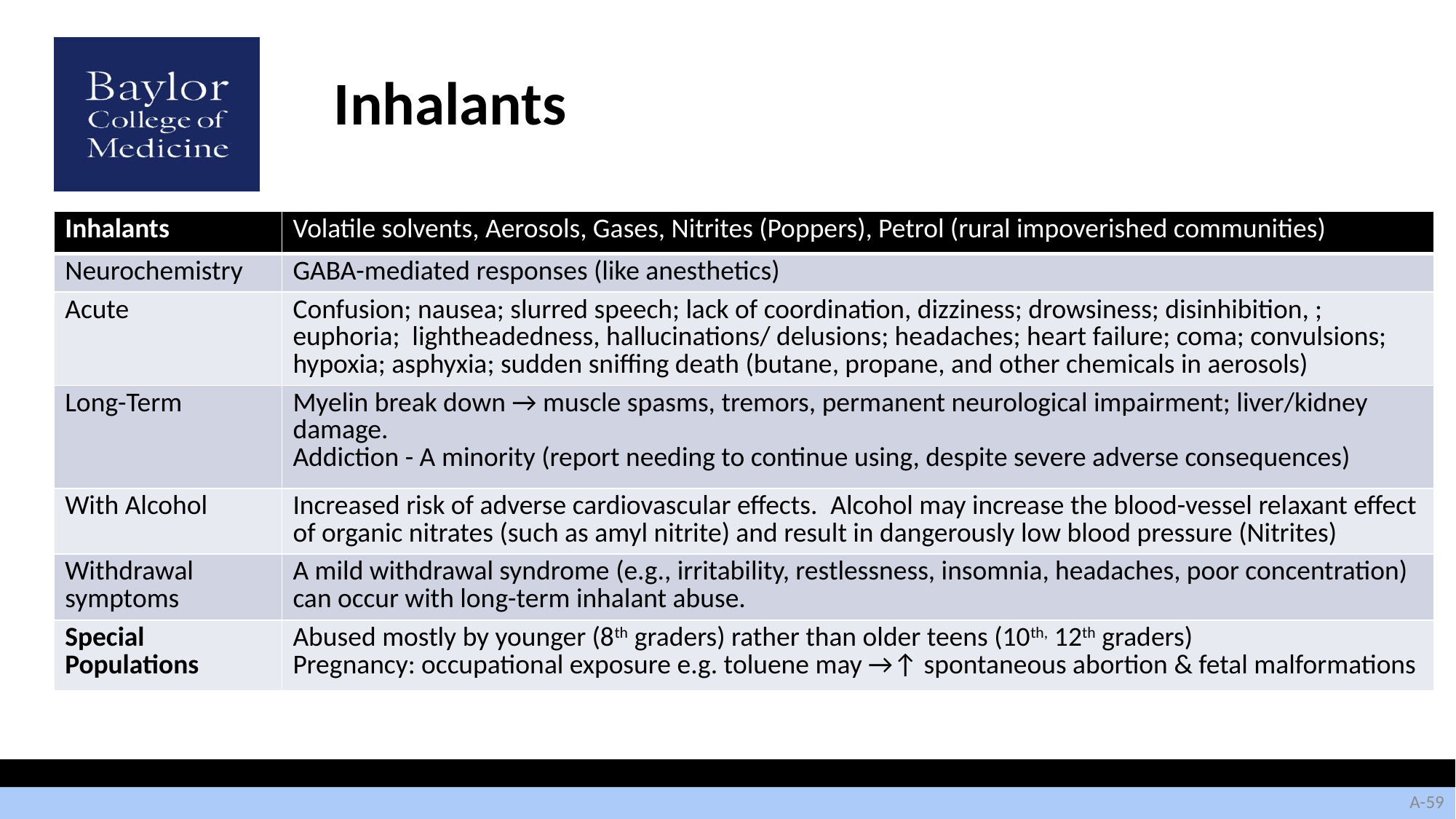

Inhalants
| Inhalants | Volatile solvents, Aerosols, Gases, Nitrites (Poppers), Petrol (rural impoverished communities) |
| --- | --- |
| Neurochemistry | GABA-mediated responses (like anesthetics) |
| Acute | Confusion; nausea; slurred speech; lack of coordination, dizziness; drowsiness; disinhibition, ; euphoria; lightheadedness, hallucinations/ delusions; headaches; heart failure; coma; convulsions; hypoxia; asphyxia; sudden sniffing death (butane, propane, and other chemicals in aerosols) |
| Long-Term | Myelin break down → muscle spasms, tremors, permanent neurological impairment; liver/kidney damage.  Addiction - A minority (report needing to continue using, despite severe adverse consequences) |
| With Alcohol | Increased risk of adverse cardiovascular effects.  Alcohol may increase the blood-vessel relaxant effect of organic nitrates (such as amyl nitrite) and result in dangerously low blood pressure (Nitrites) |
| Withdrawal symptoms | A mild withdrawal syndrome (e.g., irritability, restlessness, insomnia, headaches, poor concentration) can occur with long-term inhalant abuse. |
| Special Populations | Abused mostly by younger (8th graders) rather than older teens (10th, 12th graders) Pregnancy: occupational exposure e.g. toluene may →↑ spontaneous abortion & fetal malformations |
A-59

## Slide 60
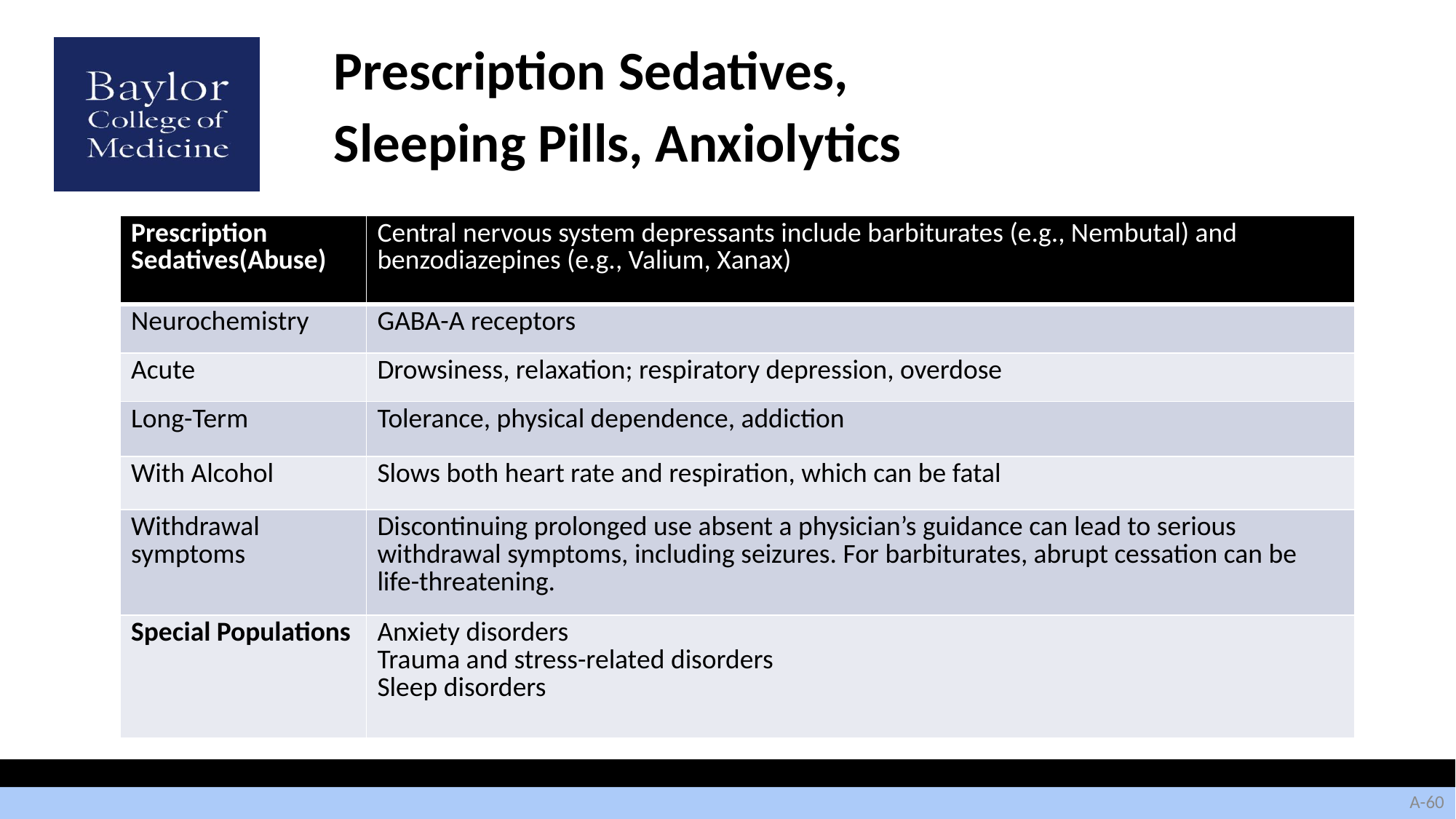

Prescription Sedatives,
Sleeping Pills, Anxiolytics
| Prescription Sedatives(Abuse) | Central nervous system depressants include barbiturates (e.g., Nembutal) and benzodiazepines (e.g., Valium, Xanax) |
| --- | --- |
| Neurochemistry | GABA-A receptors |
| Acute | Drowsiness, relaxation; respiratory depression, overdose |
| Long-Term | Tolerance, physical dependence, addiction |
| With Alcohol | Slows both heart rate and respiration, which can be fatal |
| Withdrawal symptoms | Discontinuing prolonged use absent a physician’s guidance can lead to serious withdrawal symptoms, including seizures. For barbiturates, abrupt cessation can be life-threatening. |
| Special Populations | Anxiety disorders Trauma and stress-related disorders Sleep disorders |
A-60

## Slide 61
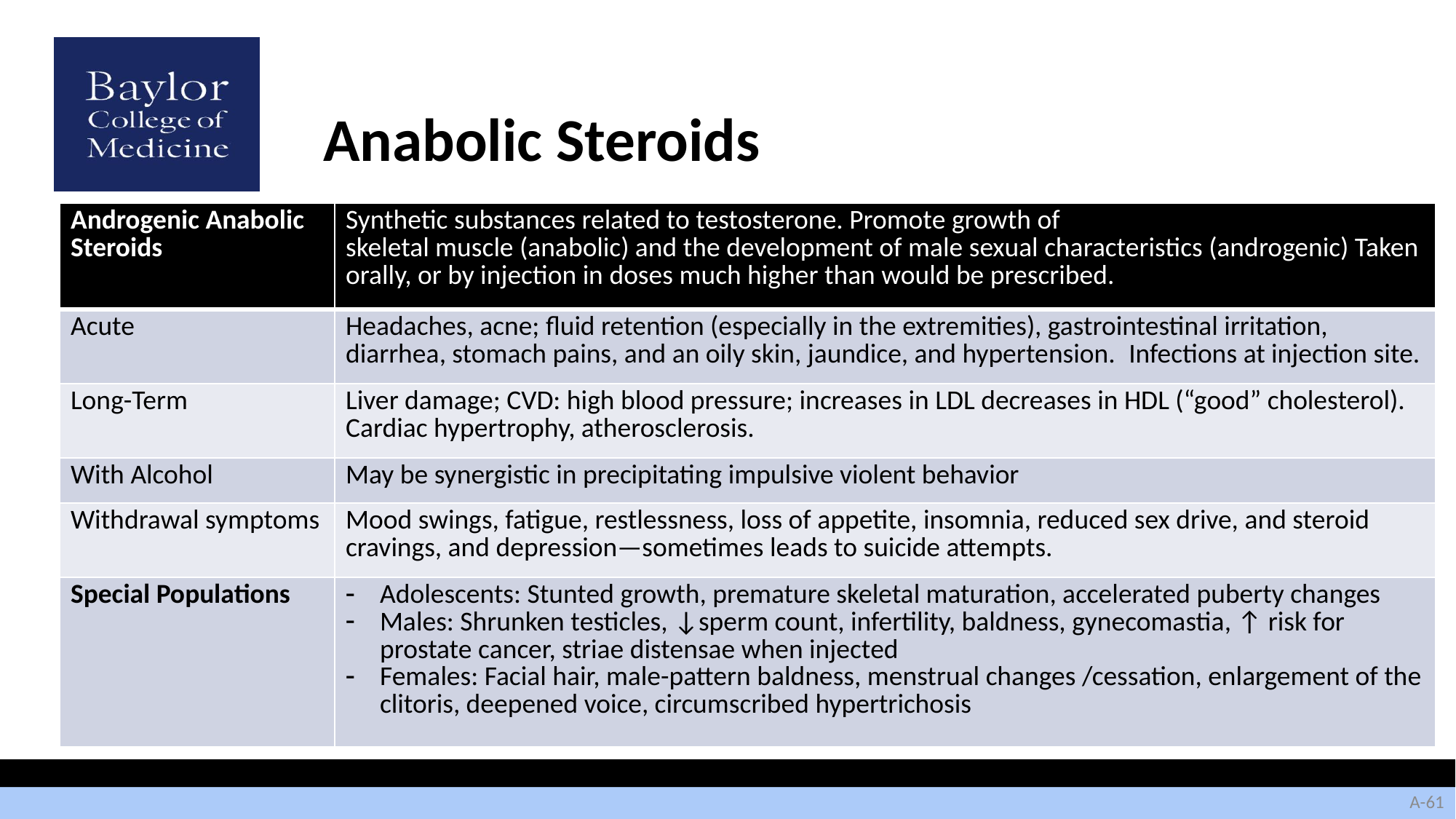

Anabolic Steroids
| Androgenic Anabolic Steroids | Synthetic substances related to testosterone. Promote growth of skeletal muscle (anabolic) and the development of male sexual characteristics (androgenic) Taken orally, or by injection in doses much higher than would be prescribed. |
| --- | --- |
| Acute | Headaches, acne; fluid retention (especially in the extremities), gastrointestinal irritation, diarrhea, stomach pains, and an oily skin, jaundice, and hypertension.  Infections at injection site. |
| Long-Term | Liver damage; CVD: high blood pressure; increases in LDL decreases in HDL (“good” cholesterol). Cardiac hypertrophy, atherosclerosis. |
| With Alcohol | May be synergistic in precipitating impulsive violent behavior |
| Withdrawal symptoms | Mood swings, fatigue, restlessness, loss of appetite, insomnia, reduced sex drive, and steroid cravings, and depression—sometimes leads to suicide attempts. |
| Special Populations | Adolescents: Stunted growth, premature skeletal maturation, accelerated puberty changes Males: Shrunken testicles, ↓sperm count, infertility, baldness, gynecomastia, ↑ risk for prostate cancer, striae distensae when injected Females: Facial hair, male-pattern baldness, menstrual changes /cessation, enlargement of the clitoris, deepened voice, circumscribed hypertrichosis |
A-61

## Slide 62
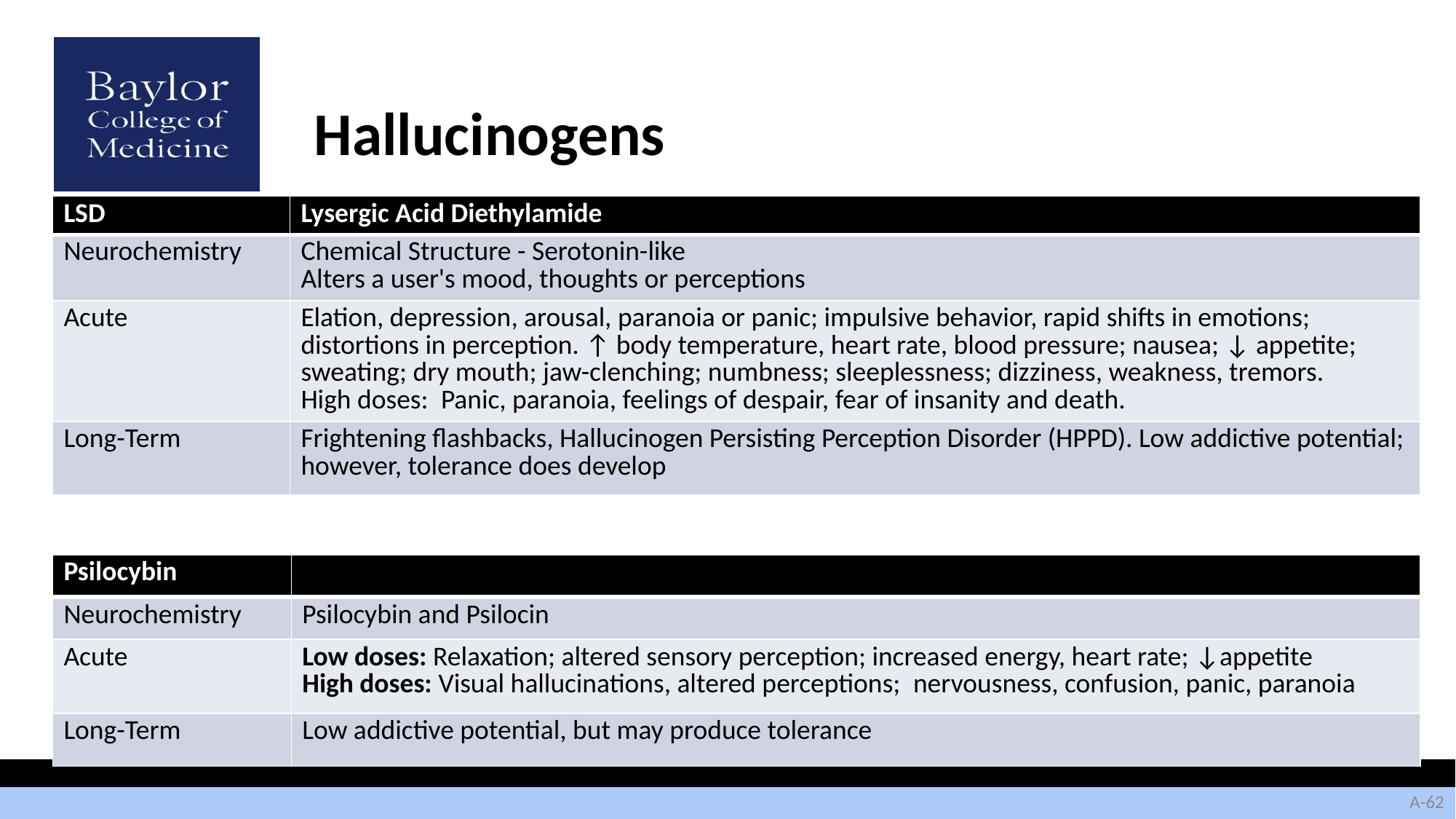

Hallucinogens
| LSD | Lysergic Acid Diethylamide |
| --- | --- |
| Neurochemistry | Chemical Structure - Serotonin-like Alters a user's mood, thoughts or perceptions |
| Acute | Elation, depression, arousal, paranoia or panic; impulsive behavior, rapid shifts in emotions; distortions in perception. ↑ body temperature, heart rate, blood pressure; nausea; ↓ appetite; sweating; dry mouth; jaw-clenching; numbness; sleeplessness; dizziness, weakness, tremors. High doses:  Panic, paranoia, feelings of despair, fear of insanity and death. |
| Long-Term | Frightening flashbacks, Hallucinogen Persisting Perception Disorder (HPPD). Low addictive potential; however, tolerance does develop |
| Psilocybin | |
| --- | --- |
| Neurochemistry | Psilocybin and Psilocin |
| Acute | Low doses: Relaxation; altered sensory perception; increased energy, heart rate; ↓appetite High doses: Visual hallucinations, altered perceptions;  nervousness, confusion, panic, paranoia |
| Long-Term | Low addictive potential, but may produce tolerance |
A-62

## Slide 63
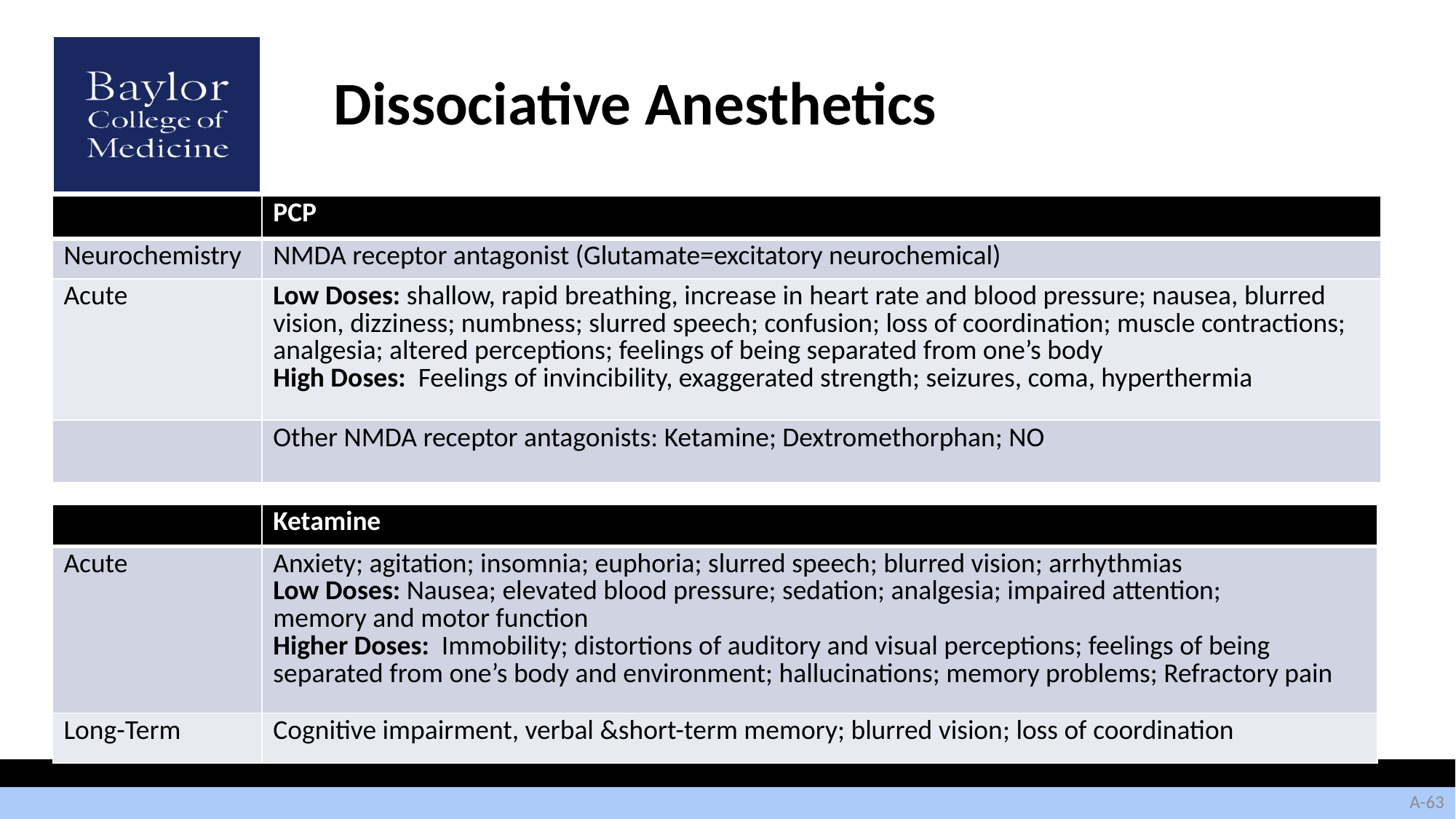

Dissociative Anesthetics
| | PCP |
| --- | --- |
| Neurochemistry | NMDA receptor antagonist (Glutamate=excitatory neurochemical) |
| Acute | Low Doses: shallow, rapid breathing, increase in heart rate and blood pressure; nausea, blurred vision, dizziness; numbness; slurred speech; confusion; loss of coordination; muscle contractions; analgesia; altered perceptions; feelings of being separated from one’s body High Doses:  Feelings of invincibility, exaggerated strength; seizures, coma, hyperthermia |
| | Other NMDA receptor antagonists: Ketamine; Dextromethorphan; NO |
| | Ketamine |
| --- | --- |
| Acute | Anxiety; agitation; insomnia; euphoria; slurred speech; blurred vision; arrhythmias Low Doses: Nausea; elevated blood pressure; sedation; analgesia; impaired attention; memory and motor function Higher Doses:  Immobility; distortions of auditory and visual perceptions; feelings of being separated from one’s body and environment; hallucinations; memory problems; Refractory pain |
| Long-Term | Cognitive impairment, verbal &short-term memory; blurred vision; loss of coordination |
A-63

## Slide 64
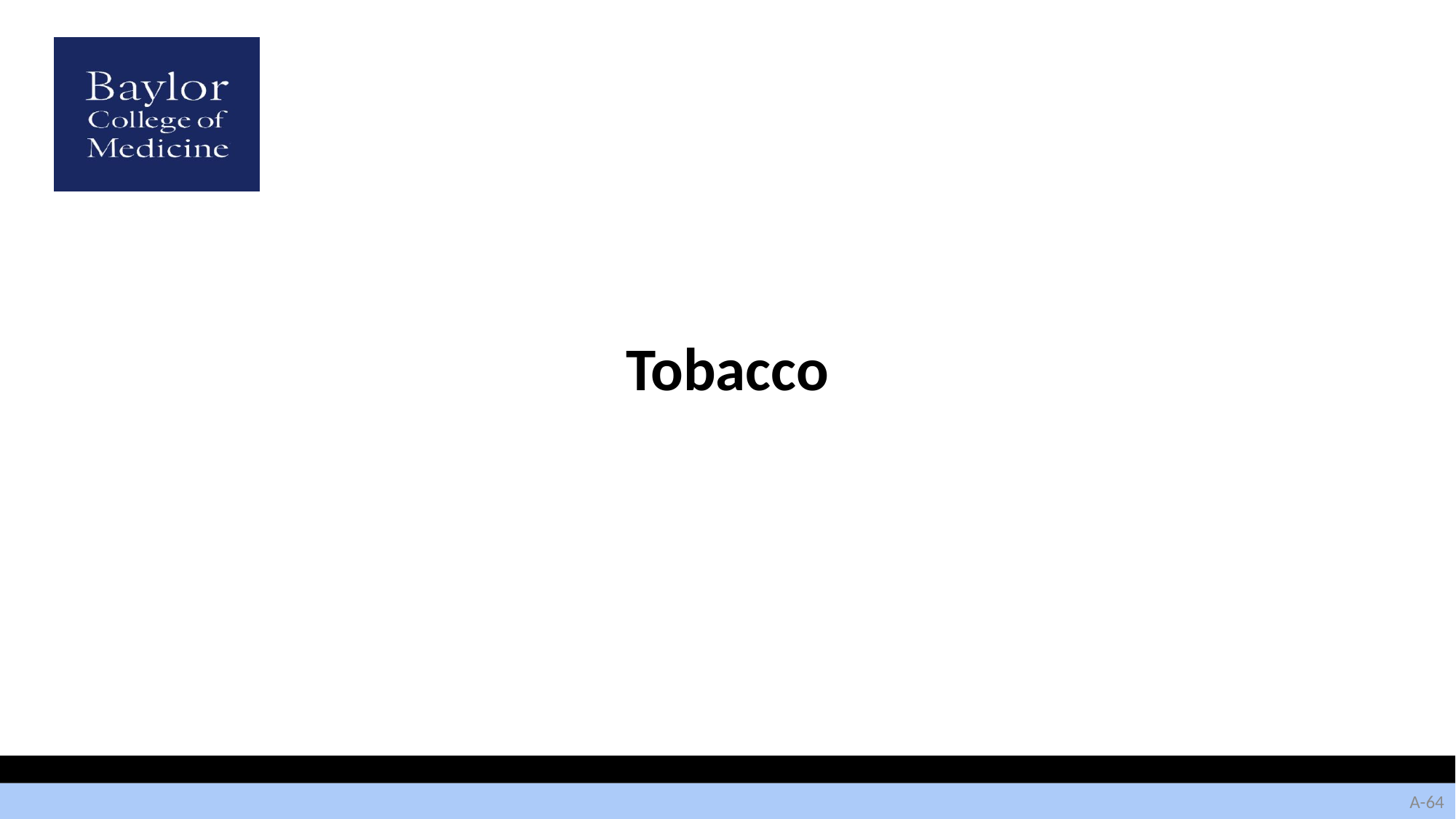

Tobacco
A-64

## Slide 65
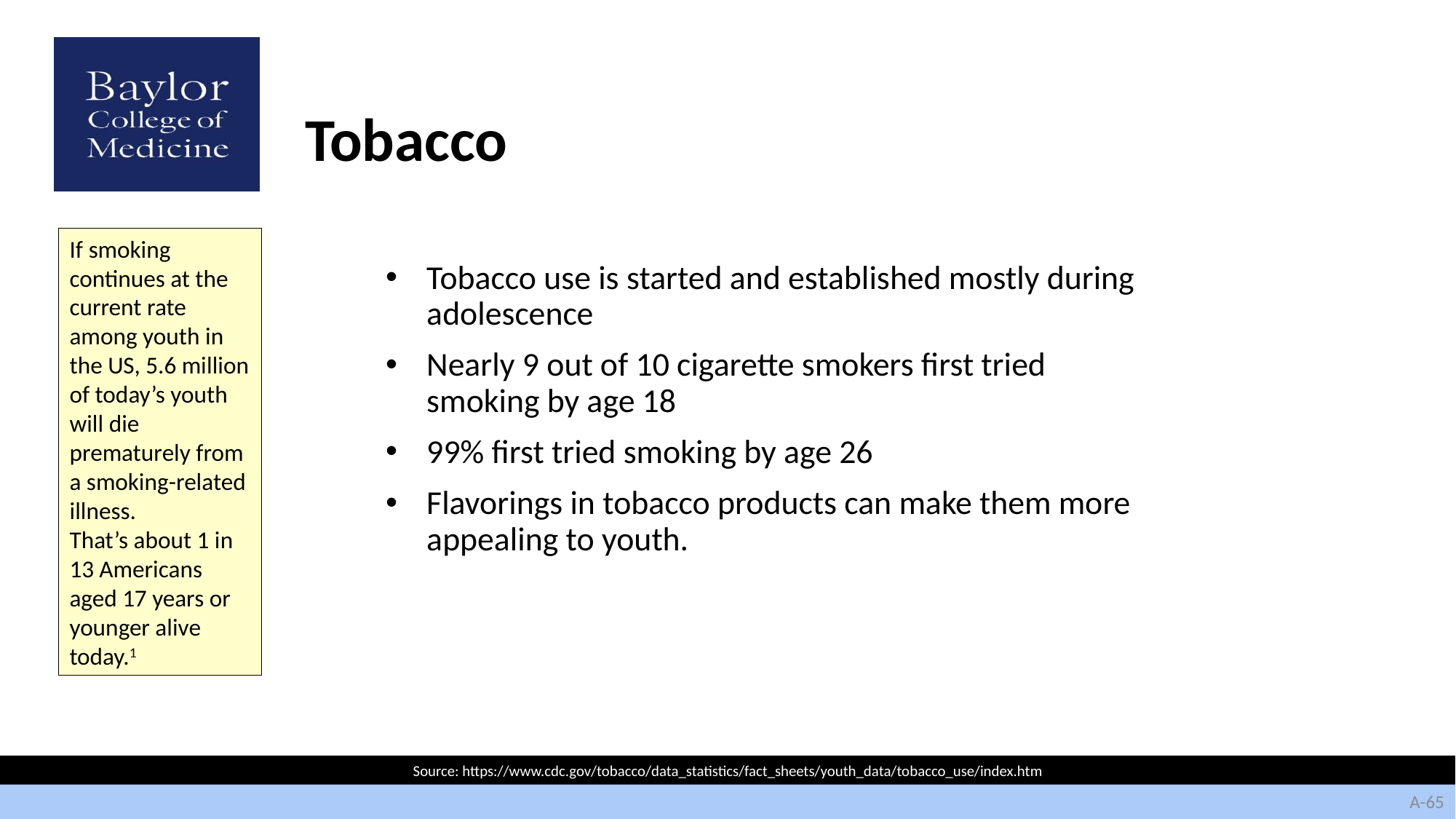

Tobacco
If smoking continues at the current rate among youth in the US, 5.6 million of today’s youth will die prematurely from a smoking-related illness.
That’s about 1 in 13 Americans aged 17 years or younger alive today.1
Tobacco use is started and established mostly during adolescence
Nearly 9 out of 10 cigarette smokers first tried smoking by age 18
99% first tried smoking by age 26
Flavorings in tobacco products can make them more appealing to youth.
Source: https://www.cdc.gov/tobacco/data_statistics/fact_sheets/youth_data/tobacco_use/index.htm
A-65

## Slide 66
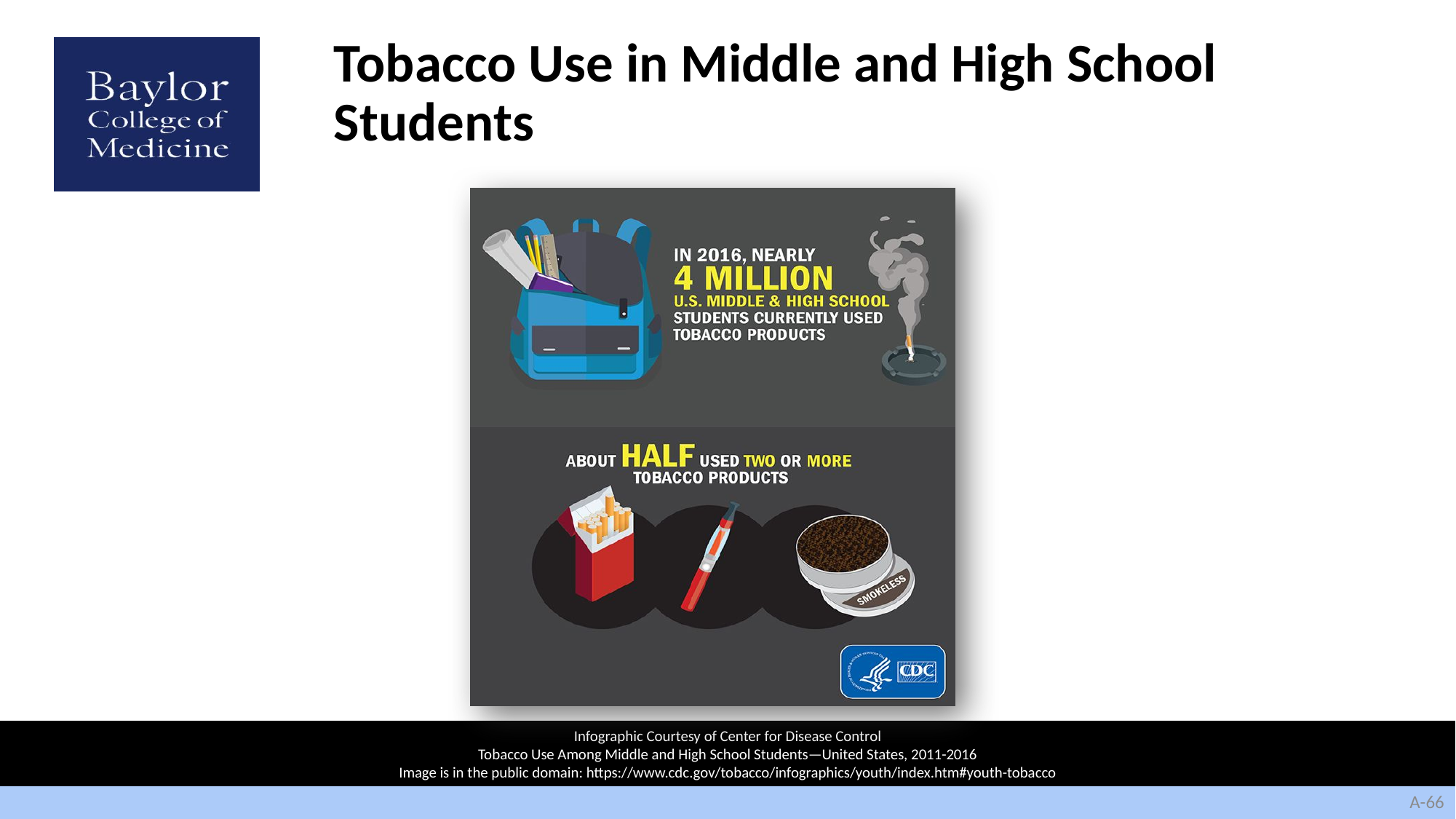

Tobacco Use in Middle and High School Students
Infographic Courtesy of Center for Disease Control
Tobacco Use Among Middle and High School Students—United States, 2011-2016
Image is in the public domain: https://www.cdc.gov/tobacco/infographics/youth/index.htm#youth-tobacco
A-66

## Slide 67
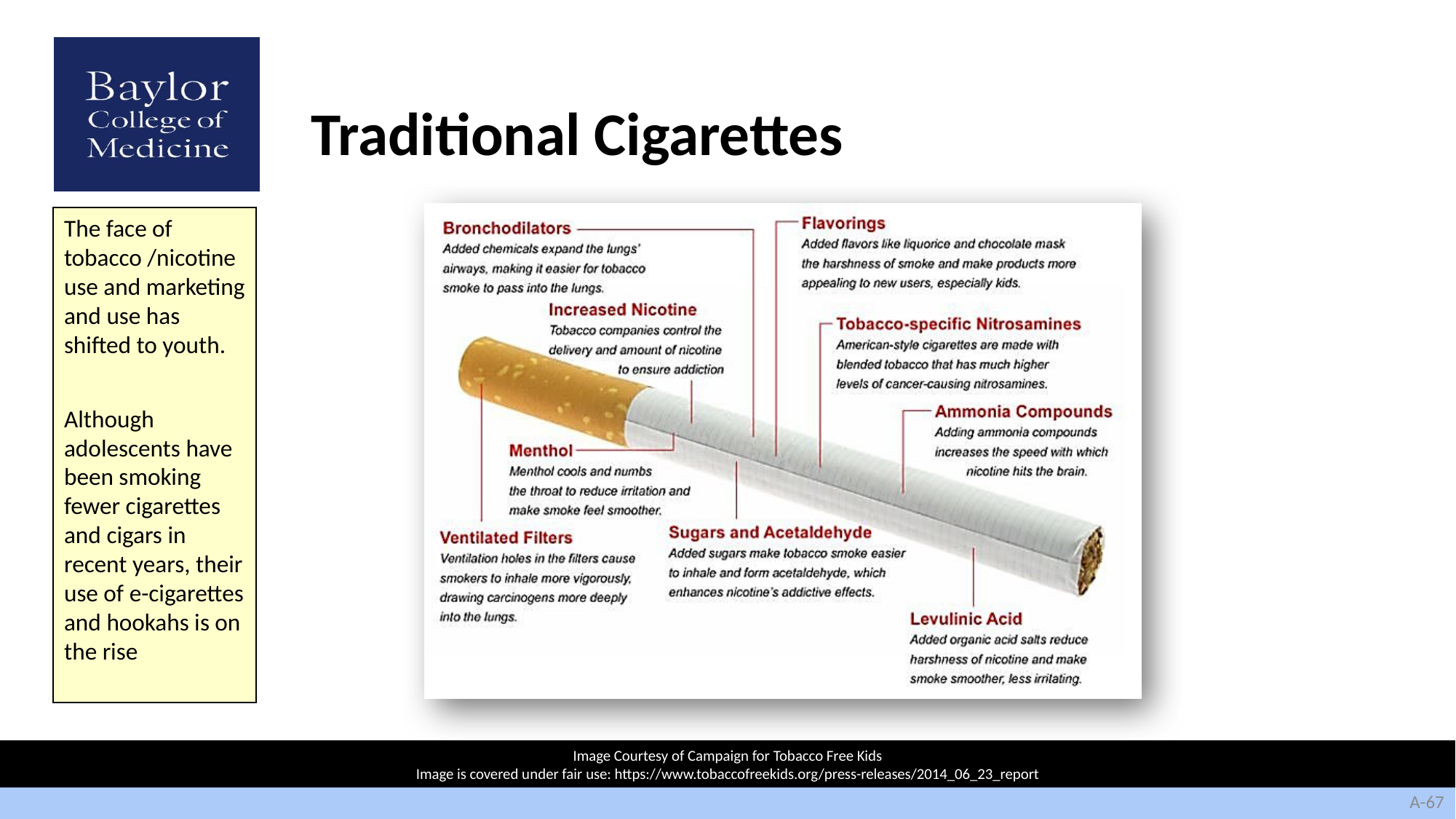

Traditional Cigarettes
The face of tobacco /nicotine use and marketing and use has shifted to youth.
Although adolescents have been smoking fewer cigarettes and cigars in recent years, their use of e-cigarettes and hookahs is on the rise
Image Courtesy of Campaign for Tobacco Free Kids
Image is covered under fair use: https://www.tobaccofreekids.org/press-releases/2014_06_23_report
A-67

## Slide 68
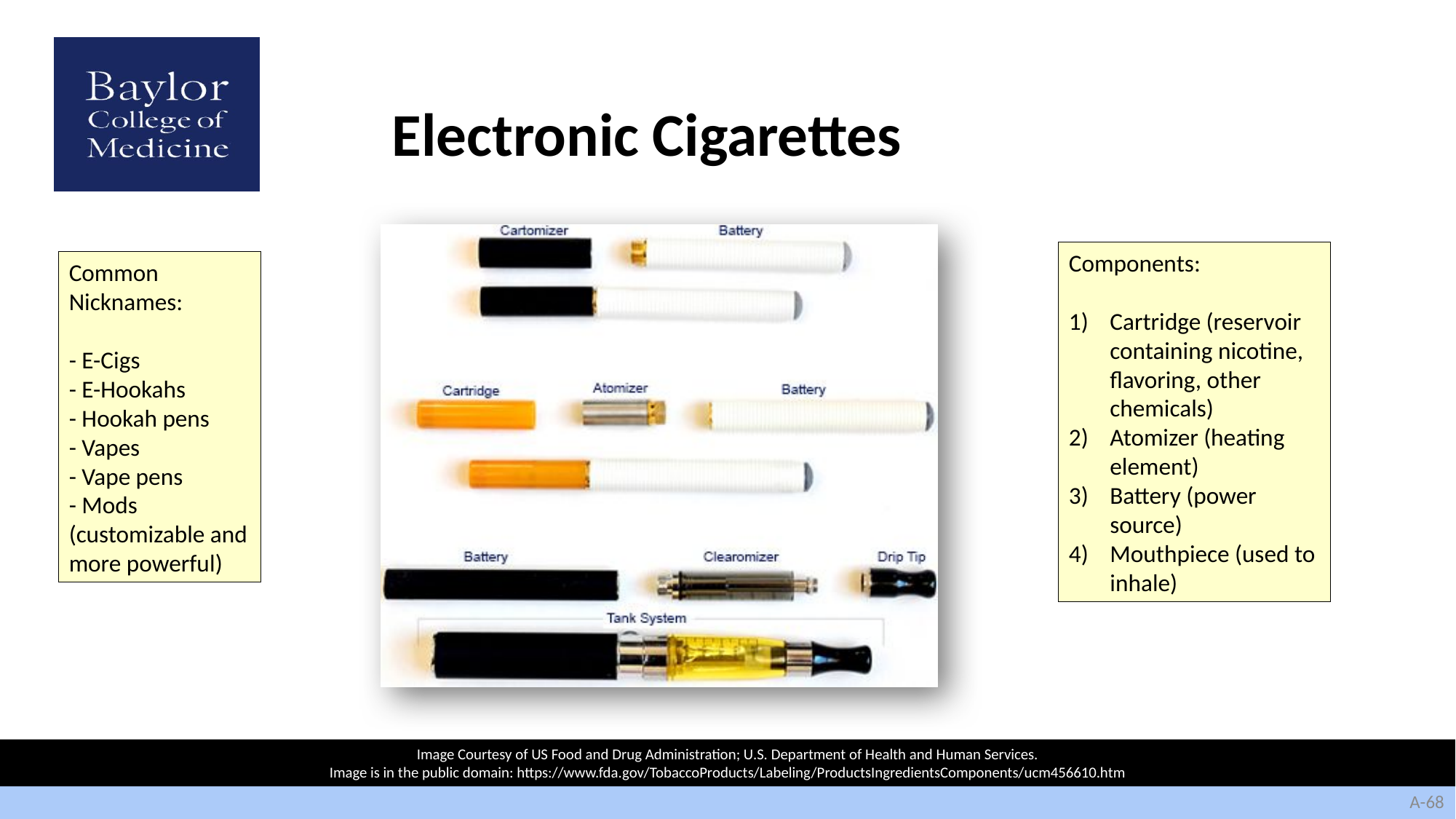

Electronic Cigarettes
Components:
Cartridge (reservoir containing nicotine, flavoring, other chemicals)
Atomizer (heating element)
Battery (power source)
Mouthpiece (used to inhale)
Common Nicknames:
- E-Cigs
- E-Hookahs
- Hookah pens
- Vapes
- Vape pens
- Mods (customizable and more powerful)
Image Courtesy of US Food and Drug Administration; U.S. Department of Health and Human Services.
Image is in the public domain: https://www.fda.gov/TobaccoProducts/Labeling/ProductsIngredientsComponents/ucm456610.htm
A-68

## Slide 69
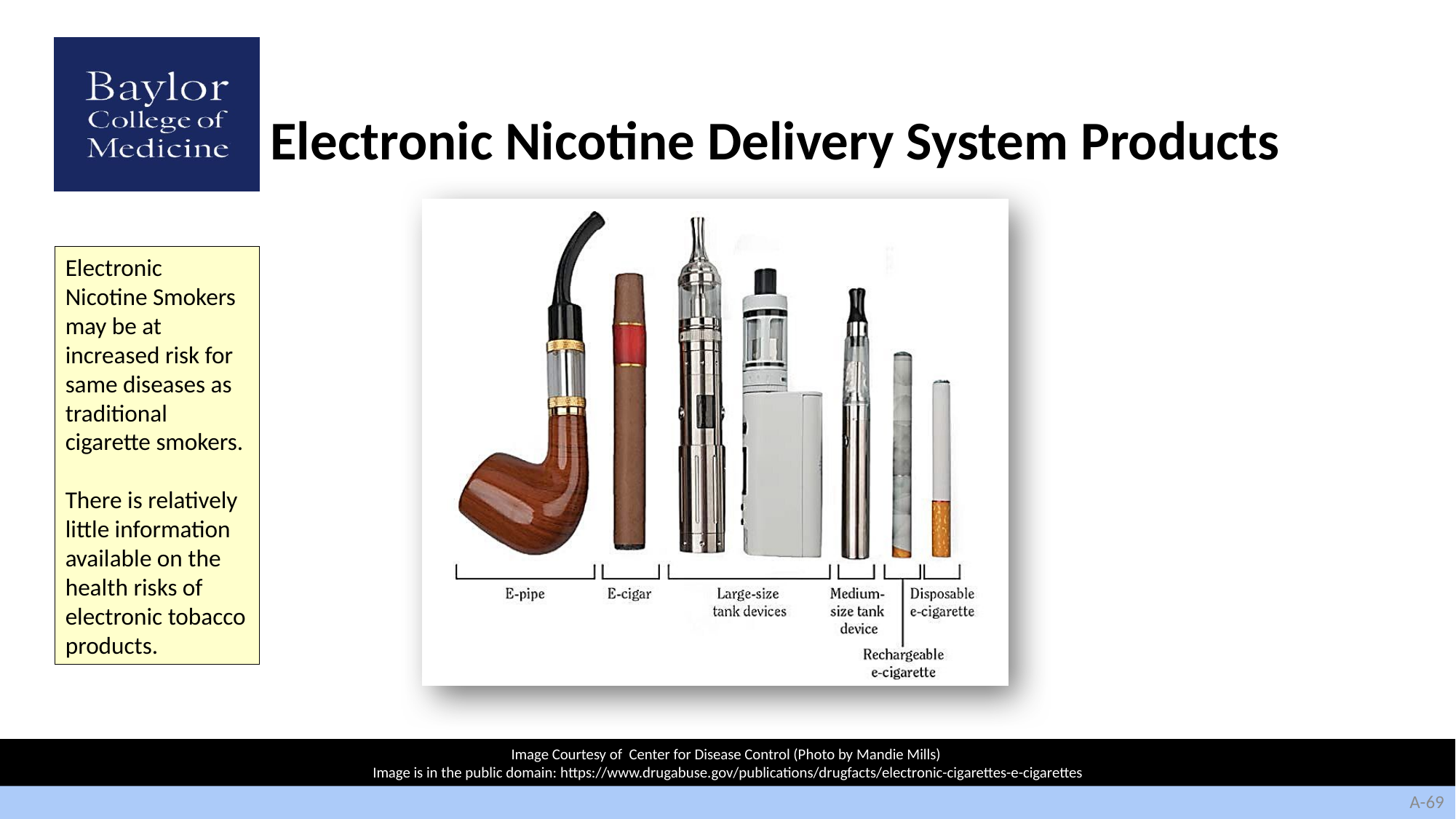

Electronic Nicotine Delivery System Products
Electronic Nicotine Smokers may be at increased risk for same diseases as traditional cigarette smokers.
There is relatively little information available on the health risks of electronic tobacco products.
Image Courtesy of Center for Disease Control (Photo by Mandie Mills)
Image is in the public domain: https://www.drugabuse.gov/publications/drugfacts/electronic-cigarettes-e-cigarettes
A-69

## Slide 70
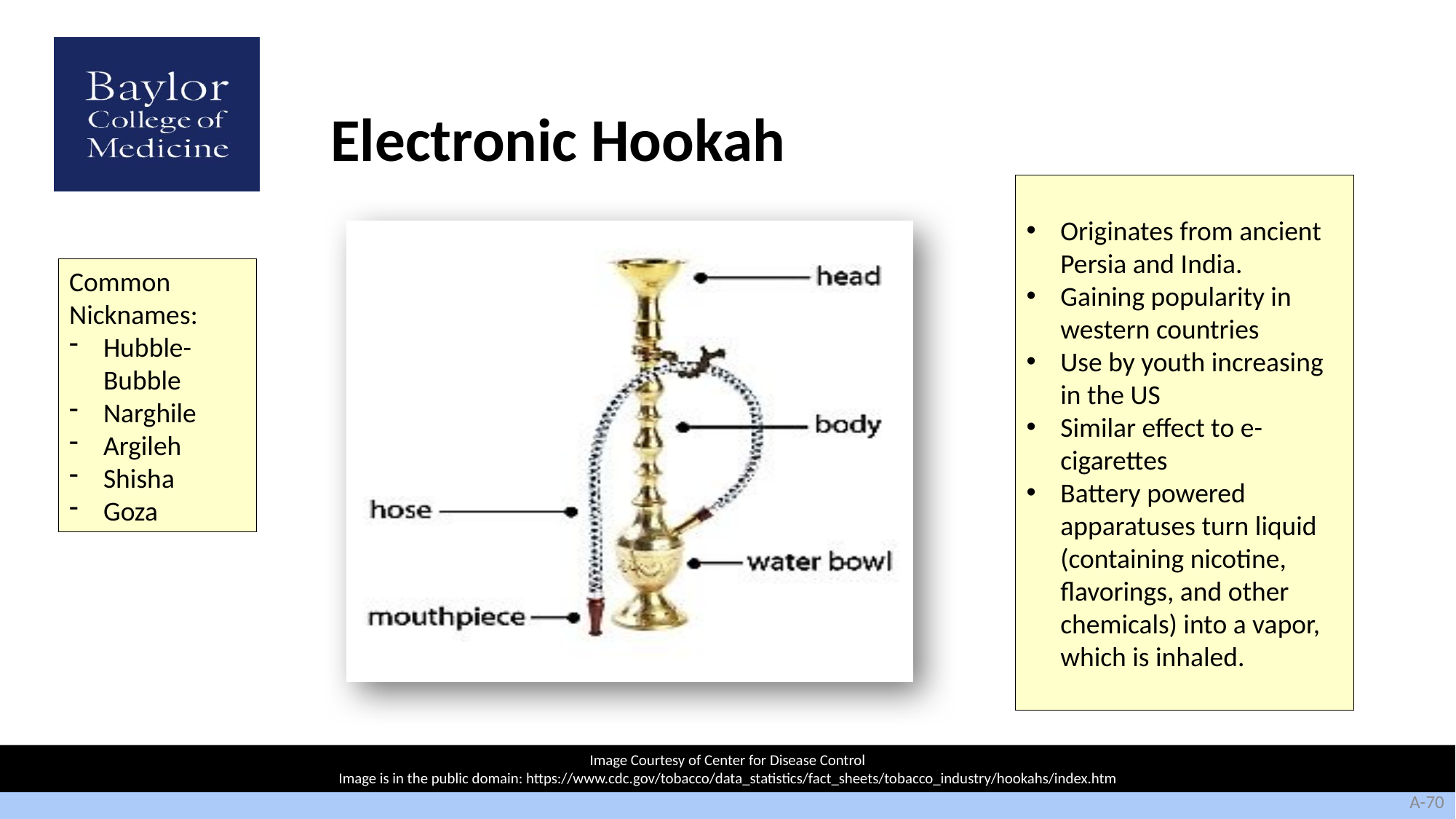

Electronic Hookah
Originates from ancient Persia and India.
Gaining popularity in western countries
Use by youth increasing in the US
Similar effect to e-cigarettes
Battery powered apparatuses turn liquid (containing nicotine, flavorings, and other chemicals) into a vapor, which is inhaled.
Common Nicknames:
Hubble-Bubble
Narghile
Argileh
Shisha
Goza
Image Courtesy of Center for Disease Control
Image is in the public domain: https://www.cdc.gov/tobacco/data_statistics/fact_sheets/tobacco_industry/hookahs/index.htm
A-70

## Slide 71
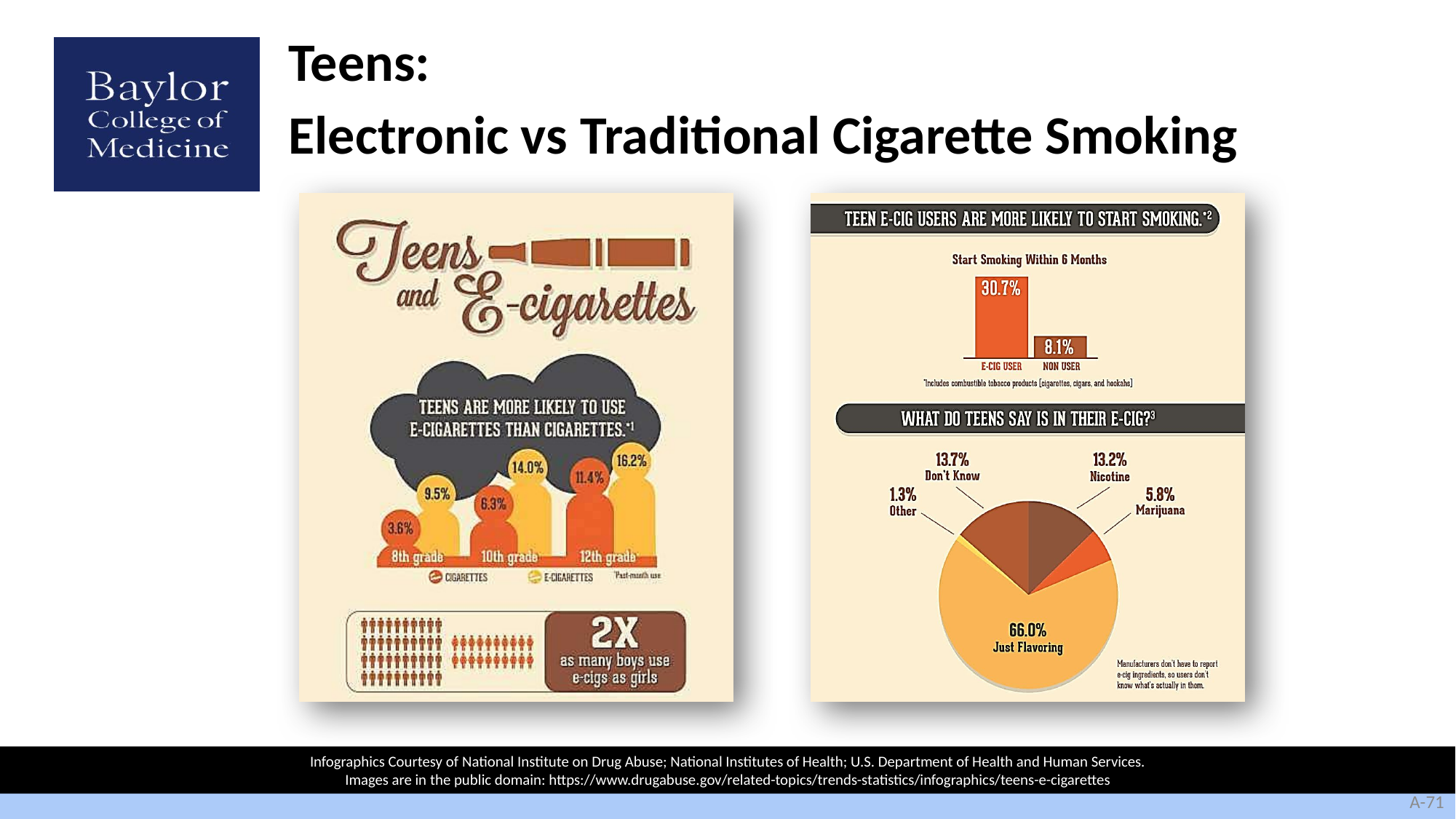

Teens:
Electronic vs Traditional Cigarette Smoking
Infographics Courtesy of National Institute on Drug Abuse; National Institutes of Health; U.S. Department of Health and Human Services.
Images are in the public domain: https://www.drugabuse.gov/related-topics/trends-statistics/infographics/teens-e-cigarettes
A-71

## Slide 72
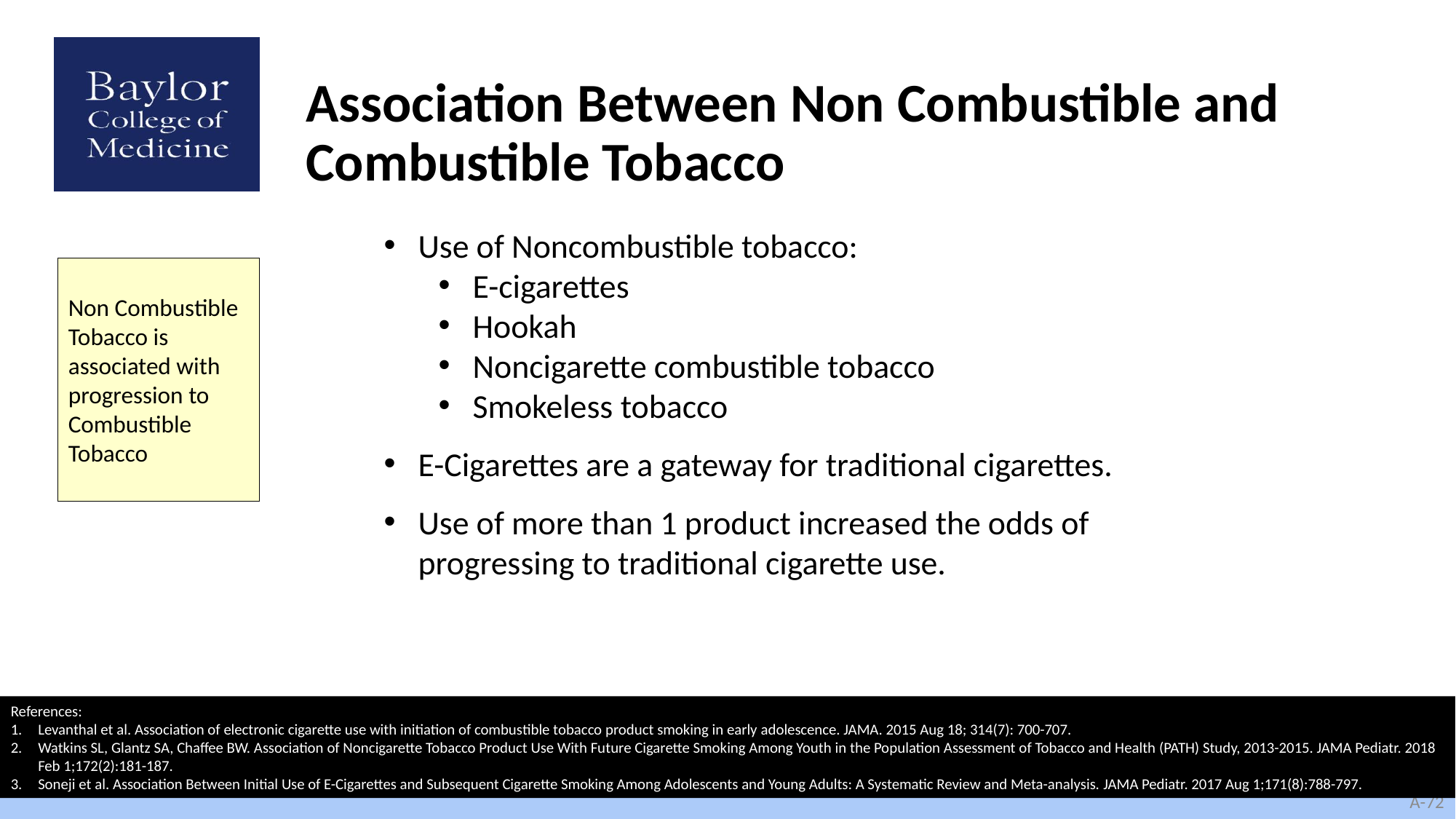

Association Between Non Combustible and Combustible Tobacco
Use of Noncombustible tobacco:
E-cigarettes
Hookah
Noncigarette combustible tobacco
Smokeless tobacco
E-Cigarettes are a gateway for traditional cigarettes.
Use of more than 1 product increased the odds of progressing to traditional cigarette use.
Non Combustible Tobacco is associated with progression to Combustible Tobacco
References:
Levanthal et al. Association of electronic cigarette use with initiation of combustible tobacco product smoking in early adolescence. JAMA. 2015 Aug 18; 314(7): 700-707.
Watkins SL, Glantz SA, Chaffee BW. Association of Noncigarette Tobacco Product Use With Future Cigarette Smoking Among Youth in the Population Assessment of Tobacco and Health (PATH) Study, 2013-2015. JAMA Pediatr. 2018 Feb 1;172(2):181-187.
Soneji et al. Association Between Initial Use of E-Cigarettes and Subsequent Cigarette Smoking Among Adolescents and Young Adults: A Systematic Review and Meta-analysis. JAMA Pediatr. 2017 Aug 1;171(8):788-797.
A-72

## Slide 73
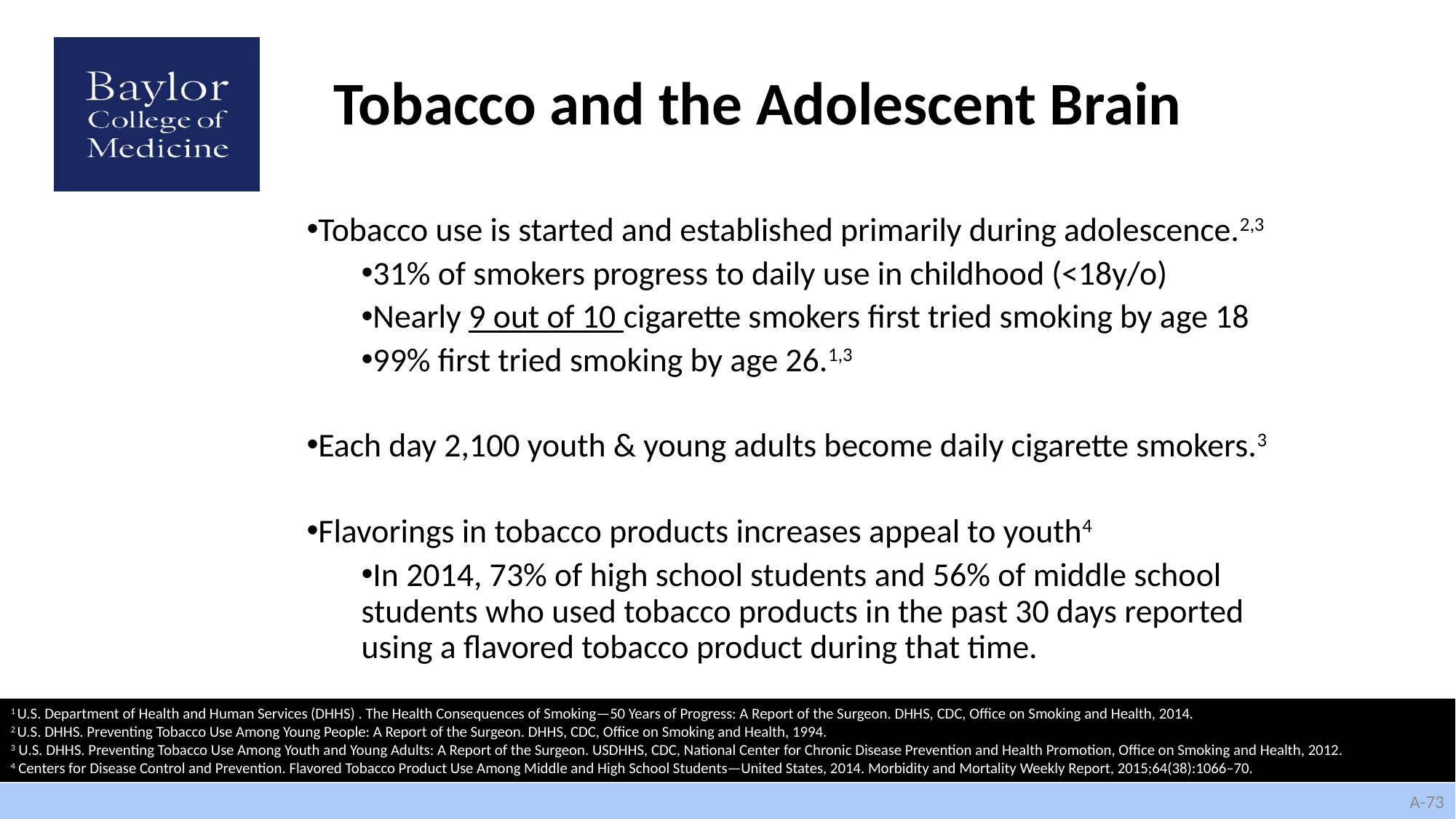

Tobacco and the Adolescent Brain
Tobacco use is started and established primarily during adolescence.2,3
31% of smokers progress to daily use in childhood (<18y/o)
Nearly 9 out of 10 cigarette smokers first tried smoking by age 18
99% first tried smoking by age 26.1,3
Each day 2,100 youth & young adults become daily cigarette smokers.3
Flavorings in tobacco products increases appeal to youth4
In 2014, 73% of high school students and 56% of middle school students who used tobacco products in the past 30 days reported using a flavored tobacco product during that time.
1 U.S. Department of Health and Human Services (DHHS) . The Health Consequences of Smoking—50 Years of Progress: A Report of the Surgeon. DHHS, CDC, Office on Smoking and Health, 2014.
2 U.S. DHHS. Preventing Tobacco Use Among Young People: A Report of the Surgeon. DHHS, CDC, Office on Smoking and Health, 1994.
3 U.S. DHHS. Preventing Tobacco Use Among Youth and Young Adults: A Report of the Surgeon. USDHHS, CDC, National Center for Chronic Disease Prevention and Health Promotion, Office on Smoking and Health, 2012.
4 Centers for Disease Control and Prevention. Flavored Tobacco Product Use Among Middle and High School Students—United States, 2014. Morbidity and Mortality Weekly Report, 2015;64(38):1066–70.
A-73

## Slide 74
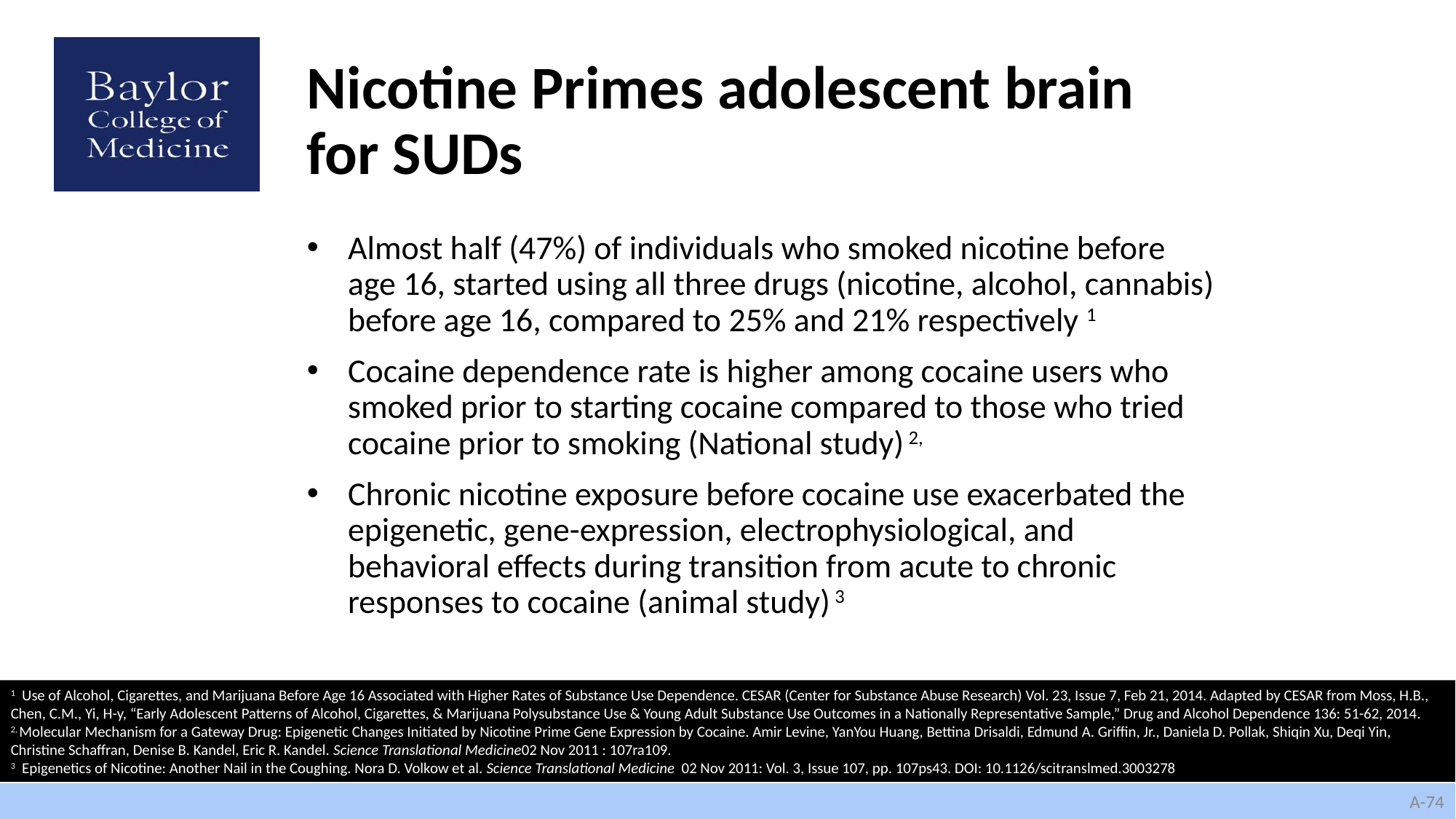

Nicotine Primes adolescent brain for SUDs
Almost half (47%) of individuals who smoked nicotine before age 16, started using all three drugs (nicotine, alcohol, cannabis) before age 16, compared to 25% and 21% respectively 1
Cocaine dependence rate is higher among cocaine users who smoked prior to starting cocaine compared to those who tried cocaine prior to smoking (National study) 2,
Chronic nicotine exposure before cocaine use exacerbated the epigenetic, gene-expression, electrophysiological, and behavioral effects during transition from acute to chronic responses to cocaine (animal study) 3
1  Use of Alcohol, Cigarettes, and Marijuana Before Age 16 Associated with Higher Rates of Substance Use Dependence. CESAR (Center for Substance Abuse Research) Vol. 23, Issue 7, Feb 21, 2014. Adapted by CESAR from Moss, H.B., Chen, C.M., Yi, H-y, “Early Adolescent Patterns of Alcohol, Cigarettes, & Marijuana Polysubstance Use & Young Adult Substance Use Outcomes in a Nationally Representative Sample,” Drug and Alcohol Dependence 136: 51-62, 2014.
2, Molecular Mechanism for a Gateway Drug: Epigenetic Changes Initiated by Nicotine Prime Gene Expression by Cocaine. Amir Levine, YanYou Huang, Bettina Drisaldi, Edmund A. Griffin, Jr., Daniela D. Pollak, Shiqin Xu, Deqi Yin, Christine Schaffran, Denise B. Kandel, Eric R. Kandel. Science Translational Medicine02 Nov 2011 : 107ra109.
3  Epigenetics of Nicotine: Another Nail in the Coughing. Nora D. Volkow et al. Science Translational Medicine  02 Nov 2011: Vol. 3, Issue 107, pp. 107ps43. DOI: 10.1126/scitranslmed.3003278
A-74

## Slide 75
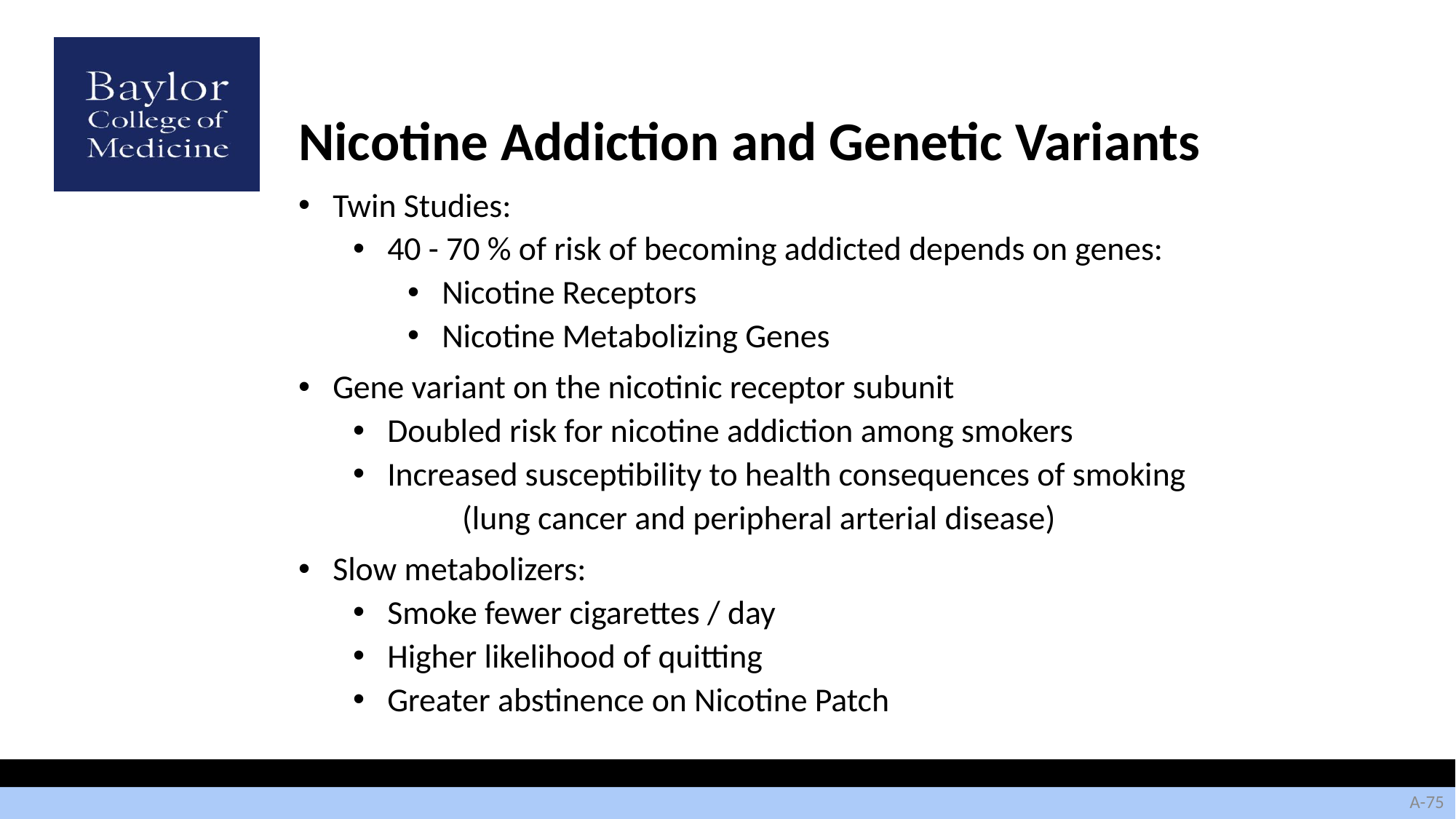

Nicotine Addiction and Genetic Variants
Twin Studies:
40 - 70 % of risk of becoming addicted depends on genes:
Nicotine Receptors
Nicotine Metabolizing Genes
Gene variant on the nicotinic receptor subunit
Doubled risk for nicotine addiction among smokers
Increased susceptibility to health consequences of smoking
	(lung cancer and peripheral arterial disease)
Slow metabolizers:
Smoke fewer cigarettes / day
Higher likelihood of quitting
Greater abstinence on Nicotine Patch
A-75

## Slide 76
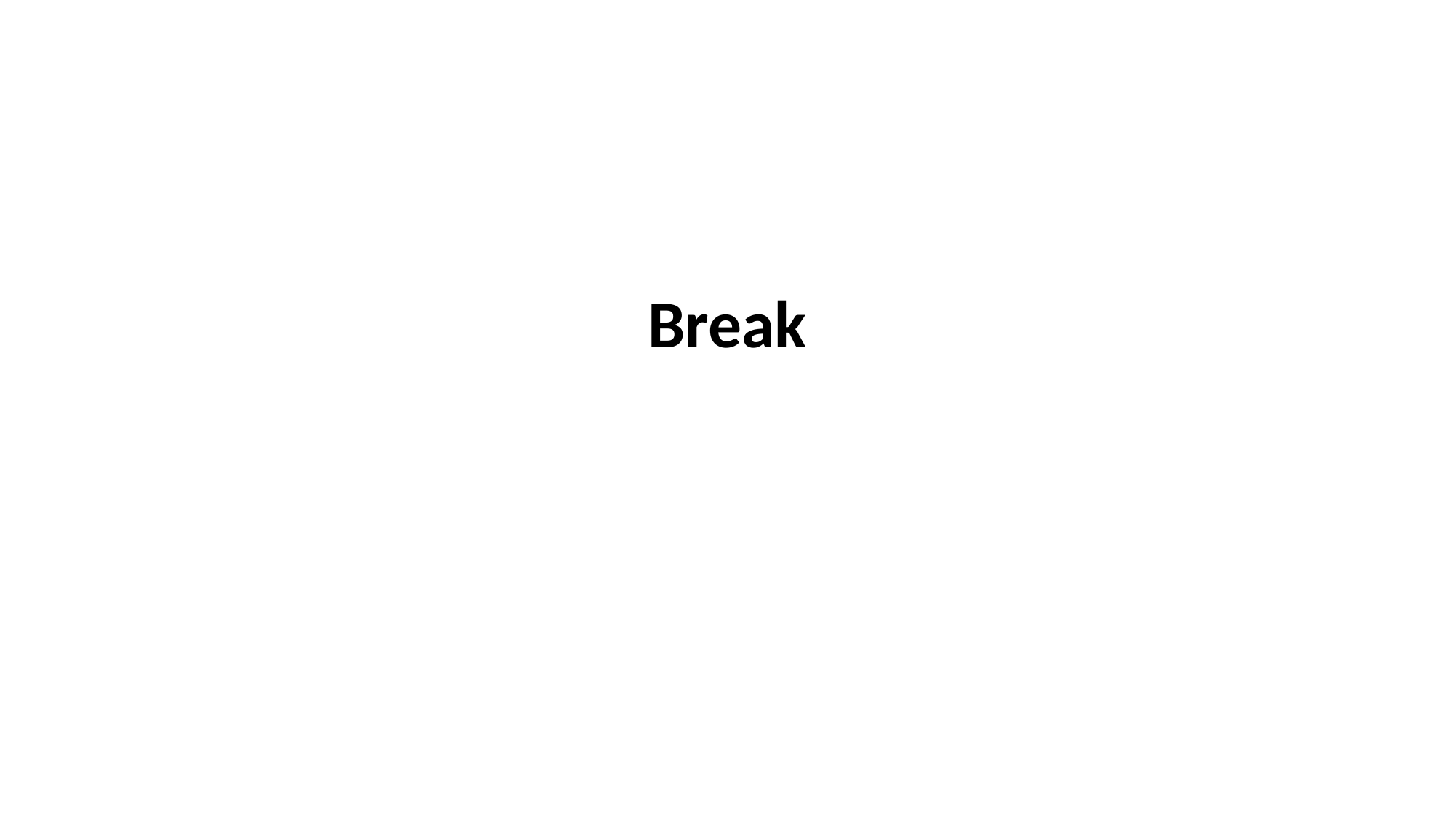

# Break

## Slide 77
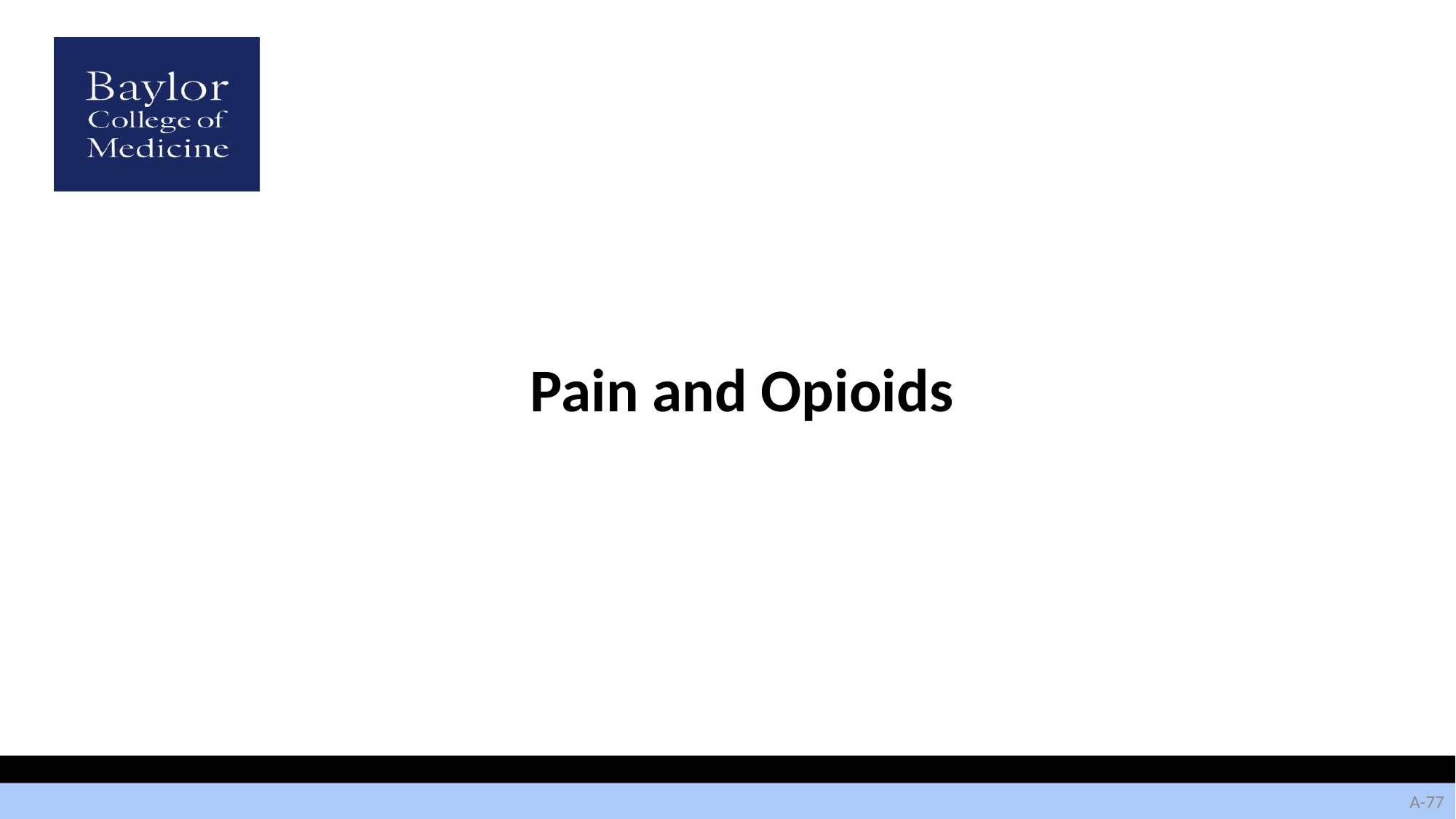

Pain and Opioids
A-77

## Slide 78
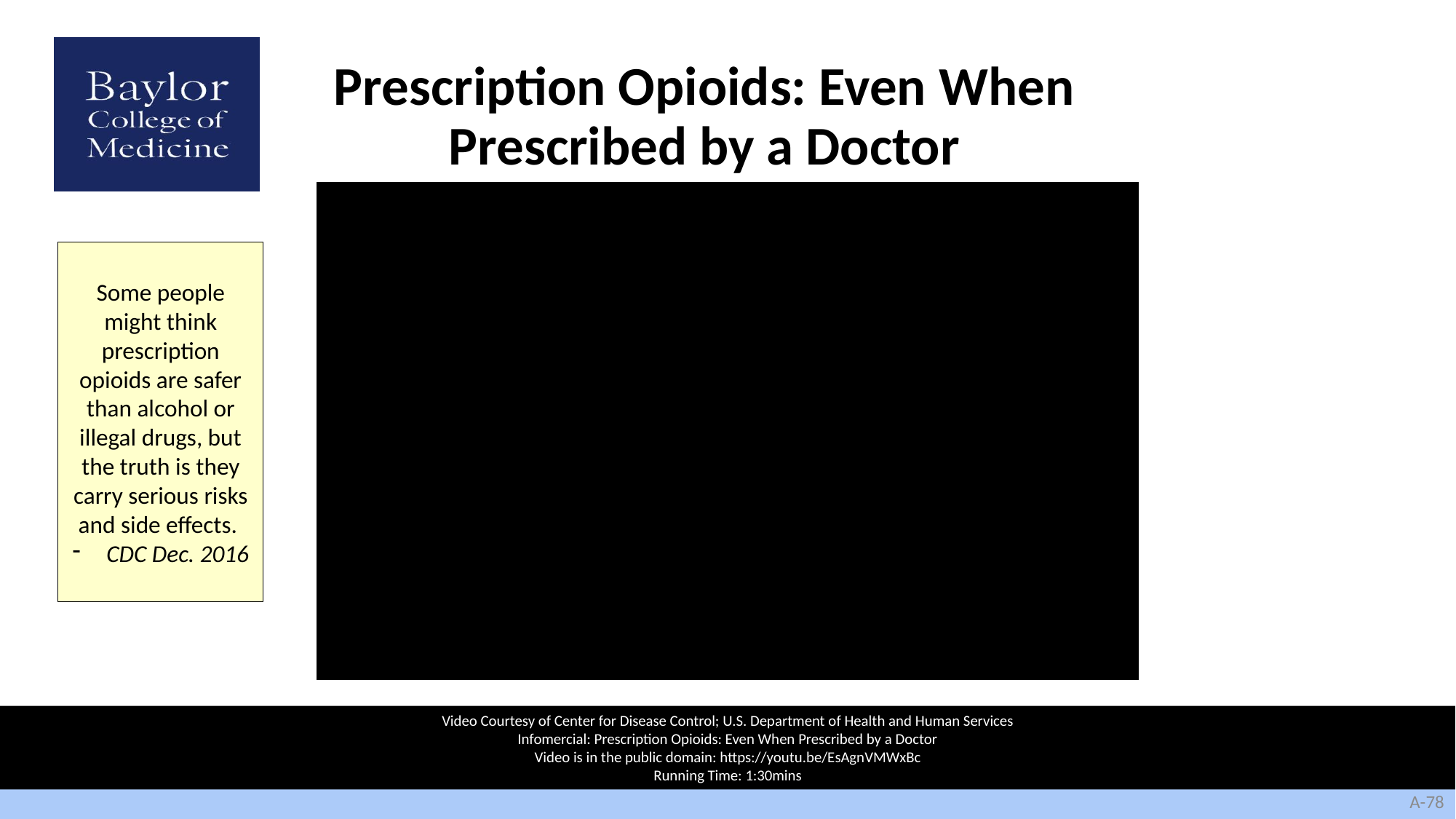

Prescription Opioids: Even When Prescribed by a Doctor
Some people might think prescription opioids are safer than alcohol or illegal drugs, but the truth is they carry serious risks and side effects.
CDC Dec. 2016
Video Courtesy of Center for Disease Control; U.S. Department of Health and Human Services
Infomercial: Prescription Opioids: Even When Prescribed by a Doctor
Video is in the public domain: https://youtu.be/EsAgnVMWxBc
Running Time: 1:30mins
A-78

## Slide 79
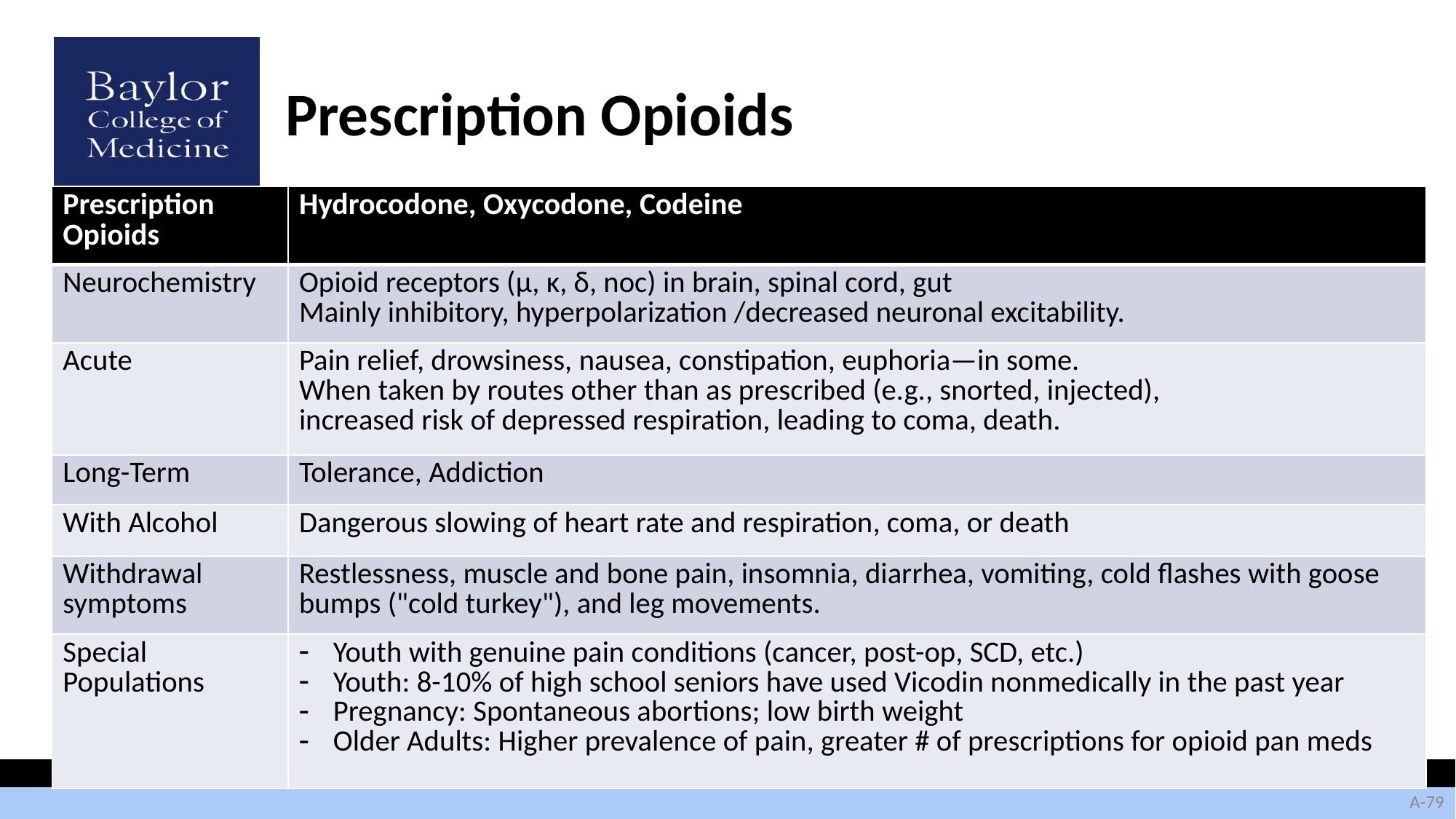

Prescription Opioids
| Prescription Opioids | Hydrocodone, Oxycodone, Codeine |
| --- | --- |
| Neurochemistry | Opioid receptors (μ, κ, δ, noc) in brain, spinal cord, gut Mainly inhibitory, hyperpolarization /decreased neuronal excitability. |
| Acute | Pain relief, drowsiness, nausea, constipation, euphoria—in some. When taken by routes other than as prescribed (e.g., snorted, injected), increased risk of depressed respiration, leading to coma, death. |
| Long-Term | Tolerance, Addiction |
| With Alcohol | Dangerous slowing of heart rate and respiration, coma, or death |
| Withdrawal symptoms | Restlessness, muscle and bone pain, insomnia, diarrhea, vomiting, cold flashes with goose bumps ("cold turkey"), and leg movements. |
| Special Populations | Youth with genuine pain conditions (cancer, post-op, SCD, etc.) Youth: 8-10% of high school seniors have used Vicodin nonmedically in the past year Pregnancy: Spontaneous abortions; low birth weight Older Adults: Higher prevalence of pain, greater # of prescriptions for opioid pan meds |
A-79

## Slide 80
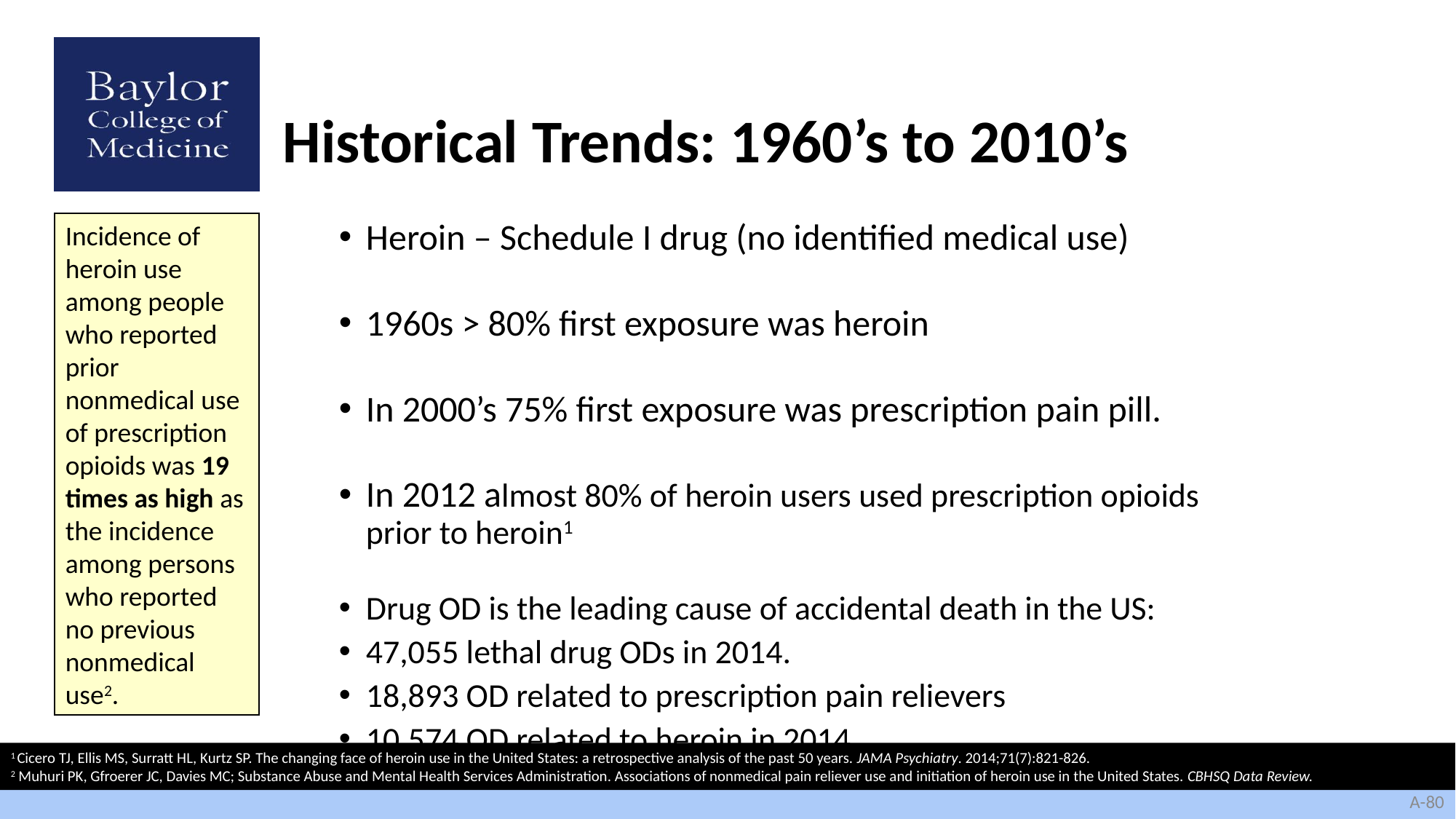

Historical Trends: 1960’s to 2010’s
Incidence of heroin use among people who reported prior nonmedical use of prescription opioids was 19 times as high as the incidence among persons who reported no previous nonmedical use2.
Heroin – Schedule I drug (no identified medical use)
1960s > 80% first exposure was heroin
In 2000’s 75% first exposure was prescription pain pill.
In 2012 almost 80% of heroin users used prescription opioids prior to heroin1
Drug OD is the leading cause of accidental death in the US:
47,055 lethal drug ODs in 2014.
18,893 OD related to prescription pain relievers
10,574 OD related to heroin in 2014.
1 Cicero TJ, Ellis MS, Surratt HL, Kurtz SP. The changing face of heroin use in the United States: a retrospective analysis of the past 50 years. JAMA Psychiatry. 2014;71(7):821-826.
2 Muhuri PK, Gfroerer JC, Davies MC; Substance Abuse and Mental Health Services Administration. Associations of nonmedical pain reliever use and initiation of heroin use in the United States. CBHSQ Data Review.
A-80

## Slide 81
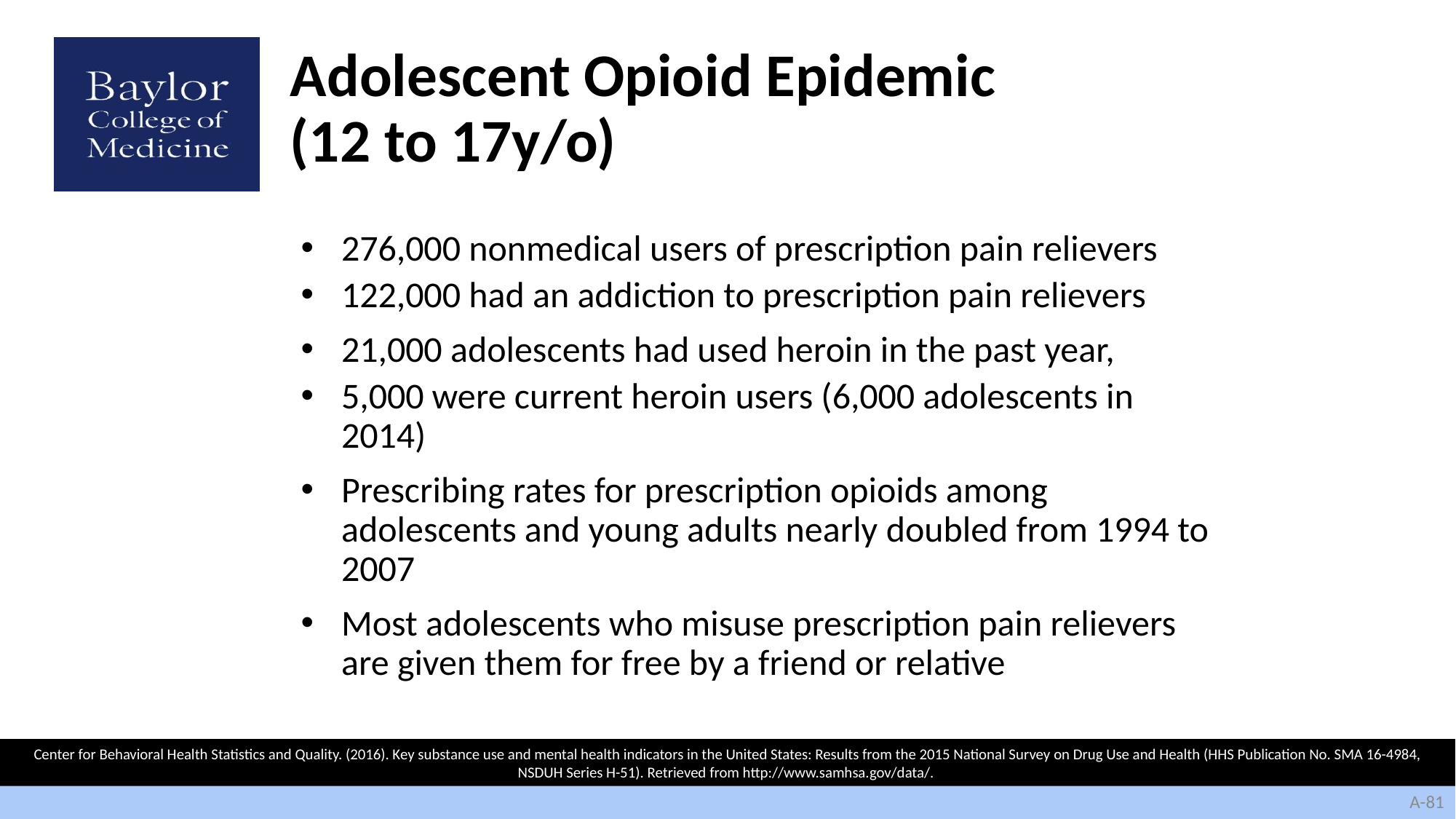

Adolescent Opioid Epidemic
(12 to 17y/o)
276,000 nonmedical users of prescription pain relievers
122,000 had an addiction to prescription pain relievers
21,000 adolescents had used heroin in the past year,
5,000 were current heroin users (6,000 adolescents in 2014)
Prescribing rates for prescription opioids among adolescents and young adults nearly doubled from 1994 to 2007
Most adolescents who misuse prescription pain relievers are given them for free by a friend or relative
Center for Behavioral Health Statistics and Quality. (2016). Key substance use and mental health indicators in the United States: Results from the 2015 National Survey on Drug Use and Health (HHS Publication No. SMA 16-4984, NSDUH Series H-51). Retrieved from http://www.samhsa.gov/data/.
A-81

## Slide 82
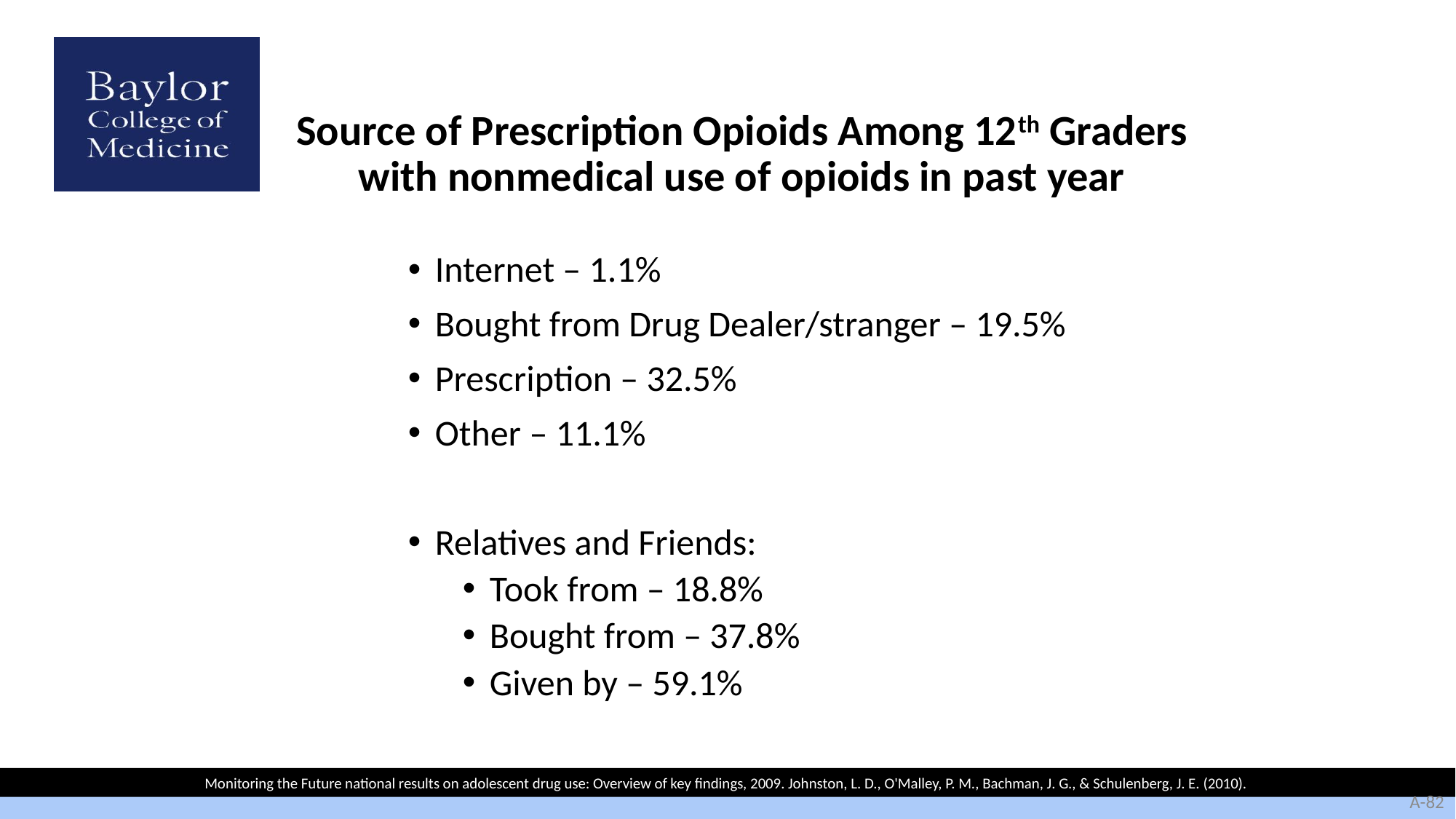

Source of Prescription Opioids Among 12th Graders with nonmedical use of opioids in past year
Internet – 1.1%
Bought from Drug Dealer/stranger – 19.5%
Prescription – 32.5%
Other – 11.1%
Relatives and Friends:
Took from – 18.8%
Bought from – 37.8%
Given by – 59.1%
Monitoring the Future national results on adolescent drug use: Overview of key findings, 2009. Johnston, L. D., O'Malley, P. M., Bachman, J. G., & Schulenberg, J. E. (2010).
A-82

## Slide 83
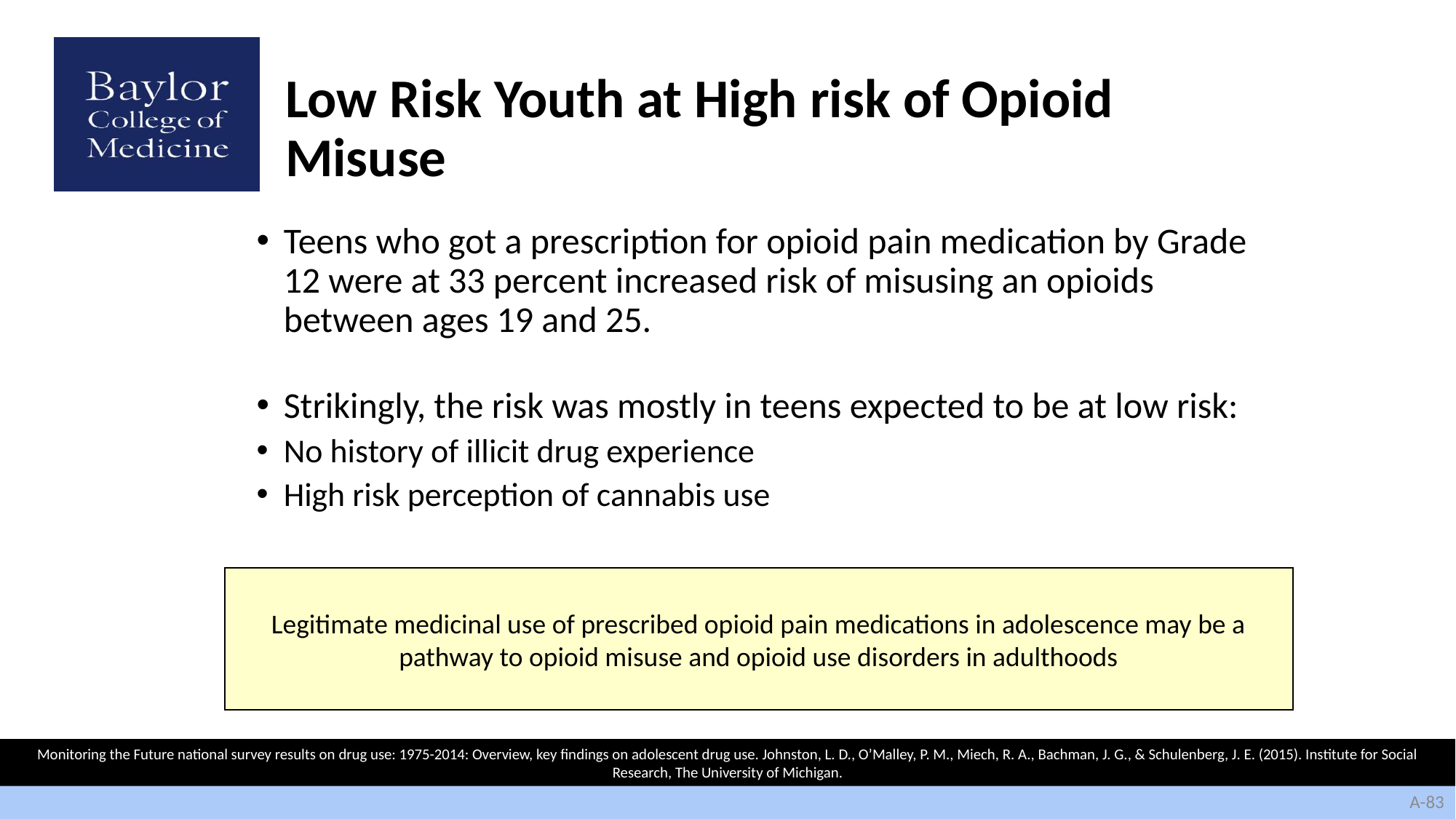

Low Risk Youth at High risk of Opioid Misuse
Teens who got a prescription for opioid pain medication by Grade 12 were at 33 percent increased risk of misusing an opioids between ages 19 and 25.
Strikingly, the risk was mostly in teens expected to be at low risk:
No history of illicit drug experience
High risk perception of cannabis use
Legitimate medicinal use of prescribed opioid pain medications in adolescence may be a pathway to opioid misuse and opioid use disorders in adulthoods
Monitoring the Future national survey results on drug use: 1975-2014: Overview, key findings on adolescent drug use. Johnston, L. D., O’Malley, P. M., Miech, R. A., Bachman, J. G., & Schulenberg, J. E. (2015). Institute for Social Research, The University of Michigan.
A-83

## Slide 84
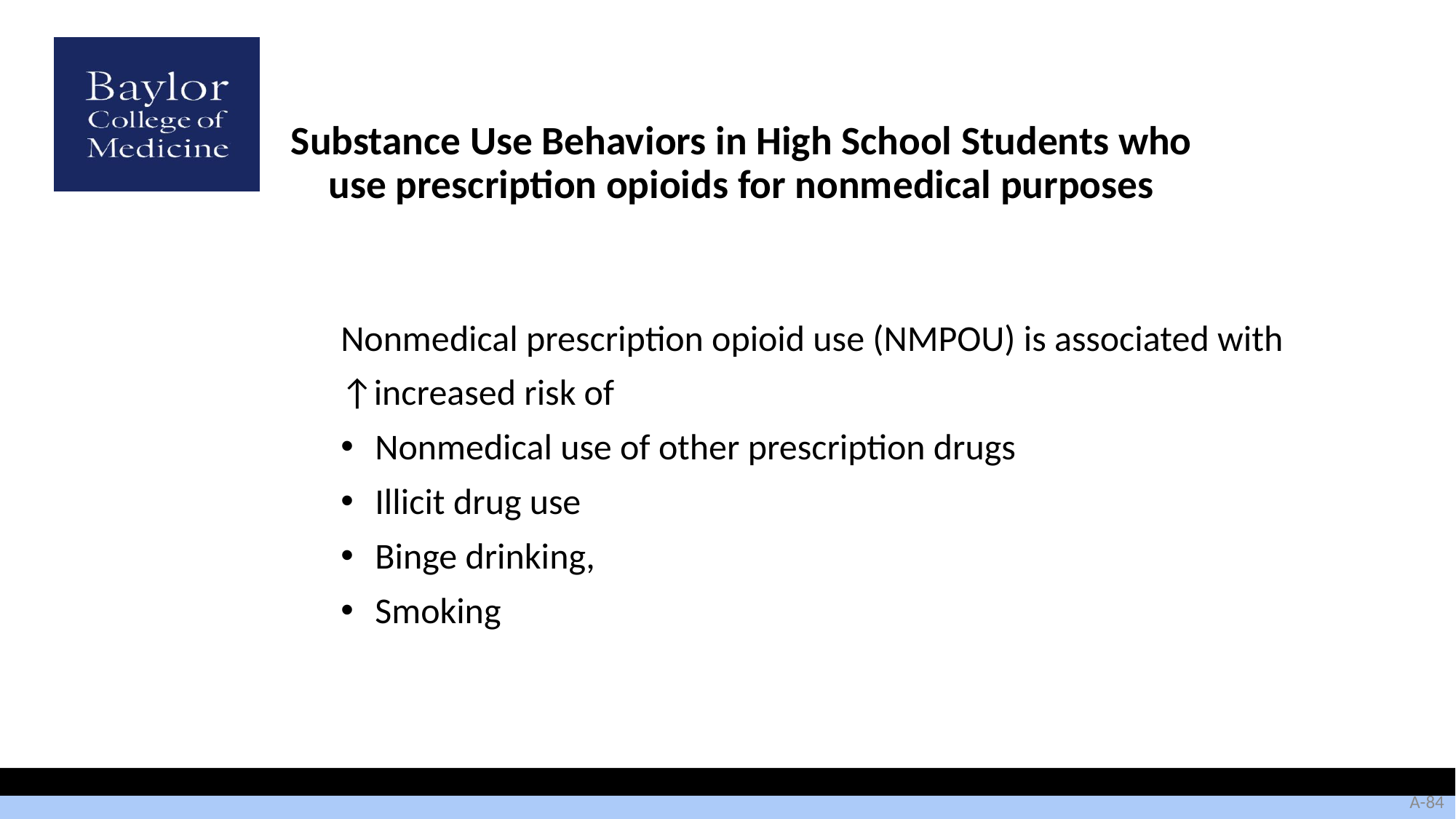

Substance Use Behaviors in High School Students who use prescription opioids for nonmedical purposes
Nonmedical prescription opioid use (NMPOU) is associated with
↑increased risk of
Nonmedical use of other prescription drugs
Illicit drug use
Binge drinking,
Smoking
A-84

## Slide 85
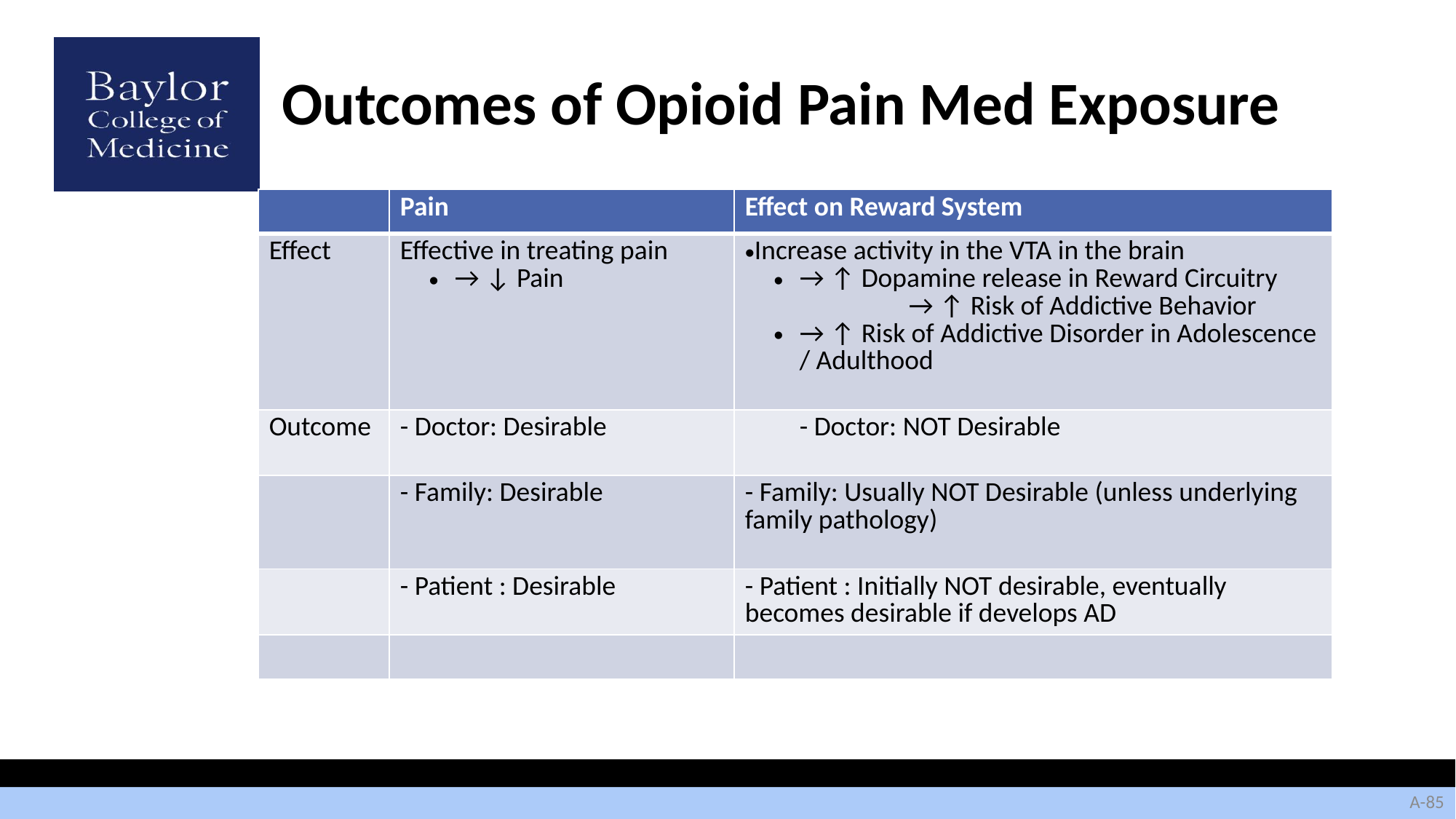

Outcomes of Opioid Pain Med Exposure
| | Pain | Effect on Reward System |
| --- | --- | --- |
| Effect | Effective in treating pain → ↓ Pain | Increase activity in the VTA in the brain → ↑ Dopamine release in Reward Circuitry → ↑ Risk of Addictive Behavior → ↑ Risk of Addictive Disorder in Adolescence / Adulthood |
| Outcome | - Doctor: Desirable | - Doctor: NOT Desirable |
| | - Family: Desirable | - Family: Usually NOT Desirable (unless underlying family pathology) |
| | - Patient : Desirable | - Patient : Initially NOT desirable, eventually becomes desirable if develops AD |
| | | |
A-85

## Slide 86
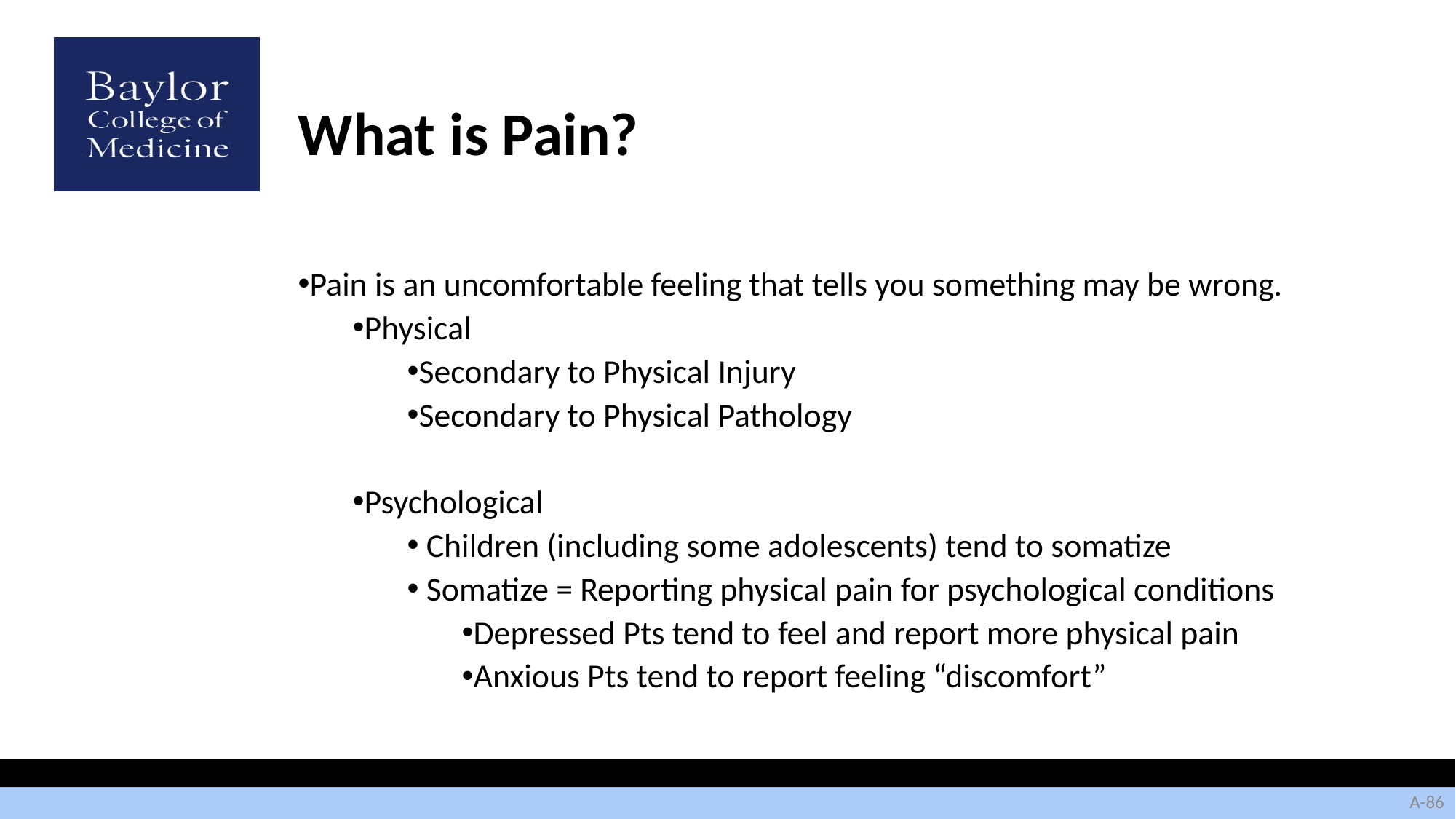

What is Pain?
Pain is an uncomfortable feeling that tells you something may be wrong.
Physical
Secondary to Physical Injury
Secondary to Physical Pathology
Psychological
 Children (including some adolescents) tend to somatize
 Somatize = Reporting physical pain for psychological conditions
Depressed Pts tend to feel and report more physical pain
Anxious Pts tend to report feeling “discomfort”
A-86

## Slide 87
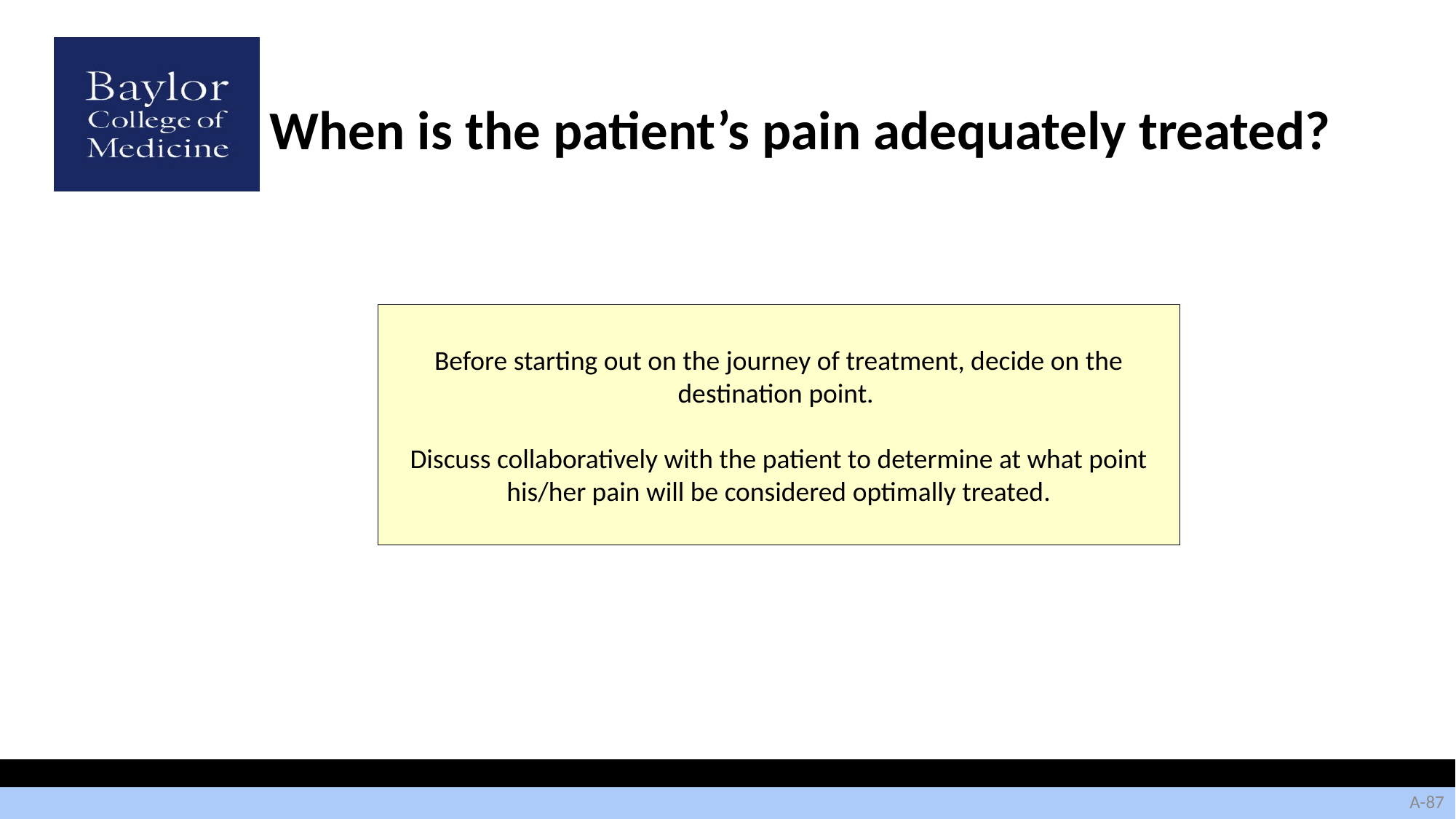

When is the patient’s pain adequately treated?
Before starting out on the journey of treatment, decide on the destination point.
Discuss collaboratively with the patient to determine at what point his/her pain will be considered optimally treated.
A-87

## Slide 88
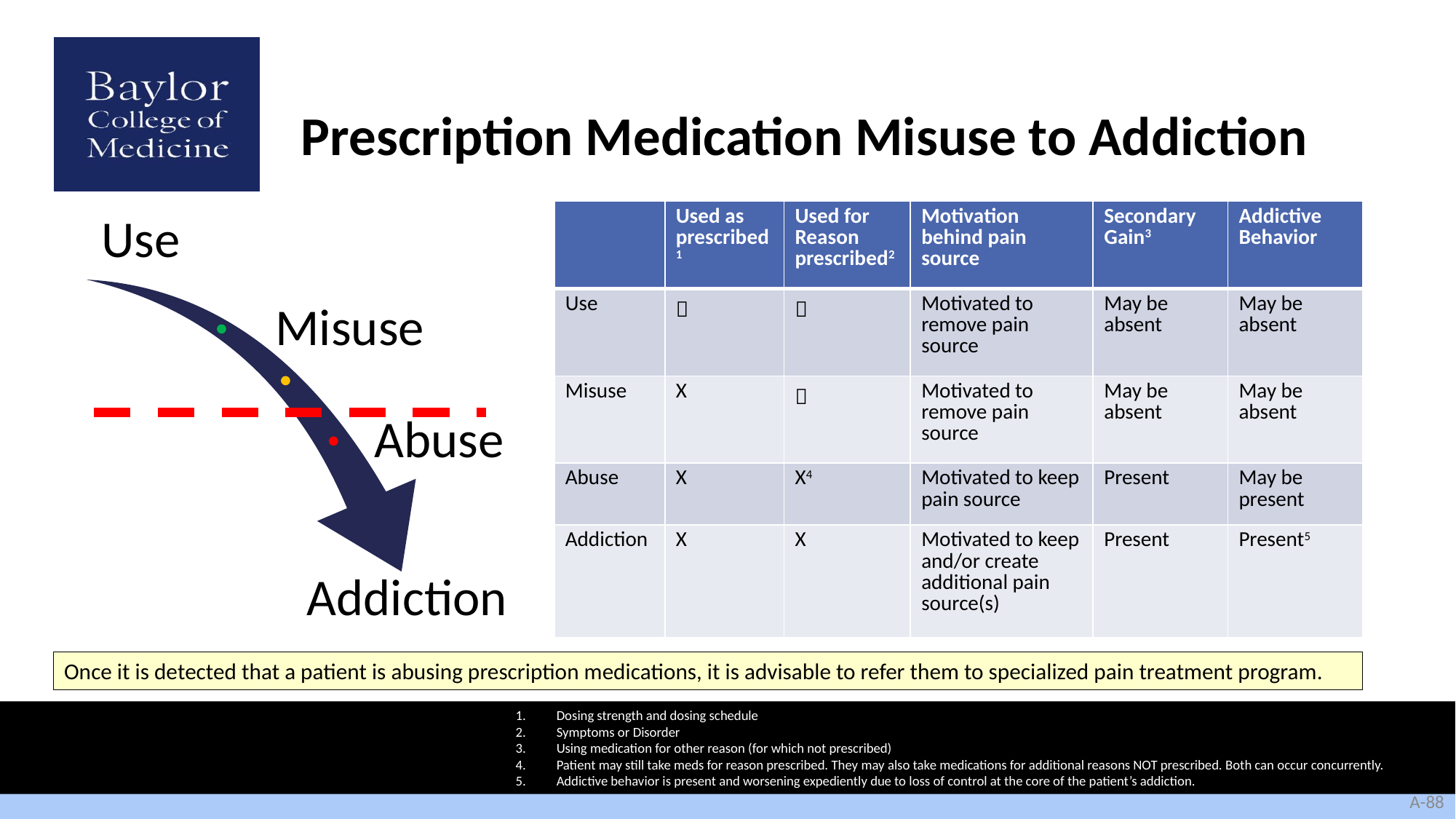

Prescription Medication Misuse to Addiction
| | Used as prescribed1 | Used for Reason prescribed2 | Motivation behind pain source | Secondary Gain3 | Addictive Behavior |
| --- | --- | --- | --- | --- | --- |
| Use |  |  | Motivated to remove pain source | May be absent | May be absent |
| Misuse | X |  | Motivated to remove pain source | May be absent | May be absent |
| Abuse | X | X4 | Motivated to keep pain source | Present | May be present |
| Addiction | X | X | Motivated to keep and/or create additional pain source(s) | Present | Present5 |
Once it is detected that a patient is abusing prescription medications, it is advisable to refer them to specialized pain treatment program.
Dosing strength and dosing schedule
Symptoms or Disorder
Using medication for other reason (for which not prescribed)
Patient may still take meds for reason prescribed. They may also take medications for additional reasons NOT prescribed. Both can occur concurrently.
Addictive behavior is present and worsening expediently due to loss of control at the core of the patient’s addiction.
A-88

## Slide 89
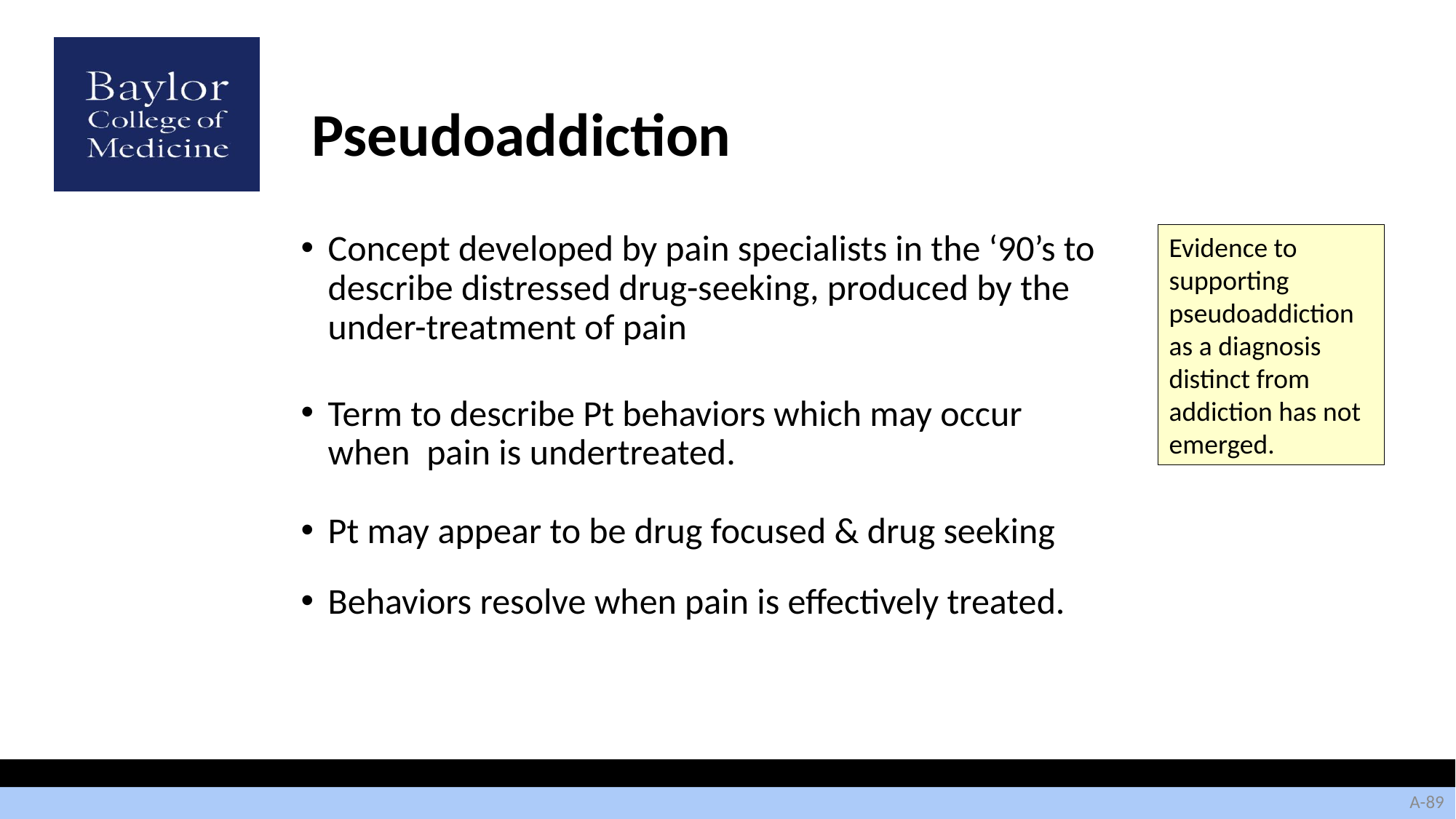

Pseudoaddiction
Concept developed by pain specialists in the ‘90’s to describe distressed drug-seeking, produced by the under-treatment of pain
Term to describe Pt behaviors which may occur when pain is undertreated.
Pt may appear to be drug focused & drug seeking
Behaviors resolve when pain is effectively treated.
Evidence to supporting pseudoaddiction as a diagnosis distinct from addiction has not emerged.
A-89

## Slide 90
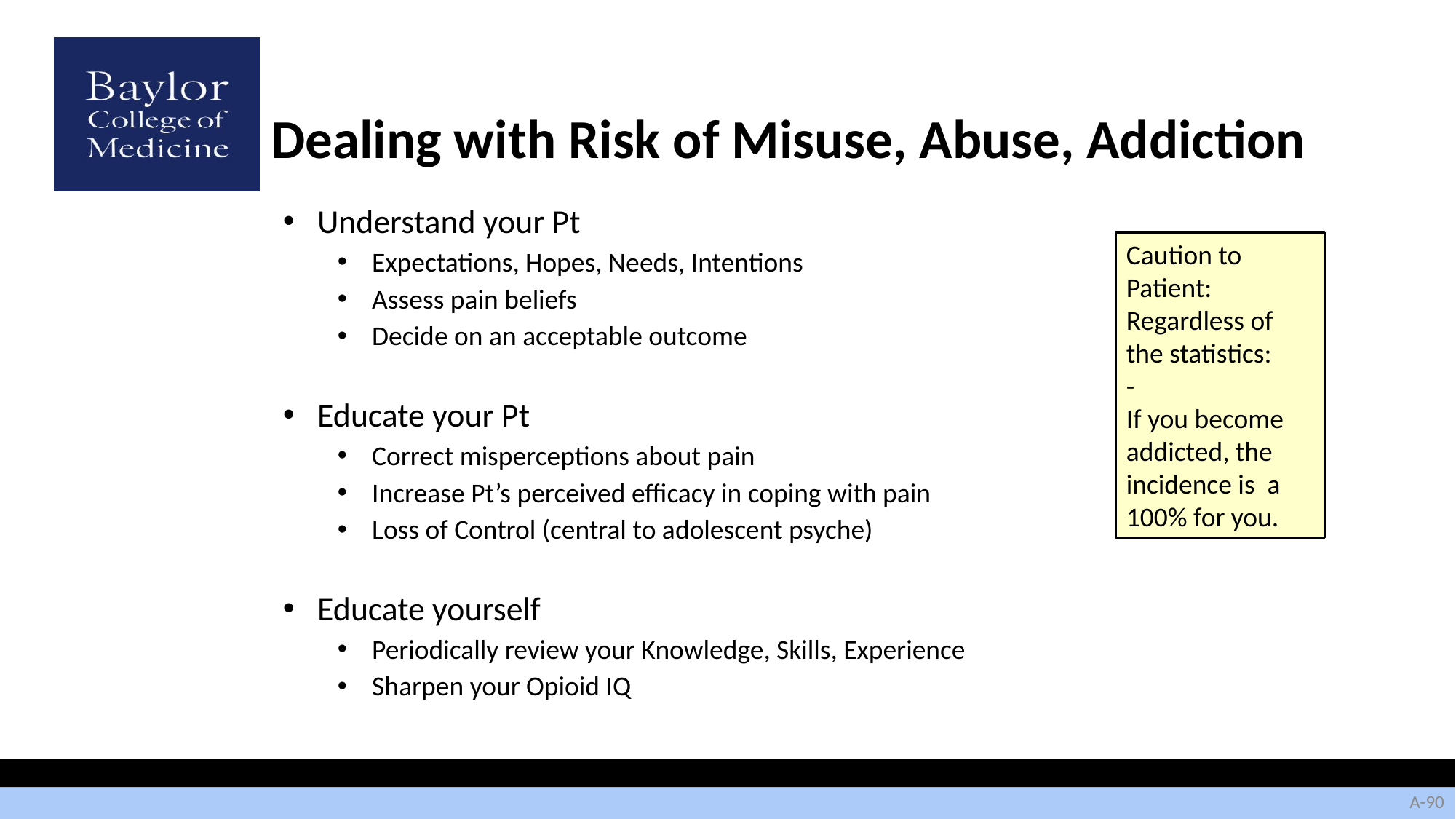

Dealing with Risk of Misuse, Abuse, Addiction
Understand your Pt
Expectations, Hopes, Needs, Intentions
Assess pain beliefs
Decide on an acceptable outcome
Educate your Pt
Correct misperceptions about pain
Increase Pt’s perceived efficacy in coping with pain
Loss of Control (central to adolescent psyche)
Educate yourself
Periodically review your Knowledge, Skills, Experience
Sharpen your Opioid IQ
Caution to Patient:
Regardless of the statistics:
-
If you become addicted, the incidence is a 100% for you.
A-90

## Slide 91
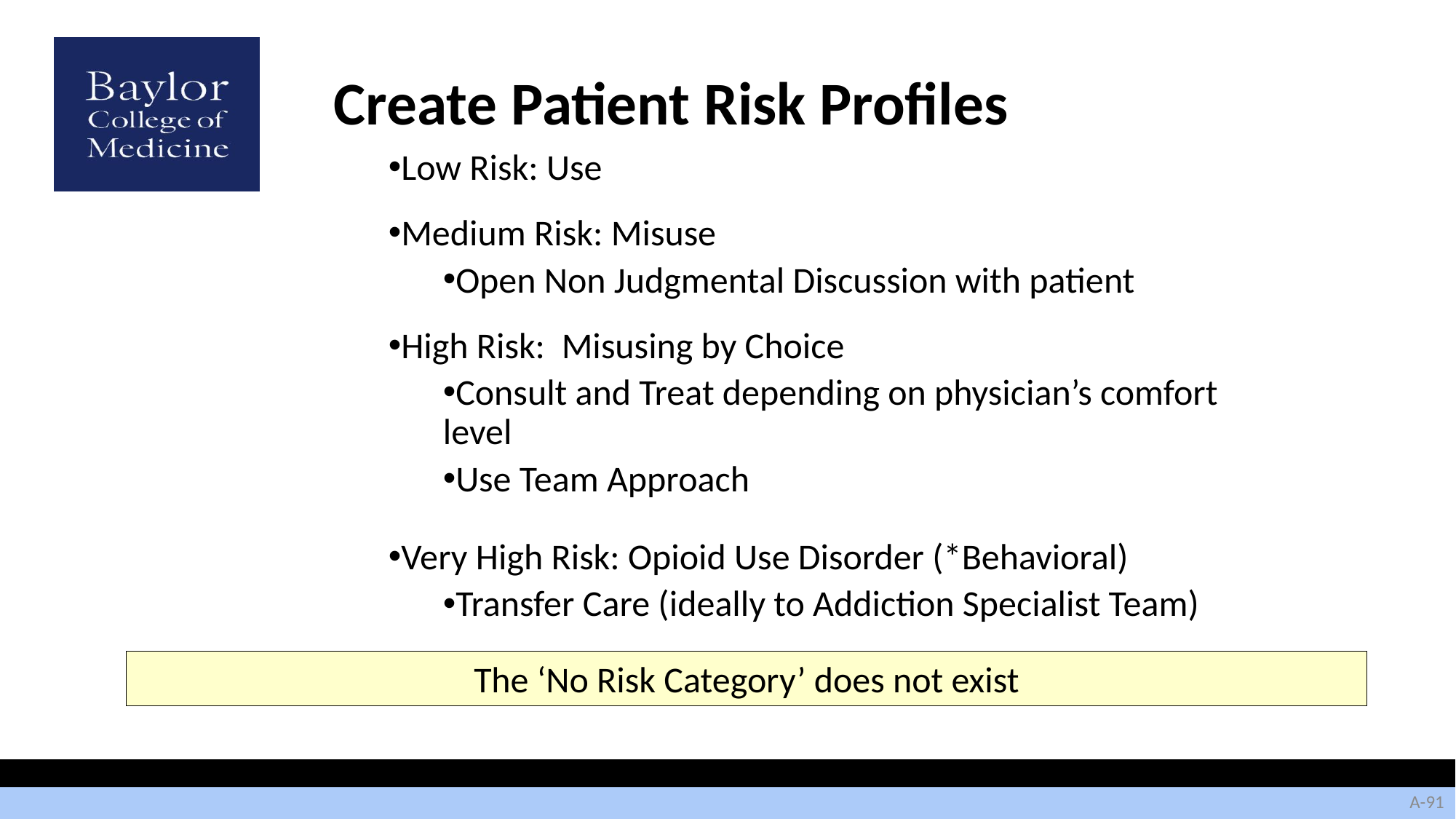

Create Patient Risk Profiles
Low Risk: Use
Medium Risk: Misuse
Open Non Judgmental Discussion with patient
High Risk: Misusing by Choice
Consult and Treat depending on physician’s comfort level
Use Team Approach
Very High Risk: Opioid Use Disorder (*Behavioral)
Transfer Care (ideally to Addiction Specialist Team)
The ‘No Risk Category’ does not exist
A-91

## Slide 92
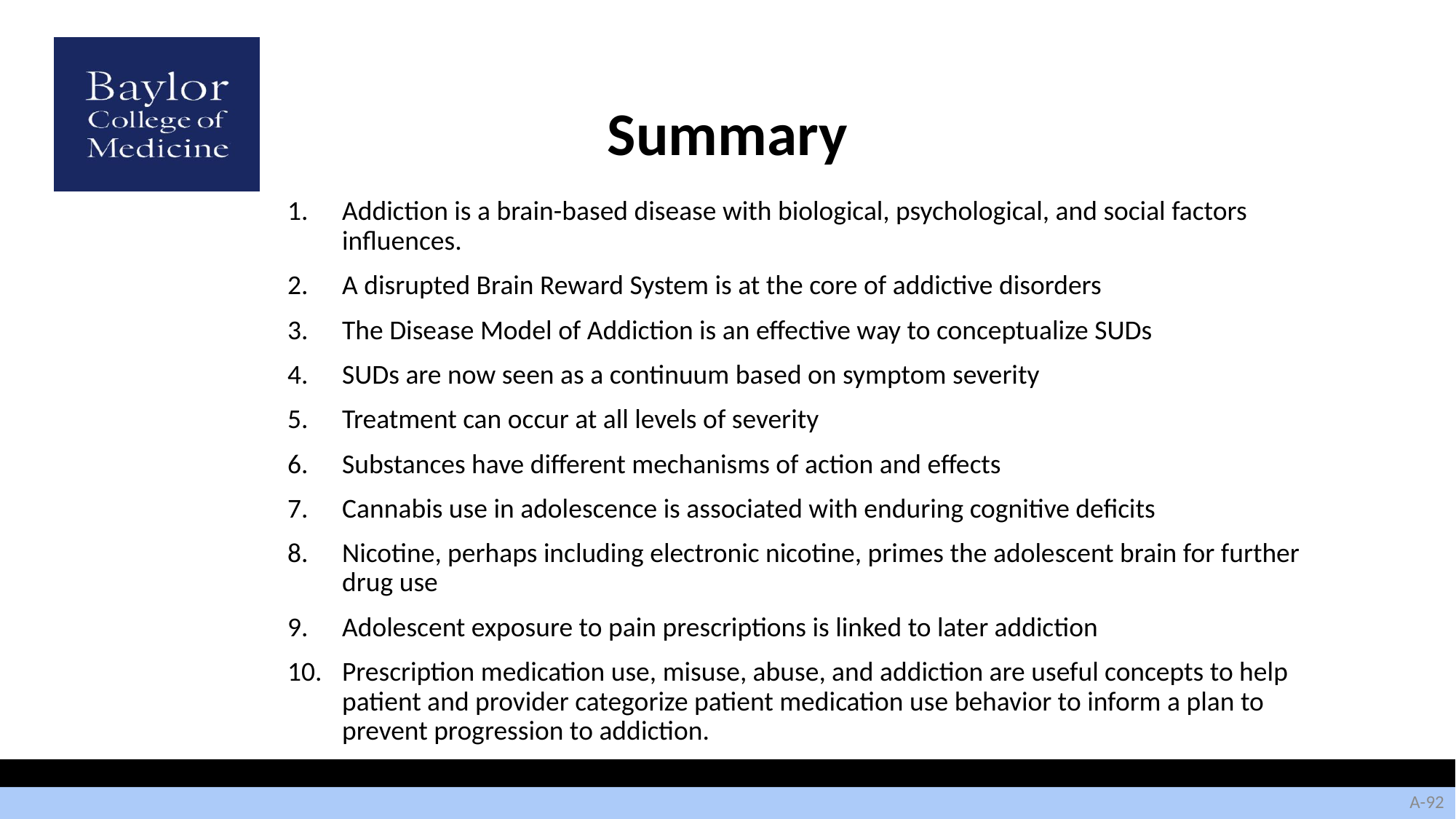

Summary
Addiction is a brain-based disease with biological, psychological, and social factors influences.
A disrupted Brain Reward System is at the core of addictive disorders
The Disease Model of Addiction is an effective way to conceptualize SUDs
SUDs are now seen as a continuum based on symptom severity
Treatment can occur at all levels of severity
Substances have different mechanisms of action and effects
Cannabis use in adolescence is associated with enduring cognitive deficits
Nicotine, perhaps including electronic nicotine, primes the adolescent brain for further drug use
Adolescent exposure to pain prescriptions is linked to later addiction
Prescription medication use, misuse, abuse, and addiction are useful concepts to help patient and provider categorize patient medication use behavior to inform a plan to prevent progression to addiction.
A-92

## Slide 93
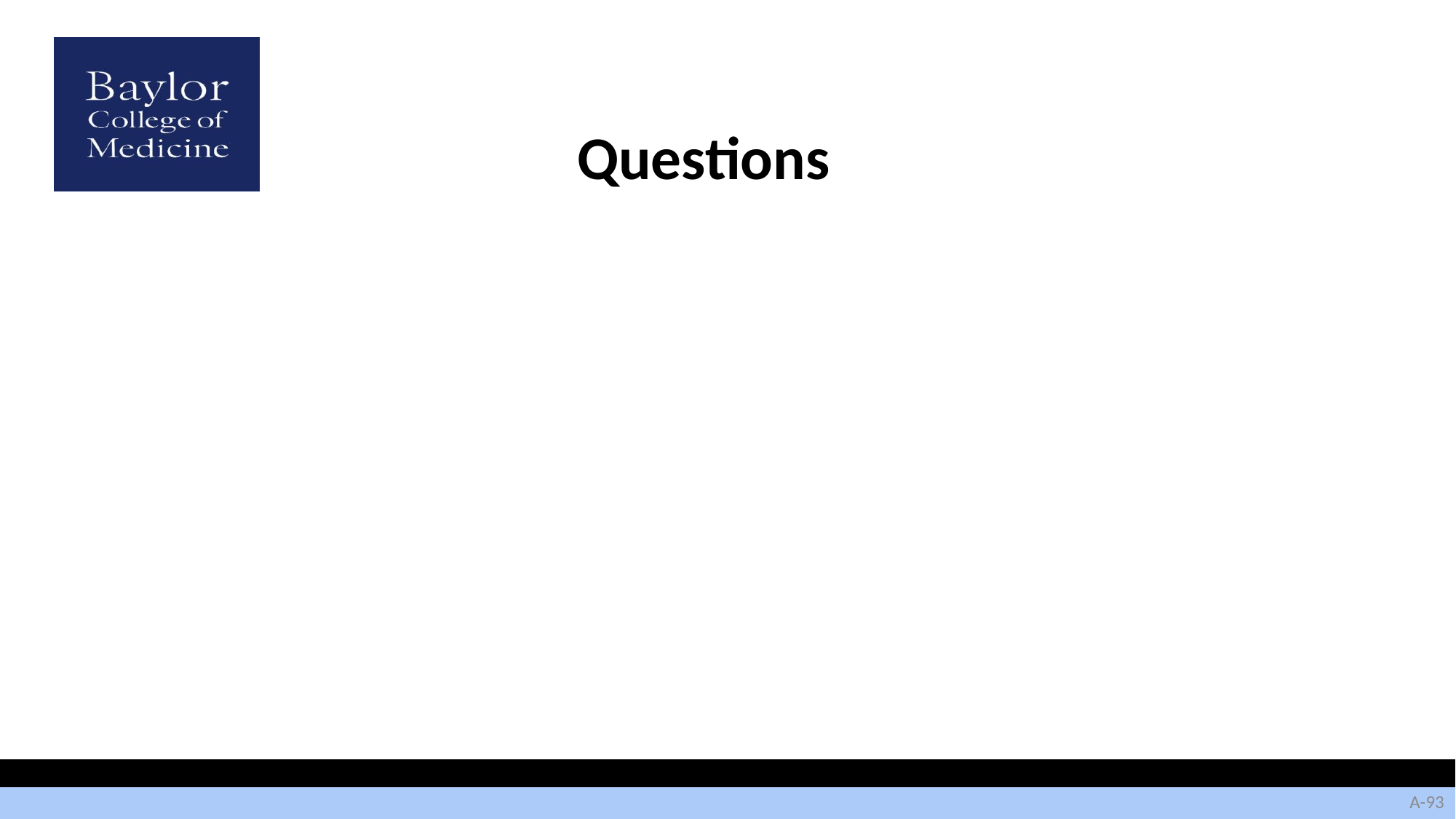

Questions
A-93

## Slide 94
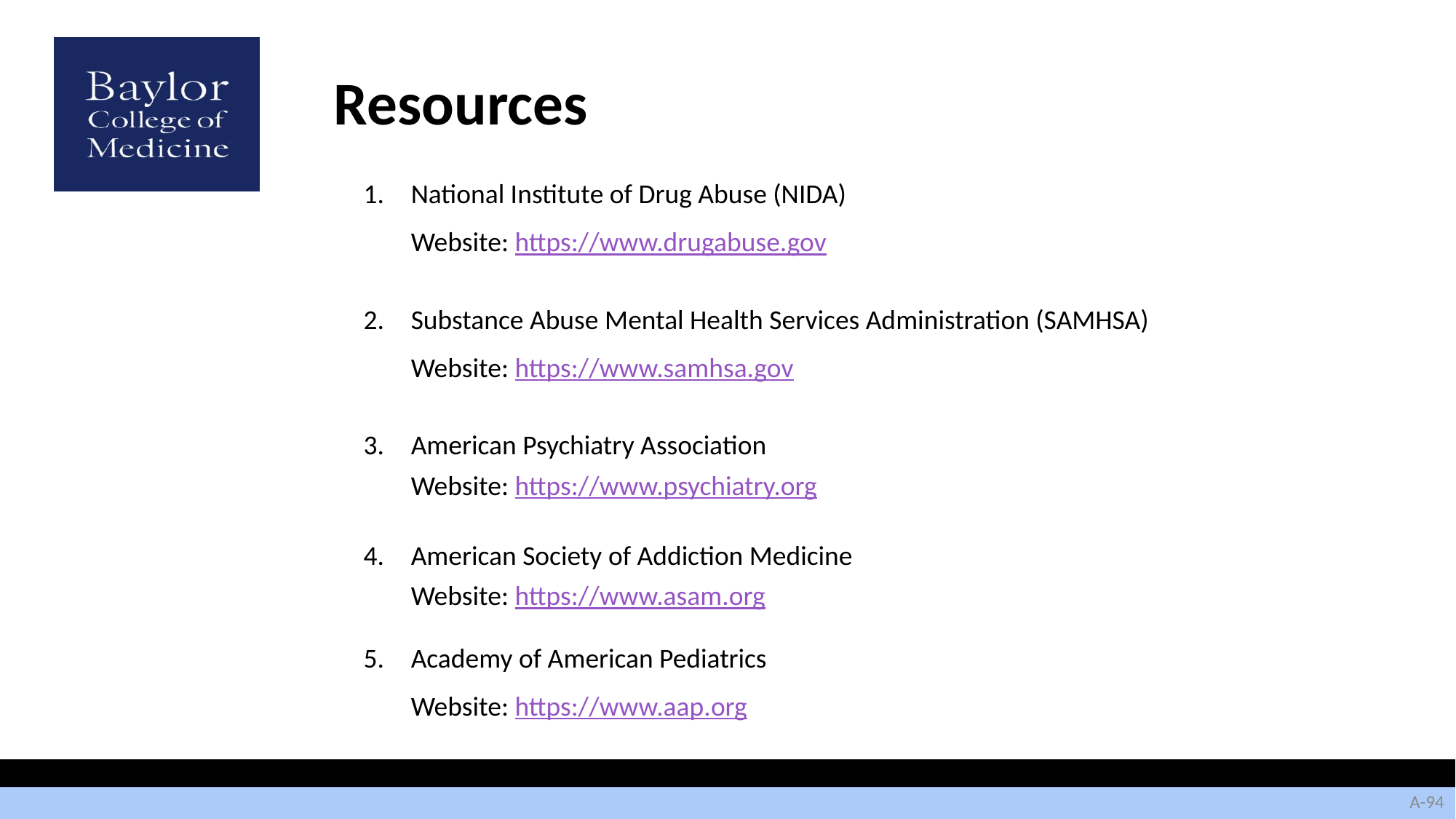

Resources
1. 	National Institute of Drug Abuse (NIDA)
	Website: https://www.drugabuse.gov
Substance Abuse Mental Health Services Administration (SAMHSA)
	Website: https://www.samhsa.gov
3.	American Psychiatry Association
	Website: https://www.psychiatry.org
4. 	American Society of Addiction Medicine
	Website: https://www.asam.org
5. 	Academy of American Pediatrics
	Website: https://www.aap.org
A-94
