# Supplementary material for: Adolescent Addiction Curriculum: Impact on Knowledge Self-Assessment in Pediatric Learners
Source: MedEdPORTAL. 2018 May 7;14:10716. doi: 10.15766/mep_2374-8265.10716 (PMC6342343; doi:10.15766/mep_2374-8265.10716)
Supplement: Supplementary file 1 — A. Addiction Session 1 Lecture Plan.docx B. Addiction Session 1 Instructor Notes.docx C. Addiction Session 1 Slides.pptx D. Addiction Session 1 Self-Assessment.docx E. Addiction Session 2 Lecture Plan.docx F. Addiction Session 2 Instructor Notes.docx G. Addiction Session 2 Slides.pptx H. Addiction Session 2 Self-Assessment.docx I. Addiction Session 2 Worksheets.docx J. Addiction Session 2 Patient Case B.docx K. Addiction Session 3 Lecture Plan.docx L. Addiction Session 3 Instructor Notes.docx M. Addiction Session 3 Slides.pptx N. Addiction Session 3 Self-Assessment.docx [file mep-14-10716-s001.zip › E._Addiction_Session_2_Lecture_Plan.docx]

**Adolescent Addiction Session 2: Lecture Plan**

**Title**: Alcohol, New Emerging Drugs, Adolescence and Addiction

**Description**: Adolescent medicine fellows and rotating pediatric residents attended this lecture as part of routine didactic schedule. This lecture will review adolescent brain development, biopsychosocial factors associated with adolescent addiction, stages of development of adolescent addiction, and co-occurring mental health disorders.

**Learning Objectives**:

By the end of this seminar, the participant will be able to:

1. Describe the Neurobiology of Alcohol Addiction
2. Conceptualize substances along a gradient of harm
3. Describe adolescent brain development and priming for substance use disorders
4. Explain the 4 stages of development of adolescent SUD
5. Identify common co-occurring mental health conditions in adolescent addictive disorders
6. Identify Risk and Protective factors related to Adolescent Addiction
7. Connect the dots . . . . . . . . . . . . . . . .

**Seminar Outline:**

1. Alcohol
2. New Substances (NPS, NED’s)
3. Overview of Substances and Physical Harm
4. Adolescence and Addiction
5. Co-Occurring conditions in Adolescent Addiction
6. Transitions, Trauma and Adolescent Addiction
7. Stages of development of Adolescent Substance Use
8. Risks and Protective Factors
9. Case Examples
10. Summary

**Suggested Reading**:

1. National Institute of Drug Abuse (NIDA)

Website: [https://www.drugabuse.gov](https://www.drugabuse.gov/)

Document: Preventing Drug Use among Children and Adolescents

Link: <https://www.drugabuse.gov/sites/default/files/preventingdruguse_2.pdf> (Accessed April 6, 2018)

2. The National Child Traumatic Stress Network (NCTSN)

Website: [www.nctsn.org](http://www.nctsn.org/)

Document: Trauma and Substance Use

Link: <http://www.nctsn.org/sites/default/files/assets/pdfs/SAToolkit_1.pdf> (Accessed April 6, 2018)

3. Siqueira, L., Smith, V. C., & Committee On Substance, A. (2015). Binge Drinking. *Pediatrics, 136*(3), e718-726. doi:10.1542/peds.2015-2337. Available at <http://pediatrics.aappublications.org/content/pediatrics/136/3/e718.full.pdf> (Accessed April 6, 2018)

4. Weitzman, C., Wegner, L., Section on Developmental and Behavioral Pediatrics, Committee on Psychosocial aspects of Child and Family Health, Council on Early Childhood, and Society for Developmental and Behavioral Pediatrics. American Academy of, P. (2015). Promoting optimal development: screening for behavioral and emotional problems. Clinical Report. Guidance for the Clinician in Rendering Pediatric Care. *Pediatrics, 135*(2), 384-395. doi:10.1542/peds.2014-371. Available at <http://pediatrics.aappublications.org/content/pediatrics/135/2/384.full.pdf> (Accessed April 6, 2018)

5. Garner, A. S., Shonkoff, J. P., Committee on Psychosocial Aspects of Child and Family Health, Committee on Early Childhood, Adoption, and Dependent Care, and Section on Developmental and Behavioral Pediatrics. Early childhood adversity, toxic stress, and the role of the pediatrician: translating developmental science into lifelong health. *Pediatrics, 129*(1), e224-231. doi:10.1542/peds.2011-2662. Available at <http://pediatrics.aappublications.org/content/pediatrics/129/1/e224.full.pdf> (Accessed April 6, 2018)
