# Supplementary material for: Adolescent Addiction Curriculum: Impact on Knowledge Self-Assessment in Pediatric Learners
Source: MedEdPORTAL. 2018 May 7;14:10716. doi: 10.15766/mep_2374-8265.10716 (PMC6342343; doi:10.15766/mep_2374-8265.10716)
Supplement: Supplementary file 1 — A. Addiction Session 1 Lecture Plan.docx B. Addiction Session 1 Instructor Notes.docx C. Addiction Session 1 Slides.pptx D. Addiction Session 1 Self-Assessment.docx E. Addiction Session 2 Lecture Plan.docx F. Addiction Session 2 Instructor Notes.docx G. Addiction Session 2 Slides.pptx H. Addiction Session 2 Self-Assessment.docx I. Addiction Session 2 Worksheets.docx J. Addiction Session 2 Patient Case B.docx K. Addiction Session 3 Lecture Plan.docx L. Addiction Session 3 Instructor Notes.docx M. Addiction Session 3 Slides.pptx N. Addiction Session 3 Self-Assessment.docx [file mep-14-10716-s001.zip › F._Addiction_Session_2_Instructor_Notes.docx]

**Adolescent Addiction Session 2 Instructor Notes**

**Learner Objectives, Activities, Notes to the Educator, and Corresponding PowerPoint Lecture Slides**

Session Title: Alcohol, New Emerging Drugs, Adolescence and Addiction

Learning Goal: To provide knowledge of adolescence with addiction within a developmental framework

Please note: alcohol and new emerging drugs are included in this session due to limited time for presentation in Session 1

|  | **Learner Objective** | **Learner Activity** | **Educator Action** | **PowerPoint Lecture Slides** |
| --- | --- | --- | --- | --- |
|  | Revise the neurobiology of alcohol | Optional Activity:  Watch YouTube video  Title: The Effects of Alcohol on the Brain   - Source: [Alcohol Challenge](https://www.youtube.com/channel/UCd7nMuTfcnnmaIl17Ru7mbg). Published Feb 22, 2016 - Running time: 8:01mins - Website: https://www.youtube.com/watch?v=-fBG8xcZoF4 | Discuss the neurobiology of alcohol.  May include genetics and epigenetics of alcohol. | B5 to B9 |
|  | Summarize alcohol use in adolescents |  | Discuss the various aspects of alcohol use in adolescents | B10 to 22 |
|  | Recognize New Psychoactive substances and Emerging Drugs |  |  | B23 |
|  | Obtain an overview of Substances and Physical Harm | Ask what the learners consider to be the more dangerous drugs. | Highlight how becoming addicted to one substance increases the risk of becoming addicted to other substances | B24 |
|  | BREAK | BREAK | BREAK | BREAK |
|  | Recognize Adolescence as a unique developmental period | Watch video titled ‘Life’s complicated enough’ (Running time: 1:13mins; Slide B29) | Divide into early, middle, late adolescence and include young adulthood  Discuss adolescence according to the biological, psychological, and social factors at play during the different stages of adolescence | B29-B34 |
|  | Outline factors which increase the incidence of drug use initiation in adolescents |  | Discuss Adolescence as prime time for substance use | B35-39 |
|  | Discuss Adolescence and reward-seeking |  | Discuss heightened reward-seeking behavior and impulsivity in adolescence | B40 |
|  | Recognize that age of onset of Adolescent substance use directly impacts their developmental trajectory and the severity of their addictive disorder. |  | Early onset of drug use is associated with worse outcomes | B41 - B46 |
|  | Relate mental health conditions to adolescent addictive behaviors including substance use and vice versa. | Ask learners which conditions they think (or know) are associated with adolescent addiction | Review comorbid mental health conditions | B47 - B50 |
|  | Identify factors related to transitions and trauma and relate this to adolescent addiction |  | Include transitions from elementary, middle, high school and college as particularly important times for increased stress and initiation of drug use. | B51 – B52 |
|  | Relate adolescent drug use to fetal drug exposure |  | May mention that up to 25% of adolescents with STDs report using a substance before intercourse | B53 |
|  | BREAK | BREAK | BREAK | BREAK |
|  | Discuss the stages of Adolescent Substance Use |  | For each teen, there may be an important differentiating step between experimentation and continued use. | B56 – B57 |
|  | Explain what Risk and Protective Factors are |  | Explain that:   1. Risk factors are factors (e.g. people, places or things) that increase inclination for towards engaging in addictive behavior(s) 2. Protective Factors are factors (e.g. people, places, or things) that decrease inclination towards engaging in addictive behavior(s) | B58- B60 |
|  | Identify risk and protective factors using the socio-ecologic model |  | Discuss this in along concentric circles, with the central factors (individual and relational) conferring the highest (and therefore more readily changeable) risk | B61 - B65 |
|  | Complete a connect the dot exercise with the goal of identifying transitions, delineating the process of addiction, identifying the impact of treatment (including of depression) resulting in abstinence and return to pre-morbid functional baseline. | Case Discussions utilizing worksheets (Appendix I) and patient case B (Appendix J) | Case discussions should:   1. Focus on reasoning backward to the antecedents of the manifestations of the addictive disorder. 2. Identify transitions in the adolescent’s path. 3. Identify potential points of intervention along the trajectory of development of the disorder. 4. Highlight the need to consult with and trouble-shoot problems with mental health providers involved in patient’s care. 5. Emphasize the importance of continued collaborative care between all providers involved in patient’s care. | Case A: B67 -B70  Case B: B71 – B80 |
|  | Formulate Take Home Points |  | Review summary slide  At the end of this session, it is helpful to emphasize to the adolescent provider the importance of anticipating and/or identifying developmentally appropriate difficulties in adjusting to transitions. Additionally, it is helpful to encourage providers to inquire about psychological and physical trauma in their patients. | B81 |
|  | Take Questions |  |  | B82 |
|  | Provide Resources |  |  | B83 |
