# Supplementary material for: Adolescent Addiction Curriculum: Impact on Knowledge Self-Assessment in Pediatric Learners
Source: MedEdPORTAL. 2018 May 7;14:10716. doi: 10.15766/mep_2374-8265.10716 (PMC6342343; doi:10.15766/mep_2374-8265.10716)
Supplement: Supplementary file 1 — A. Addiction Session 1 Lecture Plan.docx B. Addiction Session 1 Instructor Notes.docx C. Addiction Session 1 Slides.pptx D. Addiction Session 1 Self-Assessment.docx E. Addiction Session 2 Lecture Plan.docx F. Addiction Session 2 Instructor Notes.docx G. Addiction Session 2 Slides.pptx H. Addiction Session 2 Self-Assessment.docx I. Addiction Session 2 Worksheets.docx J. Addiction Session 2 Patient Case B.docx K. Addiction Session 3 Lecture Plan.docx L. Addiction Session 3 Instructor Notes.docx M. Addiction Session 3 Slides.pptx N. Addiction Session 3 Self-Assessment.docx [file mep-14-10716-s001.zip › G._Addiction_Session_2_Slides.pptx]

## Slide 1
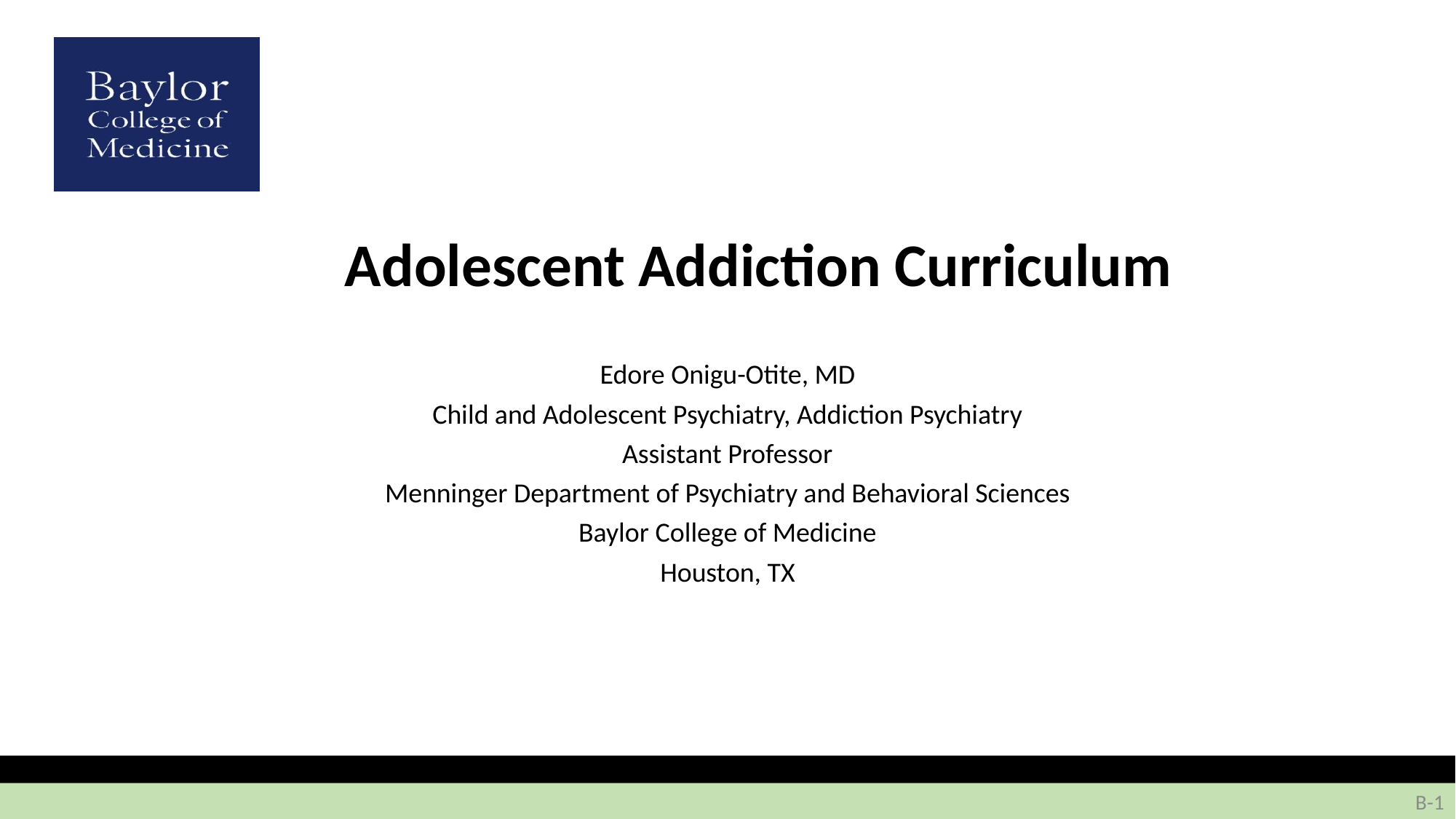

Adolescent Addiction Curriculum
Edore Onigu-Otite, MD
Child and Adolescent Psychiatry, Addiction Psychiatry
Assistant Professor
Menninger Department of Psychiatry and Behavioral Sciences
Baylor College of Medicine
Houston, TX
B-1

## Slide 2
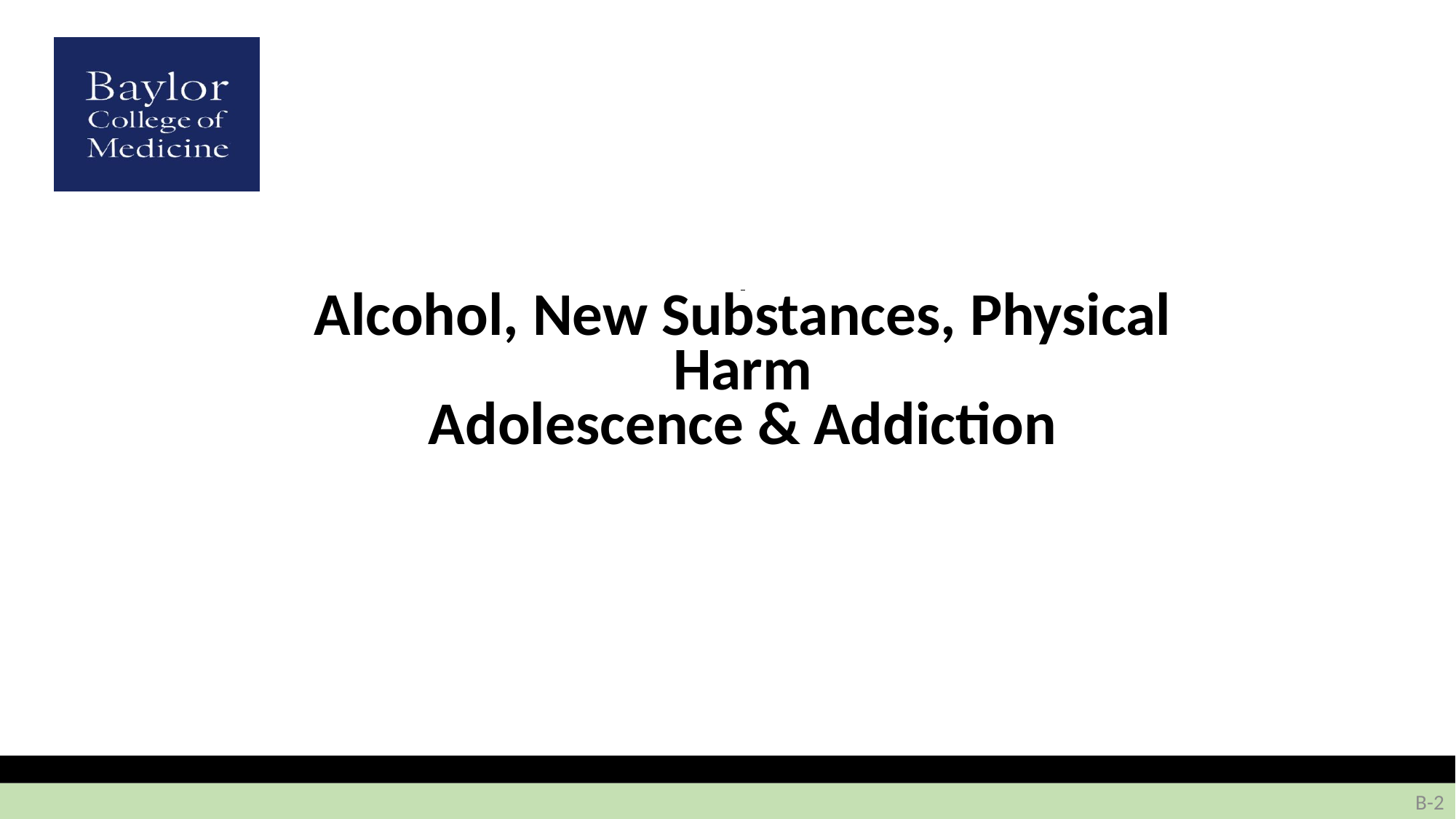

Alcohol, New Substances, Physical Harm
Adolescence & Addiction
B-2

## Slide 3
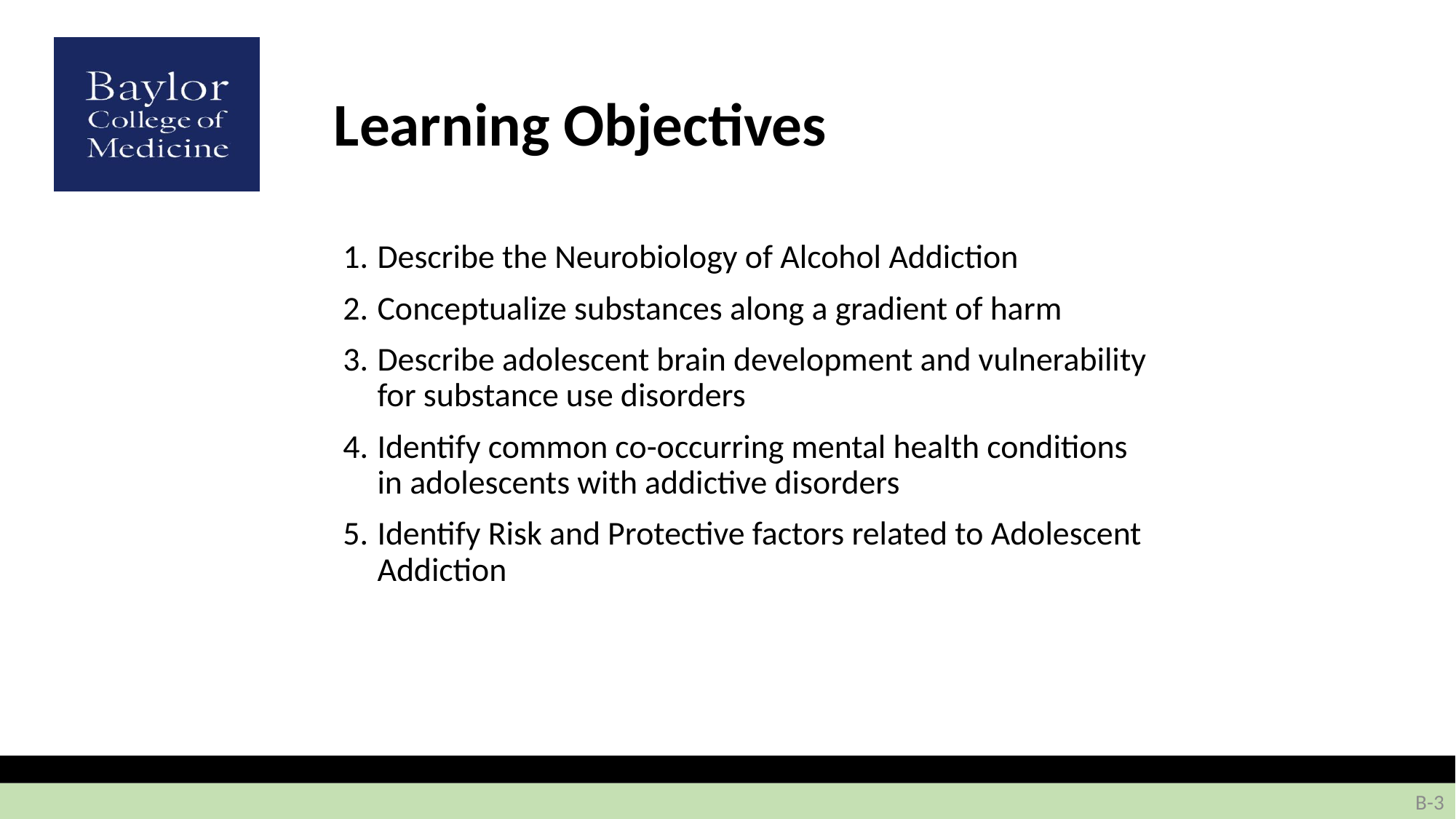

Learning Objectives
1.	Describe the Neurobiology of Alcohol Addiction
2.	Conceptualize substances along a gradient of harm
3.	Describe adolescent brain development and vulnerability for substance use disorders
4. 	Identify common co-occurring mental health conditions in adolescents with addictive disorders
5.	Identify Risk and Protective factors related to Adolescent Addiction
B-3

## Slide 4
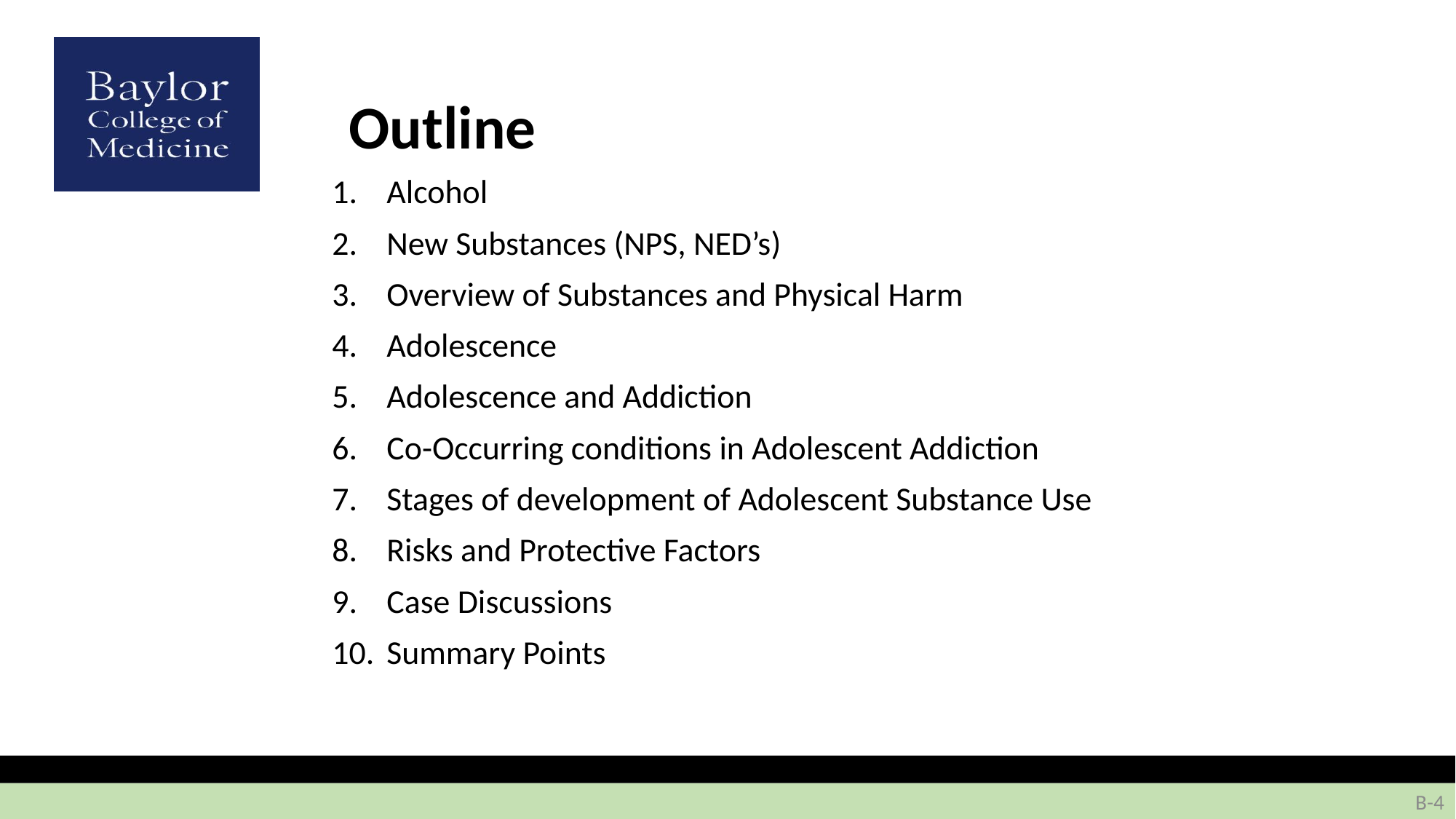

Outline
Alcohol
New Substances (NPS, NED’s)
Overview of Substances and Physical Harm
Adolescence
Adolescence and Addiction
Co-Occurring conditions in Adolescent Addiction
Stages of development of Adolescent Substance Use
Risks and Protective Factors
Case Discussions
Summary Points
B-4

## Slide 5
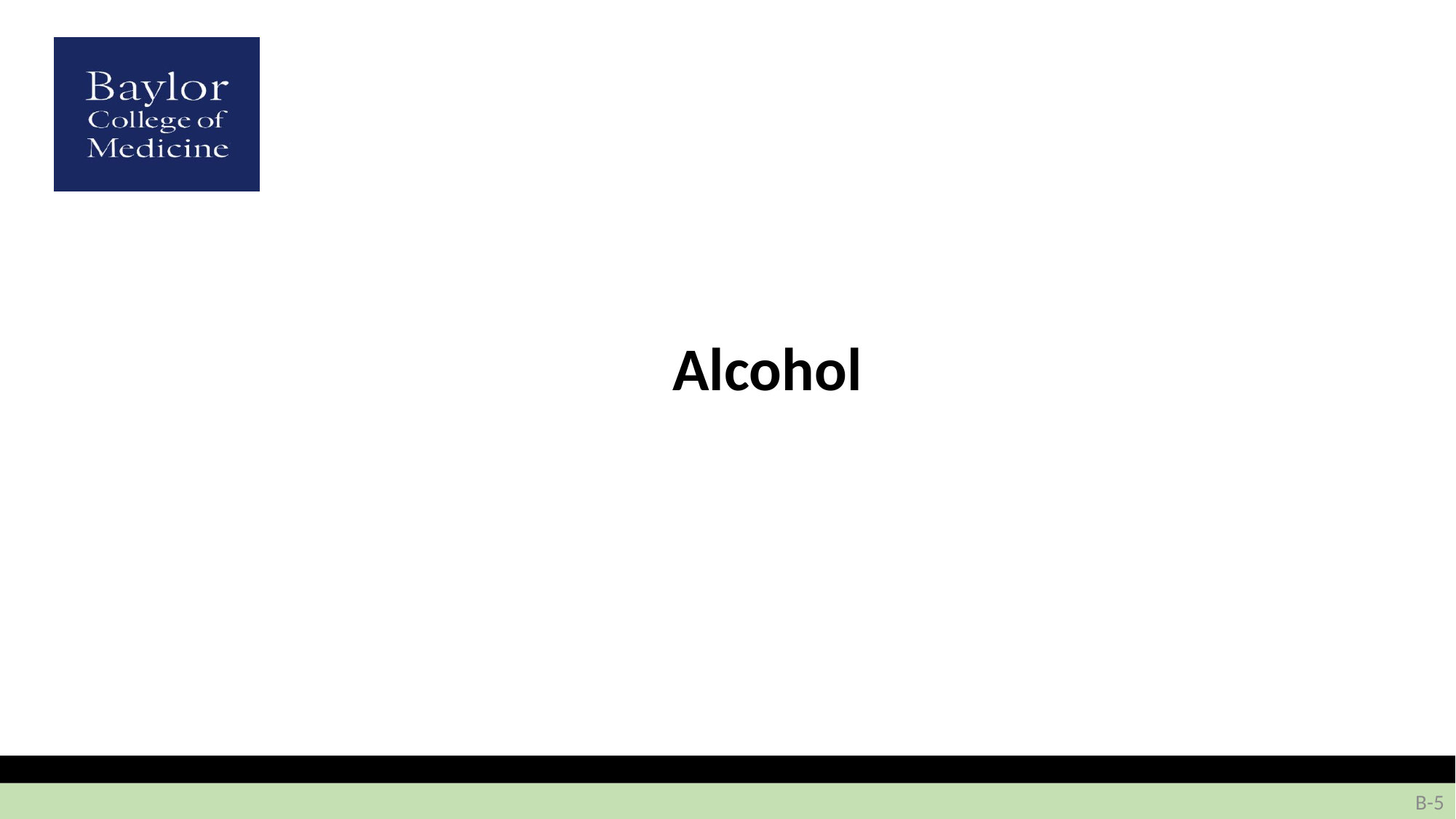

Alcohol
B-5

## Slide 6
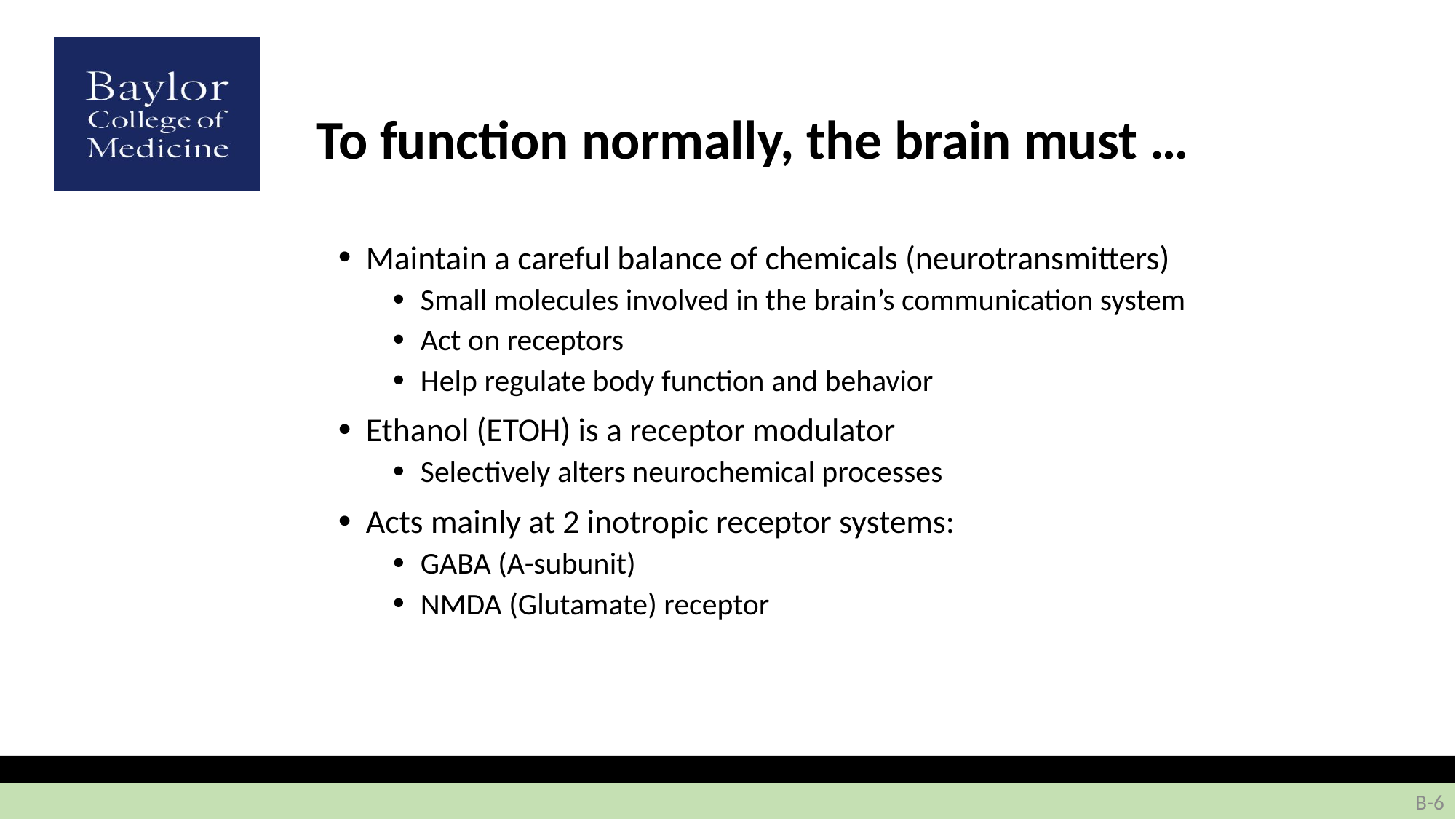

To function normally, the brain must …
Maintain a careful balance of chemicals (neurotransmitters)
Small molecules involved in the brain’s communication system
Act on receptors
Help regulate body function and behavior
Ethanol (ETOH) is a receptor modulator
Selectively alters neurochemical processes
Acts mainly at 2 inotropic receptor systems:
GABA (A-subunit)
NMDA (Glutamate) receptor
B-6

## Slide 7
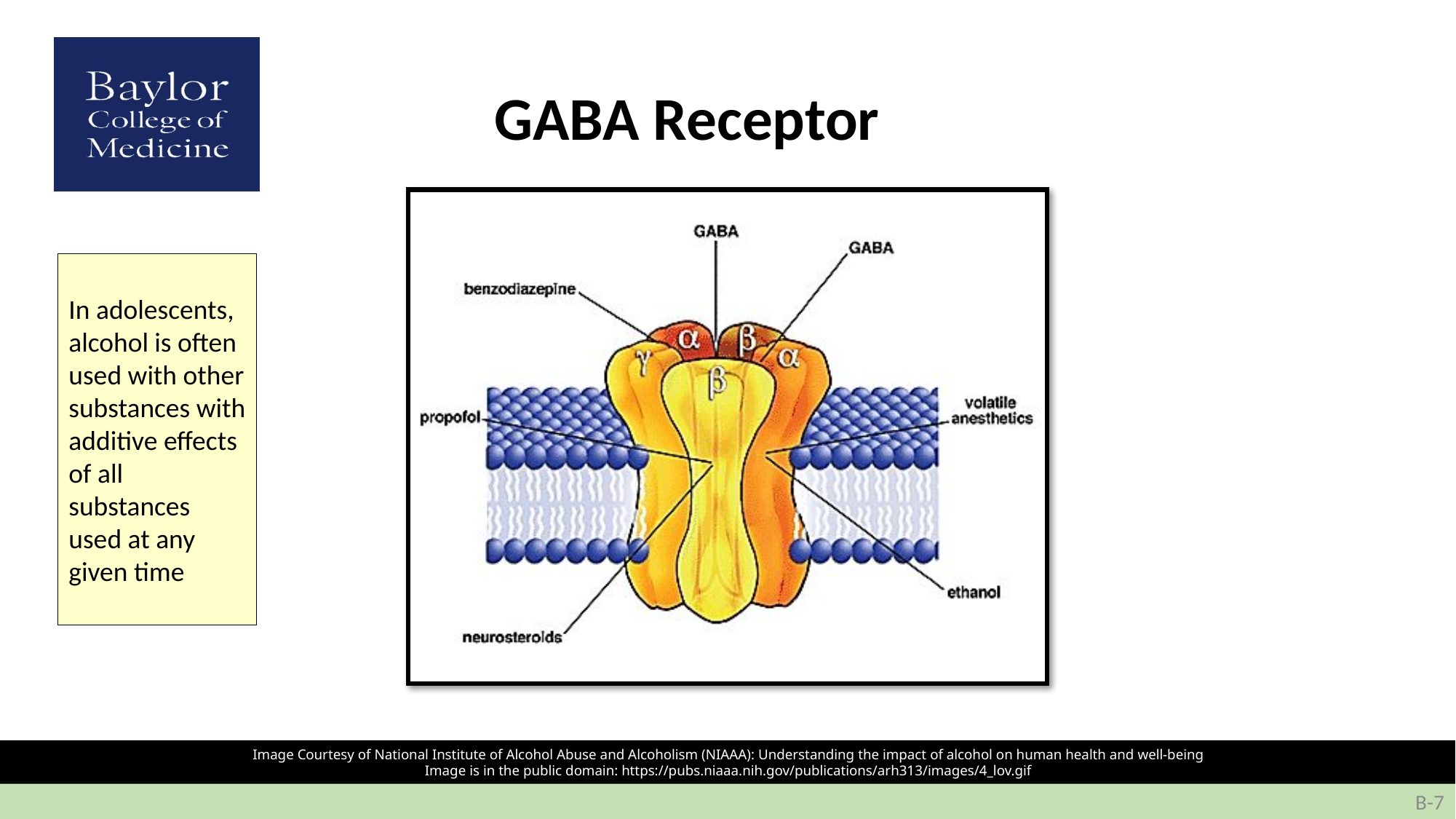

GABA Receptor
In adolescents, alcohol is often used with other substances with additive effects of all substances used at any given time
Image Courtesy of National Institute of Alcohol Abuse and Alcoholism (NIAAA): Understanding the impact of alcohol on human health and well-being
Image is in the public domain: https://pubs.niaaa.nih.gov/publications/arh313/images/4_lov.gif
B-7

## Slide 8
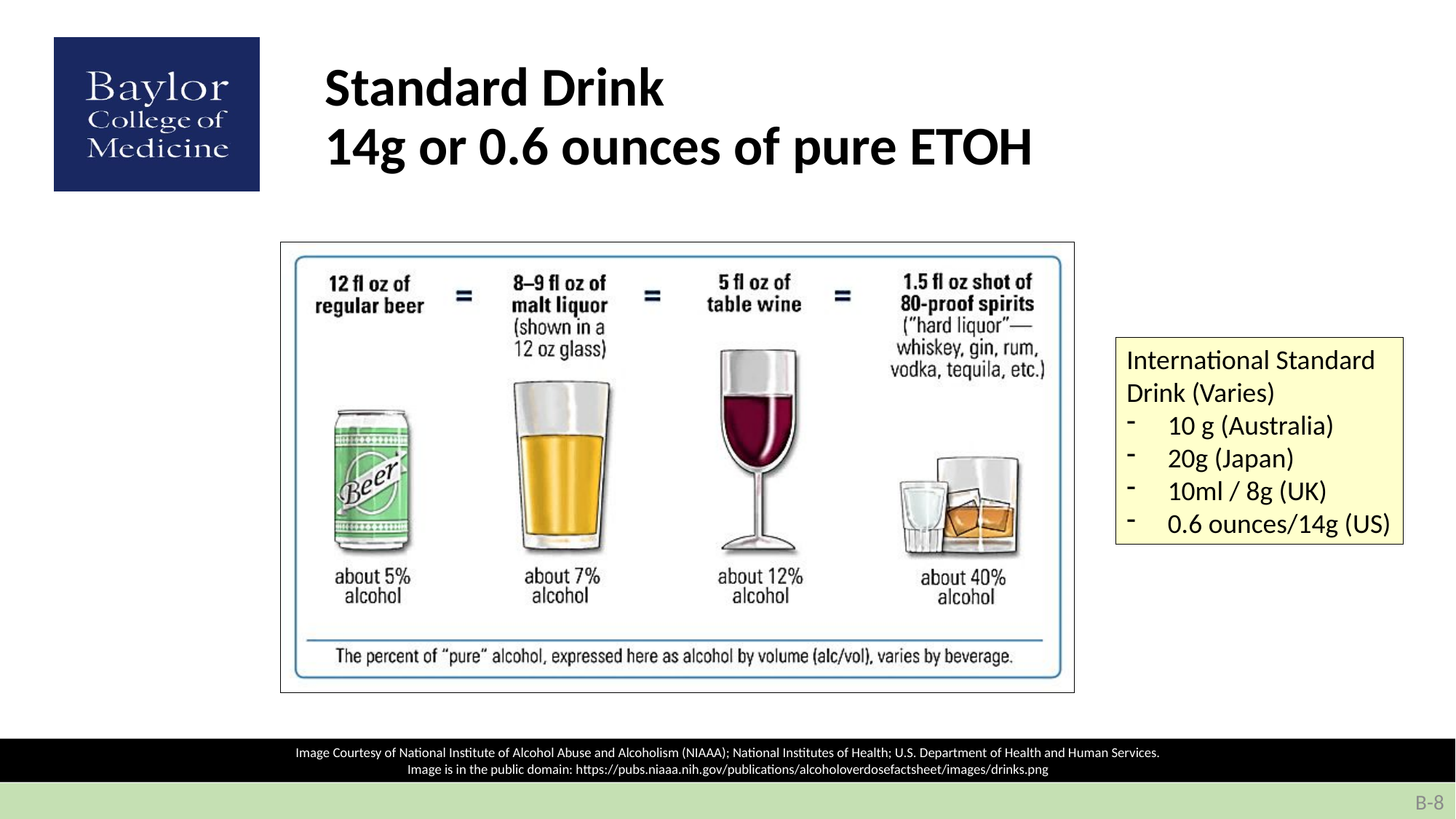

Standard Drink
14g or 0.6 ounces of pure ETOH
International Standard Drink (Varies)
10 g (Australia)
20g (Japan)
10ml / 8g (UK)
0.6 ounces/14g (US)
Image Courtesy of National Institute of Alcohol Abuse and Alcoholism (NIAAA); National Institutes of Health; U.S. Department of Health and Human Services.
Image is in the public domain: https://pubs.niaaa.nih.gov/publications/alcoholoverdosefactsheet/images/drinks.png
B-8

## Slide 9
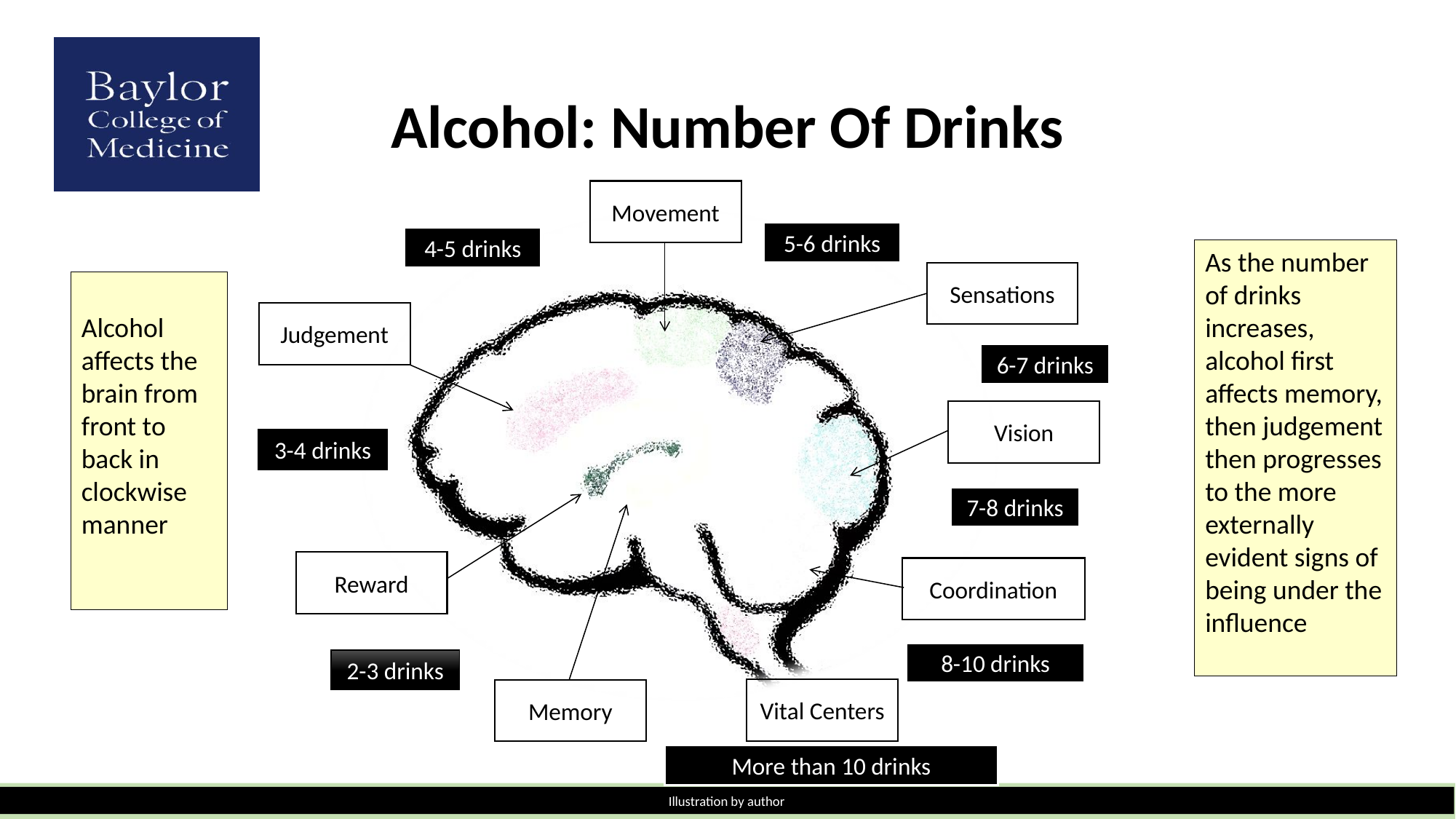

Alcohol: Number Of Drinks
Movement
5-6 drinks
4-5 drinks
As the number of drinks increases, alcohol first affects memory, then judgement then progresses to the more externally evident signs of being under the influence
Sensations
Alcohol affects the brain from front to back in clockwise manner
Judgement
6-7 drinks
Vision
3-4 drinks
7-8 drinks
Reward
Coordination
8-10 drinks
2-3 drinks
Vital Centers
Memory
More than 10 drinks
B-9
Illustration by author

## Slide 10
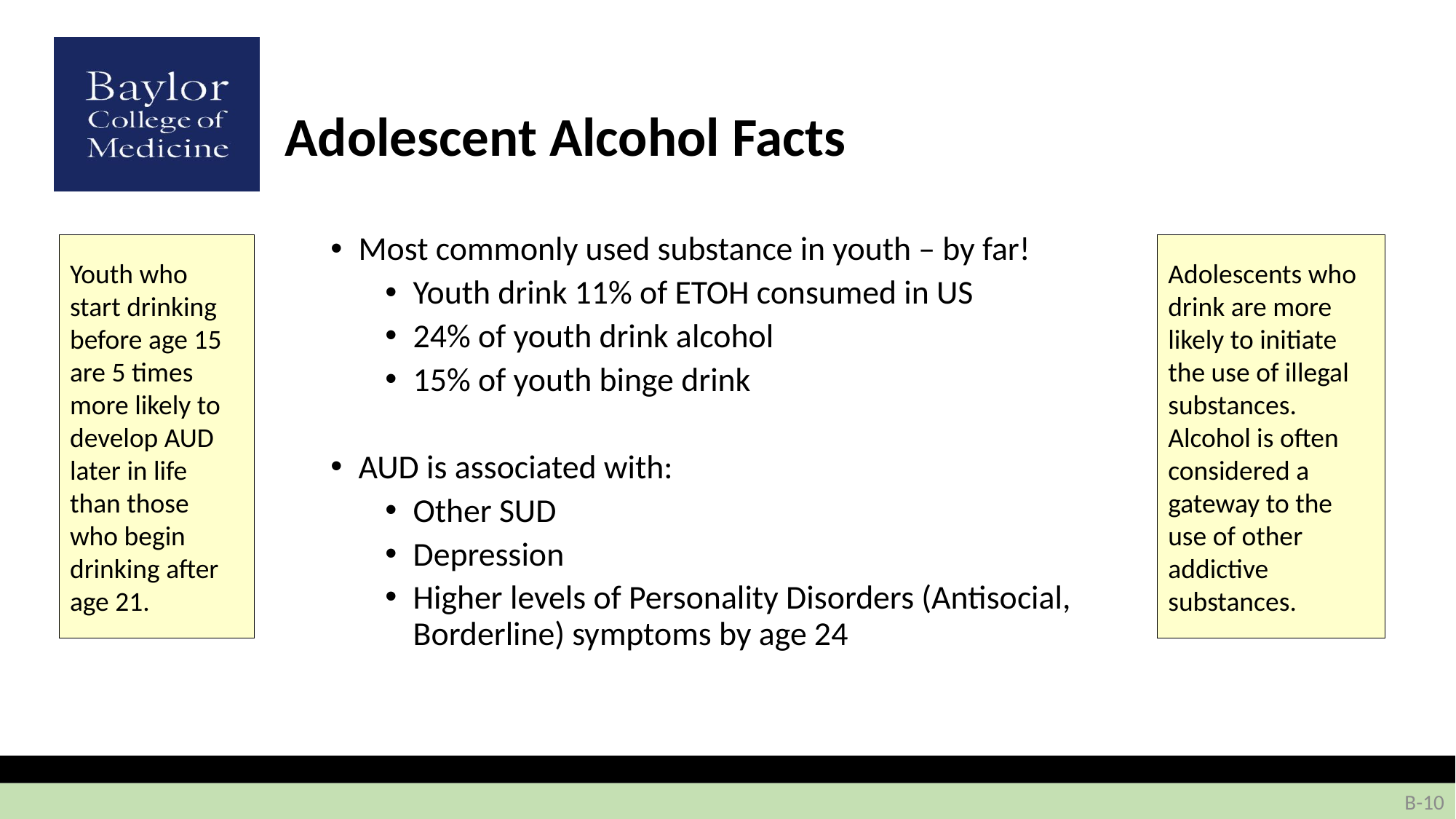

Adolescent Alcohol Facts
Most commonly used substance in youth – by far!
Youth drink 11% of ETOH consumed in US
24% of youth drink alcohol
15% of youth binge drink
AUD is associated with:
Other SUD
Depression
Higher levels of Personality Disorders (Antisocial, Borderline) symptoms by age 24
Youth who start drinking before age 15 are 5 times more likely to develop AUD later in life than those who begin drinking after age 21.
Adolescents who drink are more likely to initiate the use of illegal substances. Alcohol is often considered a gateway to the use of other addictive substances.
B-10

## Slide 11
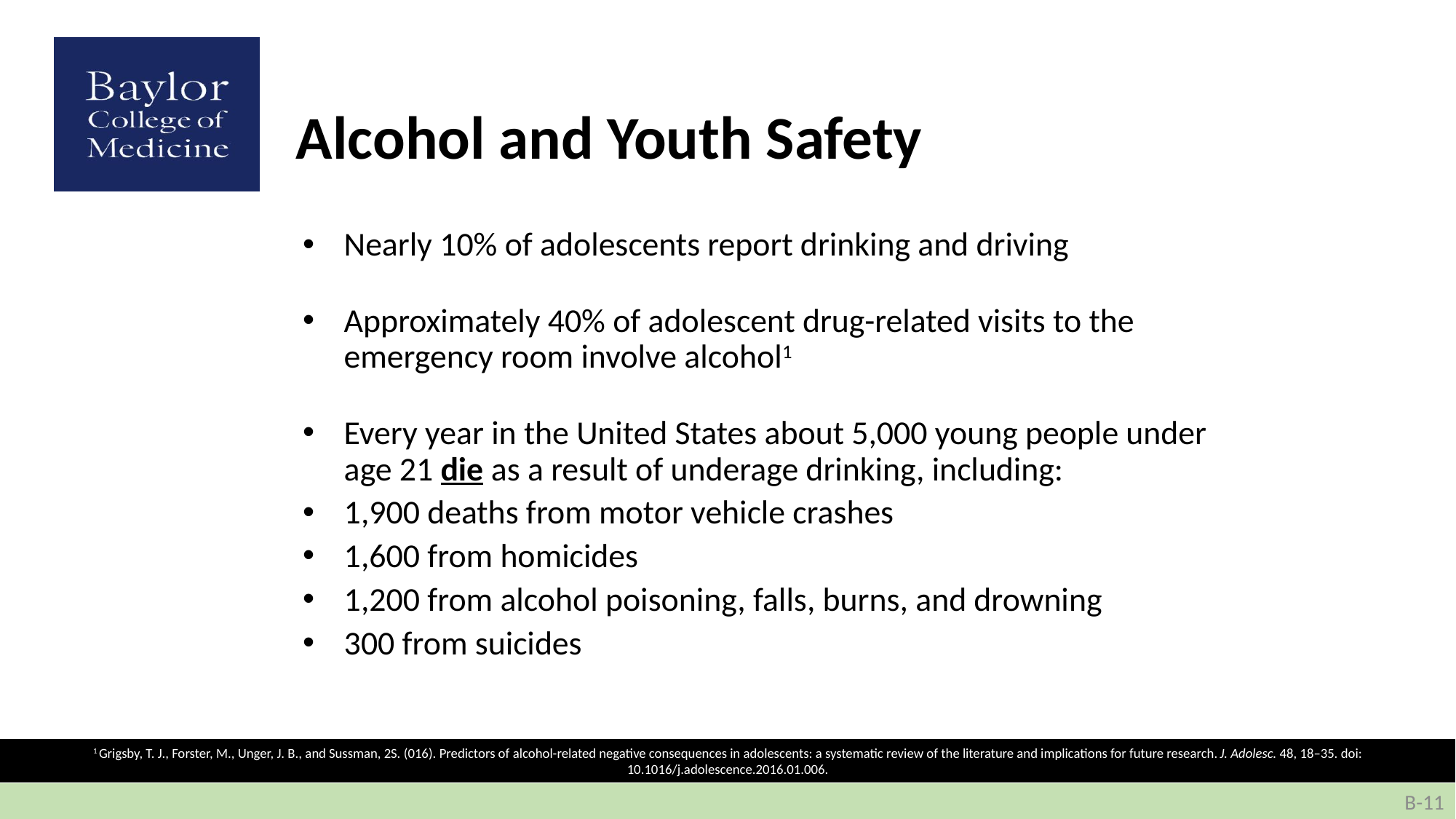

Alcohol and Youth Safety
Nearly 10% of adolescents report drinking and driving
Approximately 40% of adolescent drug-related visits to the emergency room involve alcohol1
Every year in the United States about 5,000 young people under age 21 die as a result of underage drinking, including:
1,900 deaths from motor vehicle crashes
1,600 from homicides
1,200 from alcohol poisoning, falls, burns, and drowning
300 from suicides
1 Grigsby, T. J., Forster, M., Unger, J. B., and Sussman, 2S. (016). Predictors of alcohol-related negative consequences in adolescents: a systematic review of the literature and implications for future research. J. Adolesc. 48, 18–35. doi: 10.1016/j.adolescence.2016.01.006.
B-11

## Slide 12
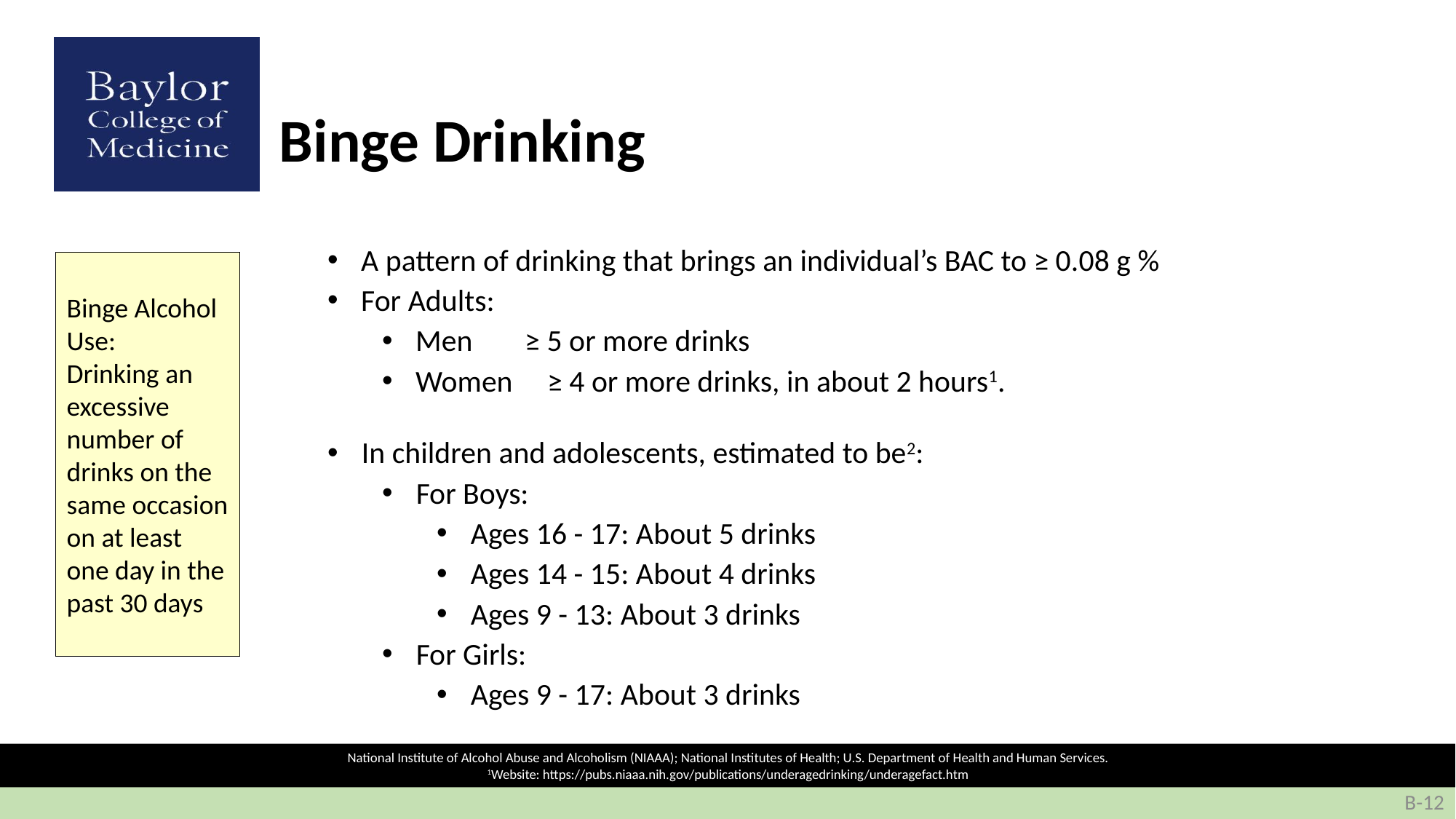

Binge Drinking
A pattern of drinking that brings an individual’s BAC to ≥ 0.08 g %
For Adults:
Men 	≥ 5 or more drinks
Women ≥ 4 or more drinks, in about 2 hours1.
In children and adolescents, estimated to be2:
For Boys:
Ages 16 - 17: About 5 drinks
Ages 14 - 15: About 4 drinks
Ages 9 - 13: About 3 drinks
For Girls:
Ages 9 - 17: About 3 drinks
Binge Alcohol Use:
Drinking an excessive number of drinks on the same occasion on at least one day in the past 30 days
National Institute of Alcohol Abuse and Alcoholism (NIAAA); National Institutes of Health; U.S. Department of Health and Human Services.
1Website: https://pubs.niaaa.nih.gov/publications/underagedrinking/underagefact.htm
B-12

## Slide 13
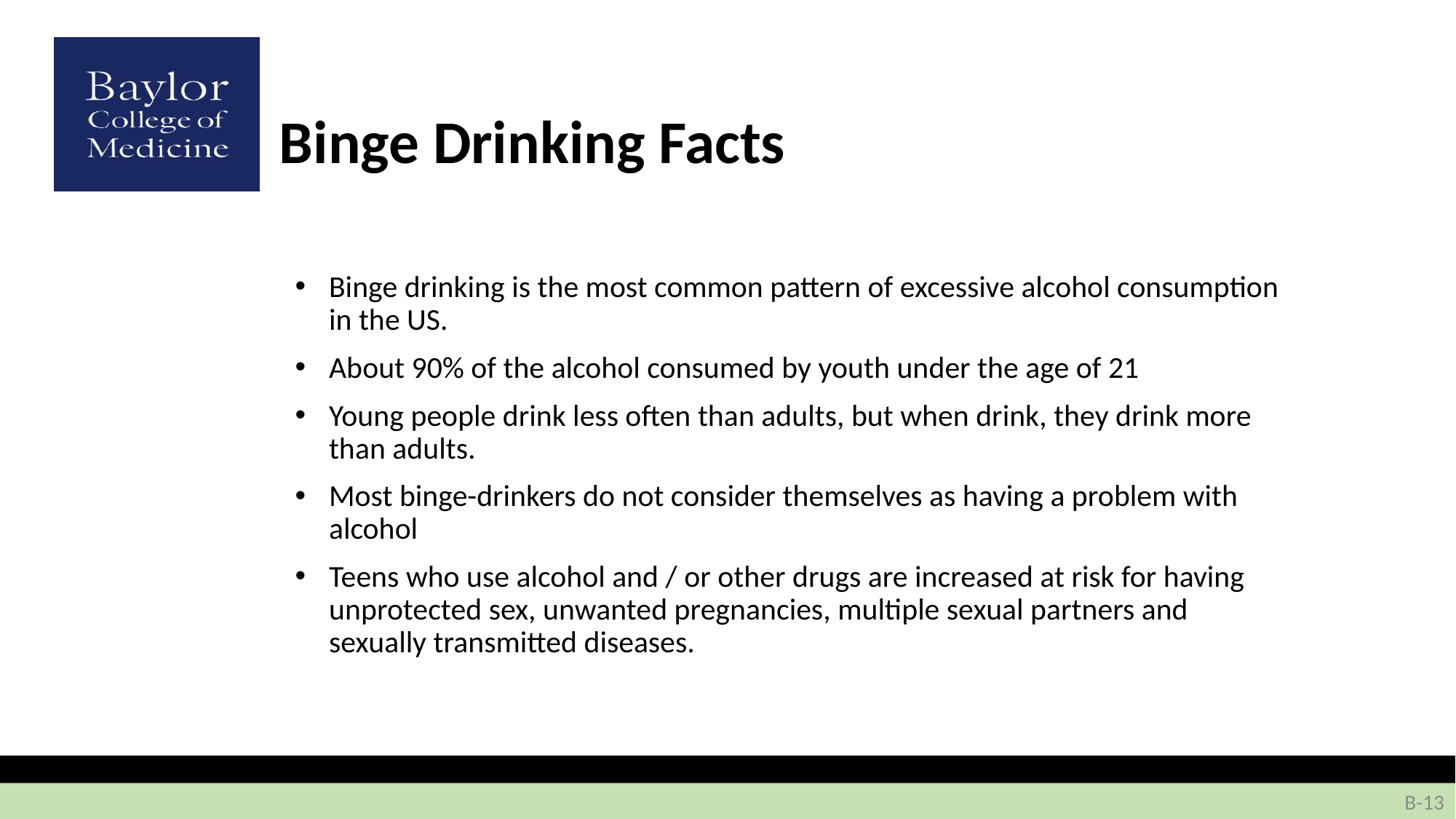

Binge Drinking Facts
Binge drinking is the most common pattern of excessive alcohol consumption in the US.
About 90% of the alcohol consumed by youth under the age of 21
Young people drink less often than adults, but when drink, they drink more than adults.
Most binge-drinkers do not consider themselves as having a problem with alcohol
Teens who use alcohol and / or other drugs are increased at risk for having unprotected sex, unwanted pregnancies, multiple sexual partners and sexually transmitted diseases.
B-13

## Slide 14
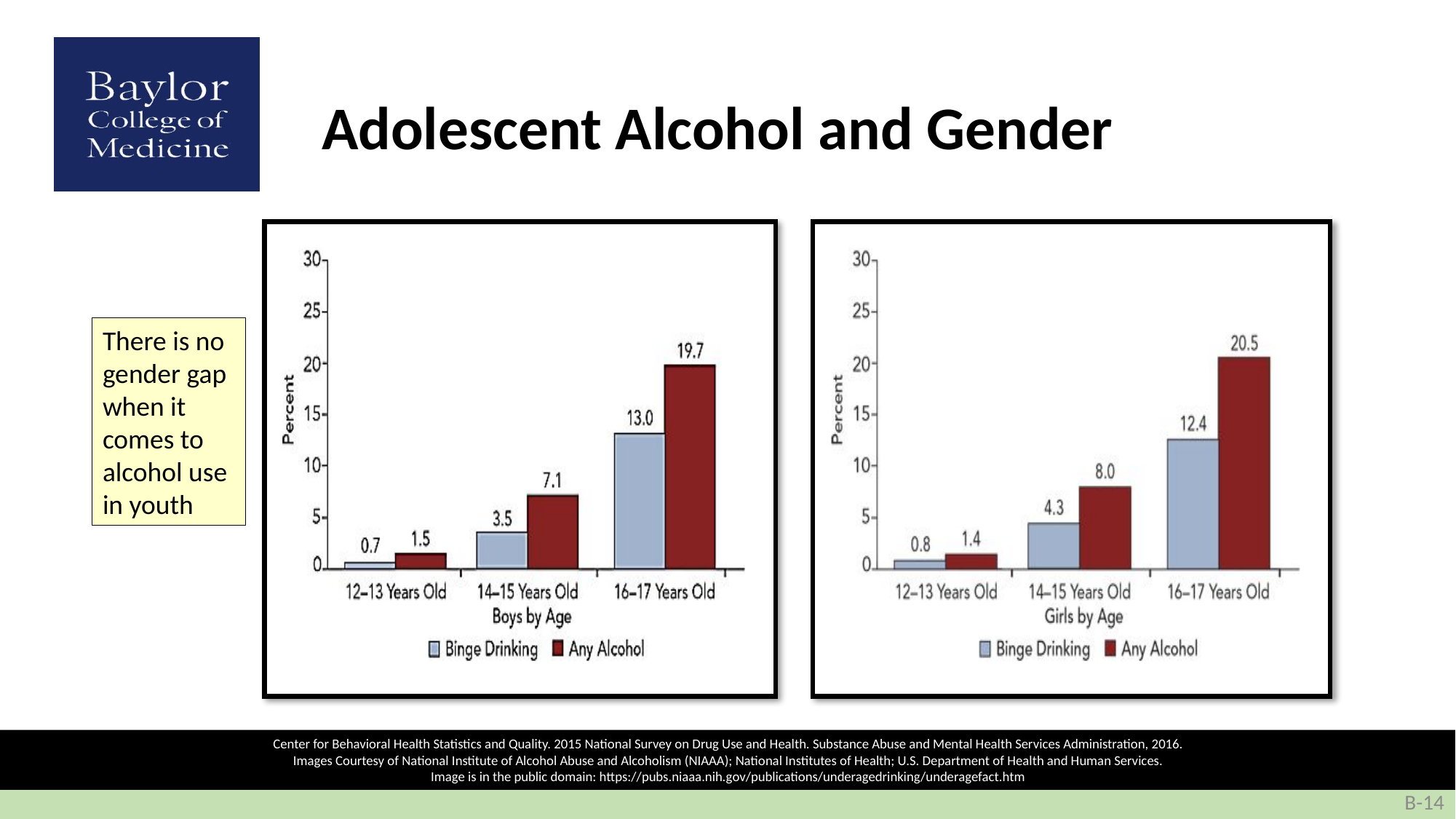

Adolescent Alcohol and Gender
There is no gender gap when it comes to alcohol use in youth
Center for Behavioral Health Statistics and Quality. 2015 National Survey on Drug Use and Health. Substance Abuse and Mental Health Services Administration, 2016.
Images Courtesy of National Institute of Alcohol Abuse and Alcoholism (NIAAA); National Institutes of Health; U.S. Department of Health and Human Services.
Image is in the public domain: https://pubs.niaaa.nih.gov/publications/underagedrinking/underagefact.htm
B-14

## Slide 15
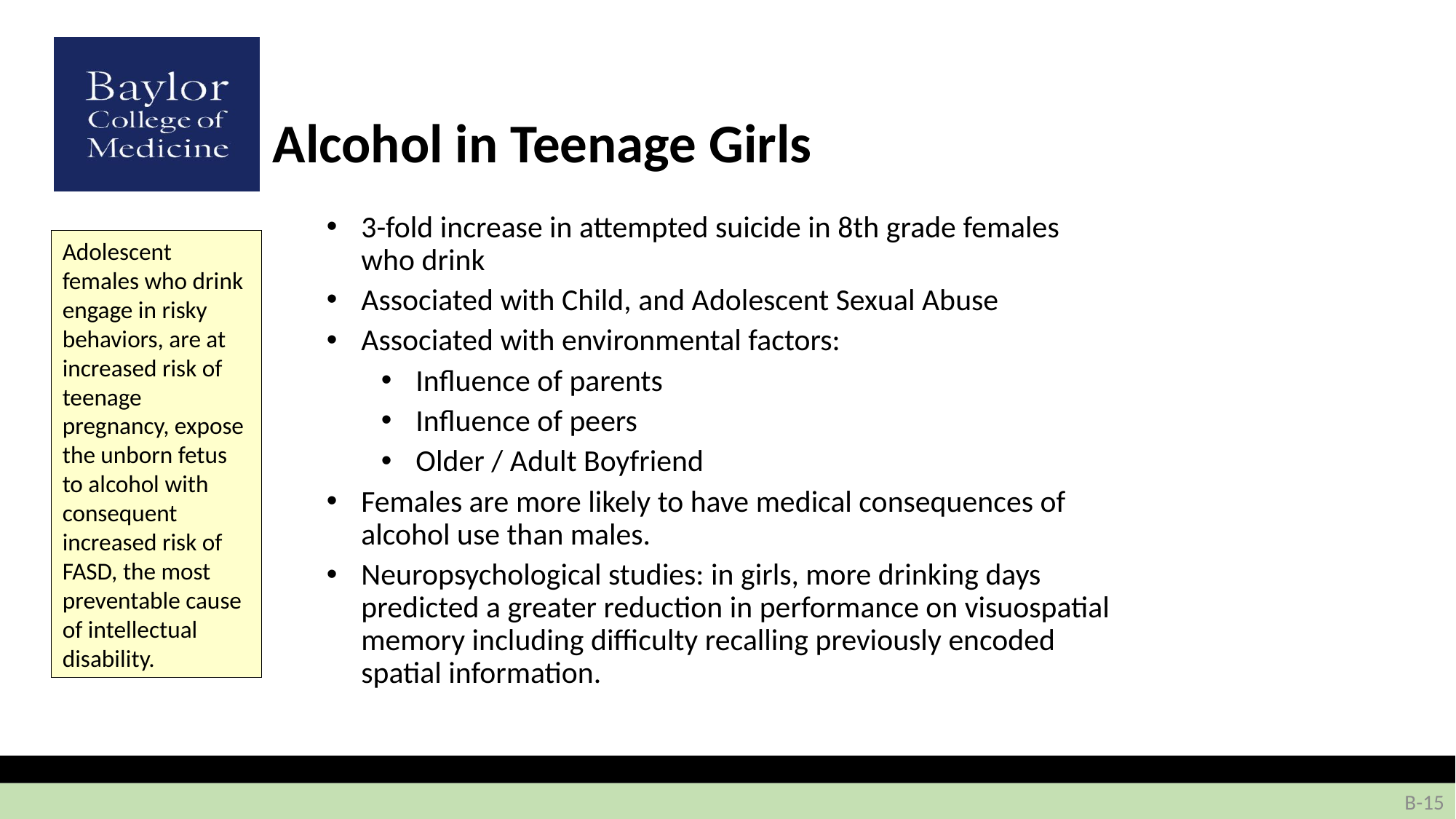

Alcohol in Teenage Girls
3-fold increase in attempted suicide in 8th grade females who drink
Associated with Child, and Adolescent Sexual Abuse
Associated with environmental factors:
Influence of parents
Influence of peers
Older / Adult Boyfriend
Females are more likely to have medical consequences of alcohol use than males.
Neuropsychological studies: in girls, more drinking days predicted a greater reduction in performance on visuospatial memory including difficulty recalling previously encoded spatial information.
Adolescent females who drink engage in risky behaviors, are at increased risk of teenage pregnancy, expose the unborn fetus to alcohol with consequent increased risk of FASD, the most preventable cause of intellectual disability.
B-15

## Slide 16
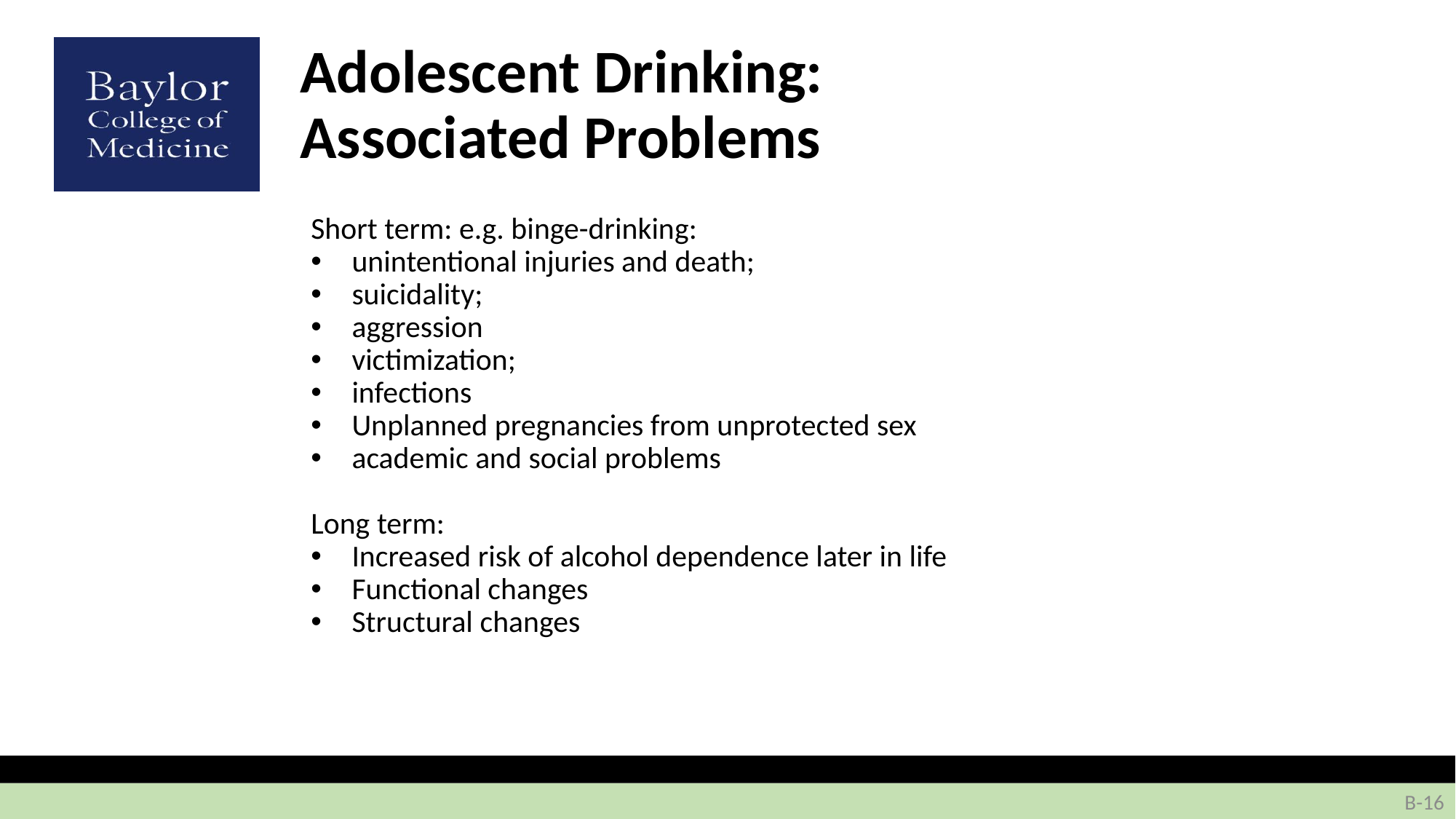

Adolescent Drinking:
Associated Problems
Short term: e.g. binge-drinking:
unintentional injuries and death;
suicidality;
aggression
victimization;
infections
Unplanned pregnancies from unprotected sex
academic and social problems
Long term:
Increased risk of alcohol dependence later in life
Functional changes
Structural changes
B-16

## Slide 17
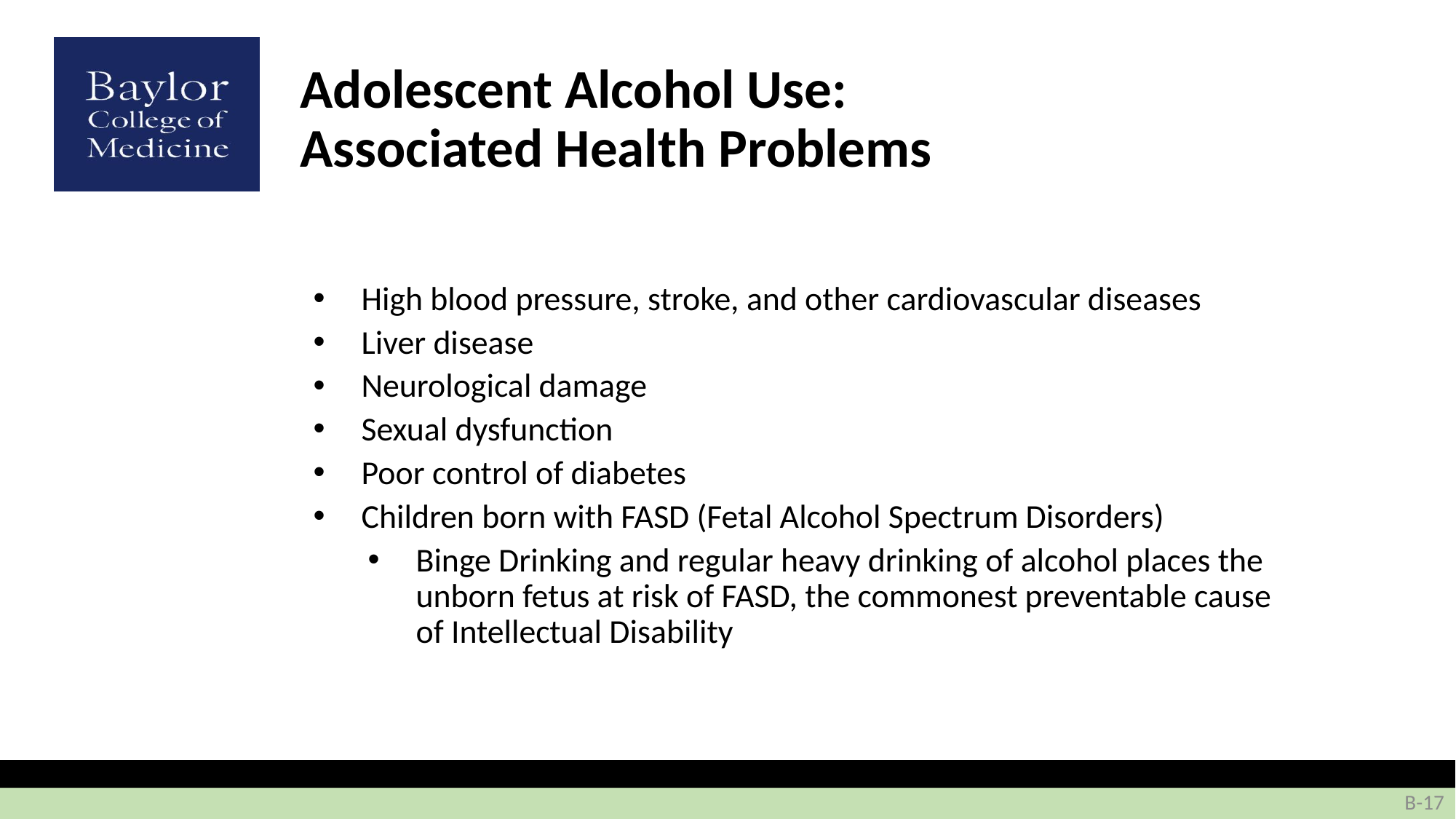

Adolescent Alcohol Use:
Associated Health Problems
High blood pressure, stroke, and other cardiovascular diseases
Liver disease
Neurological damage
Sexual dysfunction
Poor control of diabetes
Children born with FASD (Fetal Alcohol Spectrum Disorders)
Binge Drinking and regular heavy drinking of alcohol places the unborn fetus at risk of FASD, the commonest preventable cause of Intellectual Disability
B-17

## Slide 18
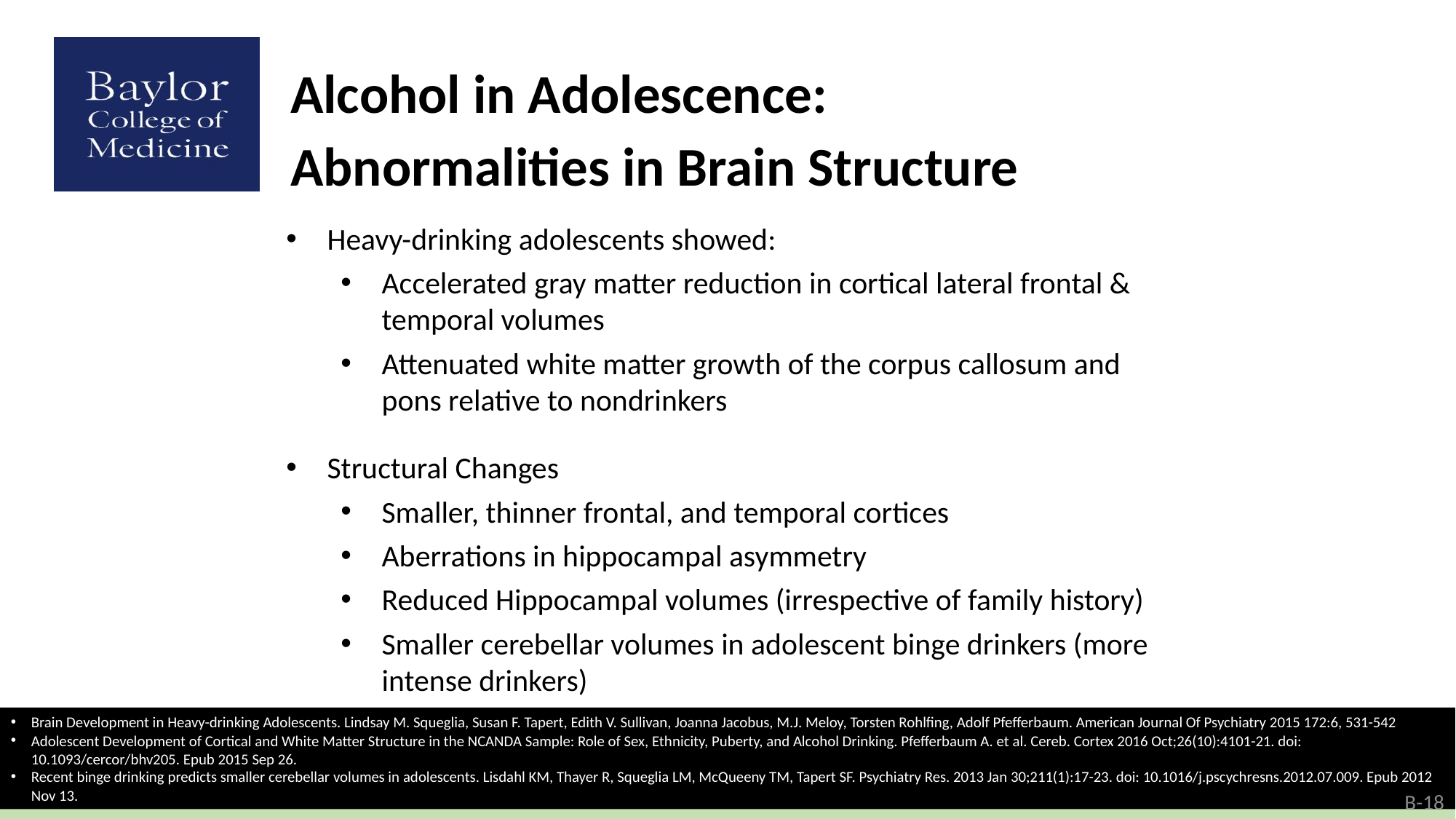

Alcohol in Adolescence:
Abnormalities in Brain Structure
Heavy-drinking adolescents showed:
Accelerated gray matter reduction in cortical lateral frontal & temporal volumes
Attenuated white matter growth of the corpus callosum and pons relative to nondrinkers
Structural Changes
Smaller, thinner frontal, and temporal cortices
Aberrations in hippocampal asymmetry
Reduced Hippocampal volumes (irrespective of family history)
Smaller cerebellar volumes in adolescent binge drinkers (more intense drinkers)
Brain Development in Heavy-drinking Adolescents. Lindsay M. Squeglia, Susan F. Tapert, Edith V. Sullivan, Joanna Jacobus, M.J. Meloy, Torsten Rohlfing, Adolf Pfefferbaum. American Journal Of Psychiatry 2015 172:6, 531-542
Adolescent Development of Cortical and White Matter Structure in the NCANDA Sample: Role of Sex, Ethnicity, Puberty, and Alcohol Drinking. Pfefferbaum A. et al. Cereb. Cortex 2016 Oct;26(10):4101-21. doi: 10.1093/cercor/bhv205. Epub 2015 Sep 26.
Recent binge drinking predicts smaller cerebellar volumes in adolescents. Lisdahl KM, Thayer R, Squeglia LM, McQueeny TM, Tapert SF. Psychiatry Res. 2013 Jan 30;211(1):17-23. doi: 10.1016/j.pscychresns.2012.07.009. Epub 2012 Nov 13.
B-18

## Slide 19
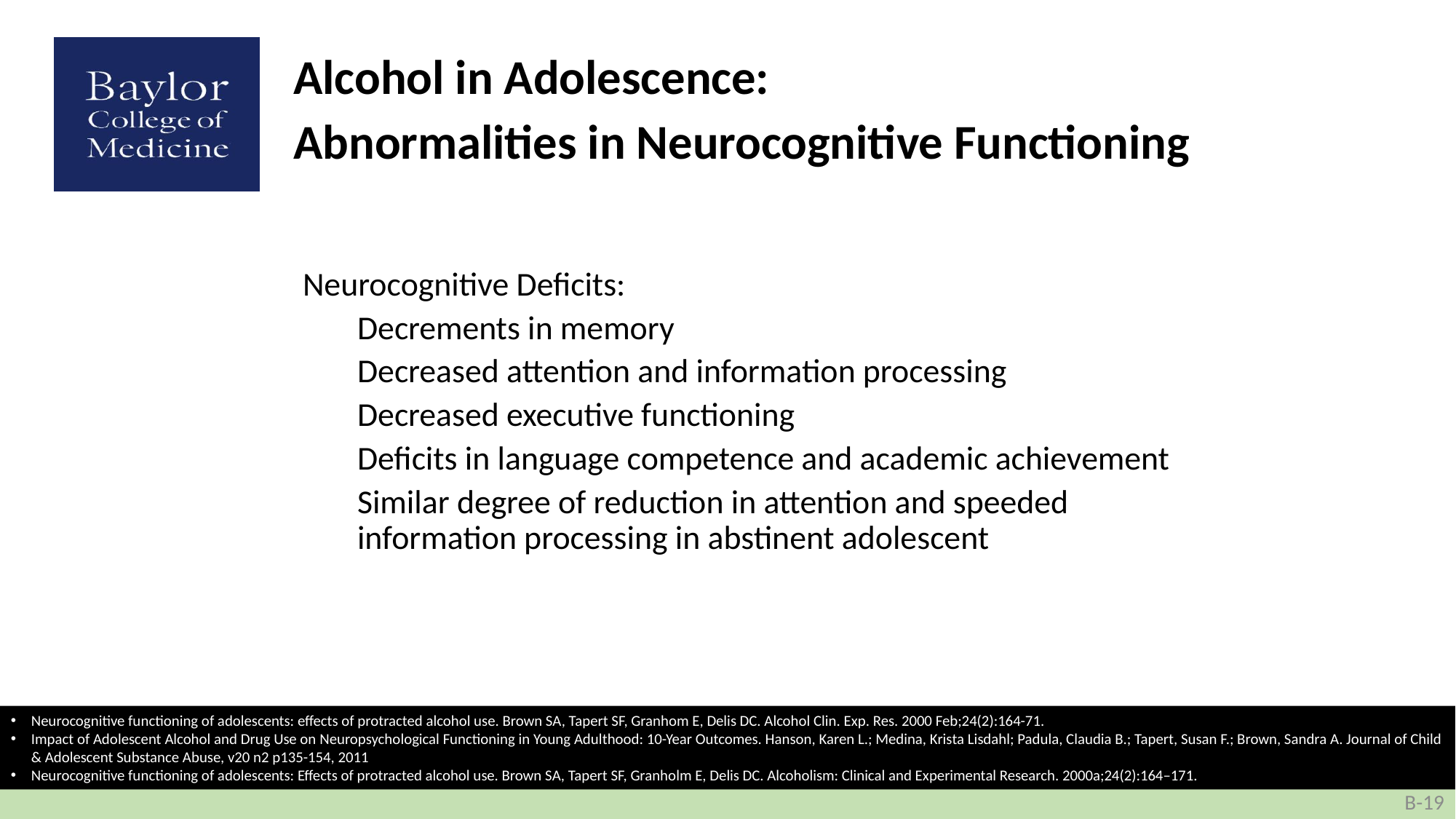

Alcohol in Adolescence:
Abnormalities in Neurocognitive Functioning
Neurocognitive Deficits:
Decrements in memory
Decreased attention and information processing
Decreased executive functioning
Deficits in language competence and academic achievement
Similar degree of reduction in attention and speeded information processing in abstinent adolescent
Neurocognitive functioning of adolescents: effects of protracted alcohol use. Brown SA, Tapert SF, Granhom E, Delis DC. Alcohol Clin. Exp. Res. 2000 Feb;24(2):164-71.
Impact of Adolescent Alcohol and Drug Use on Neuropsychological Functioning in Young Adulthood: 10-Year Outcomes. Hanson, Karen L.; Medina, Krista Lisdahl; Padula, Claudia B.; Tapert, Susan F.; Brown, Sandra A. Journal of Child & Adolescent Substance Abuse, v20 n2 p135-154, 2011
Neurocognitive functioning of adolescents: Effects of protracted alcohol use. Brown SA, Tapert SF, Granholm E, Delis DC. Alcoholism: Clinical and Experimental Research. 2000a;24(2):164–171.
B-19

## Slide 20
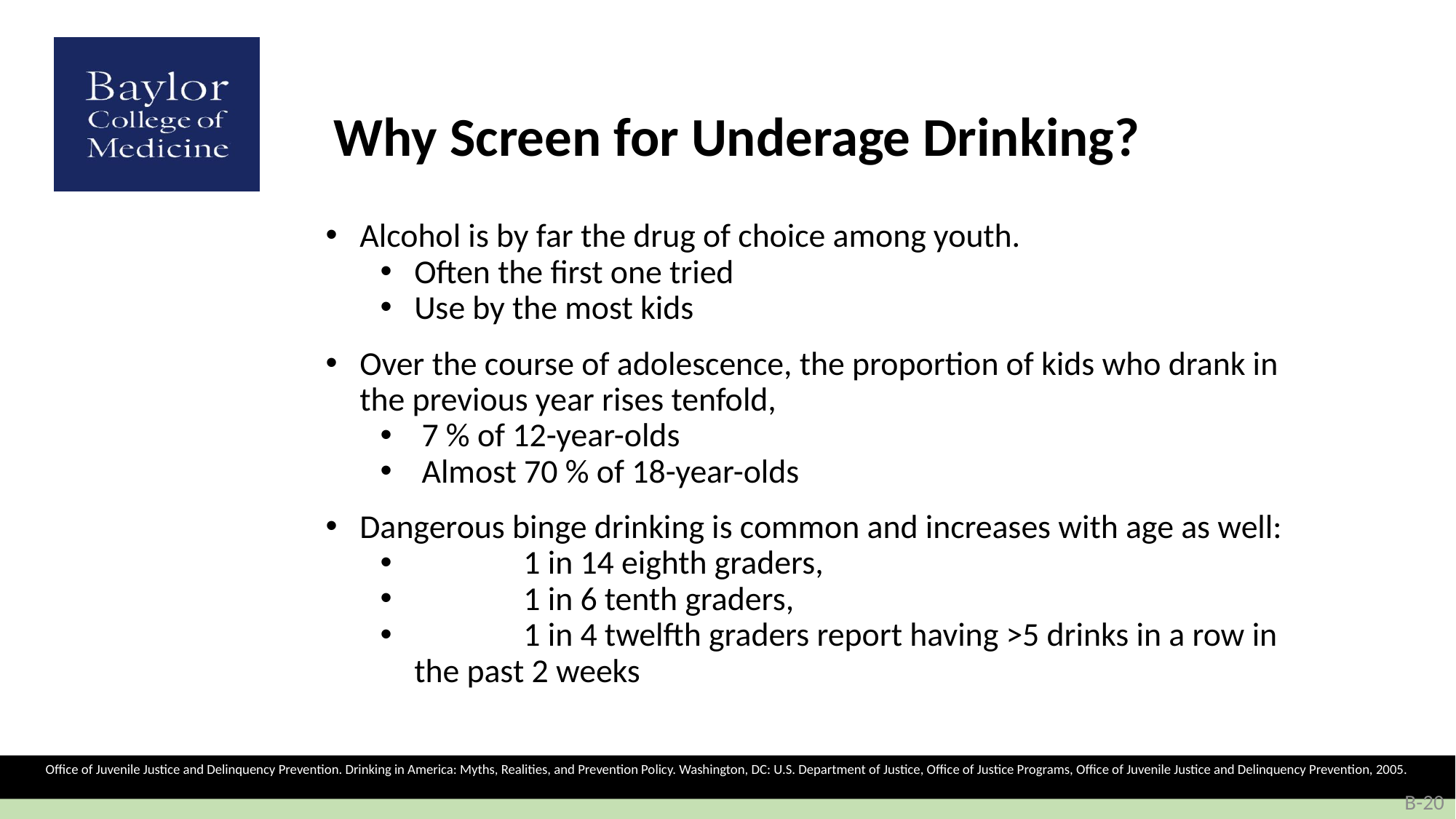

Why Screen for Underage Drinking?
Alcohol is by far the drug of choice among youth.
Often the first one tried
Use by the most kids
Over the course of adolescence, the proportion of kids who drank in the previous year rises tenfold,
 7 % of 12-year-olds
 Almost 70 % of 18-year-olds
Dangerous binge drinking is common and increases with age as well:
	1 in 14 eighth graders,
	1 in 6 tenth graders,
	1 in 4 twelfth graders report having >5 drinks in a row in the past 2 weeks
Office of Juvenile Justice and Delinquency Prevention. Drinking in America: Myths, Realities, and Prevention Policy. Washington, DC: U.S. Department of Justice, Office of Justice Programs, Office of Juvenile Justice and Delinquency Prevention, 2005.
B-20

## Slide 21
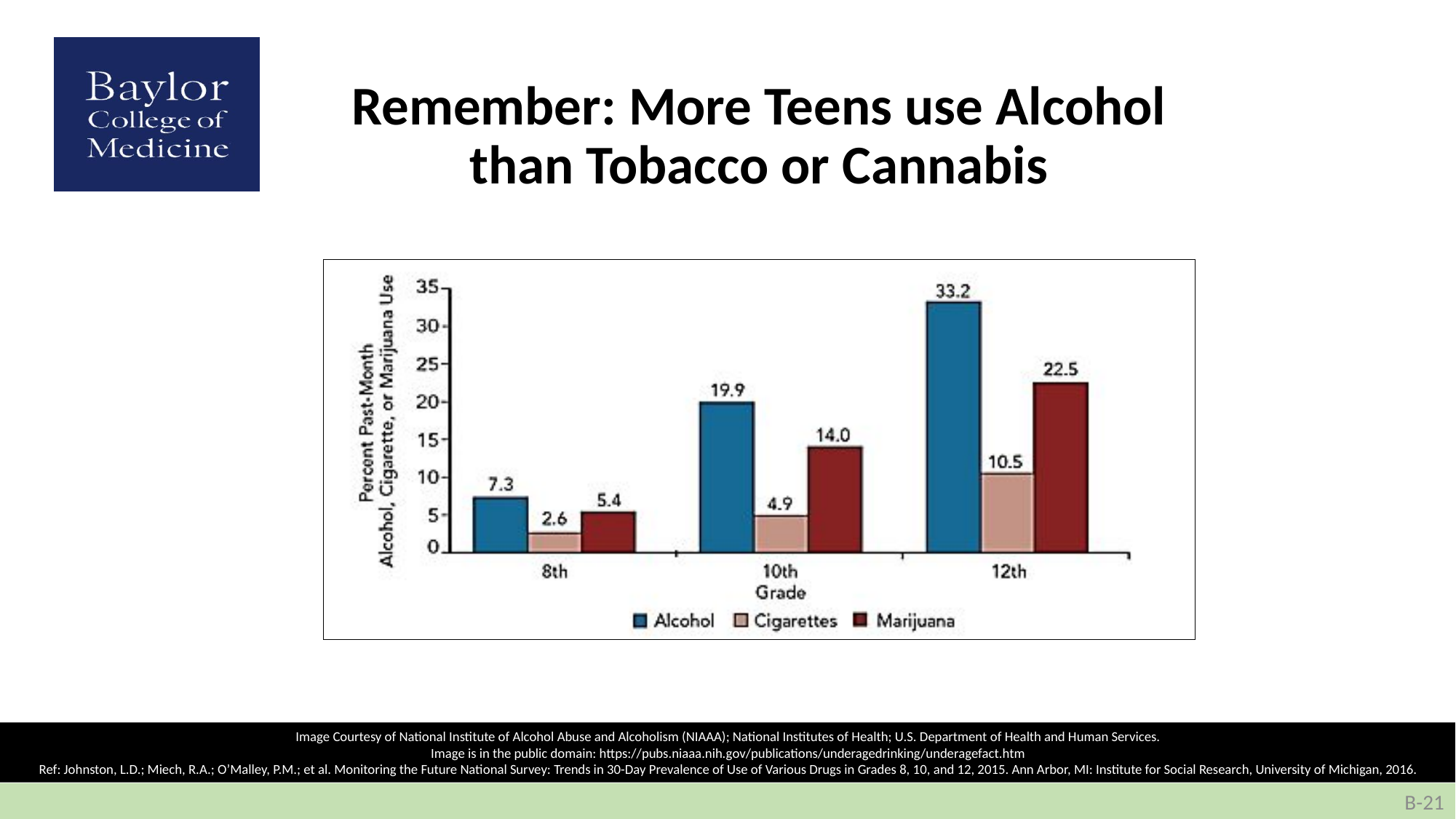

Remember: More Teens use Alcohol than Tobacco or Cannabis
Image Courtesy of National Institute of Alcohol Abuse and Alcoholism (NIAAA); National Institutes of Health; U.S. Department of Health and Human Services.
Image is in the public domain: https://pubs.niaaa.nih.gov/publications/underagedrinking/underagefact.htm
Ref: Johnston, L.D.; Miech, R.A.; O’Malley, P.M.; et al. Monitoring the Future National Survey: Trends in 30-Day Prevalence of Use of Various Drugs in Grades 8, 10, and 12, 2015. Ann Arbor, MI: Institute for Social Research, University of Michigan, 2016.
B-21

## Slide 22
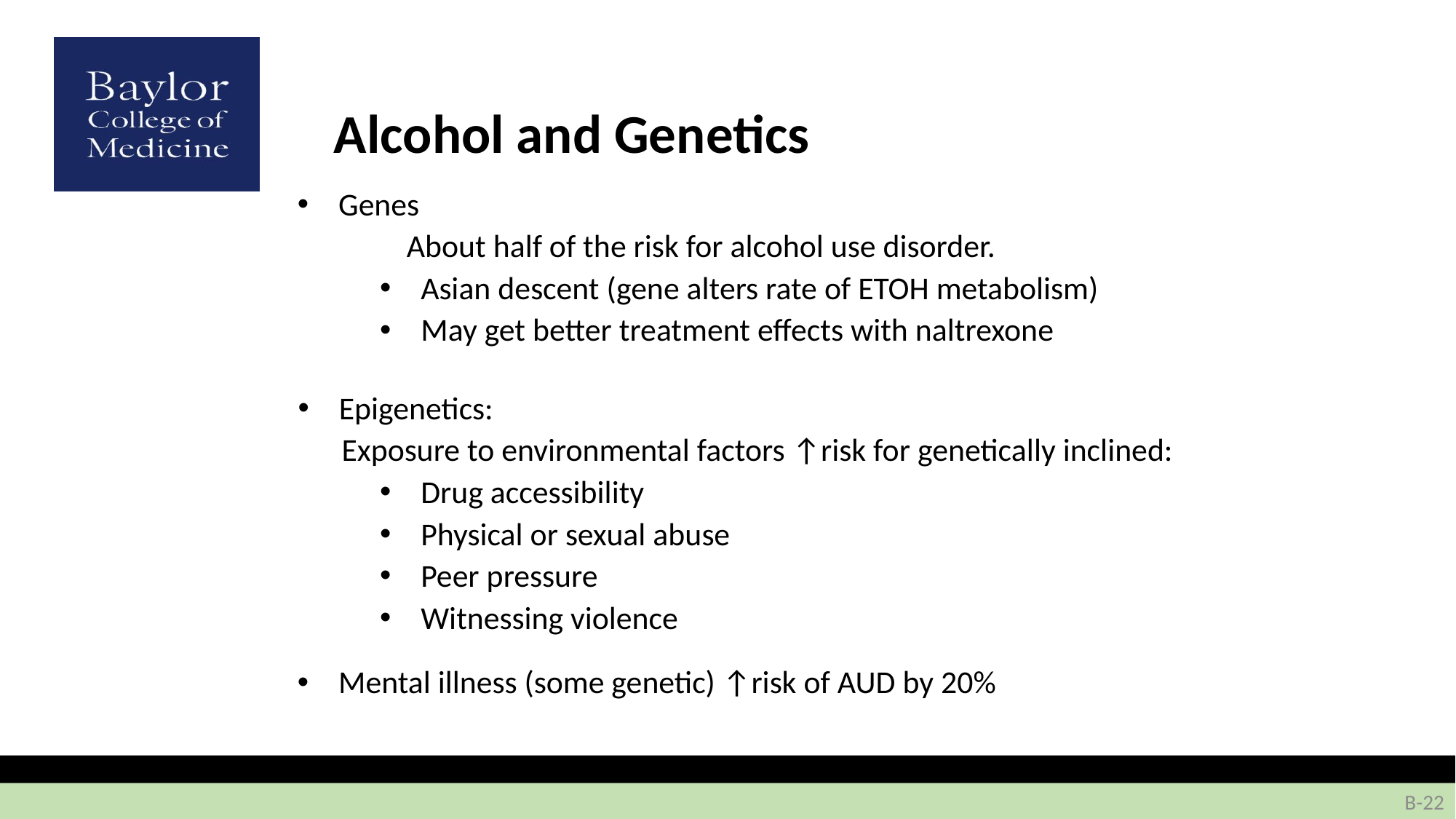

Alcohol and Genetics
Genes
	About half of the risk for alcohol use disorder.
Asian descent (gene alters rate of ETOH metabolism)
May get better treatment effects with naltrexone
Epigenetics:
 Exposure to environmental factors ↑risk for genetically inclined:
Drug accessibility
Physical or sexual abuse
Peer pressure
Witnessing violence
Mental illness (some genetic) ↑risk of AUD by 20%
B-22

## Slide 23
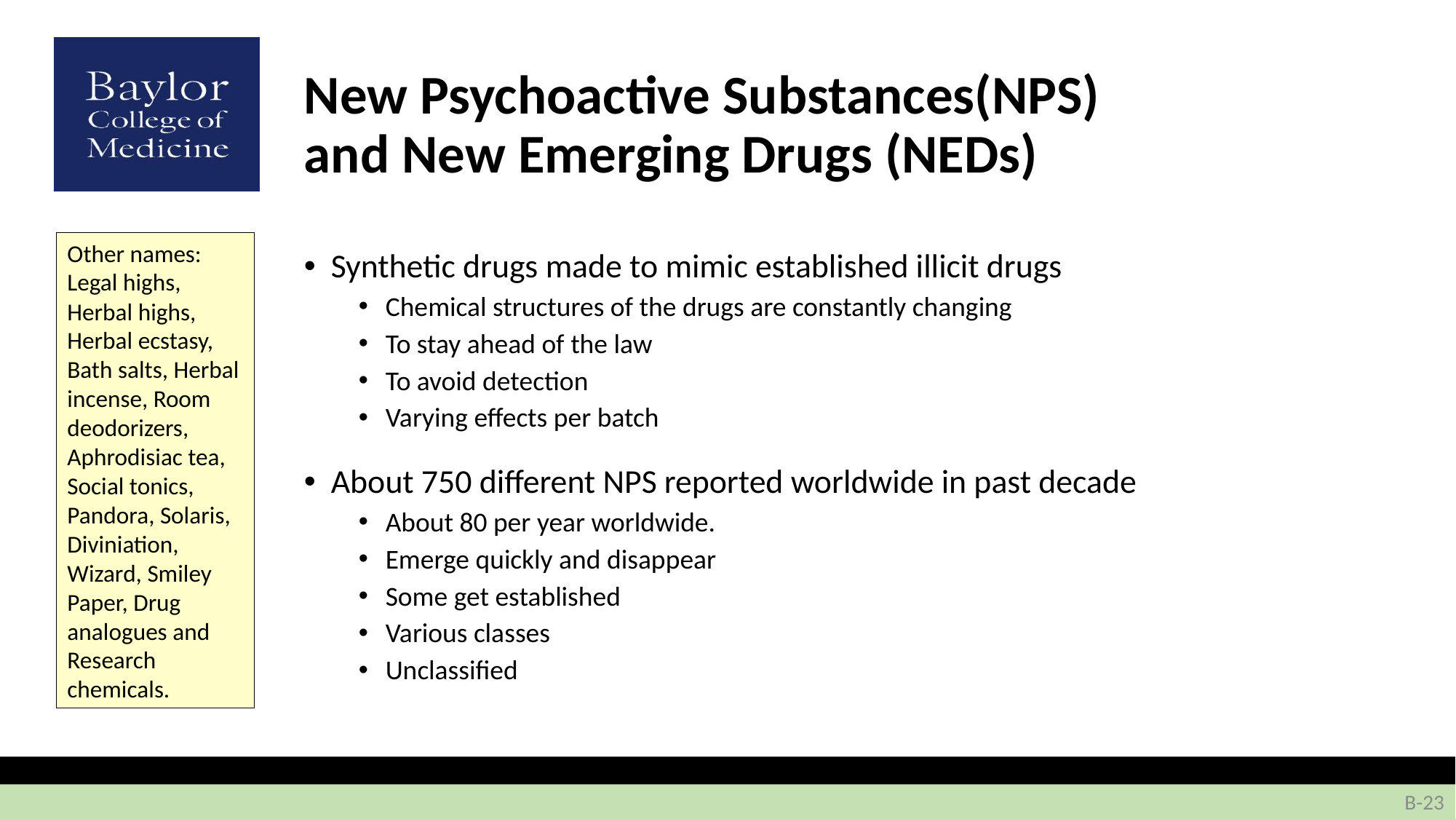

New Psychoactive Substances(NPS)
and New Emerging Drugs (NEDs)
Other names: Legal highs, Herbal highs, Herbal ecstasy, Bath salts, Herbal incense, Room deodorizers, Aphrodisiac tea, Social tonics, Pandora, Solaris, Diviniation, Wizard, Smiley Paper, Drug analogues and Research chemicals.
Synthetic drugs made to mimic established illicit drugs
Chemical structures of the drugs are constantly changing
To stay ahead of the law
To avoid detection
Varying effects per batch
About 750 different NPS reported worldwide in past decade
About 80 per year worldwide.
Emerge quickly and disappear
Some get established
Various classes
Unclassified
B-23

## Slide 24
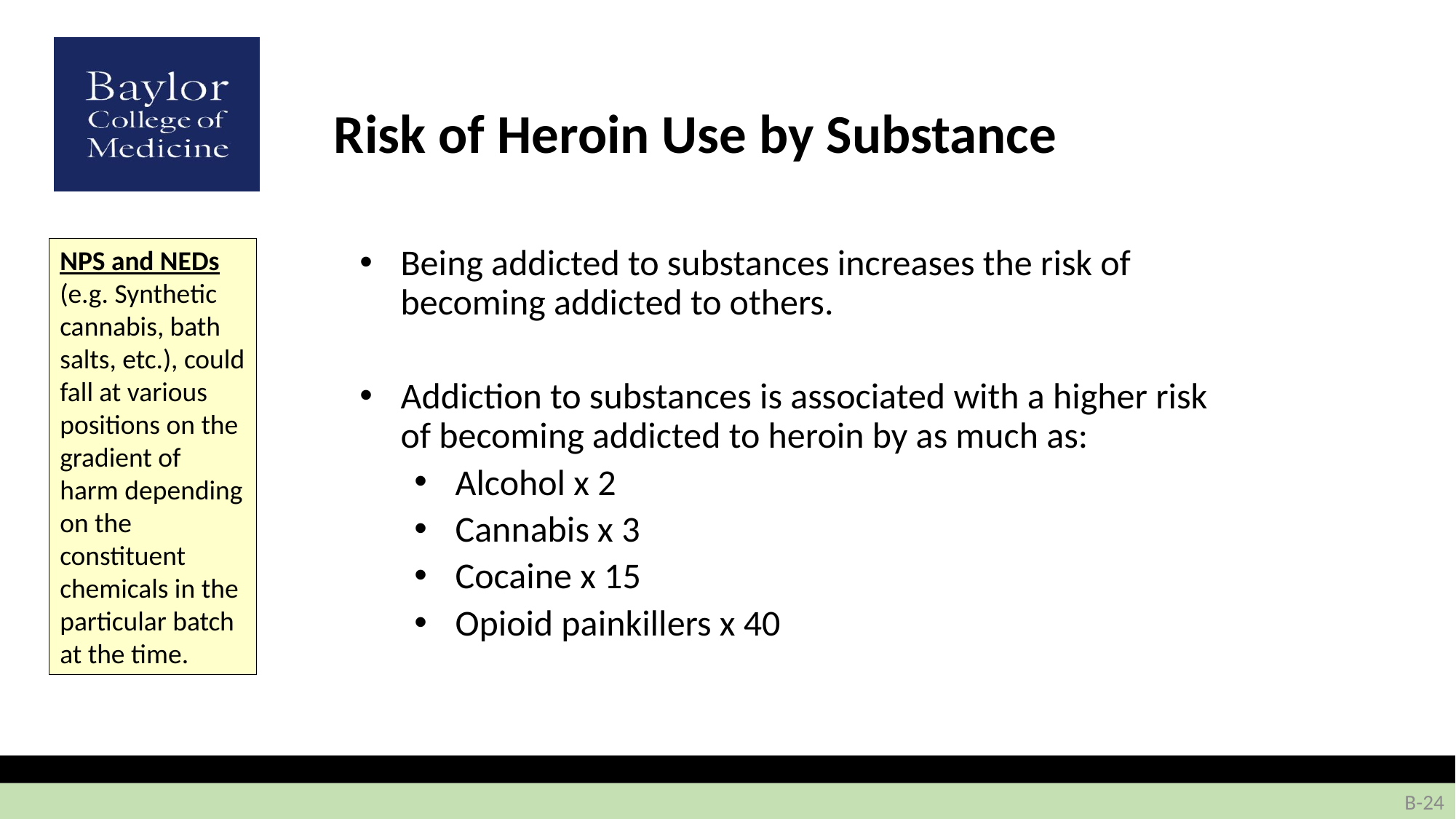

Risk of Heroin Use by Substance
NPS and NEDs
(e.g. Synthetic cannabis, bath salts, etc.), could fall at various positions on the gradient of harm depending on the constituent chemicals in the particular batch at the time.
Being addicted to substances increases the risk of becoming addicted to others.
Addiction to substances is associated with a higher risk of becoming addicted to heroin by as much as:
Alcohol x 2
Cannabis x 3
Cocaine x 15
Opioid painkillers x 40
B-24

## Slide 25
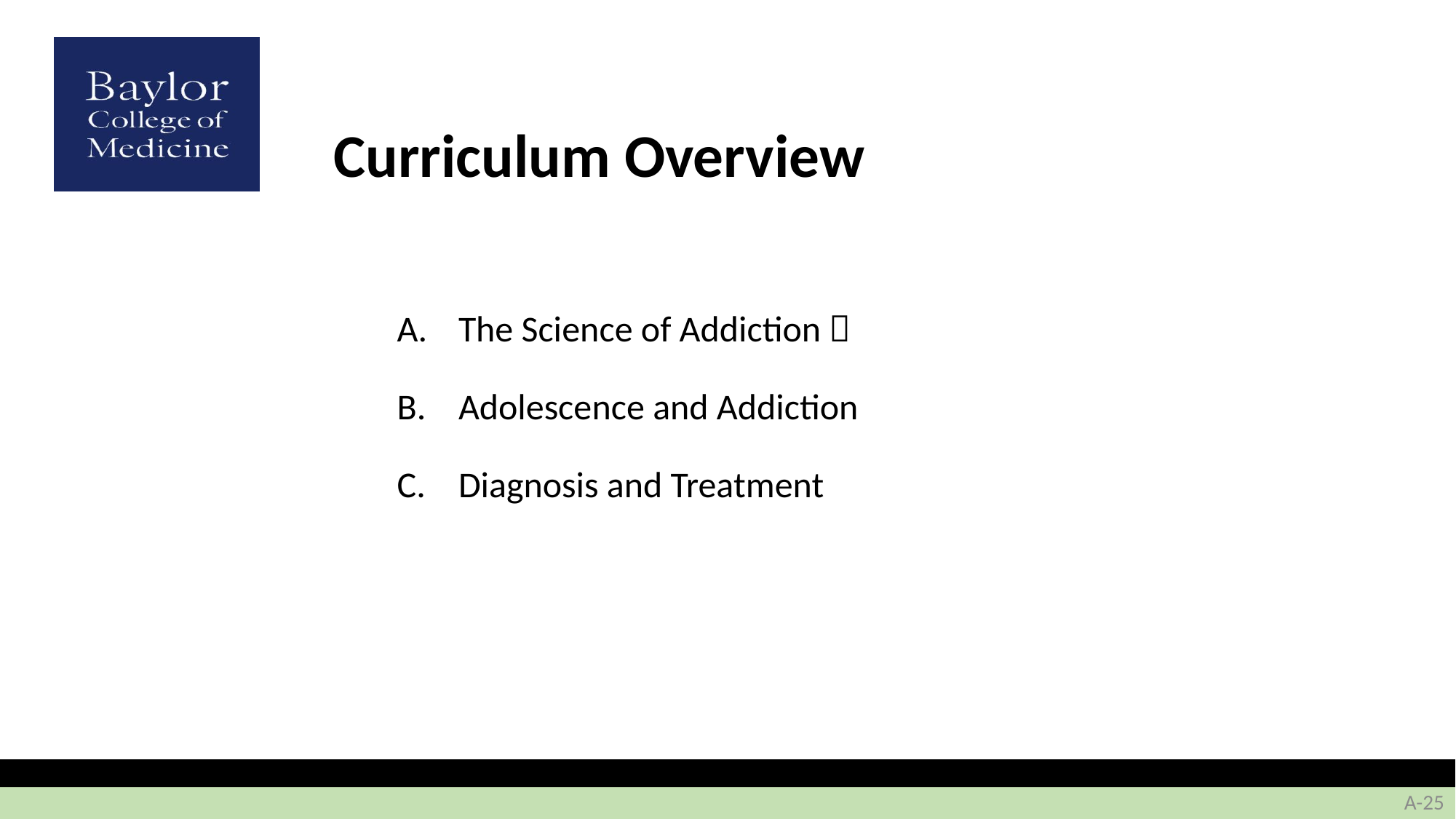

Curriculum Overview
The Science of Addiction 
Adolescence and Addiction
Diagnosis and Treatment
A-25

## Slide 26
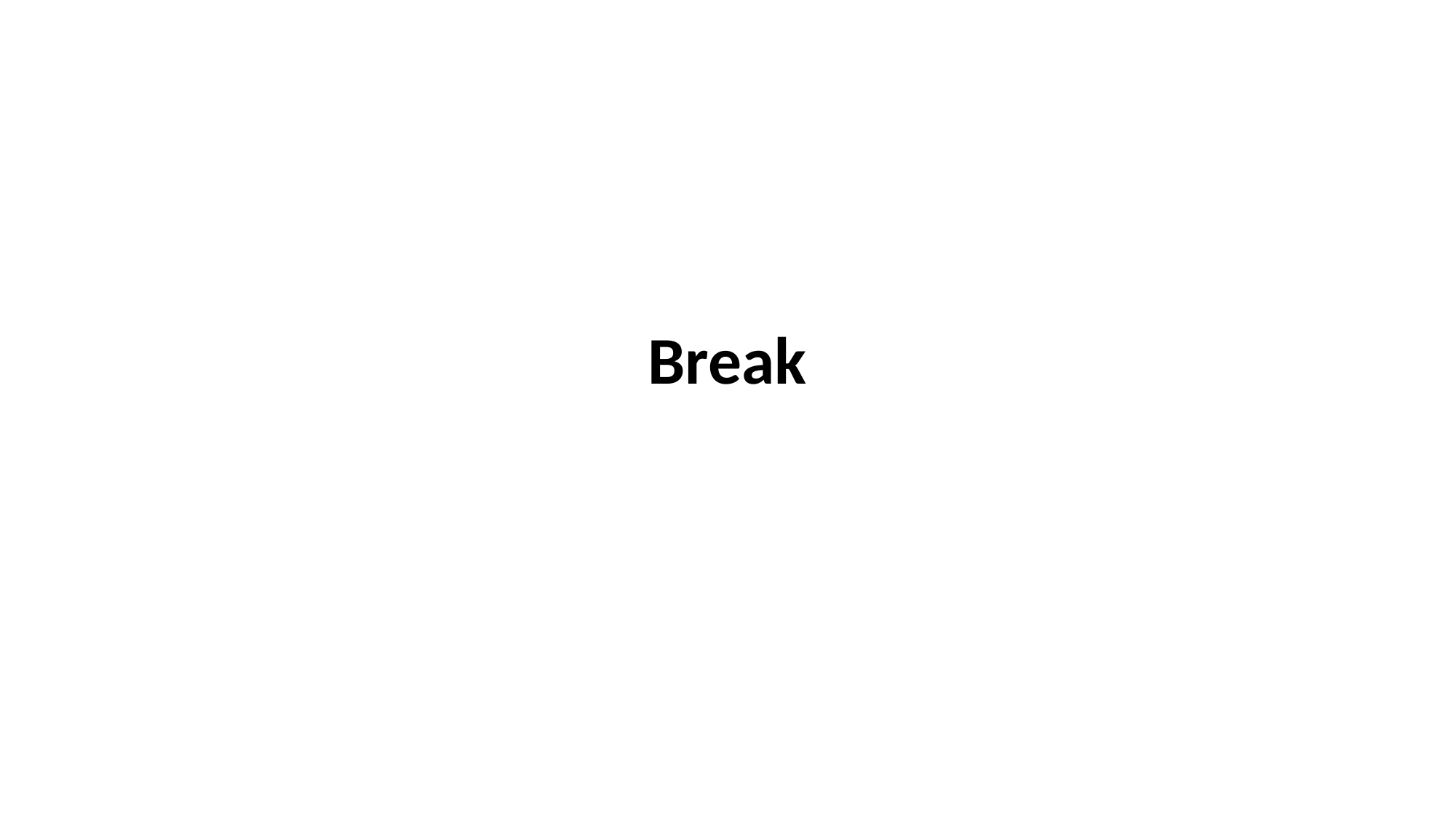

# Break

## Slide 27
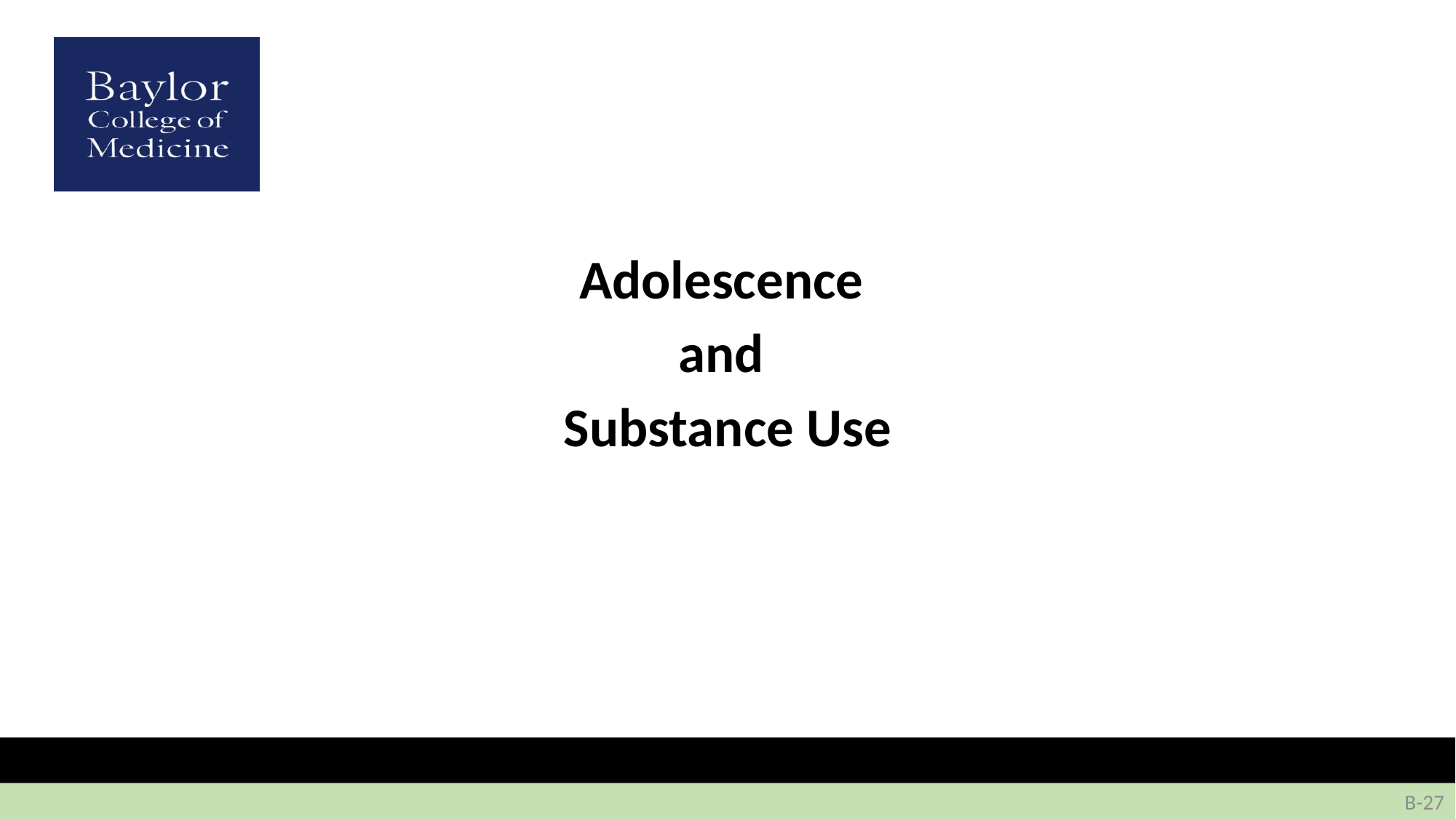

Adolescence
and
Substance Use
B-27

## Slide 28
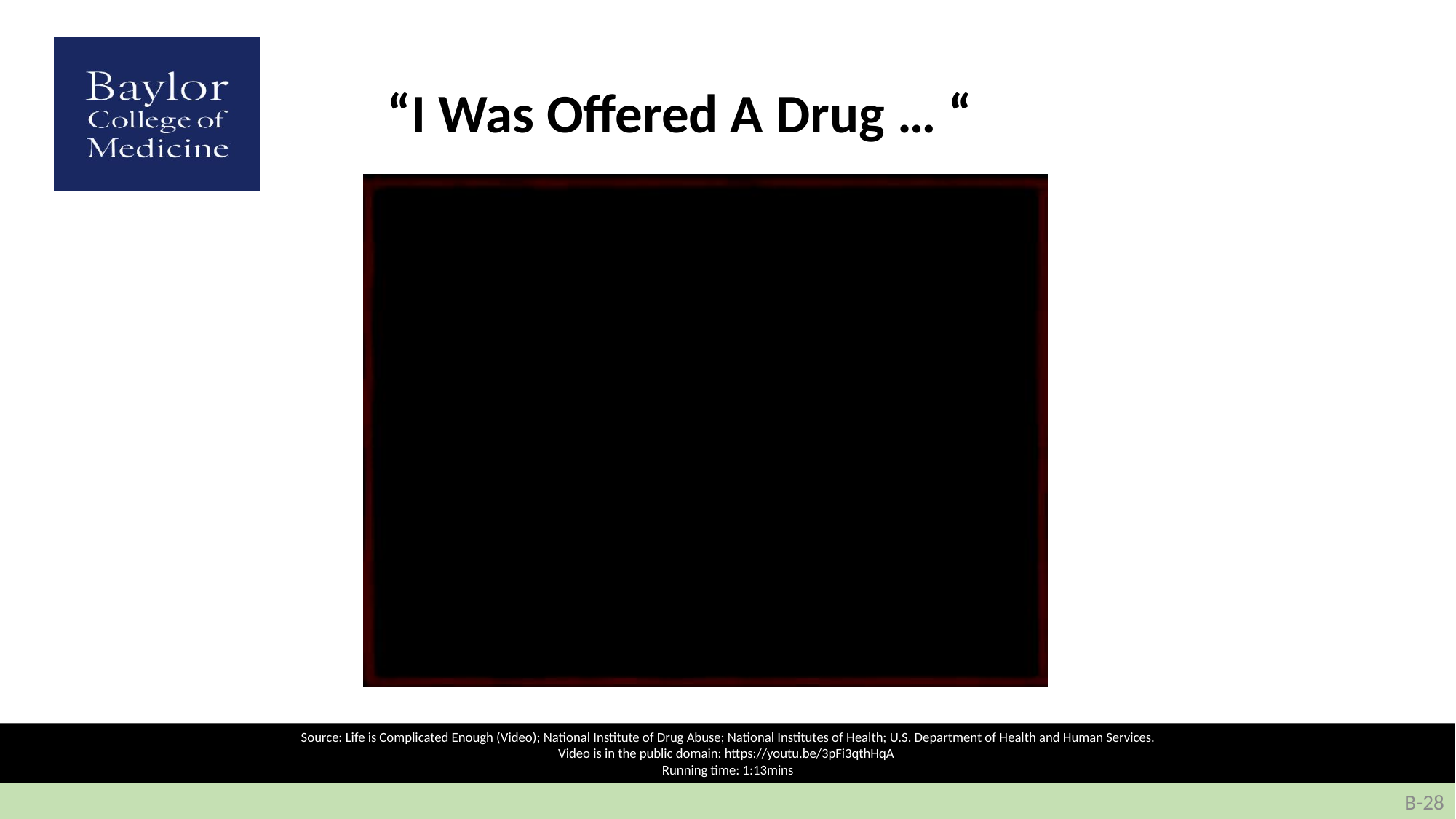

“I Was Offered A Drug … “
Source: Life is Complicated Enough (Video); National Institute of Drug Abuse; National Institutes of Health; U.S. Department of Health and Human Services.
Video is in the public domain: https://youtu.be/3pFi3qthHqA
Running time: 1:13mins
B-28

## Slide 29
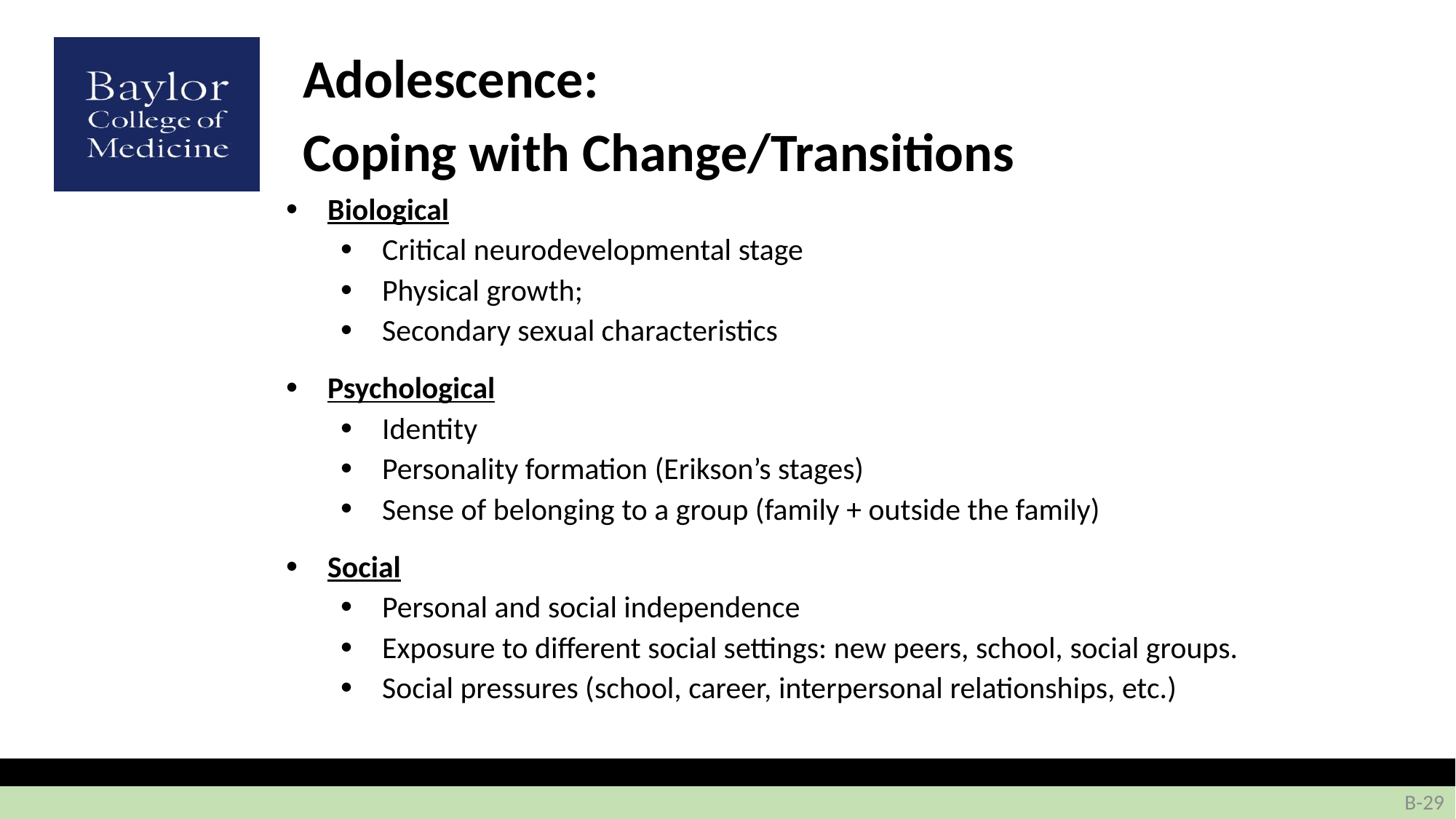

Adolescence:
Coping with Change/Transitions
Biological
Critical neurodevelopmental stage
Physical growth;
Secondary sexual characteristics
Psychological
Identity
Personality formation (Erikson’s stages)
Sense of belonging to a group (family + outside the family)
Social
Personal and social independence
Exposure to different social settings: new peers, school, social groups.
Social pressures (school, career, interpersonal relationships, etc.)
B-29

## Slide 30
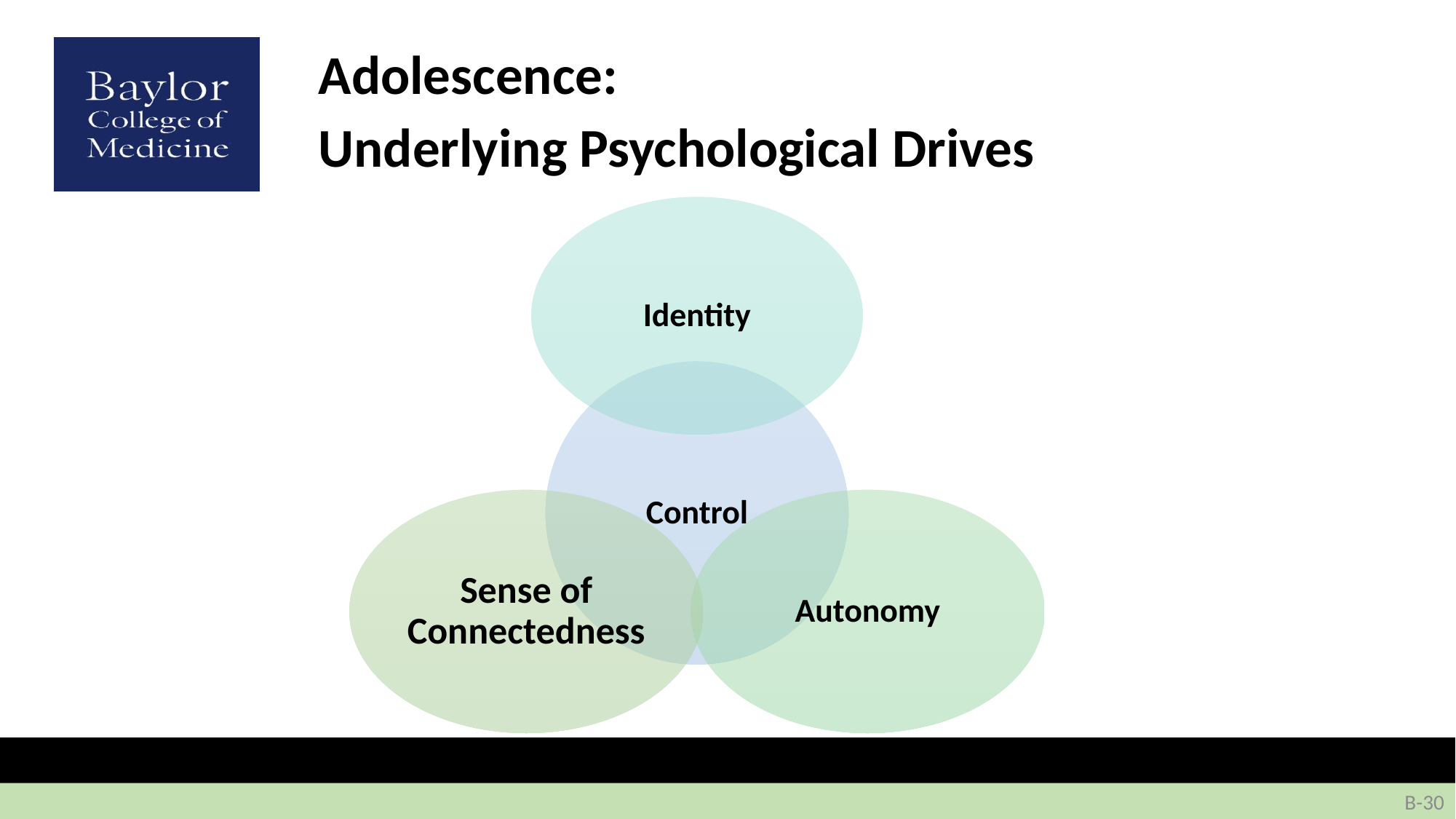

Adolescence:
Underlying Psychological Drives
B-30

## Slide 31
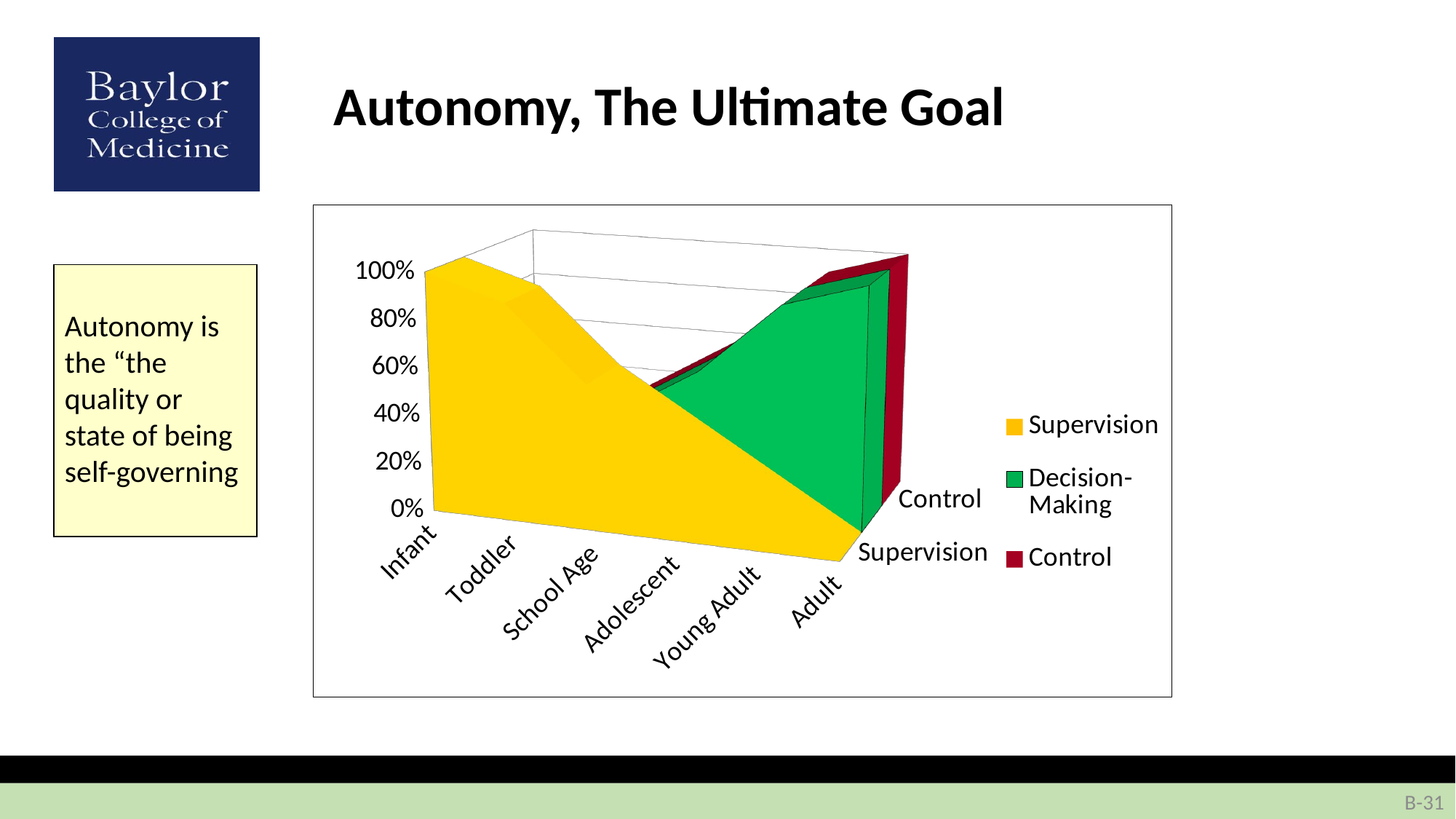

Autonomy, The Ultimate Goal
[unsupported chart]
Autonomy is the “the quality or state of being self-governing
B-31

## Slide 32
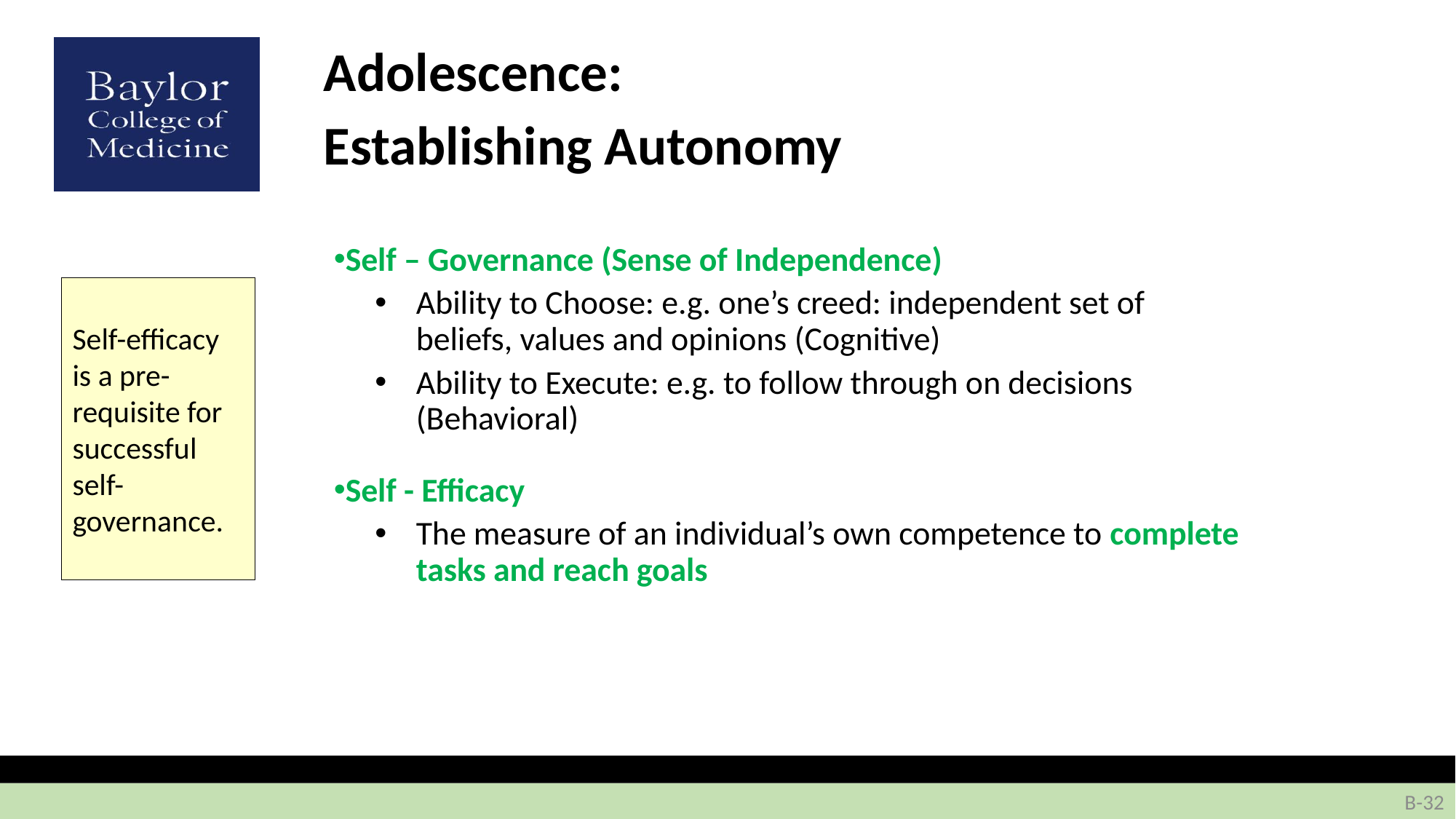

Adolescence:
Establishing Autonomy
Self – Governance (Sense of Independence)
Ability to Choose: e.g. one’s creed: independent set of beliefs, values and opinions (Cognitive)
Ability to Execute: e.g. to follow through on decisions (Behavioral)
Self - Efficacy
The measure of an individual’s own competence to complete tasks and reach goals
Self-efficacy is a pre-requisite for successful self-governance.
B-32

## Slide 33
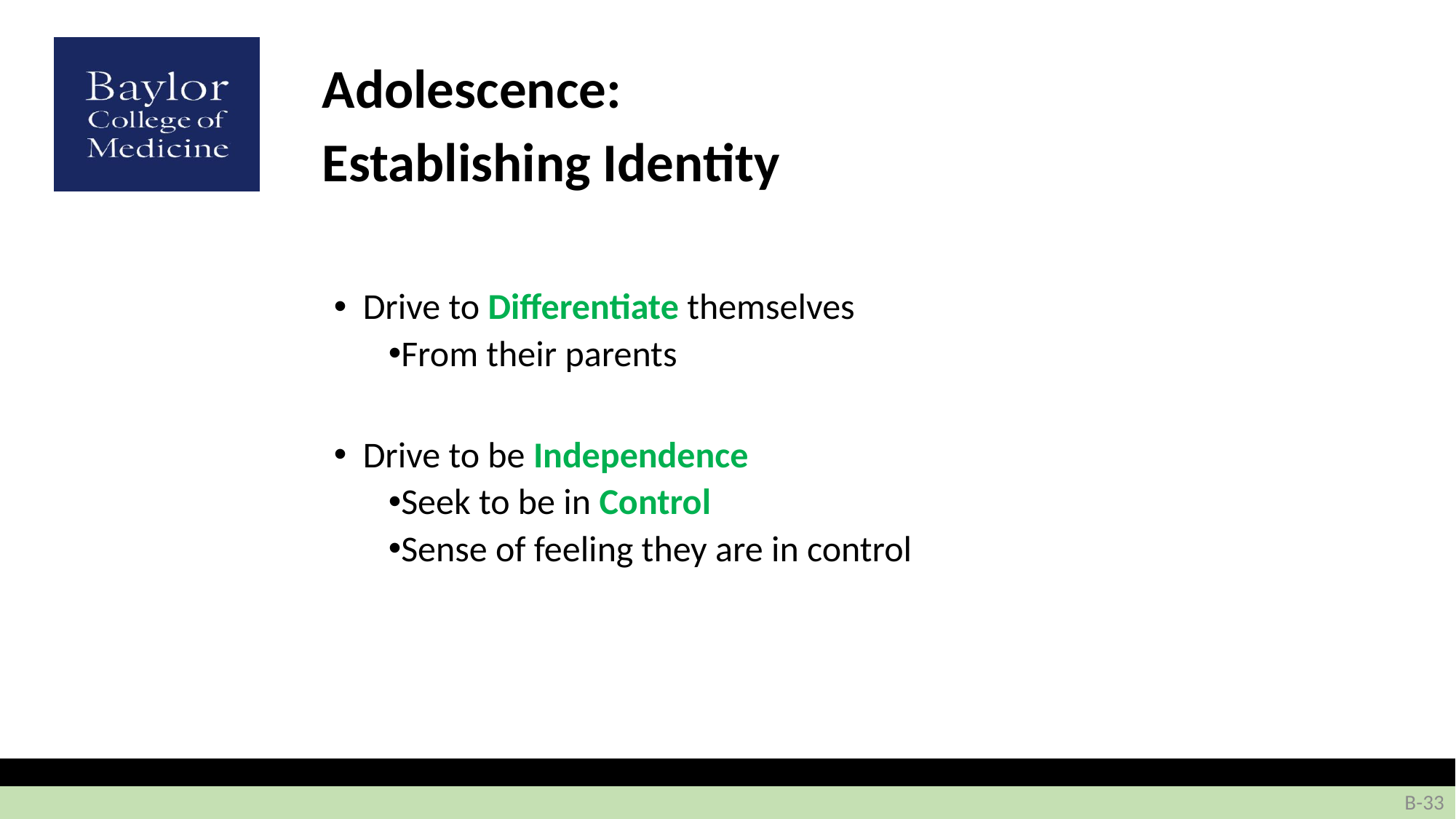

Adolescence:
Establishing Identity
 Drive to Differentiate themselves
From their parents
 Drive to be Independence
Seek to be in Control
Sense of feeling they are in control
B-33

## Slide 34
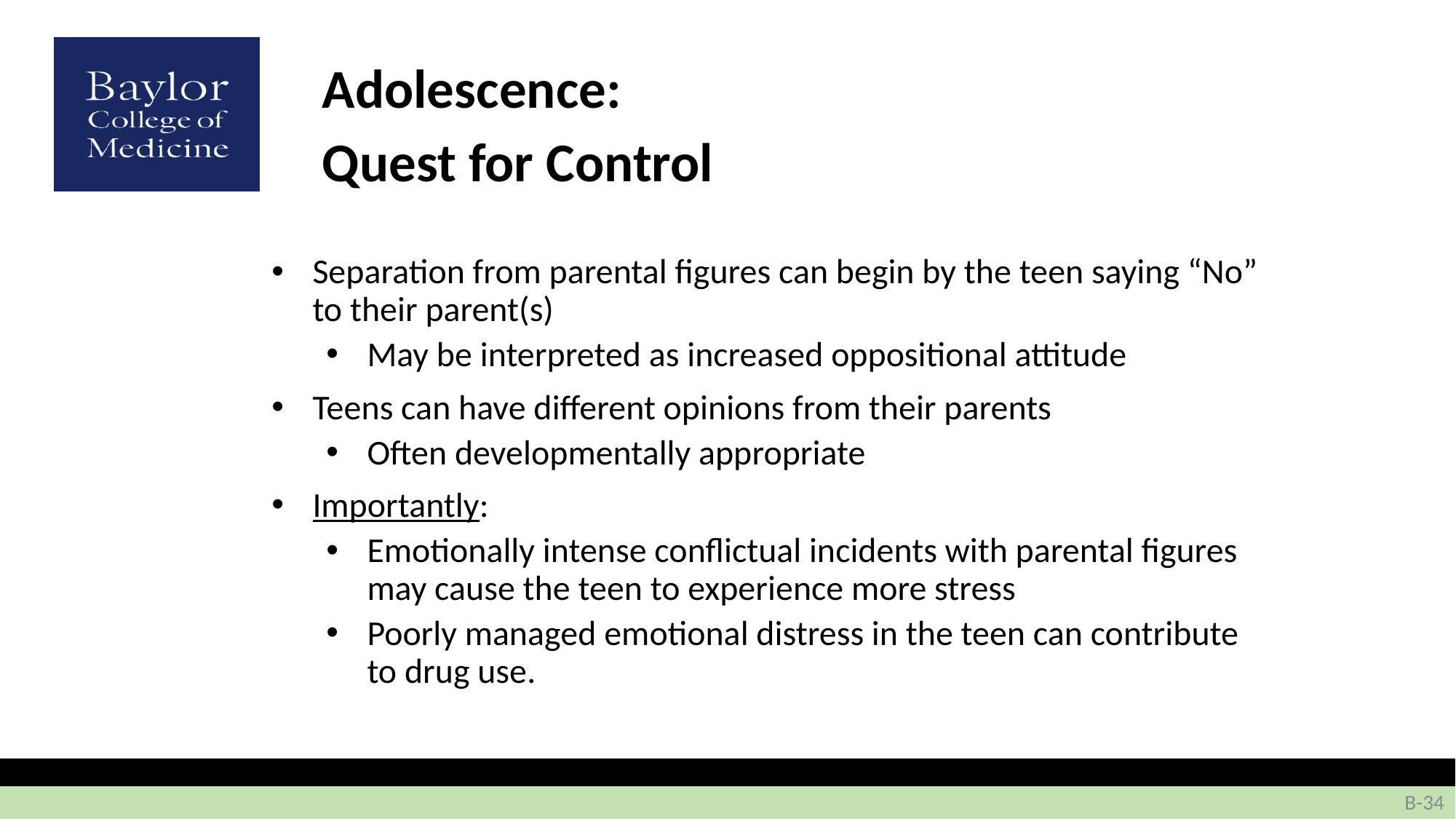

Adolescence:
Quest for Control
Separation from parental figures can begin by the teen saying “No” to their parent(s)
May be interpreted as increased oppositional attitude
Teens can have different opinions from their parents
Often developmentally appropriate
Importantly:
Emotionally intense conflictual incidents with parental figures may cause the teen to experience more stress
Poorly managed emotional distress in the teen can contribute to drug use.
B-34

## Slide 35
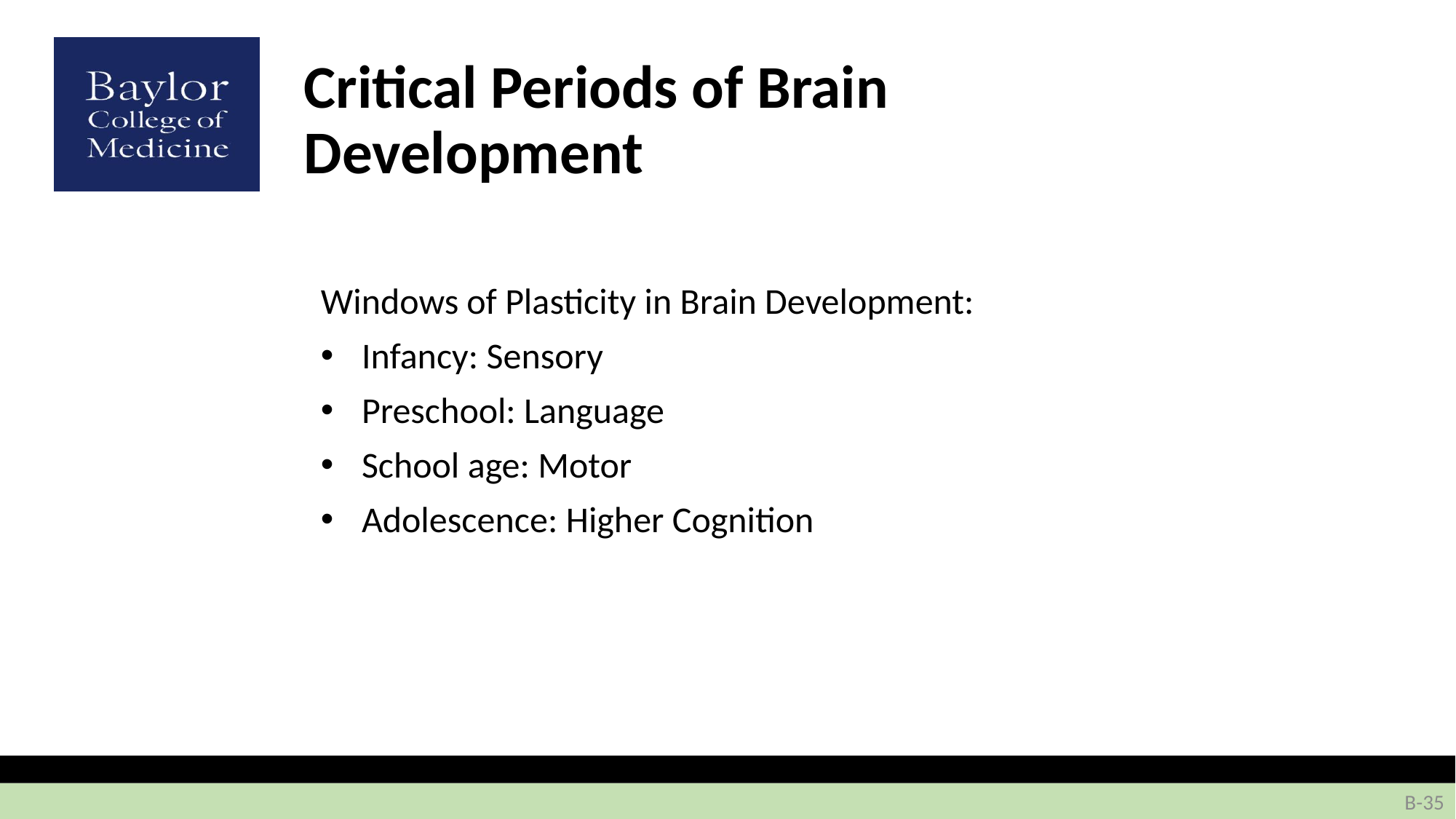

Critical Periods of Brain Development
Windows of Plasticity in Brain Development:
Infancy: Sensory
Preschool: Language
School age: Motor
Adolescence: Higher Cognition
B-35

## Slide 36
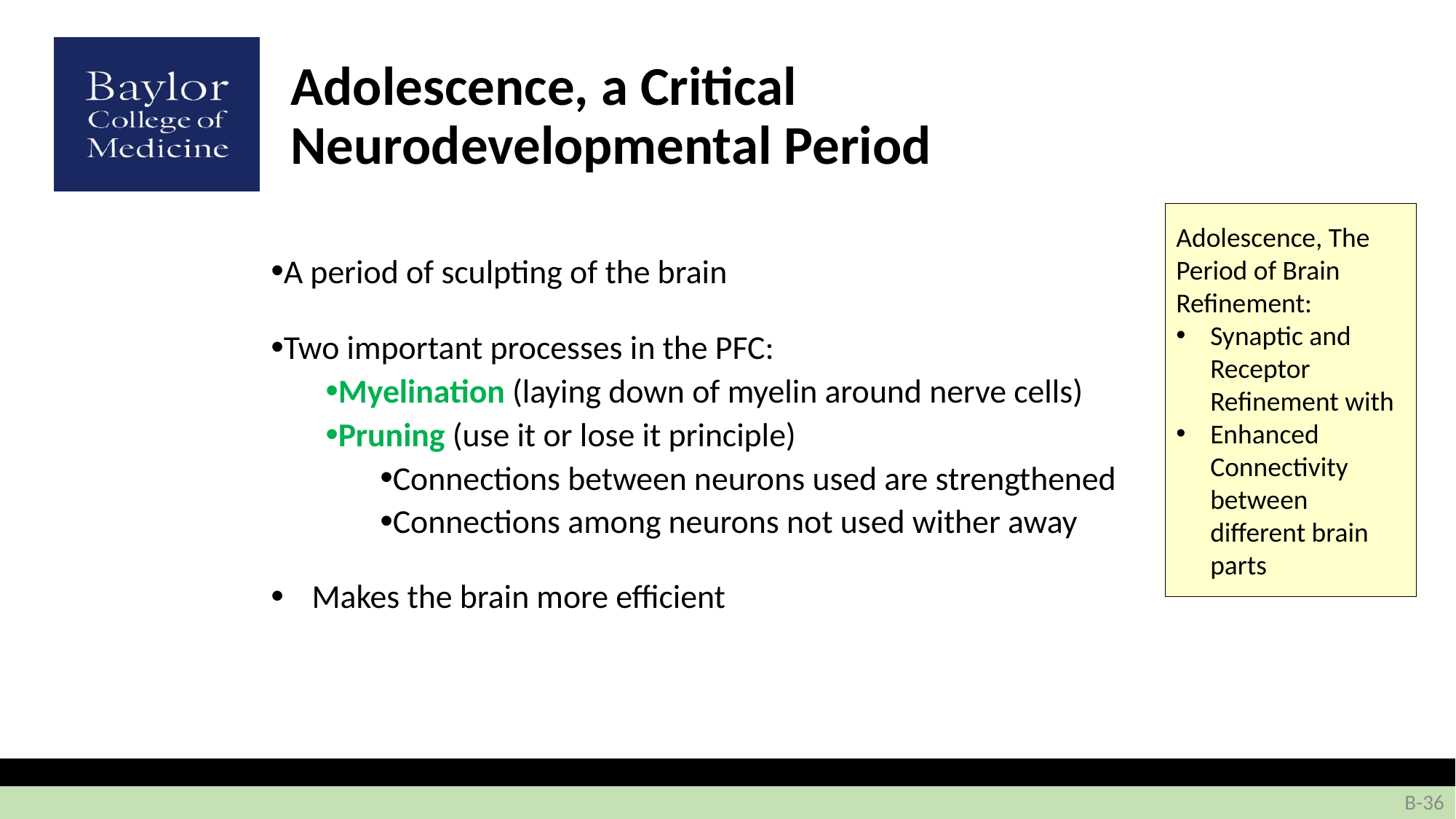

Adolescence, a Critical Neurodevelopmental Period
Adolescence, The Period of Brain Refinement:
Synaptic and Receptor Refinement with
Enhanced Connectivity between different brain parts
A period of sculpting of the brain
Two important processes in the PFC:
Myelination (laying down of myelin around nerve cells)
Pruning (use it or lose it principle)
Connections between neurons used are strengthened
Connections among neurons not used wither away
Makes the brain more efficient
B-36

## Slide 37
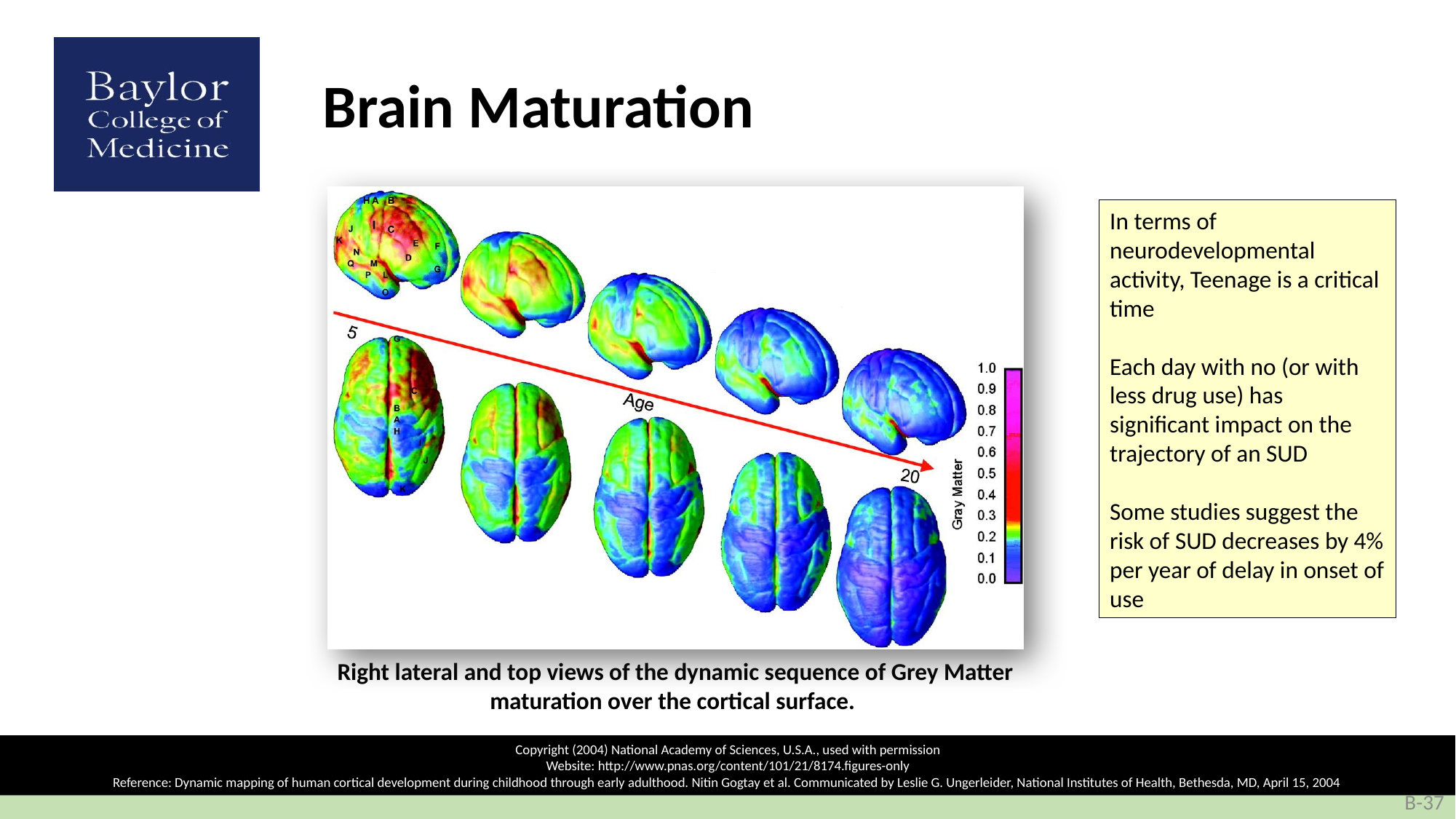

Brain Maturation
In terms of neurodevelopmental activity, Teenage is a critical time
Each day with no (or with less drug use) has significant impact on the trajectory of an SUD
Some studies suggest the risk of SUD decreases by 4% per year of delay in onset of use
Right lateral and top views of the dynamic sequence of Grey Matter maturation over the cortical surface.
Copyright (2004) National Academy of Sciences, U.S.A., used with permission
Website: http://www.pnas.org/content/101/21/8174.figures-only
Reference: Dynamic mapping of human cortical development during childhood through early adulthood. Nitin Gogtay et al. Communicated by Leslie G. Ungerleider, National Institutes of Health, Bethesda, MD, April 15, 2004
B-37

## Slide 38
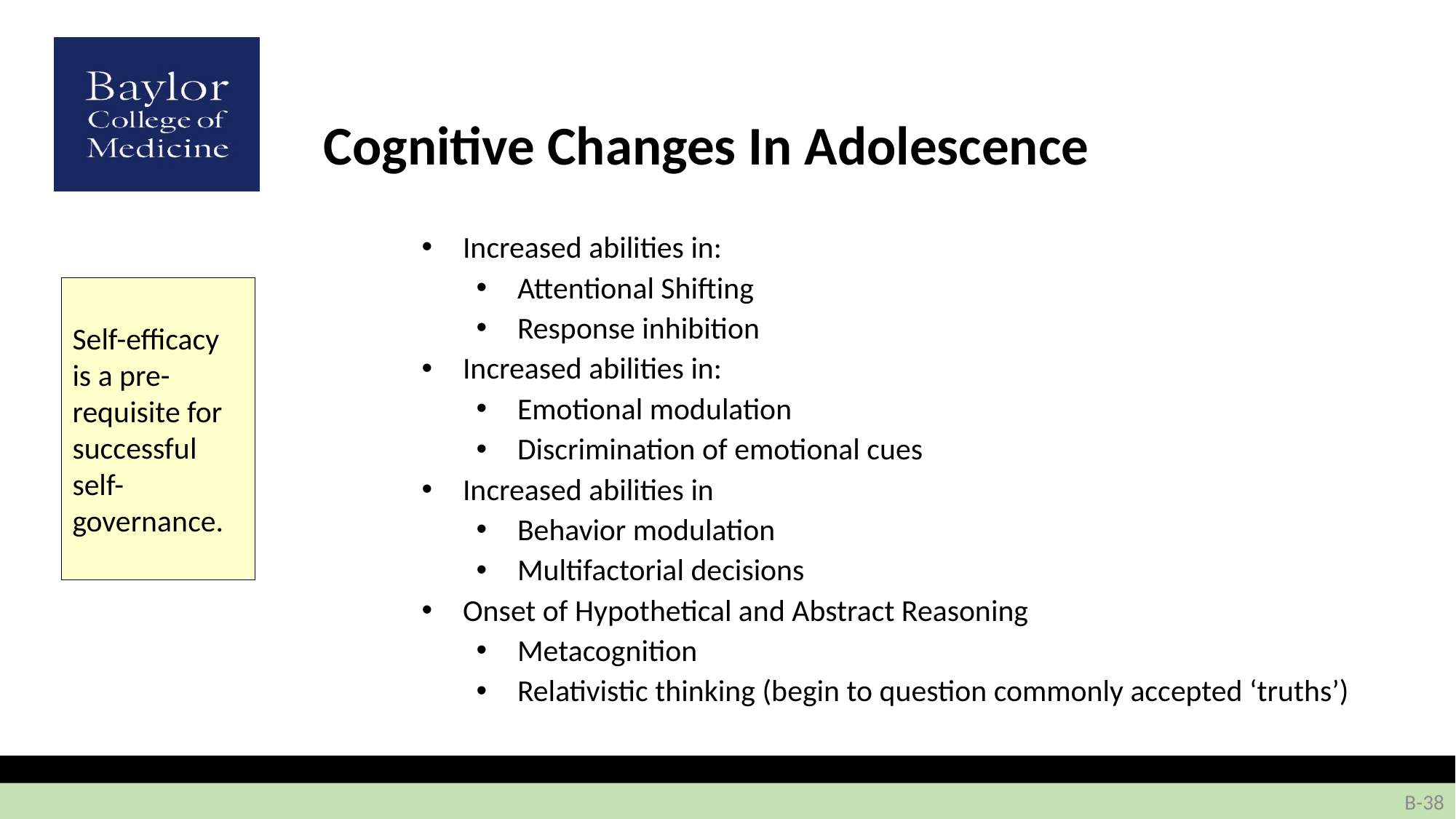

Cognitive Changes In Adolescence
Increased abilities in:
Attentional Shifting
Response inhibition
Increased abilities in:
Emotional modulation
Discrimination of emotional cues
Increased abilities in
Behavior modulation
Multifactorial decisions
Onset of Hypothetical and Abstract Reasoning
Metacognition
Relativistic thinking (begin to question commonly accepted ‘truths’)
Self-efficacy is a pre-requisite for successful self-governance.
B-38

## Slide 39
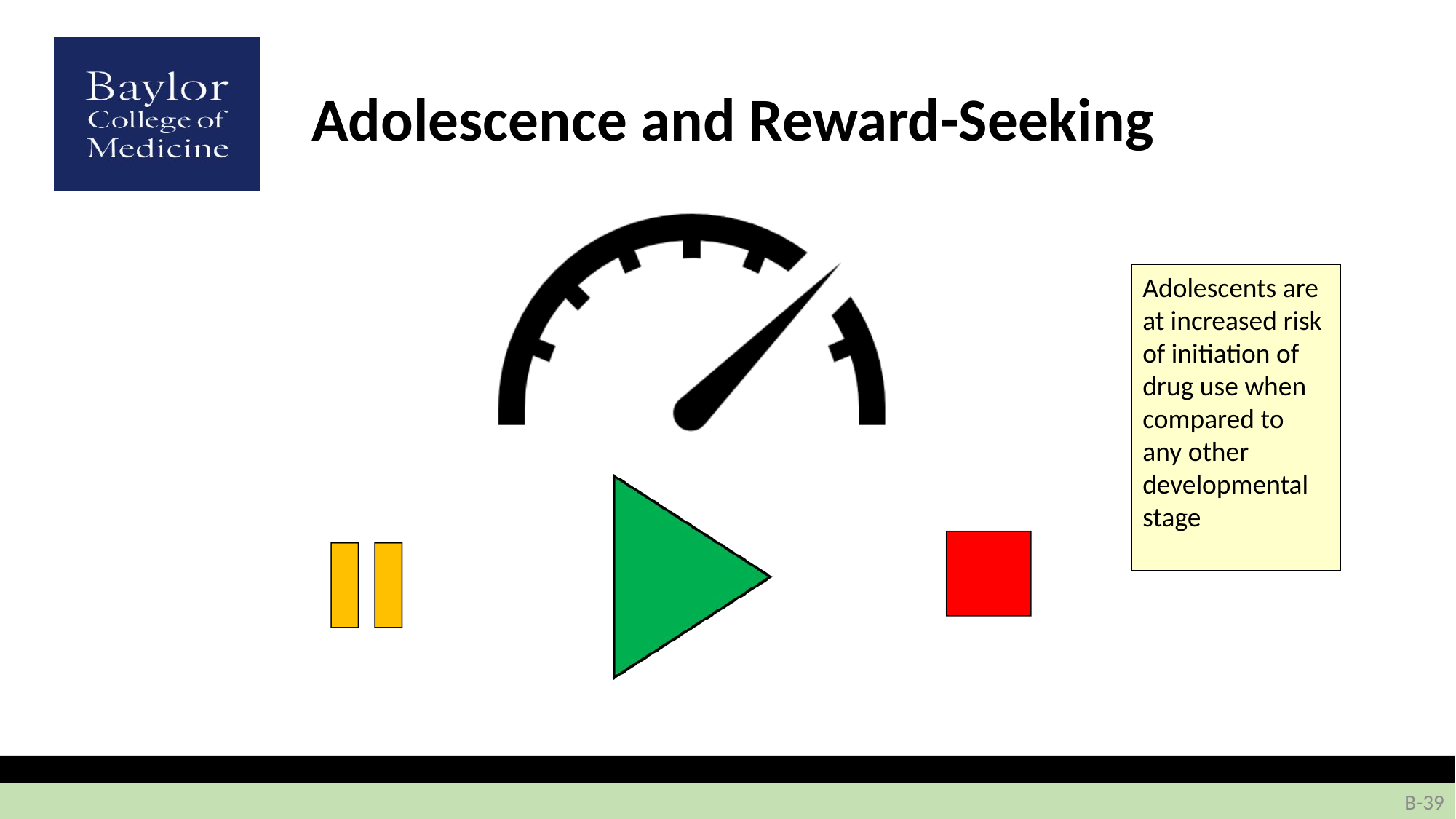

Adolescence and Reward-Seeking
Adolescents are at increased risk of initiation of drug use when compared to any other developmental stage
B-39

## Slide 40
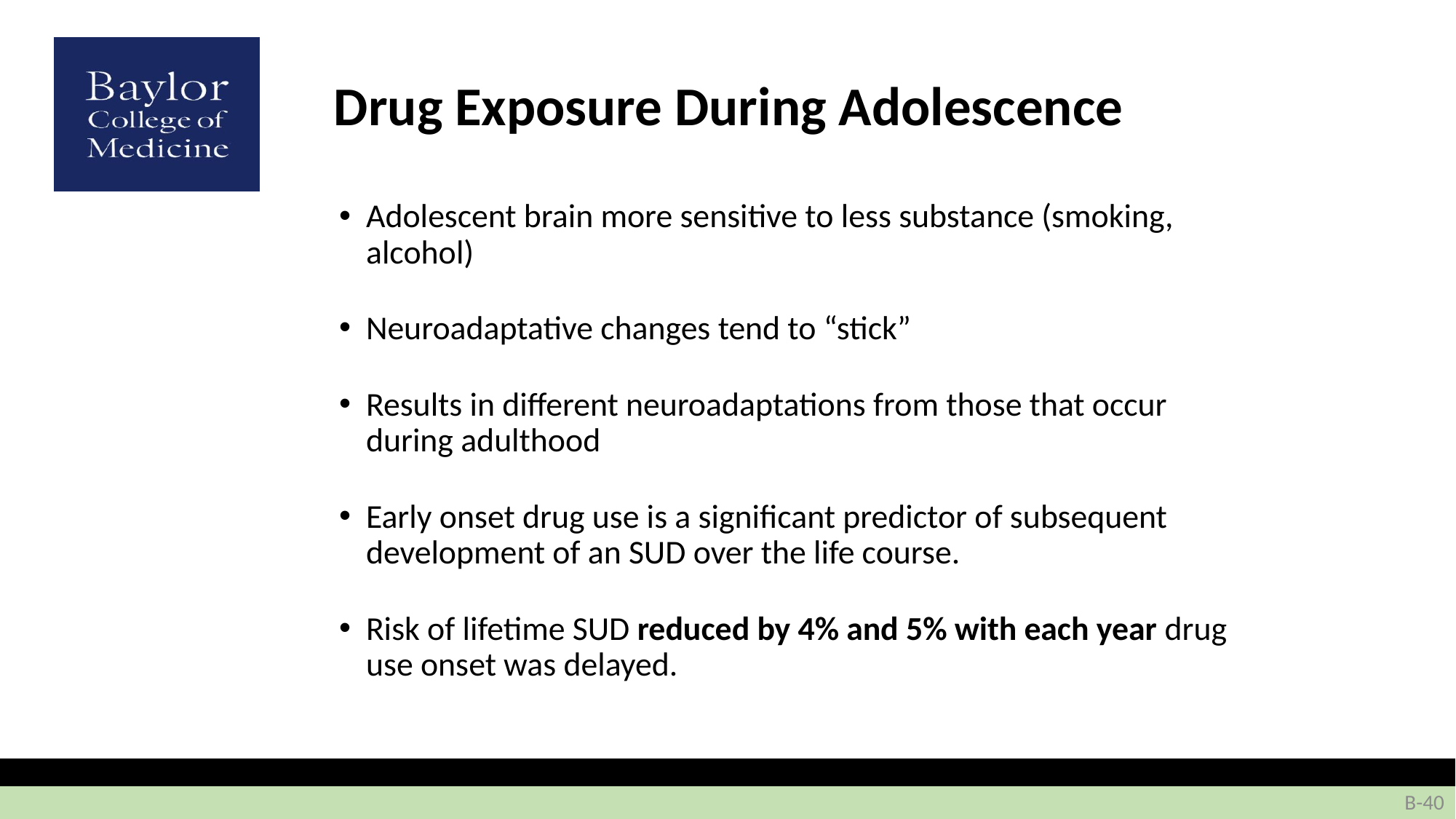

Drug Exposure During Adolescence
Adolescent brain more sensitive to less substance (smoking, alcohol)
Neuroadaptative changes tend to “stick”
Results in different neuroadaptations from those that occur during adulthood
Early onset drug use is a significant predictor of subsequent development of an SUD over the life course.
Risk of lifetime SUD reduced by 4% and 5% with each year drug use onset was delayed.
B-40

## Slide 41
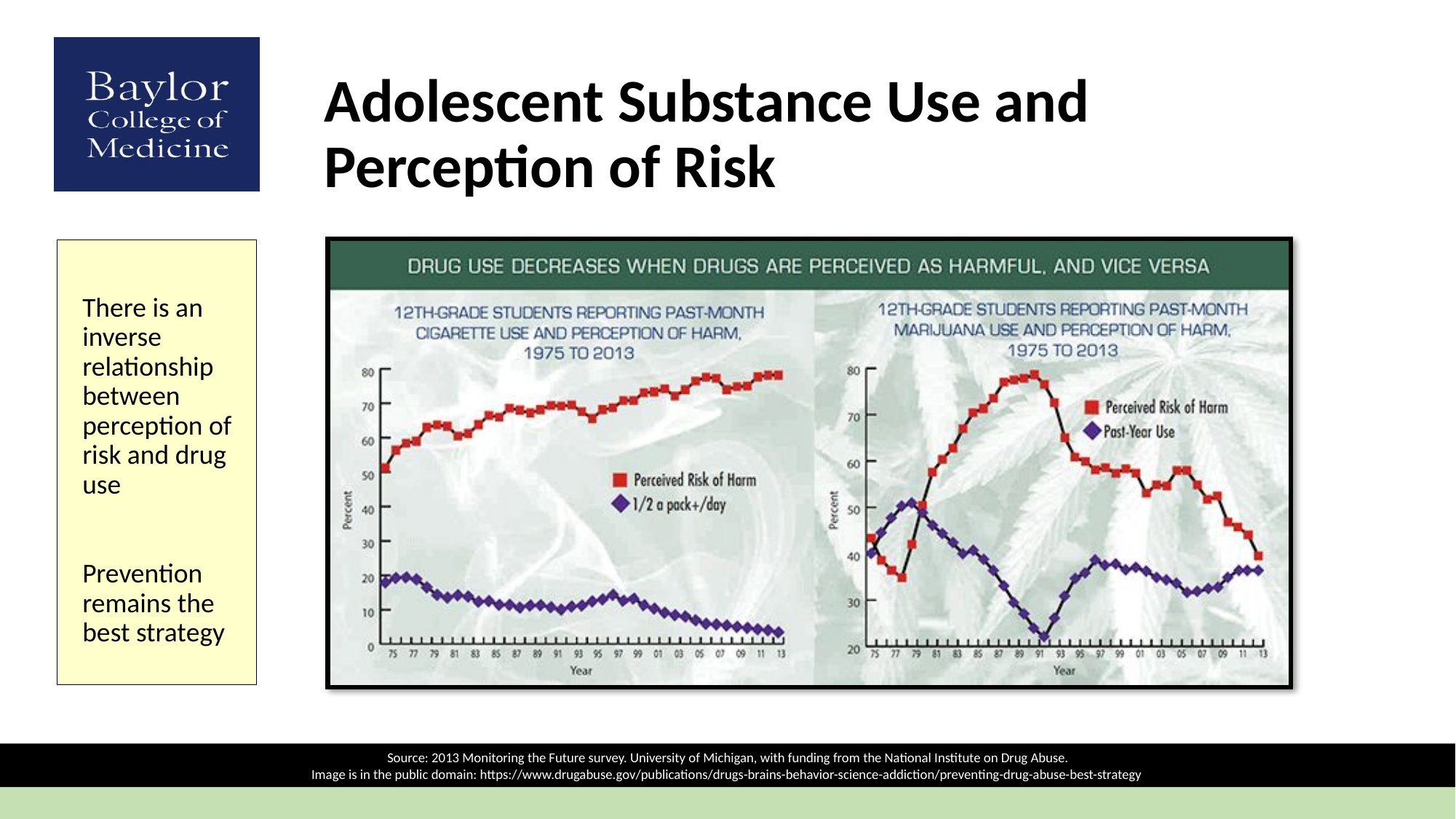

Adolescent Substance Use and Perception of Risk
There is an inverse relationship between perception of risk and drug use
Prevention remains the best strategy
Source: 2013 Monitoring the Future survey. University of Michigan, with funding from the National Institute on Drug Abuse.
Image is in the public domain: https://www.drugabuse.gov/publications/drugs-brains-behavior-science-addiction/preventing-drug-abuse-best-strategy

## Slide 42
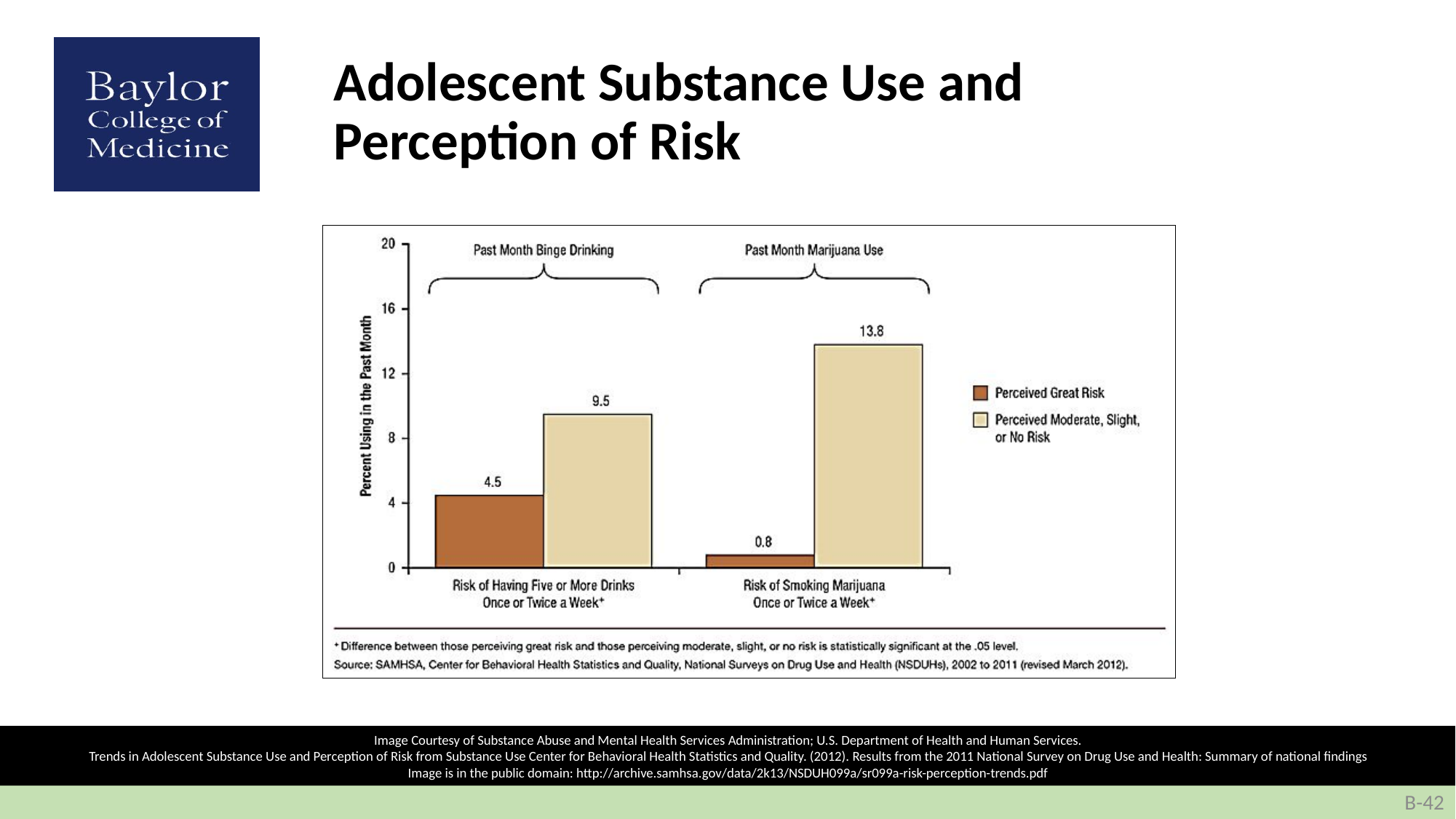

Adolescent Substance Use and Perception of Risk
Image Courtesy of Substance Abuse and Mental Health Services Administration; U.S. Department of Health and Human Services.
Trends in Adolescent Substance Use and Perception of Risk from Substance Use Center for Behavioral Health Statistics and Quality. (2012). Results from the 2011 National Survey on Drug Use and Health: Summary of national findings
Image is in the public domain: http://archive.samhsa.gov/data/2k13/NSDUH099a/sr099a-risk-perception-trends.pdf
B-42

## Slide 43
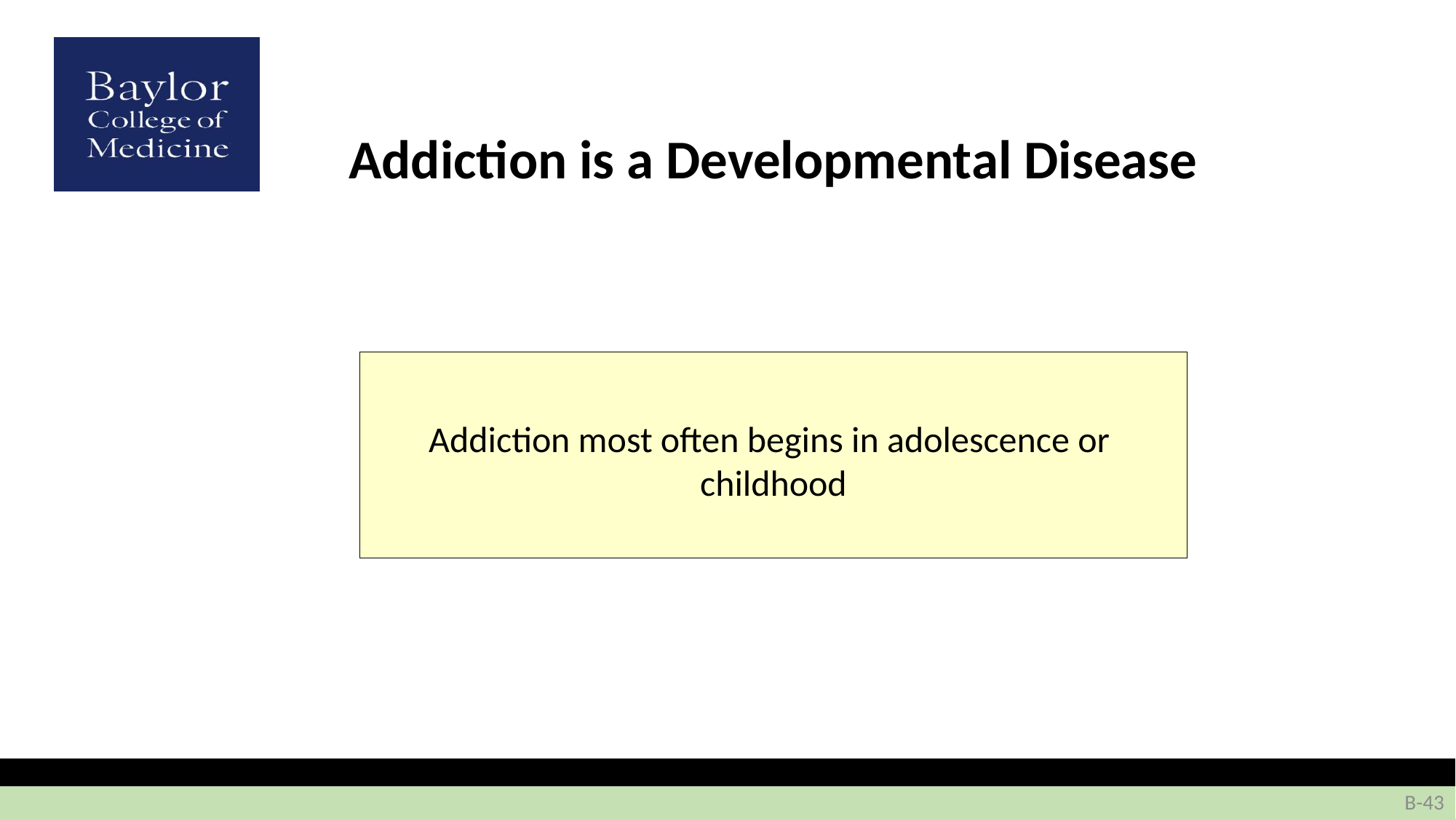

Addiction is a Developmental Disease
Addiction most often begins in adolescence or childhood
B-43

## Slide 44
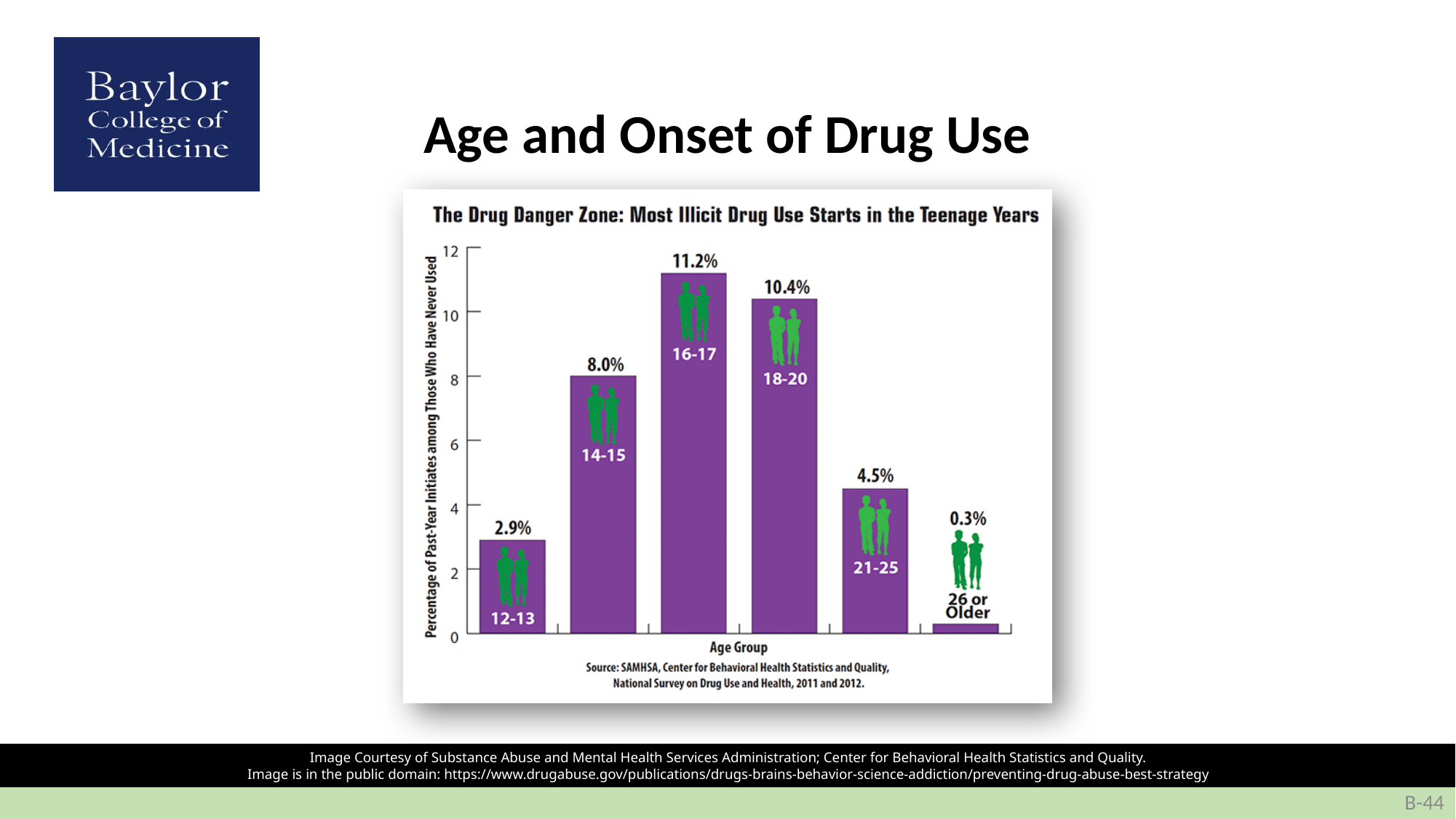

Age and Onset of Drug Use
Image Courtesy of Substance Abuse and Mental Health Services Administration; Center for Behavioral Health Statistics and Quality.
Image is in the public domain: https://www.drugabuse.gov/publications/drugs-brains-behavior-science-addiction/preventing-drug-abuse-best-strategy
B-44

## Slide 45
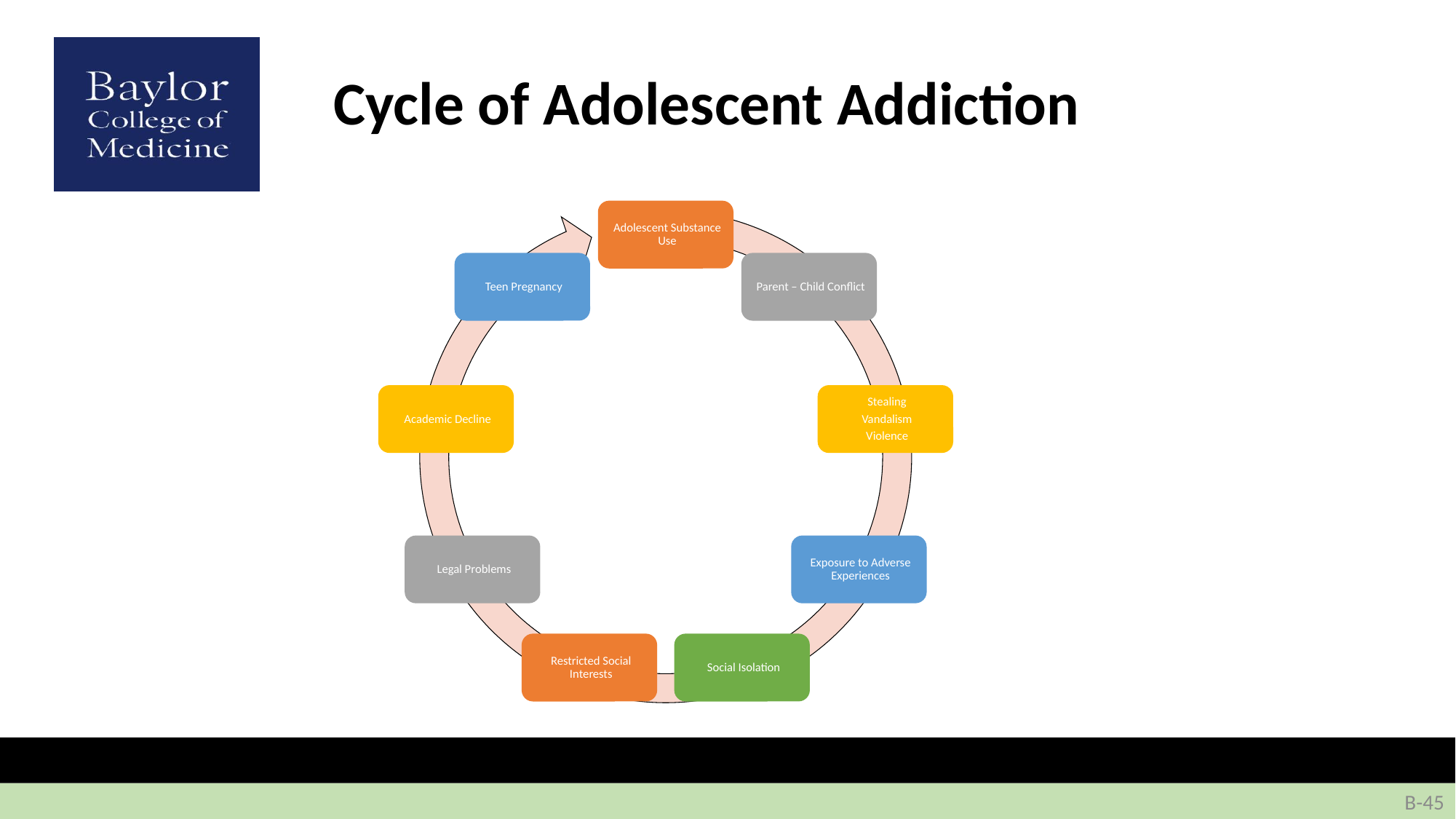

Cycle of Adolescent Addiction
B-45

## Slide 46
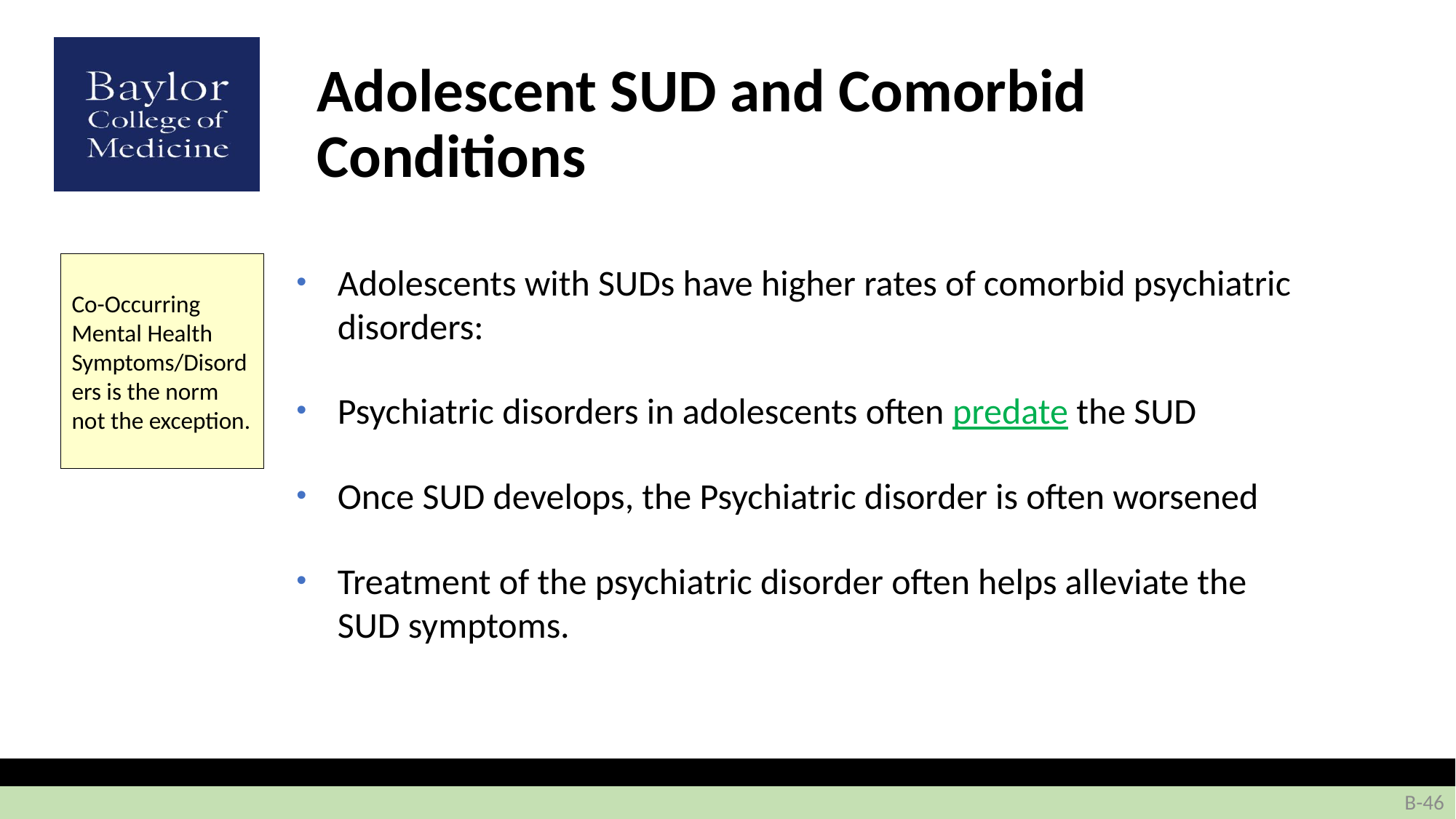

Adolescent SUD and Comorbid Conditions
Co-Occurring Mental Health Symptoms/Disorders is the norm not the exception.
Adolescents with SUDs have higher rates of comorbid psychiatric disorders:
Psychiatric disorders in adolescents often predate the SUD
Once SUD develops, the Psychiatric disorder is often worsened
Treatment of the psychiatric disorder often helps alleviate the SUD symptoms.
B-46

## Slide 47
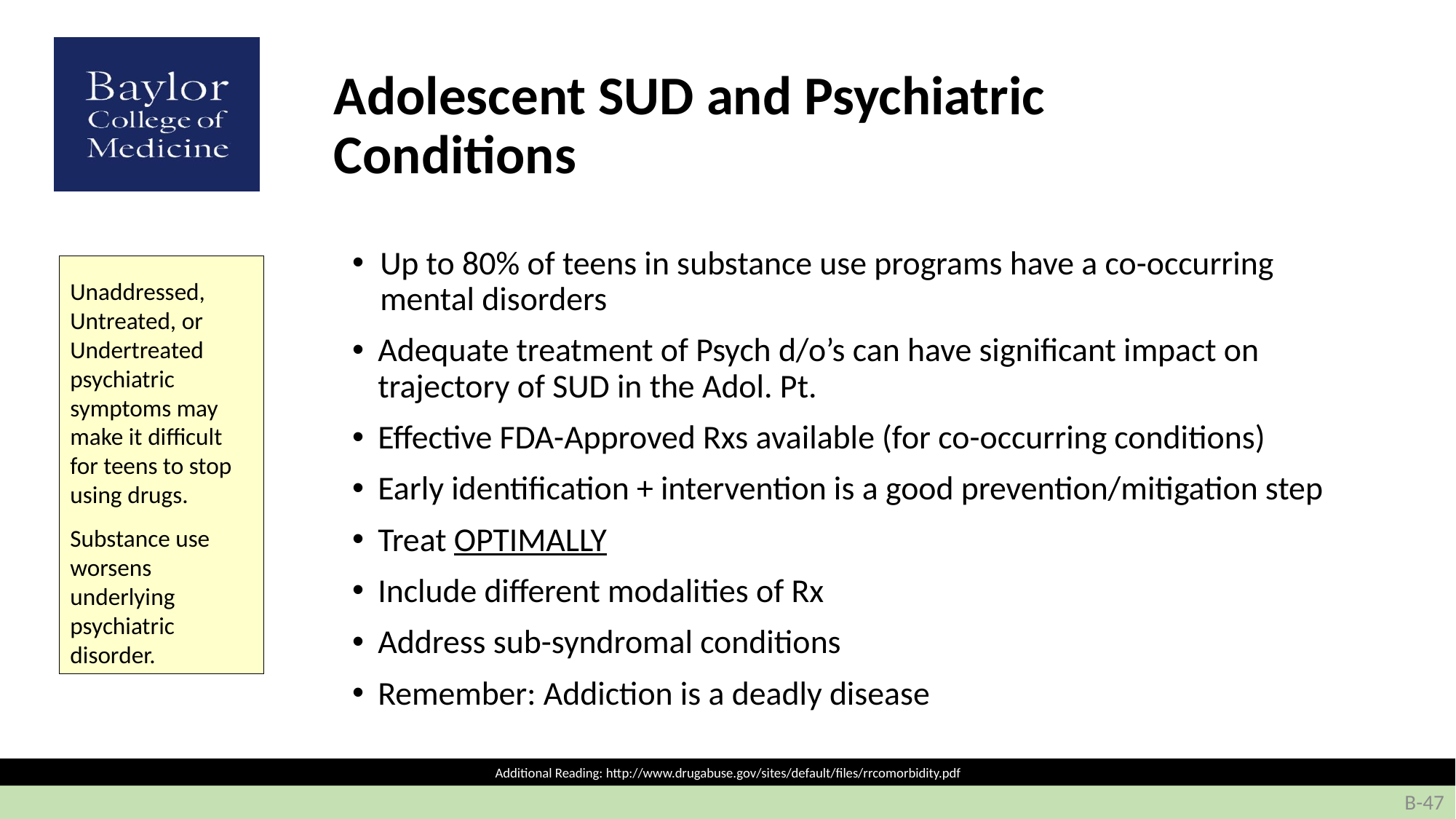

Adolescent SUD and Psychiatric Conditions
Up to 80% of teens in substance use programs have a co-occurring mental disorders
Adequate treatment of Psych d/o’s can have significant impact on trajectory of SUD in the Adol. Pt.
Effective FDA-Approved Rxs available (for co-occurring conditions)
Early identification + intervention is a good prevention/mitigation step
Treat OPTIMALLY
Include different modalities of Rx
Address sub-syndromal conditions
Remember: Addiction is a deadly disease
Unaddressed, Untreated, or Undertreated psychiatric symptoms may make it difficult for teens to stop using drugs.
Substance use worsens underlying psychiatric disorder.
Additional Reading: http://www.drugabuse.gov/sites/default/files/rrcomorbidity.pdf
B-47

## Slide 48
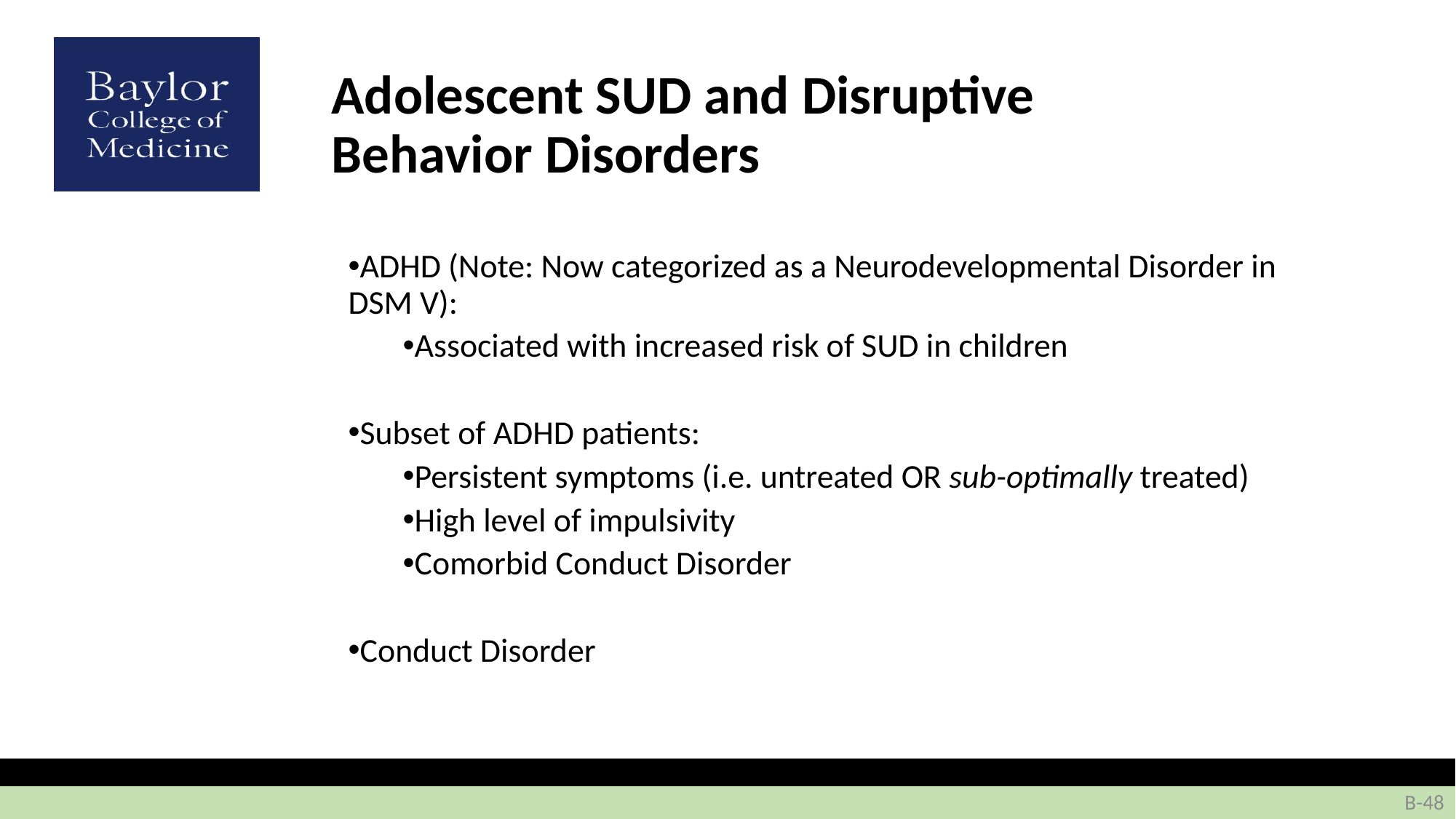

Adolescent SUD and Disruptive Behavior Disorders
ADHD (Note: Now categorized as a Neurodevelopmental Disorder in DSM V):
Associated with increased risk of SUD in children
Subset of ADHD patients:
Persistent symptoms (i.e. untreated OR sub-optimally treated)
High level of impulsivity
Comorbid Conduct Disorder
Conduct Disorder
B-48

## Slide 49
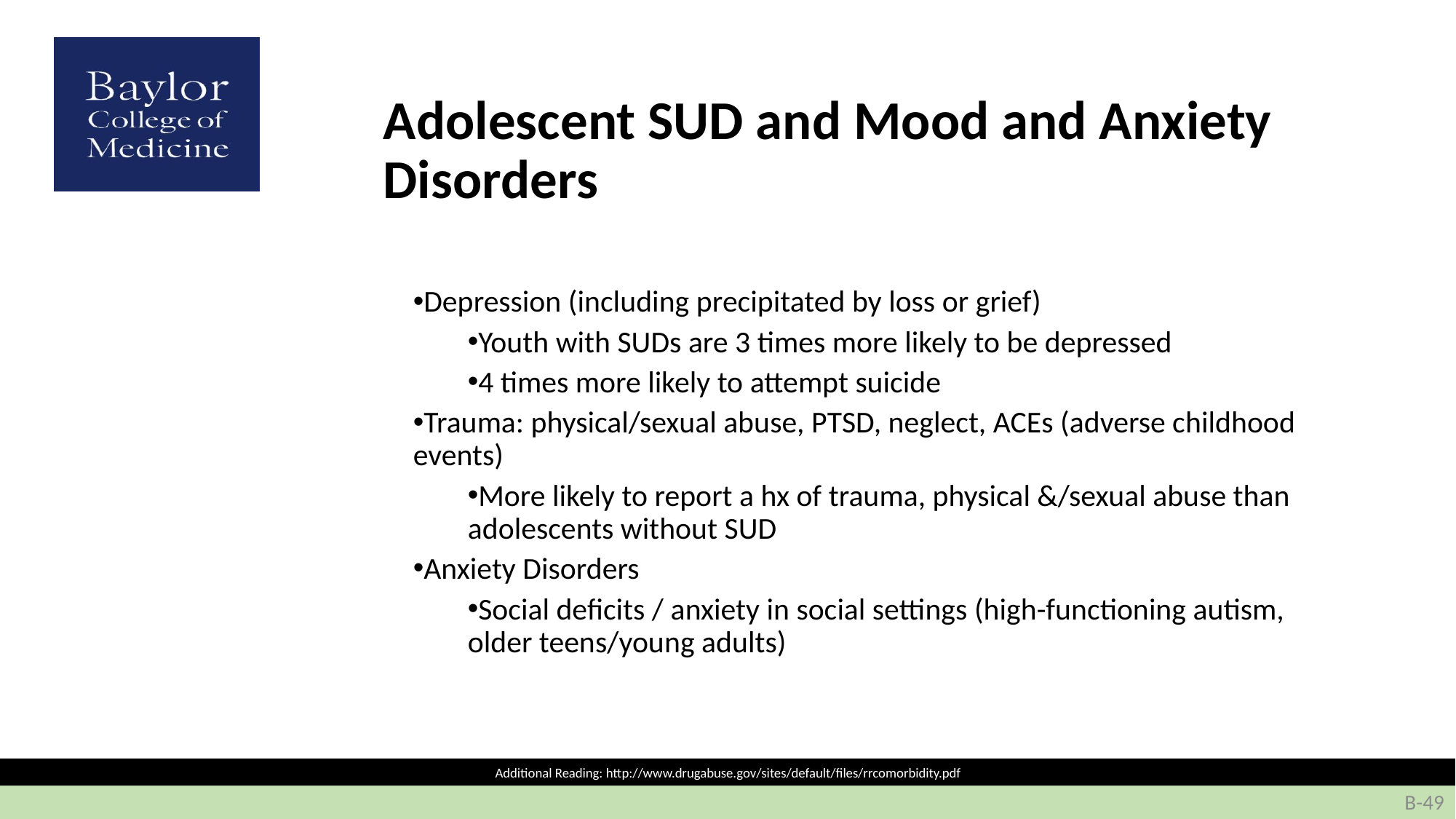

Adolescent SUD and Mood and Anxiety Disorders
Depression (including precipitated by loss or grief)
Youth with SUDs are 3 times more likely to be depressed
4 times more likely to attempt suicide
Trauma: physical/sexual abuse, PTSD, neglect, ACEs (adverse childhood events)
More likely to report a hx of trauma, physical &/sexual abuse than adolescents without SUD
Anxiety Disorders
Social deficits / anxiety in social settings (high-functioning autism, older teens/young adults)
Additional Reading: http://www.drugabuse.gov/sites/default/files/rrcomorbidity.pdf
B-49

## Slide 50
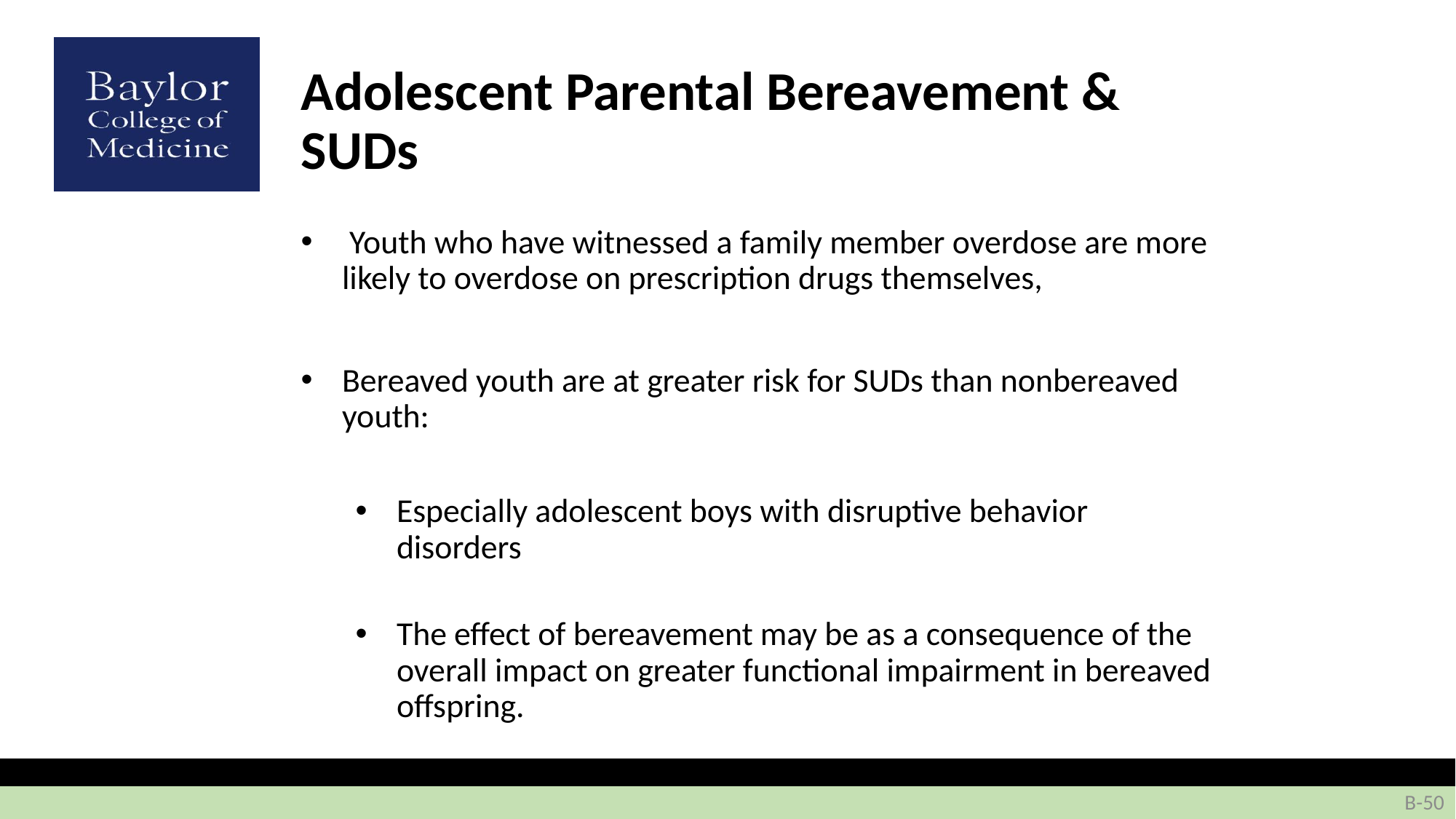

Adolescent Parental Bereavement & SUDs
 Youth who have witnessed a family member overdose are more likely to overdose on prescription drugs themselves,
Bereaved youth are at greater risk for SUDs than nonbereaved youth:
Especially adolescent boys with disruptive behavior disorders
The effect of bereavement may be as a consequence of the overall impact on greater functional impairment in bereaved offspring.
B-50

## Slide 51
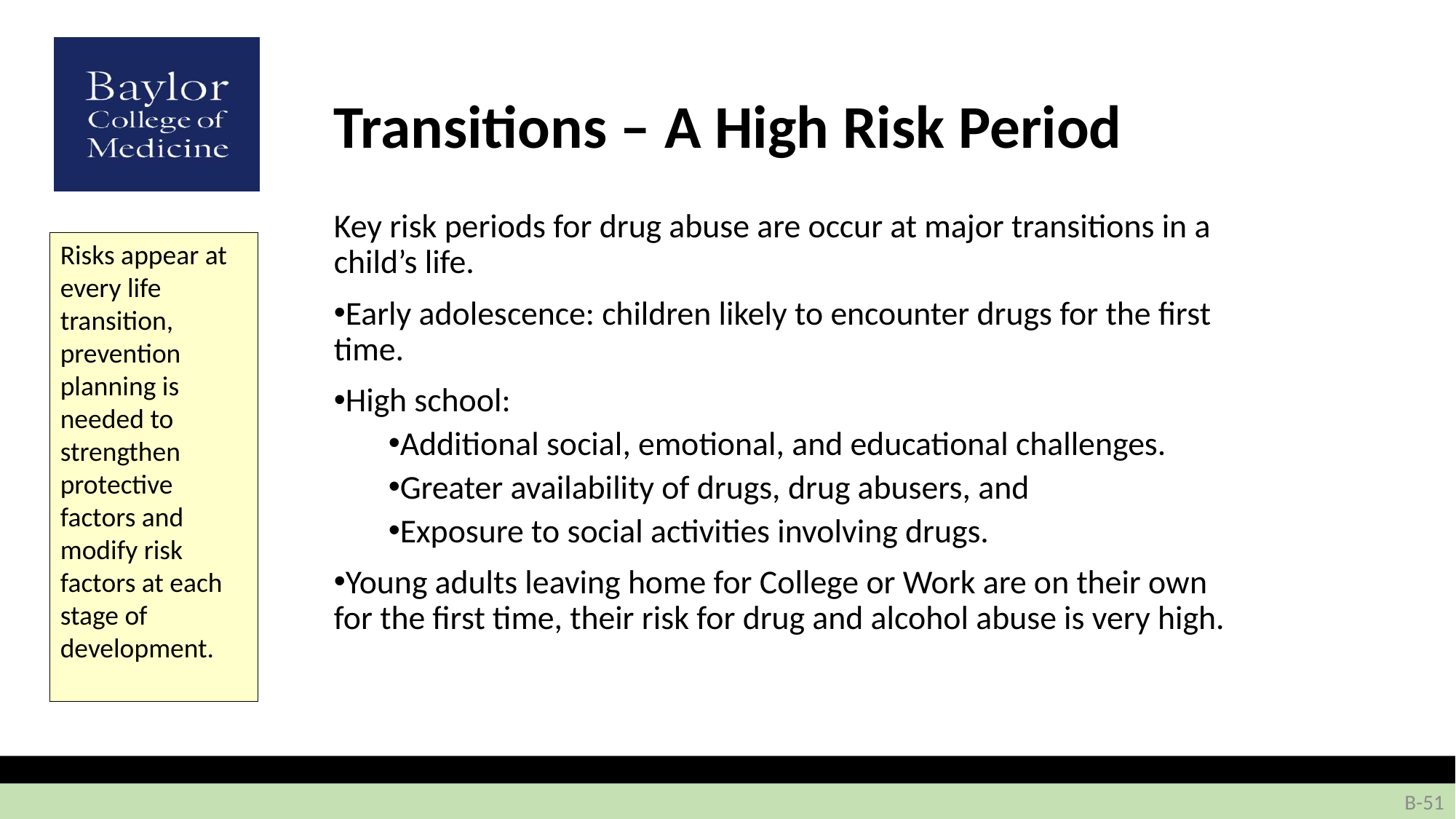

Transitions – A High Risk Period
Key risk periods for drug abuse are occur at major transitions in a child’s life.
Early adolescence: children likely to encounter drugs for the first time.
High school:
Additional social, emotional, and educational challenges.
Greater availability of drugs, drug abusers, and
Exposure to social activities involving drugs.
Young adults leaving home for College or Work are on their own for the first time, their risk for drug and alcohol abuse is very high.
Risks appear at every life transition, prevention planning is needed to strengthen protective factors and modify risk factors at each stage of development.
B-51

## Slide 52
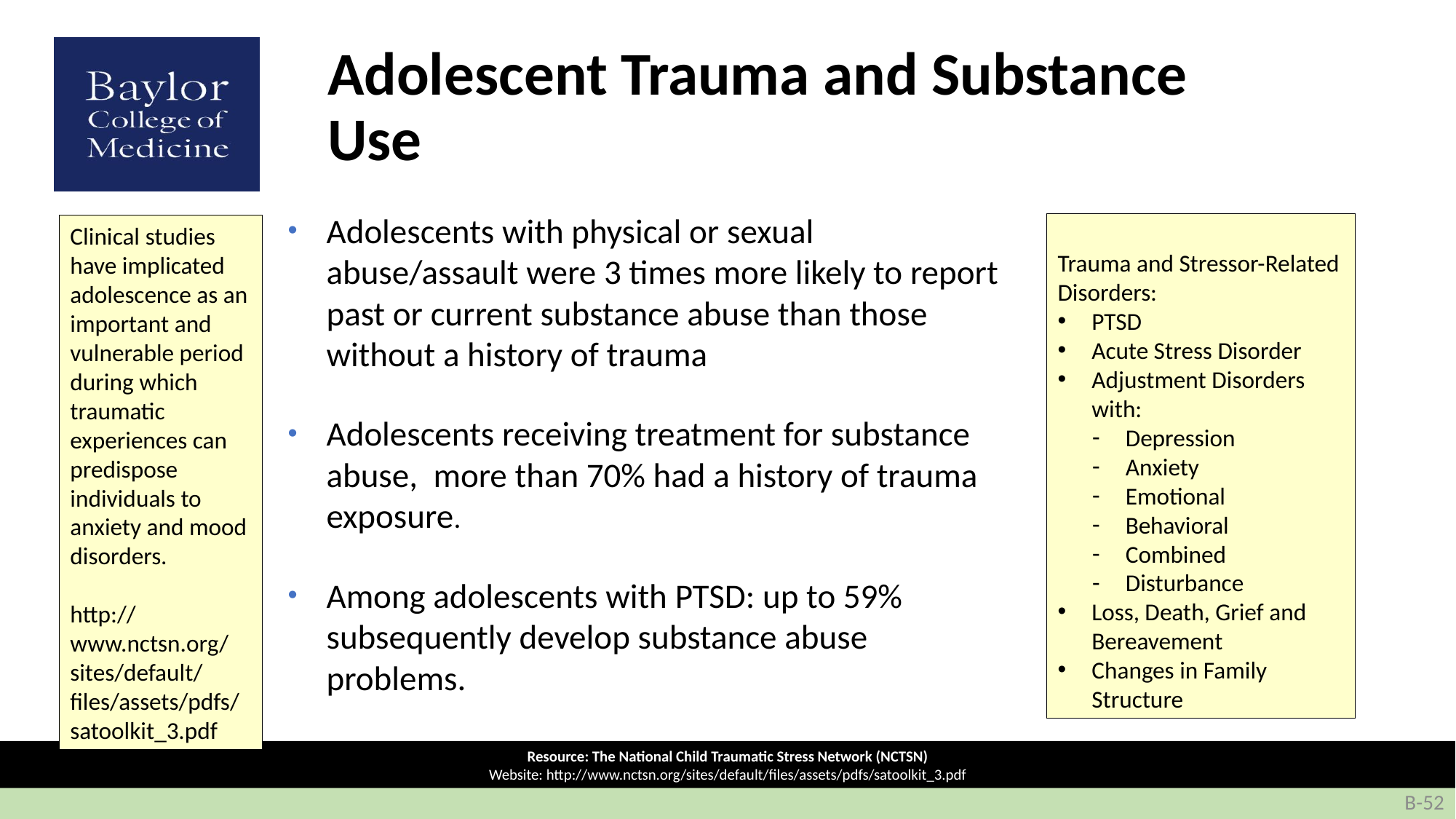

Adolescent Trauma and Substance Use
Adolescents with physical or sexual abuse/assault were 3 times more likely to report past or current substance abuse than those without a history of trauma
Adolescents receiving treatment for substance abuse, more than 70% had a history of trauma exposure.
Among adolescents with PTSD: up to 59% subsequently develop substance abuse problems.
Trauma and Stressor-Related Disorders:
PTSD
Acute Stress Disorder
Adjustment Disorders with:
Depression
Anxiety
Emotional
Behavioral
Combined
Disturbance
Loss, Death, Grief and Bereavement
Changes in Family Structure
Clinical studies have implicated adolescence as an important and vulnerable period during which traumatic experiences can predispose individuals to anxiety and mood disorders.
http://www.nctsn.org/sites/default/files/assets/pdfs/satoolkit_3.pdf
Resource: The National Child Traumatic Stress Network (NCTSN)
Website: http://www.nctsn.org/sites/default/files/assets/pdfs/satoolkit_3.pdf
B-52

## Slide 53
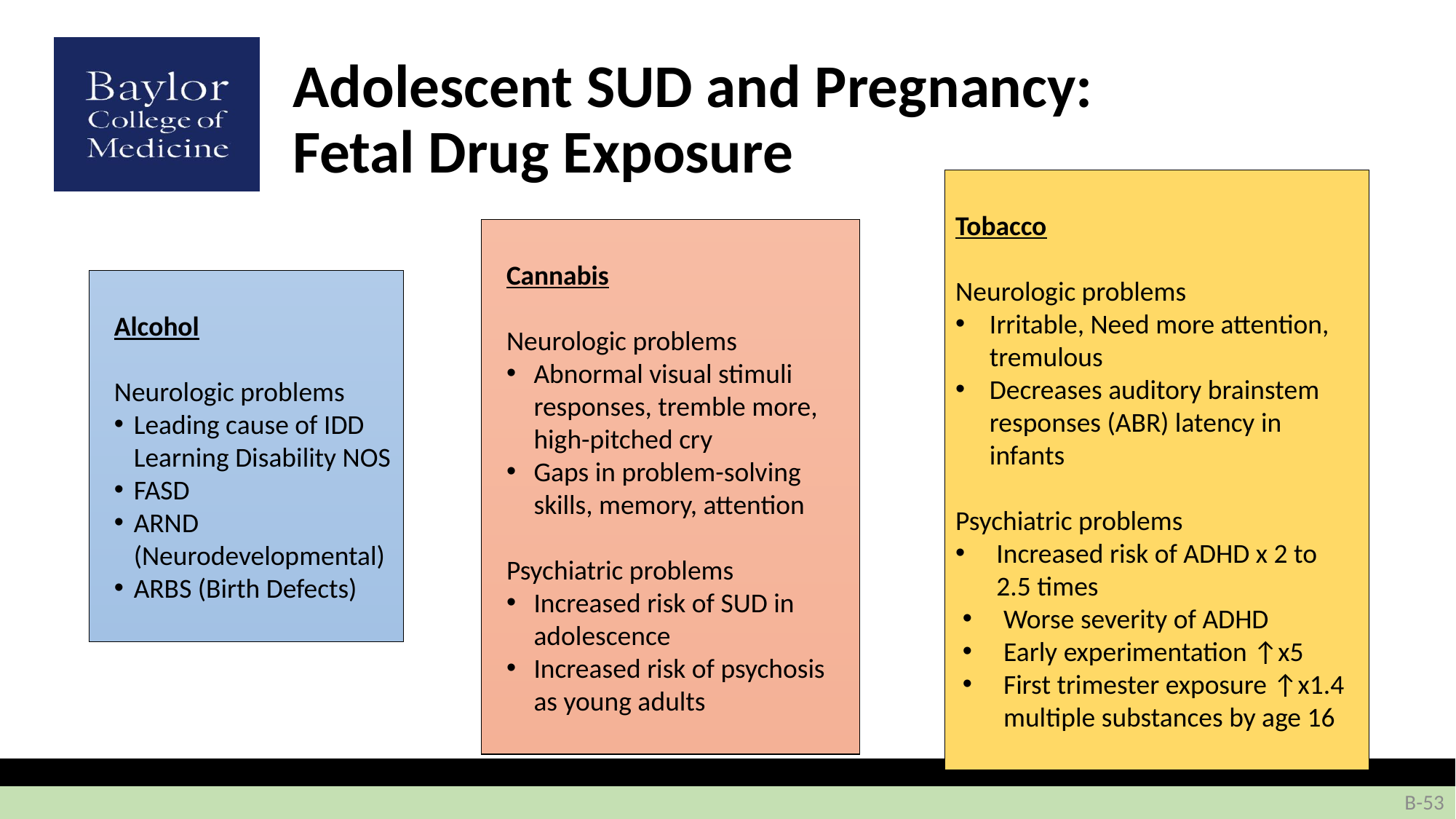

Adolescent SUD and Pregnancy:
Fetal Drug Exposure
Tobacco
Neurologic problems
Irritable, Need more attention, tremulous
Decreases auditory brainstem responses (ABR) latency in infants
Psychiatric problems
Increased risk of ADHD x 2 to 2.5 times
Worse severity of ADHD
Early experimentation ↑x5
First trimester exposure ↑x1.4 multiple substances by age 16
Cannabis
Neurologic problems
Abnormal visual stimuli responses, tremble more, high-pitched cry
Gaps in problem-solving skills, memory, attention
Psychiatric problems
Increased risk of SUD in adolescence
Increased risk of psychosis as young adults
Alcohol
Neurologic problems
Leading cause of IDD Learning Disability NOS
FASD
ARND (Neurodevelopmental)
ARBS (Birth Defects)
B-53

## Slide 54
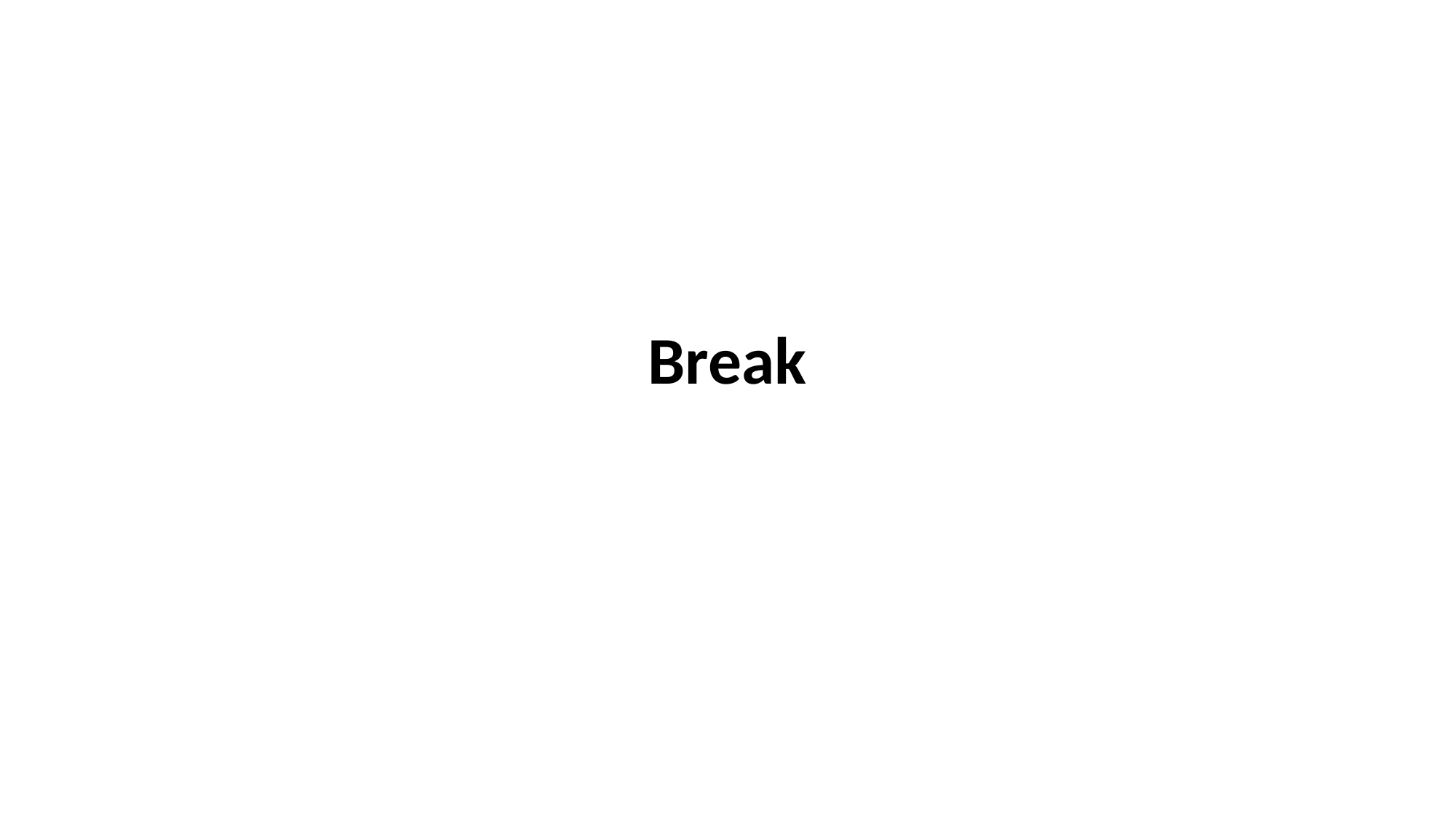

# Break

## Slide 55
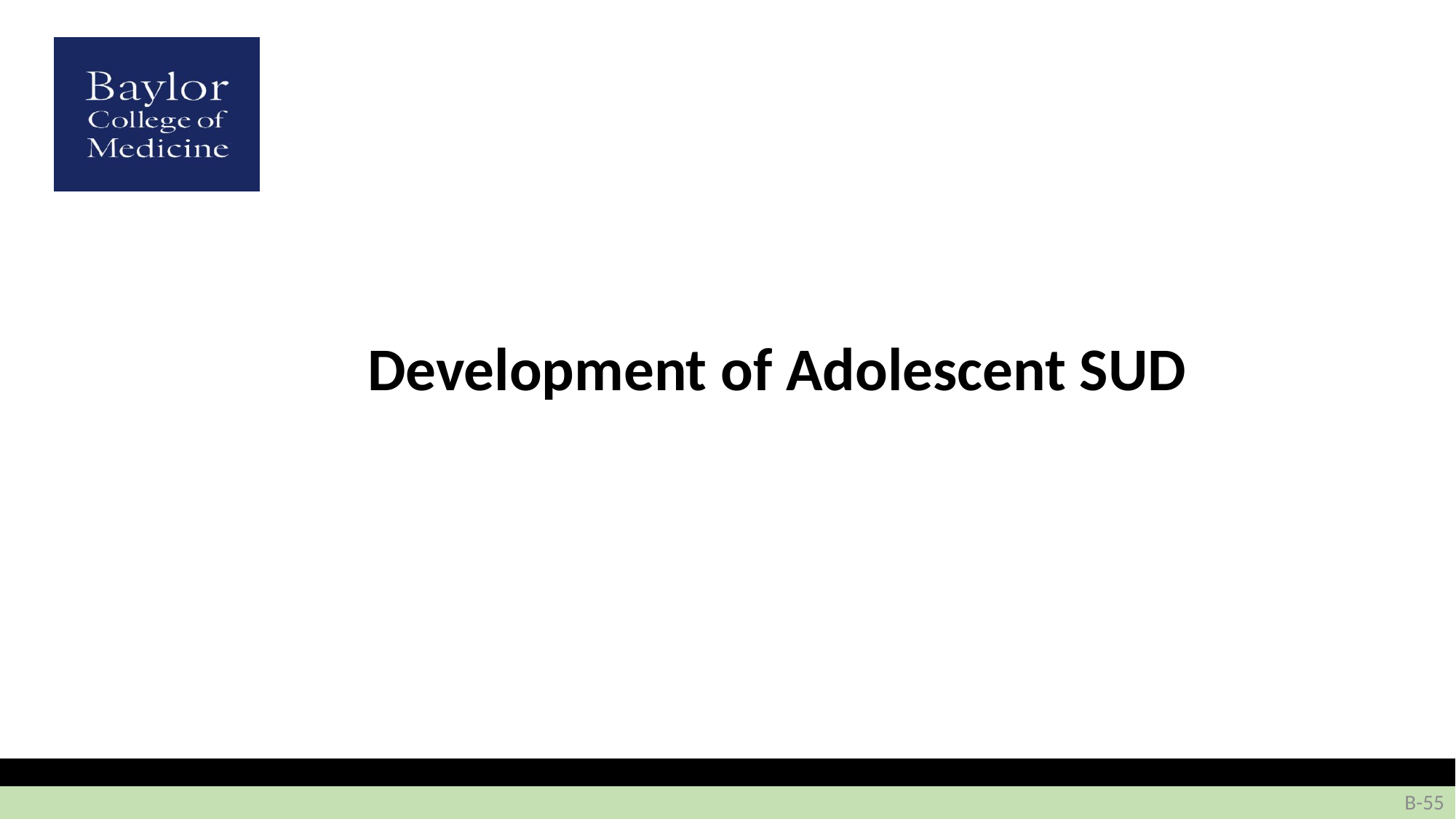

Development of Adolescent SUD
B-55

## Slide 56
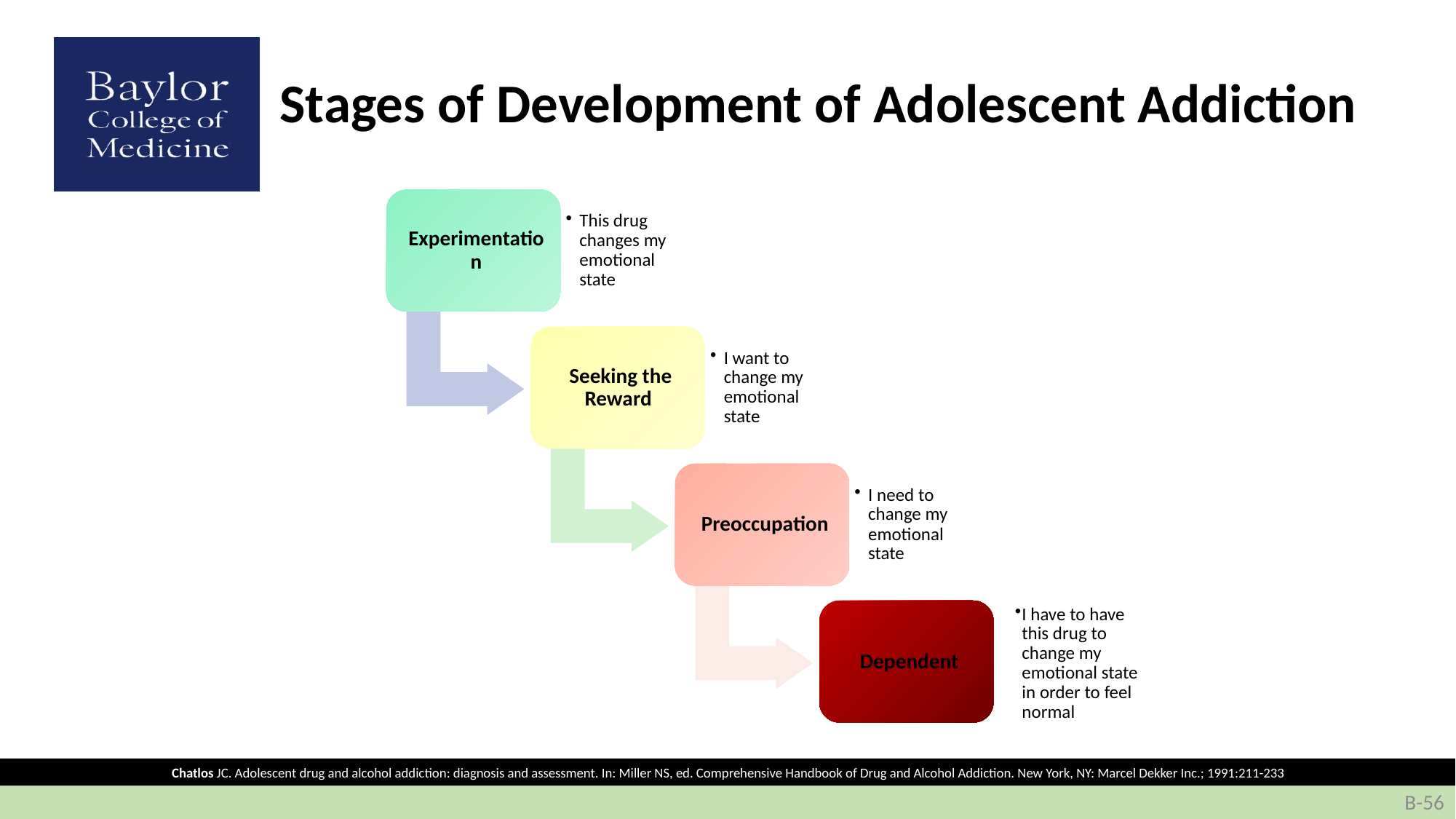

Stages of Development of Adolescent Addiction
I have to have this drug to change my emotional state in order to feel normal
Chatlos JC. Adolescent drug and alcohol addiction: diagnosis and assessment. In: Miller NS, ed. Comprehensive Handbook of Drug and Alcohol Addiction. New York, NY: Marcel Dekker Inc.; 1991:211-233
B-56

## Slide 57
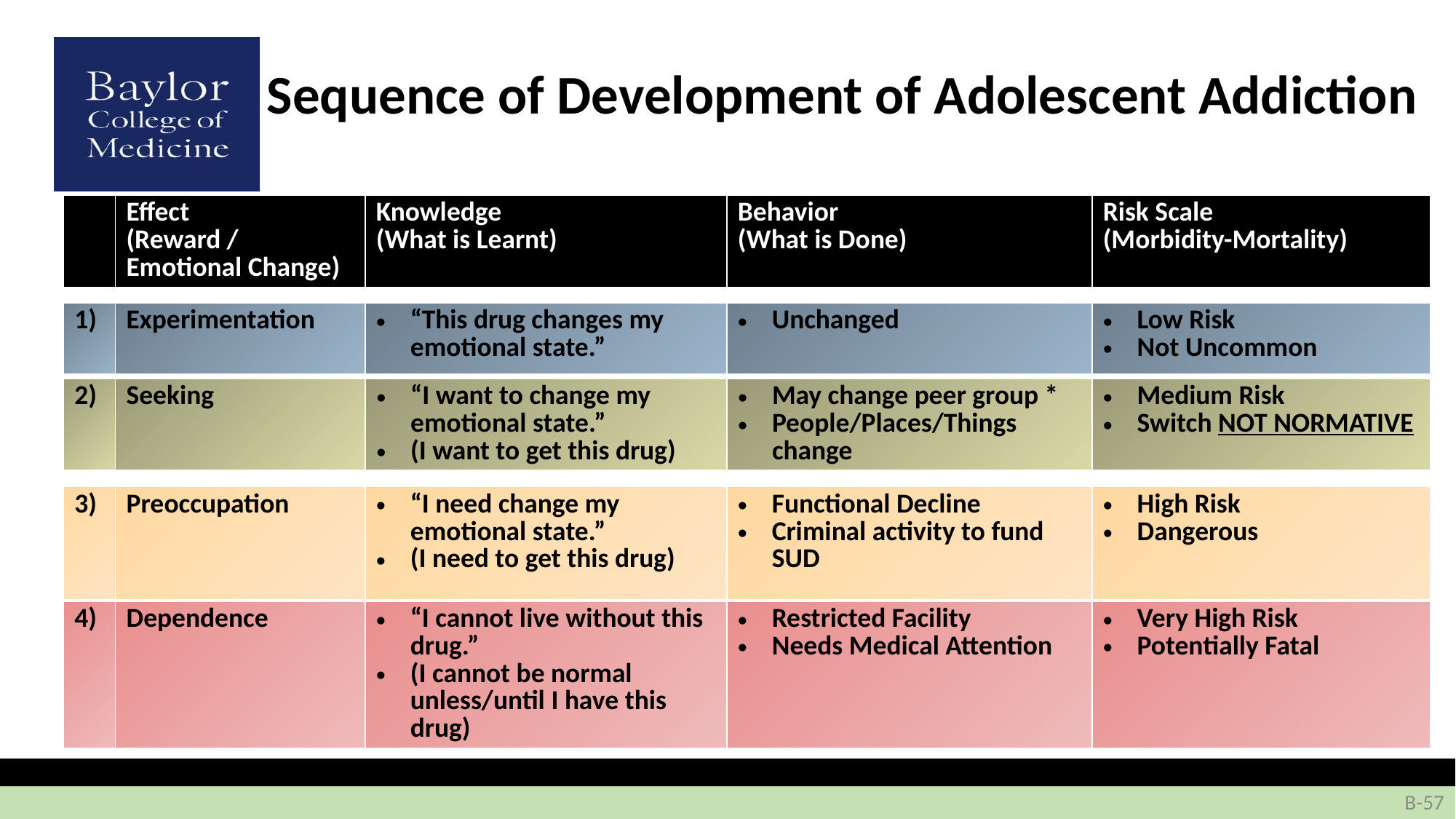

Sequence of Development of Adolescent Addiction
| | Effect (Reward / Emotional Change) | Knowledge (What is Learnt) | Behavior (What is Done) | Risk Scale (Morbidity-Mortality) |
| --- | --- | --- | --- | --- |
| 1) | Experimentation | “This drug changes my emotional state.” | Unchanged | Low Risk Not Uncommon |
| --- | --- | --- | --- | --- |
| 2) | Seeking | “I want to change my emotional state.” (I want to get this drug) | May change peer group \* People/Places/Things change | Medium Risk Switch NOT NORMATIVE |
| --- | --- | --- | --- | --- |
| 3) | Preoccupation | “I need change my emotional state.” (I need to get this drug) | Functional Decline Criminal activity to fund SUD | High Risk Dangerous |
| --- | --- | --- | --- | --- |
| 4) | Dependence | “I cannot live without this drug.” (I cannot be normal unless/until I have this drug) | Restricted Facility Needs Medical Attention | Very High Risk Potentially Fatal |
| --- | --- | --- | --- | --- |
B-57

## Slide 58
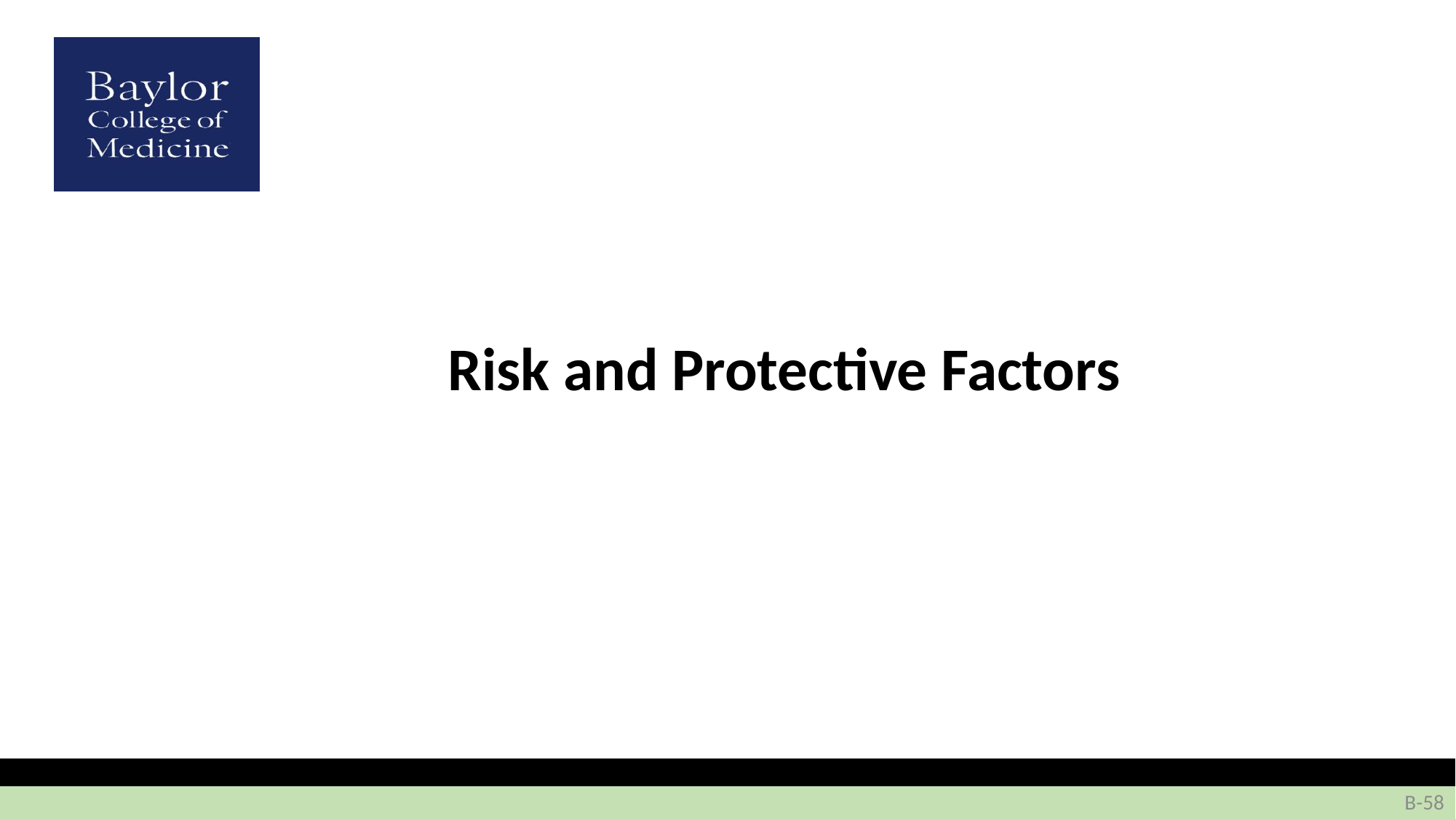

Risk and Protective Factors
B-58

## Slide 59
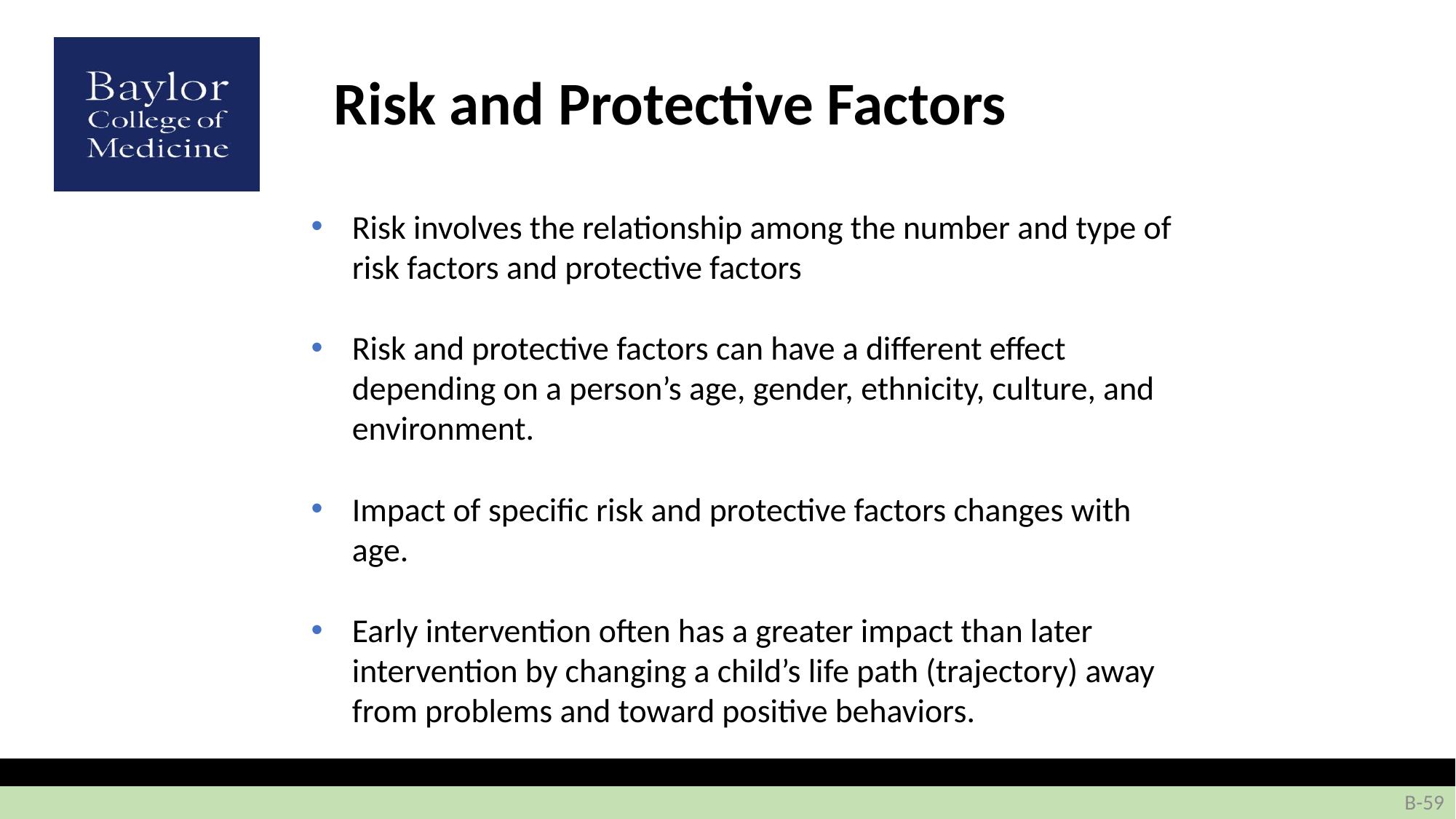

Risk and Protective Factors
Risk involves the relationship among the number and type of risk factors and protective factors
Risk and protective factors can have a different effect depending on a person’s age, gender, ethnicity, culture, and environment.
Impact of specific risk and protective factors changes with age.
Early intervention often has a greater impact than later intervention by changing a child’s life path (trajectory) away from problems and toward positive behaviors.
B-59

## Slide 60
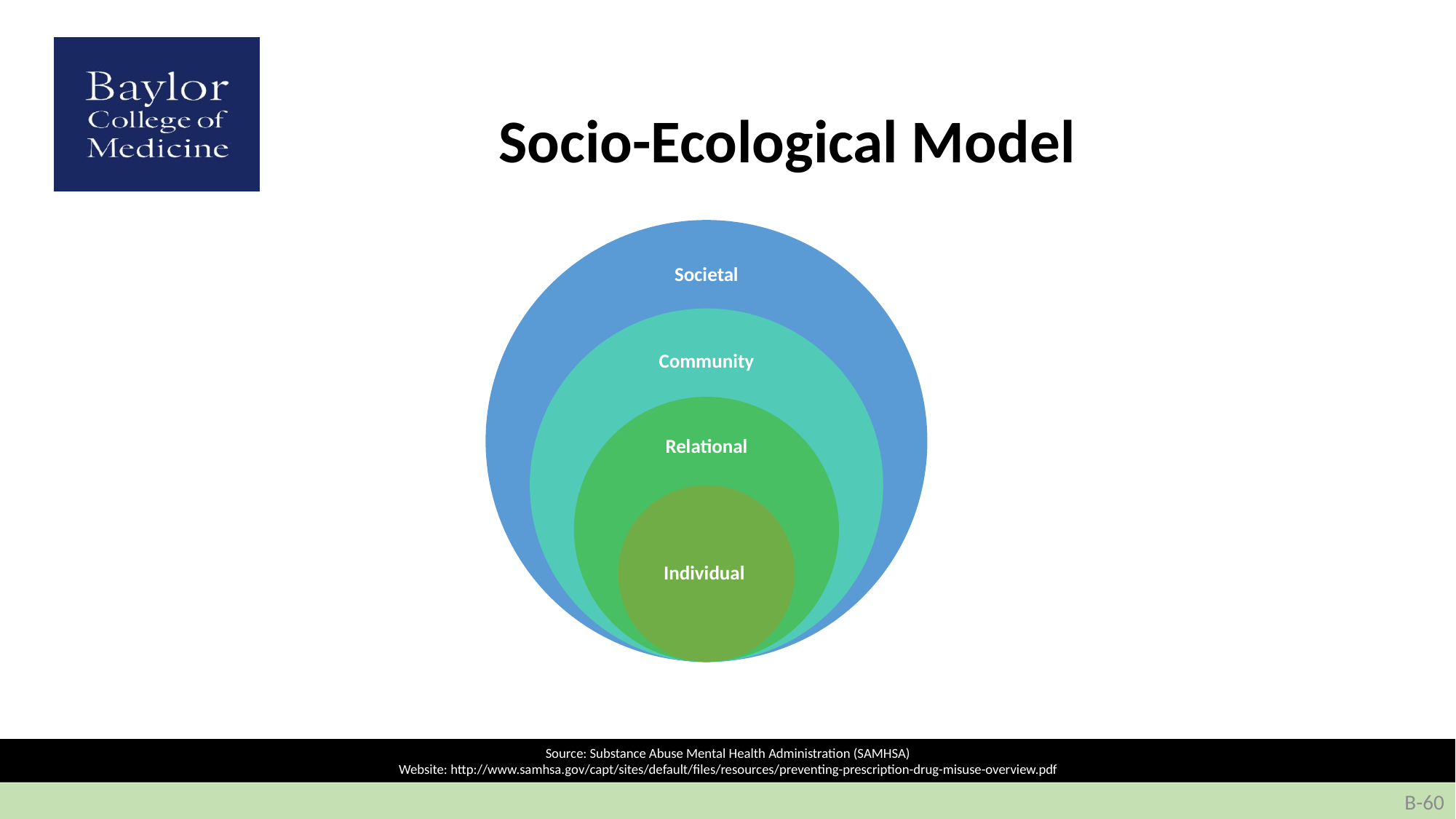

Socio-Ecological Model
Source: Substance Abuse Mental Health Administration (SAMHSA)
Website: http://www.samhsa.gov/capt/sites/default/files/resources/preventing-prescription-drug-misuse-overview.pdf
B-60

## Slide 61
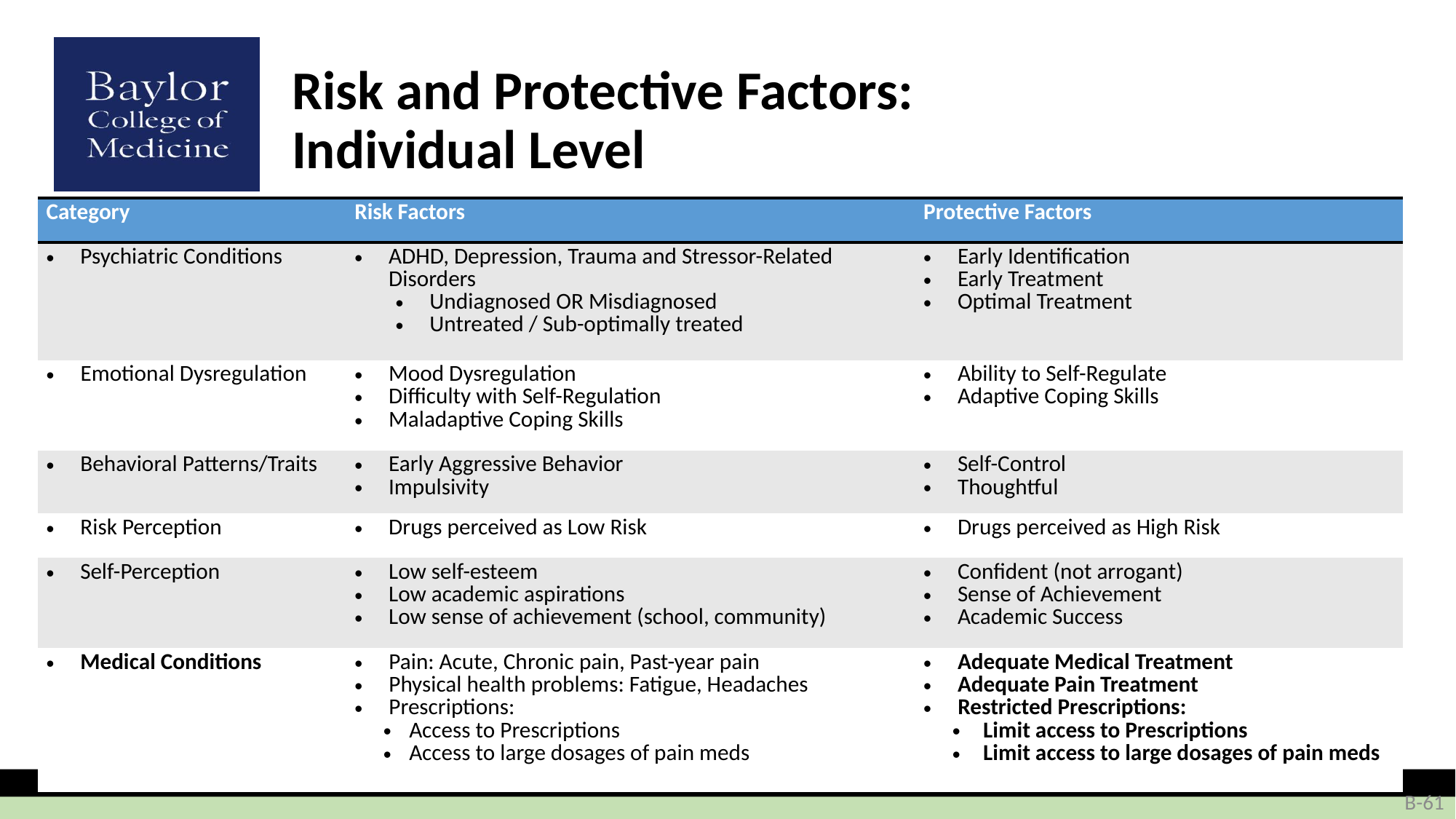

Risk and Protective Factors:
Individual Level
| Category | Risk Factors | Protective Factors |
| --- | --- | --- |
| Psychiatric Conditions | ADHD, Depression, Trauma and Stressor-Related Disorders Undiagnosed OR Misdiagnosed Untreated / Sub-optimally treated | Early Identification Early Treatment Optimal Treatment |
| Emotional Dysregulation | Mood Dysregulation Difficulty with Self-Regulation Maladaptive Coping Skills | Ability to Self-Regulate Adaptive Coping Skills |
| Behavioral Patterns/Traits | Early Aggressive Behavior Impulsivity | Self-Control Thoughtful |
| Risk Perception | Drugs perceived as Low Risk | Drugs perceived as High Risk |
| Self-Perception | Low self-esteem Low academic aspirations Low sense of achievement (school, community) | Confident (not arrogant) Sense of Achievement Academic Success |
| Medical Conditions | Pain: Acute, Chronic pain, Past-year pain Physical health problems: Fatigue, Headaches Prescriptions: Access to Prescriptions Access to large dosages of pain meds | Adequate Medical Treatment Adequate Pain Treatment Restricted Prescriptions: Limit access to Prescriptions Limit access to large dosages of pain meds |
B-61

## Slide 62
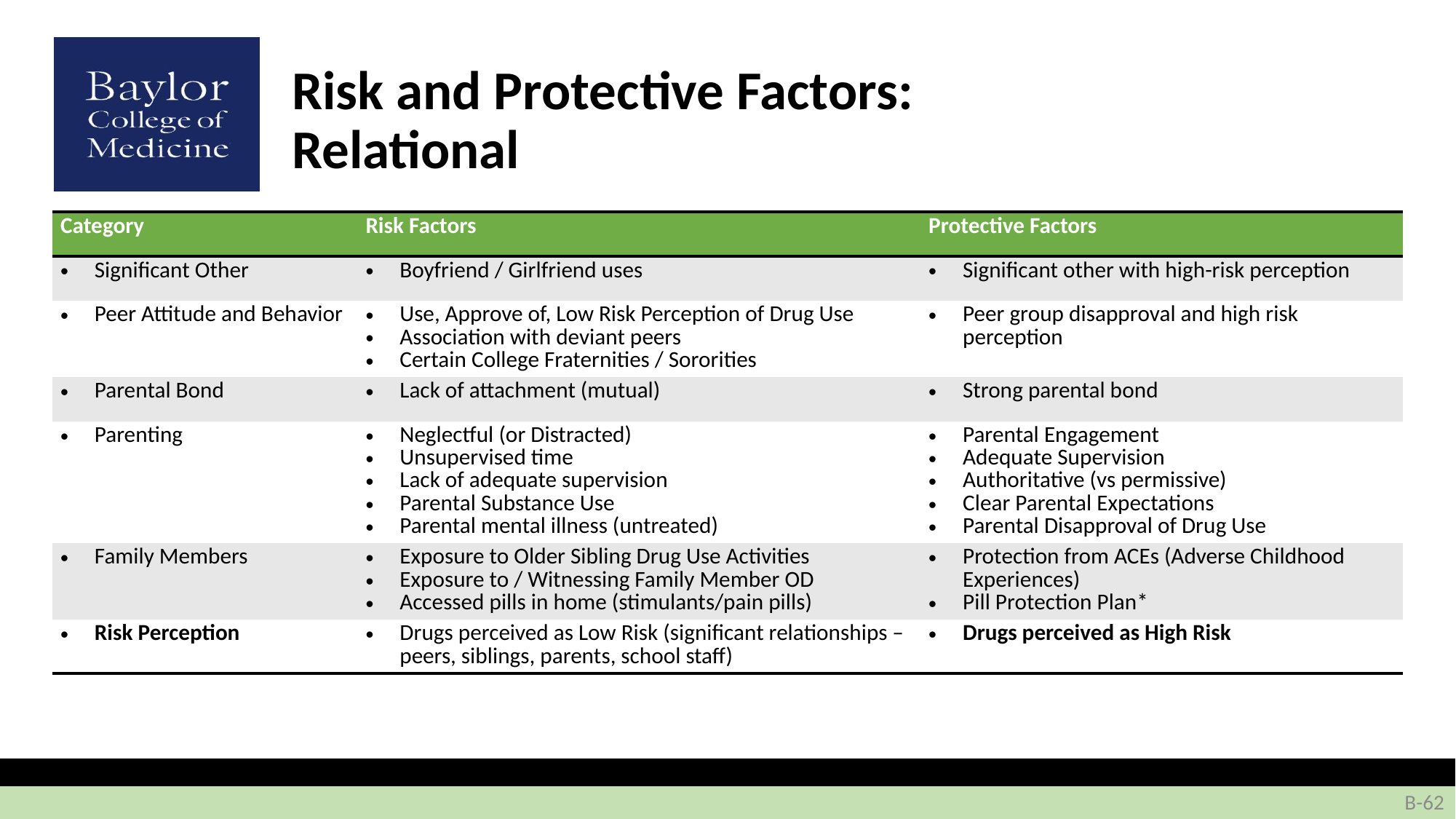

Risk and Protective Factors:
Relational
| Category | Risk Factors | Protective Factors |
| --- | --- | --- |
| Significant Other | Boyfriend / Girlfriend uses | Significant other with high-risk perception |
| Peer Attitude and Behavior | Use, Approve of, Low Risk Perception of Drug Use Association with deviant peers Certain College Fraternities / Sororities | Peer group disapproval and high risk perception |
| Parental Bond | Lack of attachment (mutual) | Strong parental bond |
| Parenting | Neglectful (or Distracted) Unsupervised time Lack of adequate supervision Parental Substance Use Parental mental illness (untreated) | Parental Engagement Adequate Supervision Authoritative (vs permissive) Clear Parental Expectations Parental Disapproval of Drug Use |
| Family Members | Exposure to Older Sibling Drug Use Activities Exposure to / Witnessing Family Member OD Accessed pills in home (stimulants/pain pills) | Protection from ACEs (Adverse Childhood Experiences) Pill Protection Plan\* |
| Risk Perception | Drugs perceived as Low Risk (significant relationships – peers, siblings, parents, school staff) | Drugs perceived as High Risk |
B-62

## Slide 63
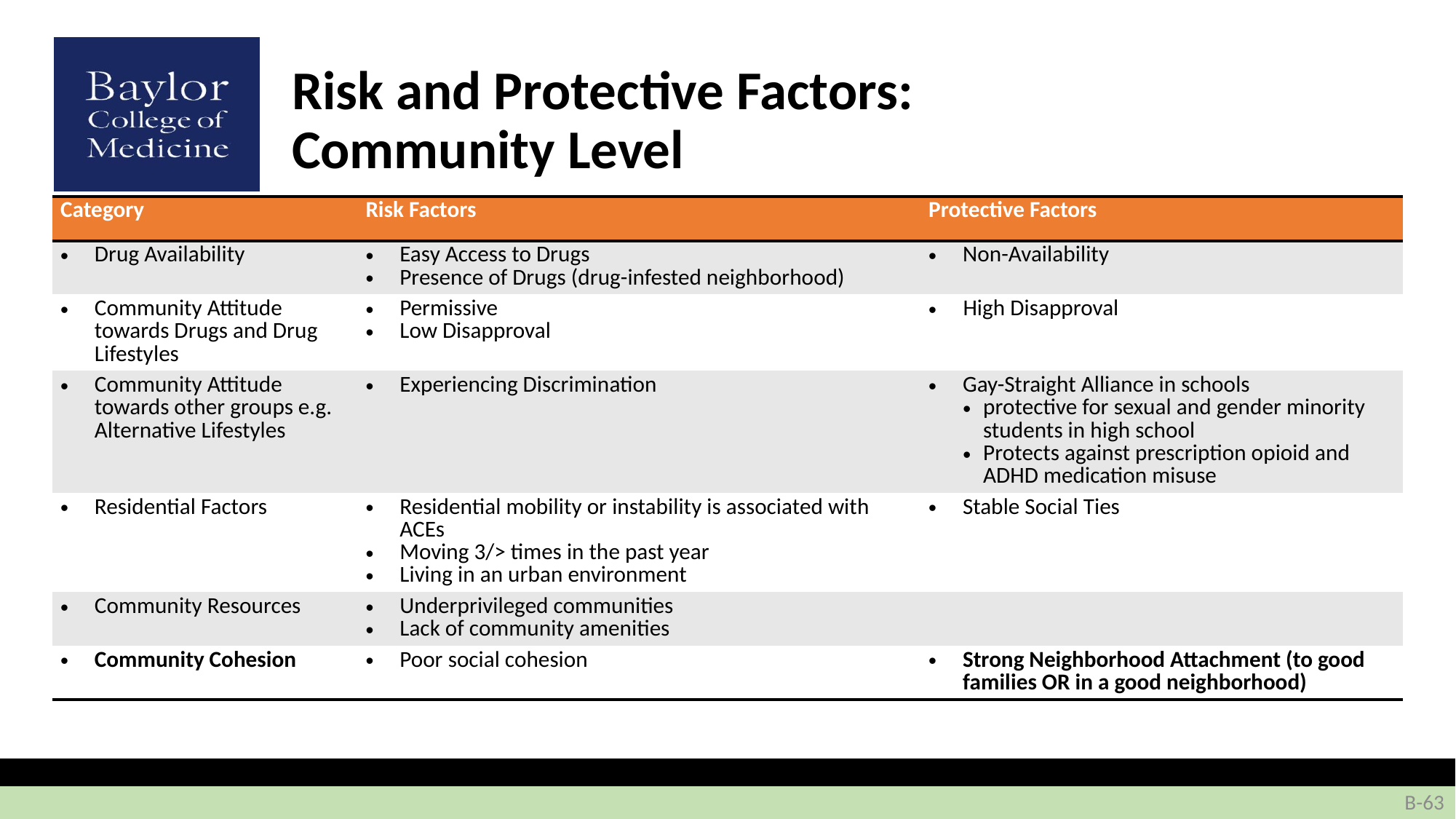

Risk and Protective Factors:
Community Level
| Category | Risk Factors | Protective Factors |
| --- | --- | --- |
| Drug Availability | Easy Access to Drugs Presence of Drugs (drug-infested neighborhood) | Non-Availability |
| Community Attitude towards Drugs and Drug Lifestyles | Permissive Low Disapproval | High Disapproval |
| Community Attitude towards other groups e.g. Alternative Lifestyles | Experiencing Discrimination | Gay-Straight Alliance in schools protective for sexual and gender minority students in high school Protects against prescription opioid and ADHD medication misuse |
| Residential Factors | Residential mobility or instability is associated with ACEs Moving 3/> times in the past year Living in an urban environment | Stable Social Ties |
| Community Resources | Underprivileged communities Lack of community amenities | |
| Community Cohesion | Poor social cohesion | Strong Neighborhood Attachment (to good families OR in a good neighborhood) |
B-63

## Slide 64
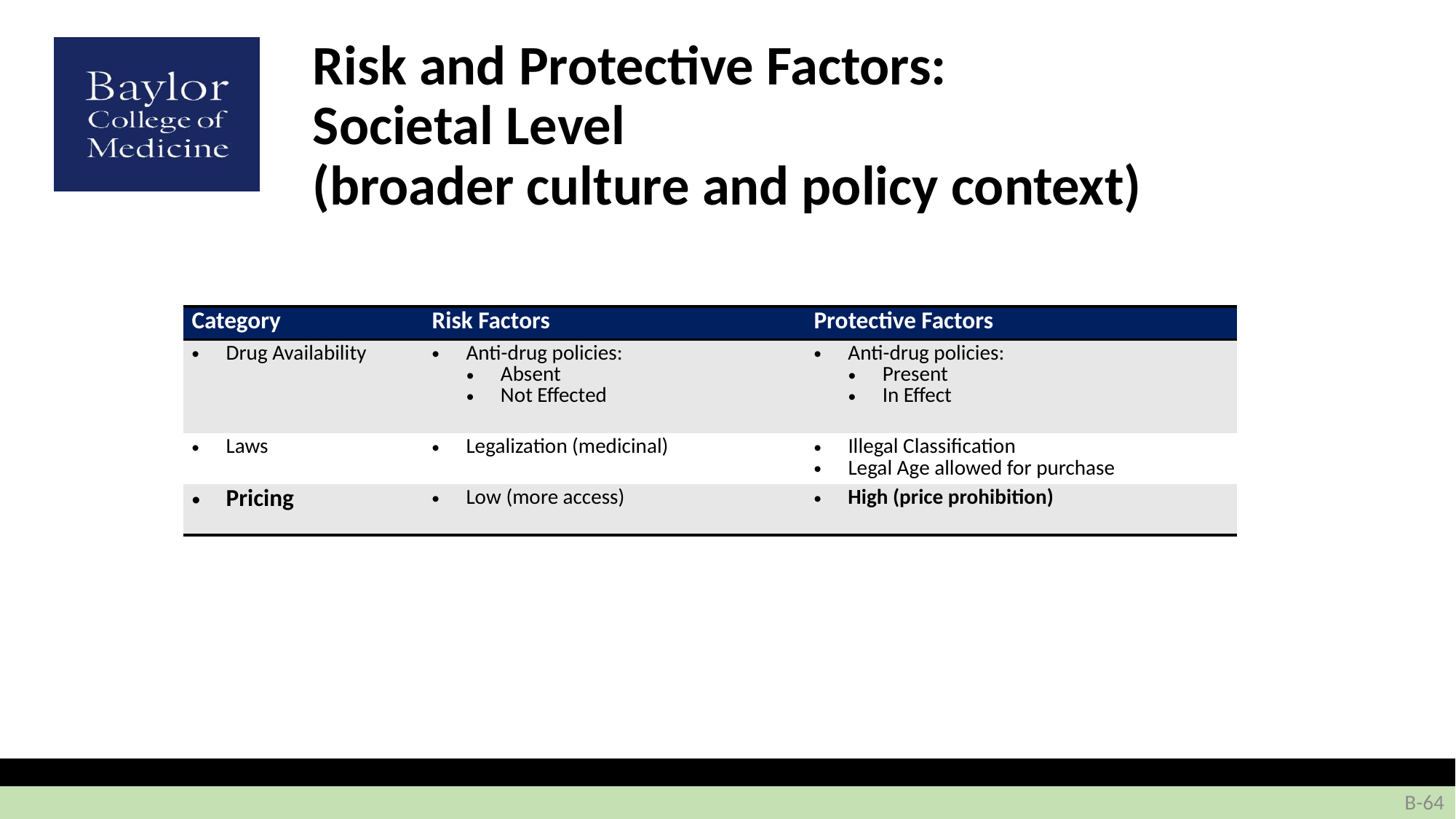

Risk and Protective Factors:
Societal Level
(broader culture and policy context)
| Category | Risk Factors | Protective Factors |
| --- | --- | --- |
| Drug Availability | Anti-drug policies: Absent Not Effected | Anti-drug policies: Present In Effect |
| Laws | Legalization (medicinal) | Illegal Classification Legal Age allowed for purchase |
| Pricing | Low (more access) | High (price prohibition) |
B-64

## Slide 65
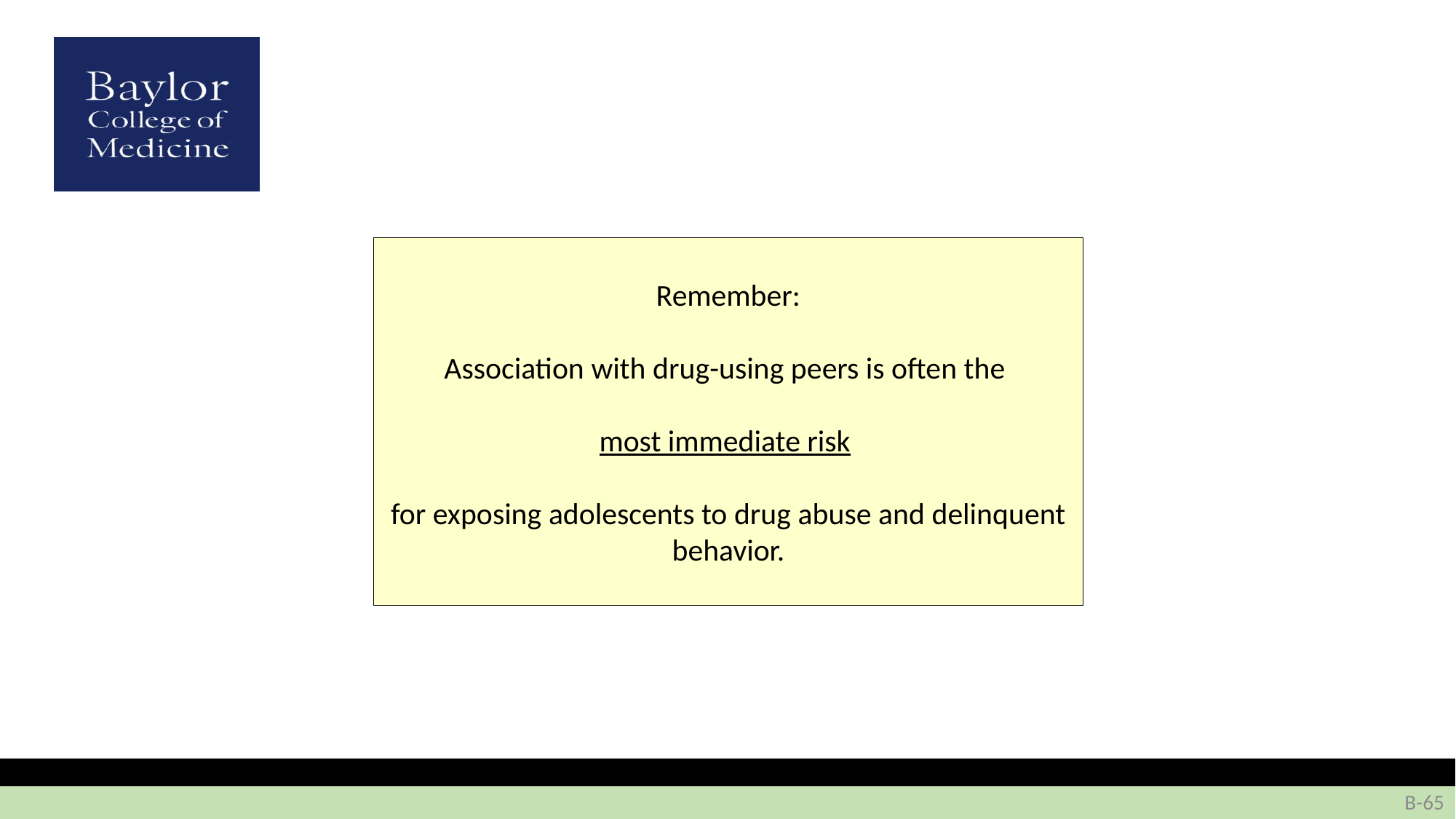

Remember:
Association with drug-using peers is often the
most immediate risk
for exposing adolescents to drug abuse and delinquent behavior.
B-65

## Slide 66
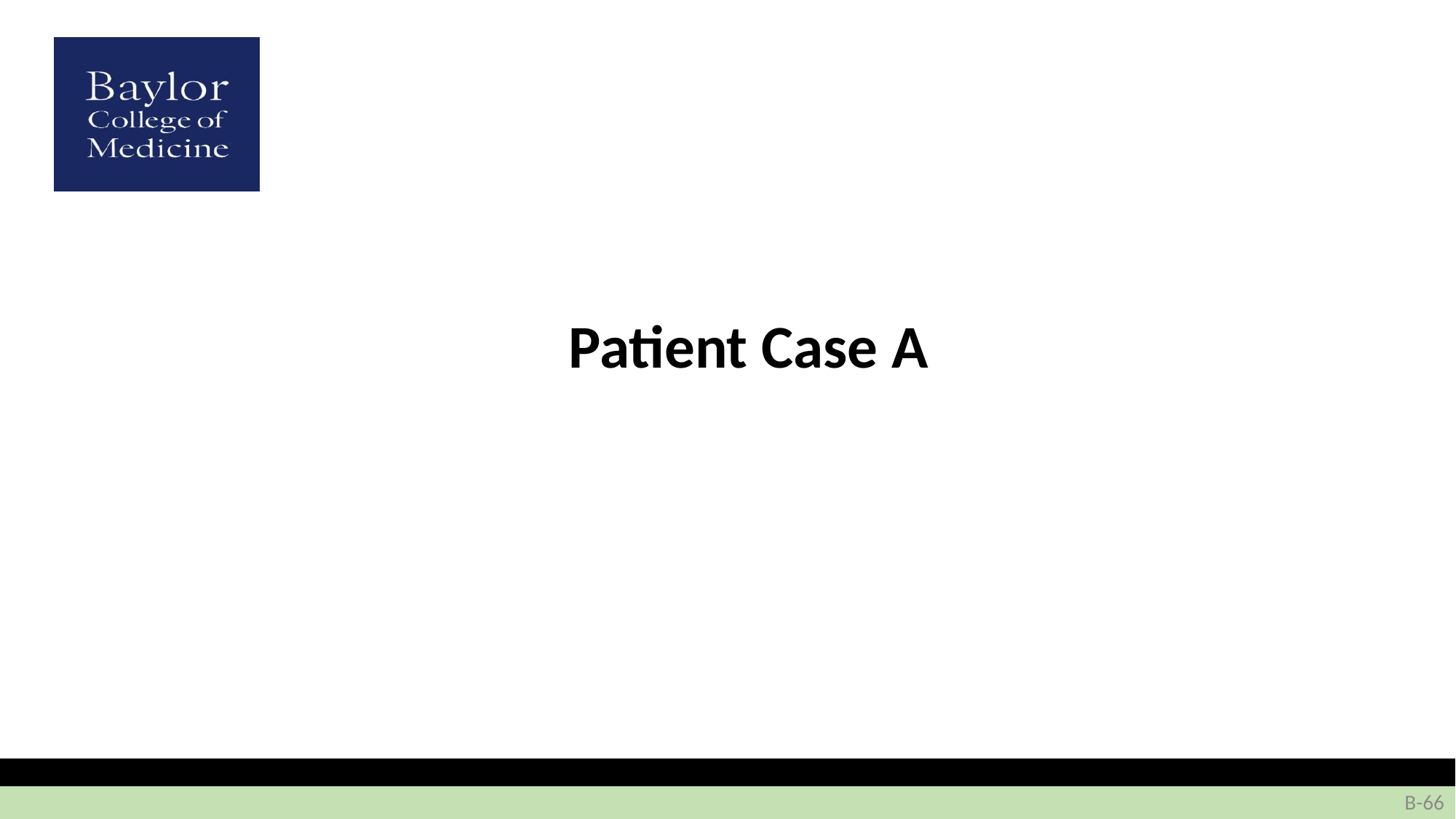

Patient Case A
B-66

## Slide 67
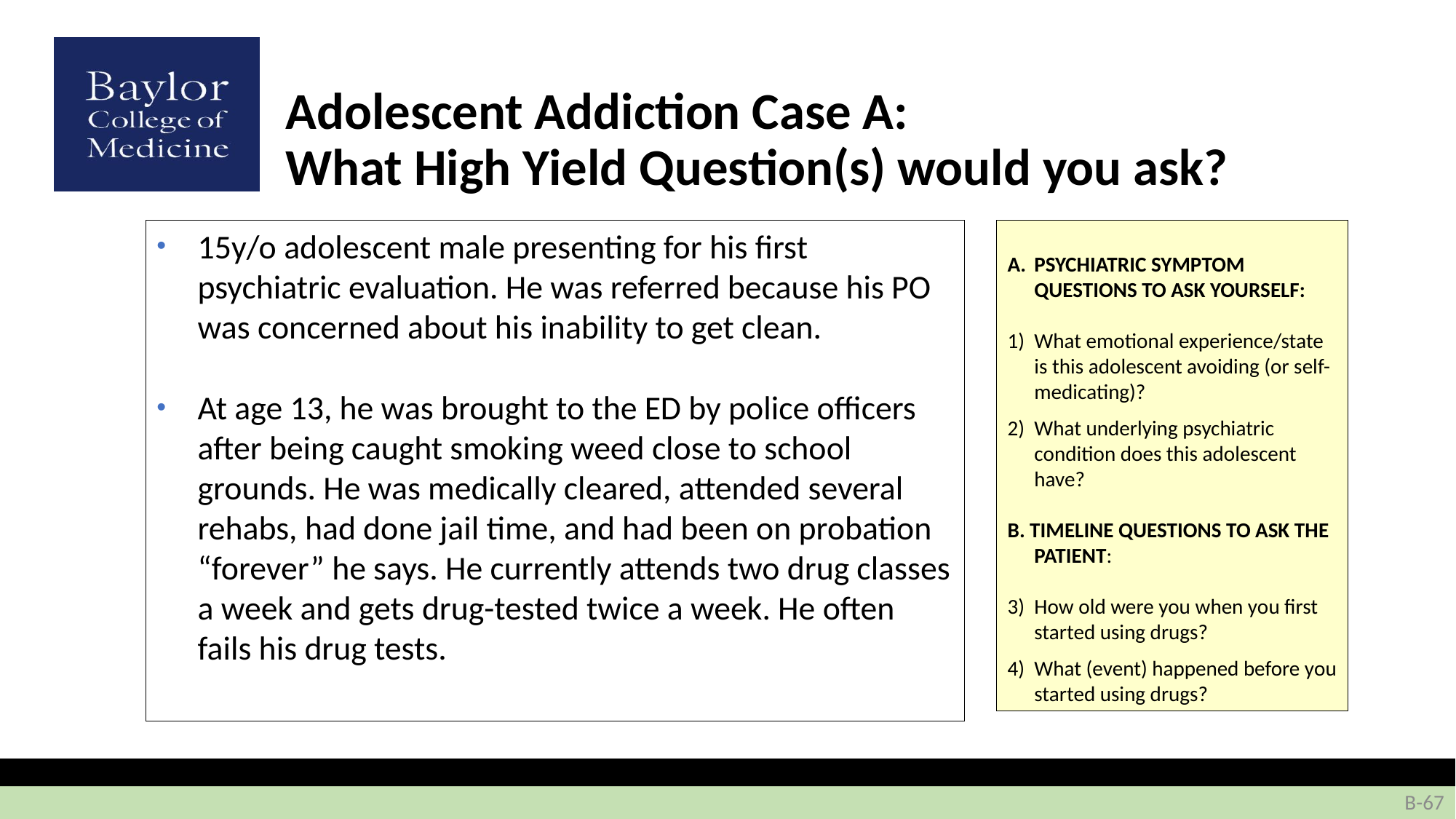

Adolescent Addiction Case A:
What High Yield Question(s) would you ask?
PSYCHIATRIC SYMPTOM QUESTIONS TO ASK YOURSELF:
What emotional experience/state is this adolescent avoiding (or self-medicating)?
What underlying psychiatric condition does this adolescent have?
B. TIMELINE QUESTIONS TO ASK THE PATIENT:
How old were you when you first started using drugs?
What (event) happened before you started using drugs?
15y/o adolescent male presenting for his first psychiatric evaluation. He was referred because his PO was concerned about his inability to get clean.
At age 13, he was brought to the ED by police officers after being caught smoking weed close to school grounds. He was medically cleared, attended several rehabs, had done jail time, and had been on probation “forever” he says. He currently attends two drug classes a week and gets drug-tested twice a week. He often fails his drug tests.
B-67

## Slide 68
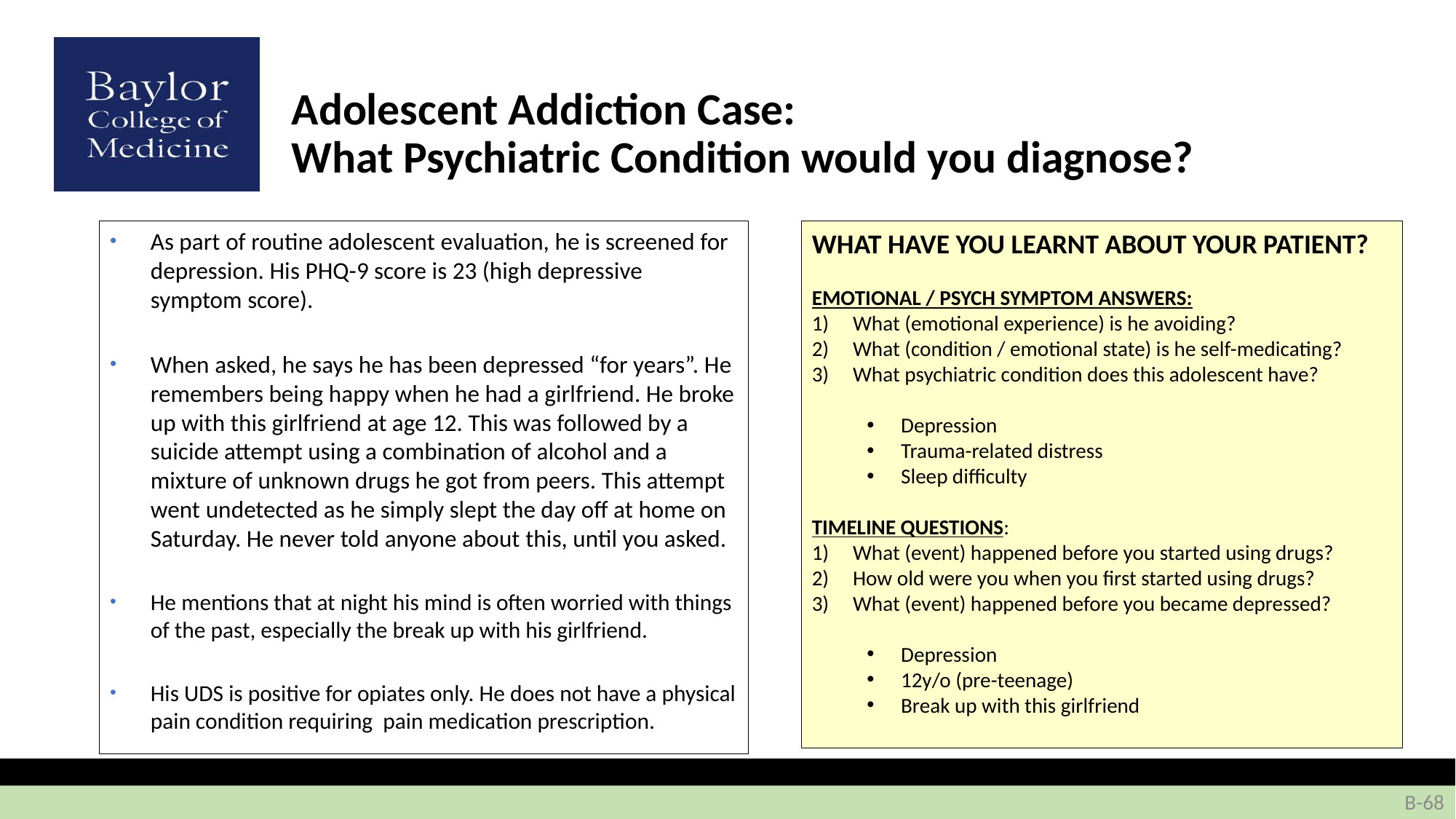

Adolescent Addiction Case:
What Psychiatric Condition would you diagnose?
As part of routine adolescent evaluation, he is screened for depression. His PHQ-9 score is 23 (high depressive symptom score).
When asked, he says he has been depressed “for years”. He remembers being happy when he had a girlfriend. He broke up with this girlfriend at age 12. This was followed by a suicide attempt using a combination of alcohol and a mixture of unknown drugs he got from peers. This attempt went undetected as he simply slept the day off at home on Saturday. He never told anyone about this, until you asked.
He mentions that at night his mind is often worried with things of the past, especially the break up with his girlfriend.
His UDS is positive for opiates only. He does not have a physical pain condition requiring pain medication prescription.
WHAT HAVE YOU LEARNT ABOUT YOUR PATIENT?
EMOTIONAL / PSYCH SYMPTOM ANSWERS:
What (emotional experience) is he avoiding?
What (condition / emotional state) is he self-medicating?
What psychiatric condition does this adolescent have?
Depression
Trauma-related distress
Sleep difficulty
TIMELINE QUESTIONS:
What (event) happened before you started using drugs?
How old were you when you first started using drugs?
What (event) happened before you became depressed?
Depression
12y/o (pre-teenage)
Break up with this girlfriend
ABC
B-68

## Slide 69
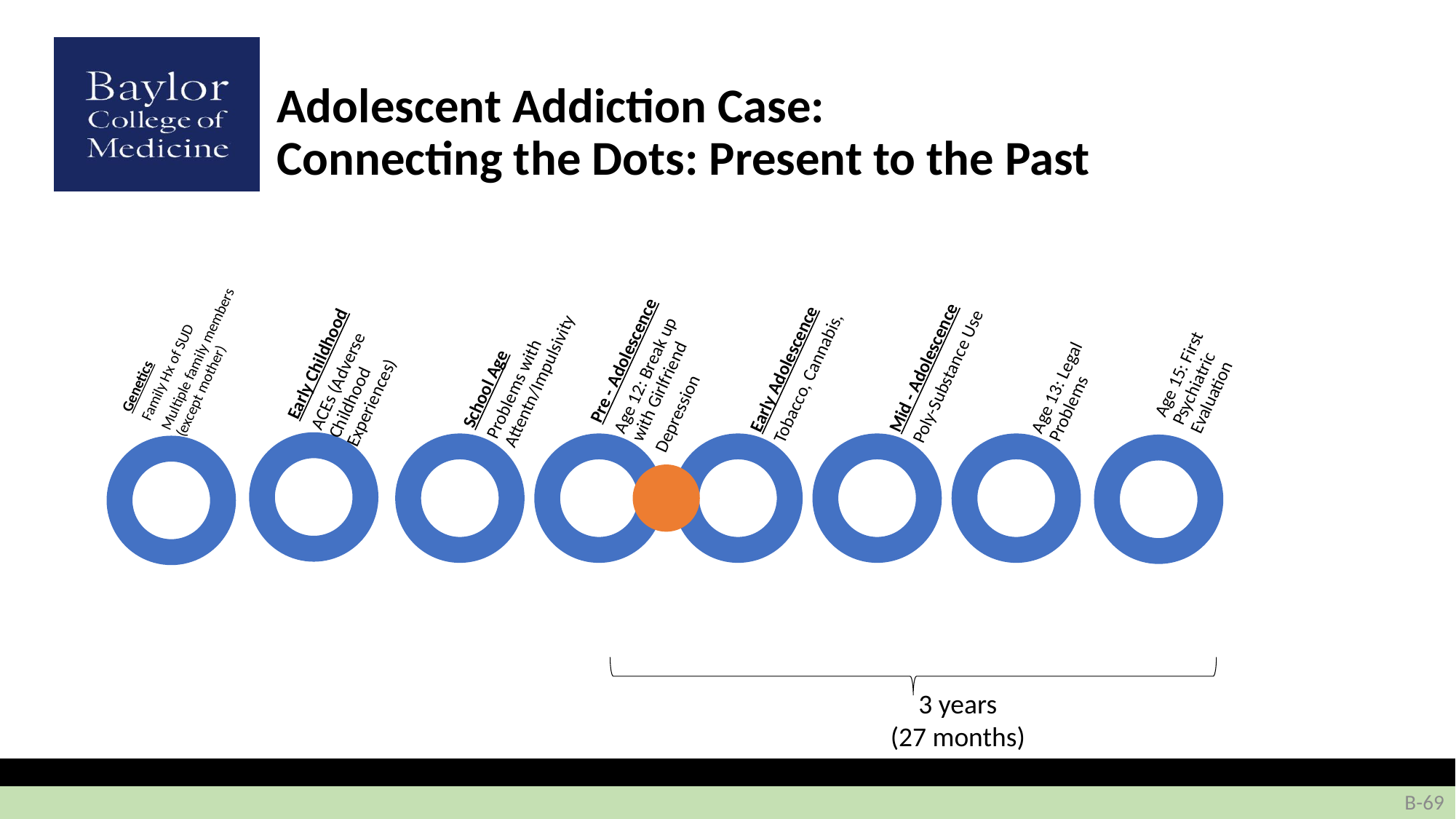

Adolescent Addiction Case:
Connecting the Dots: Present to the Past
3 years
(27 months)
B-69

## Slide 70
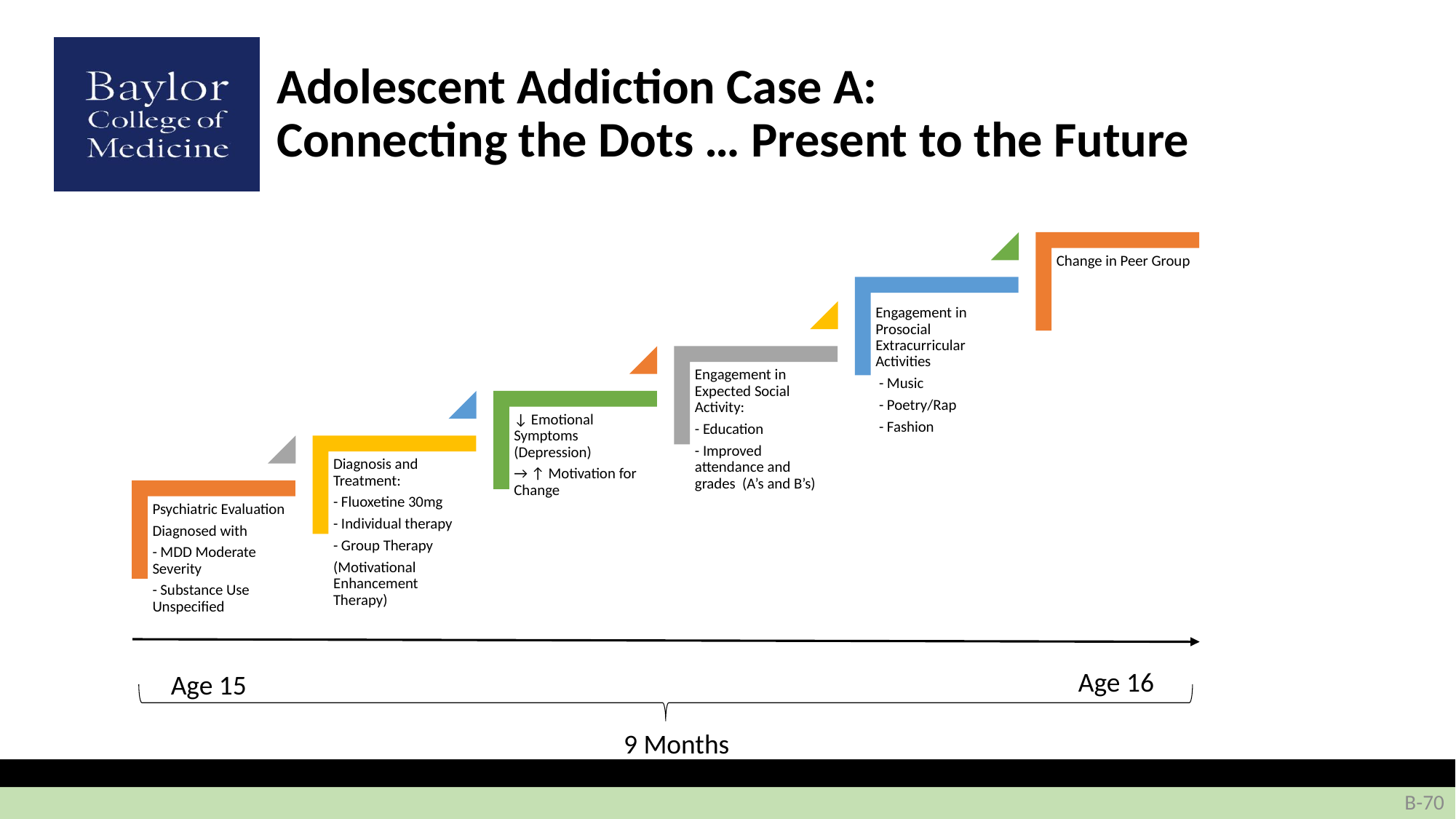

Adolescent Addiction Case A:
Connecting the Dots … Present to the Future
Age 16
Age 15
9 Months
B-70

## Slide 71
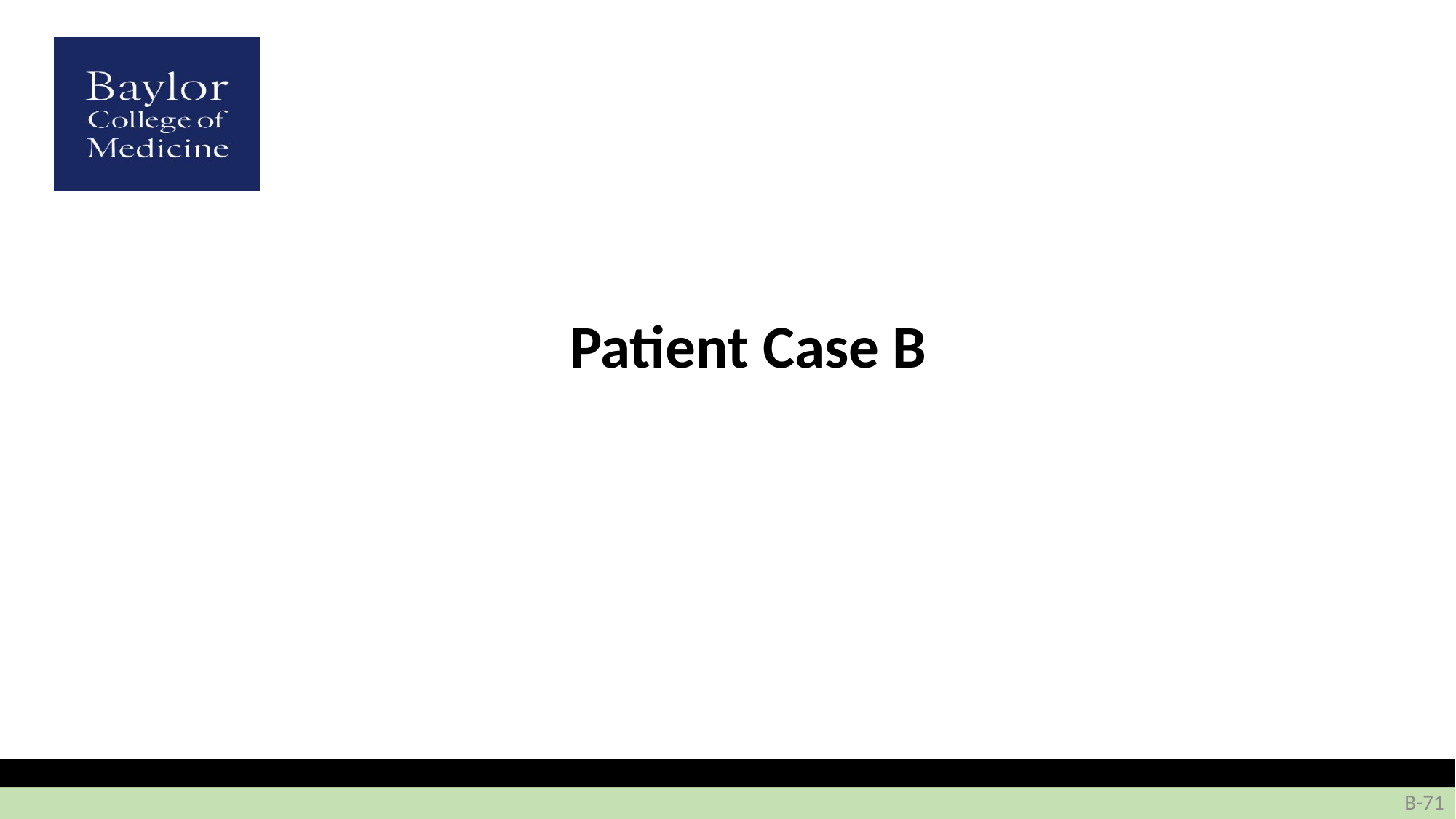

Patient Case B
B-71

## Slide 72
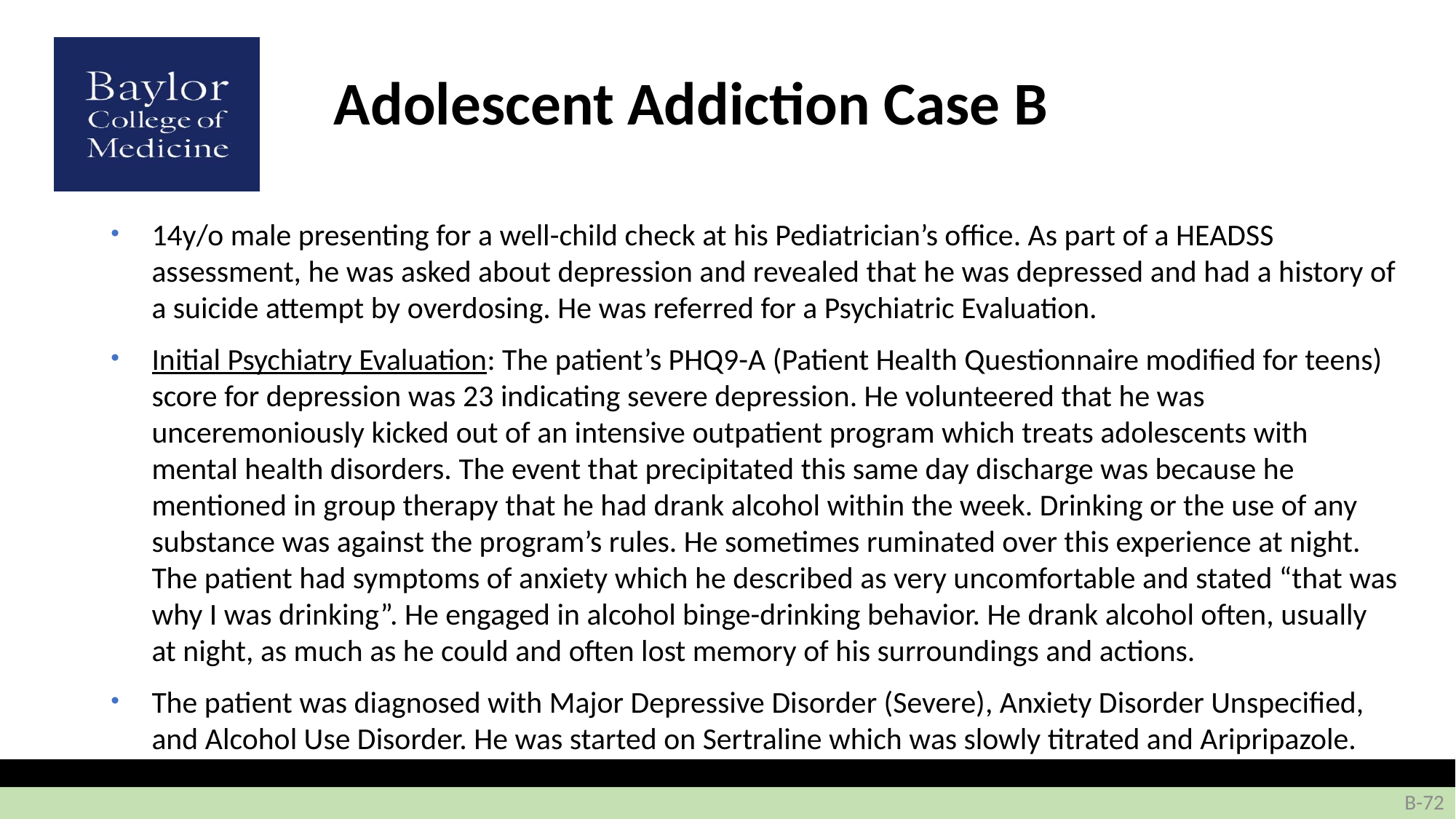

Adolescent Addiction Case B
14y/o male presenting for a well-child check at his Pediatrician’s office. As part of a HEADSS assessment, he was asked about depression and revealed that he was depressed and had a history of a suicide attempt by overdosing. He was referred for a Psychiatric Evaluation.
Initial Psychiatry Evaluation: The patient’s PHQ9-A (Patient Health Questionnaire modified for teens) score for depression was 23 indicating severe depression. He volunteered that he was unceremoniously kicked out of an intensive outpatient program which treats adolescents with mental health disorders. The event that precipitated this same day discharge was because he mentioned in group therapy that he had drank alcohol within the week. Drinking or the use of any substance was against the program’s rules. He sometimes ruminated over this experience at night. The patient had symptoms of anxiety which he described as very uncomfortable and stated “that was why I was drinking”. He engaged in alcohol binge-drinking behavior. He drank alcohol often, usually at night, as much as he could and often lost memory of his surroundings and actions.
The patient was diagnosed with Major Depressive Disorder (Severe), Anxiety Disorder Unspecified, and Alcohol Use Disorder. He was started on Sertraline which was slowly titrated and Aripripazole.
B-72

## Slide 73
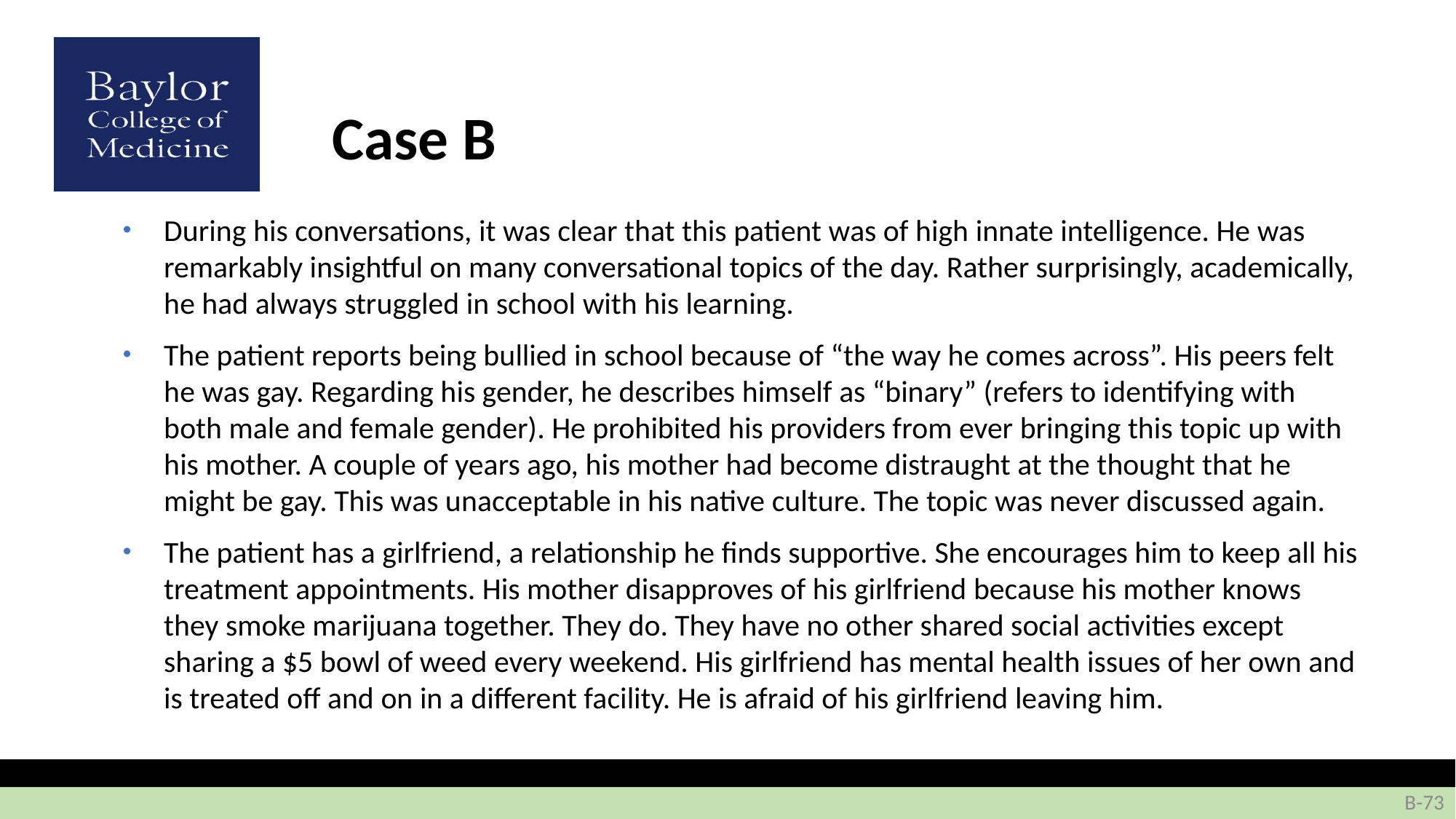

Case B
During his conversations, it was clear that this patient was of high innate intelligence. He was remarkably insightful on many conversational topics of the day. Rather surprisingly, academically, he had always struggled in school with his learning.
The patient reports being bullied in school because of “the way he comes across”. His peers felt he was gay. Regarding his gender, he describes himself as “binary” (refers to identifying with both male and female gender). He prohibited his providers from ever bringing this topic up with his mother. A couple of years ago, his mother had become distraught at the thought that he might be gay. This was unacceptable in his native culture. The topic was never discussed again.
The patient has a girlfriend, a relationship he finds supportive. She encourages him to keep all his treatment appointments. His mother disapproves of his girlfriend because his mother knows they smoke marijuana together. They do. They have no other shared social activities except sharing a $5 bowl of weed every weekend. His girlfriend has mental health issues of her own and is treated off and on in a different facility. He is afraid of his girlfriend leaving him.
B-73

## Slide 74
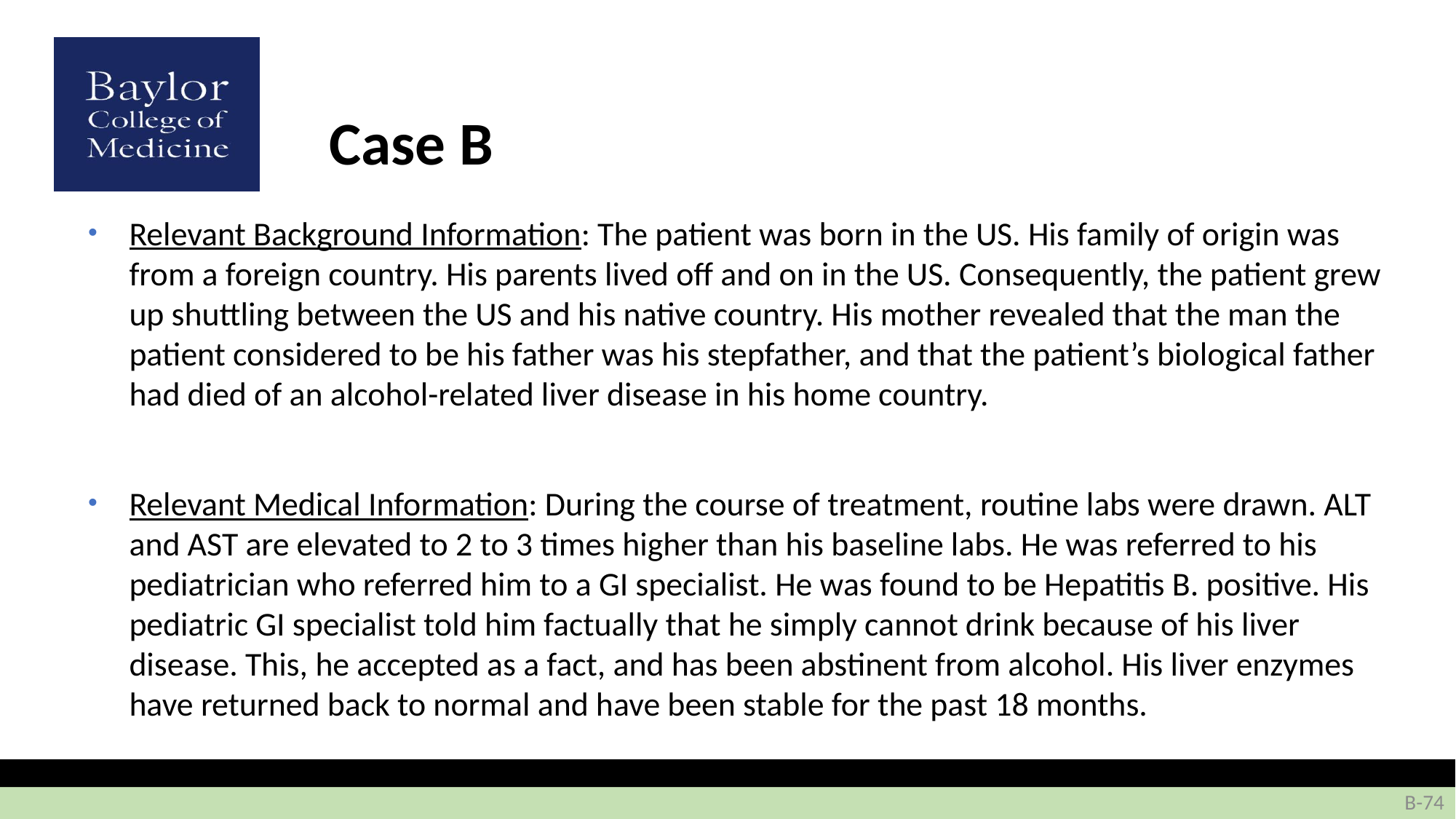

Case B
Relevant Background Information: The patient was born in the US. His family of origin was from a foreign country. His parents lived off and on in the US. Consequently, the patient grew up shuttling between the US and his native country. His mother revealed that the man the patient considered to be his father was his stepfather, and that the patient’s biological father had died of an alcohol-related liver disease in his home country.
Relevant Medical Information: During the course of treatment, routine labs were drawn. ALT and AST are elevated to 2 to 3 times higher than his baseline labs. He was referred to his pediatrician who referred him to a GI specialist. He was found to be Hepatitis B. positive. His pediatric GI specialist told him factually that he simply cannot drink because of his liver disease. This, he accepted as a fact, and has been abstinent from alcohol. His liver enzymes have returned back to normal and have been stable for the past 18 months.
B-74

## Slide 75
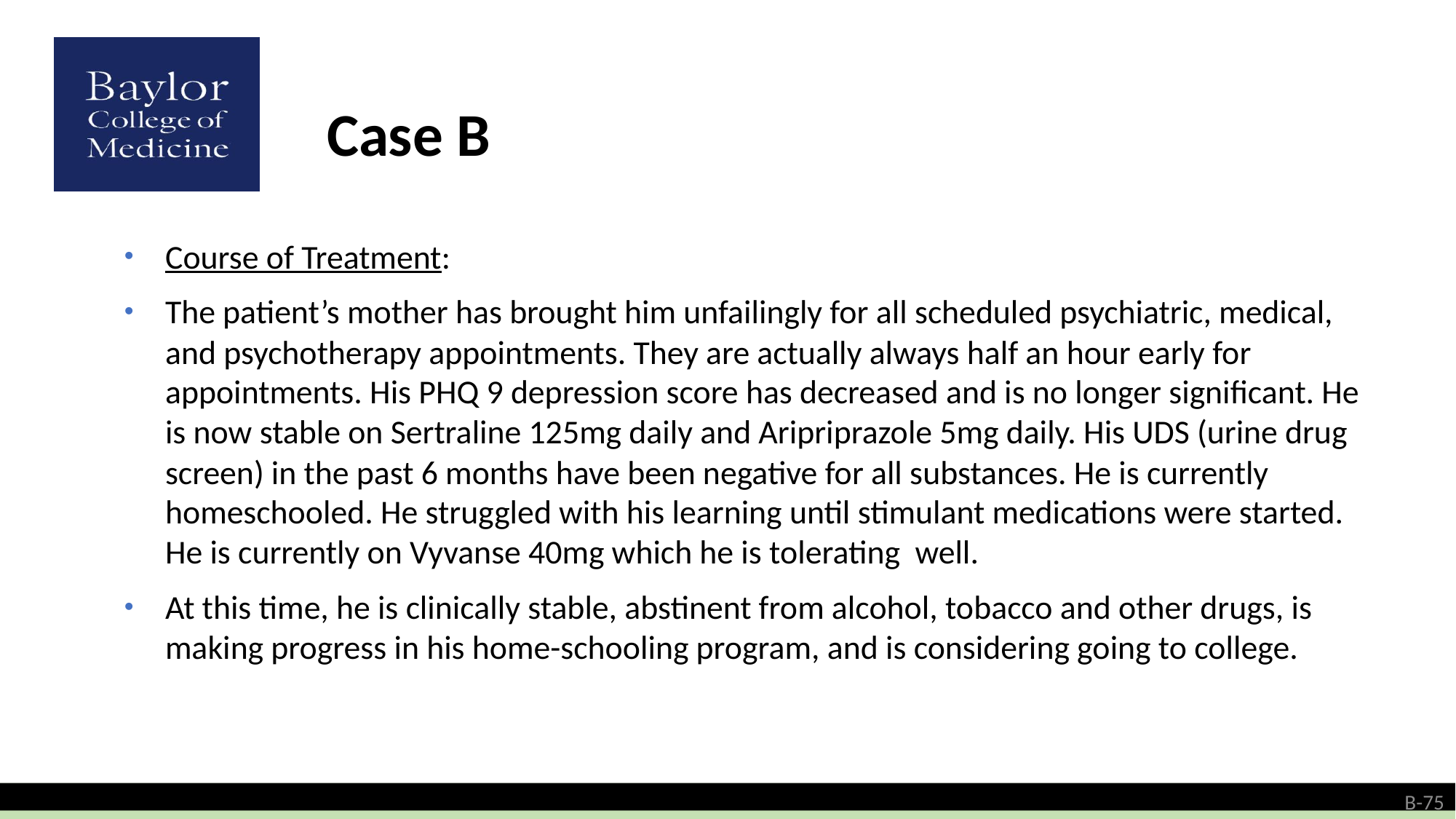

Case B
Course of Treatment:
The patient’s mother has brought him unfailingly for all scheduled psychiatric, medical, and psychotherapy appointments. They are actually always half an hour early for appointments. His PHQ 9 depression score has decreased and is no longer significant. He is now stable on Sertraline 125mg daily and Aripriprazole 5mg daily. His UDS (urine drug screen) in the past 6 months have been negative for all substances. He is currently homeschooled. He struggled with his learning until stimulant medications were started. He is currently on Vyvanse 40mg which he is tolerating well.
At this time, he is clinically stable, abstinent from alcohol, tobacco and other drugs, is making progress in his home-schooling program, and is considering going to college.
B-75

## Slide 76
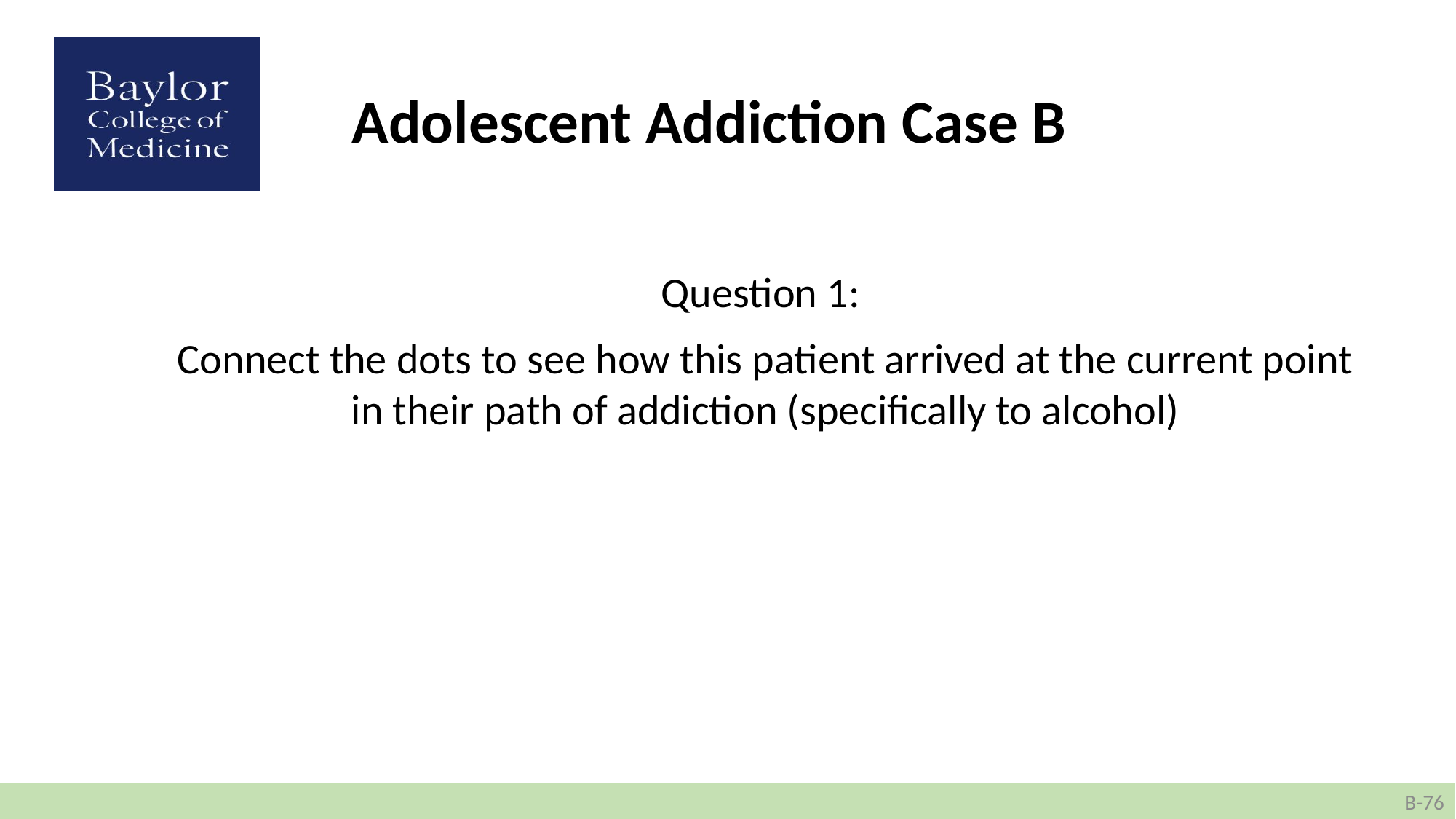

Adolescent Addiction Case B
Question 1:
Connect the dots to see how this patient arrived at the current point in their path of addiction (specifically to alcohol)
B-76

## Slide 77
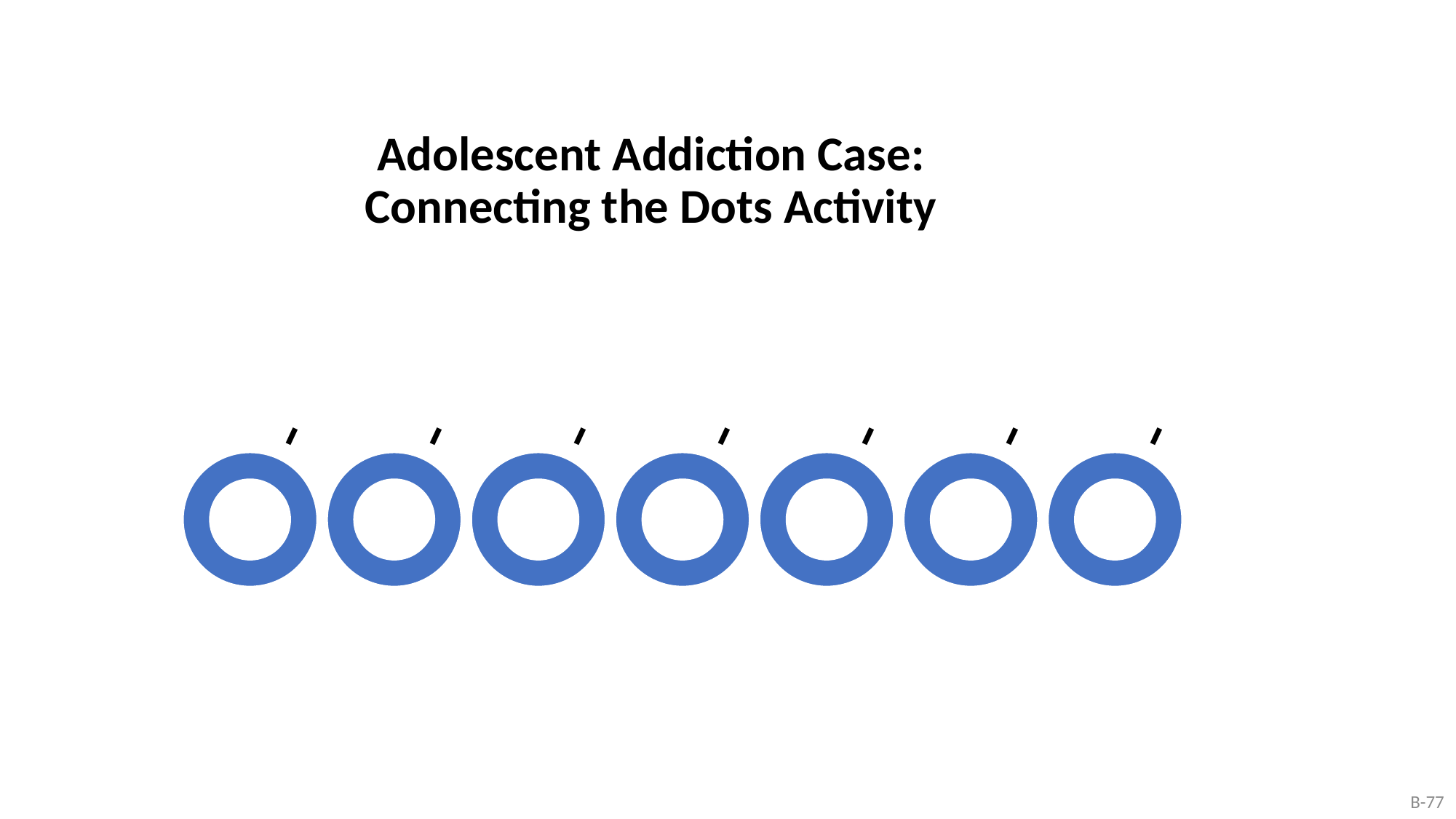

# Adolescent Addiction Case: Connecting the Dots Activity
B-77

## Slide 78
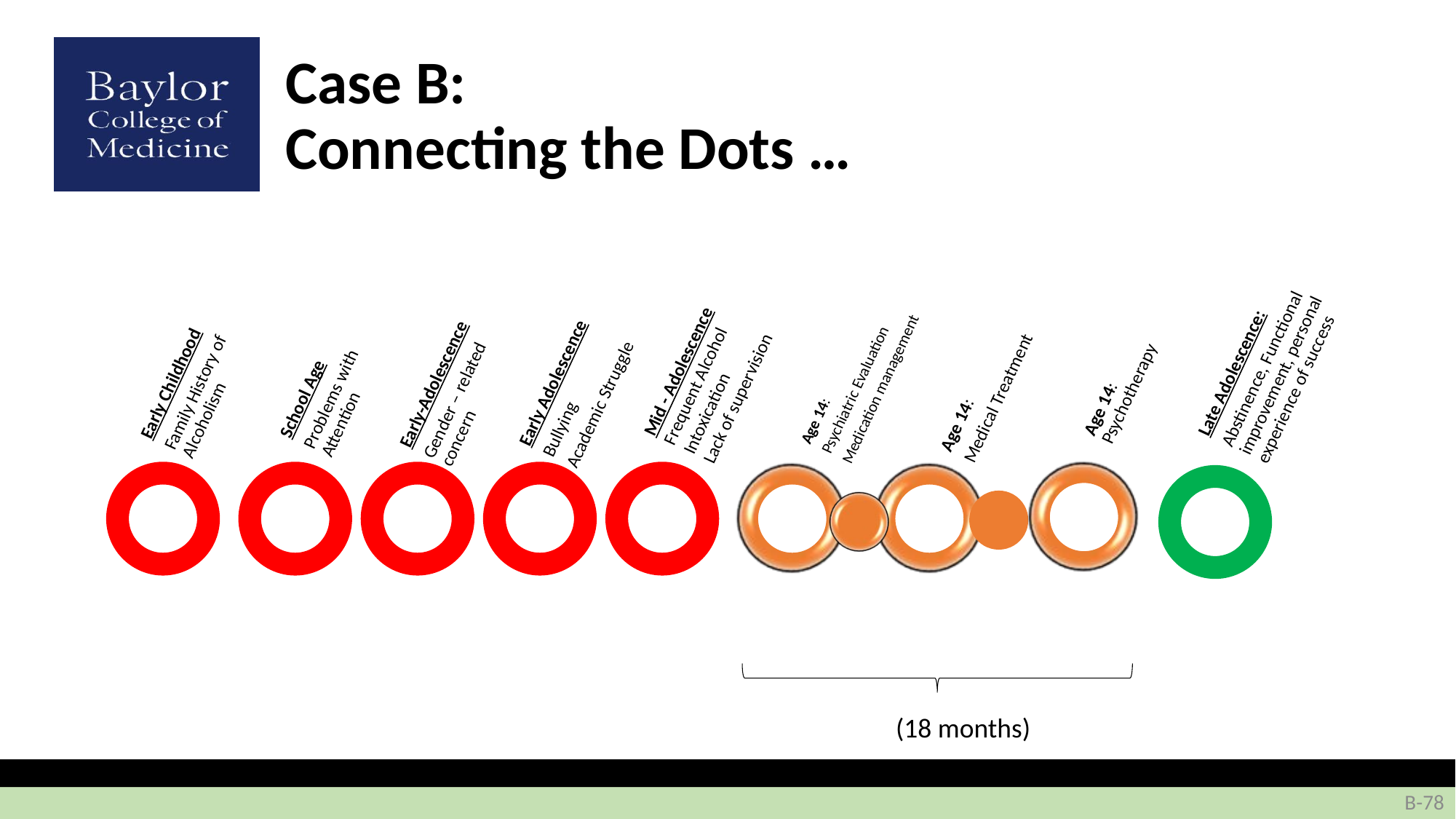

Case B:
Connecting the Dots …
(18 months)
B-78

## Slide 79
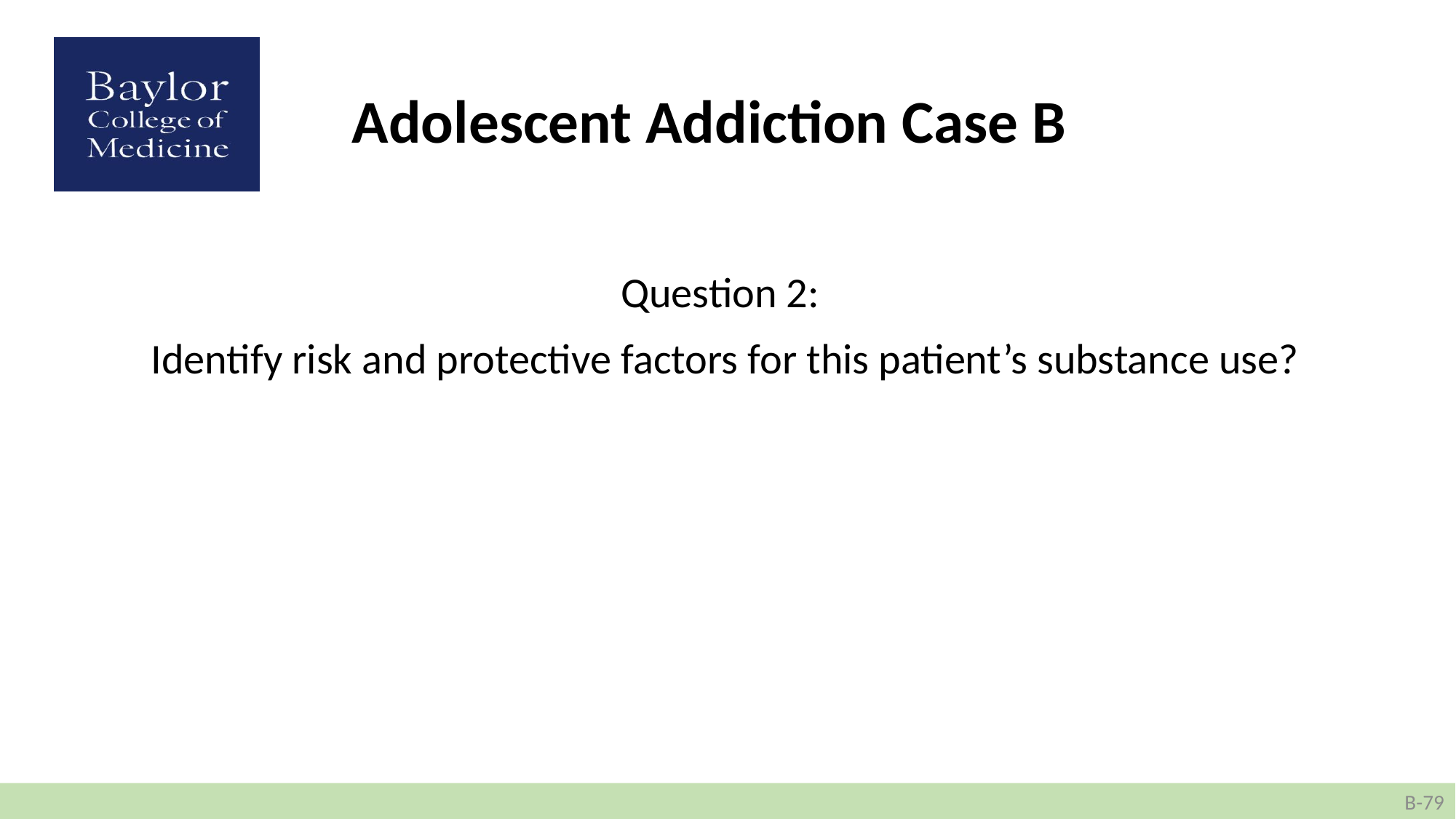

Adolescent Addiction Case B
Question 2:
Identify risk and protective factors for this patient’s substance use?
B-79

## Slide 80
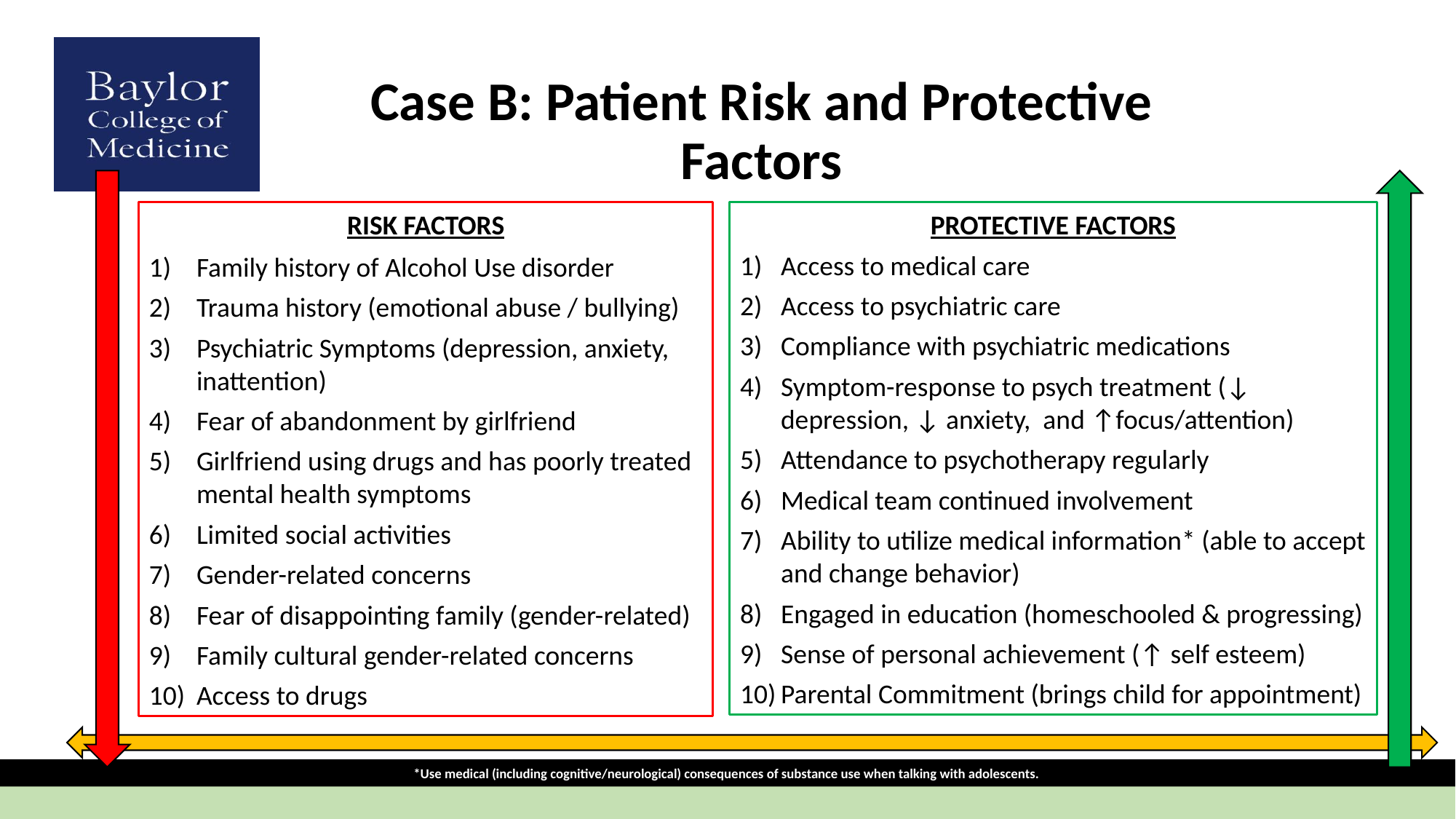

Case B: Patient Risk and Protective Factors
RISK FACTORS
Family history of Alcohol Use disorder
Trauma history (emotional abuse / bullying)
Psychiatric Symptoms (depression, anxiety, inattention)
Fear of abandonment by girlfriend
Girlfriend using drugs and has poorly treated mental health symptoms
Limited social activities
Gender-related concerns
Fear of disappointing family (gender-related)
Family cultural gender-related concerns
Access to drugs
PROTECTIVE FACTORS
Access to medical care
Access to psychiatric care
Compliance with psychiatric medications
Symptom-response to psych treatment (↓ depression, ↓ anxiety, and ↑focus/attention)
Attendance to psychotherapy regularly
Medical team continued involvement
Ability to utilize medical information* (able to accept and change behavior)
Engaged in education (homeschooled & progressing)
Sense of personal achievement (↑ self esteem)
Parental Commitment (brings child for appointment)
*Use medical (including cognitive/neurological) consequences of substance use when talking with adolescents.

## Slide 81
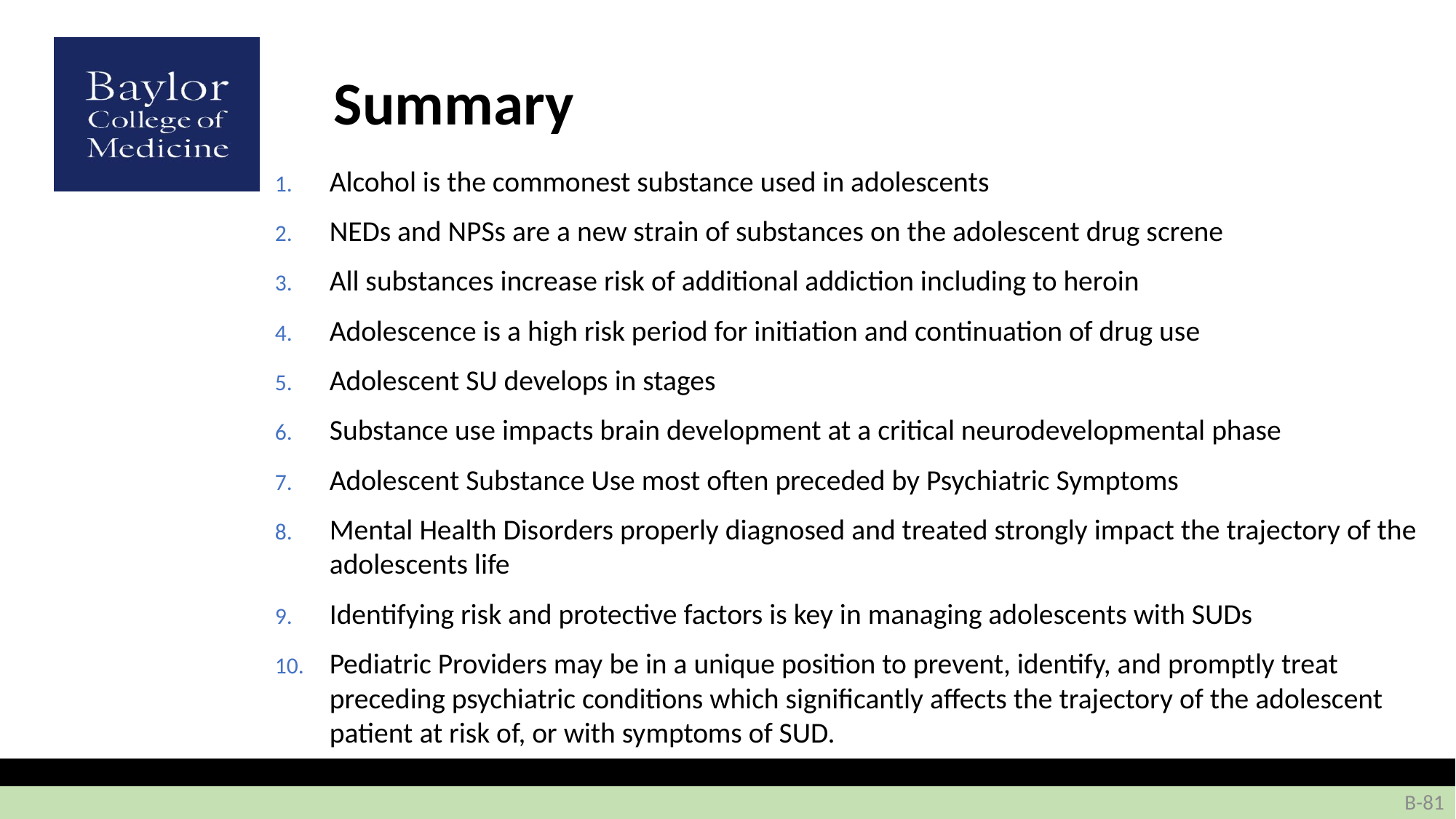

Summary
Alcohol is the commonest substance used in adolescents
NEDs and NPSs are a new strain of substances on the adolescent drug screne
All substances increase risk of additional addiction including to heroin
Adolescence is a high risk period for initiation and continuation of drug use
Adolescent SU develops in stages
Substance use impacts brain development at a critical neurodevelopmental phase
Adolescent Substance Use most often preceded by Psychiatric Symptoms
Mental Health Disorders properly diagnosed and treated strongly impact the trajectory of the adolescents life
Identifying risk and protective factors is key in managing adolescents with SUDs
Pediatric Providers may be in a unique position to prevent, identify, and promptly treat preceding psychiatric conditions which significantly affects the trajectory of the adolescent patient at risk of, or with symptoms of SUD.
B-81

## Slide 82
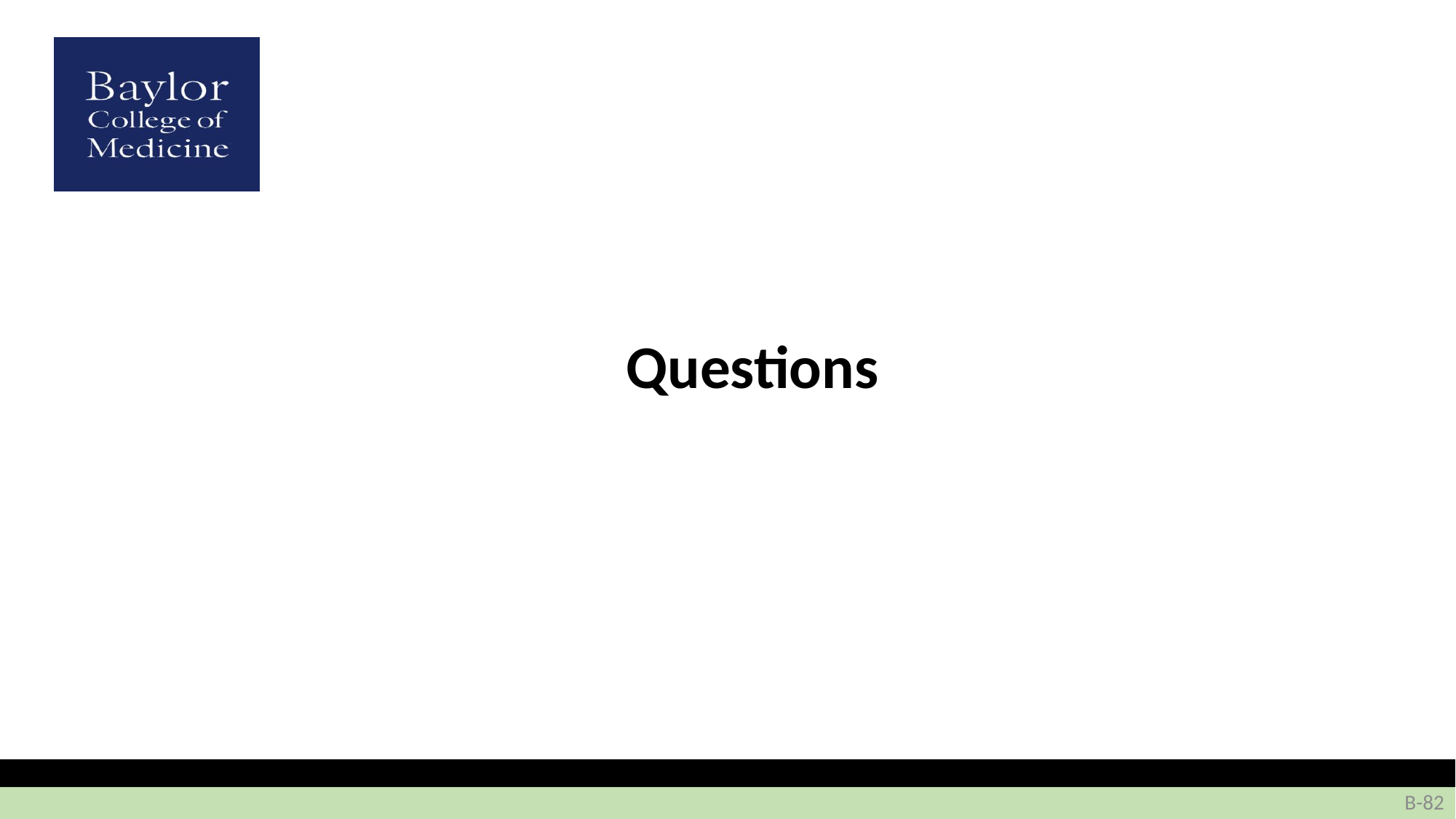

Questions
B-82

## Slide 83
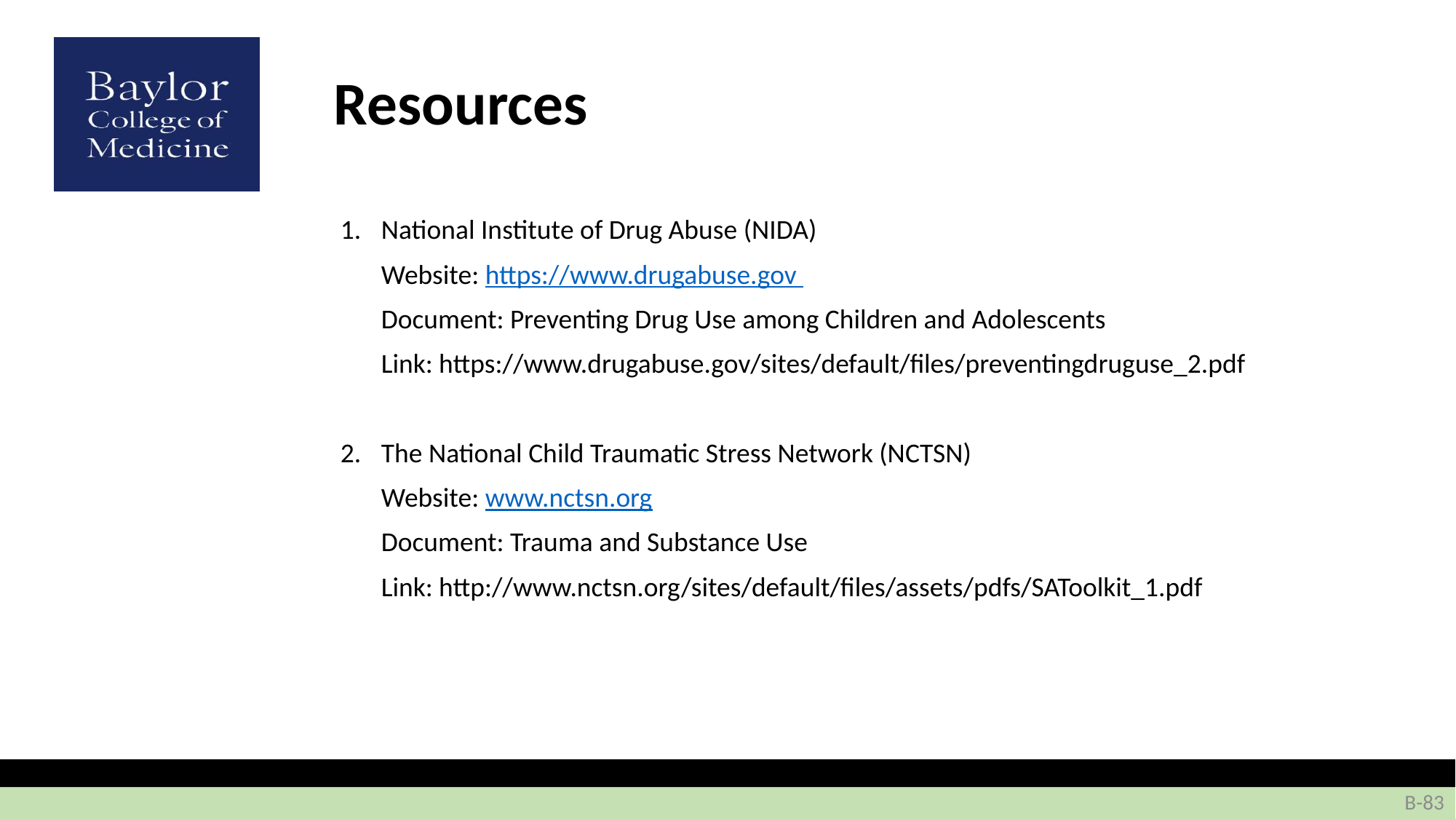

Resources
1. 	National Institute of Drug Abuse (NIDA)
	Website: https://www.drugabuse.gov
	Document: Preventing Drug Use among Children and Adolescents
	Link: https://www.drugabuse.gov/sites/default/files/preventingdruguse_2.pdf
2.	The National Child Traumatic Stress Network (NCTSN)
	Website: www.nctsn.org
	Document: Trauma and Substance Use
	Link: http://www.nctsn.org/sites/default/files/assets/pdfs/SAToolkit_1.pdf
B-83
