# Supplementary material for: Adolescent Addiction Curriculum: Impact on Knowledge Self-Assessment in Pediatric Learners
Source: MedEdPORTAL. 2018 May 7;14:10716. doi: 10.15766/mep_2374-8265.10716 (PMC6342343; doi:10.15766/mep_2374-8265.10716)
Supplement: Supplementary file 1 — A. Addiction Session 1 Lecture Plan.docx B. Addiction Session 1 Instructor Notes.docx C. Addiction Session 1 Slides.pptx D. Addiction Session 1 Self-Assessment.docx E. Addiction Session 2 Lecture Plan.docx F. Addiction Session 2 Instructor Notes.docx G. Addiction Session 2 Slides.pptx H. Addiction Session 2 Self-Assessment.docx I. Addiction Session 2 Worksheets.docx J. Addiction Session 2 Patient Case B.docx K. Addiction Session 3 Lecture Plan.docx L. Addiction Session 3 Instructor Notes.docx M. Addiction Session 3 Slides.pptx N. Addiction Session 3 Self-Assessment.docx [file mep-14-10716-s001.zip › H._Addiction_Session_2_Self-Assessment.docx]

**Adolescent Addiction Session 2 Self-Assessment**

I attended:

1^st^ session ( / / ): Y N

| Title of Lecture: | **Alcohol, New Substances & Physical Harm; Adolescence and Addiction** | | | |
| --- | --- | --- | --- | --- |
| Presenter: |  | | | |
| Date: |  | | | |
| Venue: |  | | | |
| Level of Training: | Resident | Fellow Year 1 | Fellow Year 2 | Other (please describe) |

*For each item, please circle the number that best reflects your assessment of your degree of knowledge*

*(1 reflects limited to no knowledge and 5 reflects being comfortable with your degree of knowledge)*

Part I. Please complete before the session

|  | **General Knowledge (Before Session)** | **Low High** | | | | |
| --- | --- | --- | --- | --- | --- | --- |
|  | I am comfortable with my knowledge of adolescent addiction. | 1 | 2 | 3 | 4 | 5 |
|  | I have a conceptual framework for the disease of addiction. | 1 | 2 | 3 | 4 | 5 |
|  | I can initiate discussions related to addiction with adolescent patients who may be exposed to, or at risk of addiction. | 1 | 2 | 3 | 4 | 5 |
|  | With patients I am treating, I am able to initiate discussions related to medication misuse or abuse. | 1 | 2 | 3 | 4 | 5 |
|  | With patients I am treating, I am able to initiate discussions to highlight emerging addictive behaviors. | 1 | 2 | 3 | 4 | 5 |
|  |  |  |  |  |  |  |

|  | **Session-Specific Knowledge (Before Session)** | **Low High** | | | | |
| --- | --- | --- | --- | --- | --- | --- |
|  | I can describe the basic neurobiology of alcohol addiction. | 1 | 2 | 3 | 4 | 5 |
|  | I am aware of the new and emerging drugs. | 1 | 2 | 3 | 4 | 5 |
|  | I know where substances are rated based on physical harm. | 1 | 2 | 3 | 4 | 5 |
|  | I recognize the link between adolescence and addiction. | 1 | 2 | 3 | 4 | 5 |
|  | I know the comorbid psychiatric conditions in adolescent addiction. | 1 | 2 | 3 | 4 | 5 |
|  | I can identify risk and protective factors for addiction in adolescents. | 1 | 2 | 3 | 4 | 5 |
|  | I appreciate the link between transitions and trauma in the development of adolescent addiction. | 1 | 2 | 3 | 4 | 5 |
|  | I can apply the Socio-Ecologic model to adolescent addiction. | 1 | 2 | 3 | 4 | 5 |
|  | I can connect the dots to better understand the process led to the development of an addictive disorder in the adolescent patient. | 1 | 2 | 3 | 4 | 5 |
|  | I can identify actions taken towards a state of health (mental and physical well-being) in the adolescent with addiction. | 1 | 2 | 3 | 4 | 5 |

Part II. Please complete after the session

|  | **Session-Specific Knowledge (After Session)** | **Low High** | | | | |
| --- | --- | --- | --- | --- | --- | --- |
|  | I can describe the basic neurobiology of alcohol addiction. | 1 | 2 | 3 | 4 | 5 |
|  | I am aware of the new and emerging drugs. | 1 | 2 | 3 | 4 | 5 |
|  | I know where substances are rated based on physical harm. | 1 | 2 | 3 | 4 | 5 |
|  | I recognize the link between adolescence and addiction. | 1 | 2 | 3 | 4 | 5 |
|  | I know the comorbid psychiatric conditions in adolescent addiction. | 1 | 2 | 3 | 4 | 5 |
|  | I can identify risk and protective factors for addiction in adolescents. | 1 | 2 | 3 | 4 | 5 |
|  | I appreciate the link between transitions and trauma in the development of adolescent addiction. | 1 | 2 | 3 | 4 | 5 |
|  | I can apply the Socio-Ecologic model to adolescent addiction. | 1 | 2 | 3 | 4 | 5 |
|  | I can connect the dots to better understand the process led to the development of an addictive disorder in the adolescent patient. | 1 | 2 | 3 | 4 | 5 |
|  | I can identify actions taken towards a state of health (mental and physical well-being) in the adolescent with addiction. | 1 | 2 | 3 | 4 | 5 |

Other Comments: ……………………………………………………………………………………………………………………………………………………………………………………………………………………………………………………………………………………………………………………………………………………………………………………………………………………………………………………………………………………………………………………………………..
