# Supplementary material for: Adolescent Addiction Curriculum: Impact on Knowledge Self-Assessment in Pediatric Learners
Source: MedEdPORTAL. 2018 May 7;14:10716. doi: 10.15766/mep_2374-8265.10716 (PMC6342343; doi:10.15766/mep_2374-8265.10716)
Supplement: Supplementary file 1 — A. Addiction Session 1 Lecture Plan.docx B. Addiction Session 1 Instructor Notes.docx C. Addiction Session 1 Slides.pptx D. Addiction Session 1 Self-Assessment.docx E. Addiction Session 2 Lecture Plan.docx F. Addiction Session 2 Instructor Notes.docx G. Addiction Session 2 Slides.pptx H. Addiction Session 2 Self-Assessment.docx I. Addiction Session 2 Worksheets.docx J. Addiction Session 2 Patient Case B.docx K. Addiction Session 3 Lecture Plan.docx L. Addiction Session 3 Instructor Notes.docx M. Addiction Session 3 Slides.pptx N. Addiction Session 3 Self-Assessment.docx [file mep-14-10716-s001.zip › J._Addiction_Session_2_Patient_Case_B.docx]

**Adolescent Addiction Session 2 Patient Case B**

- 14y/o male presenting for a well-child check at his Pediatrician’s office. As part of a HEADSS assessment, he was asked about depression and revealed that he was depressed and had a history of a suicide attempt by overdosing. He was referred for a Psychiatric Evaluation.
- Initial Psychiatry Evaluation: The patient’s PHQ9-A (Patient Health Questionnaire modified for teens) score for depression was 23 indicating severe depression. He volunteered that he was unceremoniously kicked out of an intensive outpatient program which treats adolescents with mental health disorders. The event that precipitated this same day discharge was because he mentioned in group therapy that he had drank alcohol within the week. Drinking or the use of any substance was against the program’s rules. He sometimes ruminated over this experience at night. The patient had symptoms of anxiety which he described as very uncomfortable and stated, “that was why I was drinking”. He engaged in alcohol binge-drinking behavior. He drank alcohol often, usually at night, as much as he could and often lost memory of his surroundings and actions.
- The patient was diagnosed with Major Depressive Disorder (Severe), Anxiety Disorder Unspecified, and Alcohol Use Disorder. He was started on Sertraline which was slowly titrated and Aripiprazole.
- During his conversations, it was clear that this patient was of high innate intelligence. He was remarkably insightful on many conversational topics of the day. Rather surprisingly, academically, he had always struggled in school with his learning.
- The patient reports being bullied in school because of “the way he comes across”. His peers felt he was gay. Regarding his gender, he describes himself as “binary” (refers to identifying with both male and female gender). He prohibited his providers from ever bringing this topic up with his mother. A couple of years ago, his mother had become distraught at the thought that he might be gay. This was unacceptable in his native culture. The topic was never discussed again.
- The patient has a girlfriend, a relationship he finds supportive. She encourages him to keep all his treatment appointments. His mother disapproves of his girlfriend because his mother knows they smoke marijuana together. They do. They have no other shared social activities except sharing a $5 bowl of weed every weekend. His girlfriend has mental health issues of her own and is treated off and on in a different facility. He is afraid of his girlfriend leaving him.

Relevant Background Information: The patient was born in the US. His family of origin was from a foreign country. His parents lived off and on in the US. Consequently, the patient grew up shuttling between the US and his native country. His mother revealed that the man the patient considered to be his father was his stepfather, and that the patient’s biological father had died of an alcohol-related liver disease in his home country.

- Relevant Medical Information: While treatment, routine labs were done periodically. ALT and AST are elevated to 2 to 3 times higher than his previous levels. He was referred to his pediatrician who referred him to a GI specialist. He was found to be Hepatitis B. positive. His pediatric GI specialist told him factually that he simply cannot drink because of his liver disease. This, he accepted as a fact, and has been abstinent from alcohol. His liver enzymes have returned to normal and have been stable for the past 18 months.
- Course of Treatment:

The patient’s mother has brought him unfailingly for all scheduled psychiatric, medical, and psychotherapy appointments. They are always half an hour early for appointments. His PHQ 9 depression score has decreased and is no longer significant. He is now stable on Sertraline 125mg daily and Aripiprazole 5mg daily. His UDS (urine drug screen) in the past 6 months have been negative for all substances. He is currently homeschooled. He struggled with his learning until stimulant medications were started. He is currently on Vyvanse 40mg which he is tolerating well.

- Currently, he is clinically stable, abstinent from alcohol, tobacco and other drugs, is making progress in his home-schooling program, and is considering going to college.
