# Supplementary material for: Adolescent Addiction Curriculum: Impact on Knowledge Self-Assessment in Pediatric Learners
Source: MedEdPORTAL. 2018 May 7;14:10716. doi: 10.15766/mep_2374-8265.10716 (PMC6342343; doi:10.15766/mep_2374-8265.10716)
Supplement: Supplementary file 1 — A. Addiction Session 1 Lecture Plan.docx B. Addiction Session 1 Instructor Notes.docx C. Addiction Session 1 Slides.pptx D. Addiction Session 1 Self-Assessment.docx E. Addiction Session 2 Lecture Plan.docx F. Addiction Session 2 Instructor Notes.docx G. Addiction Session 2 Slides.pptx H. Addiction Session 2 Self-Assessment.docx I. Addiction Session 2 Worksheets.docx J. Addiction Session 2 Patient Case B.docx K. Addiction Session 3 Lecture Plan.docx L. Addiction Session 3 Instructor Notes.docx M. Addiction Session 3 Slides.pptx N. Addiction Session 3 Self-Assessment.docx [file mep-14-10716-s001.zip › K._Addiction_Session_3_Lecture_Plan.docx]

**Adolescent Addiction Session 3: Lecture Plan**

**Title**: Diagnosis and Treatment of Adolescent Addictive Disorders

**Description**: Adolescent medicine fellows and rotating pediatric residents attended this lecture as part of routine didactic schedule. This lecture will review diagnosis and treatment of addictive disorders.

**Learning Objectives**:

By the end of this seminar, the participant will be able to:

1. Recognize symptoms and signs of addictive disorders using Bio-Psychosocial framework
2. Diagnose Adolescents with Addictive Disorders
3. Identify Appropriate Tests to Screen and Assess Adolescents with Addictive Disorders
4. Apply the Principles of Treatment for Adolescents with Addictive Disorders
5. Know the available Biological and Psychosocial treatments

**Seminar Outline:**

1. Diagnostic Criteria for Addictive Disorder (DSM V)
2. Stages of Adolescent Substance Use
3. Screening
4. History - Taking
5. Examination
6. Ancillary Tests
7. Principles of Treatment
8. Medications
9. Psychosocial Treatments
10. Brief Motivational Interviewing

**Recommended Reading**:

1. NIDA for Teens. <https://teens.drugabuse.gov/>
2. American Academy of Pediatrics, Committee on Substance and Prevention. Policy Statement. Substance Use Screening, Brief Intervention, and Referral to Treatment for Pediatricians. Available at <http://www.accessmhct.com/wp-content/uploads/2017/03/AAP-Policy-Statement-SBIRT.pdf> (Accessed April 6, 2018)
3. American Academy of Pediatrics, Committee on Substance and Prevention. A., Levy, S. J., & Kokotailo, P. K. (2011). Substance use screening, brief intervention, and referral to treatment for pediatricians. *Pediatrics, 128*(5), e1330-1340. doi:10.1542/peds.2011-1754. Available at <http://pediatrics.aappublications.org/content/pediatrics/138/1/e20161211.full.pdf> (Accessed April 6, 2018)
4. American Academy of Pediatrics, Committee on Substance and Prevention. Levy, S., Siqueira, L. M., Ammerman, S. D., Gonzalez, P. K., Ryan, S. A., . . . Smith, V. C. (2014). Testing for drugs of abuse in children and adolescents. *Pediatrics, 133*(6), e1798-1807. doi:10.1542/peds.2014-0865.
5. American Academy of Pediatrics Committee on Substance and Prevention. Policy Statement. Medication-Assisted Treatment of Adolescents With Opioid Use Disorders. Pediatrics. 2016;138(3):e20161893 Available at <http://pediatrics.aappublications.org/content/pediatrics/early/2016/08/18/peds.2016-1893.full.pdf>

(Accessed April 6, 2018)
