# Supplementary material for: Adolescent Addiction Curriculum: Impact on Knowledge Self-Assessment in Pediatric Learners
Source: MedEdPORTAL. 2018 May 7;14:10716. doi: 10.15766/mep_2374-8265.10716 (PMC6342343; doi:10.15766/mep_2374-8265.10716)
Supplement: Supplementary file 1 — A. Addiction Session 1 Lecture Plan.docx B. Addiction Session 1 Instructor Notes.docx C. Addiction Session 1 Slides.pptx D. Addiction Session 1 Self-Assessment.docx E. Addiction Session 2 Lecture Plan.docx F. Addiction Session 2 Instructor Notes.docx G. Addiction Session 2 Slides.pptx H. Addiction Session 2 Self-Assessment.docx I. Addiction Session 2 Worksheets.docx J. Addiction Session 2 Patient Case B.docx K. Addiction Session 3 Lecture Plan.docx L. Addiction Session 3 Instructor Notes.docx M. Addiction Session 3 Slides.pptx N. Addiction Session 3 Self-Assessment.docx [file mep-14-10716-s001.zip › L._Addiction_Session_3_Instructor_Notes.docx]

**Adolescent Addiction Session 3 Instructor Notes**

**Learner Objectives, Activities, Notes to the Educator, and Corresponding PowerPoint Lecture Slides**

Session Title: Diagnosis and Treatment

Learning Goal: To facilitate learners identifying adolescents with addictive disorders, inform on the principles of treatment, and brief interventions available for use in office settings.

|  | **Learner Objective** | **Learner Activity** | **Instructions for Educator** | **PowerPoint Lecture Slides** |
| --- | --- | --- | --- | --- |
|  | Revise diagnostic criteria for addictive disorders | For learners who attended previous sessions, check for recall of diagnostic criteria discussed in Session A. | Review DSM V criteria for SUD | C6 and C7 |
|  | Discuss the clinical assessment of the Adolescent with SUD | From the learners’ experience, ask how learners identified adolescents with addictive disorders. | Discuss assessment along the bio-psycho-social framework  Highlight biological aspects e.g. changes in cognition, memory lapses, uncharacteristic sleep/appetite changes, etc.  Provide useful clinical tips | C8 - C14 |
|  | Identify appropriate screening tests |  | Review validated screening tests  May mention evidence-based screening tests | C15 - C23 |
|  | Determine appropriate drug tests and the circumstances to use them |  | Discuss utility of drug testing  Highlight need to do random drug testing to deter drug use. In principle, this may reduce the presence (or amount) of drugs during a critical neurodevelopmental stage. | C24 - C32 |
|  | Review manifestations of Internet Gaming Disorder (e.g. video game addiction) |  | Highlight that the same fundamental principles of addiction apply (e.g. loss of control, obsessive thinking over, withdrawal symptoms, etc.) | C33 |
|  | BREAK | BREAK | BREAK | BREAK |
|  | Describe components of a comprehensive treatment plan |  | Discuss engagement in other avenues which facilitate closer monitoring of the teen, e.g. school, medical, legal, etc.  Highlight the utility of legal leverage in fostering abstinence (e.g. the teen on probation provides a fresh opportunity to address the core components of their disorder and psychosocial complications, etc.) | C36 |
|  | Discuss patient assessment and clinical recommendations |  | Discuss various levels of care for different levels of severity of the disorder. | C37 |
|  | Determine goals of treatment in the adolescent with substance use disorder |  | Identify ultimate target symptoms is drug-seeking behavior  Highlight goal of total abstinence  Highlight benefits of secondary goal of harm reduction | C38 |
|  | Review the principles of treatment of adolescent addiction |  | Highlight the importance of:   1. Identifying strengths to build upon in the youth. 2. Engaging the youth in prosocial activities 3. Associating with positive peers | C39 |
|  | Revise FDA approved medications for addictive disorders |  | Currently, although there are very few medications approved by the FDA for use in individuals with addictive disorders under the age of 18, a good knowledge base of available medications is helpful. | C40 – C48 |
|  | List some evidence-based psychosocial treatments for adolescents with substance use disorders |  | Highlight the need to have the individual engaged in psychotherapy with a psychotherapist. | C49 – C56 |
|  | BREAK | BREAK | BREAK | BREAK |
|  | Review Brief Interventions |  |  | C50 – C56 |
|  | Describe Brief Motivational Interviewing components |  | Review principles of Motivational Intervention  Provide interviewing tools (OARS) with examples | C58 - 76 |
|  | Explain the importance of prosocial activities in facilitating normal development |  | Highlight the importance of identifying drug-free activities for adolescents to engage in | C77 |
|  | Practice Brief Motivational Interviewing | Think, Pair, Share Activity using MI in 2 scenarios:   1. Personal lifestyle change 2. Adolescent who recently started smoking marijuana 3. Switch interviewer/interviewee roles | Facilitate small group discussion  Have learners share their experience, both as the interviewer and the interviewee | C78 |
|  | Formulate Take Home Points |  | Review summary slide  At the end of this session, it is helpful to emphasize the importance of the clinical assessment of the adolescent. This accomplishes the following: (1) facilitates learner engagement in early identification of addictive disorders, (2) encourages prompt delivery of optimal treatment of any underlying (comorbid) mental health conditions, and (3) focuses learner on goal of decreasing risk of onset and/or progression of existing addictive disorders in their patients. | C79 |
|  | Take Questions |  |  | C80 |
|  | Provide Resources |  |  | C81 |
