# Supplementary material for: Adolescent Addiction Curriculum: Impact on Knowledge Self-Assessment in Pediatric Learners
Source: MedEdPORTAL. 2018 May 7;14:10716. doi: 10.15766/mep_2374-8265.10716 (PMC6342343; doi:10.15766/mep_2374-8265.10716)
Supplement: Supplementary file 1 — A. Addiction Session 1 Lecture Plan.docx B. Addiction Session 1 Instructor Notes.docx C. Addiction Session 1 Slides.pptx D. Addiction Session 1 Self-Assessment.docx E. Addiction Session 2 Lecture Plan.docx F. Addiction Session 2 Instructor Notes.docx G. Addiction Session 2 Slides.pptx H. Addiction Session 2 Self-Assessment.docx I. Addiction Session 2 Worksheets.docx J. Addiction Session 2 Patient Case B.docx K. Addiction Session 3 Lecture Plan.docx L. Addiction Session 3 Instructor Notes.docx M. Addiction Session 3 Slides.pptx N. Addiction Session 3 Self-Assessment.docx [file mep-14-10716-s001.zip › M._Addiction_Session_3_Slides.pptx]

## Slide 1
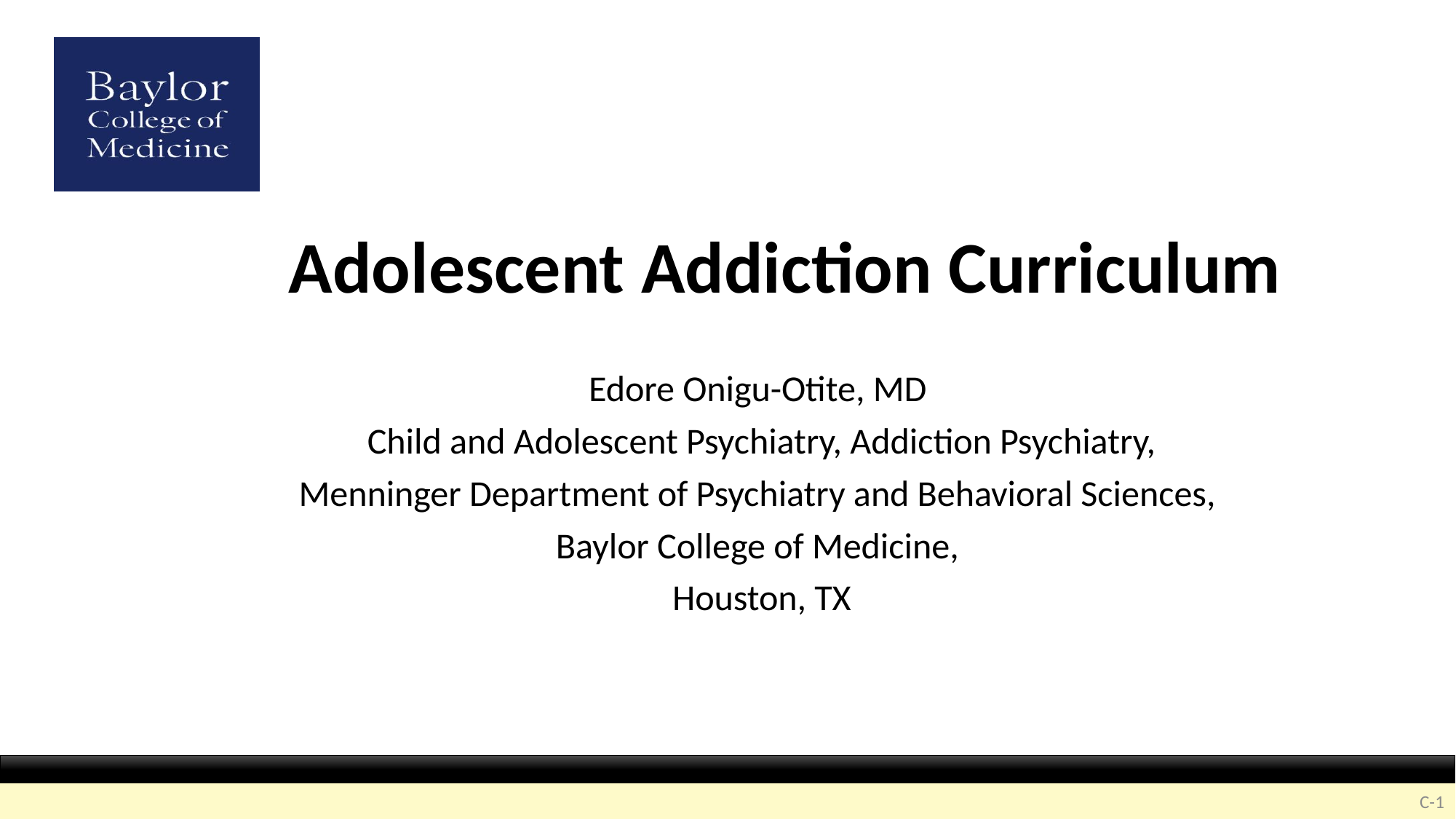

Adolescent Addiction Curriculum
Edore Onigu-Otite, MD
Child and Adolescent Psychiatry, Addiction Psychiatry,
Menninger Department of Psychiatry and Behavioral Sciences,
Baylor College of Medicine,
Houston, TX
C-1

## Slide 2
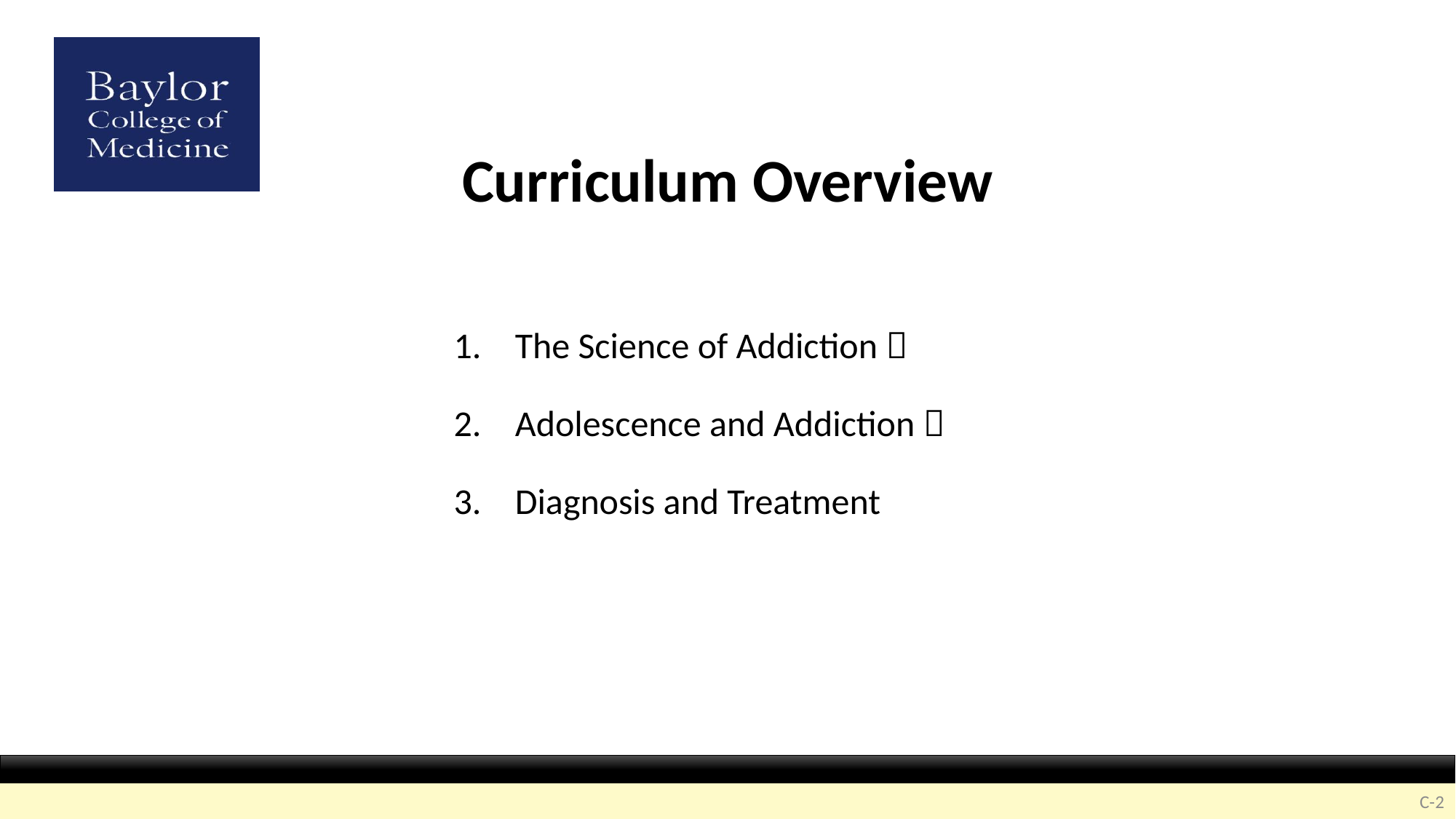

Curriculum Overview
The Science of Addiction 
Adolescence and Addiction 
Diagnosis and Treatment
C-2

## Slide 3
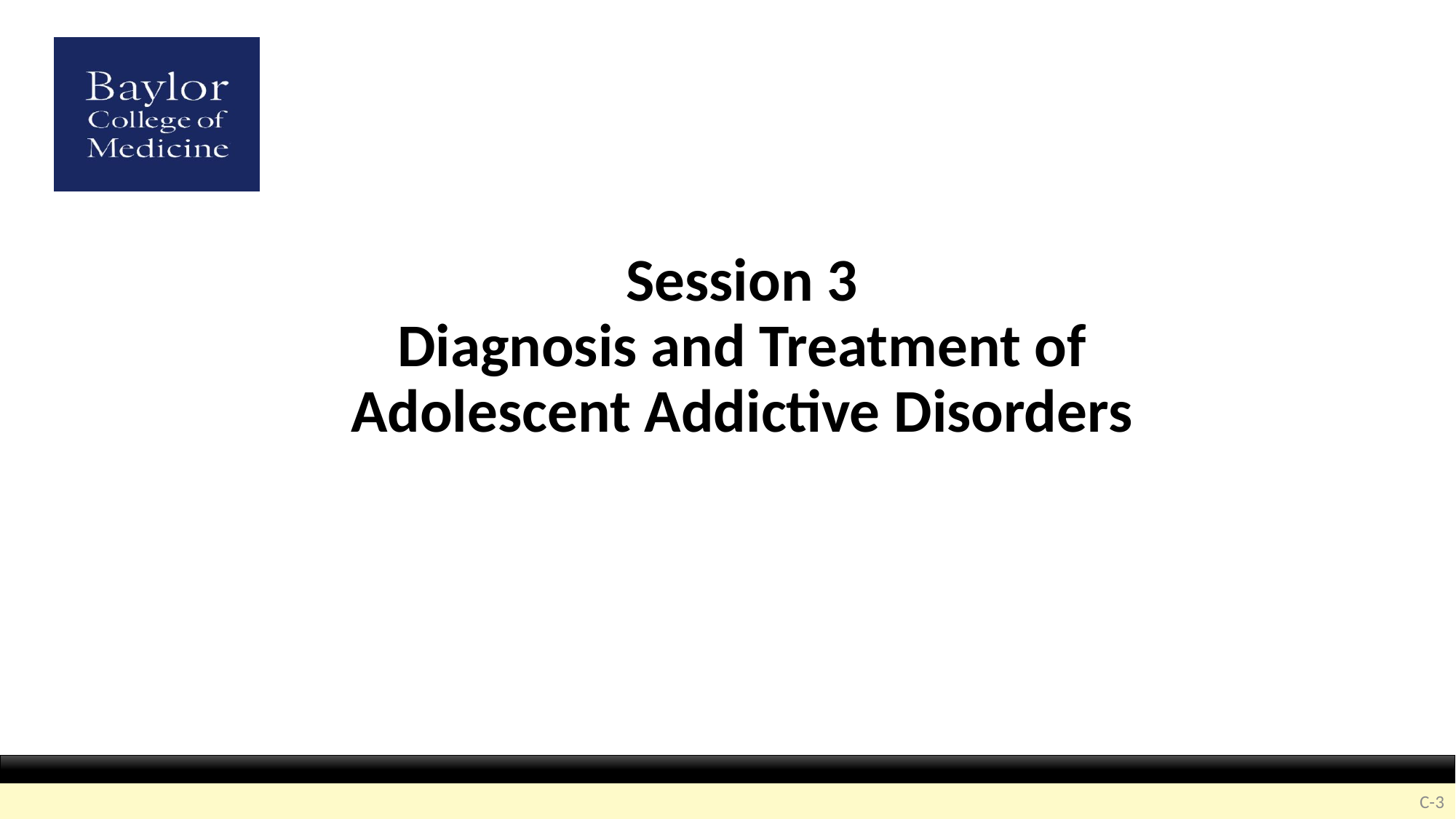

Session 3
Diagnosis and Treatment of Adolescent Addictive Disorders
C-3

## Slide 4
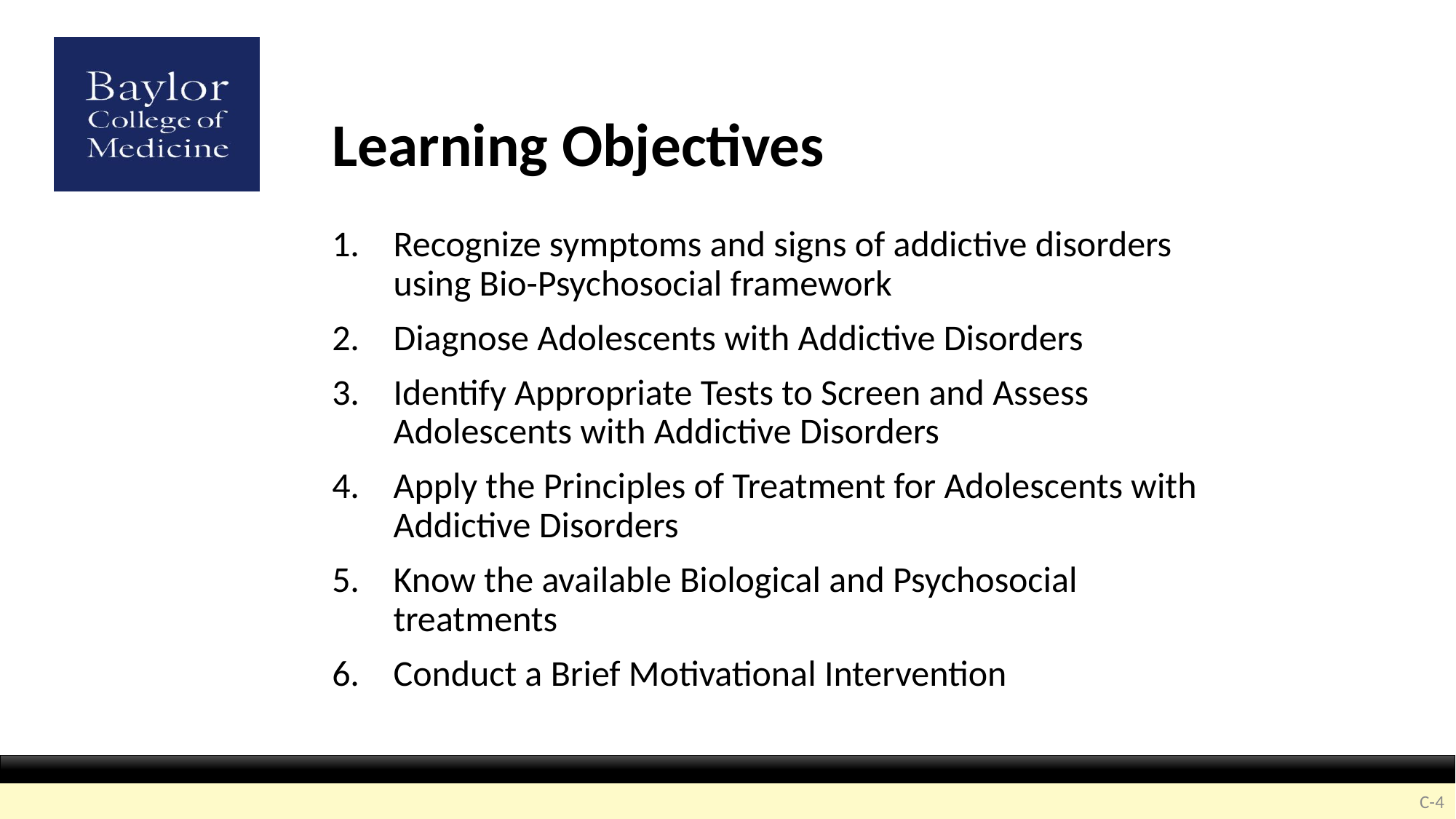

Learning Objectives
Recognize symptoms and signs of addictive disorders using Bio-Psychosocial framework
Diagnose Adolescents with Addictive Disorders
Identify Appropriate Tests to Screen and Assess Adolescents with Addictive Disorders
Apply the Principles of Treatment for Adolescents with Addictive Disorders
Know the available Biological and Psychosocial treatments
Conduct a Brief Motivational Intervention
C-4

## Slide 5
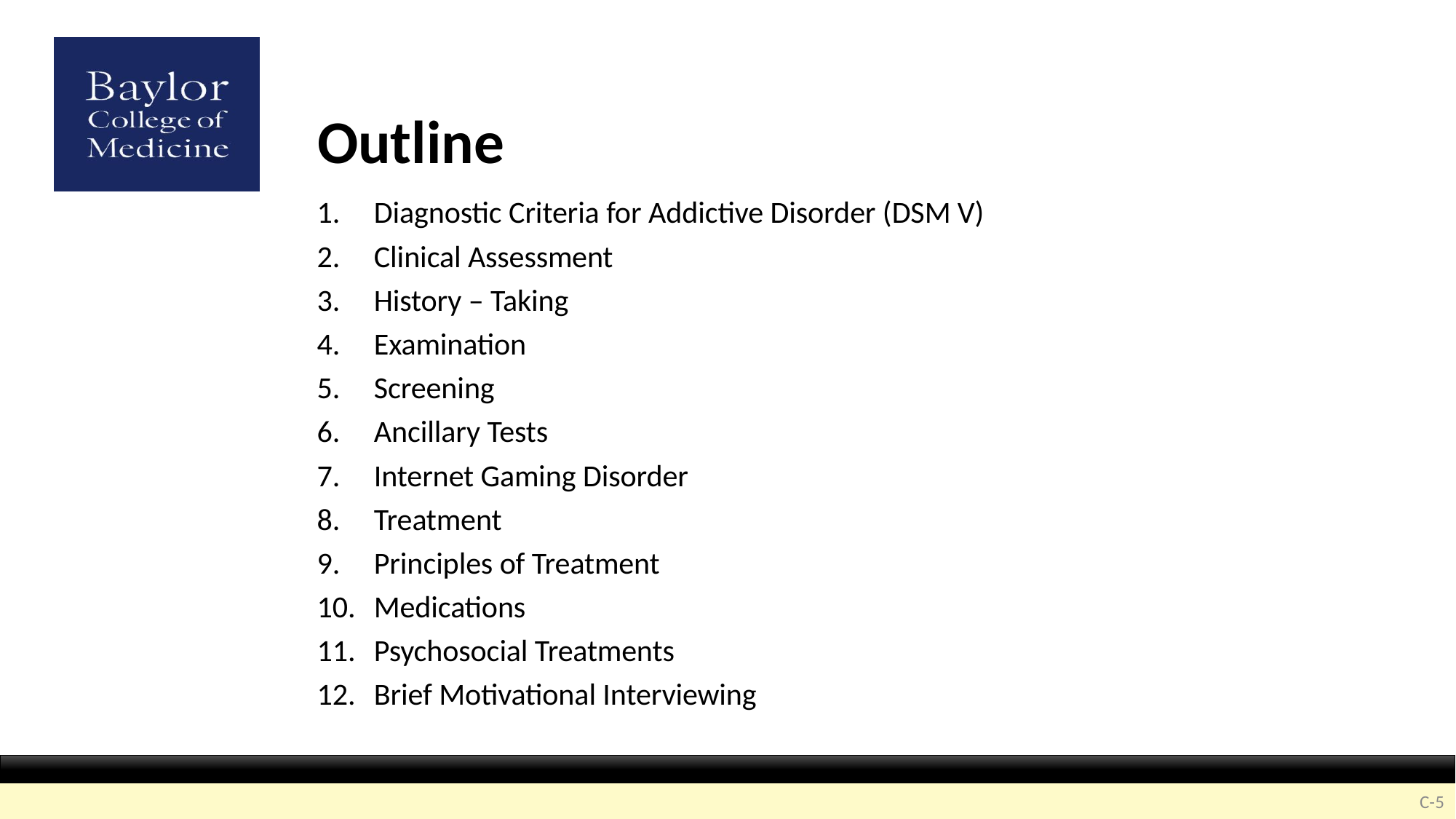

Outline
Diagnostic Criteria for Addictive Disorder (DSM V)
Clinical Assessment
History – Taking
Examination
Screening
Ancillary Tests
Internet Gaming Disorder
Treatment
Principles of Treatment
Medications
Psychosocial Treatments
Brief Motivational Interviewing
C-5

## Slide 6
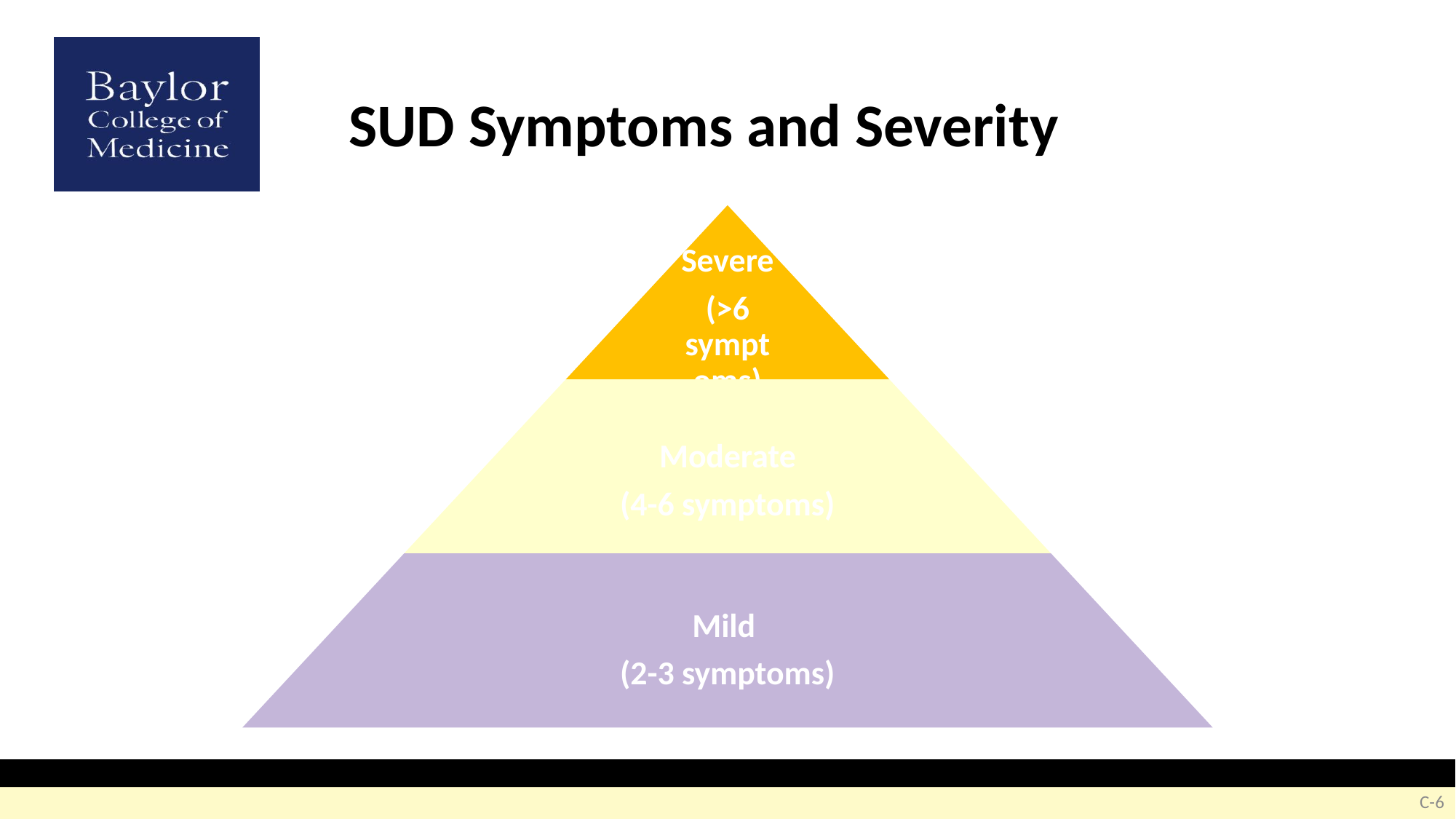

SUD Symptoms and Severity
C-6

## Slide 7
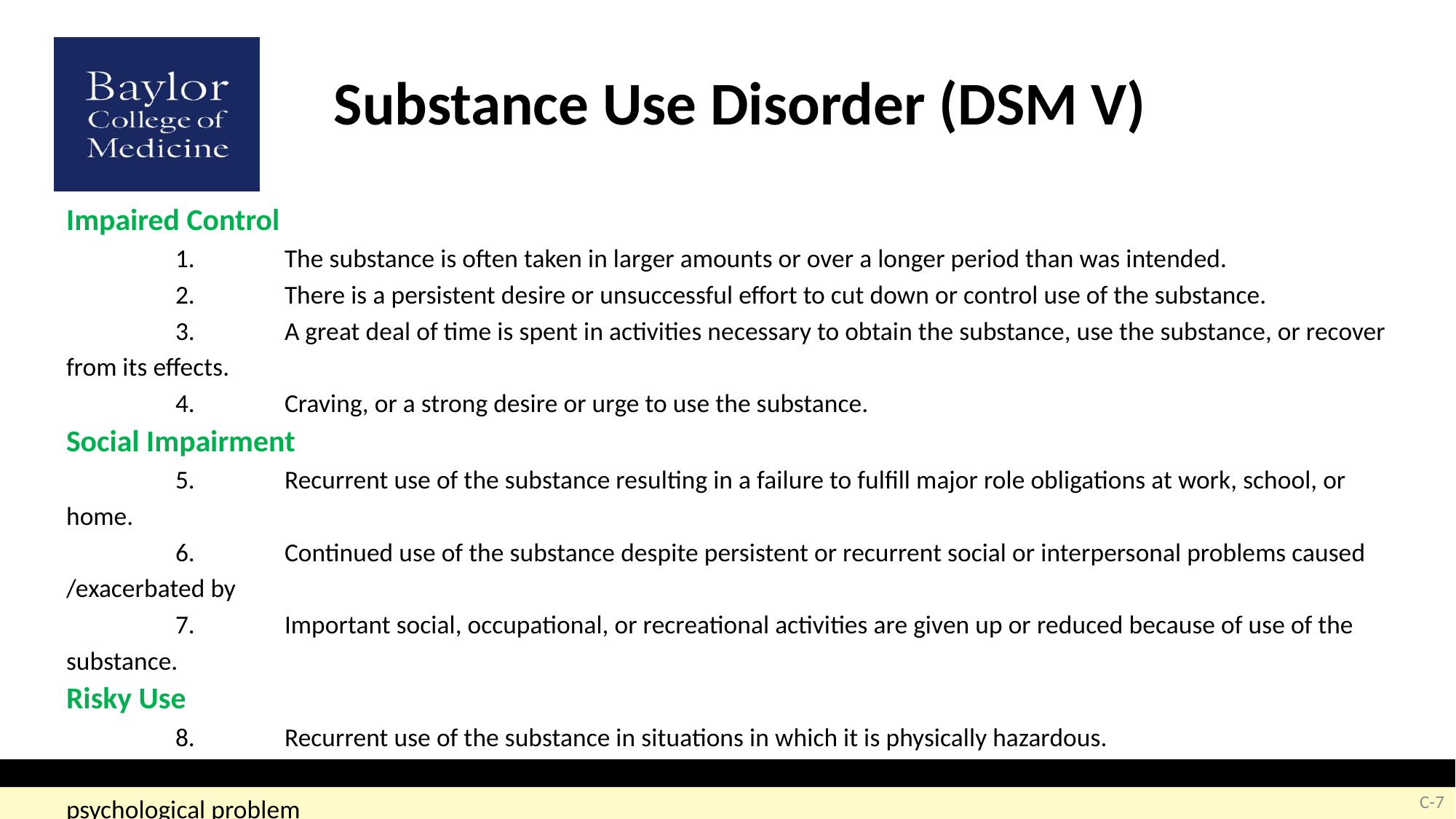

Substance Use Disorder (DSM V)
Impaired Control
	1.	The substance is often taken in larger amounts or over a longer period than was intended.
	2.	There is a persistent desire or unsuccessful effort to cut down or control use of the substance.
	3.	A great deal of time is spent in activities necessary to obtain the substance, use the substance, or recover from its effects.
	4.	Craving, or a strong desire or urge to use the substance.
Social Impairment
	5.	Recurrent use of the substance resulting in a failure to fulfill major role obligations at work, school, or home.
	6.	Continued use of the substance despite persistent or recurrent social or interpersonal problems caused /exacerbated by
	7.	Important social, occupational, or recreational activities are given up or reduced because of use of the substance.
Risky Use
	8.	Recurrent use of the substance in situations in which it is physically hazardous.
	9.	Use of the substance is continued despite knowledge of having a persistent or recurrent physical or psychological problem
Pharmacological Criteria
	10.	Tolerance
	11.	Withdrawal
C-7

## Slide 8
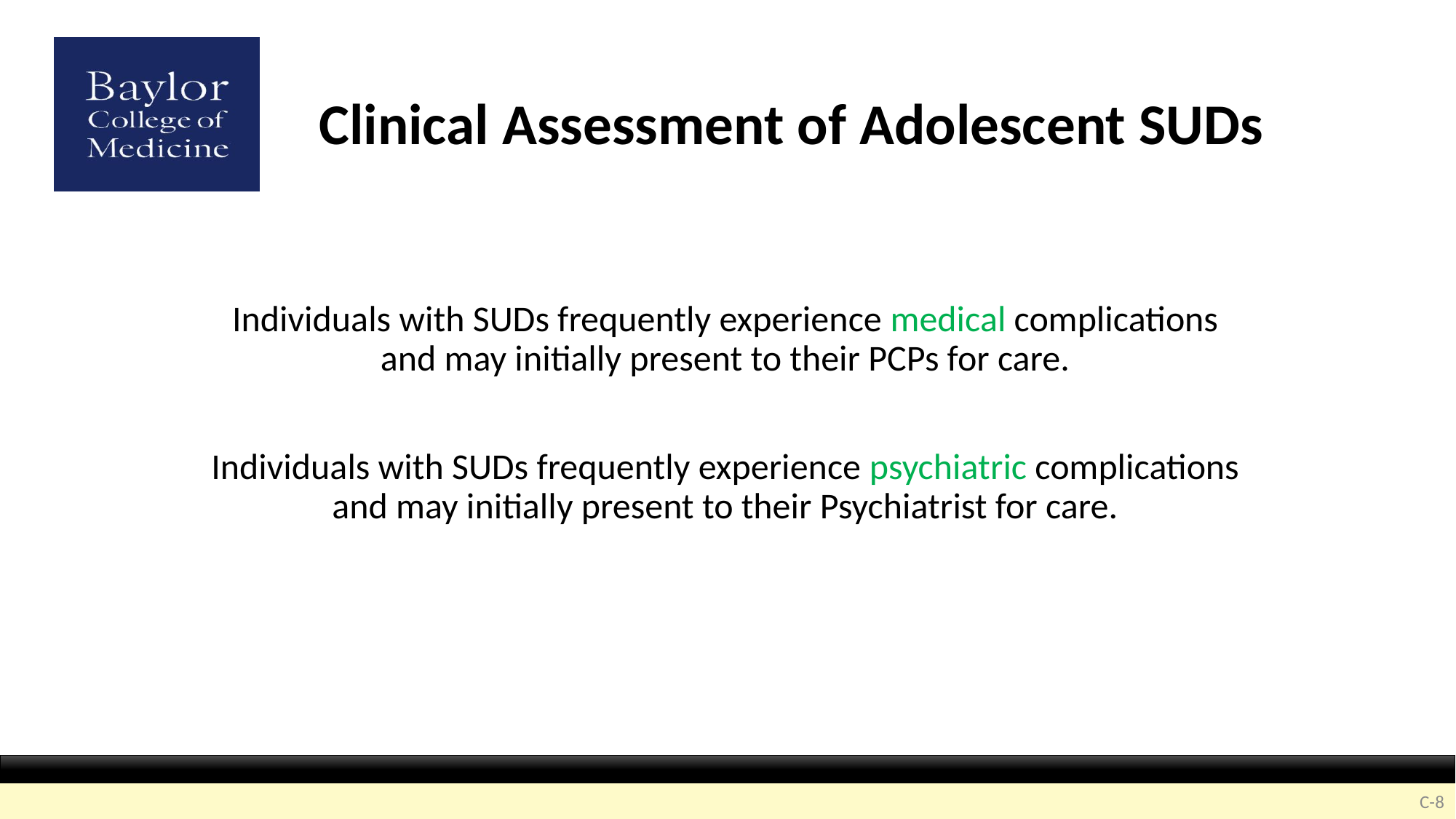

Clinical Assessment of Adolescent SUDs
Individuals with SUDs frequently experience medical complications and may initially present to their PCPs for care.
Individuals with SUDs frequently experience psychiatric complications and may initially present to their Psychiatrist for care.
C-8

## Slide 9
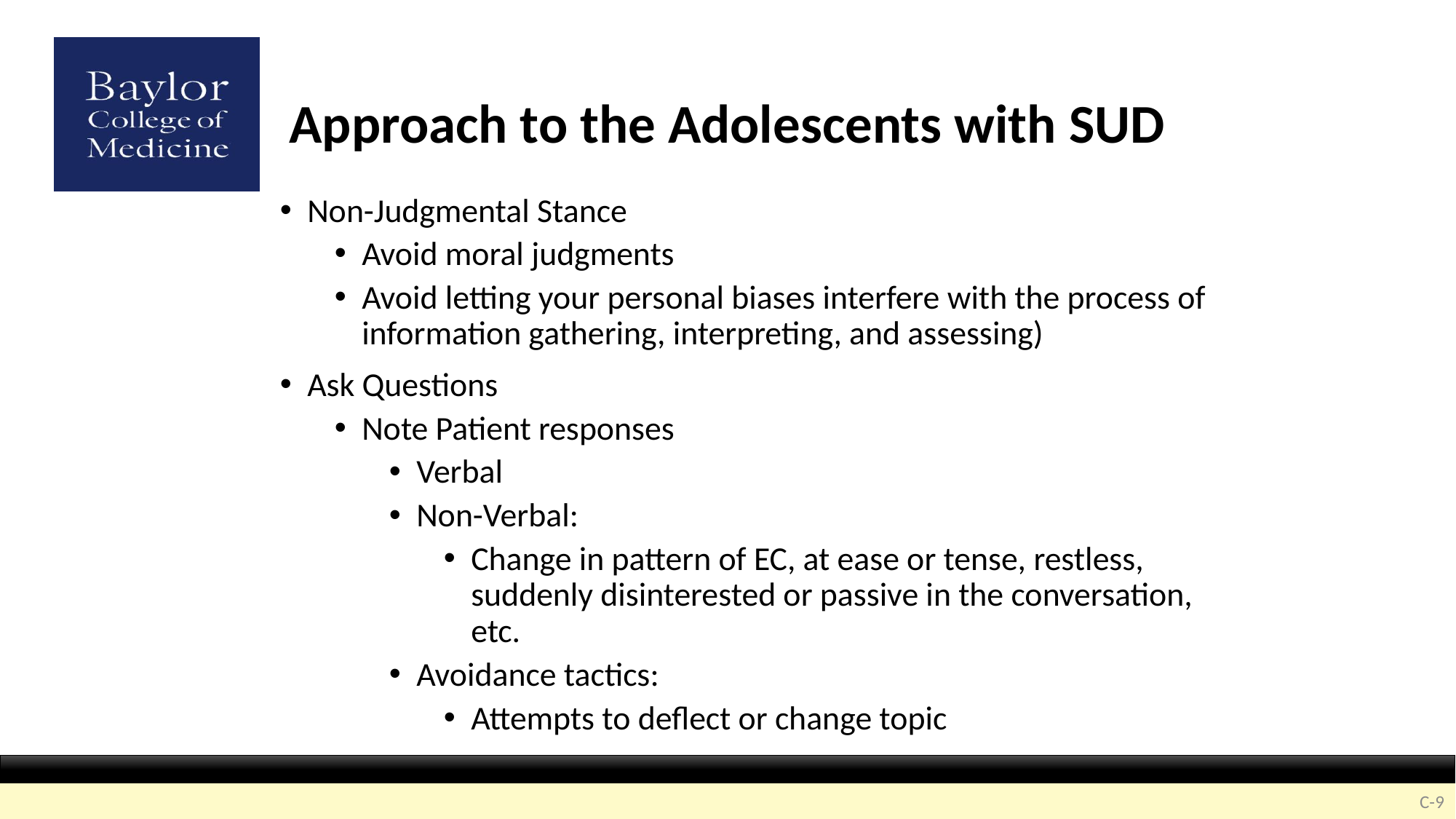

Approach to the Adolescents with SUD
Non-Judgmental Stance
Avoid moral judgments
Avoid letting your personal biases interfere with the process of information gathering, interpreting, and assessing)
Ask Questions
Note Patient responses
Verbal
Non-Verbal:
Change in pattern of EC, at ease or tense, restless, suddenly disinterested or passive in the conversation, etc.
Avoidance tactics:
Attempts to deflect or change topic
C-9

## Slide 10
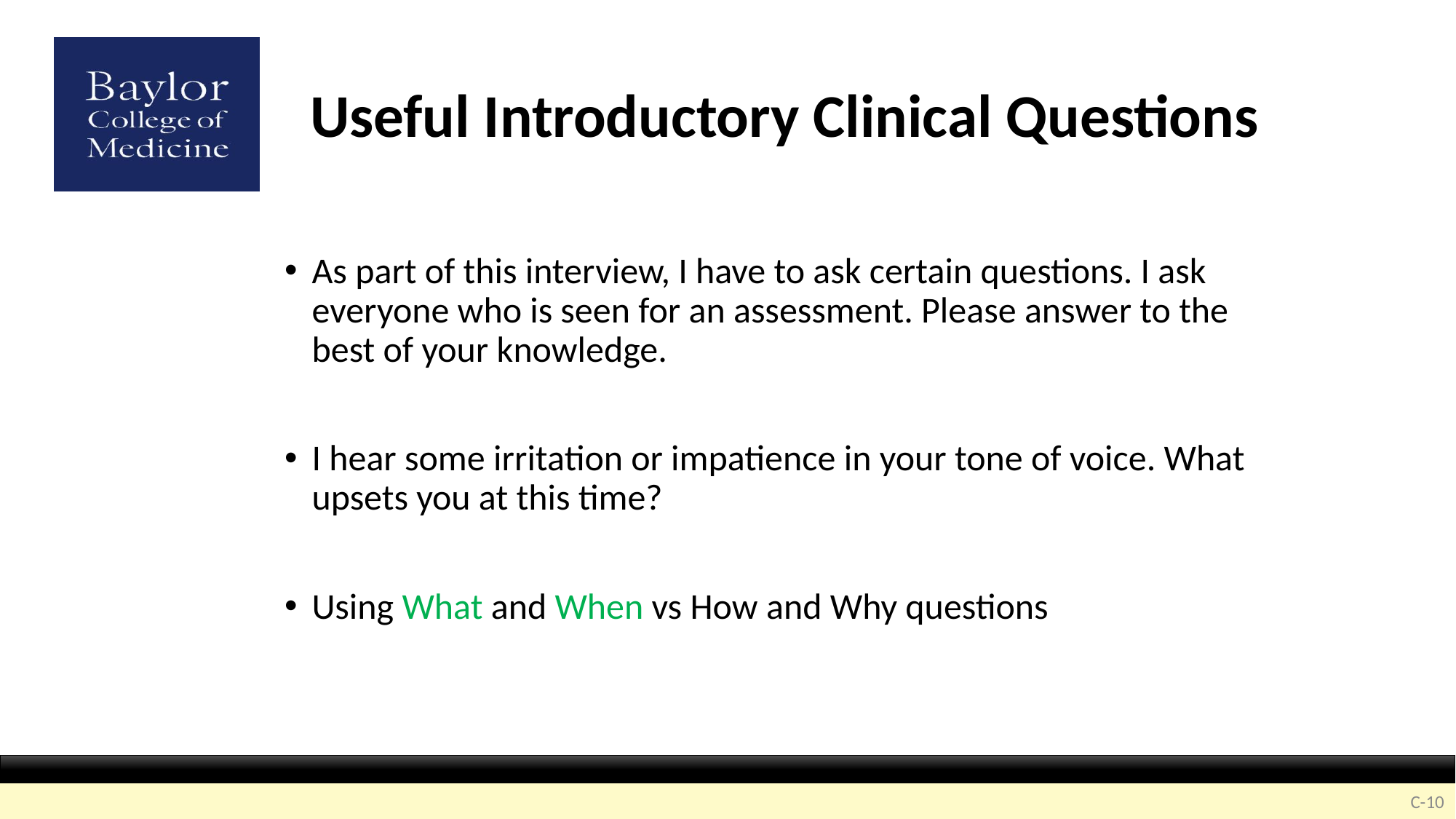

Useful Introductory Clinical Questions
As part of this interview, I have to ask certain questions. I ask everyone who is seen for an assessment. Please answer to the best of your knowledge.
I hear some irritation or impatience in your tone of voice. What upsets you at this time?
Using What and When vs How and Why questions
C-10

## Slide 11
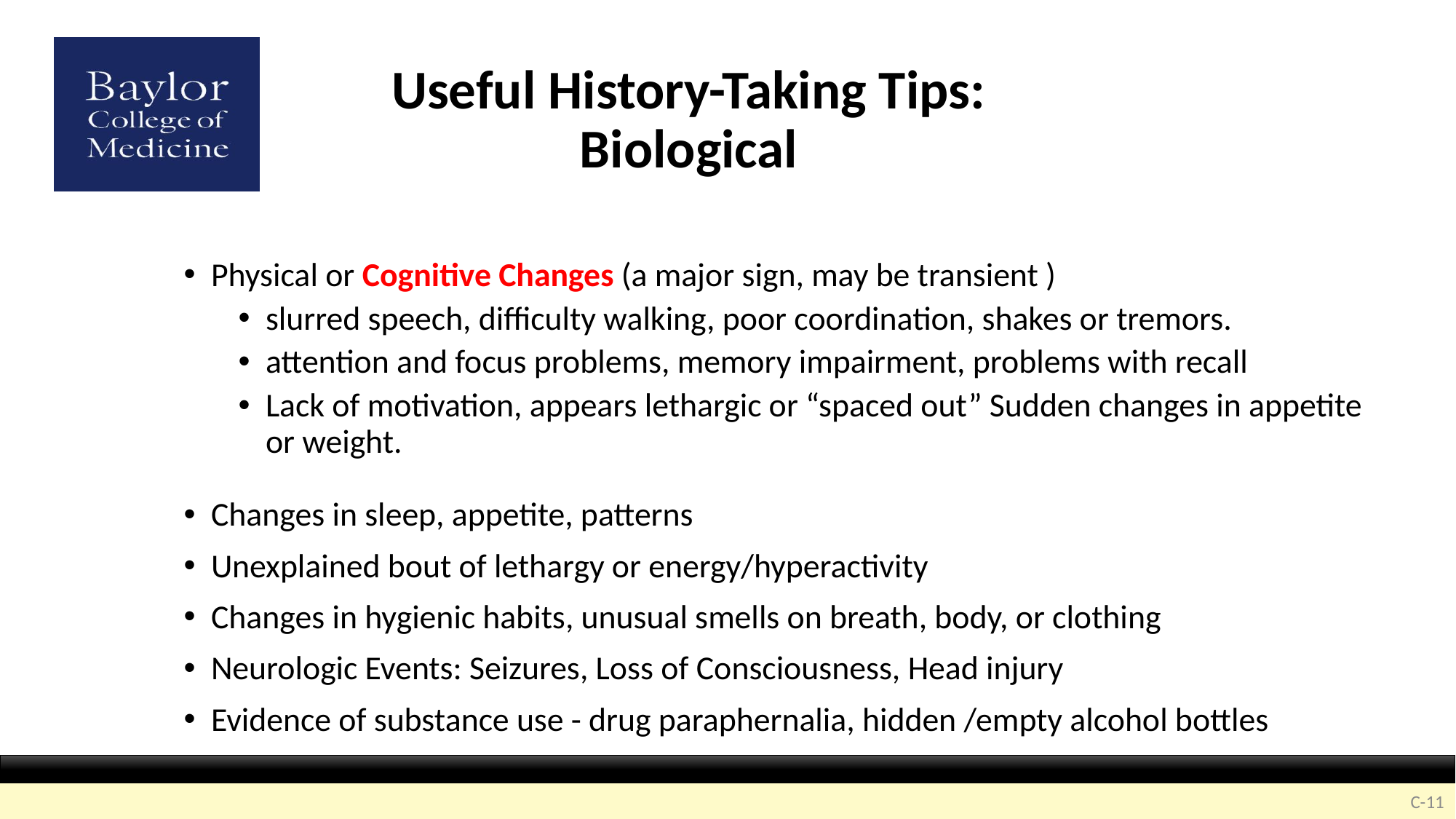

Useful History-Taking Tips:
Biological
Physical or Cognitive Changes (a major sign, may be transient )
slurred speech, difficulty walking, poor coordination, shakes or tremors.
attention and focus problems, memory impairment, problems with recall
Lack of motivation, appears lethargic or “spaced out” Sudden changes in appetite or weight.
Changes in sleep, appetite, patterns
Unexplained bout of lethargy or energy/hyperactivity
Changes in hygienic habits, unusual smells on breath, body, or clothing
Neurologic Events: Seizures, Loss of Consciousness, Head injury
Evidence of substance use - drug paraphernalia, hidden /empty alcohol bottles
C-11

## Slide 12
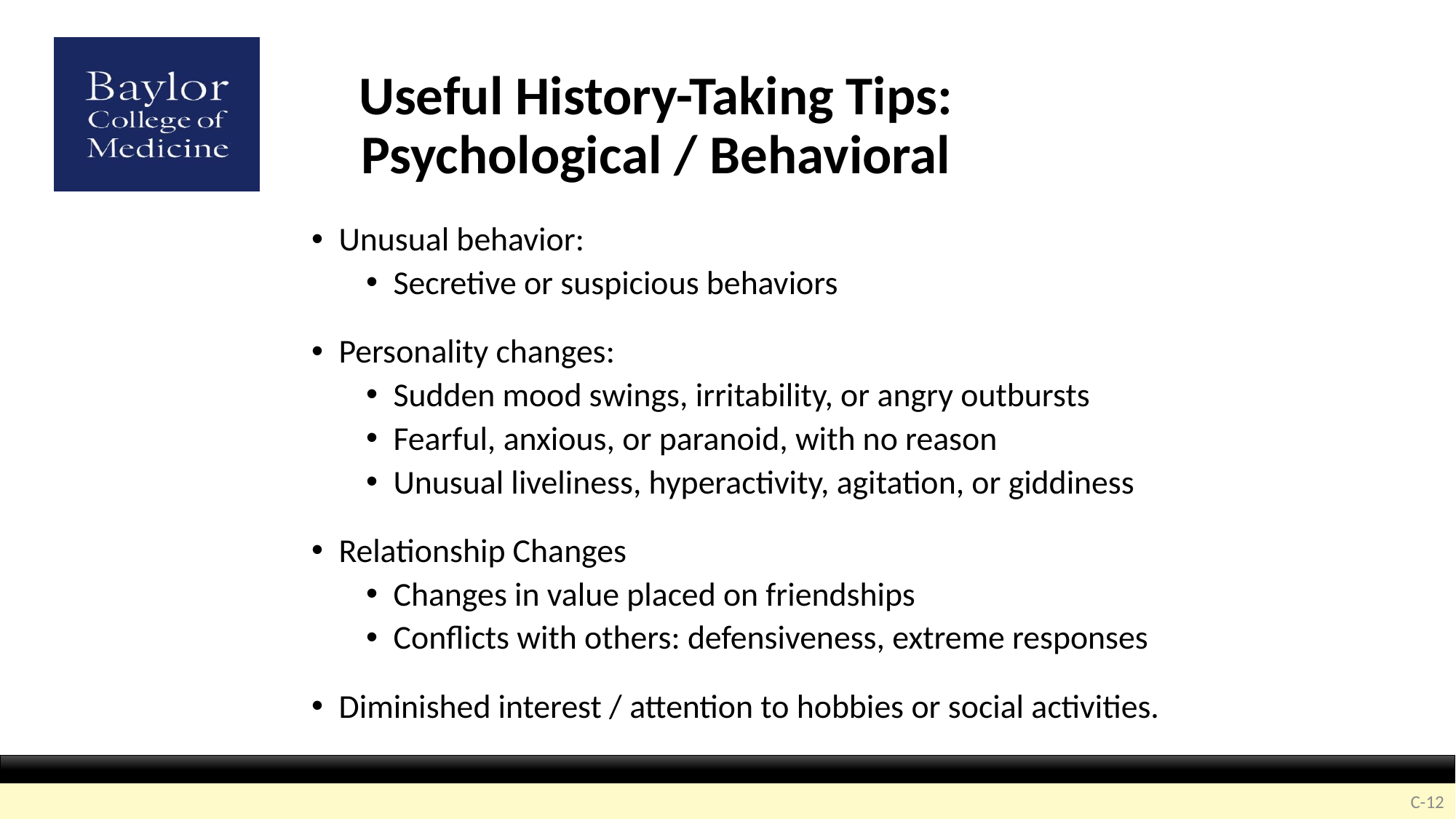

Useful History-Taking Tips:
Psychological / Behavioral
Unusual behavior:
Secretive or suspicious behaviors
Personality changes:
Sudden mood swings, irritability, or angry outbursts
Fearful, anxious, or paranoid, with no reason
Unusual liveliness, hyperactivity, agitation, or giddiness
Relationship Changes
Changes in value placed on friendships
Conflicts with others: defensiveness, extreme responses
Diminished interest / attention to hobbies or social activities.
C-12

## Slide 13
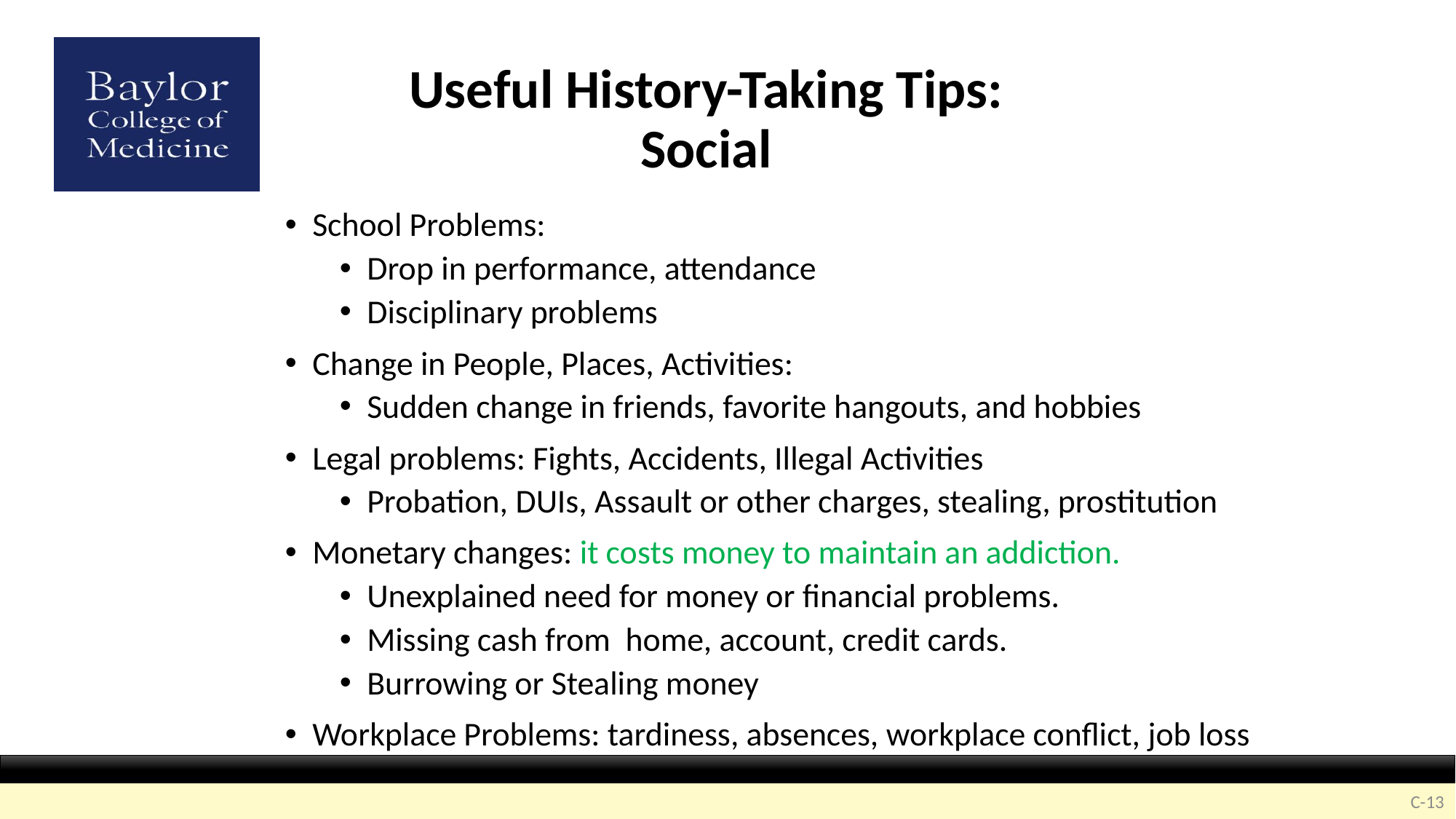

Useful History-Taking Tips:
Social
School Problems:
Drop in performance, attendance
Disciplinary problems
Change in People, Places, Activities:
Sudden change in friends, favorite hangouts, and hobbies
Legal problems: Fights, Accidents, Illegal Activities
Probation, DUIs, Assault or other charges, stealing, prostitution
Monetary changes: it costs money to maintain an addiction.
Unexplained need for money or financial problems.
Missing cash from home, account, credit cards.
Burrowing or Stealing money
Workplace Problems: tardiness, absences, workplace conflict, job loss
C-13

## Slide 14
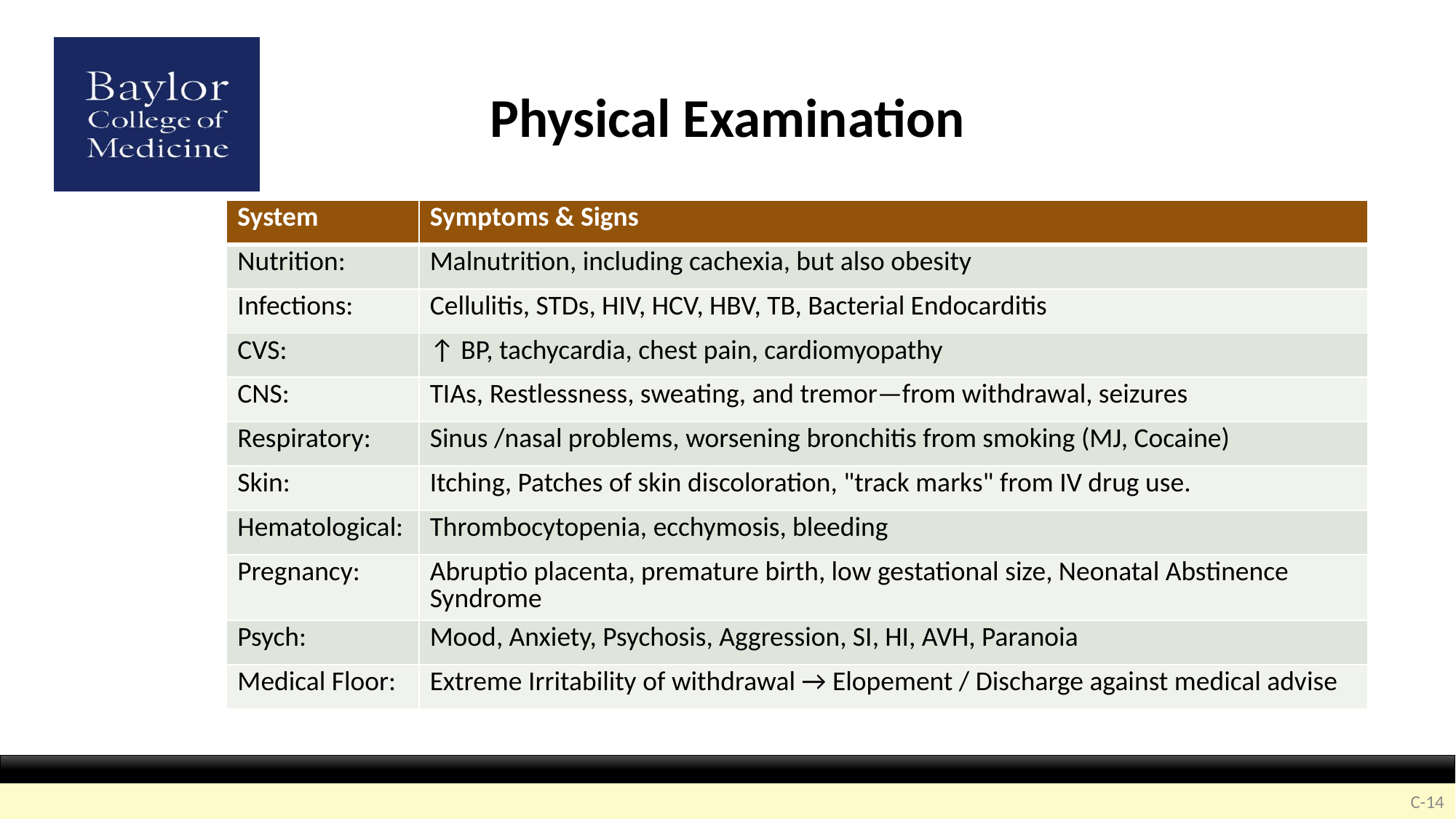

Physical Examination
| System | Symptoms & Signs |
| --- | --- |
| Nutrition: | Malnutrition, including cachexia, but also obesity |
| Infections: | Cellulitis, STDs, HIV, HCV, HBV, TB, Bacterial Endocarditis |
| CVS: | ↑ BP, tachycardia, chest pain, cardiomyopathy |
| CNS: | TIAs, Restlessness, sweating, and tremor—from withdrawal, seizures |
| Respiratory: | Sinus /nasal problems, worsening bronchitis from smoking (MJ, Cocaine) |
| Skin: | Itching, Patches of skin discoloration, "track marks" from IV drug use. |
| Hematological: | Thrombocytopenia, ecchymosis, bleeding |
| Pregnancy: | Abruptio placenta, premature birth, low gestational size, Neonatal Abstinence Syndrome |
| Psych: | Mood, Anxiety, Psychosis, Aggression, SI, HI, AVH, Paranoia |
| Medical Floor: | Extreme Irritability of withdrawal → Elopement / Discharge against medical advise |
C-14

## Slide 15
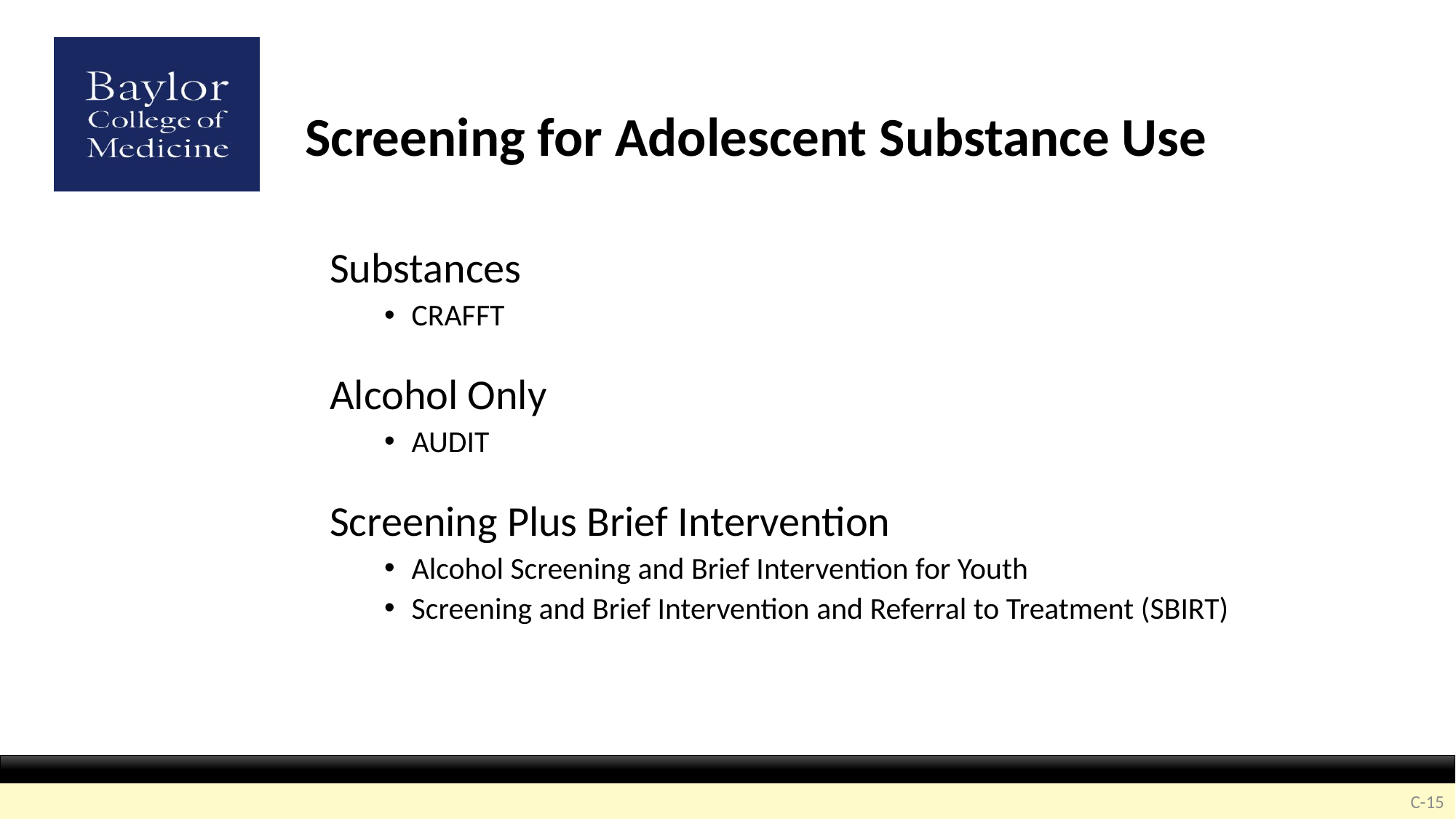

Screening for Adolescent Substance Use
Substances
CRAFFT
Alcohol Only
AUDIT
Screening Plus Brief Intervention
Alcohol Screening and Brief Intervention for Youth
Screening and Brief Intervention and Referral to Treatment (SBIRT)
C-15

## Slide 16
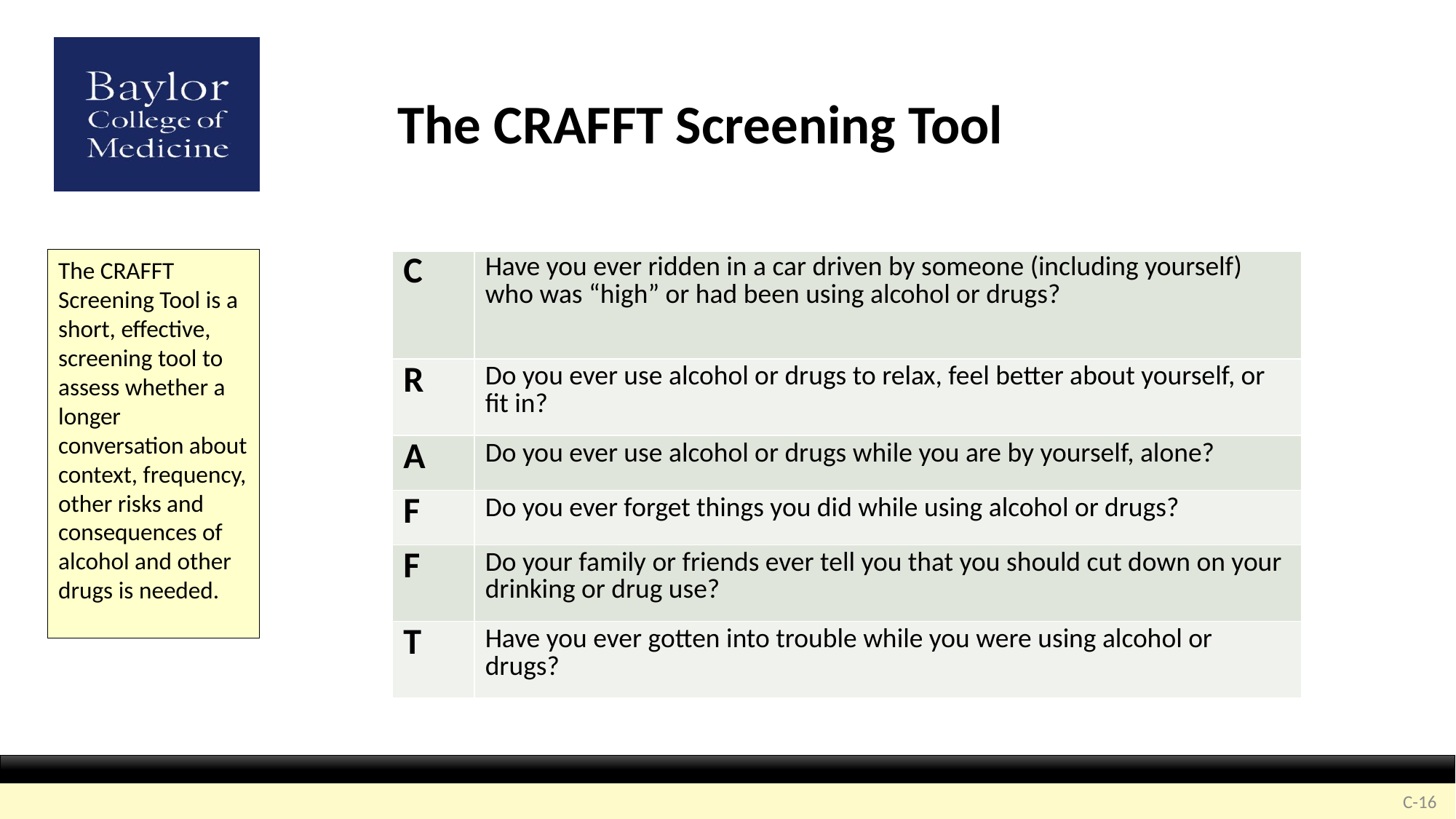

The CRAFFT Screening Tool
| | |
| --- | --- |
| C | Have you ever ridden in a car driven by someone (including yourself) who was “high” or had been using alcohol or drugs? |
| R | Do you ever use alcohol or drugs to relax, feel better about yourself, or fit in? |
| A | Do you ever use alcohol or drugs while you are by yourself, alone? |
| F | Do you ever forget things you did while using alcohol or drugs? |
| F | Do your family or friends ever tell you that you should cut down on your drinking or drug use? |
| T | Have you ever gotten into trouble while you were using alcohol or drugs? |
The CRAFFT Screening Tool is a short, effective, screening tool to assess whether a longer conversation about context, frequency, other risks and consequences of alcohol and other drugs is needed.
C-16

## Slide 17
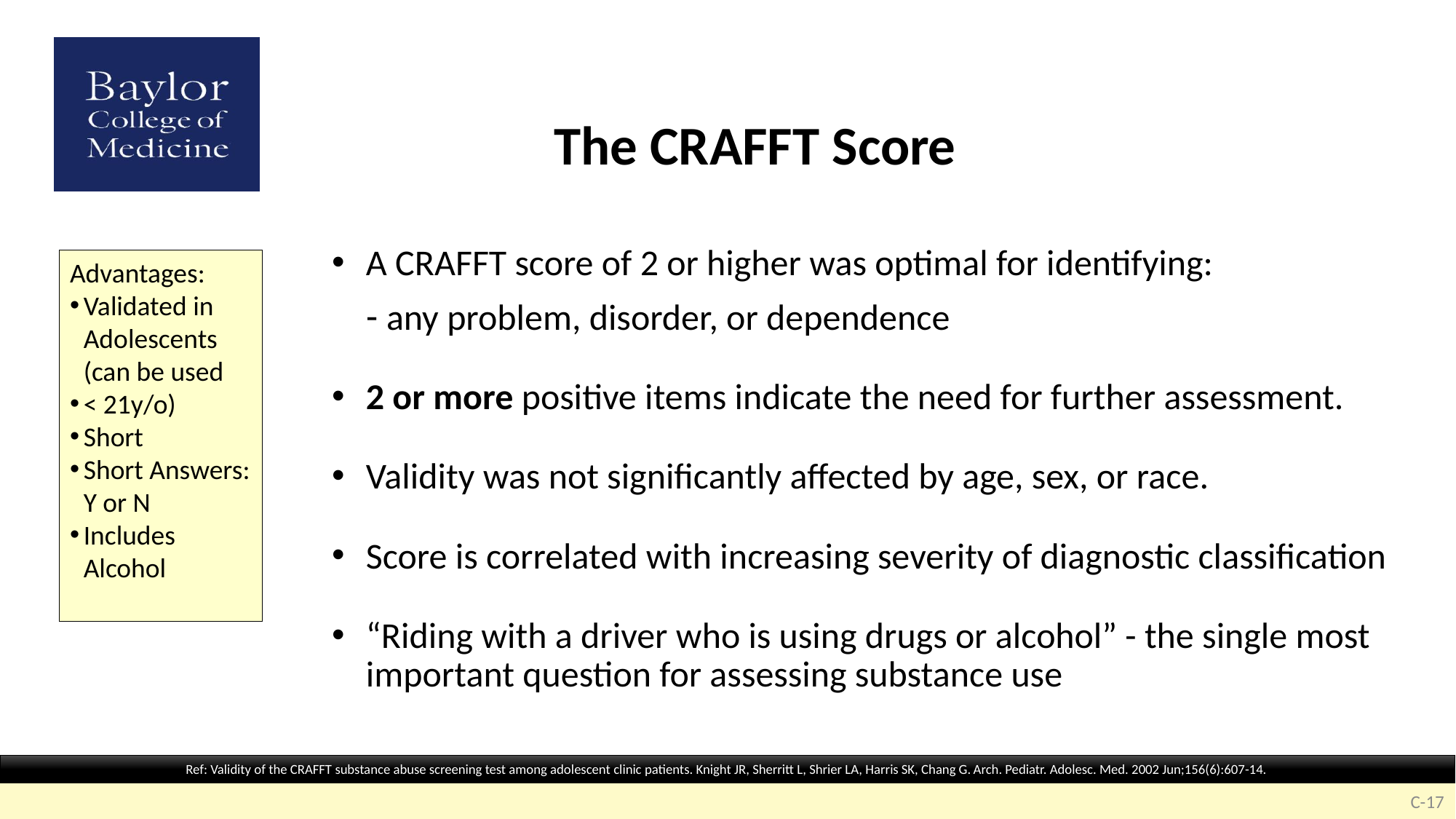

The CRAFFT Score
A CRAFFT score of 2 or higher was optimal for identifying:
any problem, disorder, or dependence
2 or more positive items indicate the need for further assessment.
Validity was not significantly affected by age, sex, or race.
Score is correlated with increasing severity of diagnostic classification
“Riding with a driver who is using drugs or alcohol” - the single most important question for assessing substance use
Advantages:
Validated in Adolescents (can be used
< 21y/o)
Short
Short Answers: Y or N
Includes Alcohol
Ref: Validity of the CRAFFT substance abuse screening test among adolescent clinic patients. Knight JR, Sherritt L, Shrier LA, Harris SK, Chang G. Arch. Pediatr. Adolesc. Med. 2002 Jun;156(6):607-14.
C-17

## Slide 18
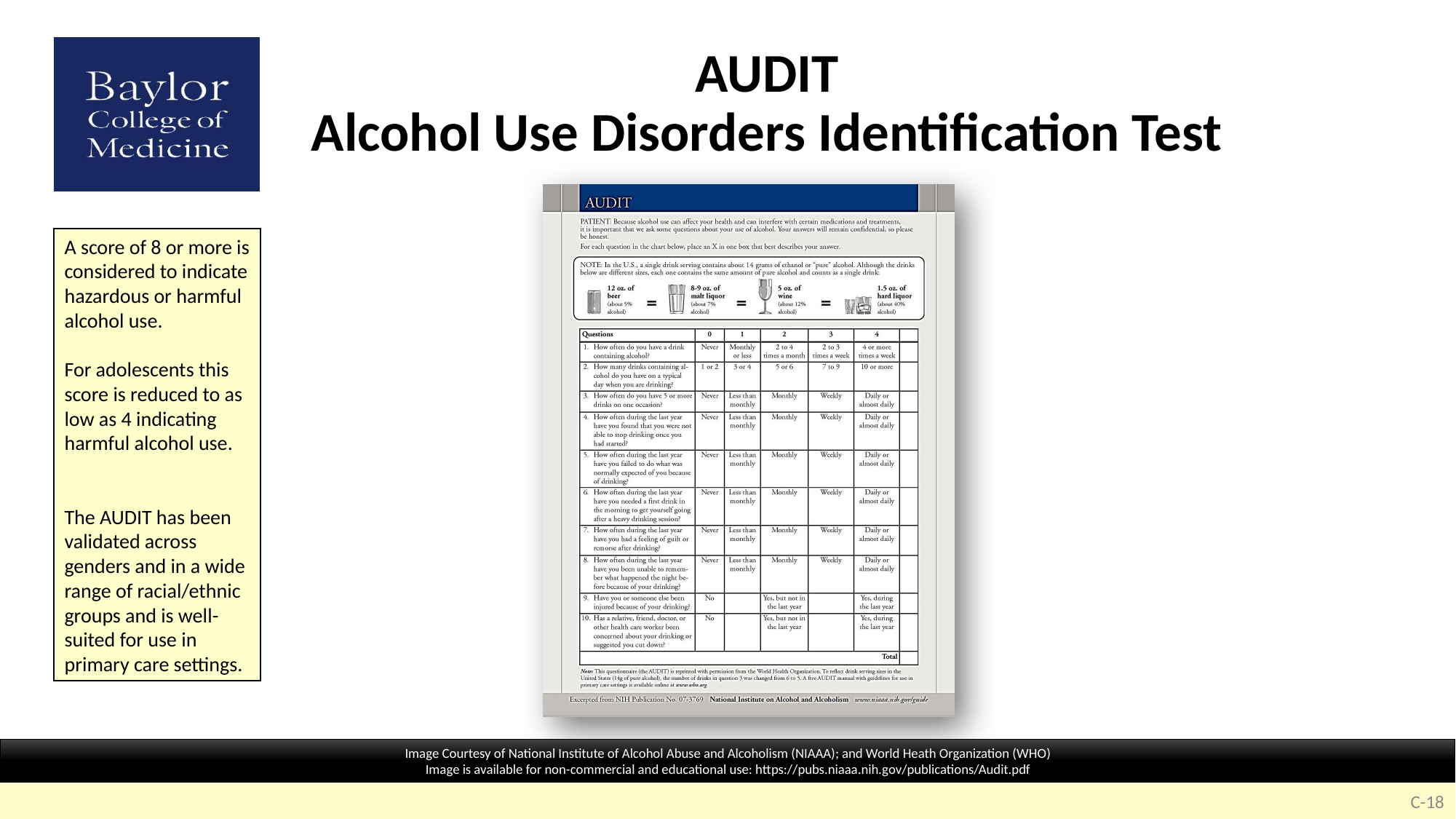

AUDIT
Alcohol Use Disorders Identification Test
A score of 8 or more is considered to indicate hazardous or harmful alcohol use.
For adolescents this score is reduced to as low as 4 indicating harmful alcohol use.
The AUDIT has been validated across genders and in a wide range of racial/ethnic groups and is well-suited for use in primary care settings.
Image Courtesy of National Institute of Alcohol Abuse and Alcoholism (NIAAA); and World Heath Organization (WHO)
Image is available for non-commercial and educational use: https://pubs.niaaa.nih.gov/publications/Audit.pdf
C-18

## Slide 19
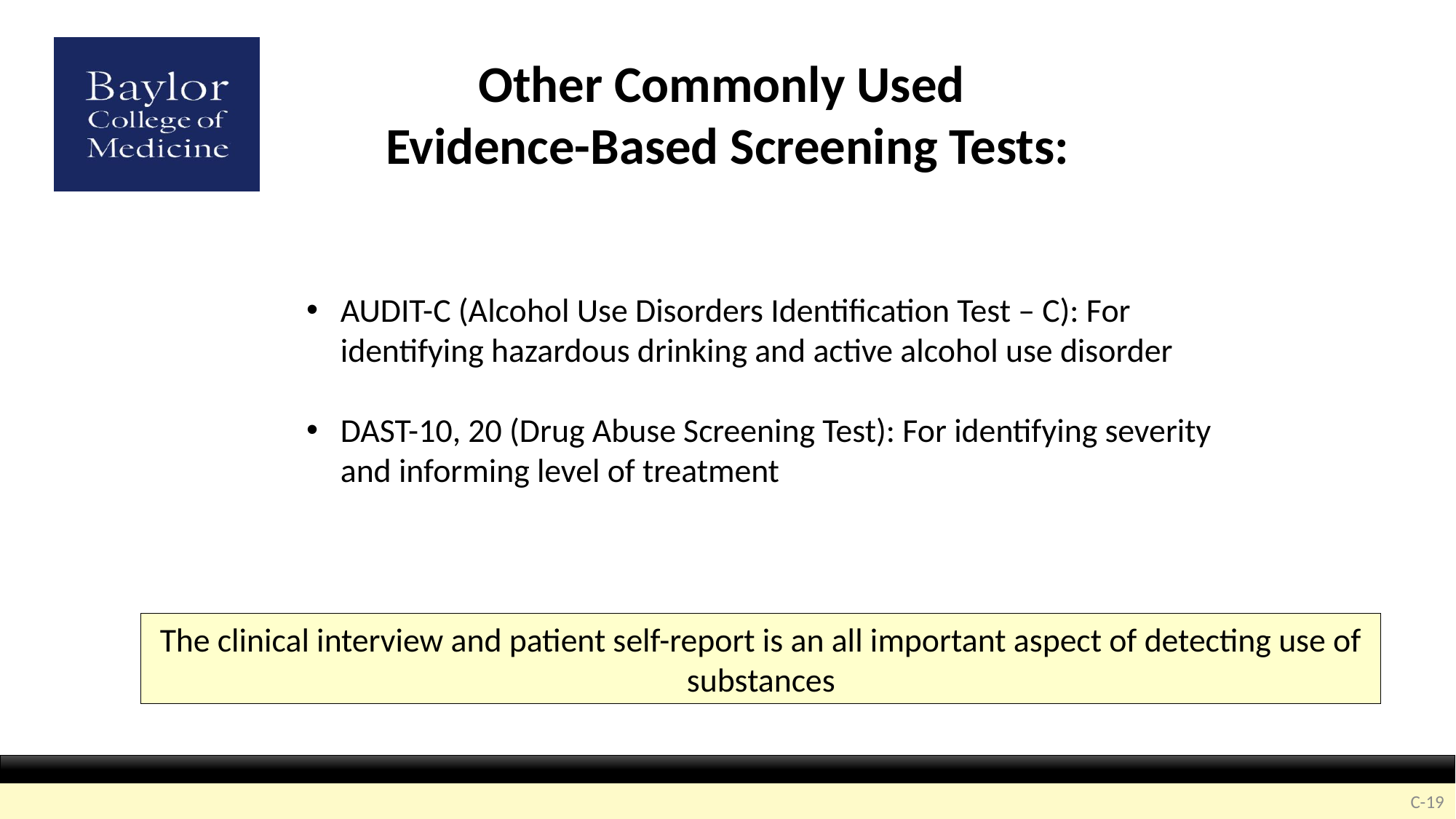

Other Commonly Used
Evidence-Based Screening Tests:
AUDIT-C (Alcohol Use Disorders Identification Test – C): For identifying hazardous drinking and active alcohol use disorder
DAST-10, 20 (Drug Abuse Screening Test): For identifying severity and informing level of treatment
The clinical interview and patient self-report is an all important aspect of detecting use of substances
C-19

## Slide 20
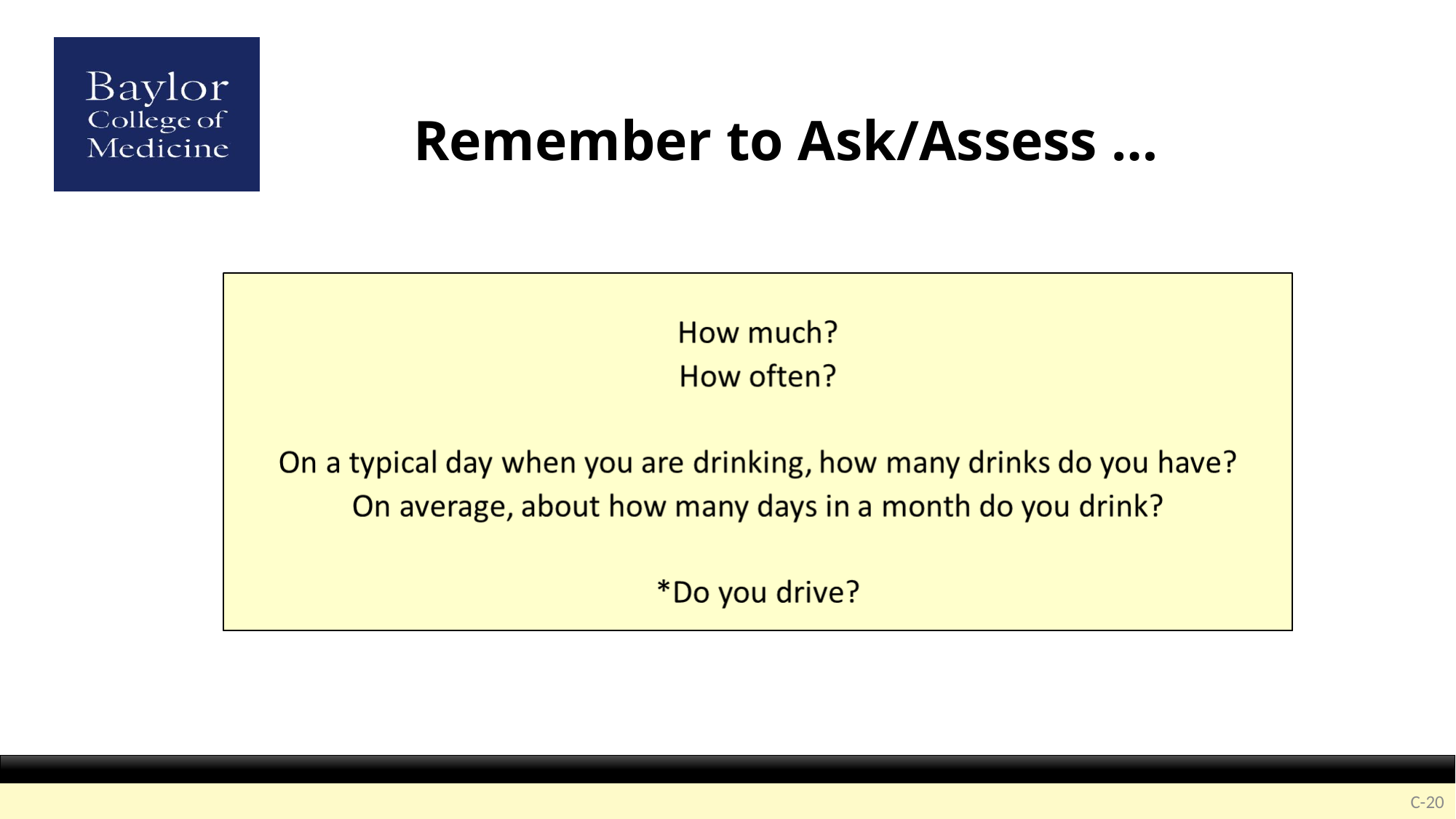

Remember to Ask/Assess …
C-20

## Slide 21
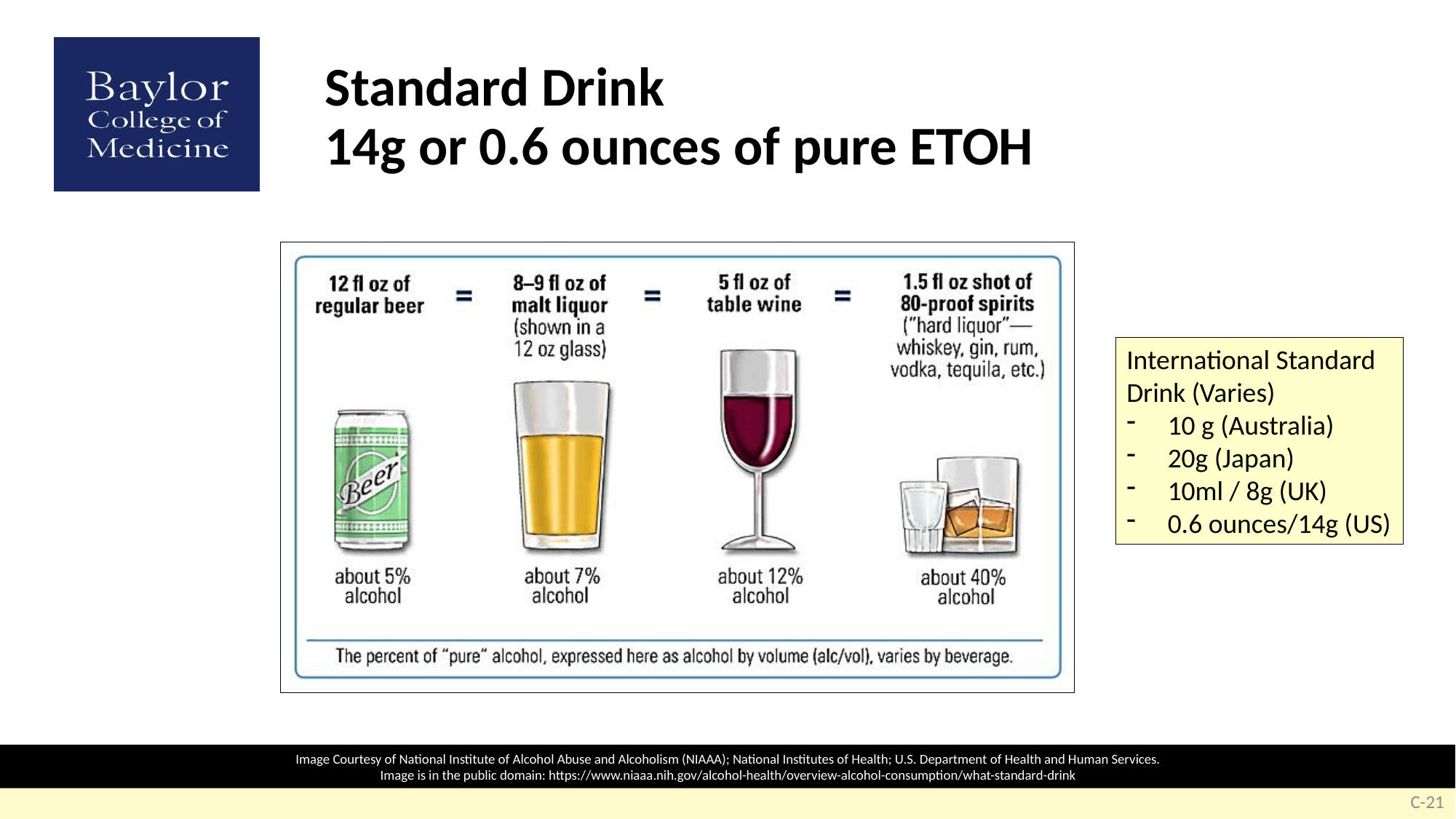

Standard Drink
14g or 0.6 ounces of pure ETOH
International Standard Drink (Varies)
10 g (Australia)
20g (Japan)
10ml / 8g (UK)
0.6 ounces/14g (US)
Image Courtesy of National Institute of Alcohol Abuse and Alcoholism (NIAAA); National Institutes of Health; U.S. Department of Health and Human Services.
Image is in the public domain: https://www.niaaa.nih.gov/alcohol-health/overview-alcohol-consumption/what-standard-drink
C-21

## Slide 22
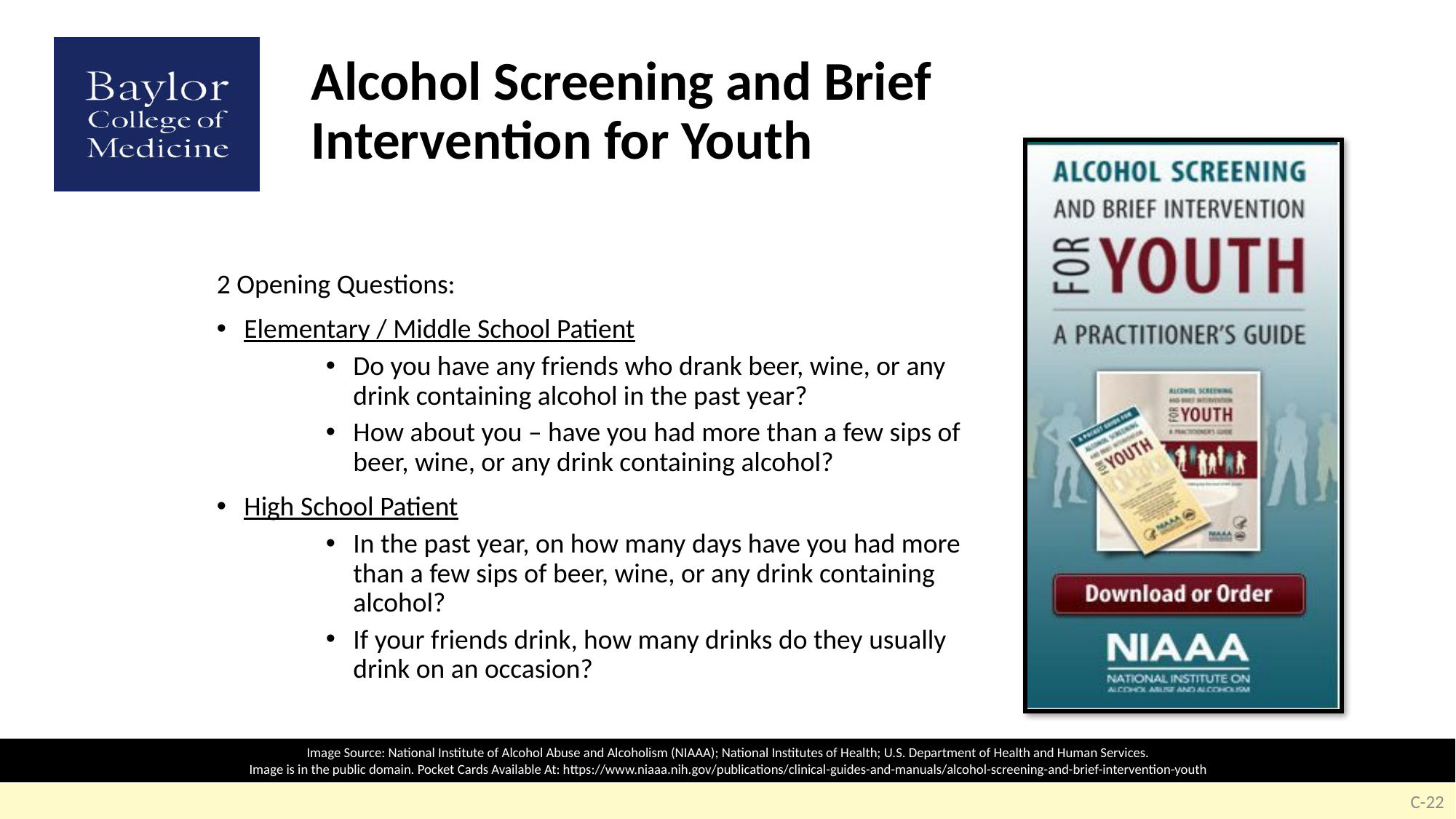

Alcohol Screening and Brief Intervention for Youth
2 Opening Questions:
Elementary / Middle School Patient
Do you have any friends who drank beer, wine, or any drink containing alcohol in the past year?
How about you – have you had more than a few sips of beer, wine, or any drink containing alcohol?
High School Patient
In the past year, on how many days have you had more than a few sips of beer, wine, or any drink containing alcohol?
If your friends drink, how many drinks do they usually drink on an occasion?
Image Source: National Institute of Alcohol Abuse and Alcoholism (NIAAA); National Institutes of Health; U.S. Department of Health and Human Services.
Image is in the public domain. Pocket Cards Available At: https://www.niaaa.nih.gov/publications/clinical-guides-and-manuals/alcohol-screening-and-brief-intervention-youth
C-22

## Slide 23
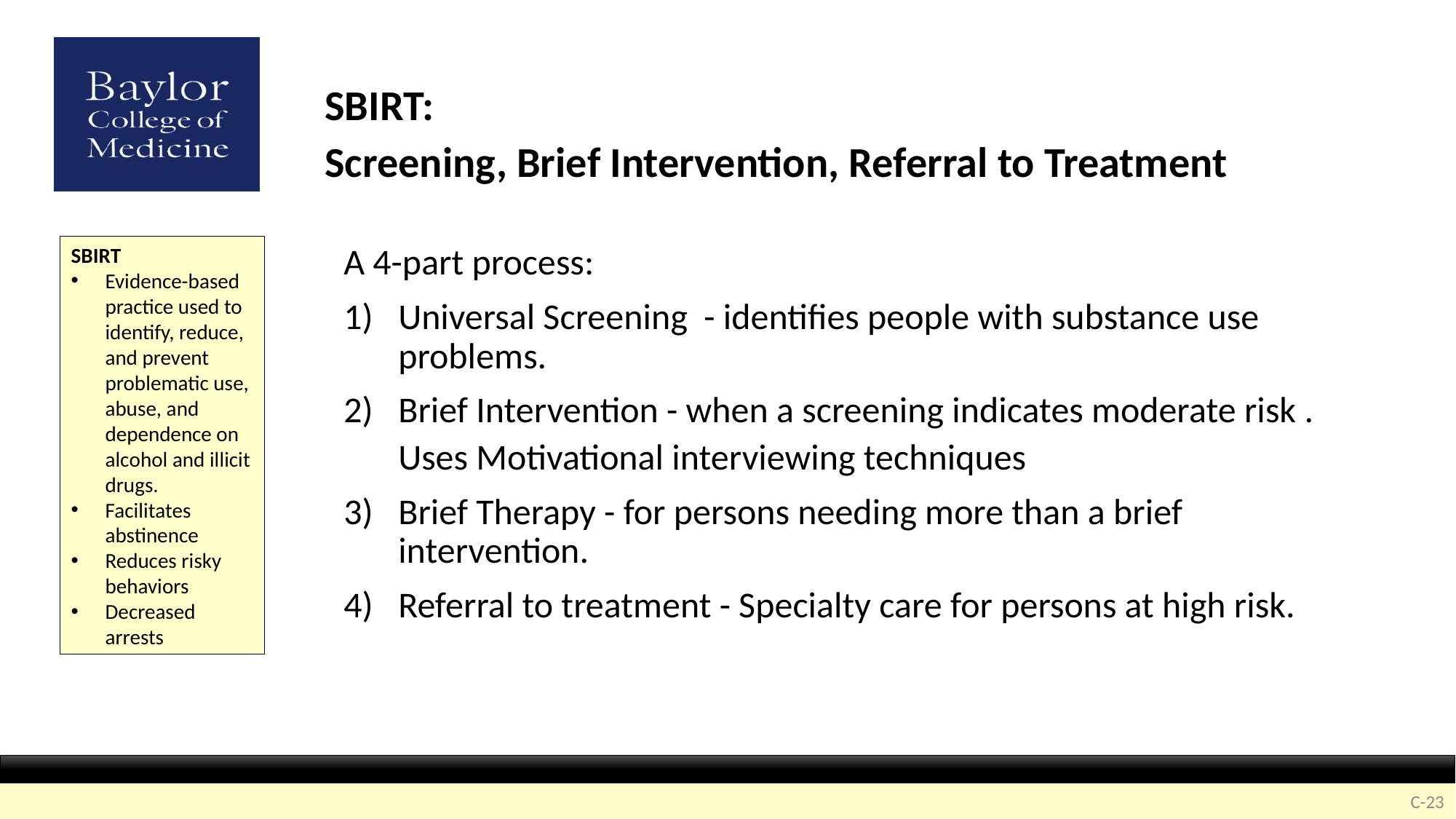

SBIRT:
Screening, Brief Intervention, Referral to Treatment
SBIRT
Evidence-based practice used to identify, reduce, and prevent problematic use, abuse, and dependence on alcohol and illicit drugs.
Facilitates abstinence
Reduces risky behaviors
Decreased arrests
A 4-part process:
Universal Screening - identifies people with substance use problems.
Brief Intervention - when a screening indicates moderate risk .
Uses Motivational interviewing techniques
Brief Therapy - for persons needing more than a brief intervention.
Referral to treatment - Specialty care for persons at high risk.
C-23

## Slide 24
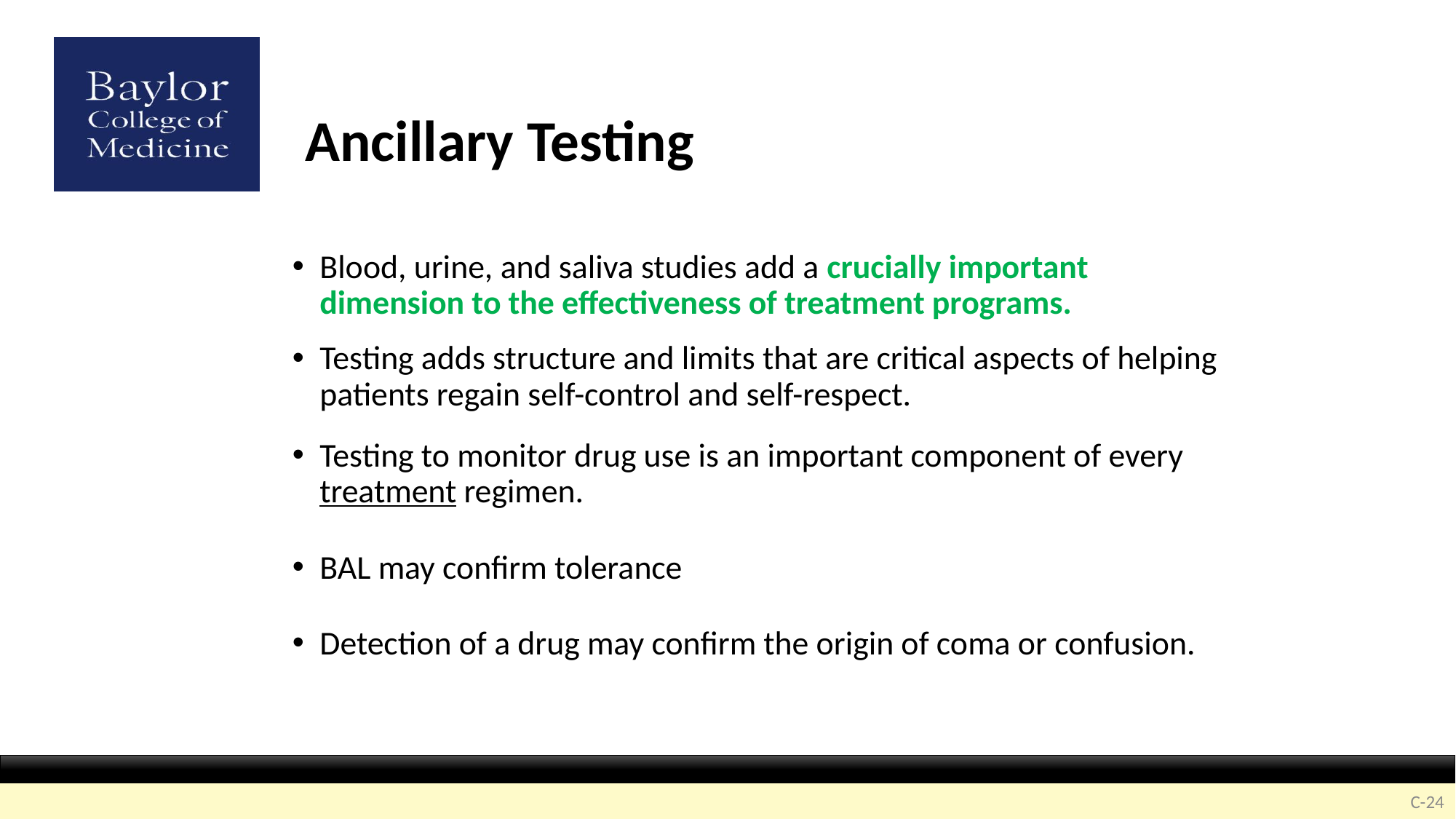

Ancillary Testing
Blood, urine, and saliva studies add a crucially important dimension to the effectiveness of treatment programs.
Testing adds structure and limits that are critical aspects of helping patients regain self-control and self-respect.
Testing to monitor drug use is an important component of every treatment regimen.
BAL may confirm tolerance
Detection of a drug may confirm the origin of coma or confusion.
C-24

## Slide 25
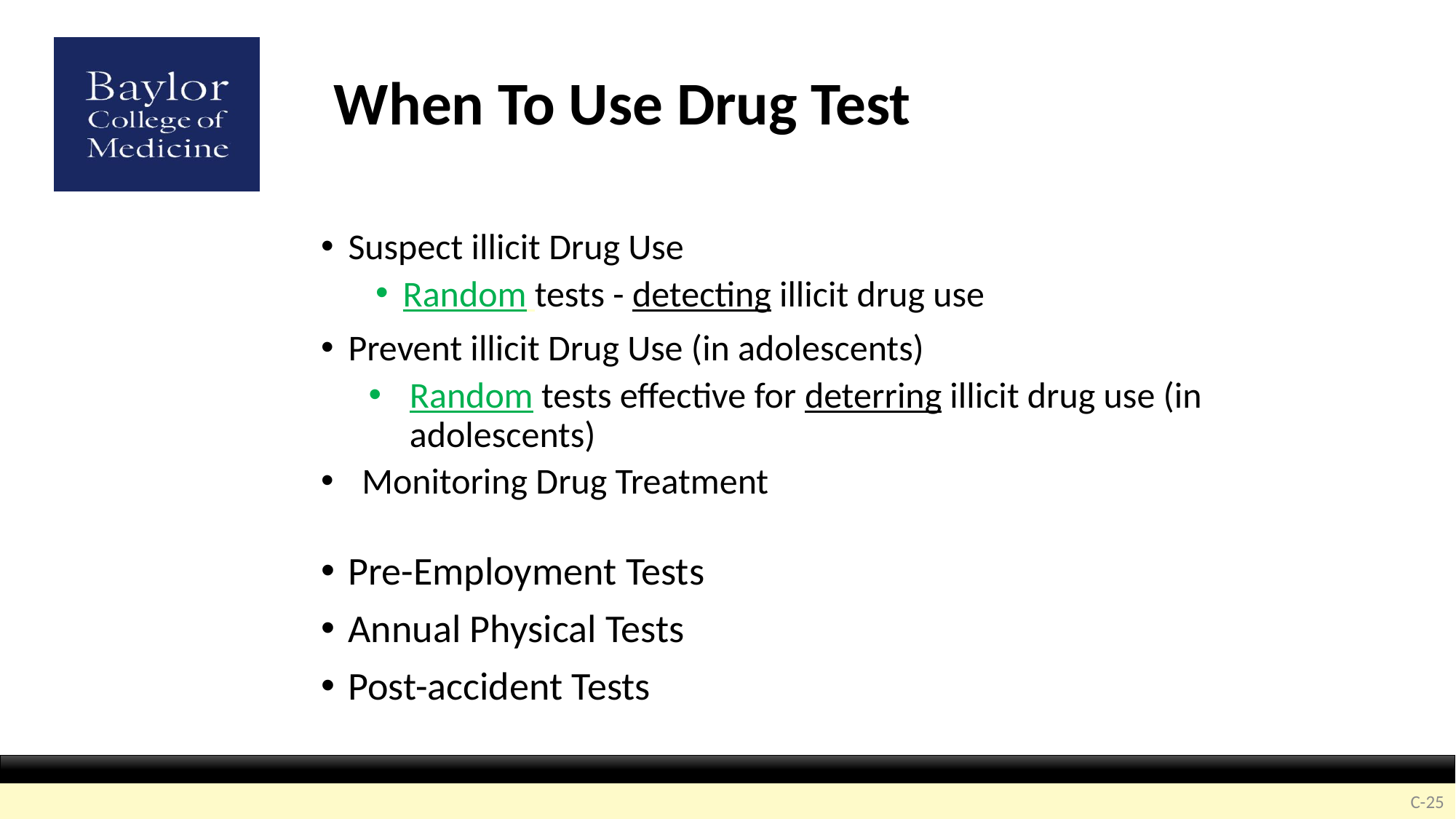

When To Use Drug Test
Suspect illicit Drug Use
Random tests - detecting illicit drug use
Prevent illicit Drug Use (in adolescents)
Random tests effective for deterring illicit drug use (in adolescents)
Monitoring Drug Treatment
Pre-Employment Tests
Annual Physical Tests
Post-accident Tests
C-25

## Slide 26
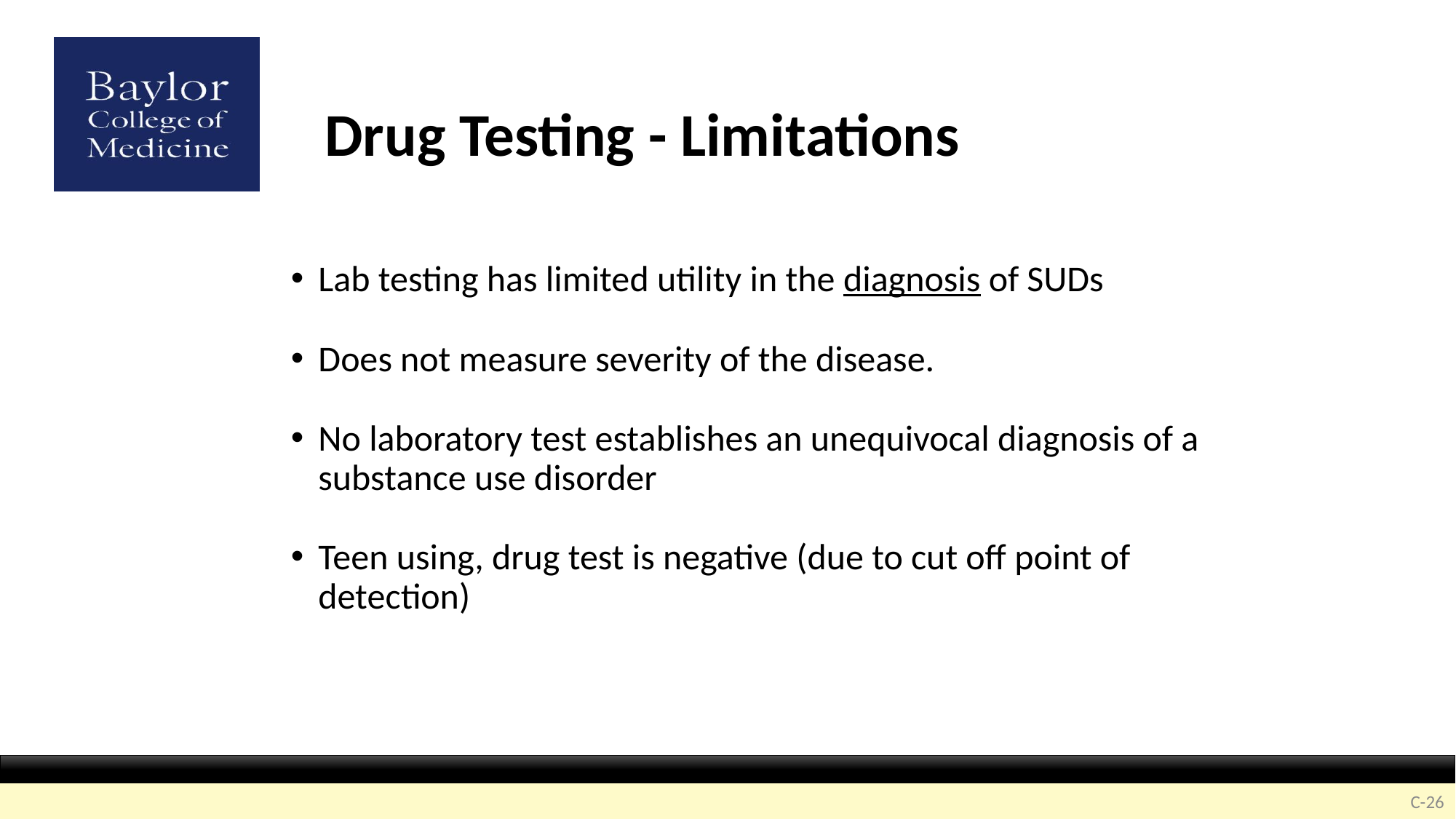

Drug Testing - Limitations
Lab testing has limited utility in the diagnosis of SUDs
Does not measure severity of the disease.
No laboratory test establishes an unequivocal diagnosis of a substance use disorder
Teen using, drug test is negative (due to cut off point of detection)
C-26

## Slide 27
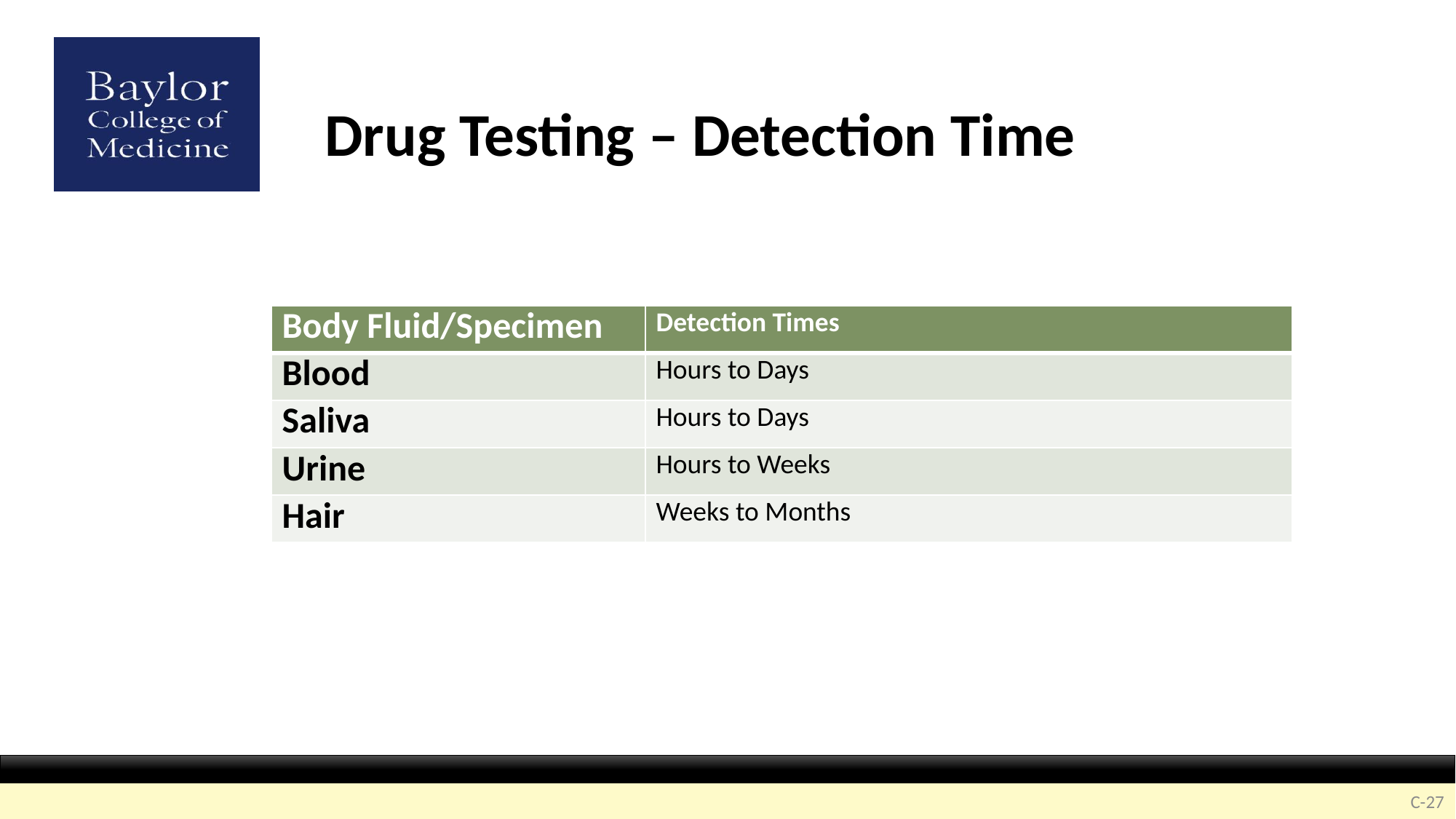

Drug Testing – Detection Time
| Body Fluid/Specimen | Detection Times |
| --- | --- |
| Blood | Hours to Days |
| Saliva | Hours to Days |
| Urine | Hours to Weeks |
| Hair | Weeks to Months |
C-27

## Slide 28
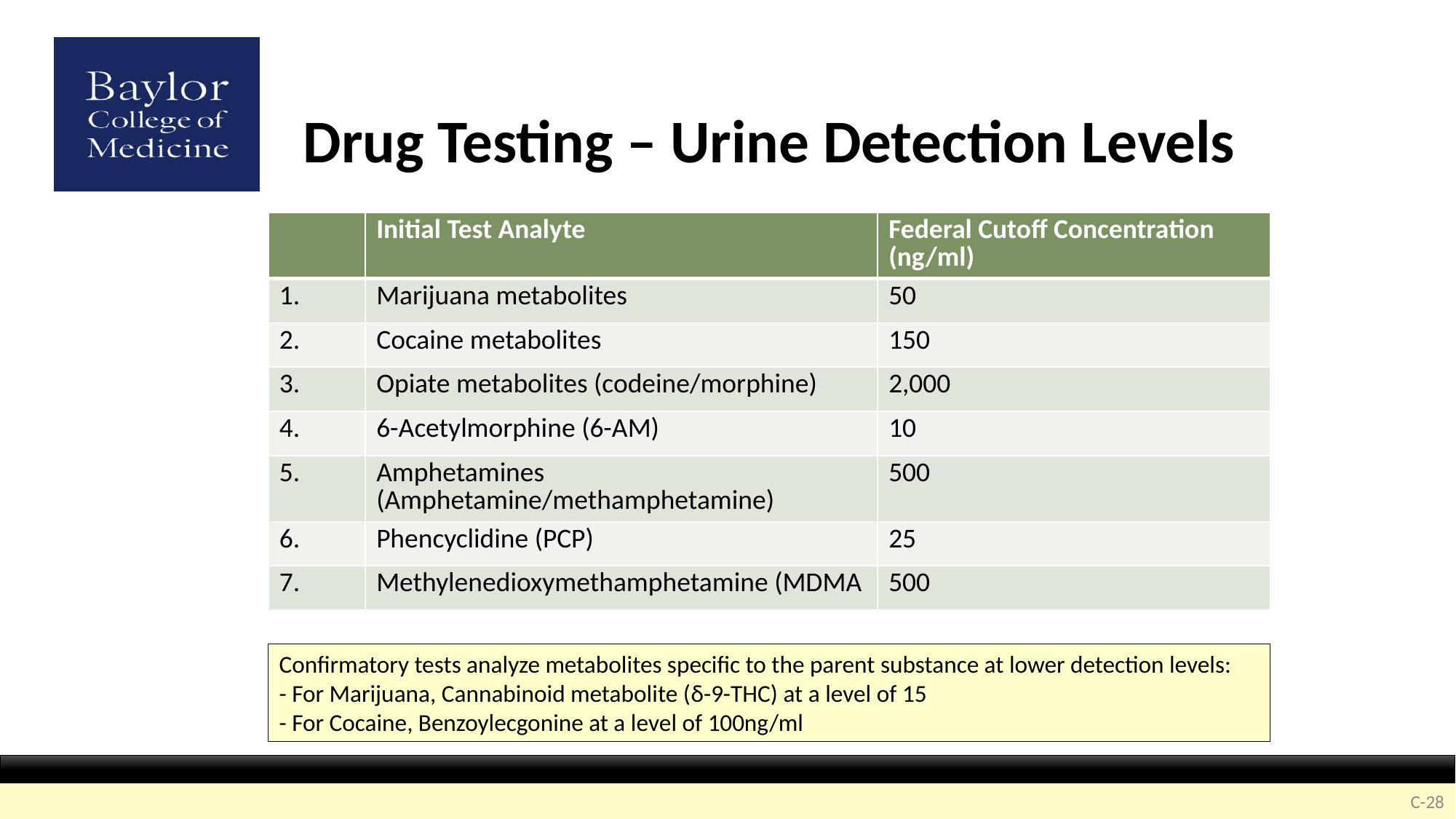

Drug Testing – Urine Detection Levels
| | Initial Test Analyte | Federal Cutoff Concentration (ng/ml) |
| --- | --- | --- |
| 1. | Marijuana metabolites | 50 |
| 2. | Cocaine metabolites | 150 |
| 3. | Opiate metabolites (codeine/morphine) | 2,000 |
| 4. | 6-Acetylmorphine (6-AM) | 10 |
| 5. | Amphetamines (Amphetamine/methamphetamine) | 500 |
| 6. | Phencyclidine (PCP) | 25 |
| 7. | Methylenedioxymethamphetamine (MDMA | 500 |
Confirmatory tests analyze metabolites specific to the parent substance at lower detection levels:
- For Marijuana, Cannabinoid metabolite (δ-9-THC) at a level of 15
- For Cocaine, Benzoylecgonine at a level of 100ng/ml
C-28

## Slide 29
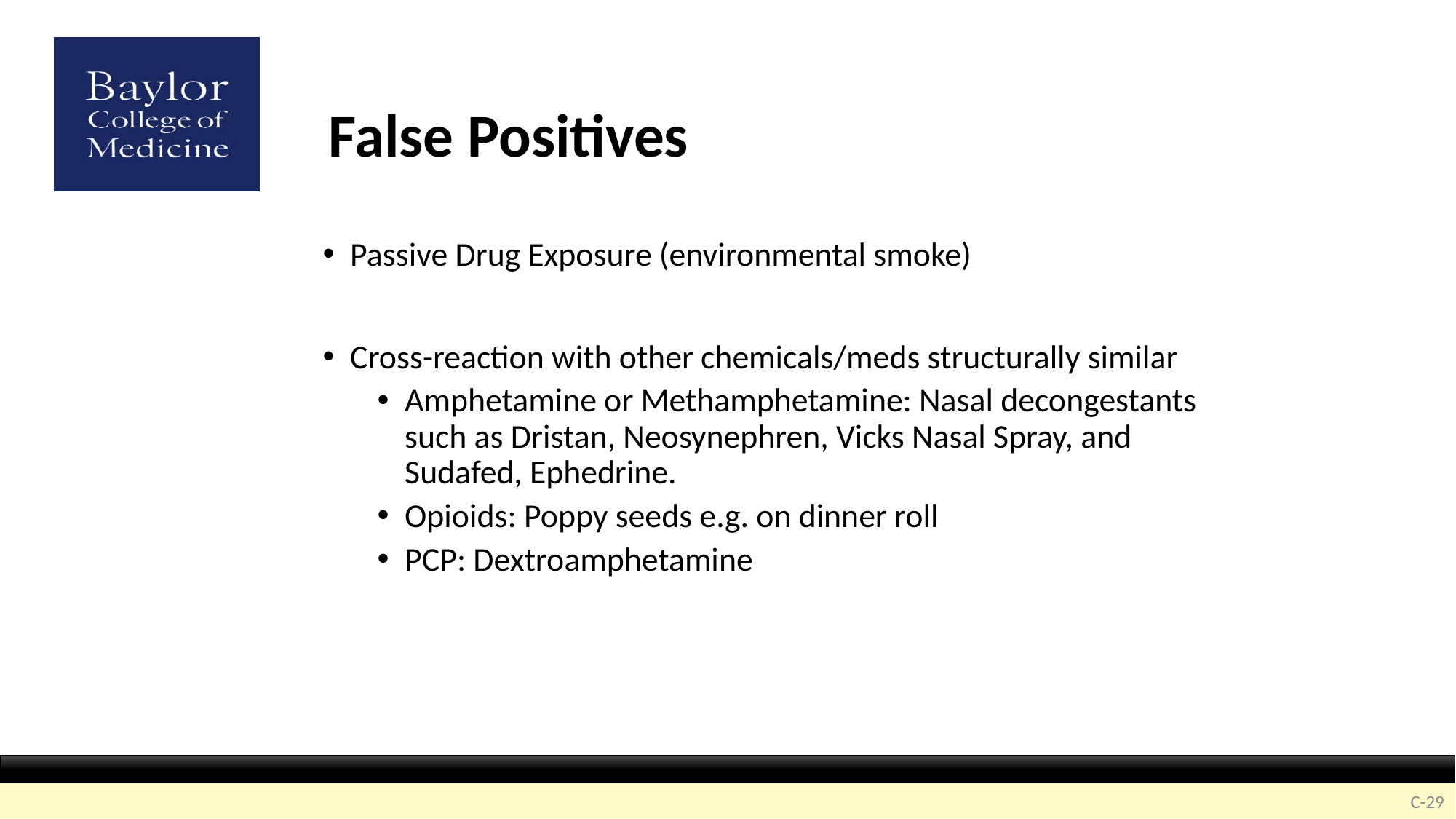

False Positives
Passive Drug Exposure (environmental smoke)
Cross-reaction with other chemicals/meds structurally similar
Amphetamine or Methamphetamine: Nasal decongestants such as Dristan, Neosynephren, Vicks Nasal Spray, and Sudafed, Ephedrine.
Opioids: Poppy seeds e.g. on dinner roll
PCP: Dextroamphetamine
C-29

## Slide 30
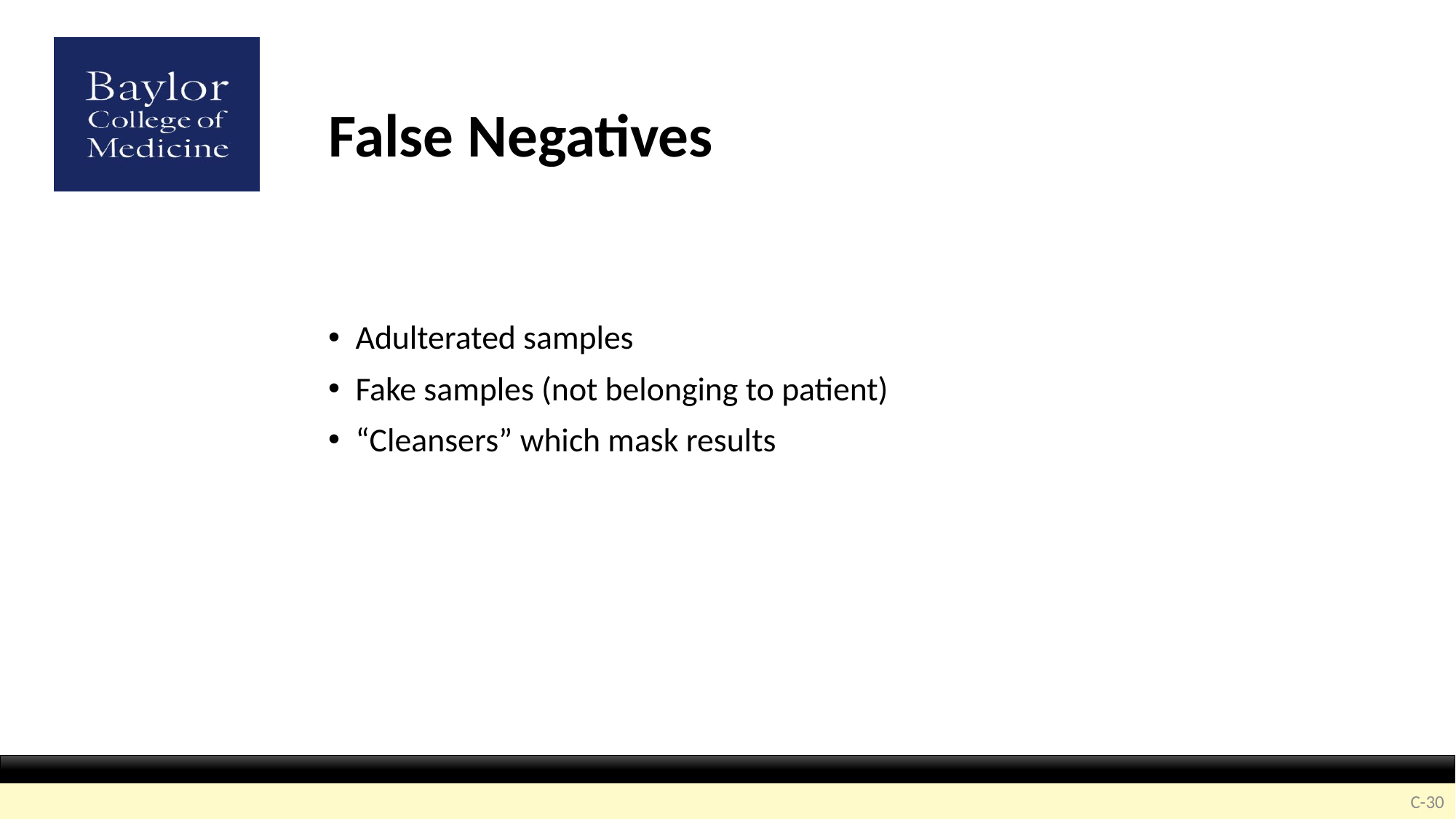

False Negatives
Adulterated samples
Fake samples (not belonging to patient)
“Cleansers” which mask results
C-30

## Slide 31
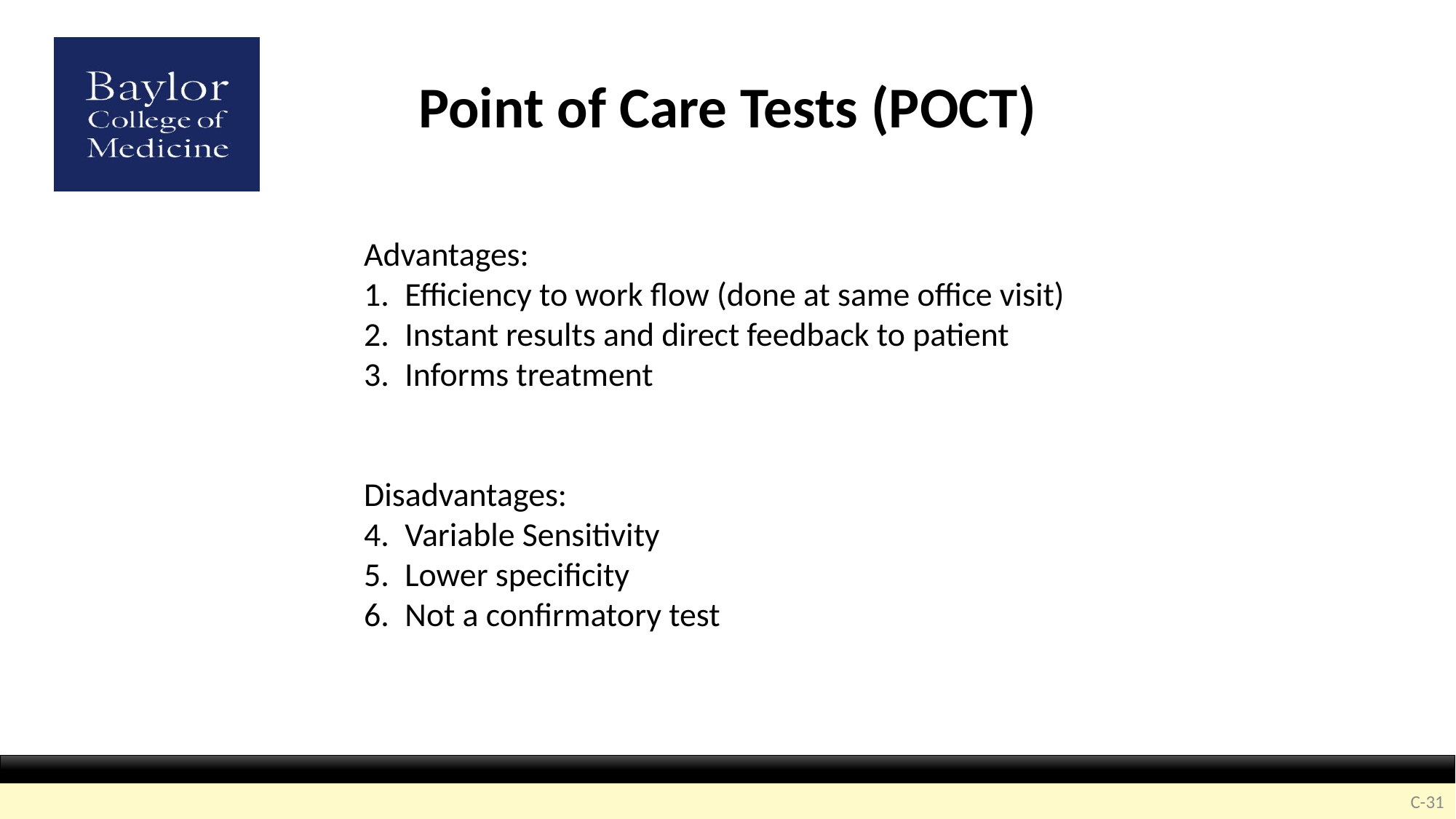

Point of Care Tests (POCT)
Advantages:
Efficiency to work flow (done at same office visit)
Instant results and direct feedback to patient
Informs treatment
Disadvantages:
Variable Sensitivity
Lower specificity
Not a confirmatory test
C-31

## Slide 32
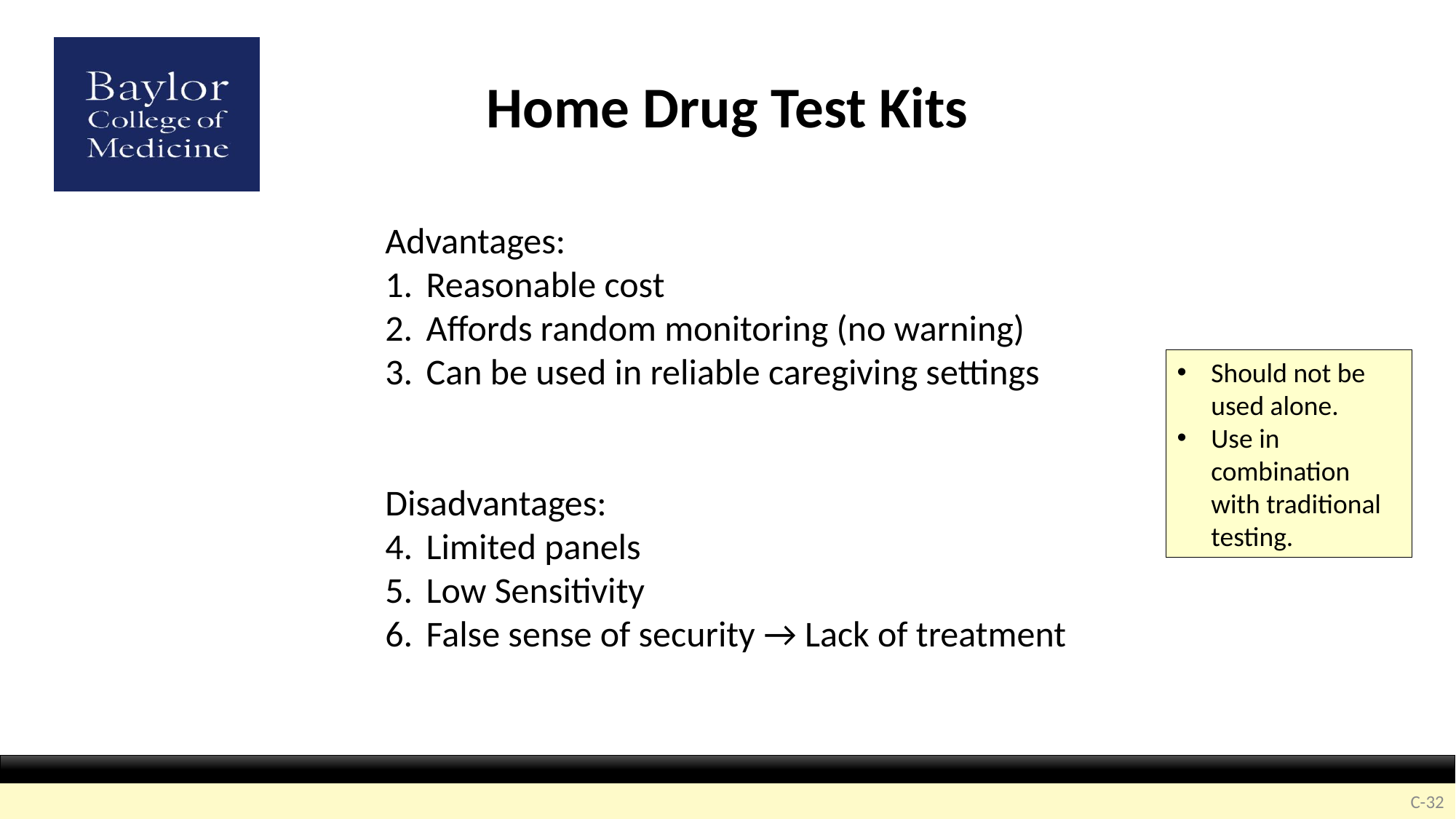

Home Drug Test Kits
Advantages:
Reasonable cost
Affords random monitoring (no warning)
Can be used in reliable caregiving settings
Disadvantages:
Limited panels
Low Sensitivity
False sense of security → Lack of treatment
Should not be used alone.
Use in combination with traditional testing.
C-32

## Slide 33
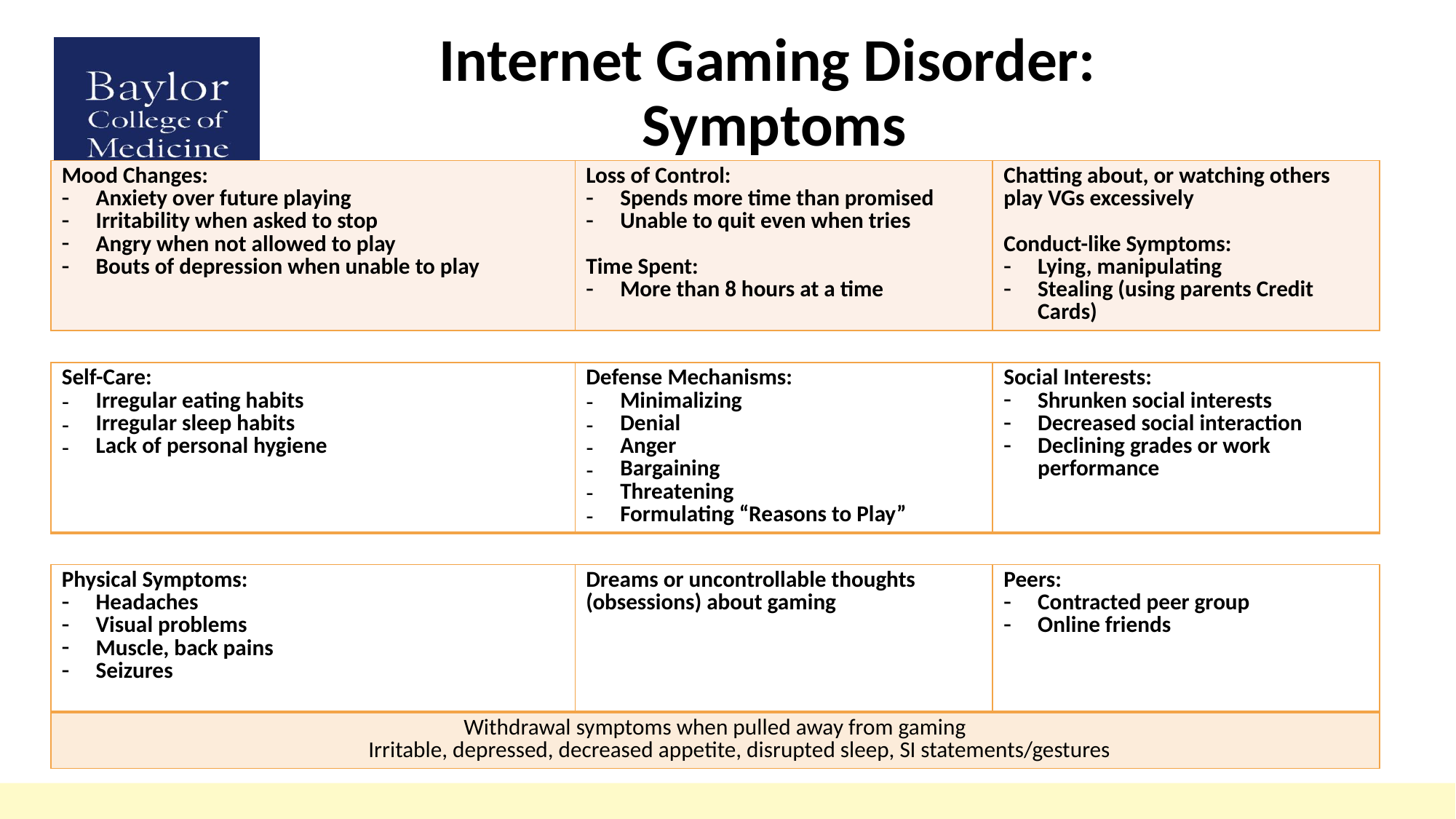

Internet Gaming Disorder:
Symptoms
| Mood Changes: Anxiety over future playing Irritability when asked to stop Angry when not allowed to play Bouts of depression when unable to play | Loss of Control: Spends more time than promised Unable to quit even when tries Time Spent: More than 8 hours at a time | Chatting about, or watching others play VGs excessively Conduct-like Symptoms: Lying, manipulating Stealing (using parents Credit Cards) |
| --- | --- | --- |
| Self-Care: Irregular eating habits Irregular sleep habits Lack of personal hygiene | Defense Mechanisms: Minimalizing Denial Anger Bargaining Threatening Formulating “Reasons to Play” | Social Interests: Shrunken social interests Decreased social interaction Declining grades or work performance |
| --- | --- | --- |
| Physical Symptoms: Headaches Visual problems Muscle, back pains Seizures | Dreams or uncontrollable thoughts (obsessions) about gaming | Peers: Contracted peer group Online friends |
| --- | --- | --- |
| Withdrawal symptoms when pulled away from gaming Irritable, depressed, decreased appetite, disrupted sleep, SI statements/gestures | | |

## Slide 34
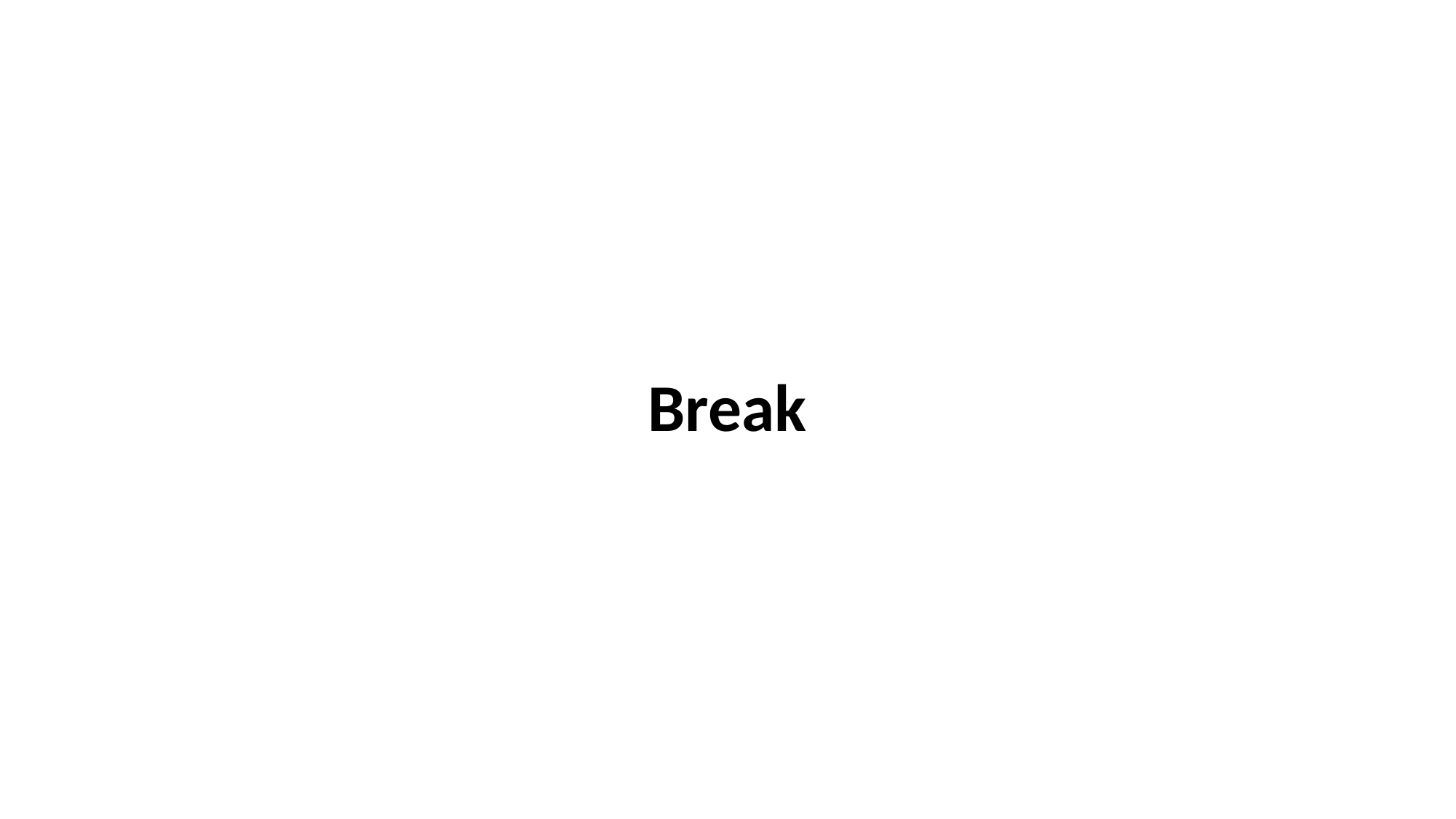

# Break

## Slide 35
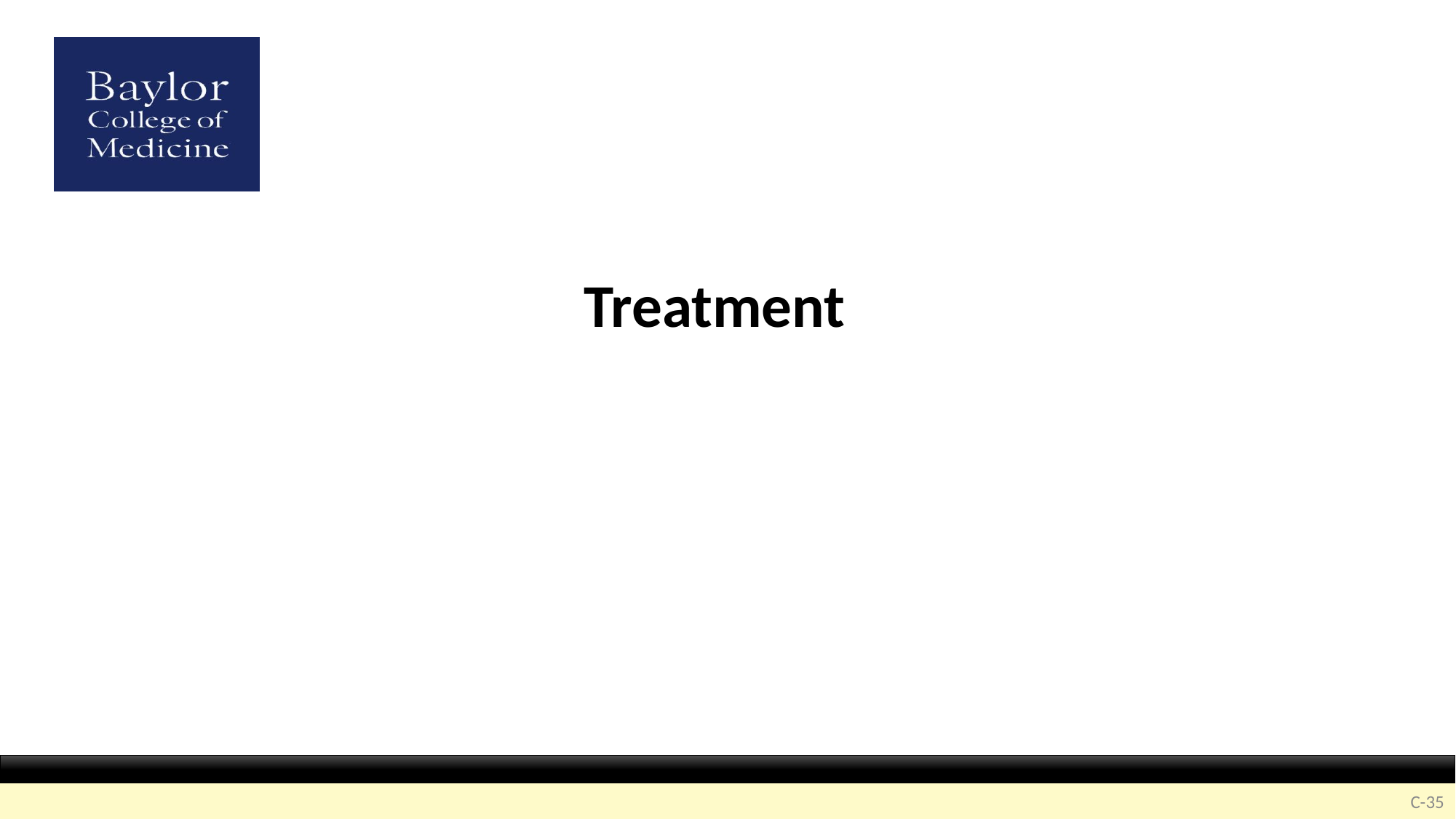

Treatment
C-35

## Slide 36
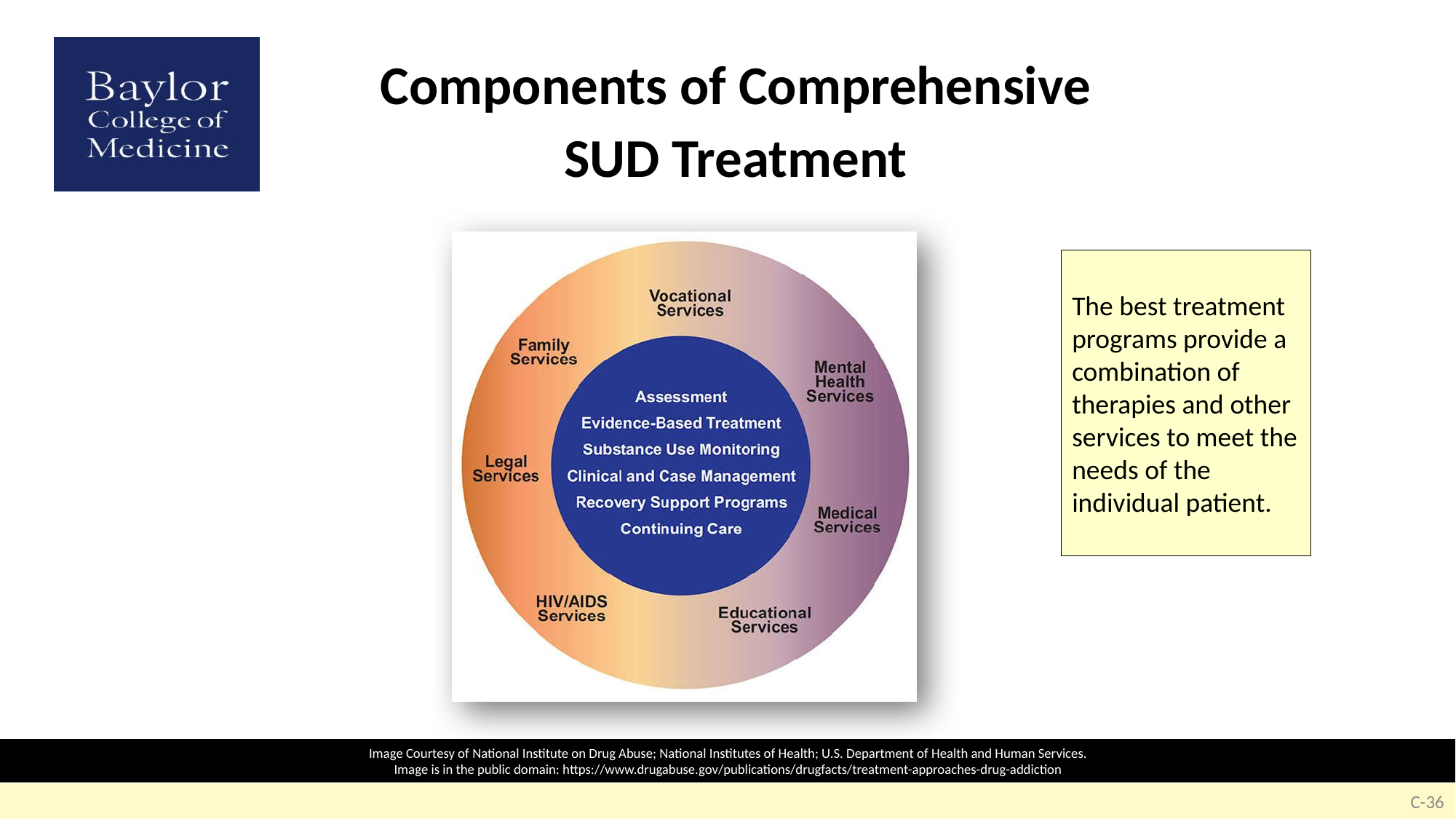

Components of Comprehensive
SUD Treatment
The best treatment programs provide a combination of therapies and other services to meet the needs of the individual patient.
Image Courtesy of National Institute on Drug Abuse; National Institutes of Health; U.S. Department of Health and Human Services.
Image is in the public domain: https://www.drugabuse.gov/publications/drugfacts/treatment-approaches-drug-addiction
C-36

## Slide 37
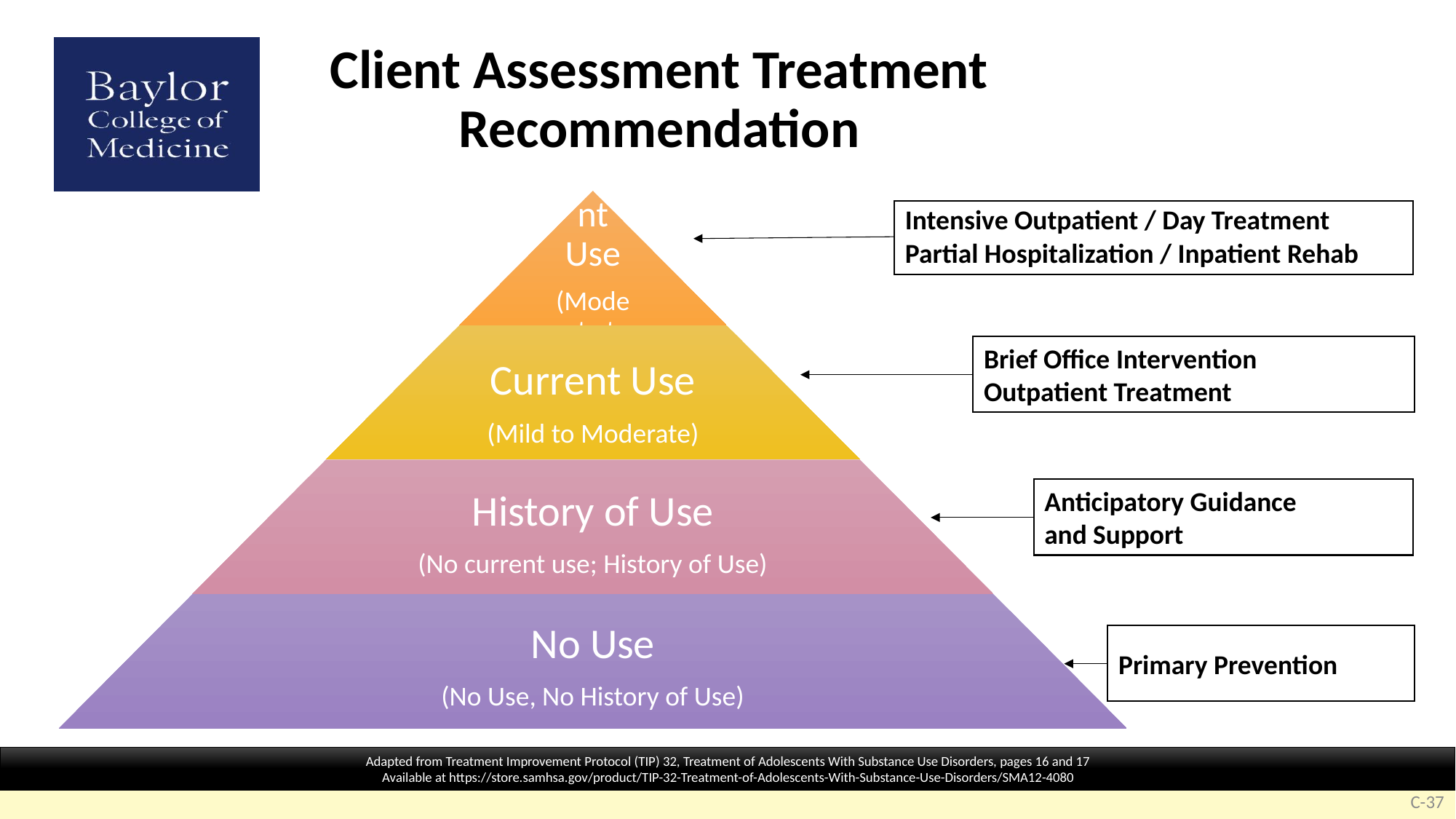

Client Assessment Treatment Recommendation
Intensive Outpatient / Day Treatment
Partial Hospitalization / Inpatient Rehab
Brief Office Intervention
Outpatient Treatment
Anticipatory Guidance
and Support
Primary Prevention
Adapted from Treatment Improvement Protocol (TIP) 32, Treatment of Adolescents With Substance Use Disorders, pages 16 and 17
Available at https://store.samhsa.gov/product/TIP-32-Treatment-of-Adolescents-With-Substance-Use-Disorders/SMA12-4080
C-37

## Slide 38
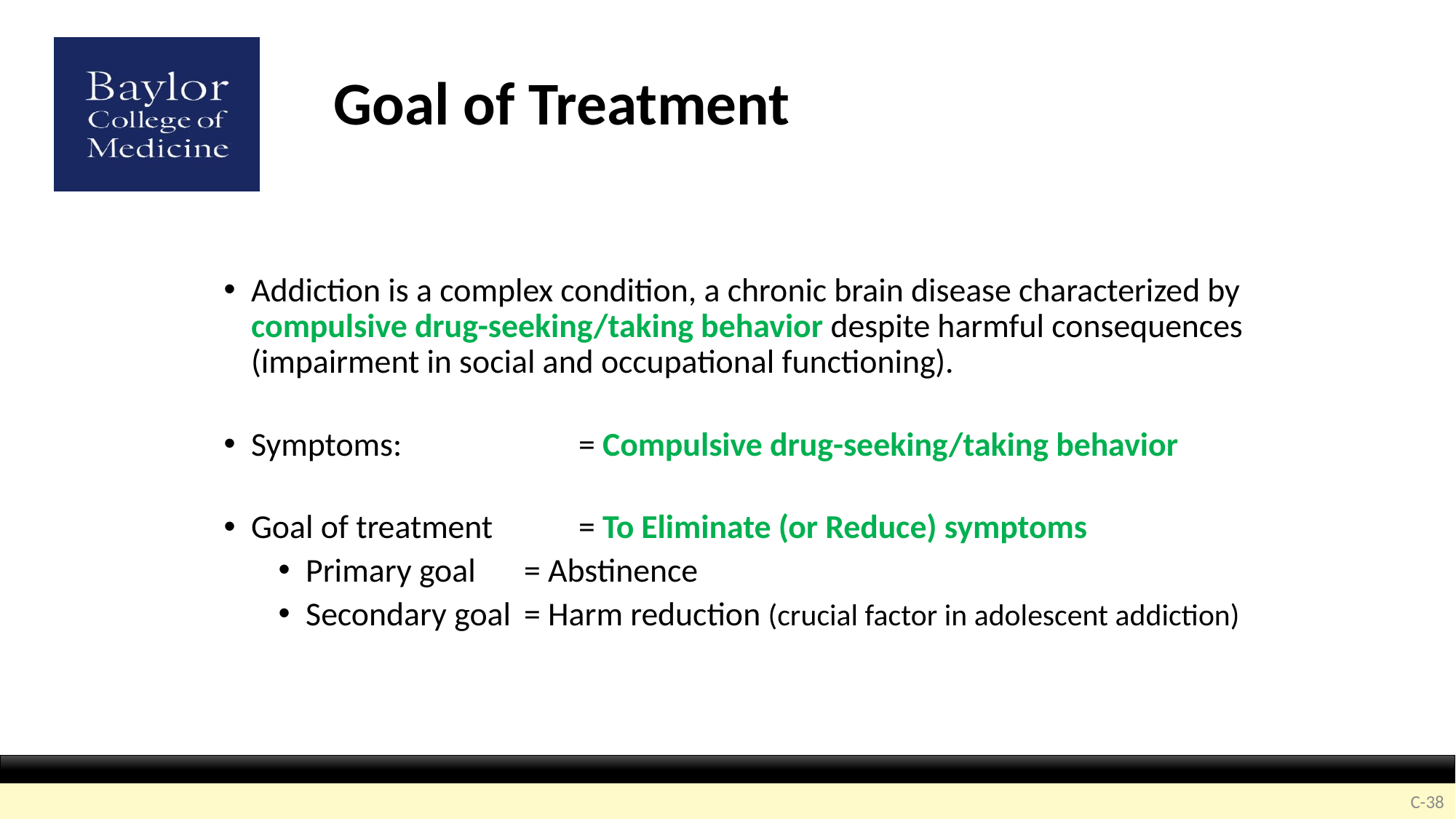

Goal of Treatment
Addiction is a complex condition, a chronic brain disease characterized by compulsive drug-seeking/taking behavior despite harmful consequences (impairment in social and occupational functioning).
Symptoms:		= Compulsive drug-seeking/taking behavior
Goal of treatment 	= To Eliminate (or Reduce) symptoms
Primary goal 	= Abstinence
Secondary goal 	= Harm reduction (crucial factor in adolescent addiction)
C-38

## Slide 39
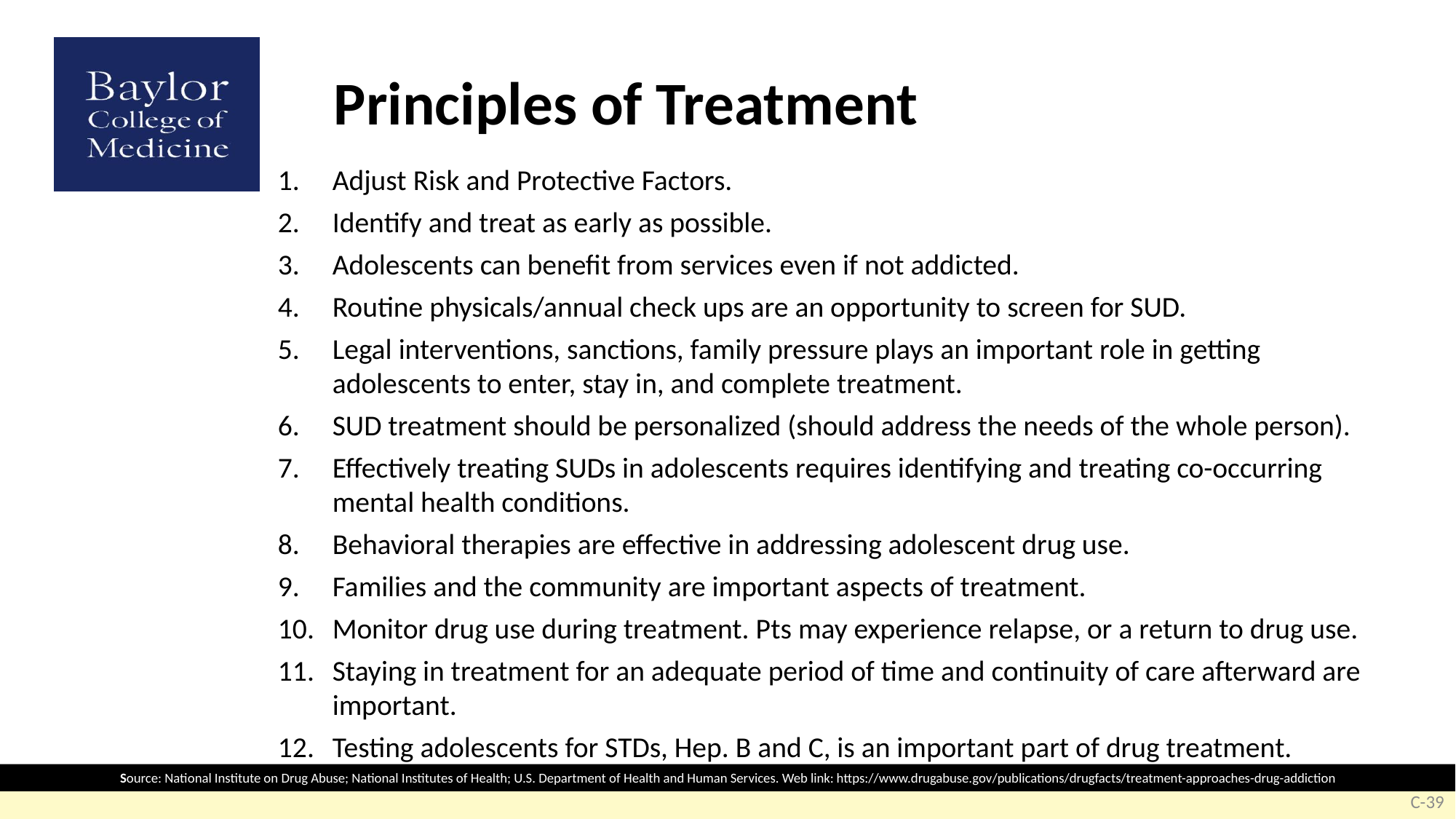

Principles of Treatment
Adjust Risk and Protective Factors.
Identify and treat as early as possible.
Adolescents can benefit from services even if not addicted.
Routine physicals/annual check ups are an opportunity to screen for SUD.
Legal interventions, sanctions, family pressure plays an important role in getting adolescents to enter, stay in, and complete treatment.
SUD treatment should be personalized (should address the needs of the whole person).
Effectively treating SUDs in adolescents requires identifying and treating co-occurring mental health conditions.
Behavioral therapies are effective in addressing adolescent drug use.
Families and the community are important aspects of treatment.
Monitor drug use during treatment. Pts may experience relapse, or a return to drug use.
Staying in treatment for an adequate period of time and continuity of care afterward are important.
Testing adolescents for STDs, Hep. B and C, is an important part of drug treatment.
Source: National Institute on Drug Abuse; National Institutes of Health; U.S. Department of Health and Human Services. Web link: https://www.drugabuse.gov/publications/drugfacts/treatment-approaches-drug-addiction
C-39

## Slide 40
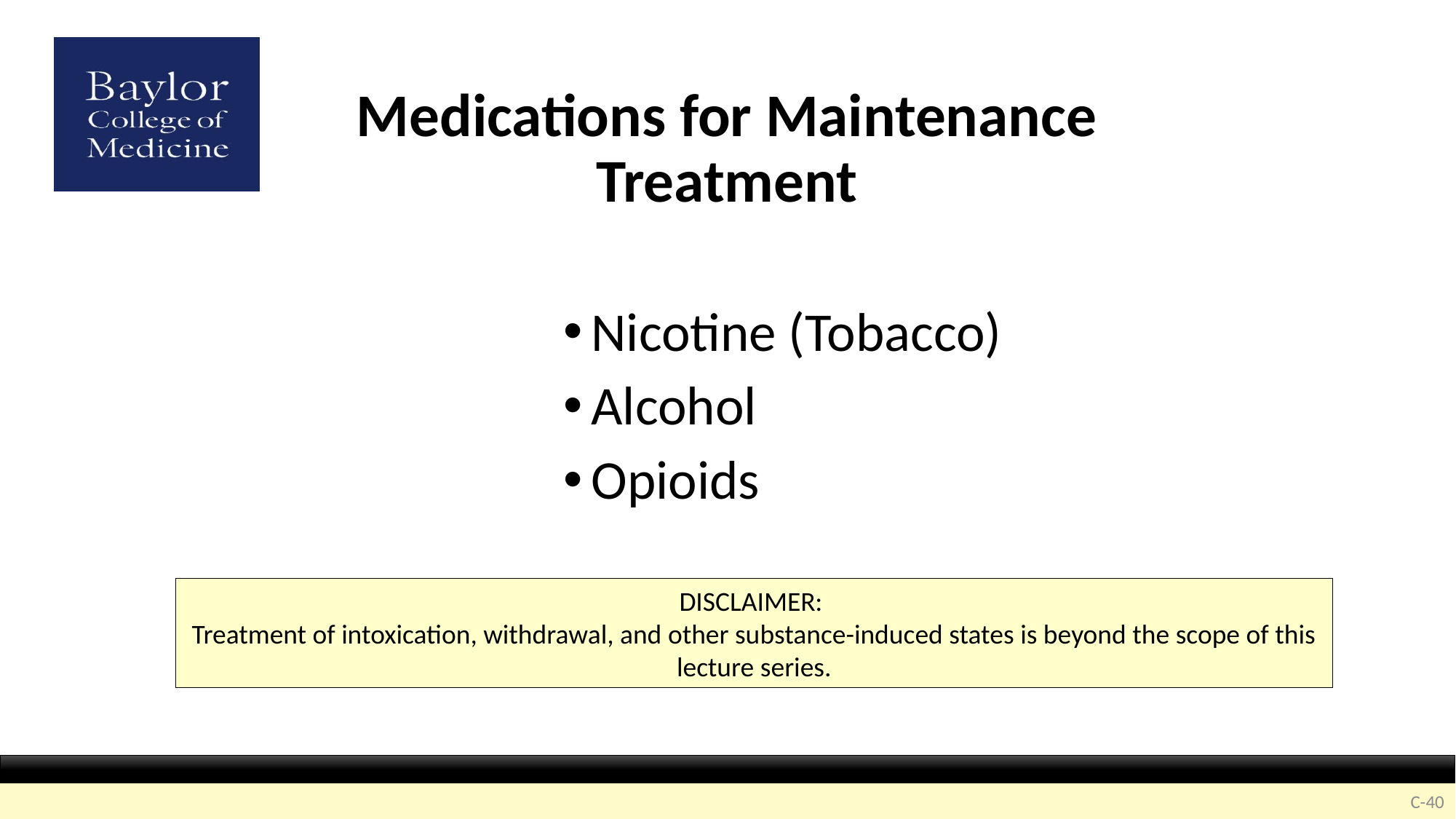

Medications for Maintenance Treatment
Nicotine (Tobacco)
Alcohol
Opioids
DISCLAIMER:
Treatment of intoxication, withdrawal, and other substance-induced states is beyond the scope of this lecture series.
C-40

## Slide 41
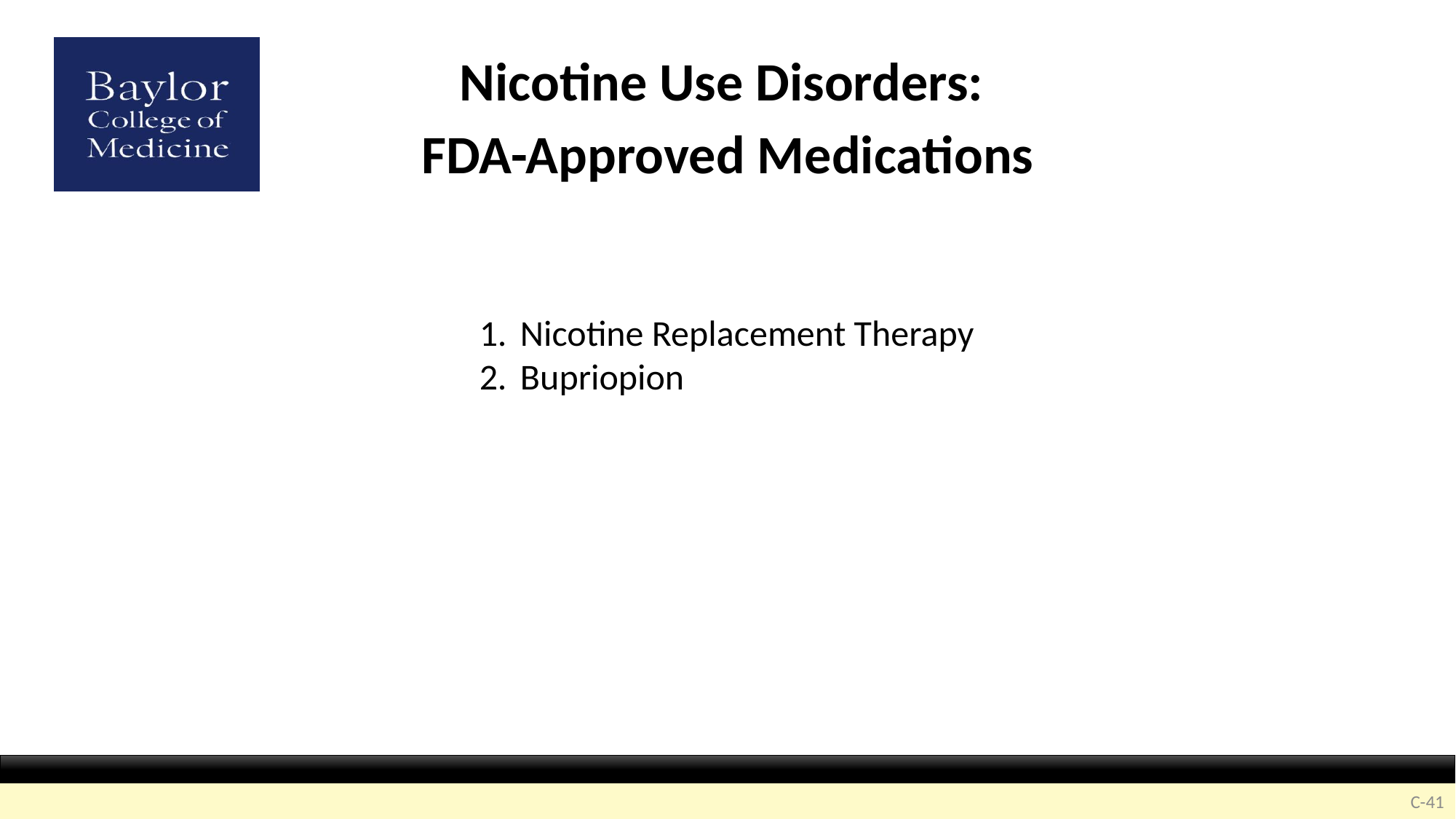

Nicotine Use Disorders:
FDA-Approved Medications
Nicotine Replacement Therapy
Bupriopion
C-41

## Slide 42
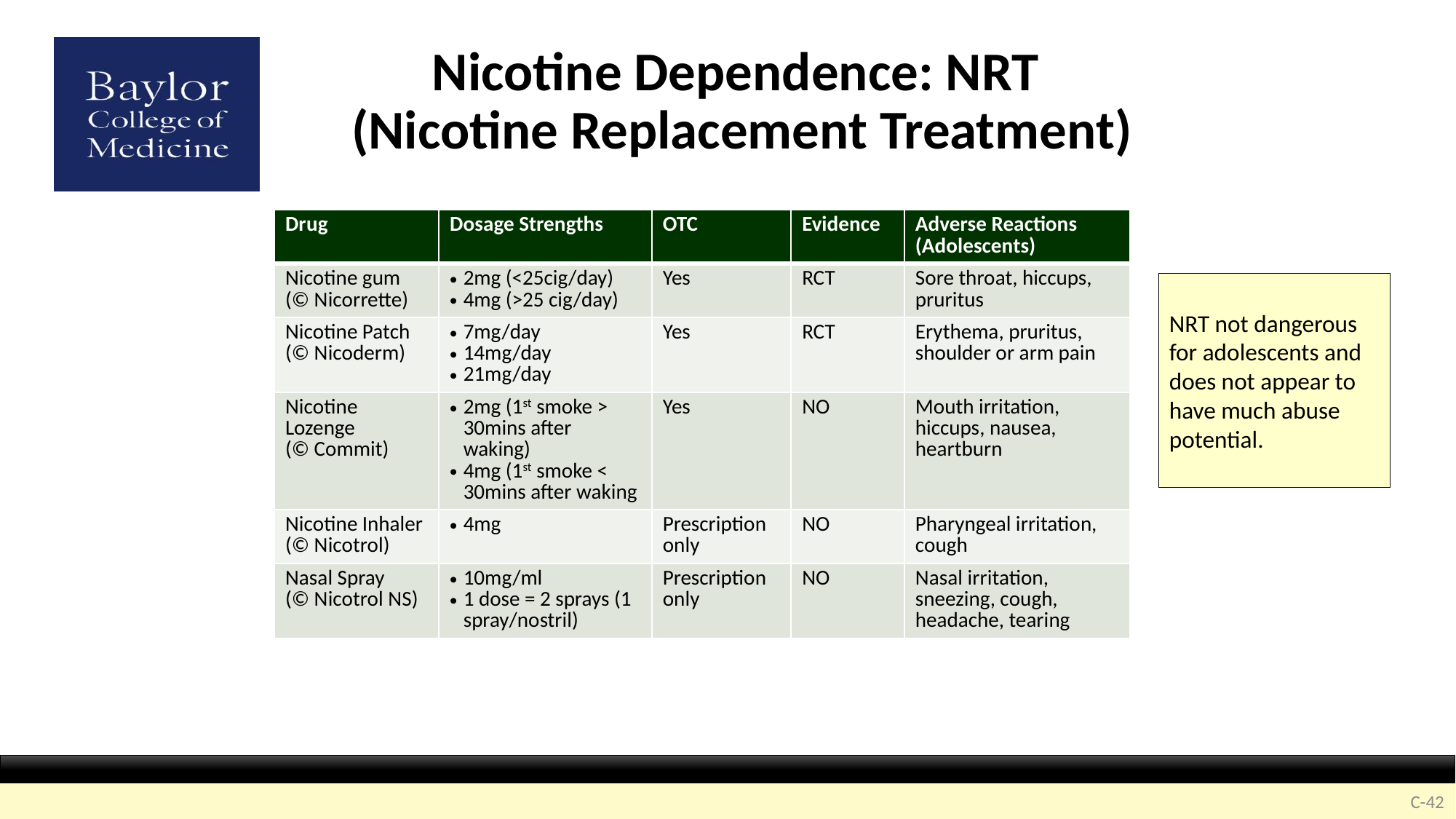

Nicotine Dependence: NRT
(Nicotine Replacement Treatment)
| Drug | Dosage Strengths | OTC | Evidence | Adverse Reactions (Adolescents) |
| --- | --- | --- | --- | --- |
| Nicotine gum (© Nicorrette) | 2mg (<25cig/day) 4mg (>25 cig/day) | Yes | RCT | Sore throat, hiccups, pruritus |
| Nicotine Patch (© Nicoderm) | 7mg/day 14mg/day 21mg/day | Yes | RCT | Erythema, pruritus, shoulder or arm pain |
| Nicotine Lozenge (© Commit) | 2mg (1st smoke > 30mins after waking) 4mg (1st smoke < 30mins after waking | Yes | NO | Mouth irritation, hiccups, nausea, heartburn |
| Nicotine Inhaler (© Nicotrol) | 4mg | Prescription only | NO | Pharyngeal irritation, cough |
| Nasal Spray (© Nicotrol NS) | 10mg/ml 1 dose = 2 sprays (1 spray/nostril) | Prescription only | NO | Nasal irritation, sneezing, cough, headache, tearing |
NRT not dangerous for adolescents and does not appear to have much abuse potential.
C-42

## Slide 43
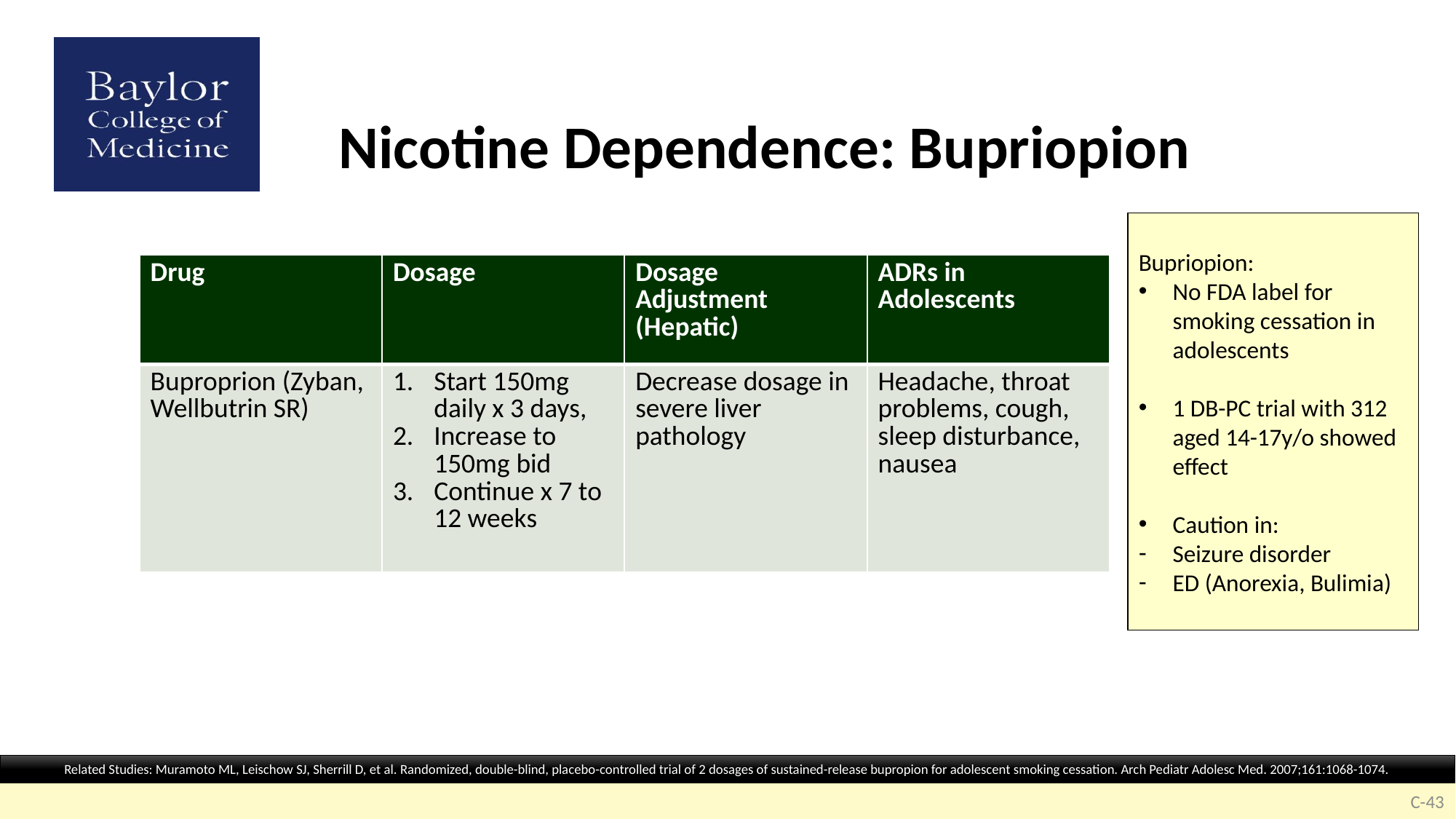

Nicotine Dependence: Bupriopion
Bupriopion:
No FDA label for smoking cessation in adolescents
1 DB-PC trial with 312 aged 14-17y/o showed effect
Caution in:
Seizure disorder
ED (Anorexia, Bulimia)
| Drug | Dosage | Dosage Adjustment (Hepatic) | ADRs in Adolescents |
| --- | --- | --- | --- |
| Buproprion (Zyban, Wellbutrin SR) | Start 150mg daily x 3 days, Increase to 150mg bid Continue x 7 to 12 weeks | Decrease dosage in severe liver pathology | Headache, throat problems, cough, sleep disturbance, nausea |
Related Studies: Muramoto ML, Leischow SJ, Sherrill D, et al. Randomized, double-blind, placebo-controlled trial of 2 dosages of sustained-release bupropion for adolescent smoking cessation. Arch Pediatr Adolesc Med. 2007;161:1068-1074.
C-43

## Slide 44
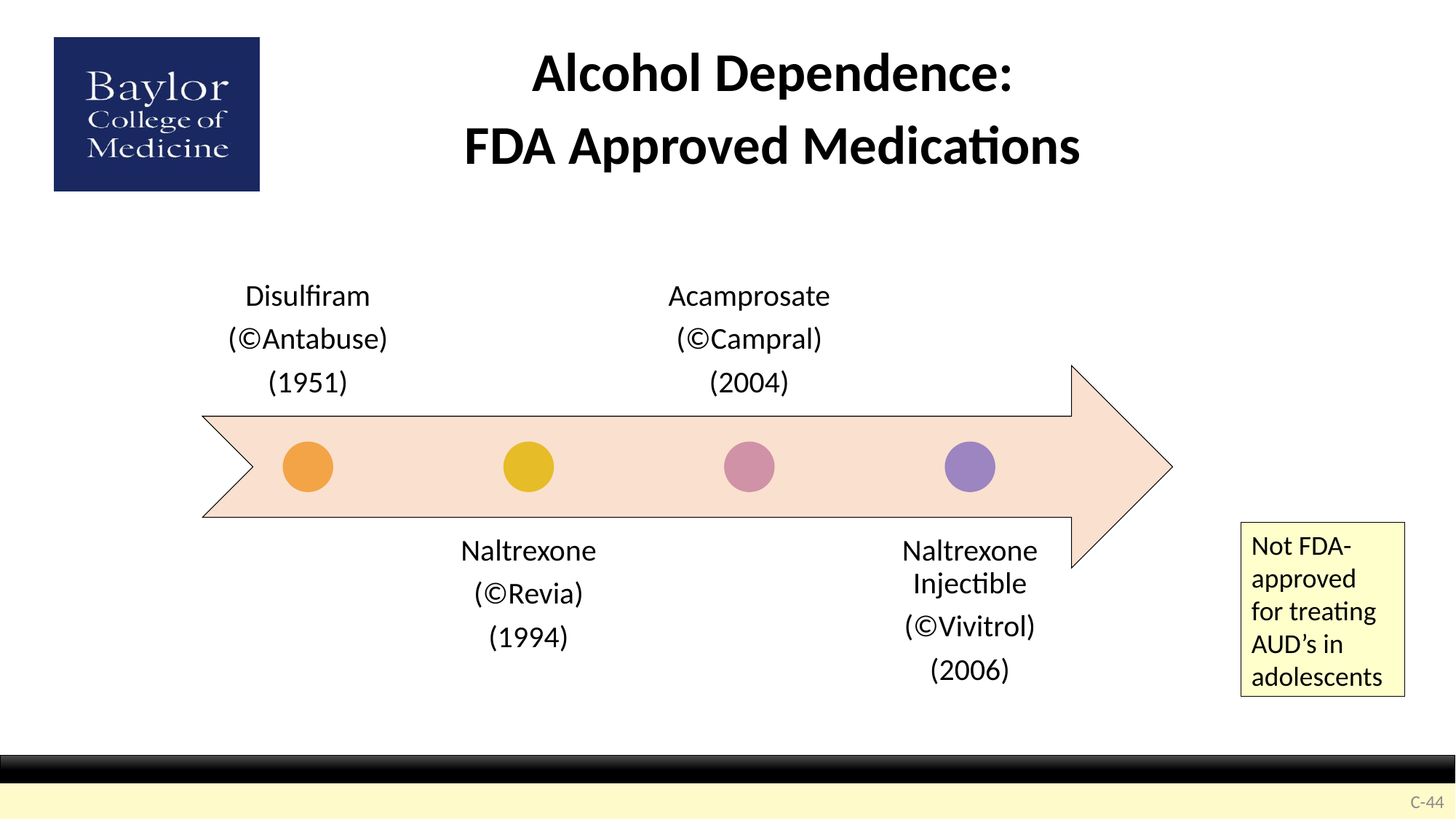

Alcohol Dependence:
FDA Approved Medications
Not FDA-approved for treating AUD’s in adolescents
C-44

## Slide 45
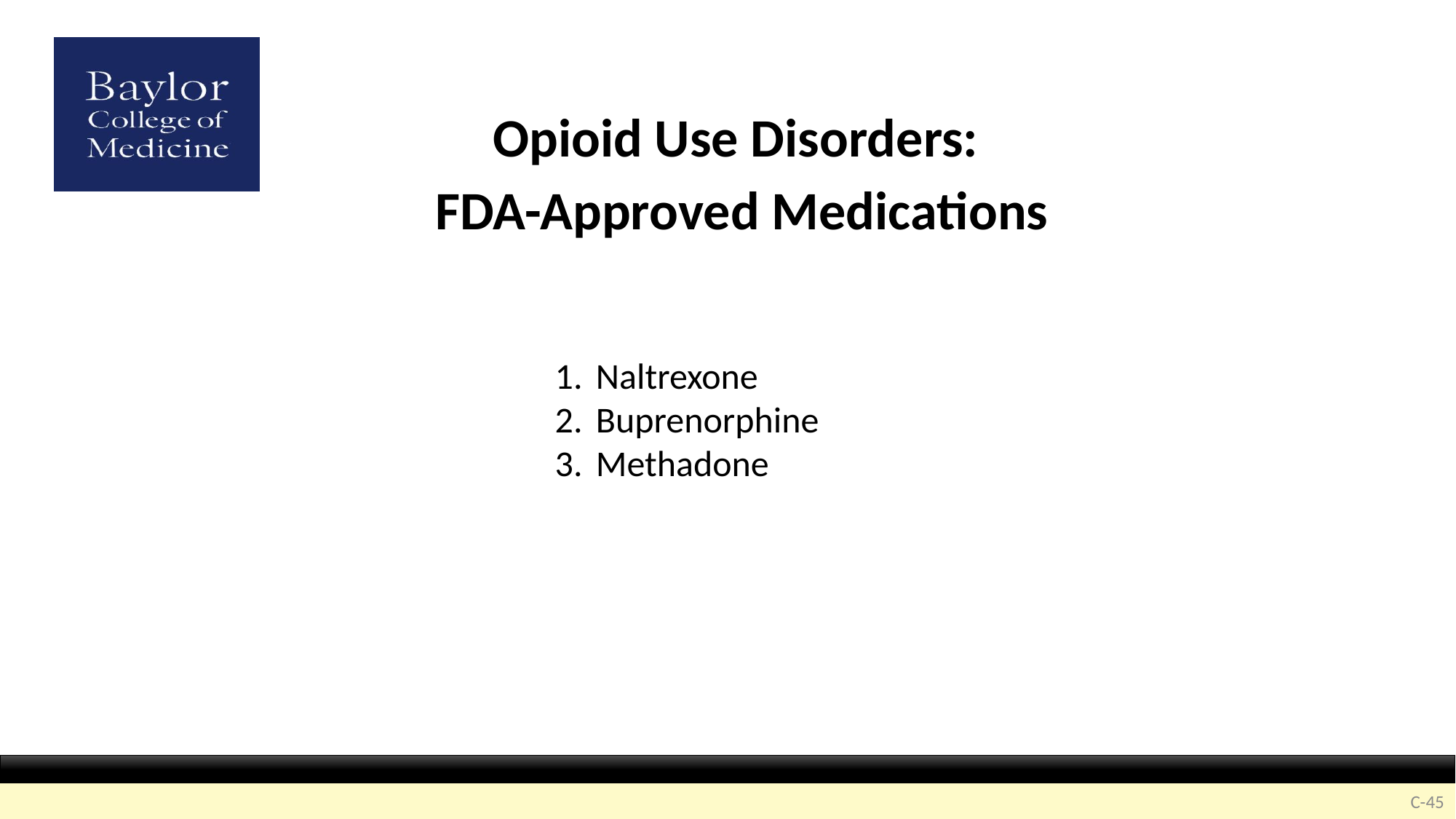

Opioid Use Disorders:
FDA-Approved Medications
Naltrexone
Buprenorphine
Methadone
C-45

## Slide 46
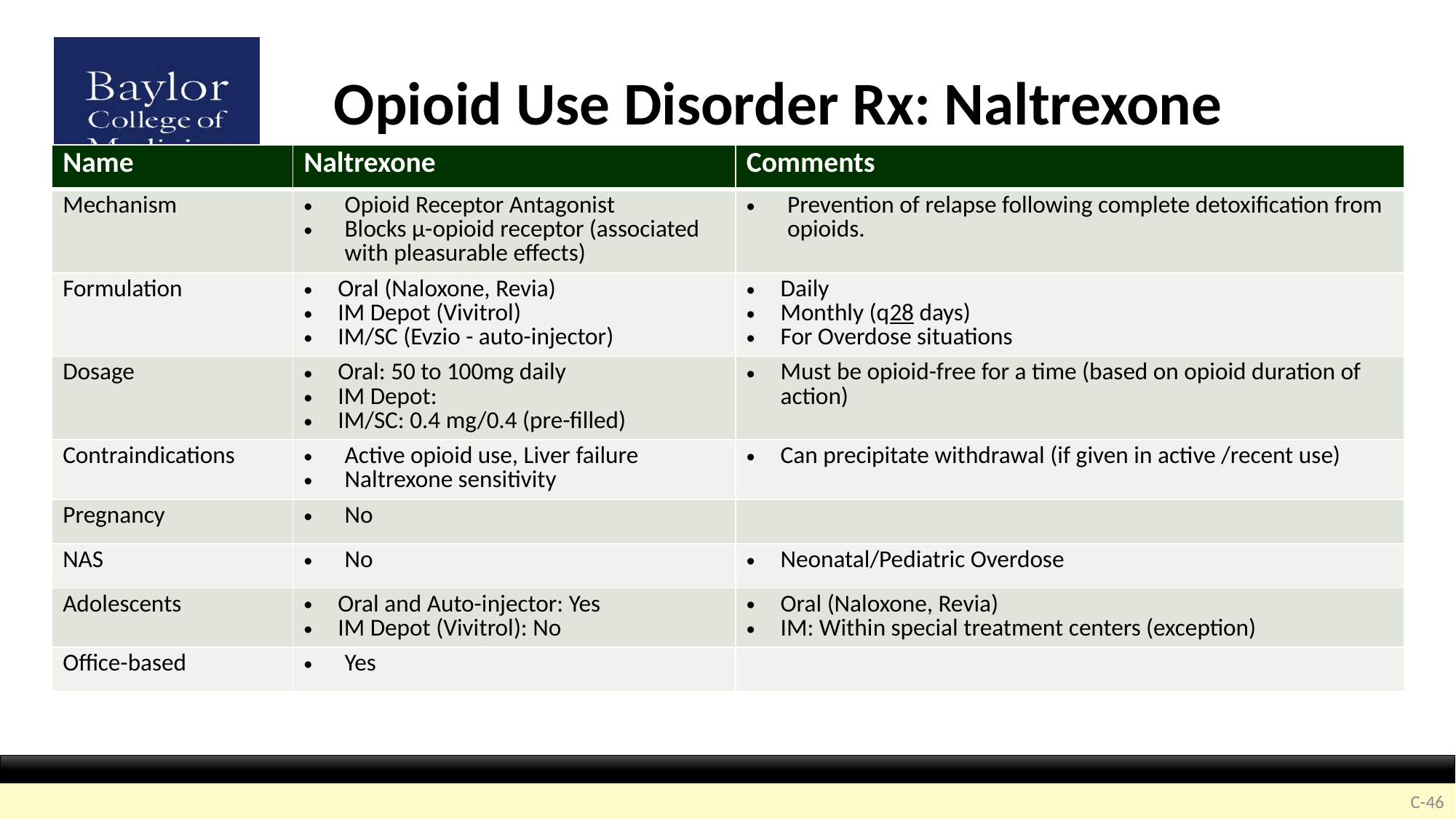

Opioid Use Disorder Rx: Naltrexone
| Name | Naltrexone | Comments |
| --- | --- | --- |
| Mechanism | Opioid Receptor Antagonist Blocks µ-opioid receptor (associated with pleasurable effects) | Prevention of relapse following complete detoxification from opioids. |
| Formulation | Oral (Naloxone, Revia) IM Depot (Vivitrol) IM/SC (Evzio - auto-injector) | Daily Monthly (q28 days) For Overdose situations |
| Dosage | Oral: 50 to 100mg daily IM Depot: IM/SC: 0.4 mg/0.4 (pre-filled) | Must be opioid-free for a time (based on opioid duration of action) |
| Contraindications | Active opioid use, Liver failure Naltrexone sensitivity | Can precipitate withdrawal (if given in active /recent use) |
| Pregnancy | No | |
| NAS | No | Neonatal/Pediatric Overdose |
| Adolescents | Oral and Auto-injector: Yes IM Depot (Vivitrol): No | Oral (Naloxone, Revia) IM: Within special treatment centers (exception) |
| Office-based | Yes | |
C-46

## Slide 47
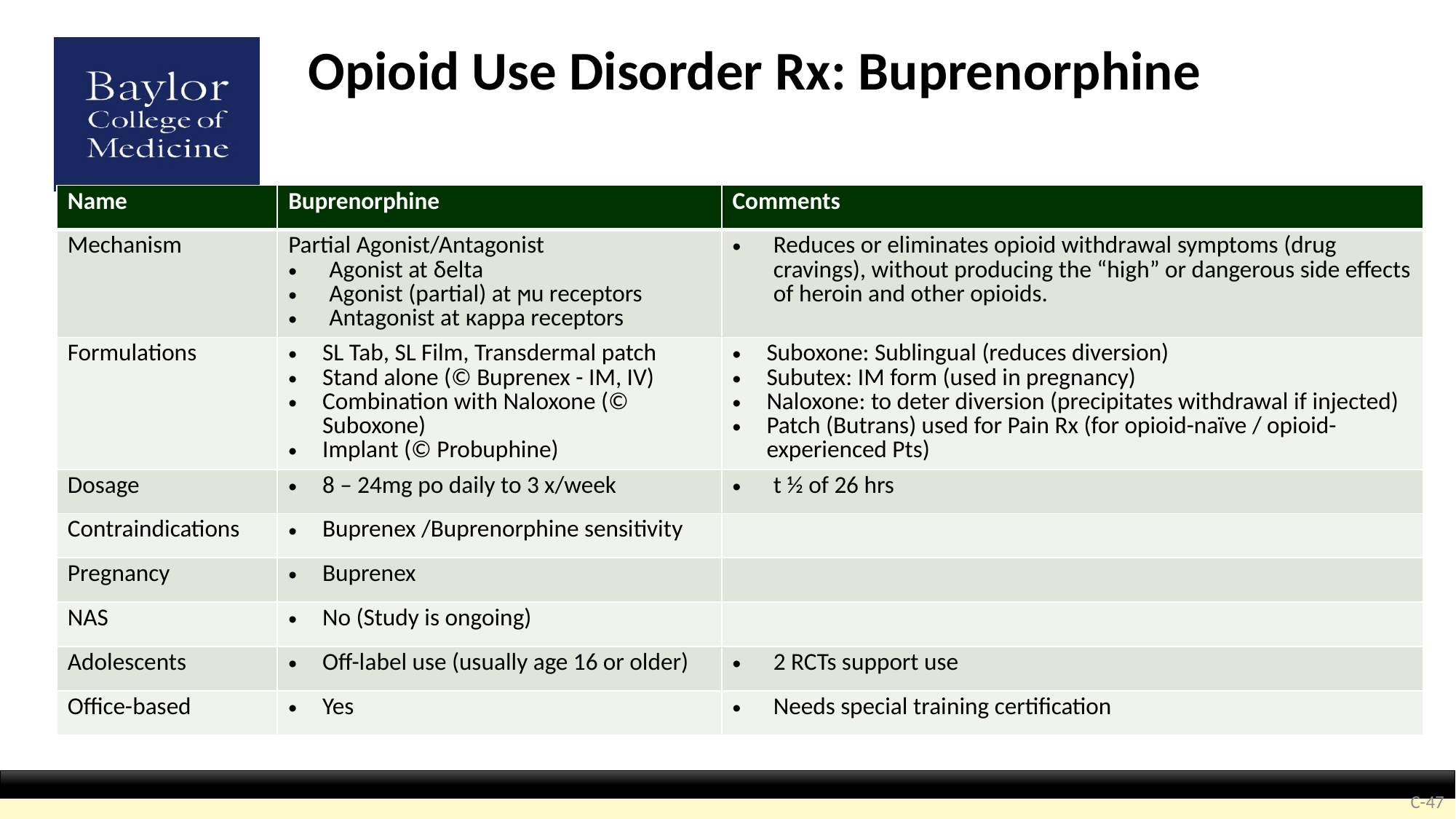

Opioid Use Disorder Rx: Buprenorphine
| Name | Buprenorphine | Comments |
| --- | --- | --- |
| Mechanism | Partial Agonist/Antagonist Agonist at δelta Agonist (partial) at ϻu receptors Antagonist at кappa receptors | Reduces or eliminates opioid withdrawal symptoms (drug cravings), without producing the “high” or dangerous side effects of heroin and other opioids. |
| Formulations | SL Tab, SL Film, Transdermal patch Stand alone (© Buprenex - IM, IV) Combination with Naloxone (© Suboxone) Implant (© Probuphine) | Suboxone: Sublingual (reduces diversion) Subutex: IM form (used in pregnancy) Naloxone: to deter diversion (precipitates withdrawal if injected) Patch (Butrans) used for Pain Rx (for opioid-naïve / opioid-experienced Pts) |
| Dosage | 8 – 24mg po daily to 3 x/week | t ½ of 26 hrs |
| Contraindications | Buprenex /Buprenorphine sensitivity | |
| Pregnancy | Buprenex | |
| NAS | No (Study is ongoing) | |
| Adolescents | Off-label use (usually age 16 or older) | 2 RCTs support use |
| Office-based | Yes | Needs special training certification |
C-47

## Slide 48
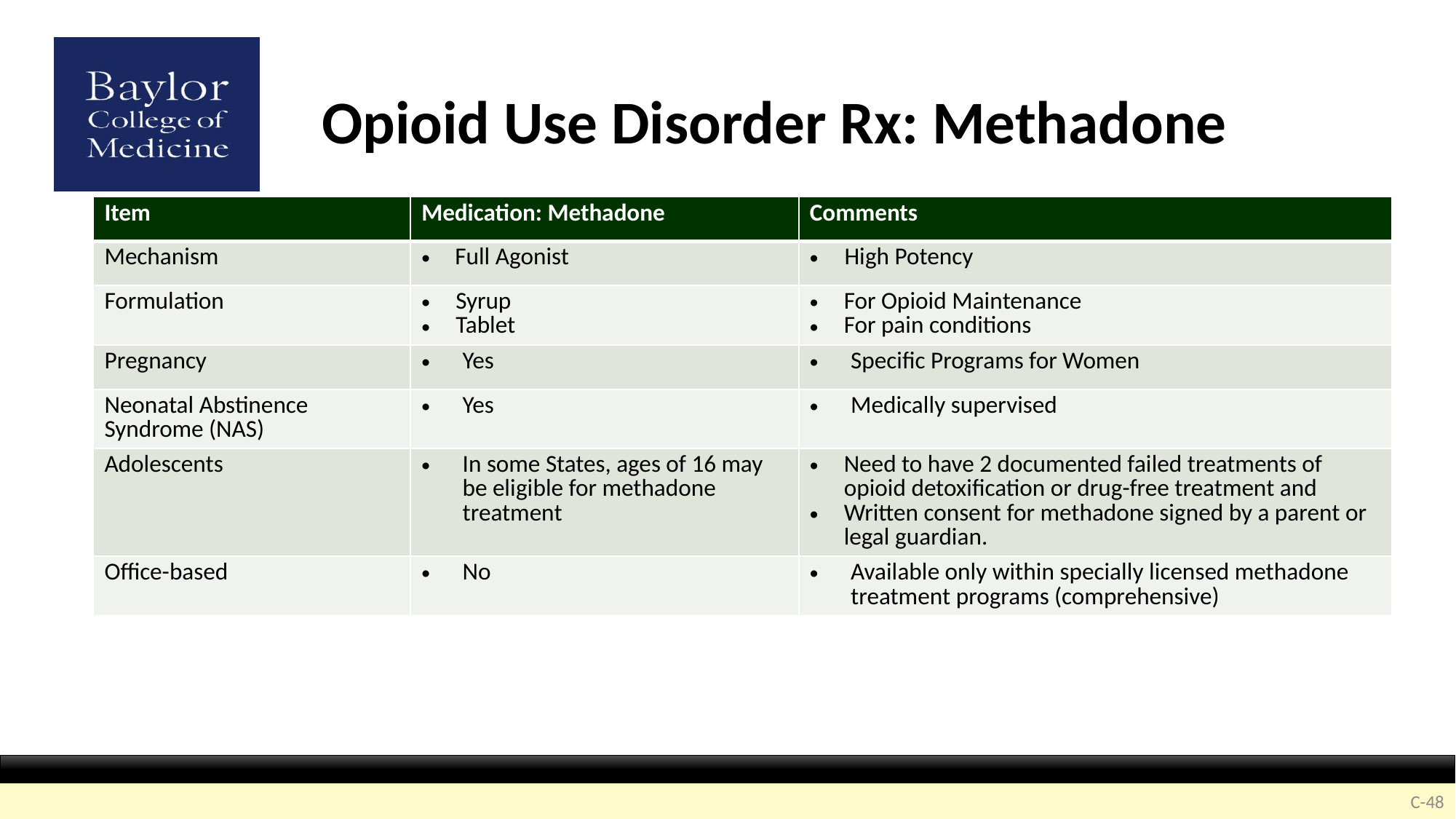

Opioid Use Disorder Rx: Methadone
| Item | Medication: Methadone | Comments |
| --- | --- | --- |
| Mechanism | Full Agonist | High Potency |
| Formulation | Syrup Tablet | For Opioid Maintenance For pain conditions |
| Pregnancy | Yes | Specific Programs for Women |
| Neonatal Abstinence Syndrome (NAS) | Yes | Medically supervised |
| Adolescents | In some States, ages of 16 may be eligible for methadone treatment | Need to have 2 documented failed treatments of opioid detoxification or drug-free treatment and Written consent for methadone signed by a parent or legal guardian. |
| Office-based | No | Available only within specially licensed methadone treatment programs (comprehensive) |
C-48

## Slide 49
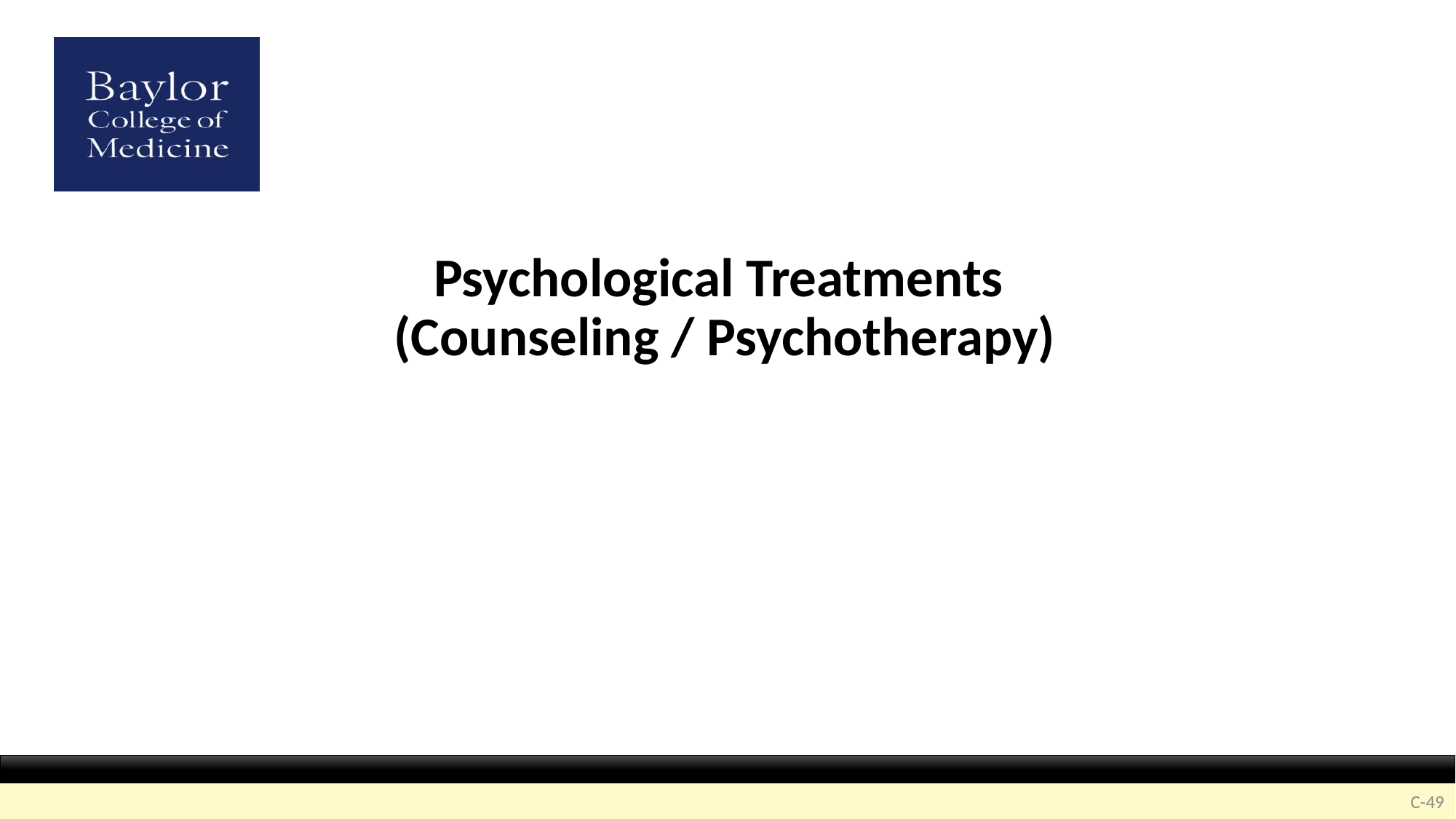

Psychological Treatments
(Counseling / Psychotherapy)
C-49

## Slide 50
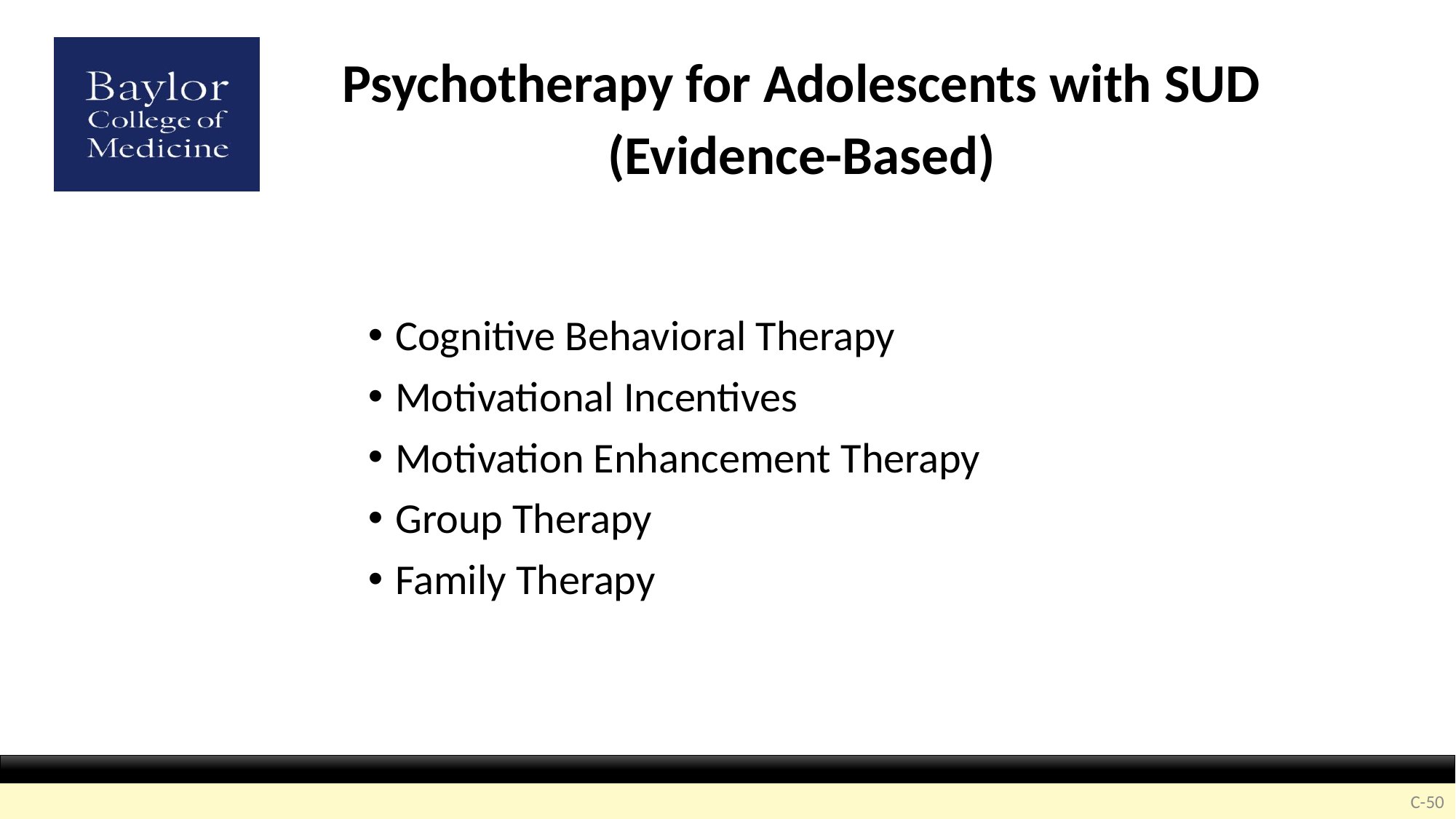

Psychotherapy for Adolescents with SUD
(Evidence-Based)
Cognitive Behavioral Therapy
Motivational Incentives
Motivation Enhancement Therapy
Group Therapy
Family Therapy
C-50

## Slide 51
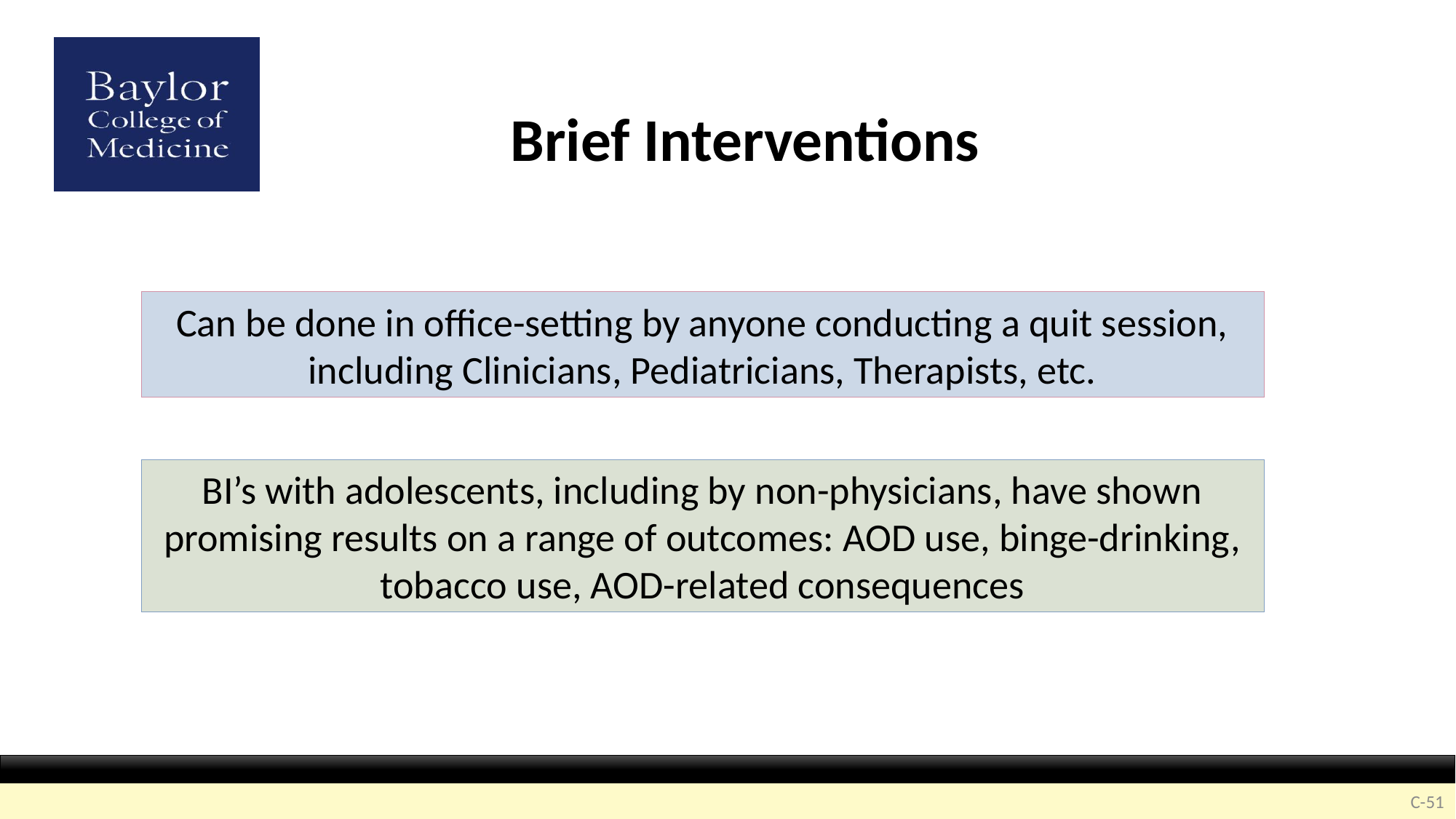

Brief Interventions
Can be done in office-setting by anyone conducting a quit session, including Clinicians, Pediatricians, Therapists, etc.
BI’s with adolescents, including by non-physicians, have shown promising results on a range of outcomes: AOD use, binge-drinking, tobacco use, AOD-related consequences
C-51

## Slide 52
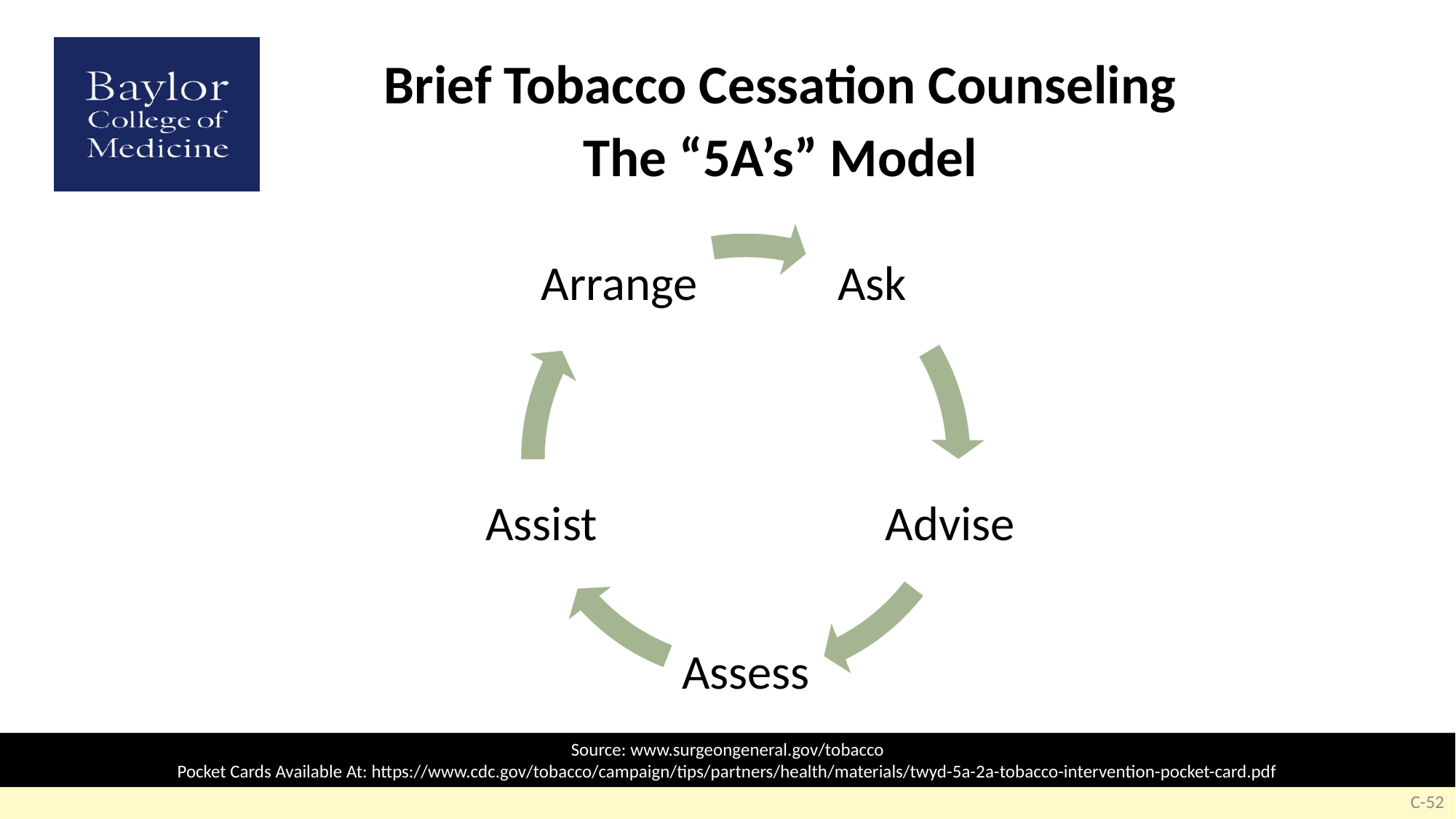

Brief Tobacco Cessation Counseling
The “5A’s” Model
Source: www.surgeongeneral.gov/tobacco
Pocket Cards Available At: https://www.cdc.gov/tobacco/campaign/tips/partners/health/materials/twyd-5a-2a-tobacco-intervention-pocket-card.pdf
C-52

## Slide 53
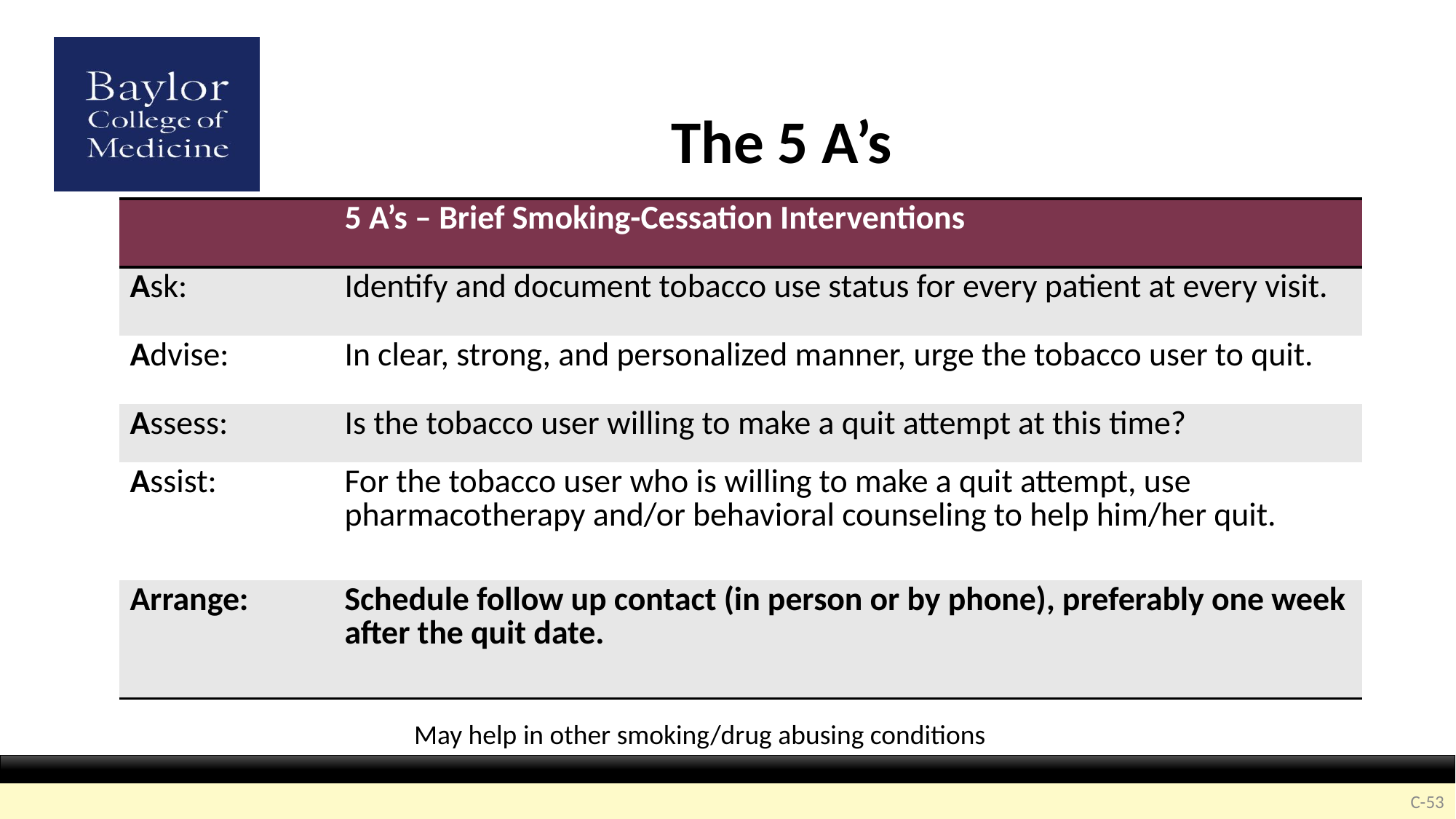

The 5 A’s
| | 5 A’s – Brief Smoking-Cessation Interventions |
| --- | --- |
| Ask: | Identify and document tobacco use status for every patient at every visit. |
| Advise: | In clear, strong, and personalized manner, urge the tobacco user to quit. |
| Assess: | Is the tobacco user willing to make a quit attempt at this time? |
| Assist: | For the tobacco user who is willing to make a quit attempt, use pharmacotherapy and/or behavioral counseling to help him/her quit. |
| Arrange: | Schedule follow up contact (in person or by phone), preferably one week after the quit date. |
May help in other smoking/drug abusing conditions
C-53

## Slide 54
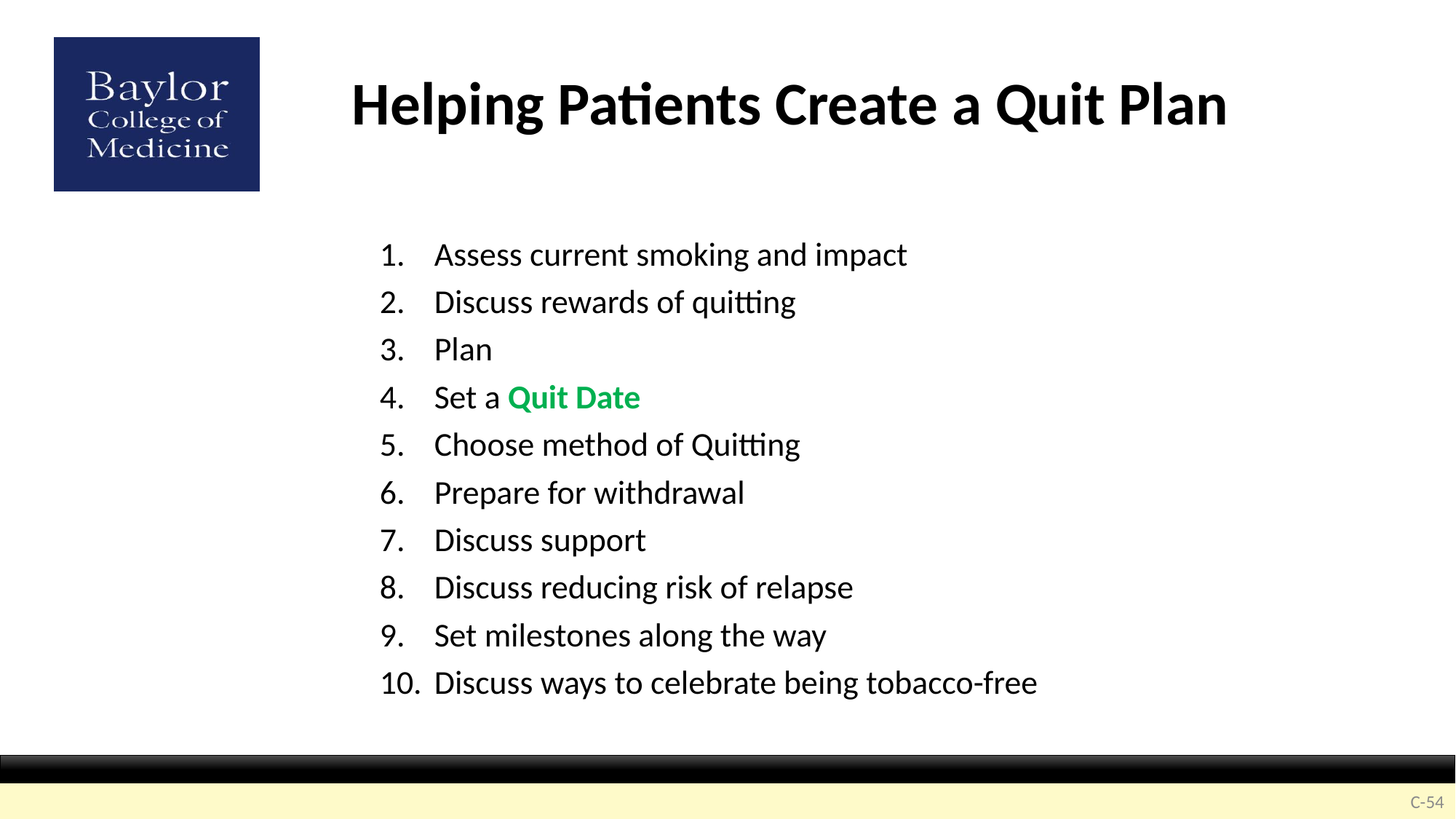

Helping Patients Create a Quit Plan
Assess current smoking and impact
Discuss rewards of quitting
Plan
Set a Quit Date
Choose method of Quitting
Prepare for withdrawal
Discuss support
Discuss reducing risk of relapse
Set milestones along the way
Discuss ways to celebrate being tobacco-free
C-54

## Slide 55
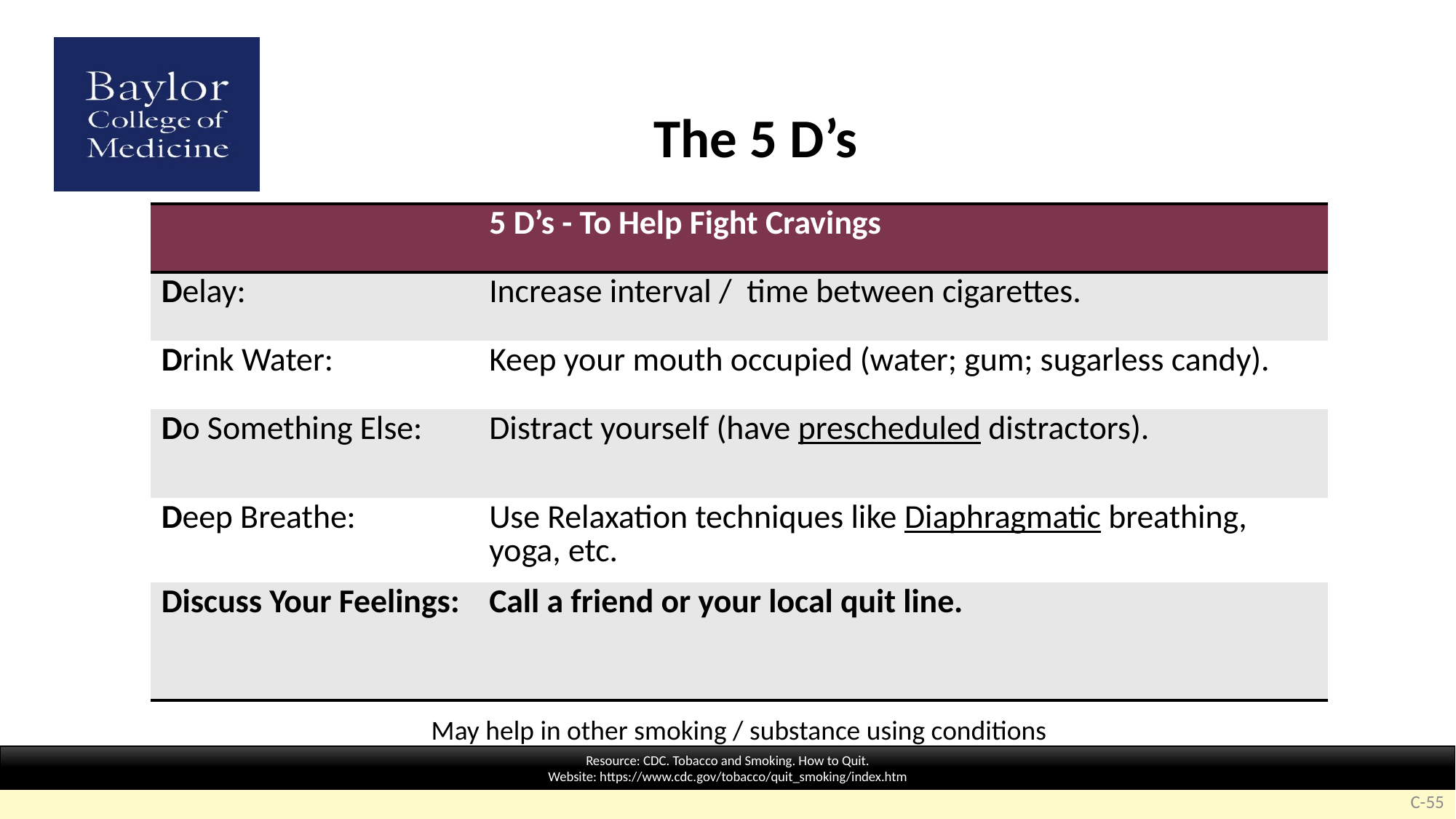

The 5 D’s
| | 5 D’s - To Help Fight Cravings |
| --- | --- |
| Delay: | Increase interval / time between cigarettes. |
| Drink Water: | Keep your mouth occupied (water; gum; sugarless candy). |
| Do Something Else: | Distract yourself (have prescheduled distractors). |
| Deep Breathe: | Use Relaxation techniques like Diaphragmatic breathing, yoga, etc. |
| Discuss Your Feelings: | Call a friend or your local quit line. |
May help in other smoking / substance using conditions
Resource: CDC. Tobacco and Smoking. How to Quit.
Website: https://www.cdc.gov/tobacco/quit_smoking/index.htm
C-55

## Slide 56
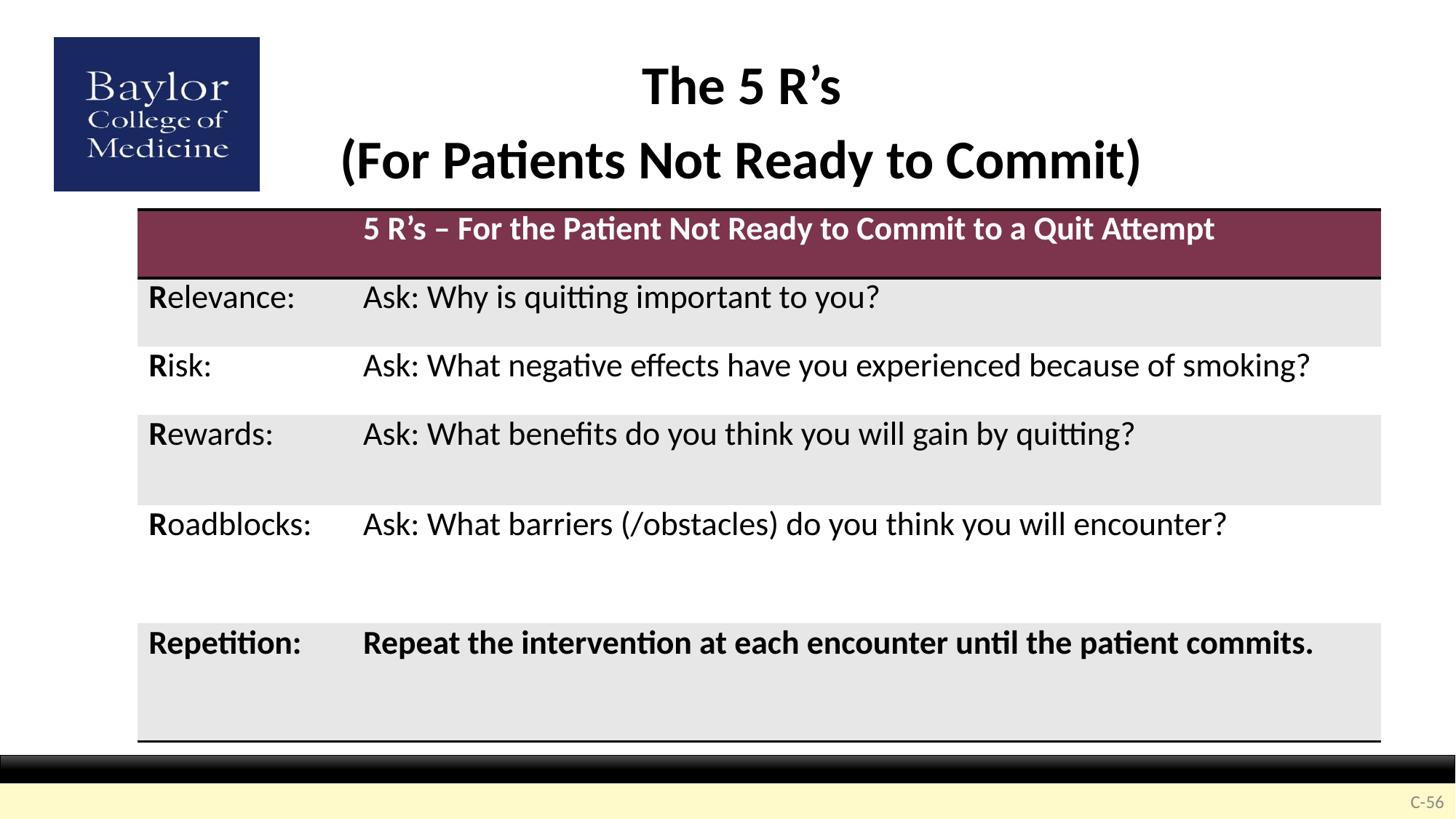

The 5 R’s
(For Patients Not Ready to Commit)
| | 5 R’s – For the Patient Not Ready to Commit to a Quit Attempt |
| --- | --- |
| Relevance: | Ask: Why is quitting important to you? |
| Risk: | Ask: What negative effects have you experienced because of smoking? |
| Rewards: | Ask: What benefits do you think you will gain by quitting? |
| Roadblocks: | Ask: What barriers (/obstacles) do you think you will encounter? |
| Repetition: | Repeat the intervention at each encounter until the patient commits. |
C-56

## Slide 57
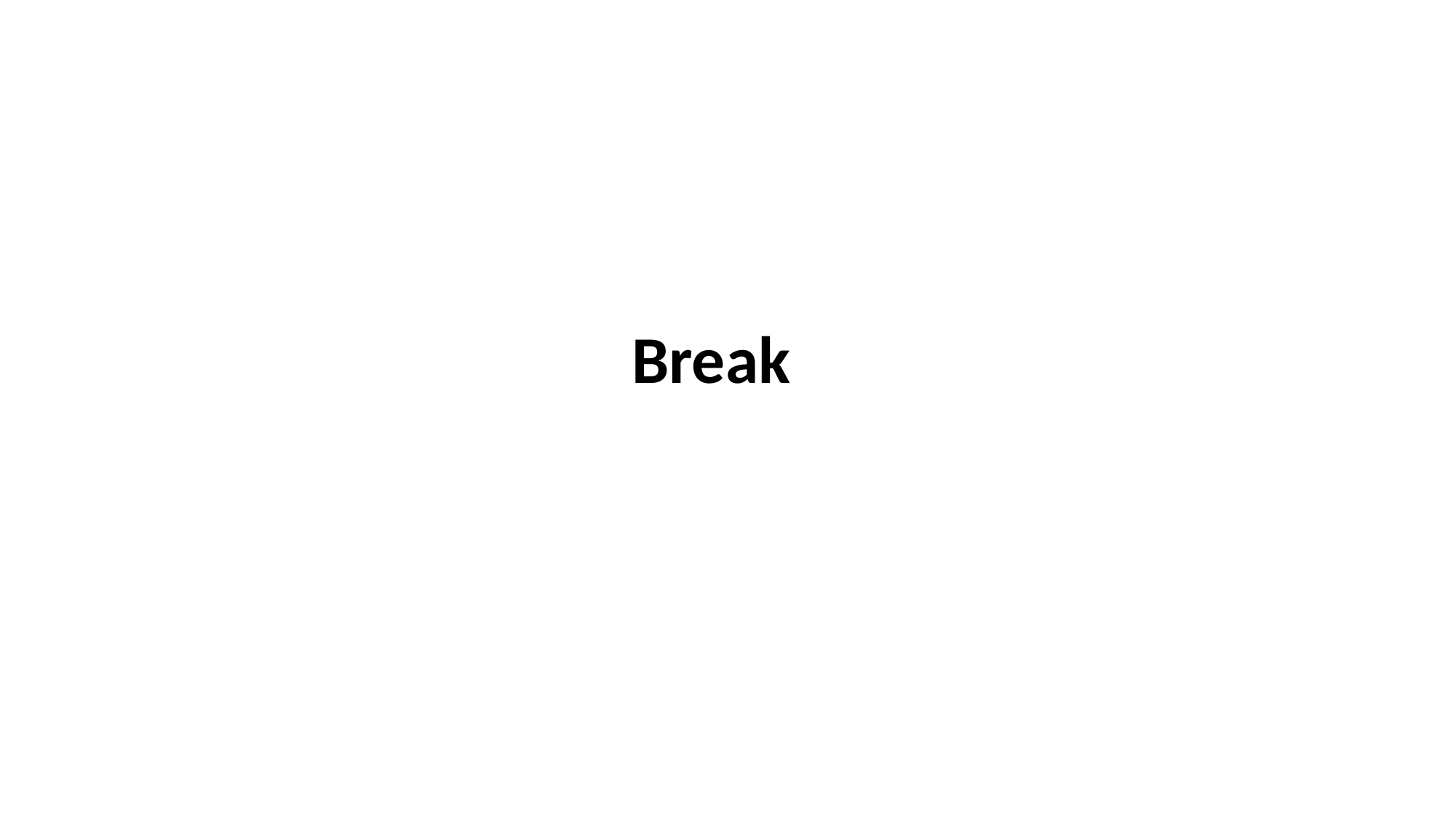

# Break

## Slide 58
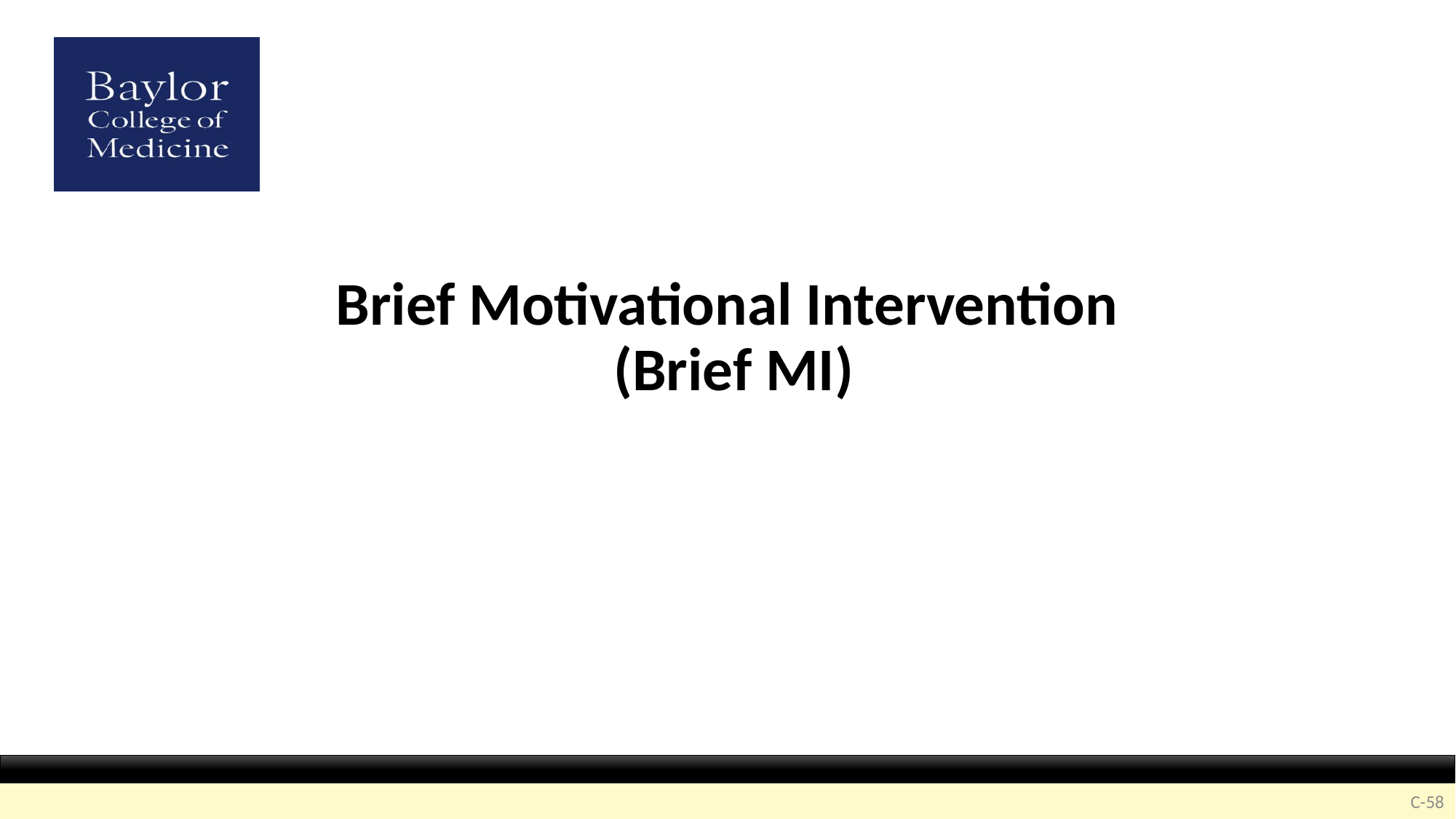

Brief Motivational Intervention
(Brief MI)
C-58

## Slide 59
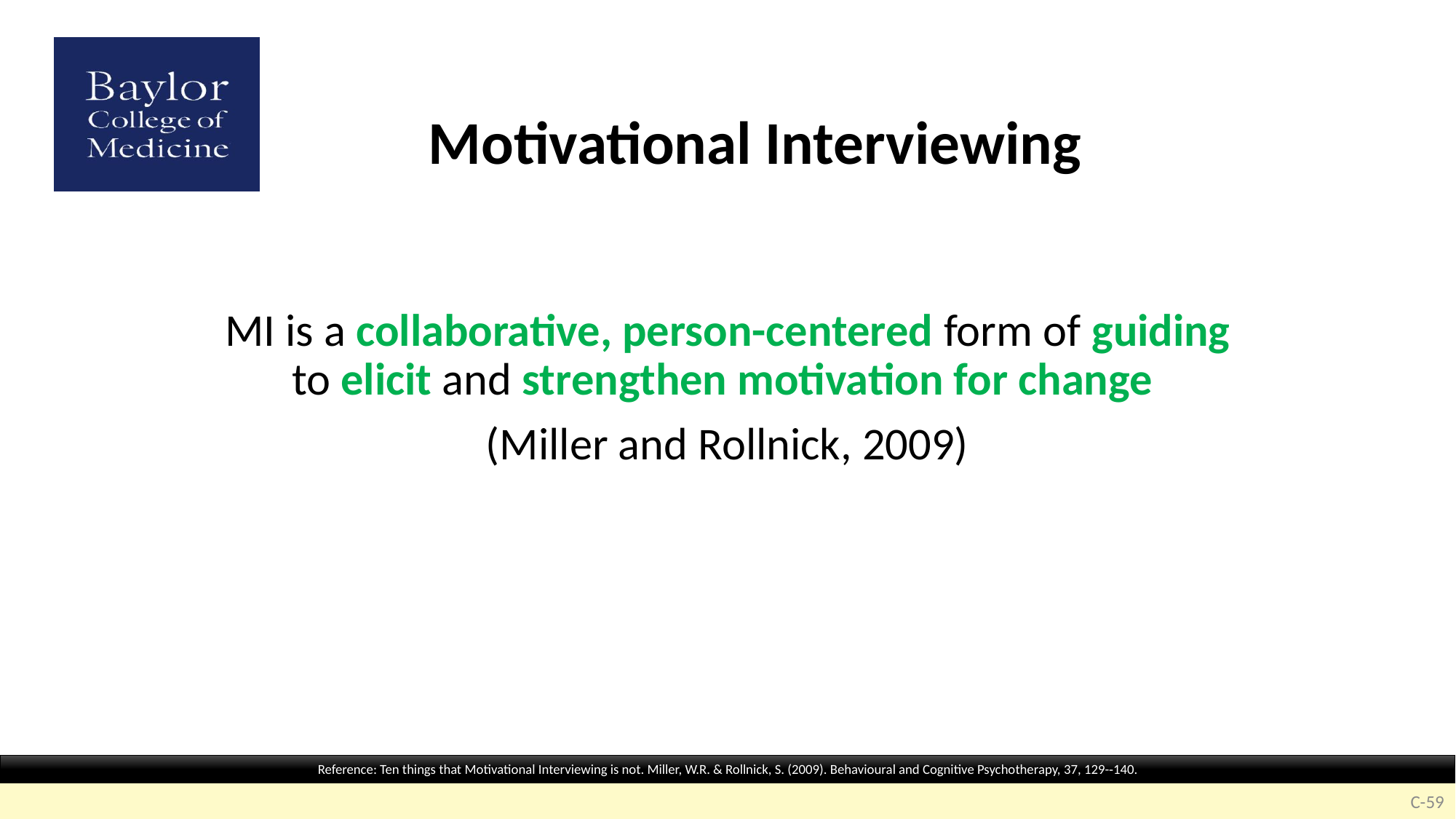

Motivational Interviewing
MI is a collaborative, person-centered form of guiding to elicit and strengthen motivation for change
(Miller and Rollnick, 2009)
Reference: Ten things that Motivational Interviewing is not. Miller, W.R. & Rollnick, S. (2009). Behavioural and Cognitive Psychotherapy, 37, 129-­‐140.
C-59

## Slide 60
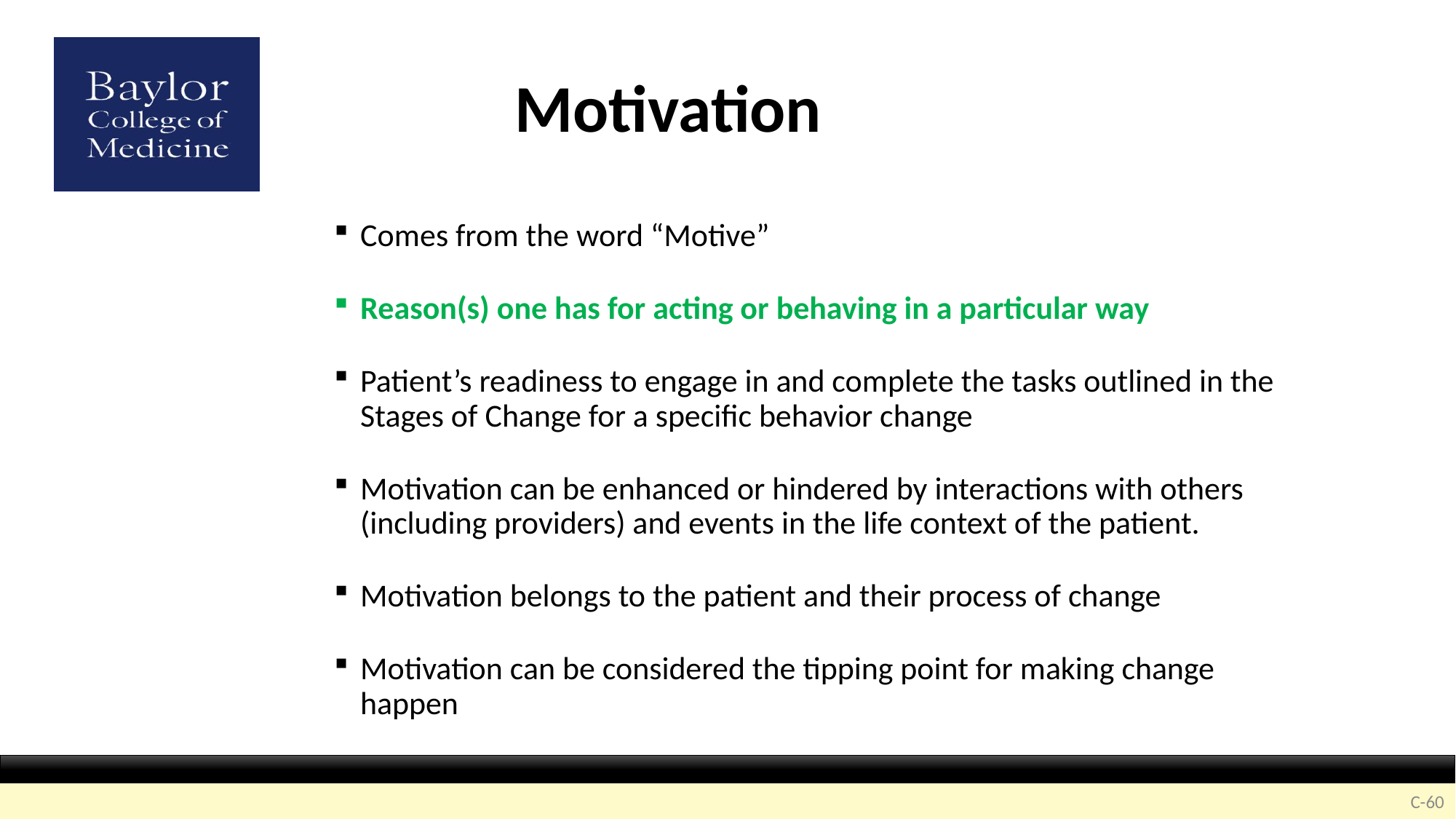

Motivation
Comes from the word “Motive”
Reason(s) one has for acting or behaving in a particular way
Patient’s readiness to engage in and complete the tasks outlined in the Stages of Change for a specific behavior change
Motivation can be enhanced or hindered by interactions with others (including providers) and events in the life context of the patient.
Motivation belongs to the patient and their process of change
Motivation can be considered the tipping point for making change happen
C-60

## Slide 61
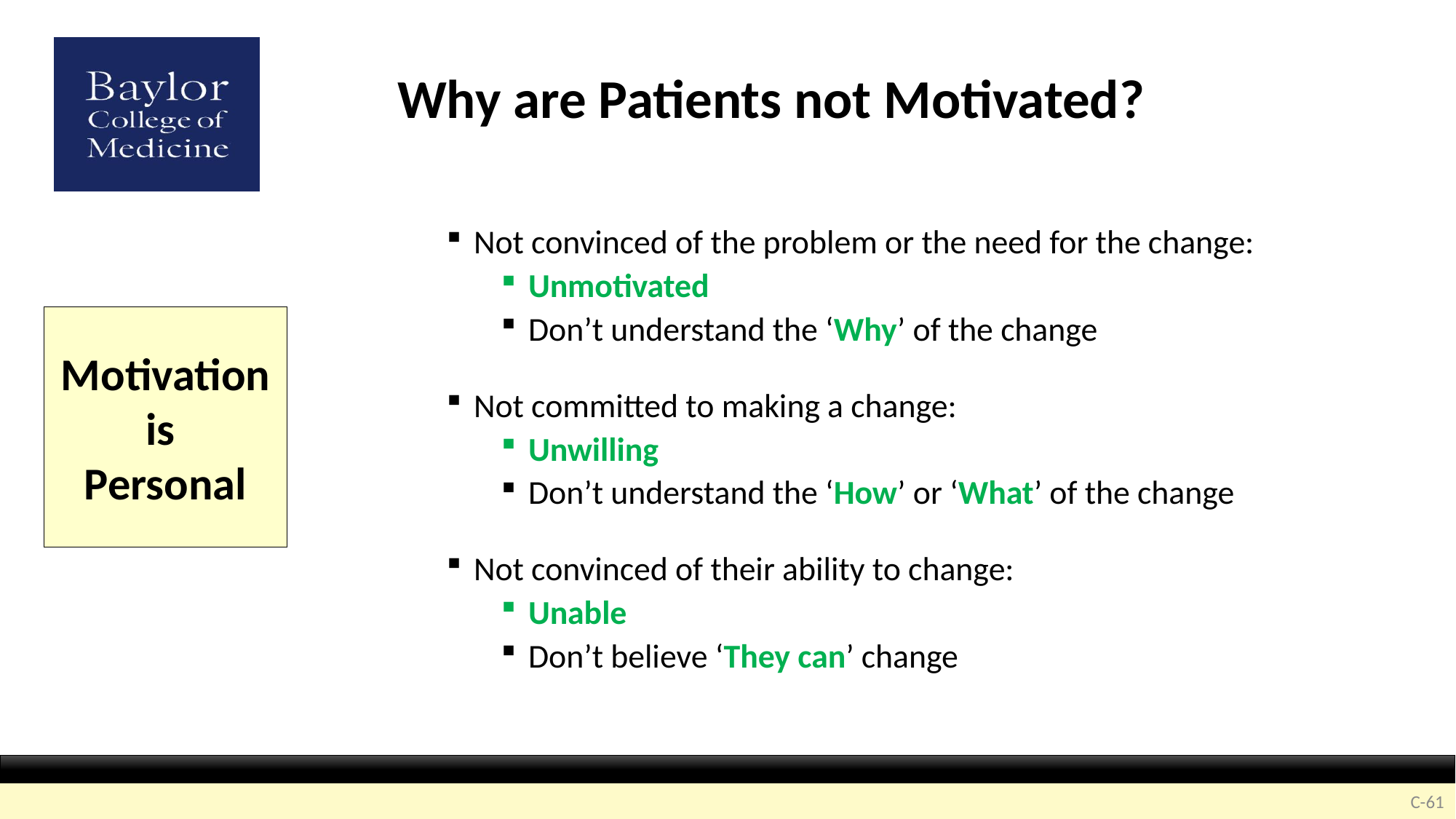

Why are Patients not Motivated?
Not convinced of the problem or the need for the change:
Unmotivated
Don’t understand the ‘Why’ of the change
Not committed to making a change:
Unwilling
Don’t understand the ‘How’ or ‘What’ of the change
Not convinced of their ability to change:
Unable
Don’t believe ‘They can’ change
Motivation is
Personal
C-61

## Slide 62
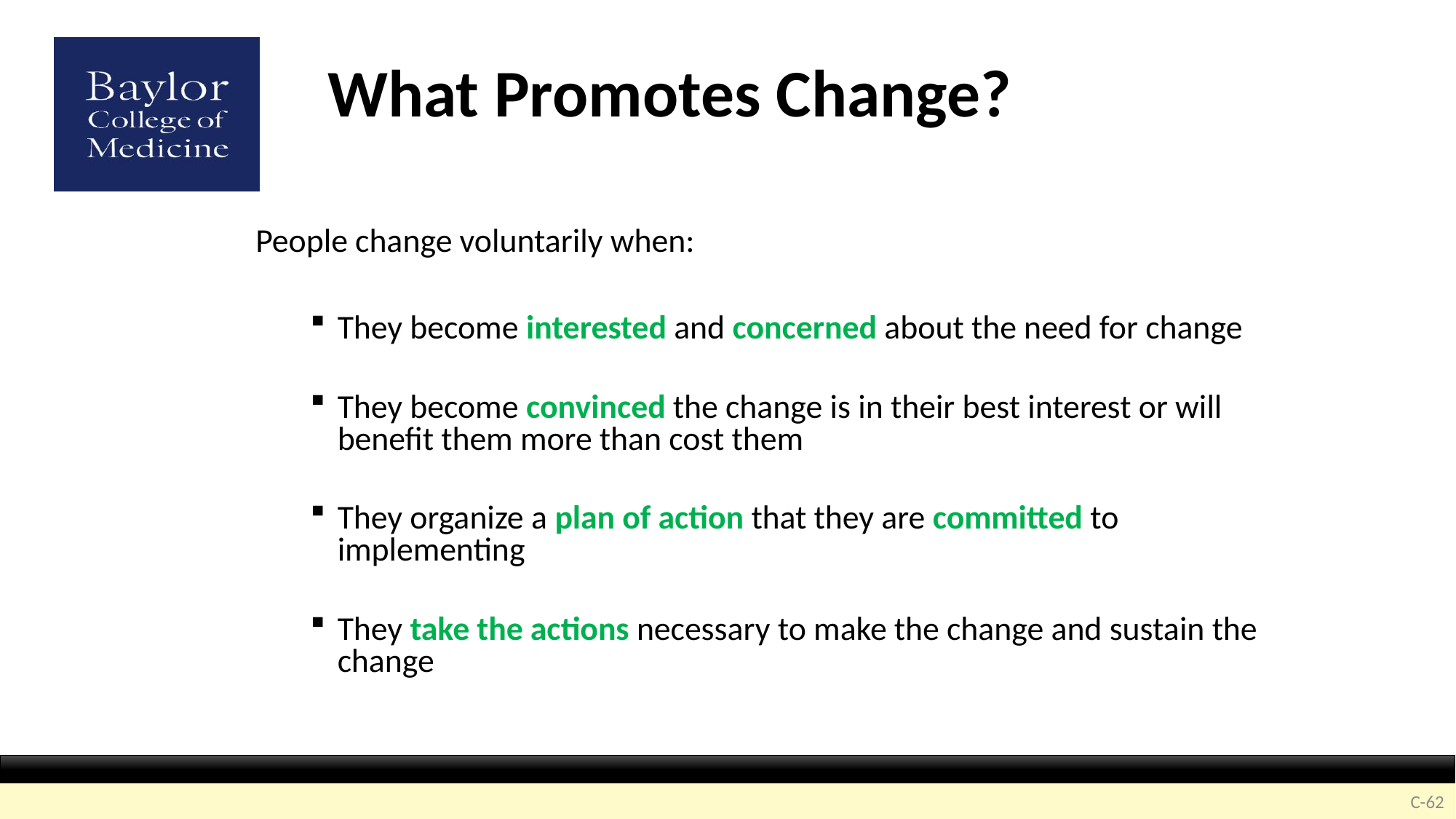

What Promotes Change?
People change voluntarily when:
They become interested and concerned about the need for change
They become convinced the change is in their best interest or will benefit them more than cost them
They organize a plan of action that they are committed to implementing
They take the actions necessary to make the change and sustain the change
C-62

## Slide 63
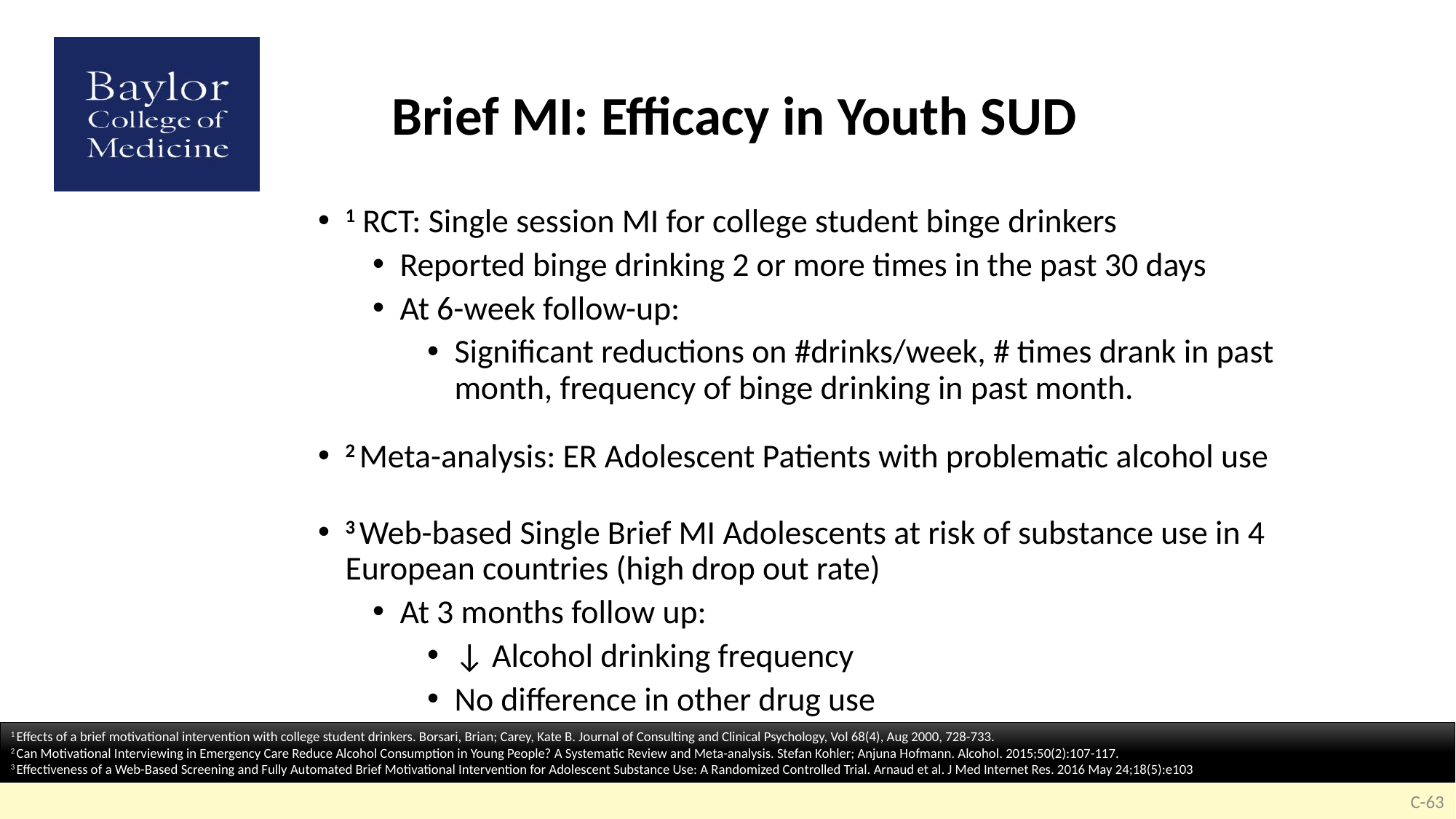

Brief MI: Efficacy in Youth SUD
1 RCT: Single session MI for college student binge drinkers
Reported binge drinking 2 or more times in the past 30 days
At 6-week follow-up:
Significant reductions on #drinks/week, # times drank in past month, frequency of binge drinking in past month.
2 Meta-analysis: ER Adolescent Patients with problematic alcohol use
3 Web-based Single Brief MI Adolescents at risk of substance use in 4 European countries (high drop out rate)
At 3 months follow up:
↓ Alcohol drinking frequency
No difference in other drug use
1 Effects of a brief motivational intervention with college student drinkers. Borsari, Brian; Carey, Kate B. Journal of Consulting and Clinical Psychology, Vol 68(4), Aug 2000, 728-733.
2 Can Motivational Interviewing in Emergency Care Reduce Alcohol Consumption in Young People? A Systematic Review and Meta-analysis. Stefan Kohler; Anjuna Hofmann. Alcohol. 2015;50(2):107-117.
3 Effectiveness of a Web-Based Screening and Fully Automated Brief Motivational Intervention for Adolescent Substance Use: A Randomized Controlled Trial. Arnaud et al. J Med Internet Res. 2016 May 24;18(5):e103
C-63

## Slide 64
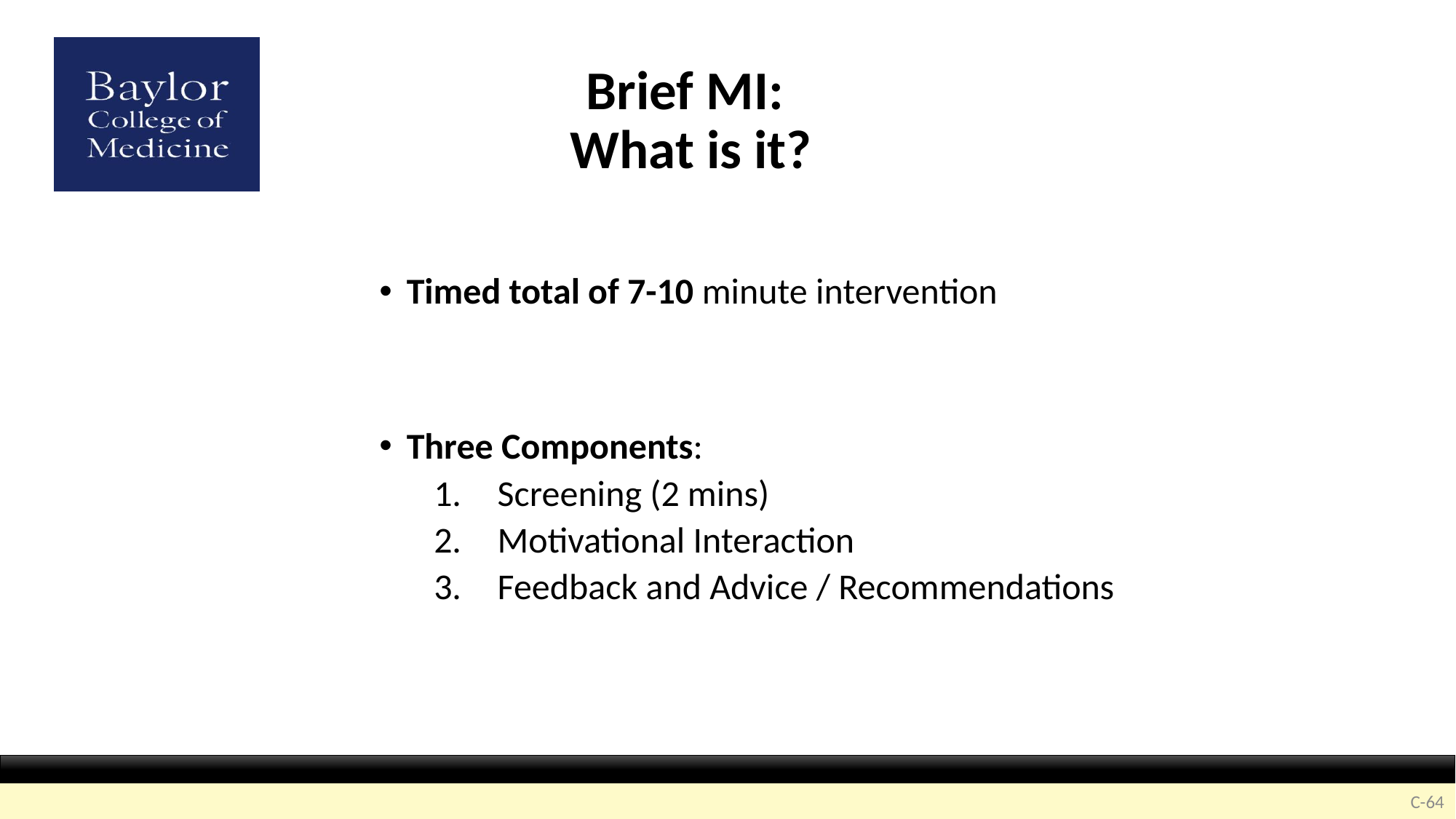

Brief MI:
What is it?
Timed total of 7-10 minute intervention
Three Components:
Screening (2 mins)
Motivational Interaction
Feedback and Advice / Recommendations
C-64

## Slide 65
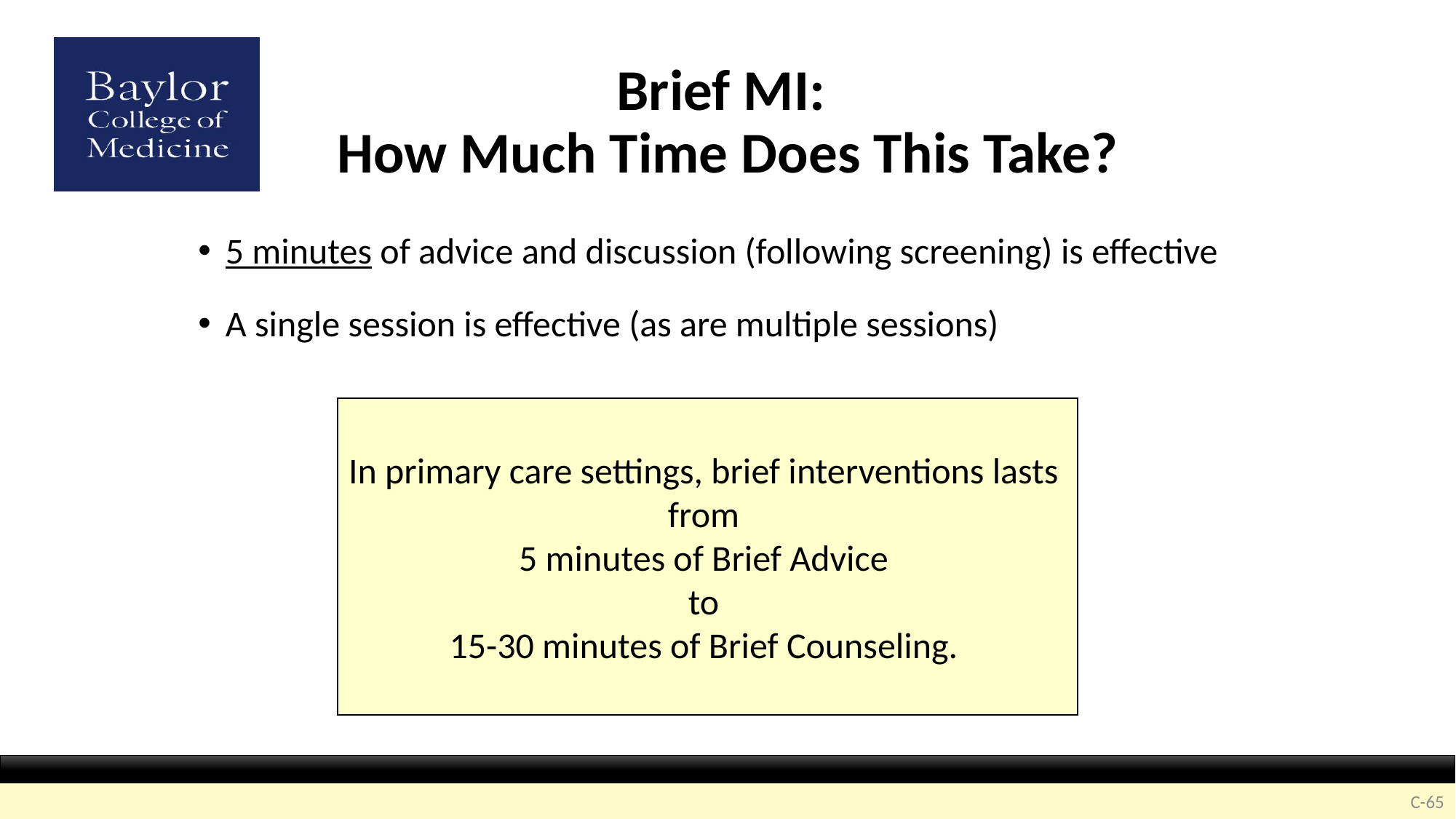

Brief MI:
How Much Time Does This Take?
5 minutes of advice and discussion (following screening) is effective
A single session is effective (as are multiple sessions)
In primary care settings, brief interventions lasts
from
5 minutes of Brief Advice
to
15-30 minutes of Brief Counseling.
C-65

## Slide 66
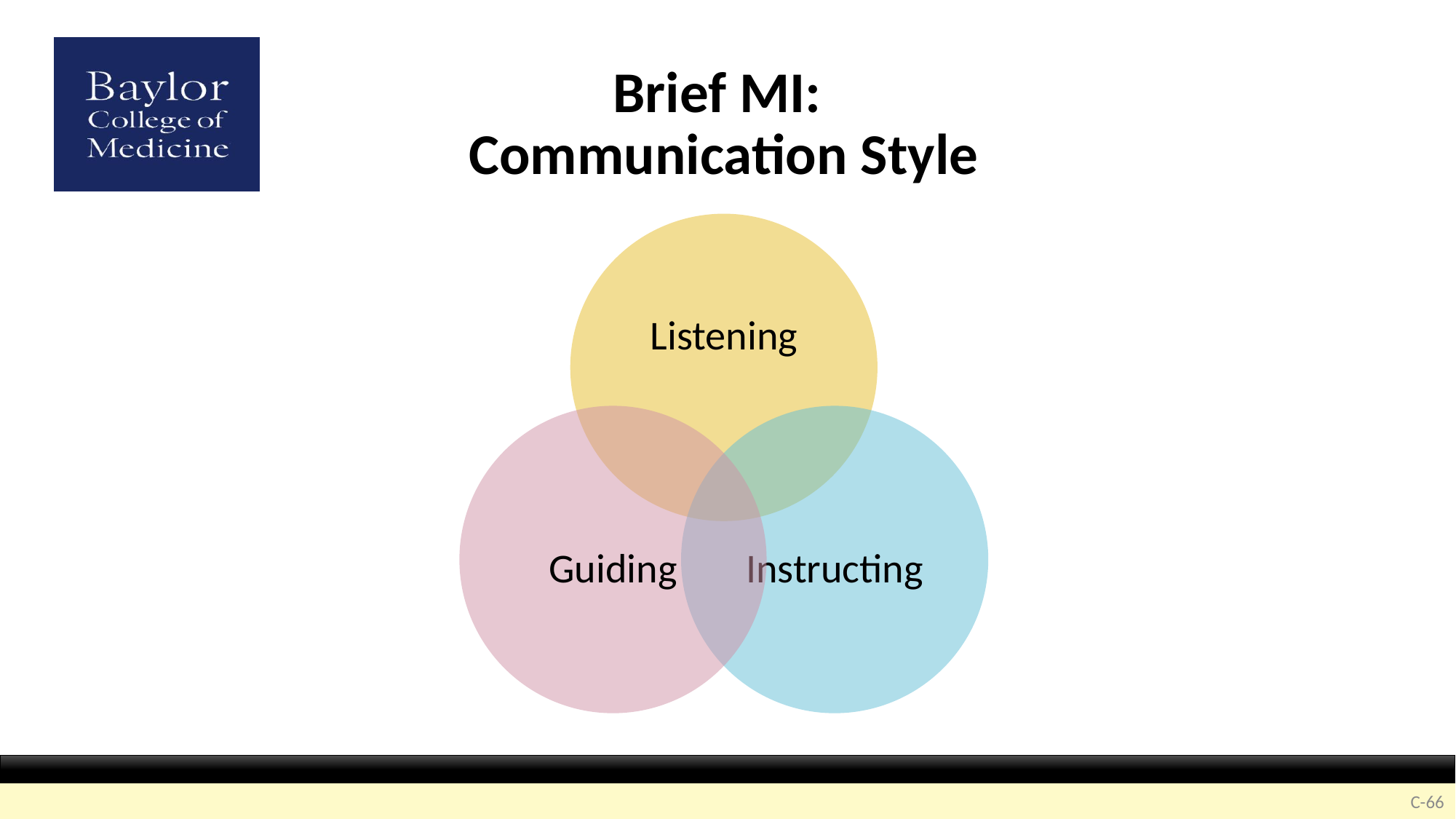

Brief MI:
Communication Style
C-66

## Slide 67
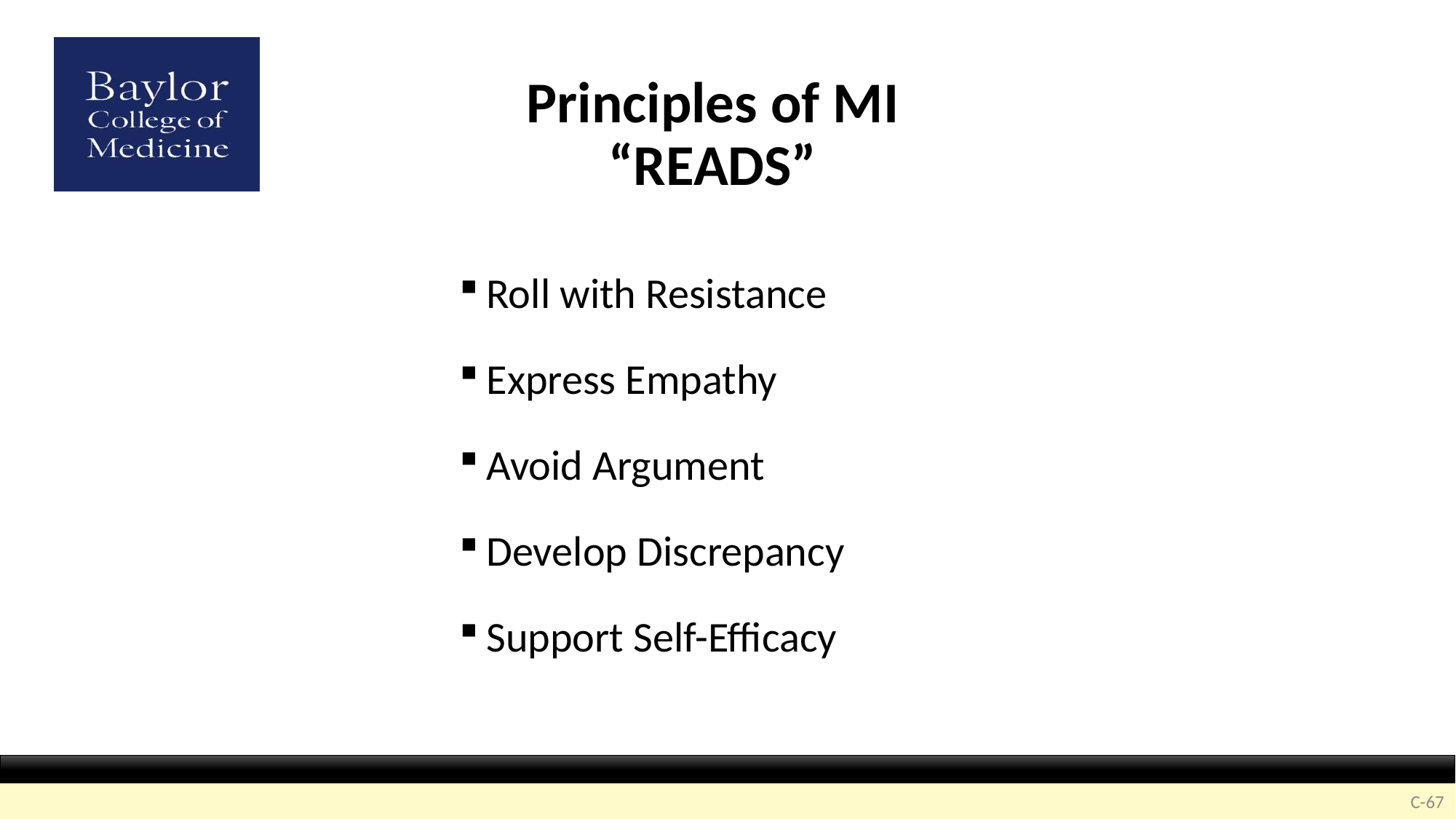

Principles of MI
“READS”
Roll with Resistance
Express Empathy
Avoid Argument
Develop Discrepancy
Support Self-Efficacy
C-67

## Slide 68
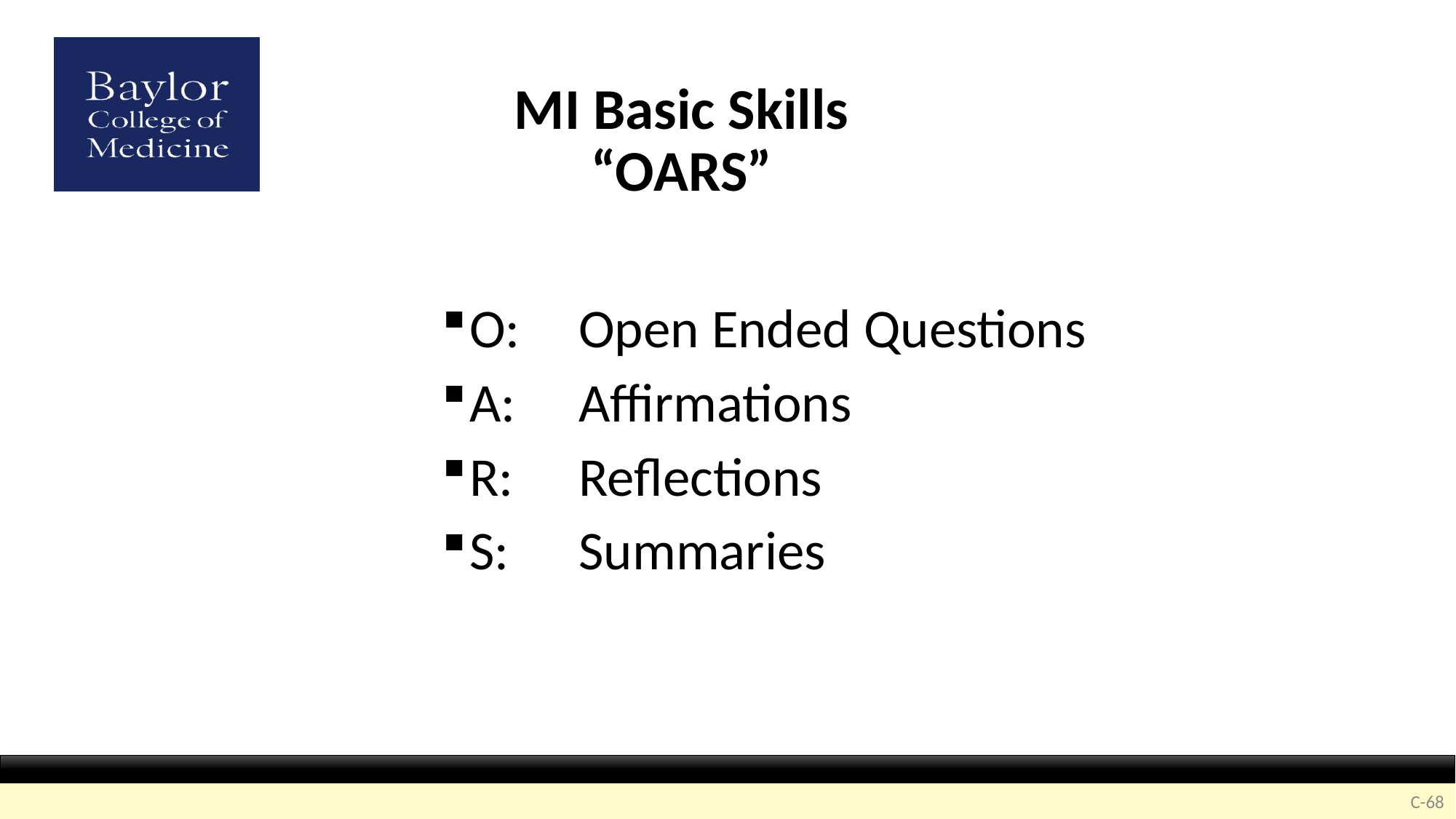

MI Basic Skills
“OARS”
O: 	Open Ended Questions
A:	Affirmations
R:	Reflections
S:	Summaries
C-68

## Slide 69
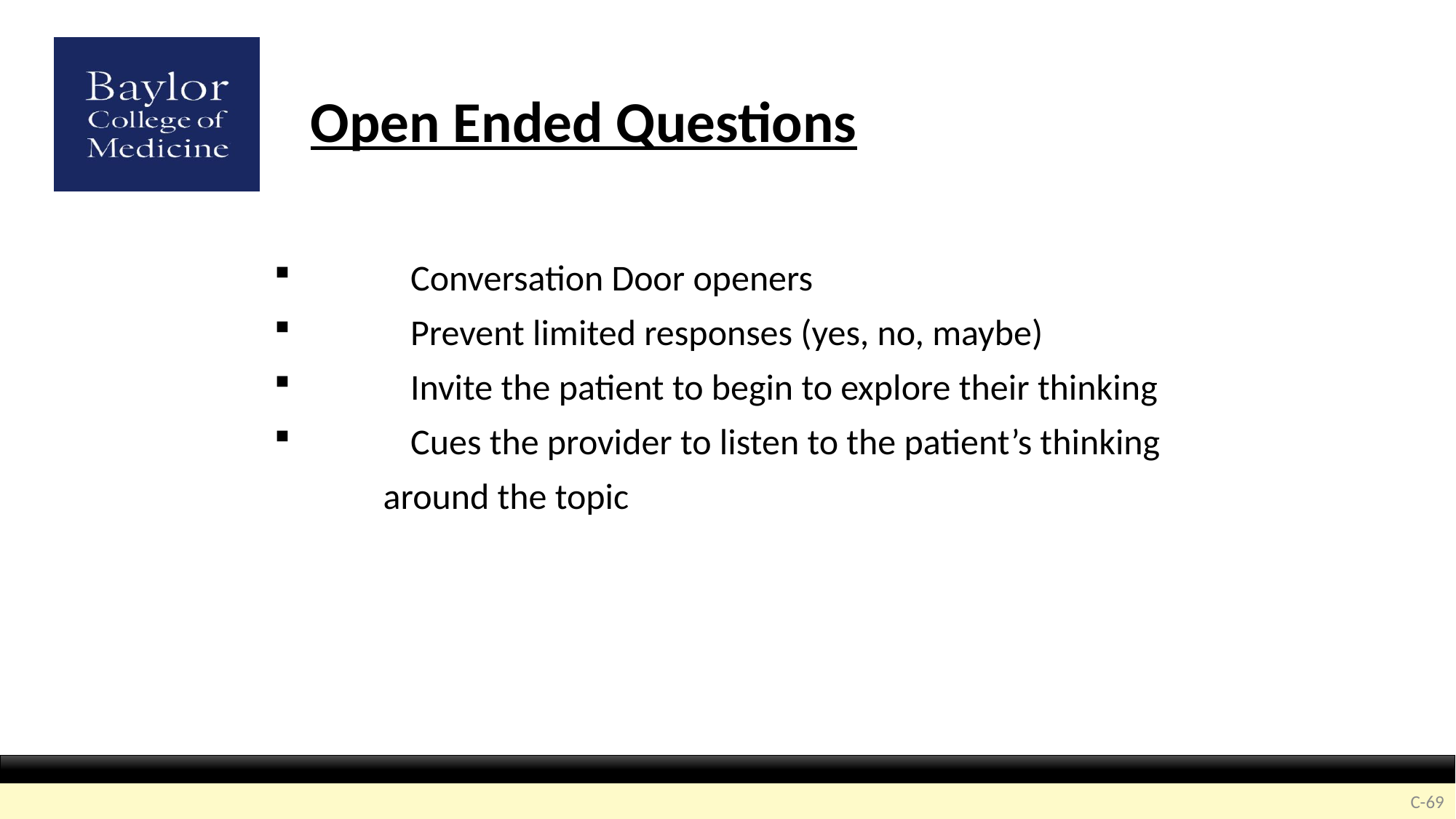

Open Ended Questions
	Conversation Door openers
	Prevent limited responses (yes, no, maybe)
	Invite the patient to begin to explore their thinking
	Cues the provider to listen to the patient’s thinking
	around the topic
C-69

## Slide 70
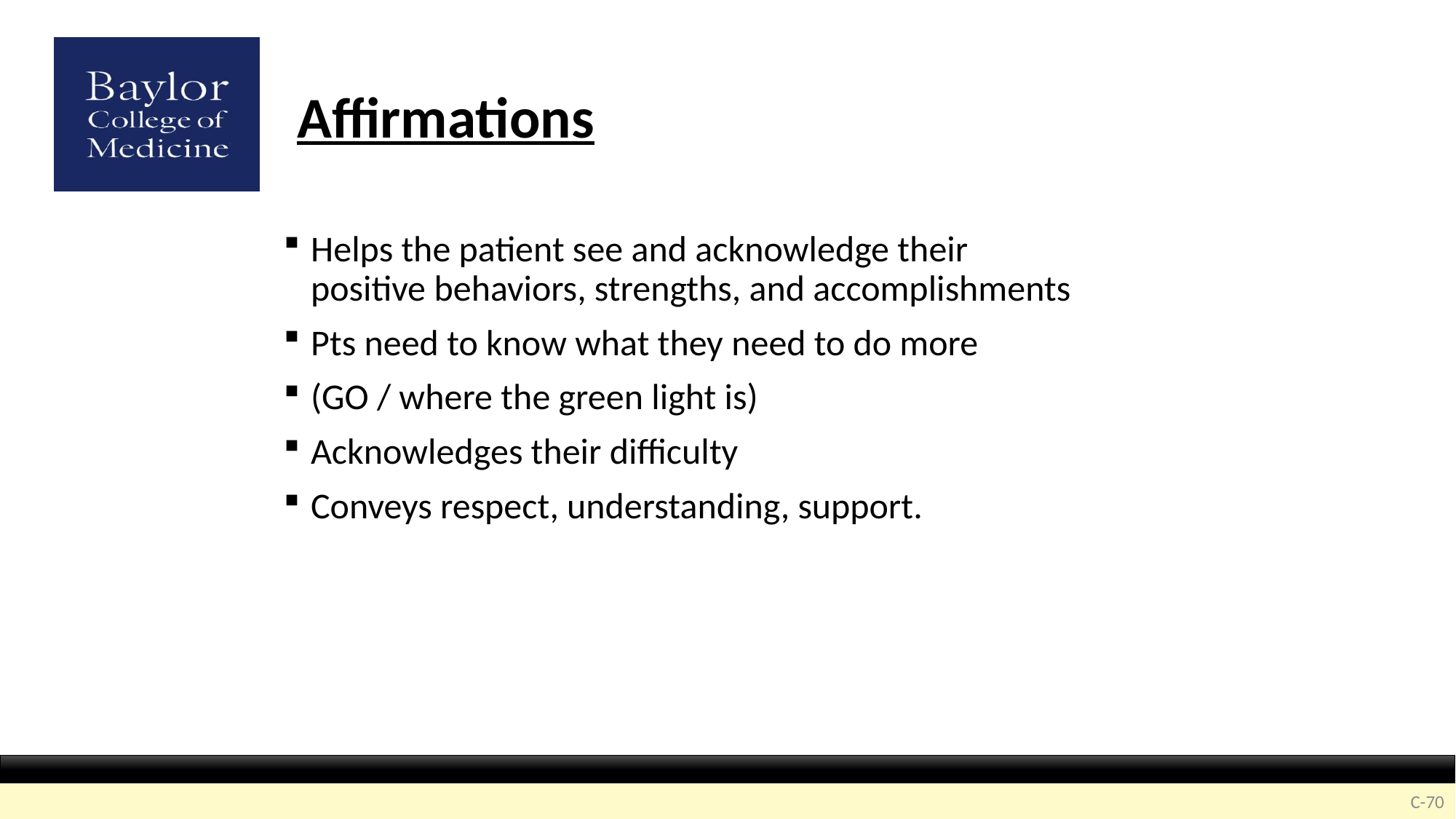

Affirmations
Helps the patient see and acknowledge their positive behaviors, strengths, and accomplishments
Pts need to know what they need to do more
(GO / where the green light is)
Acknowledges their difficulty
Conveys respect, understanding, support.
C-70

## Slide 71
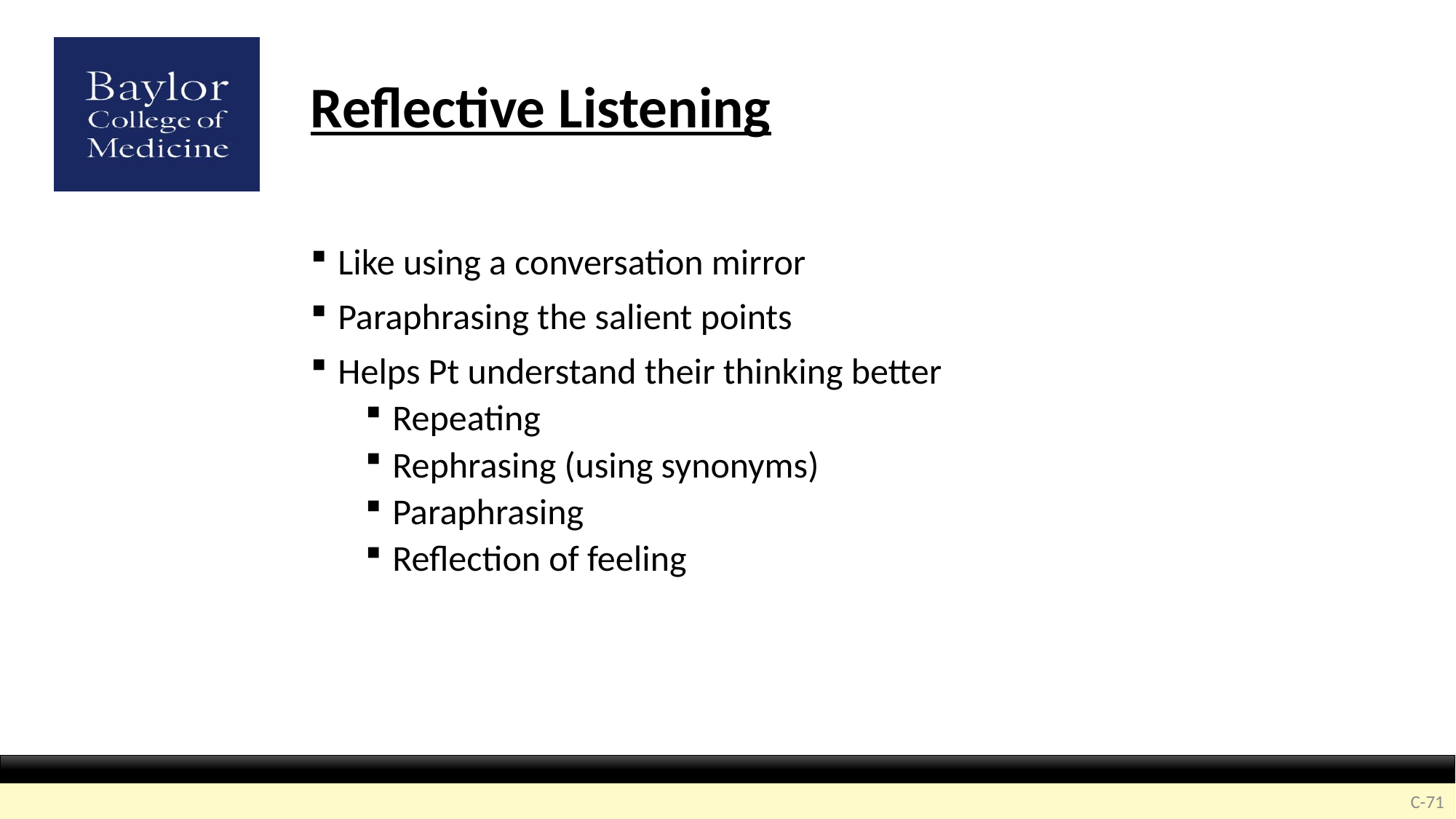

Reflective Listening
Like using a conversation mirror
Paraphrasing the salient points
Helps Pt understand their thinking better
Repeating
Rephrasing (using synonyms)
Paraphrasing
Reflection of feeling
C-71

## Slide 72
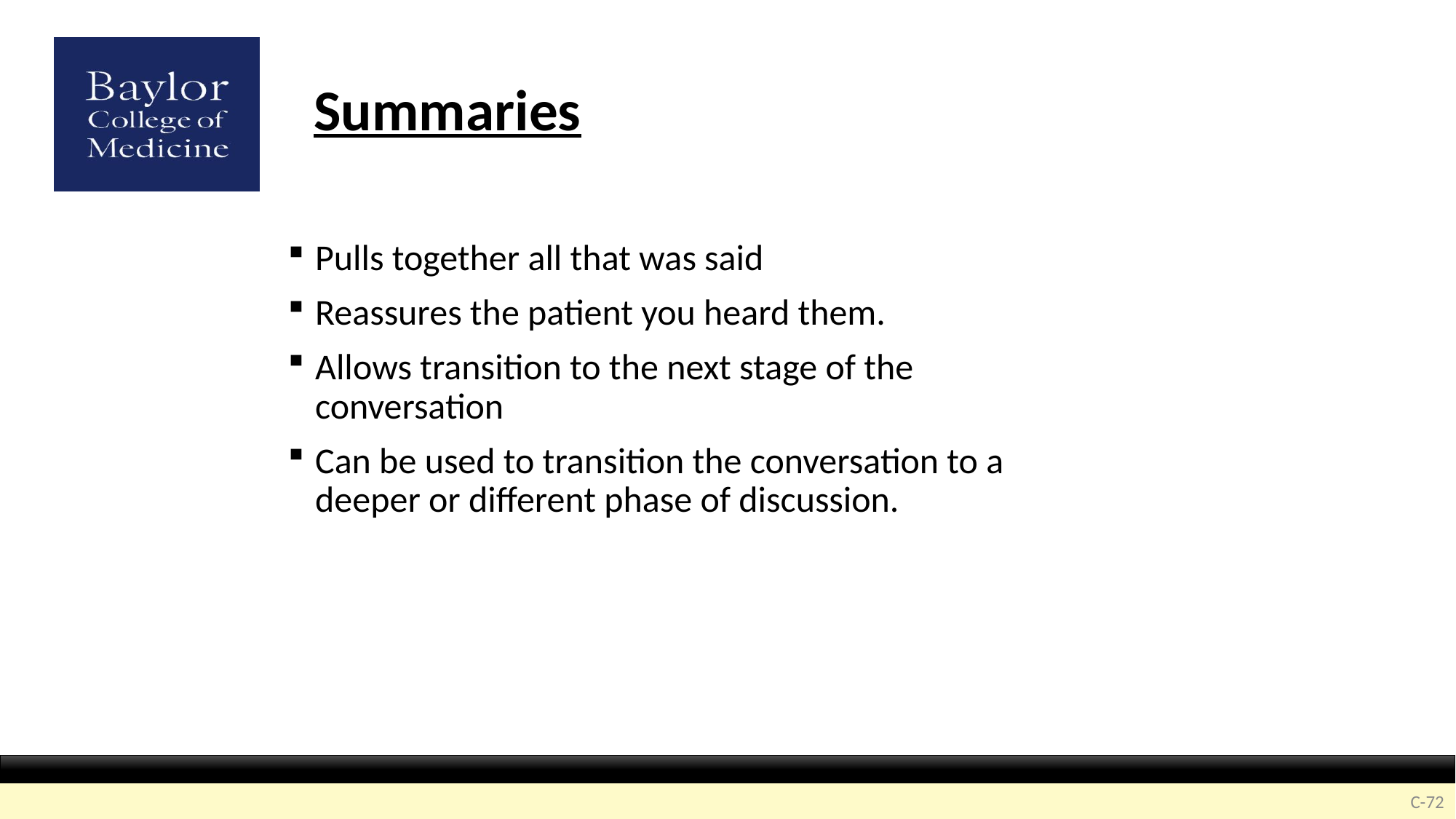

Summaries
Pulls together all that was said
Reassures the patient you heard them.
Allows transition to the next stage of the conversation
Can be used to transition the conversation to a deeper or different phase of discussion.
C-72

## Slide 73
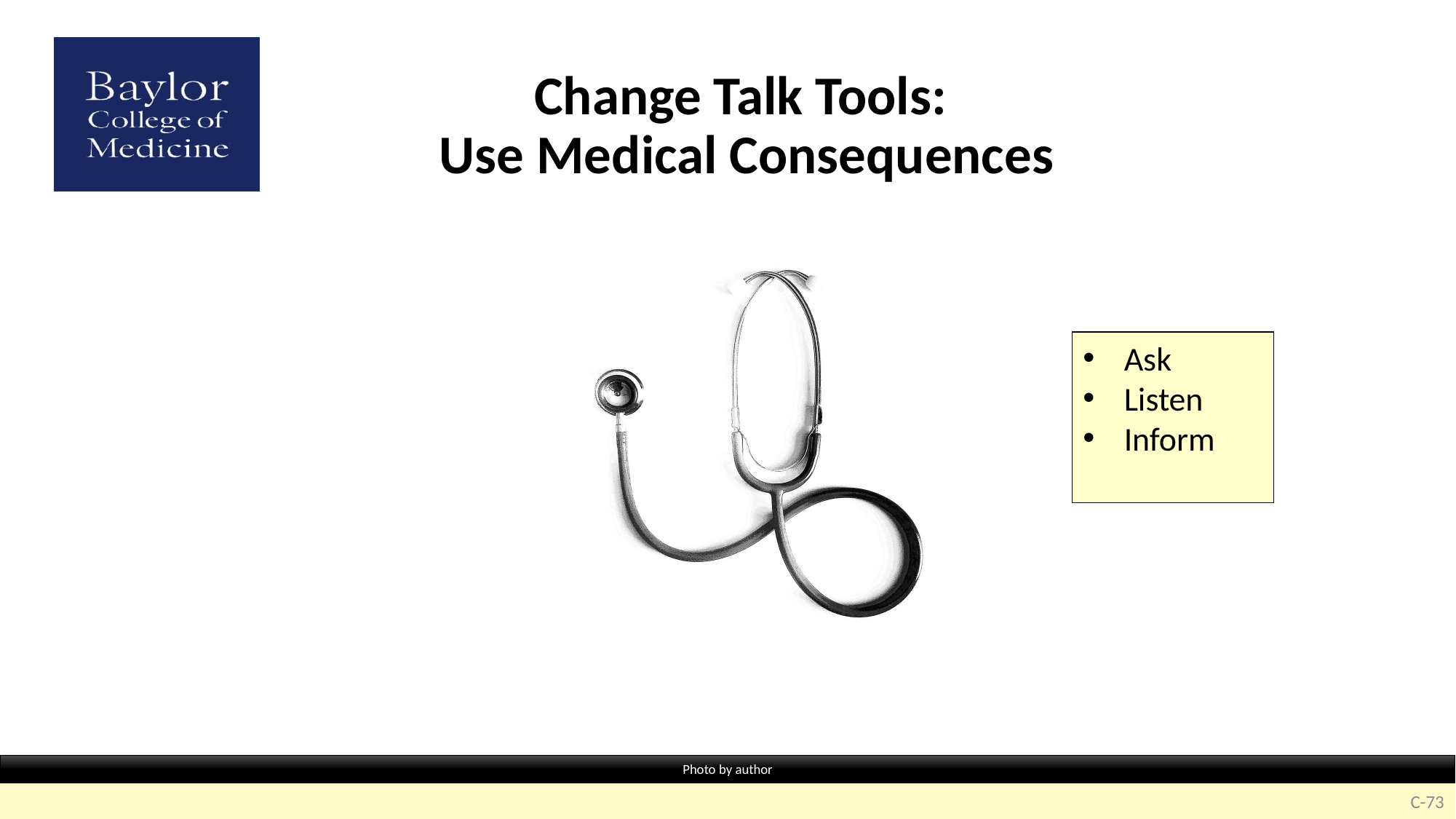

Change Talk Tools:
Use Medical Consequences
Ask
Listen
Inform
Photo by author
C-73

## Slide 74
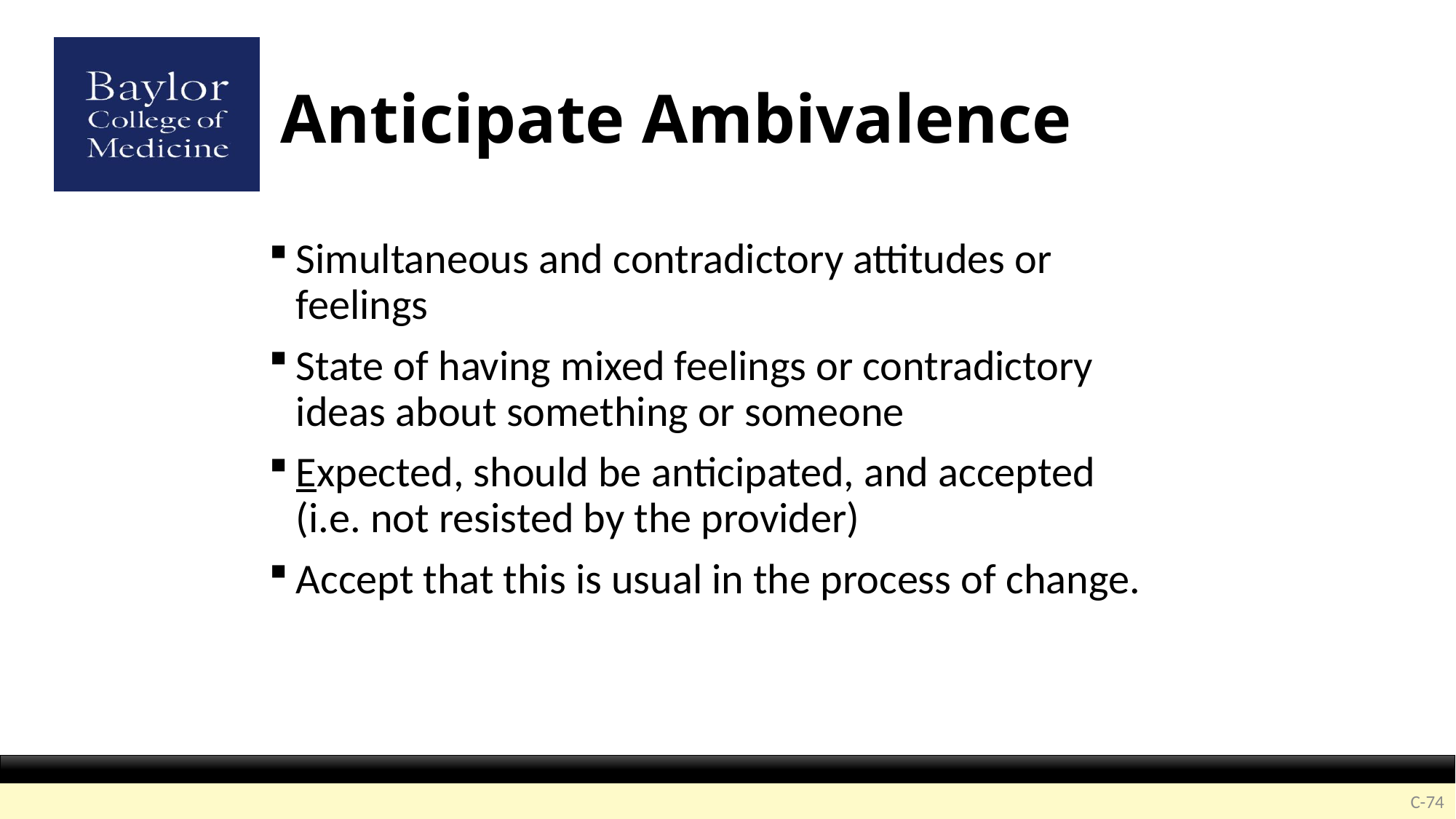

Anticipate Ambivalence
Simultaneous and contradictory attitudes or feelings
State of having mixed feelings or contradictory ideas about something or someone
Expected, should be anticipated, and accepted (i.e. not resisted by the provider)
Accept that this is usual in the process of change.
C-74

## Slide 75
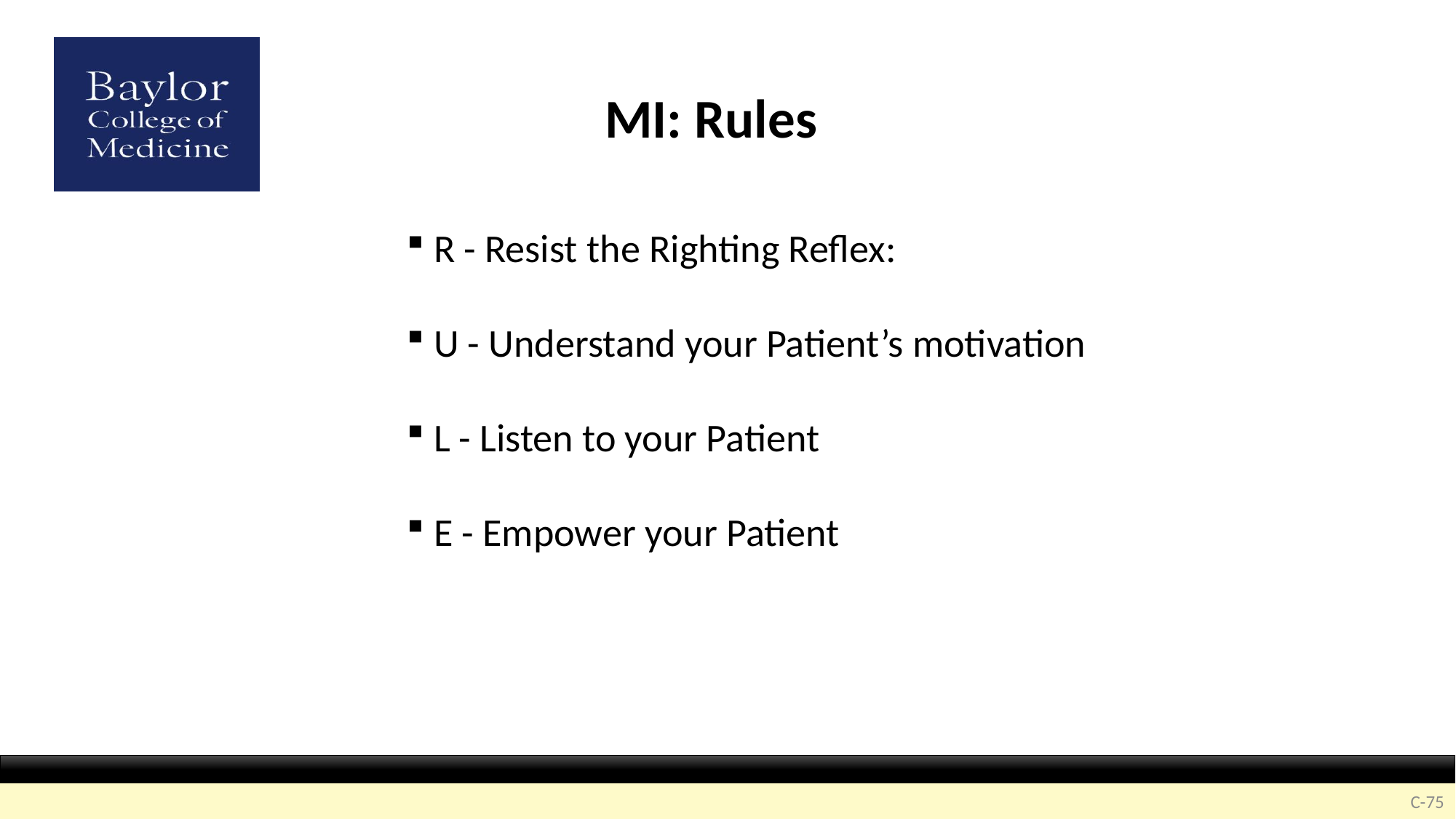

MI: Rules
R - Resist the Righting Reflex:
U - Understand your Patient’s motivation
L - Listen to your Patient
E - Empower your Patient
C-75

## Slide 76
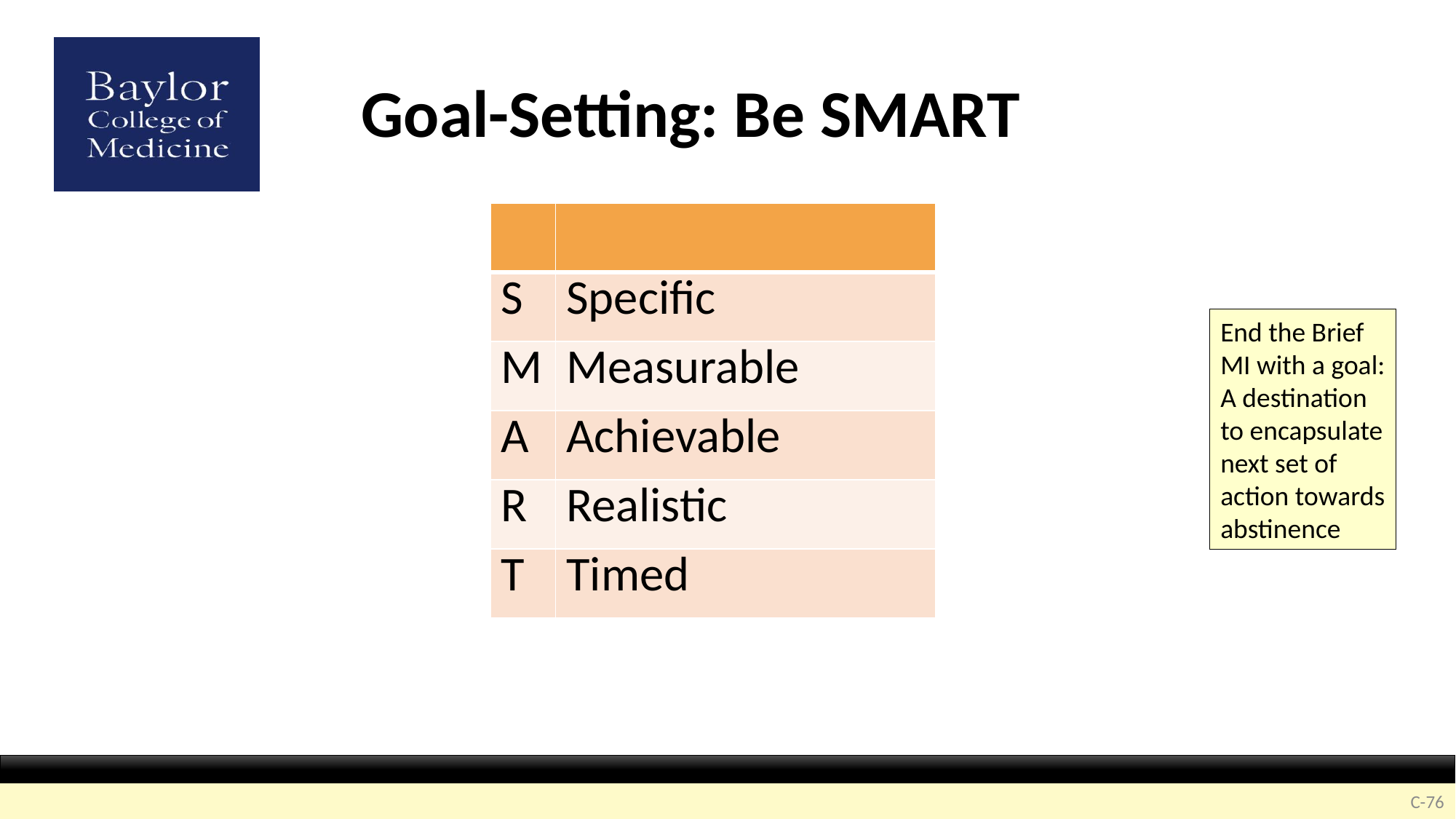

Goal-Setting: Be SMART
| | |
| --- | --- |
| S | Specific |
| M | Measurable |
| A | Achievable |
| R | Realistic |
| T | Timed |
End the Brief MI with a goal: A destination to encapsulate next set of action towards abstinence
C-76

## Slide 77
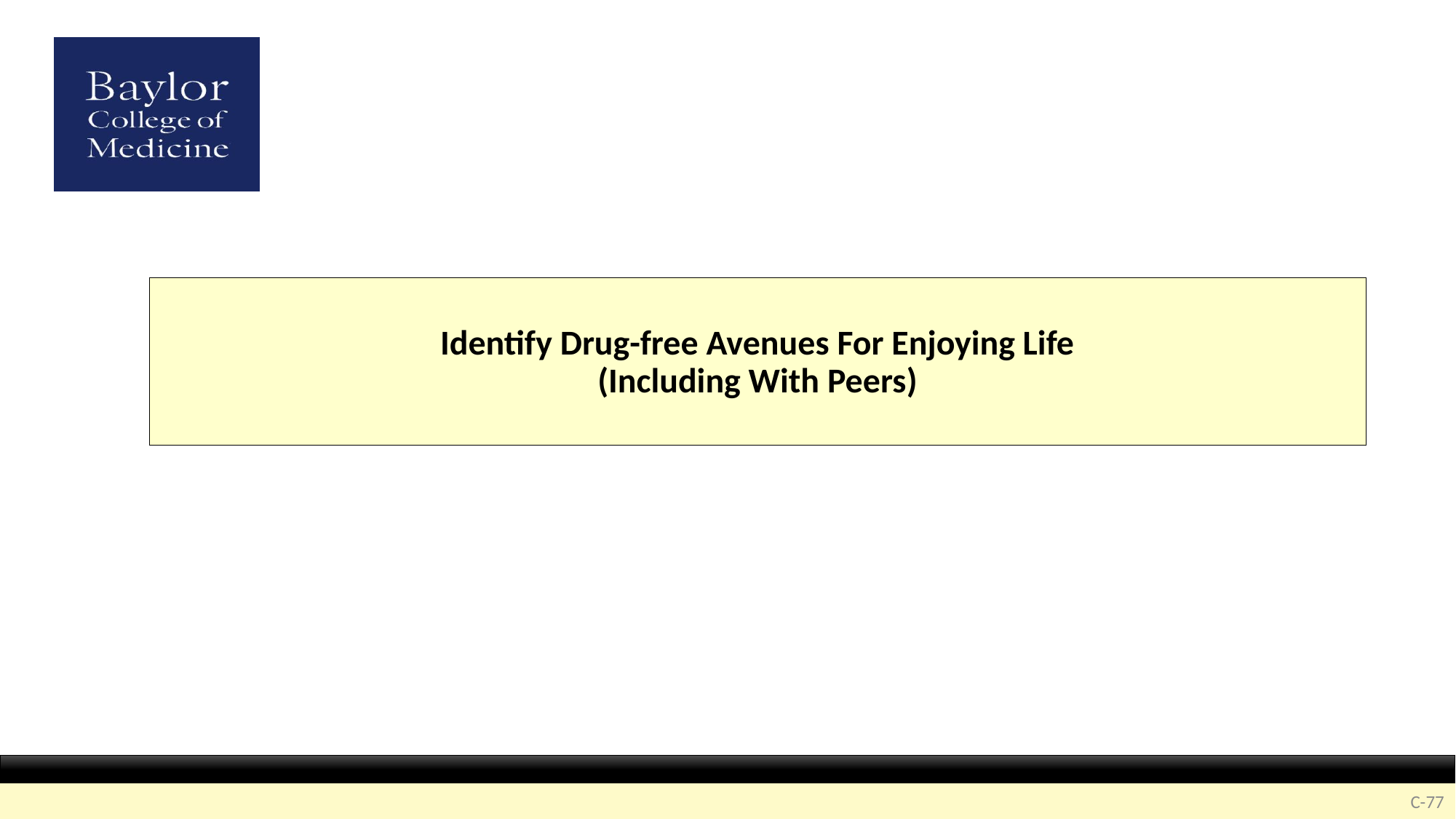

Identify Drug-free Avenues For Enjoying Life
(Including With Peers)
C-77

## Slide 78
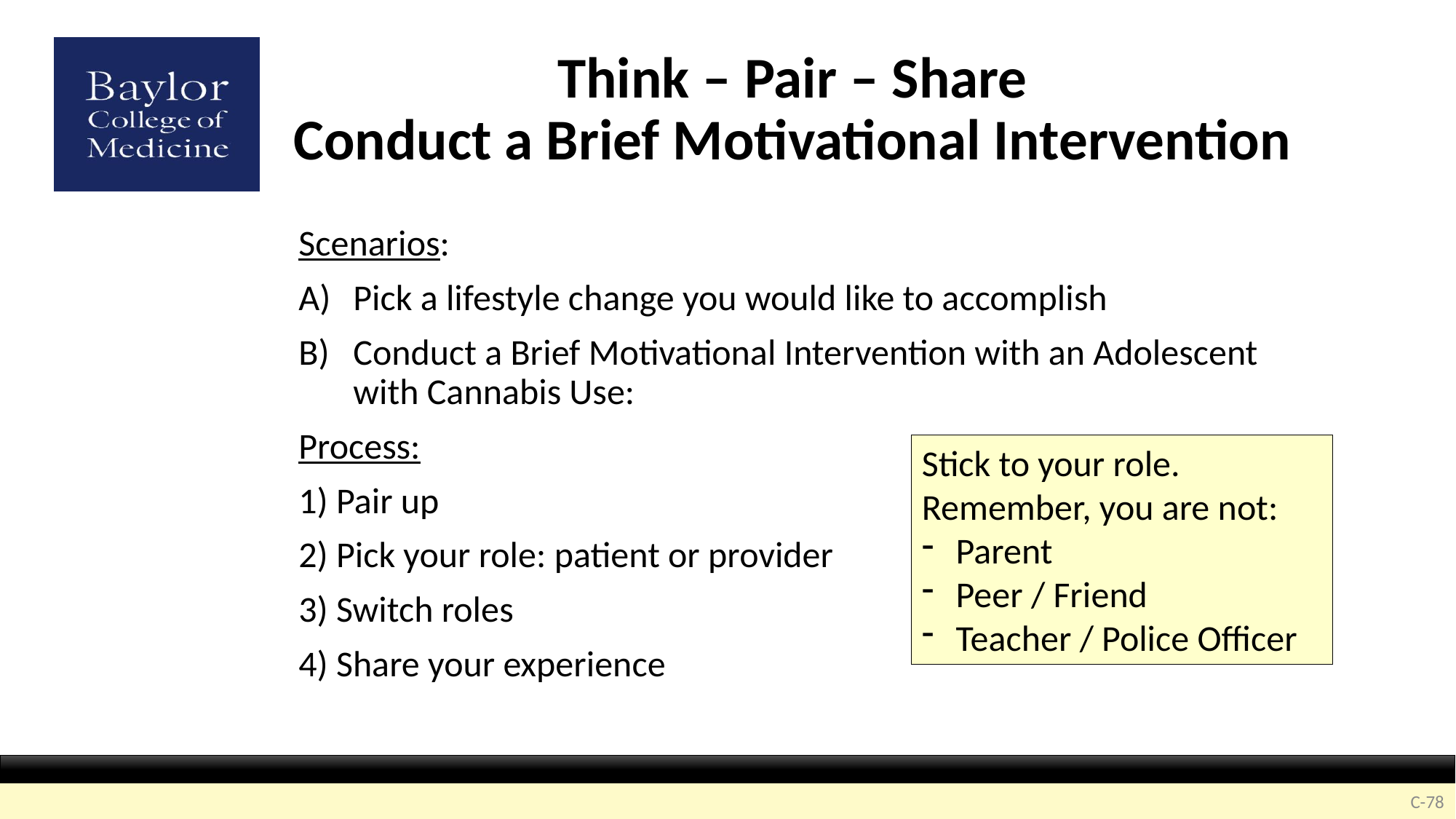

Think – Pair – Share
Conduct a Brief Motivational Intervention
Scenarios:
Pick a lifestyle change you would like to accomplish
Conduct a Brief Motivational Intervention with an Adolescent with Cannabis Use:
Process:
1) Pair up
2) Pick your role: patient or provider
3) Switch roles
4) Share your experience
Stick to your role. Remember, you are not:
Parent
Peer / Friend
Teacher / Police Officer
C-78

## Slide 79
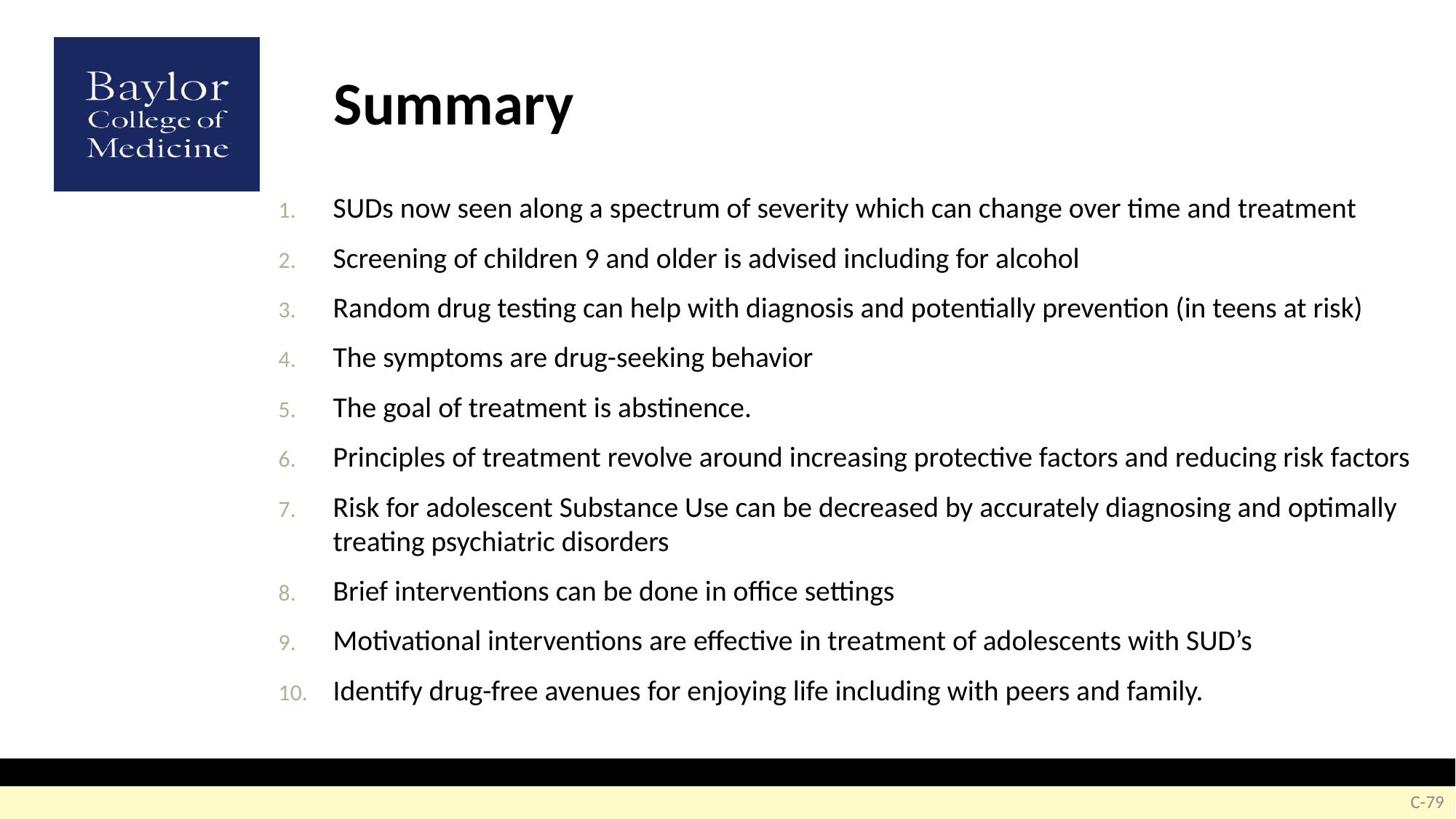

Summary
SUDs now seen along a spectrum of severity which can change over time and treatment
Screening of children 9 and older is advised including for alcohol
Random drug testing can help with diagnosis and potentially prevention (in teens at risk)
The symptoms are drug-seeking behavior
The goal of treatment is abstinence.
Principles of treatment revolve around increasing protective factors and reducing risk factors
Risk for adolescent Substance Use can be decreased by accurately diagnosing and optimally treating psychiatric disorders
Brief interventions can be done in office settings
Motivational interventions are effective in treatment of adolescents with SUD’s
Identify drug-free avenues for enjoying life including with peers and family.
C-79

## Slide 80
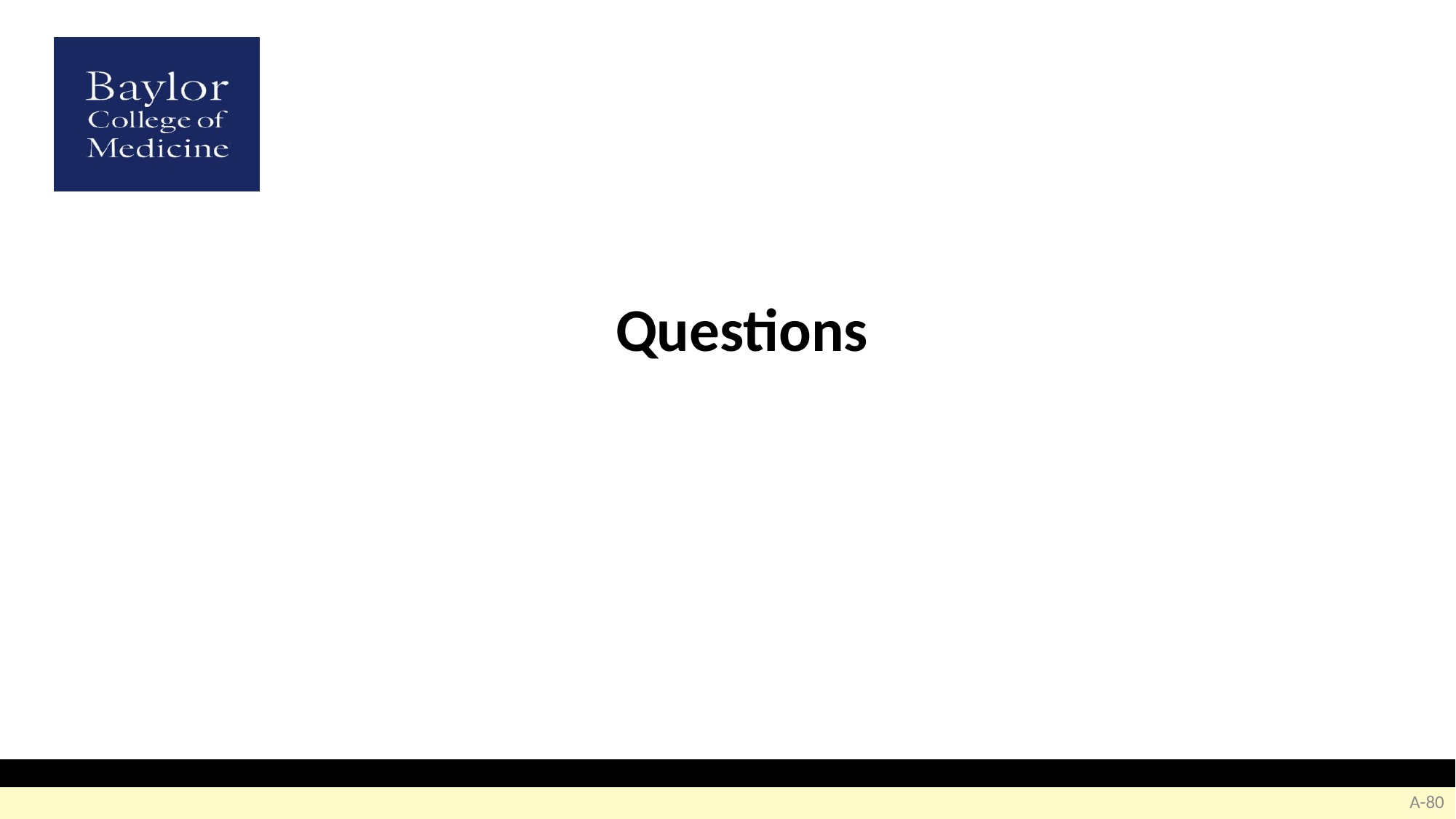

Questions
A-80

## Slide 81
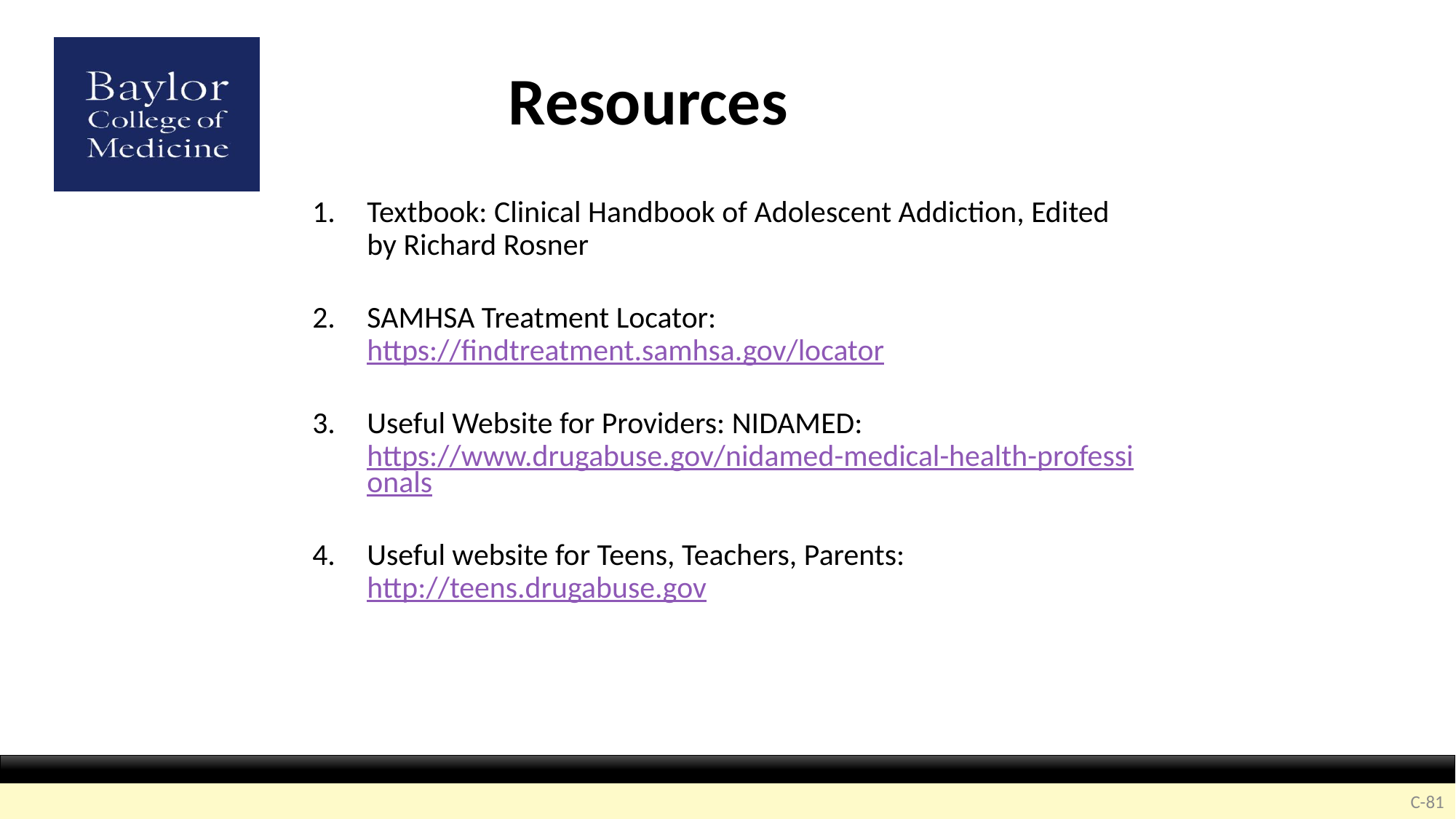

Resources
Textbook: Clinical Handbook of Adolescent Addiction, Edited by Richard Rosner
SAMHSA Treatment Locator: https://findtreatment.samhsa.gov/locator
Useful Website for Providers: NIDAMED: https://www.drugabuse.gov/nidamed-medical-health-professionals
Useful website for Teens, Teachers, Parents: http://teens.drugabuse.gov
C-81
